# Supplementary material for: Interpretable and context-free deconvolution of multi-scale whole transcriptomic data with UniCell deconvolve
Source: Nat Commun. 2023 Mar 11;14:1350. doi: 10.1038/s41467-023-36961-8 (PMC10008582; doi:10.1038/s41467-023-36961-8)
Supplement: Supplementary file 1 — Supplementary Information [file 41467_2023_36961_MOESM1_ESM.pdf]

Supplementary Figures

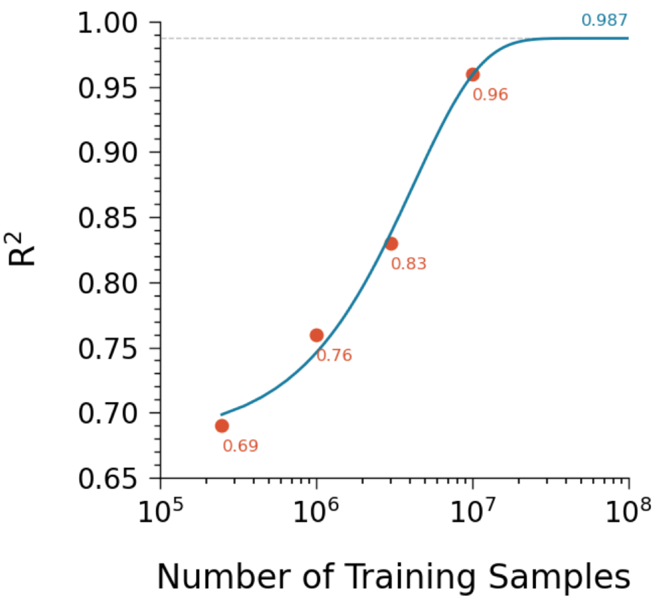

Supplementary Figure 1. Estimating Maximum Theoretical Performance of UniCell Deconvolve. Exponential curve (blue) fit to four peak performance measures for Unicell Deconvolve Base (UCDBase) models trained on varying numbers of samples (250K, 1M, 3M, and 10M samples). Y-axis represents the model coefficient of determination (R^2) as a measure of deconvolution accuracy, while the x-axis represents the number of training mixture samples.

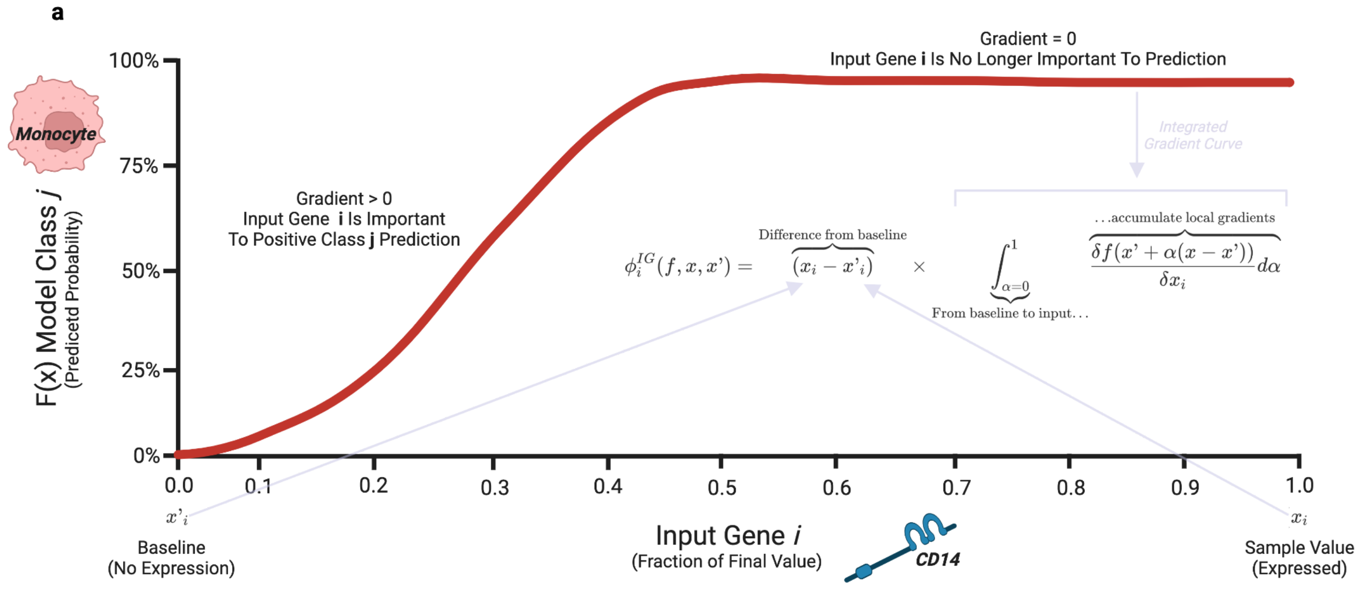

$$\phi_i^{IG}(f, x, x') = \overbrace{(x_i - x'_i)}^{\text{Difference from baseline}} \times \underbrace{\int_{\alpha=0}^{1}}_{\text{From baseline to input...}} \overbrace{\frac{\delta f(x' + \alpha(x - x'))}{\delta x_i}}^{\text{...accumulate local gradients}} d\alpha$$

Supplementary Figure 2. Intuition Behind Integrated Gradients For Cell Type Prediction Using Deep Neural Networks. Demonstrative line plot showing the effect of increasing the value of an input gene, for example CD14, from a zero-like baseline to its final expression value within a given sample. As the expression level of CD14 is increased within the sample, the model's predicted probability of a given cell type, in this case monocytes, is recorded. In this case, increased expression of CD14 is associated with an increased probability of monocytes being present in the sample, which would yield a positive integrated gradient.

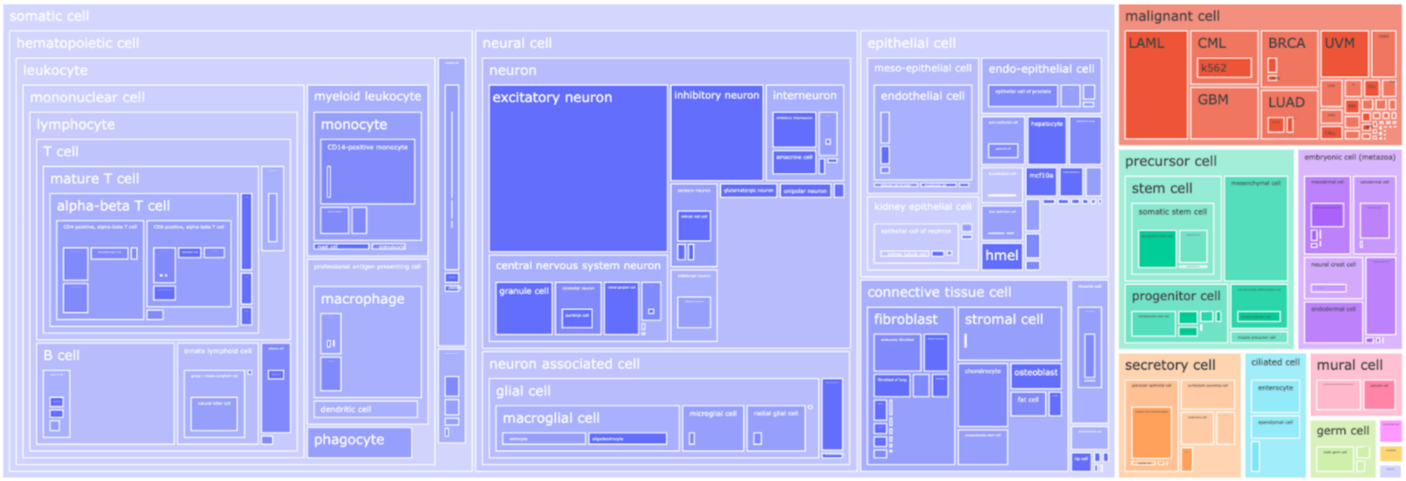

Supplementary Figure 3. UniCell Single Cell Database Visualized. A nested rectangle visualization of the cell type distribution hierarchy for over 28 million single cells comprising the UniCell Deconvolve Base (UCDBase) training dataset.

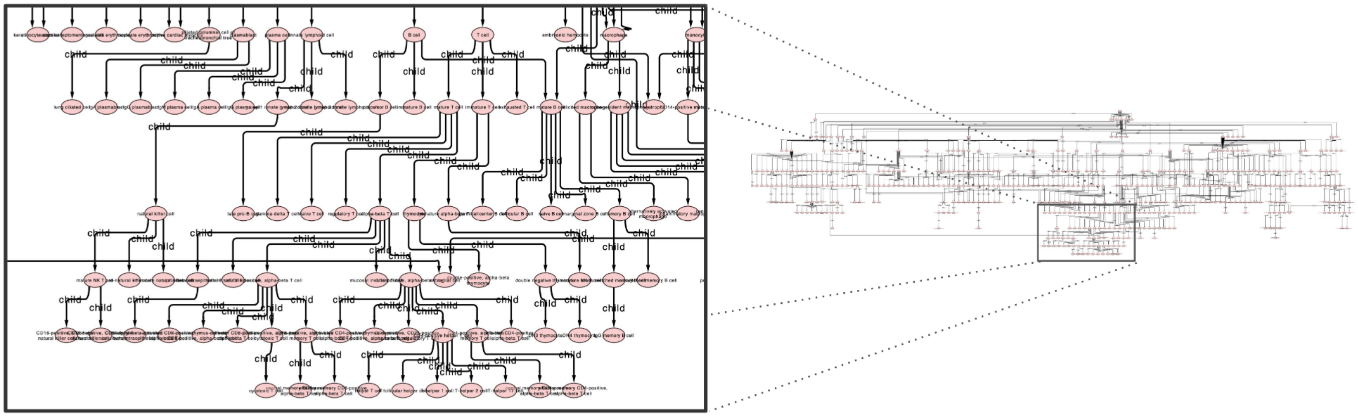

Supplementary Figure 4. Cell Ontology For Belief Propagation. Network visualization of manually-curated cell type hierarchy (right). A section is highlighted and zoomed in as a demonstration showing several t cell subtypes (left).

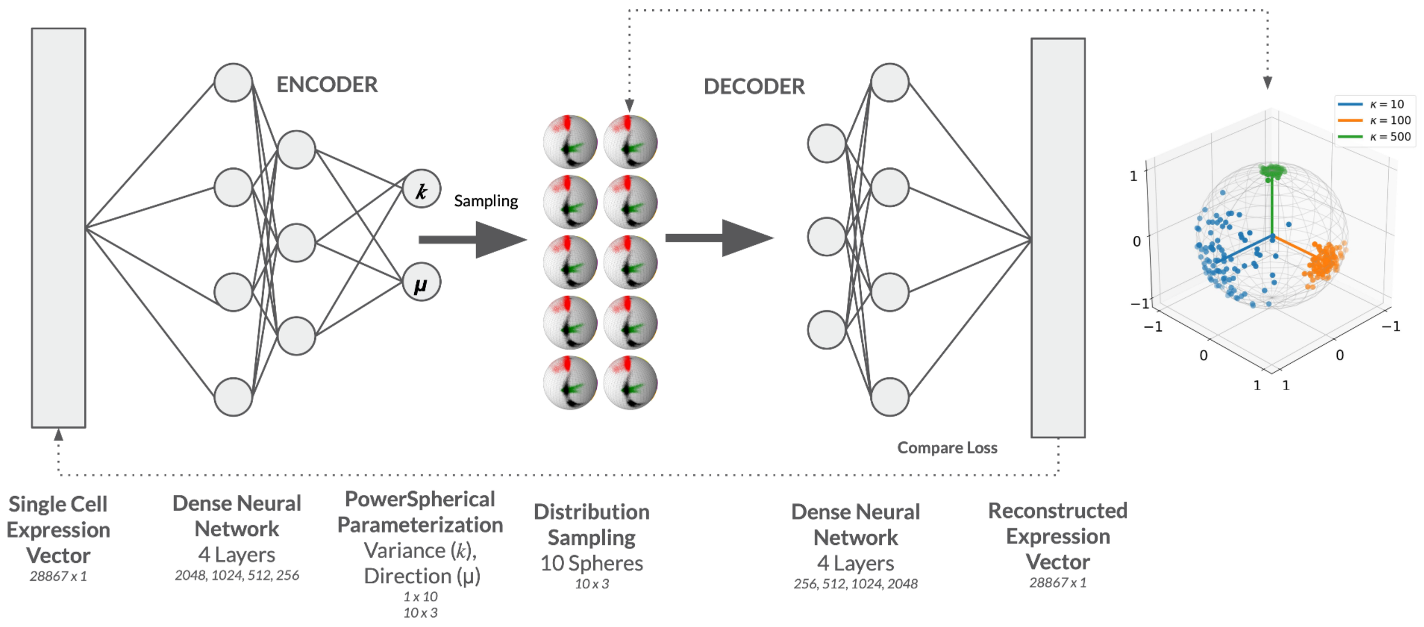

Supplementary Figure 5. Spherical Variational Autoencoder Framework. Overview of architecture for Spherical Variational Autoencoder (SVAE) used to generate cell embeddings and support guided label transfer strategy for training data construction. Key hyperparameters of the model are indicated in the architecture diagram.

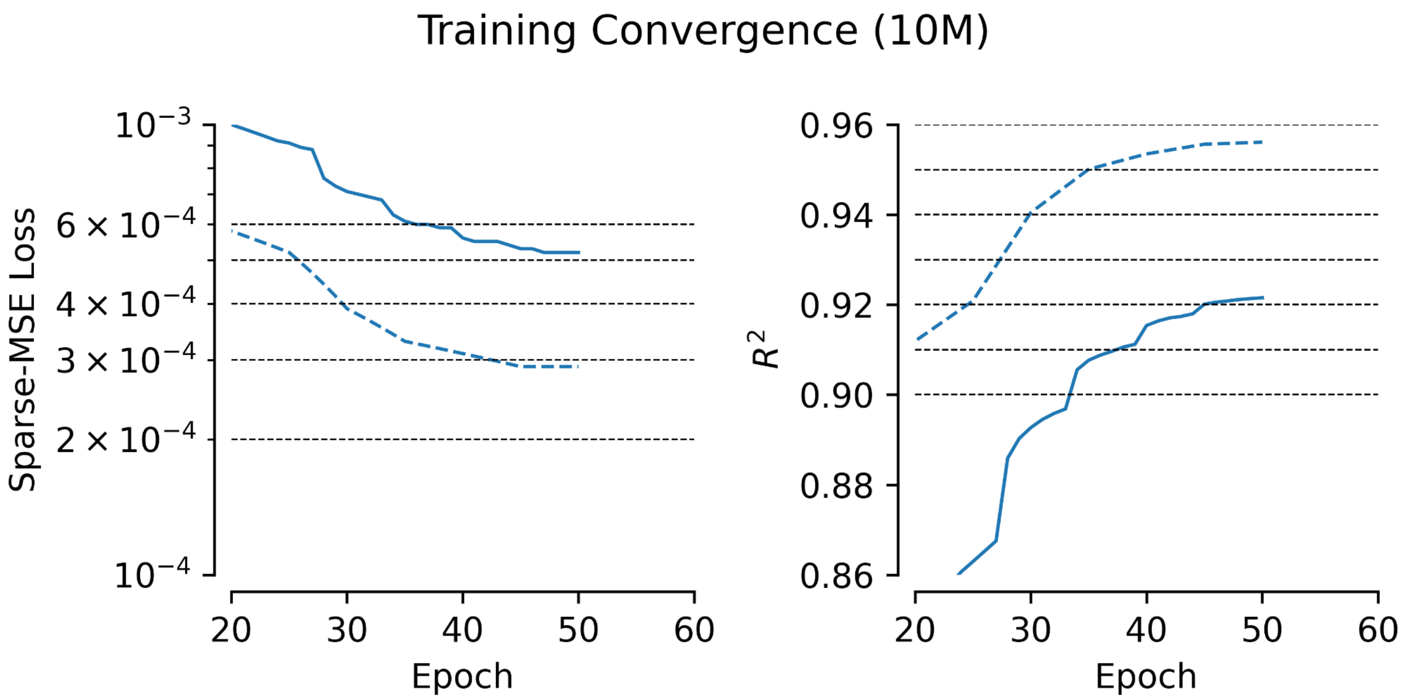

Supplementary Figure 6. Validation of Model Convergence. (left) Loss function (Sparse-MSE, y-axis) as a function of training epoch (x-axis) for the last 30 epochs of model training is shown with a log-scaled y-axis to visually confirm model convergence. (right) Model accuracy as measured by coefficient of determination (R^2) is shown for the final 30 epochs (Source Data File - Supp. Fig 1F).

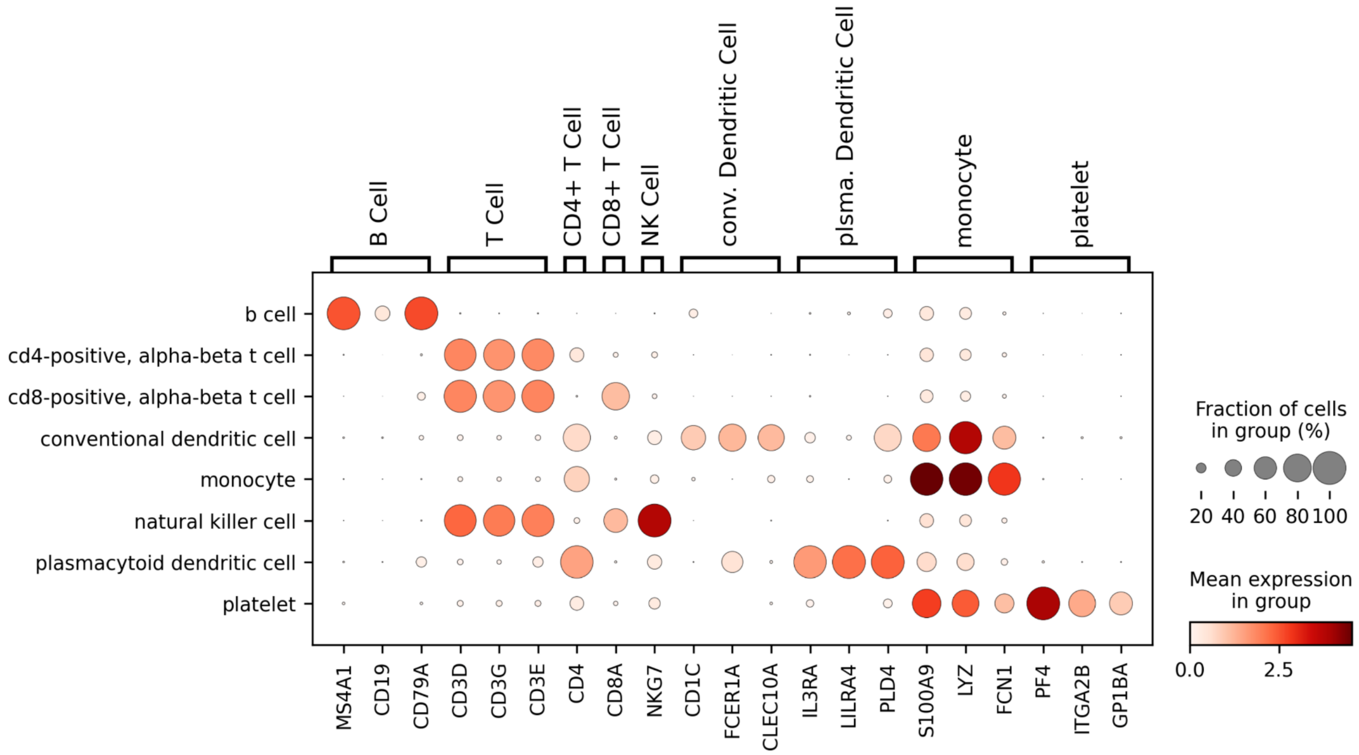

Supplementary Figure 7. Canonical Marker Genes Used to Annotate Cell Type Clusters. Dot plot highlighting expression of canonical marker genes (x-axis) across clusters labeled by cell type (y-axis) for *human* peripheral blood mononuclear (PBMC) single cell dataset used to generate pseudobulk mixtures for benchmarking UniCell performance.

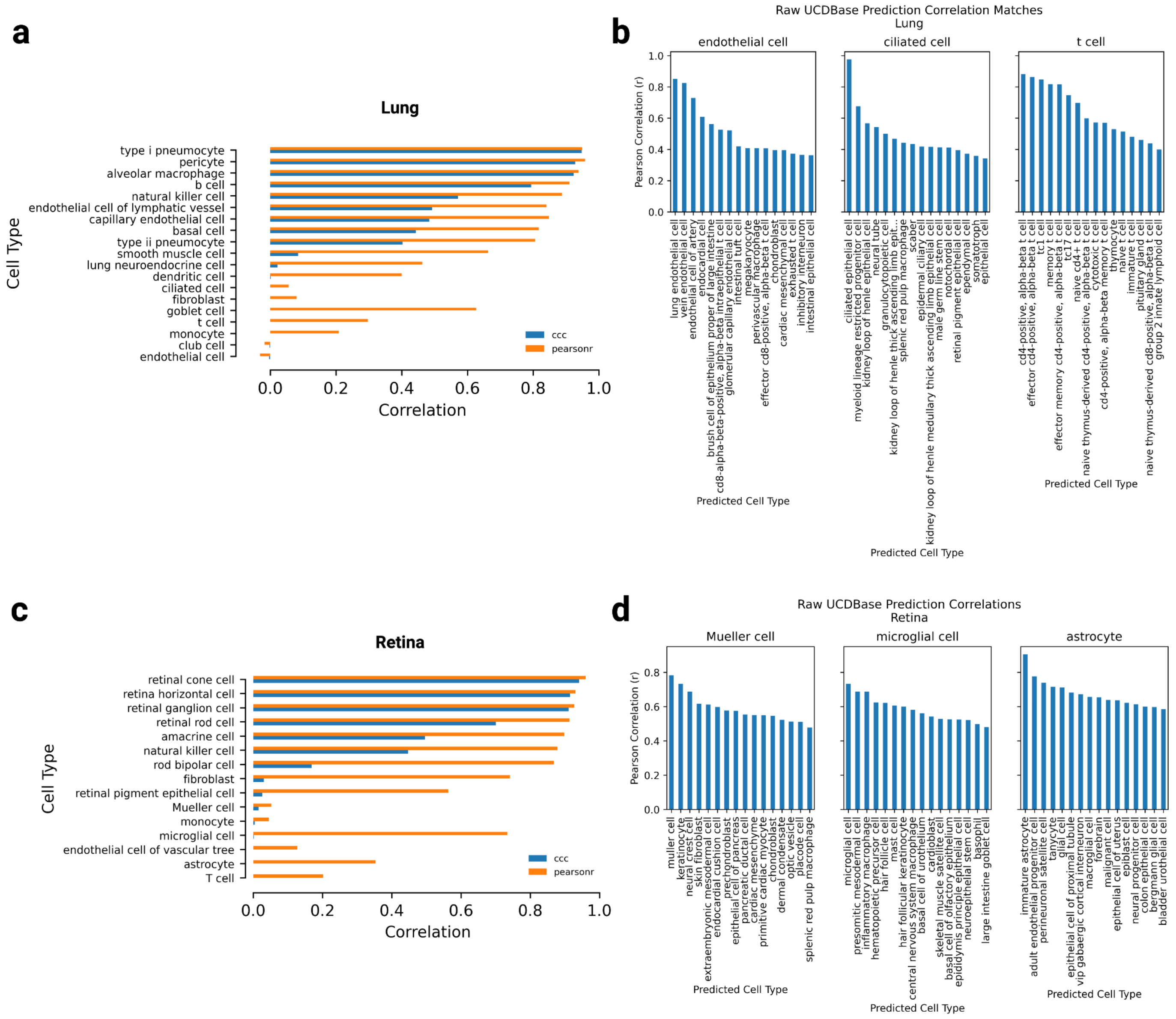

Supplementary Figure 8. Assessing Label Mismatch Between Target Datasets and UCDBase Annotations. a) Raw non propagated UniCell Deconvolve Base (UCDBase) prediction results for lung mixture benchmark dataset, measured by pearson correlation (orange) and concordance correlation coefficient (CCC, blue), are plotted (x-axis) as barplots for each cell type (y-axis) (Source Data File - Supp. Fig 2B-a). **b)** Top 16 cell types (x-axis) based on their correlation (y-axis) with the target cell type (title) in the lung mixture dataset are shown as barplots (Source Data File - Supp. Fig 2B-b). **c)** Raw non propagated UniCell Deconvolve Base (UCDBase) prediction results for retina mixture benchmark dataset, measured by pearson correlation (orange) and concordance correlation coefficient (CCC, blue), are plotted (x-axis) as barplots for each cell type (y-axis) (Source Data File - Supp. Fig 2B-c). **d)** Top 16 cell types (x-axis) based on their correlation (y-axis) with the target cell type (title) in the retina mixture dataset are shown as barplots (Source Data File - Supp. Fig 2B-d).

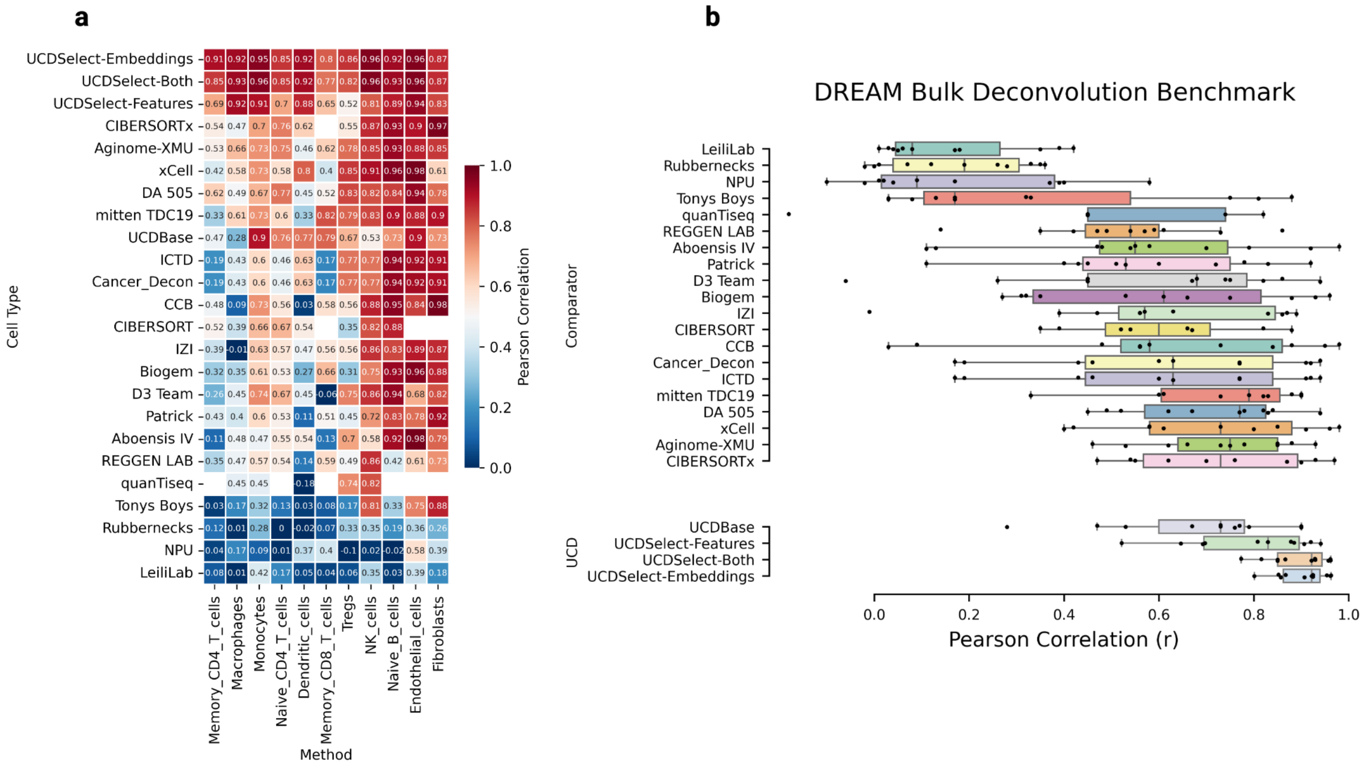

Supplementary Figure 9. Bulk RNA Deconvolution DREAM Challenge Benchmark. a) Heatmap comparing pearson correlations between ground truth and predicted cell type fractions of 11 cell types (x-axis) for various deconvolution algorithms (y-axis) using the DREAM Bulk Deconvolution challenge dataset consisting of 96 mixture samples. **b)** Boxplots of pearson correlations (x-axis) for cell types (n = 11) stratified by method (y-axis). For boxplots, the center line, box limits and box whiskers correspond to the median, first and third quartiles, and the 1.5x interquartile range, respectively. Individual data points are superimposed over each boxplot (Source Data File - Supp. Fig 2C).

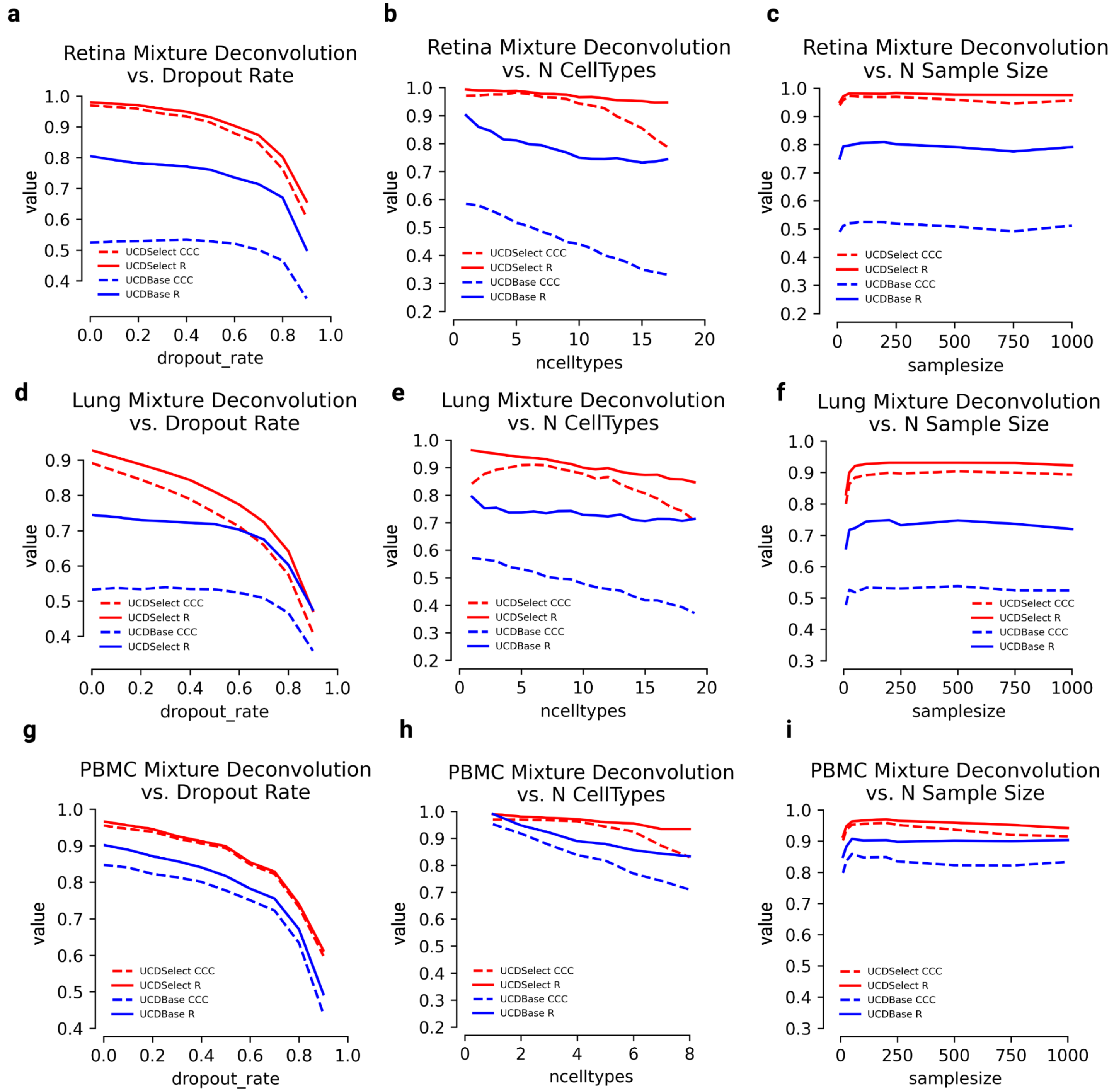

Supplementary Figure 10. Unicell Deconvolution Sensitivity Against Key Mixture Hyperparameters. Each line plot compares deconvolution performance with respect to concordance correlation coefficient (dashed lines, y-axis) and pearson's r (solid lines, y-axis) with respect to a variable hyperparameter using pseudo bulk mixture samples derived from single cell RNA sequencing dataset. **a/d/g** vary the gene dropout rate, where 0 indicates all genes expression values are used for prediction, while 1 indicates no gene expression values are passed through for prediction. **b/e/h** vary the number of cell types used in generating a pseudobulk mixtures. **c/f/i** vary the number of samples (i.e. cells) used to generate each pseudo bulk mixture. The first, second, and third rows correspond to retina, lung, and PBMC pseudo bulk mixture datasets, respectively. For each line plot, red lines denote the UCDSelect fine-tuned model was used, while blue lines denote UCDBase was used (Source Data File - Supp. Fig 2D-a/b/c/d/e/f/g/h/i).

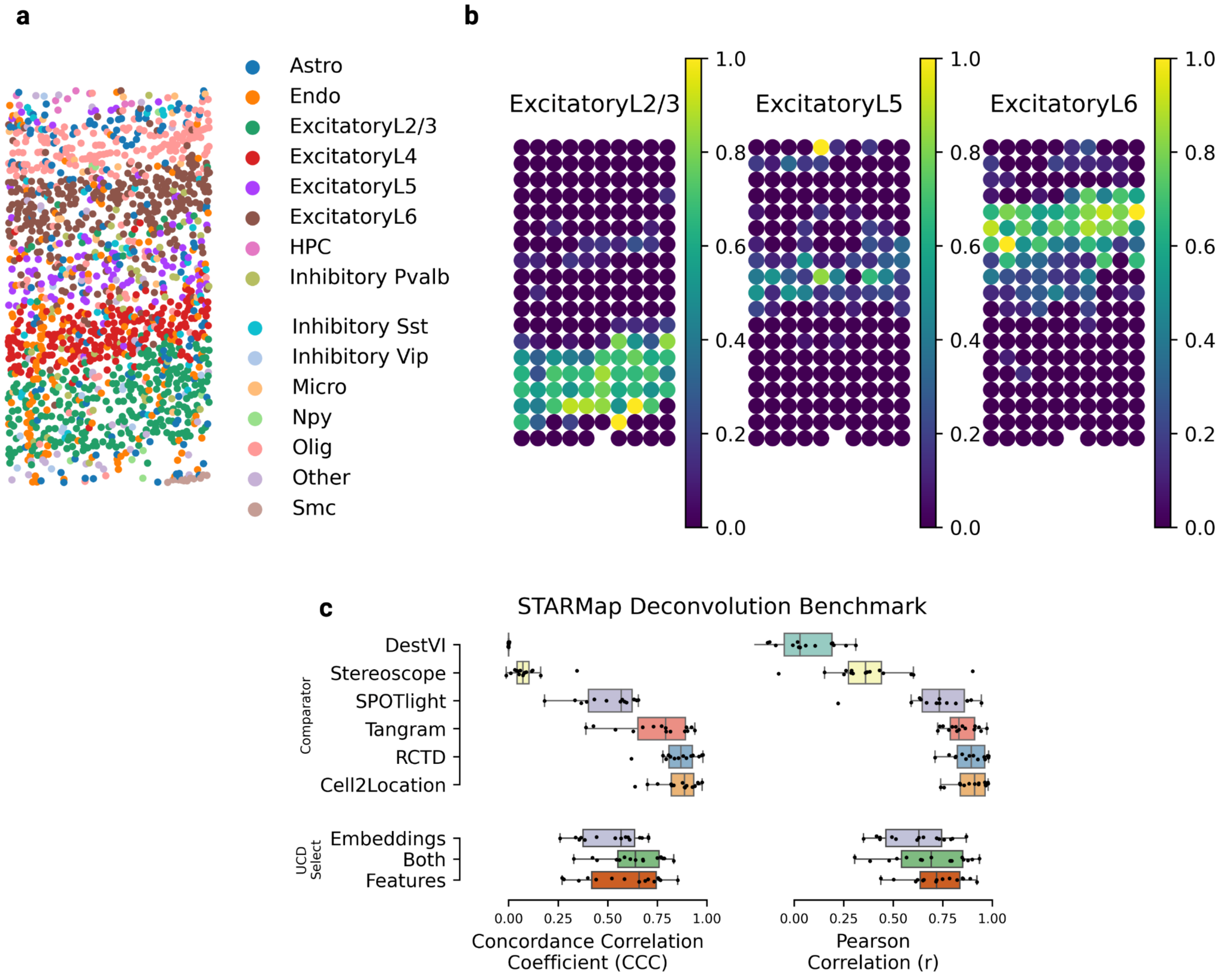

Supplementary Figure 11. Unicell Deconvolution Performance on in-situ STARMap Dataset. a) Spatial scatterplot of annotated cell types of mouse visual cortex. **b)** Representative distribution of cell type frequencies calculated using spatially downsampled STARMap data, aggregating cells within defined spatial proximity to form a ground-truth spatial mixture of cell types with known fractions. Colorbar scale denotes fraction of a given spatial spot corresponding to a given cell type (range 0 - 1 indicating no to all cells in a spot being of a given type). **c)** Box plots of concordance correlation coefficient (CCC, x-axis, left) and pearson correlation (x-axis, right) calculated for deconvolved cell types (n = 15 cell types) from downsampled spatial mixtures stratified by deconvolution method (y-axis). For boxplots, the center line, box limits and box whiskers correspond to the median, first and third quartiles, and the 1.5x interquartile range, respectively. Individual data points are superimposed over each boxplot (Source Data File - Supp. Fig 2E-c).

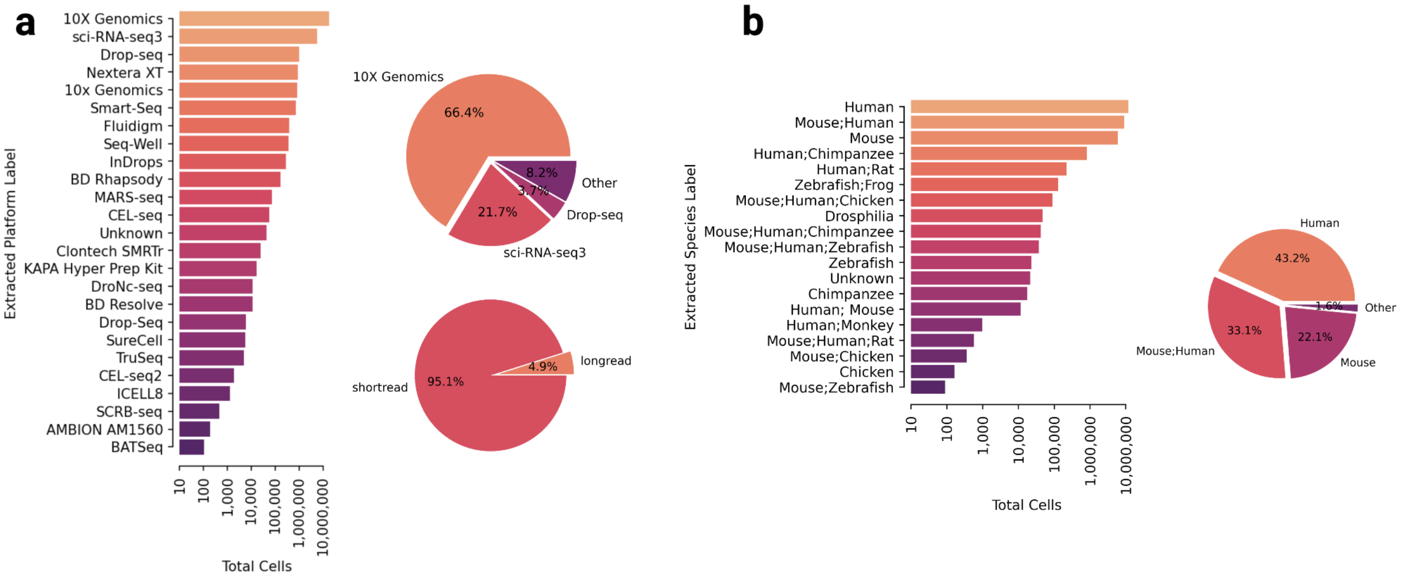

Supplementary Figure 12. Overview of Platform Technology & Species Metadata Among Training Data. a) (left) Barplot of single cell technology platform labels (y-axis) from automated text scanning of project accessions in relation to number of cells represented by log scale (x-axis) (Source Data File - Supp. Fig 2F-a-left). (right) Pie charts of top three technology platforms (top, Source Data File - Supp. Fig 2F-a-topright) and distribution of short vs. long read platforms (bottom, Source Data File - Supp. Fig 2F-a-bottomright). **b)** (left) Barplot of species labels (y-axis) from automated text scanning of project accessions in relation to the number of cells represented by log scale (x-axis) (Source Data File - Supp. Fig 2F-b-left). (right) Pie chart of top three species keyword pair(s) shown for linear comparison (Source Data File - Supp. Fig 2F-b-right).

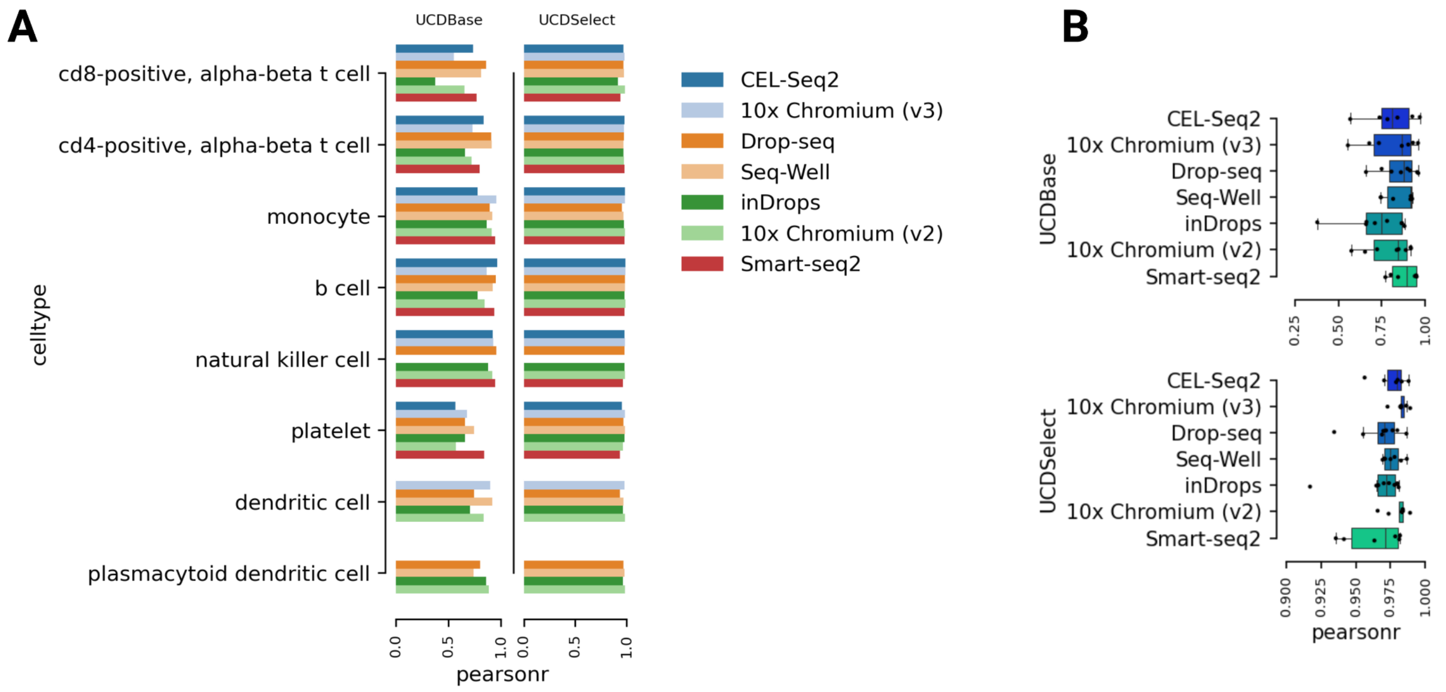

Supplementary Figure 13. Deconvolution Performance Differences by Technical Platform. a) Bar plots visualizing deconvolution accuracy, measured with pearson correlation (x-axis), of UniCell Deconvolve (UCD) Base and UCDSelect pre-trained models across different cell types (y-axis) derived from PBMC samples prepared using different single cell platform technologies. **b)** Alternative visualization showing box plots of cell type (n = 8) deconvolution accuracy measured by pearson correlation (x-axis), with respect to different single cell platform technologies (y-axis). For the boxplots, the center line, box limits and box whiskers correspond to the median, first and third quartiles, and the 1.5x interquartile range, respectively. Individual data points are superimposed over each boxplot (Supp. Fig 2G).

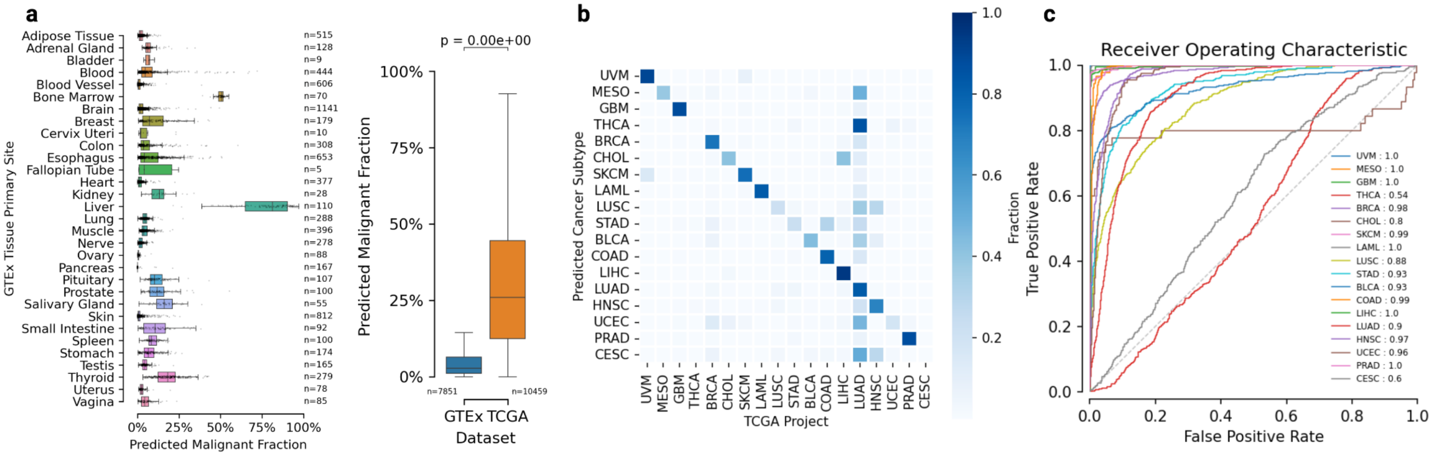

Supplementary Figure 14. UniCell differentiates between normal & malignant tissue and accurately classifies cancer subtypes. a) (left) Box plots of Unicell Deconvolve Base (UCDBase) predicted malignant cell fractions (x-axis) for presumed normal GTEx bulk RNA samples stratified by primary tissue site (y-axis). Sample sizes for each stratification are shown within the figure. Individual data points are superimposed over each boxplot Source Data File - Supp. Fig. 3A-a-left. (right) Box plot showing UCDBase predicted malignant cell fraction (y-axis) compared between all GTEx (n = 7,851) and all TCGA (n = 10459) samples (x-axis). P value indicates the unadjusted significance of difference between groups using an unpaired two-sided Wilcoxon rank sum test. For all boxplots, the center line, box limits and box whiskers correspond to the median, first and third quartiles, and the 1.5x interquartile range, respectively (Source Data File - Supp. Fig. 3A-a-right). **b)** Normalized prediction accuracy for cancer subtypes based on TCGA bulk RNA-seq sample deconvolution by UCDBase is shown as a heatmap. The x-axis represents samples classified into one of 18 TCGA projects, while the y-axis represents one of 18 potential cancer subtypes predicted by UCDBase. Prediction scores are normalized row-wise such that each row sums to 1 (Source Data File - Supp. Fig 3A-b). c) Receiver operating curves (ROC) for each cancer subtype present in both UCDBase and TCGA reflecting classification accuracy (Source Data File - Supp. Fig 3A-c).

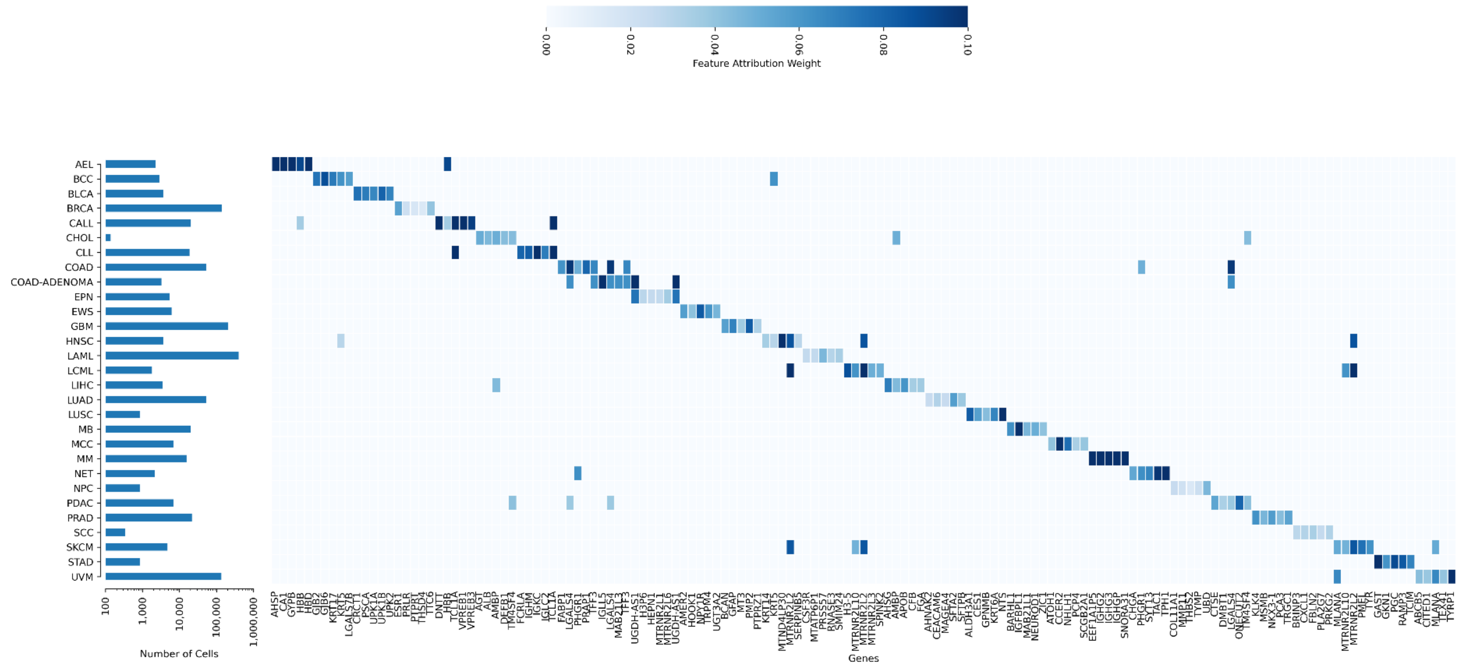

Supplementary Figure 15. Gene attribution analysis (GEA) with integrated gradients method identifies gene signatures unique to cancer subtypes. a) Heatmap of gene feature weights learned by UCDBase indicating positive associations between expression of a given gene (x-axis) and probability of predicting a corresponding cancer subtype (y-axis). Top 5 genes by positive feature attribution weight are shown (Source Data File - Supp. Fig 3B-right). Log of cell counts for each primary cancer type are shown on the left sub-axis (Source Data File - Supp. Fig 3B-left).

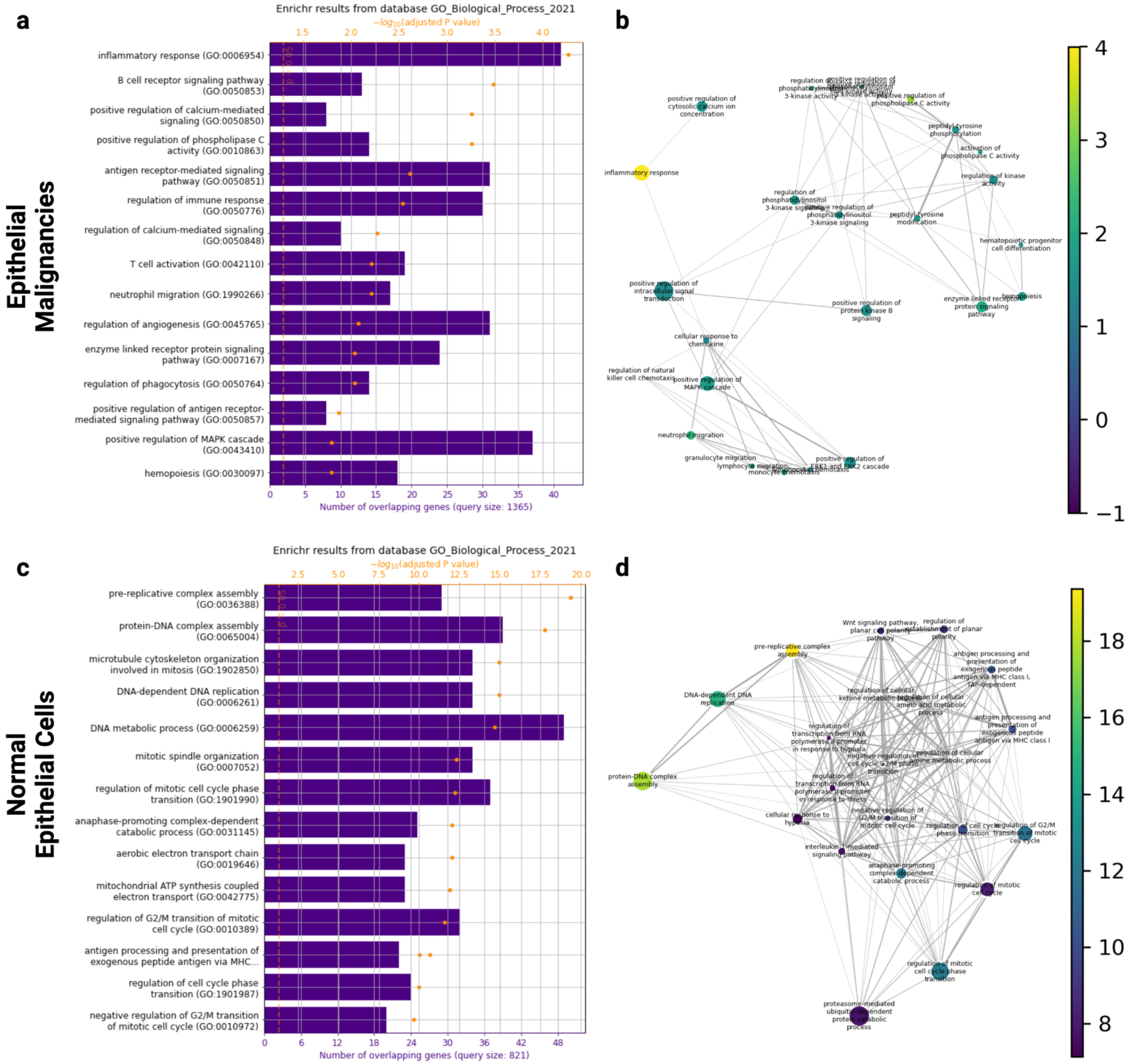

Supplementary Figure 16. Gene Set Enrichment Analysis (GSEA) of Grouped Feature Weights Using Enrichr Distinguishes Learned Attributes of Epithelial Malignancy. a) Bar plots of Enrichr results for top learned feature weights across pooled malignant epithelial cells (n = 1,365 positively attributed genes) (Source Data File - Supp. Fig 3C-a). **c)** Bar plots of Enrichr results for top learned feature weights across pooled normal epithelial cells (n = 821 positively attributed genes) (Source Data File - Supp. Fig 3C-c). For both **a/c**, the y-axis represents GO_BIOLOGICAL_PROCESS terms while the x-axis represents the number of overlapping genes within each term colored in purple and marked by the height of the horizontal barplot, while the yellow dots horizontal position represent the *-log10* adjusted p-value. Significance is calculated by Enrichr using a fisher's exact test, and subsequently corrected using the benjamini-hochberg method to account for multiple comparisons. **b/d)** Network plots visualizing related ontology terms for malignant and normal epithelial cell GSEA results, respectively. The colorbars of the network plots signify the strength of significance, as measured by the negative log of the adjusted p value calculated in a/c.

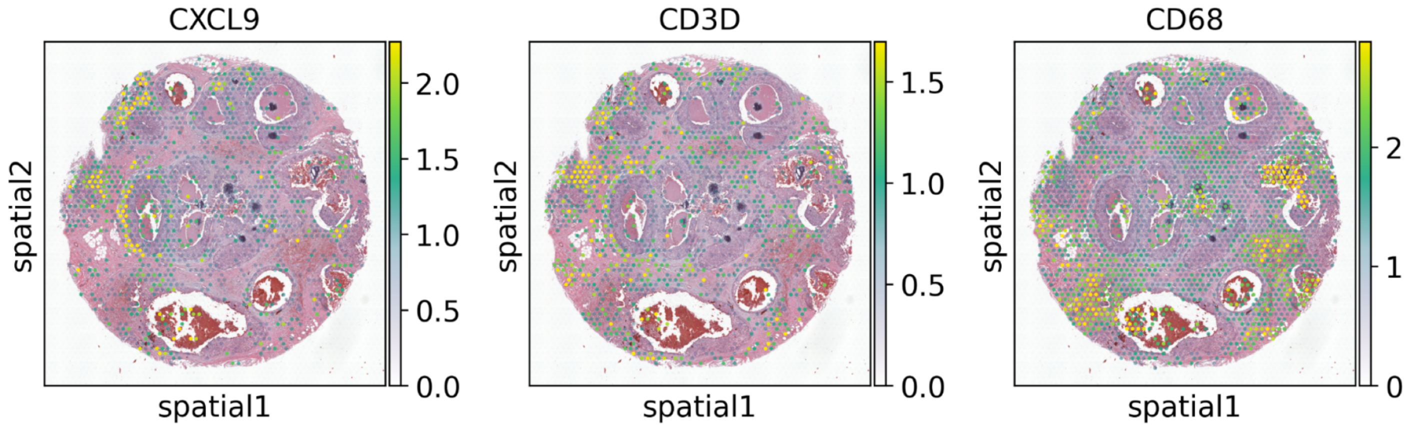

Supplementary Figure 17. Breast Adenocarcinoma Spatial Gene Expression Profiles of Key Immune Cell Markers. Spatial expression patterns of immune cell markers CD3D (t cell), CD68 (macrophage), and immune mediator chemokine CXCL9 within profiled spatial transcriptomic section of a breast adenocarcinoma (BRCA). Colorbars denote log normalized expression for each gene.

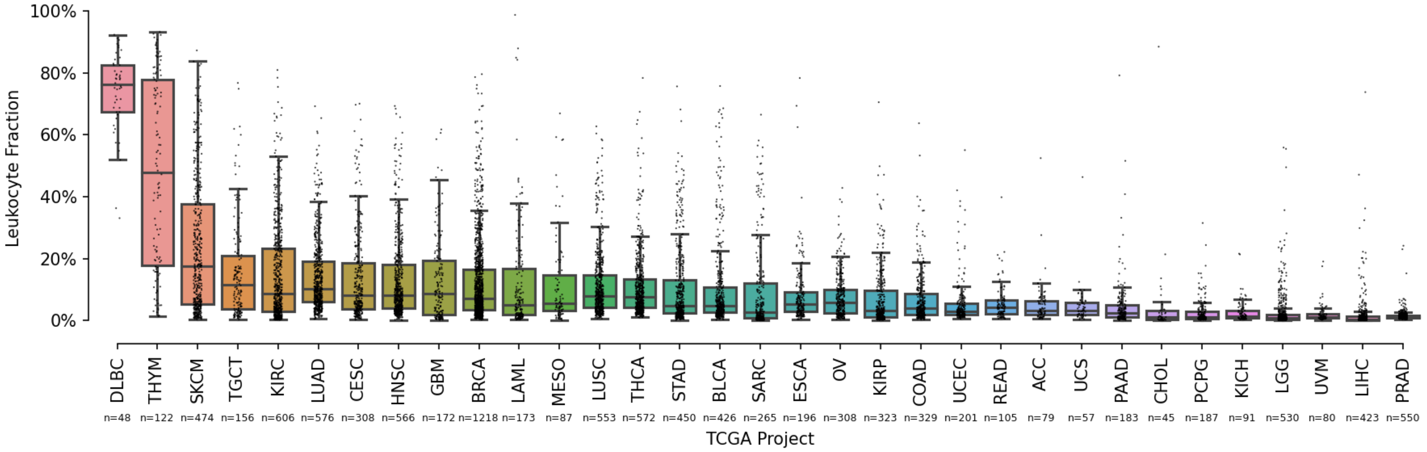

Supplementary Figure 18. Fraction of Immune Cells Predicted By UCDBase Across TCGA Subtypes. Box plots of normal immune cells by fraction of total sample (y-axis) deconvolved from bulk RNA-Seq samples by UniCell Deconvolve Base (UCDBase) derived from different TCGA cancer subtypes (x-axis). For all boxplots, the center line, box limits and box whiskers correspond to the median, first and third quartiles, and the 1.5x interquartile range, respectively. Individual data points are superimposed over each boxplot (Source Data File - Supp. Fig 3E).

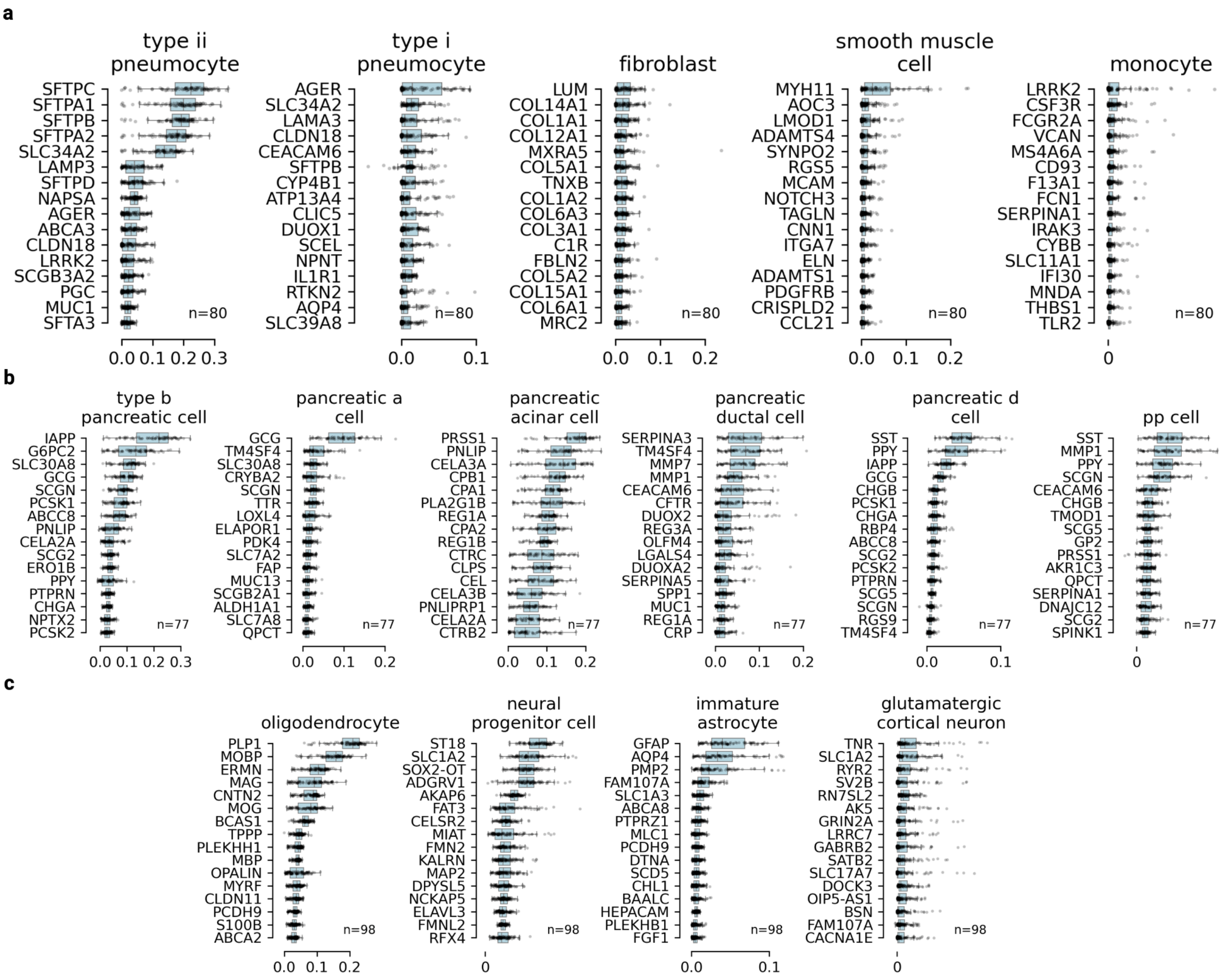

Supplementary Figure 19. Feature Attribution Weights for Key Cell Types Deconvolved By UniCell Base From Bulk RNA Profiled Tissues. a) Box plots of feature attribution weights (x-axis) for genes (y-axis) indicative of select cell types predicted to be present in lung tissue of patients with either idiopathic pulmonary fibrosis, acute lung injury , or healthy controls. Sample sizes shown on each figure represent individual patient samples (n = 80) (Source Data File - Supp. Fig 4A-a). **b)** Box plots of feature attribution weights (x-axis) for genes (y-axis) indicative of select cell types predicted to be present in pancreatic tissue of patients with diabetes, pre-diabetes, or healthy controls. Sample sizes shown on each figure represent individual patient samples (n = 77) (Source Data File - Supp. Fig 4A-b). **c)** Box plots of feature attribution weights (x-axis) for genes (y-axis) indicative of select cell types predicted to be present in brain tissue of patients with multiple sclerosis. Sample sizes shown on each figure represent individual patient samples (n = 98) (Source Data File - Supp. Fig 4A-c). For all boxplots in **a/b/c**, the center line, box limits and box whiskers correspond to the median, first and third quartiles, and the 1.5x interquartile range, respectively. Individual data points are superimposed over each boxplot.

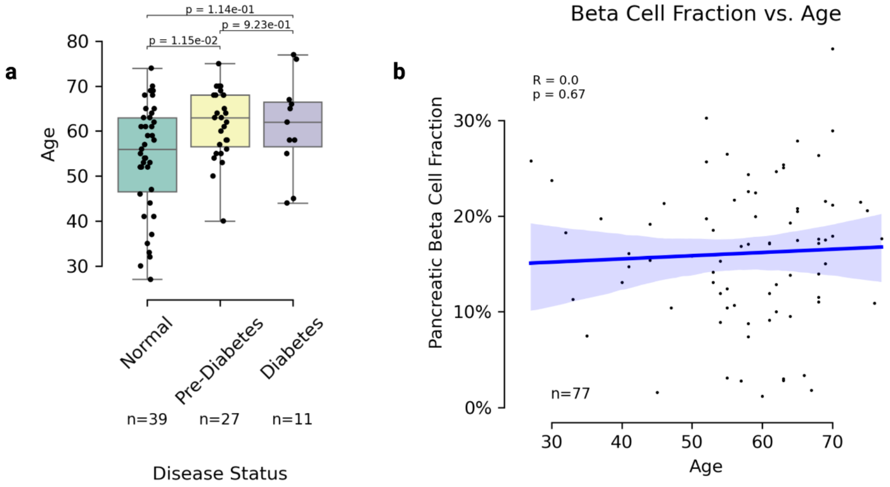

Supplementary Figure 20. Association Between Disease Status, Age, and Beta Cell Fraction in Type 2 Diabetes. a) Box plots of disease status (x-axis) with respect to age (y-axis) in patients with diabetes, pre-diabetes, or healthy controls. Sample sizes shown for each category of disease status represent individual patient samples. For boxplots, the center line, box limits and box whiskers correspond to the median, first and third quartiles, and the 1.5x interquartile range, respectively. Individual data points are superimposed over each boxplot. P values indicate the significance of difference between groups evaluated using an unpaired two-sided Wilcoxon rank sum test, with Benjamini-Hochberg correction for multiple comparisons. **b)** Scatterplot with superimposed linear regression line comparing the association between age (x-axis) and percentage of predicted beta cell fraction by UniCell Base (y-axis). The R value represents pearson correlation coefficient. The p value calculated for the significance level of the pearson correlation fit was computed using a two-tailed t-test. The confidence bands surrounding the regression line represent the standard deviation of the underlying observations (Source Data File - Supp. Fig 4B).

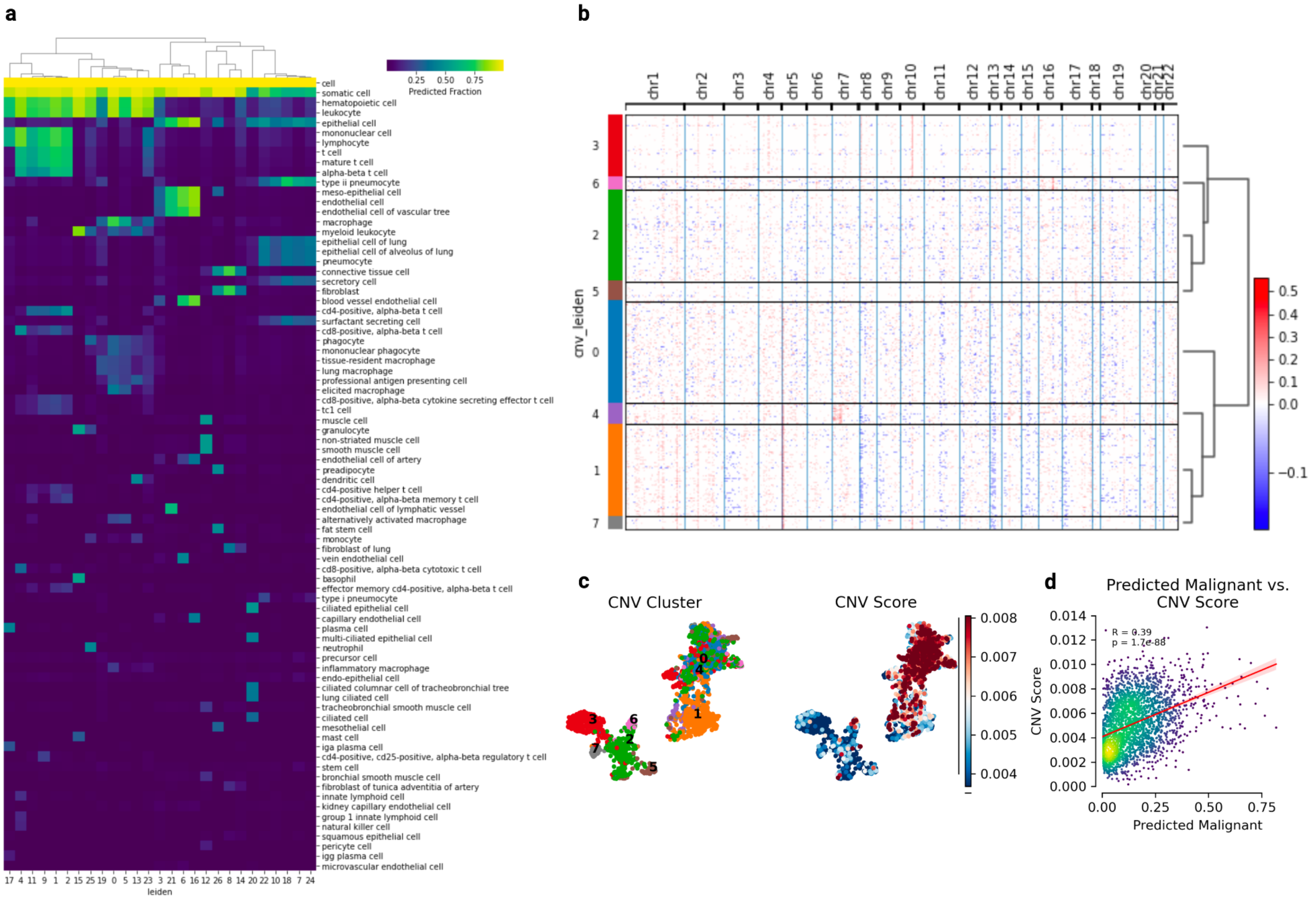

Supplementary Figure 21. UniCell Base (UCDBase) Cell Type Predictions For Non-small Cell Lung Cancer (NSCLC) Dataset & Orthogonal Validation of Malignant Cell States Using Copy Number Variation (CNV) Analysis. a) Heatmap of hierarchically ordered cell type predictions (y-axis) stratified by unsupervised leiden cluster (x-axis) for integrated NSCLC single cell dataset. Colorbar scale represents UCDBase prediction cell type fraction associated with a given leiden cluster. Results represent belief propagated cell type predictions, such that predictions for most the generic annotation “cell” shown on top sum to 1, with more specific annotations stratifying clusters into common cell subtypes. **b)** Heatmap of estimated chromosomal copy number variations (CNVs) across epithelial cell subsets, using several stromal cell types (smooth muscle, endothelial cells, lung ciliated cells) as ground-truth reference normal controls. **c)** UMAP visualization of epithelial cell subset and reclustred, colored by CNV-based leiden clusters (left) and absolute average CNV scores (right). **d)** Scatterplot showing UCDBase predicted malignant cell probability (x-axis) with respect to calculated average CNV score (y-axis). Each point represents an epithelial cell (n = 2509), while color of each point is reflective of point density (brighter indicates more tightly clustered points). The fit line represents the result of linear regression. The R value represents pearson correlation coefficient. The p value calculated for the significance level of the pearson correlation fit was computed using a two-tailed t-test. The confidence bands surrounding the regression line represent the standard deviation of the underlying observations (Source Data File - Supp. Fig 5A-d).

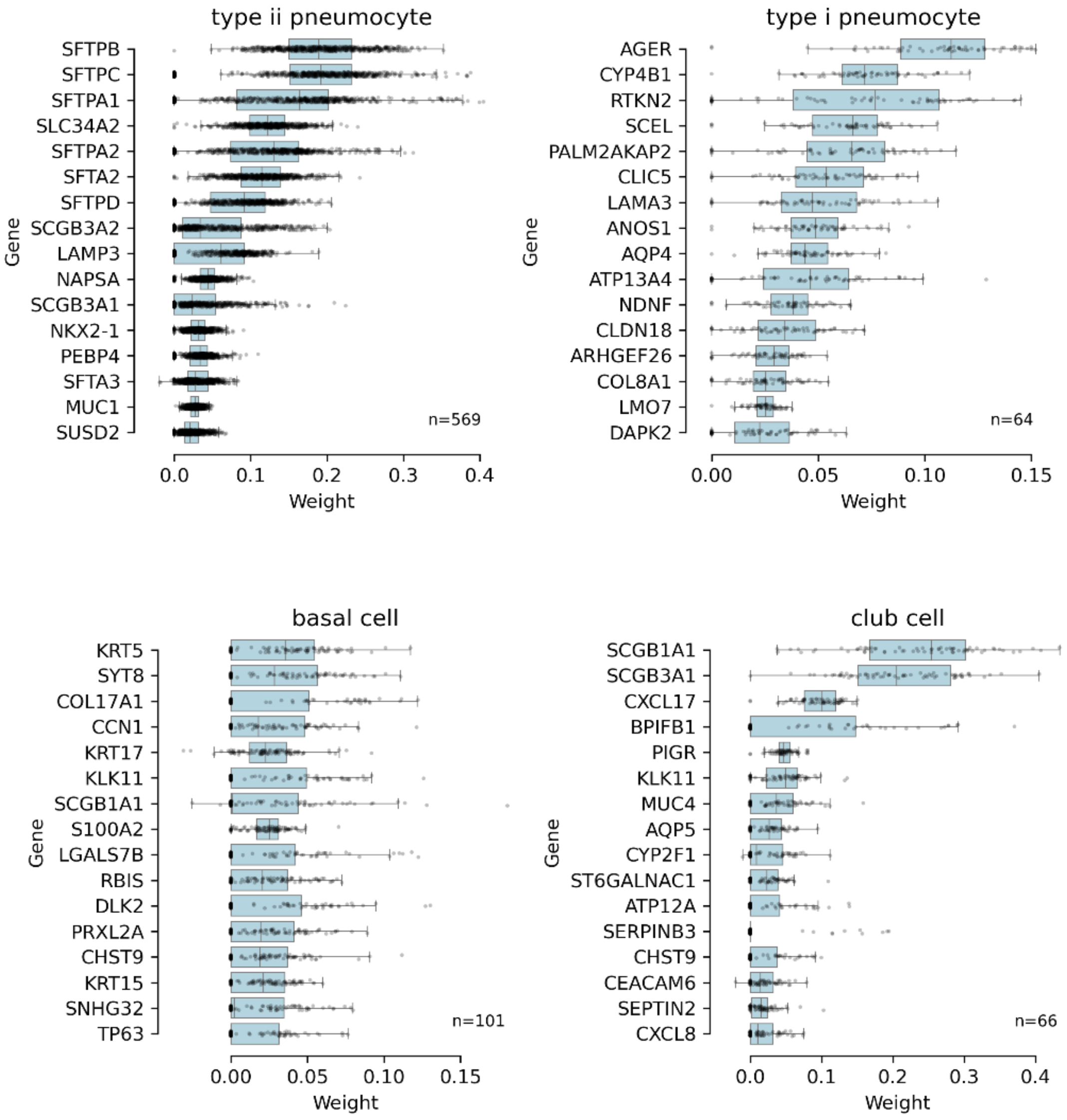

Supplementary Figure 22. Feature Attribution Weights For Key Epithelial Cell Subtypes Predicted By UniCell Deconvolve Base. Box plots of feature attribution weights (x-axis) for genes (y-axis) indicative of select non-cancerous cell types predicted to be present in lung tissue of patients with non-small cell lung cancer (NSCLC). Sample sizes shown on each figure represent individual single cells annotated as a given cell type (Source Data File - Supp. Fig 5B). For boxplots, the center line, box limits and box whiskers correspond to the median, first and third quartiles, and the 1.5x interquartile range, respectively. Individual data points are superimposed over each boxplot.

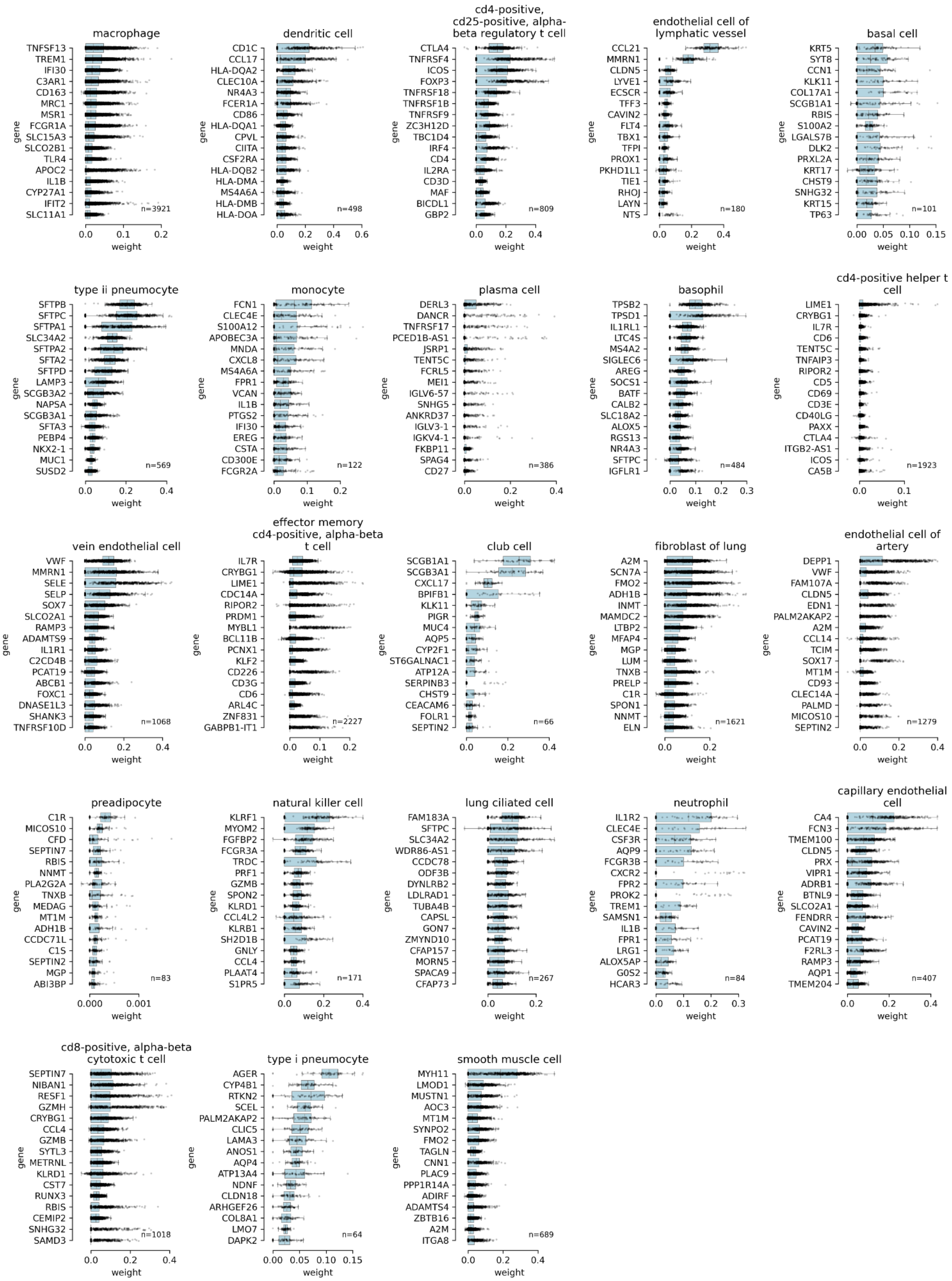

Supplementary Figure 23. Feature Attribution Weights For Key Epithelial Cell Subtypes Predicted By UniCell Deconvolve Base. Box plots of feature attribution weights (x-axis) for genes (y-axis) indicative of select non-cancerous cell types predicted to be present in lung tissue of patients with non-small cell lung cancer (NSCLC). Sample sizes shown on each figure represent individual single cells annotated as a given celltype. For boxplots, the center line, box limits and box whiskers correspond to the median, first and third quartiles, and the 1.5x interquartile range, respectively. Individual data points are superimposed over each boxplot (Source Data File - Supp. Fig 5C).

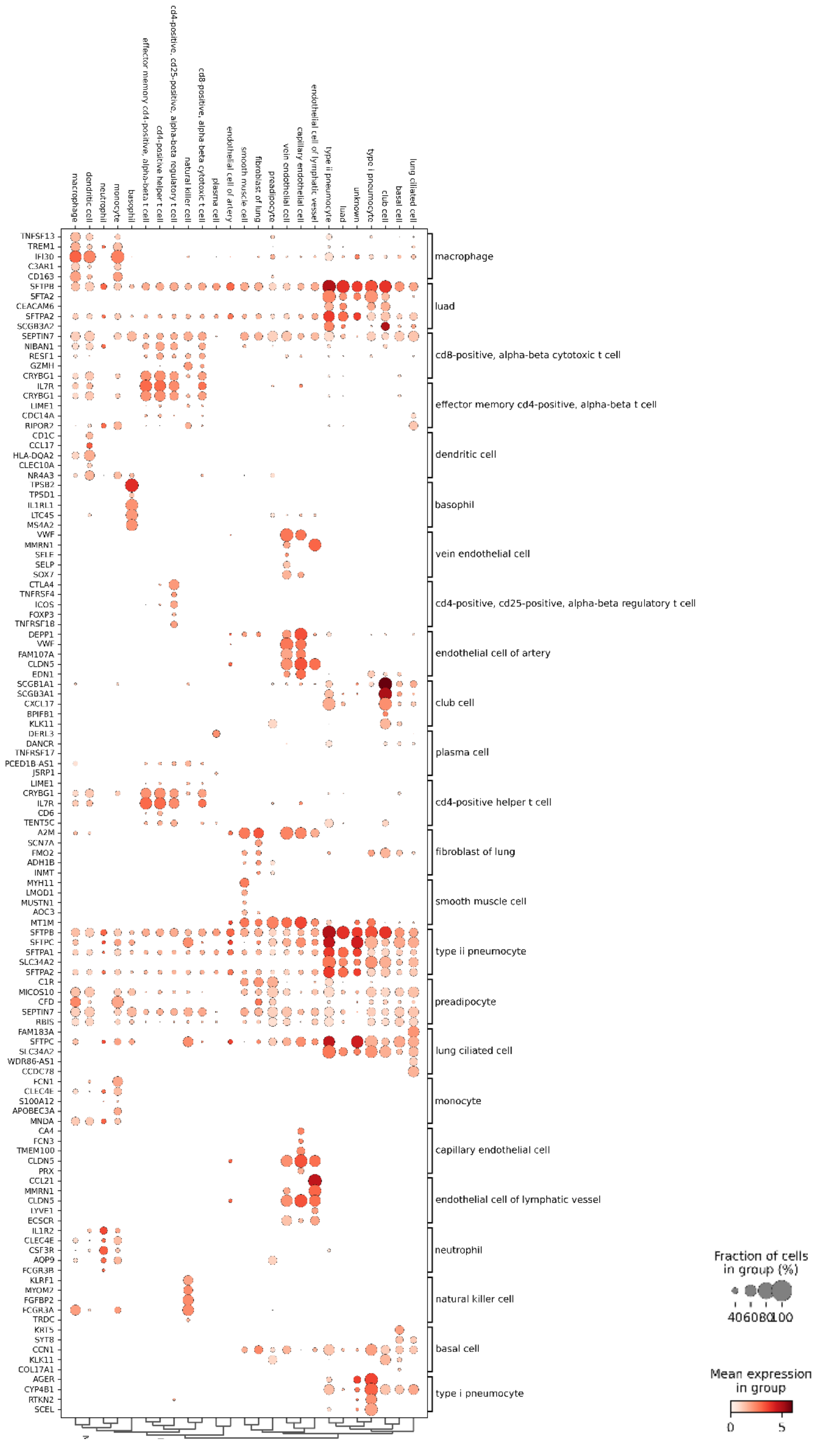

Supplementary Figure 24. Expression of UniCell Deconvolve Base Identified Marker Genes By Annotated Cell Cluster. Dotplot showing expression levels of marker genes for cell types identified by UniCell Deconvolve Base feature attributes (y-axis) stratified by clusters annotated to respective cell types (x-axis). Color of each spot represents the mean log-scaled expression of a given gene within a cluster. The size of each dot represents the fraction of cells in each cluster expressing the given gene.

Supplementary Table 1. Key Benchmarking Datasets & Sources

Dataset Description	Figure	Use	In UCD	Source	Accession	Link
10K PBMC Healthy Donor	2	Mixture	No	*10X Genomics*	n/a	source
5K PBMC Healthy Donor	2	Reference	No	*10X Genomics*	n/a	source
Wang et. al 2020 Lung	2	Mixture	No	cellxgene	GSE161382	source
Travaglini et. al 2020 Lung	2	Reference	Yes	cellxgene	PRJNA632939	source
Cowan et al. 2020 Retina Periphery	2	Mixture	No	cellxgene	EGAS00001004561	source
Cowan et al. 2020 Retina Fovea	2	Reference	No	cellxgene	EGAS00001004561	source
DREAM Bulk Deconvolution Challenge	S2	Reference & Mixture	No	GEO	GSE199324	source
Murine Kidney Injury	3	n/a	No	*rebuildingakidney (RBK)*	n/a	source
Human Breast Cancer	4	n/a	No	*10X Genomics*	n/a	source
Human Prostate Cancer	4	n/a	No	*10X Genomics*	n/a	source
Human Colon Cancer	4	n/a	No	*10X Genomics*	n/a	source
Idiopathic Pulmonary Fibrosis	5	n/a	No	GEO	GSE134692	source
Type II Diabetes	5	n/a	No	GEO	GSE50244	source
Multiple Sclerosis	5	n/a	No	GEO	GSE138614	source
STARMap	S2	n/a	No	Qu Kun Lab	n/a	source
Ding et. al. 2019 PBMC Technical Comparison	S2	n/a	No	GEO	GSE132044	source

Supplementary Table 2. Cell Type Deconvolution Gene Feature Attribute Detailed Interpretations. [illegible] sample-specific attributed marker genes, and review their biologic relevance to each study.

Fig	Tissue	Cell Type	Sample-Specific Attributed Marker(s)	Relevance	Ref
		Tubule Epithelial Cells	SLC34A1	The solute membrane transporter SLC34A1, a top predictor for PCT cells, is commonly overexpressed in the early cortical sections S1 & S2 of the PCT.	[1]
		Brush Cell	SLC6A18, SLC22A7	Both solute membrane transporters 6A18 and 22A7 are known markers for the S3 PCT, which adopts a brush-cell like phenotype.	[2,3]
		Distal Convoluted Tubule Epithelial Cell	SLC12A3	Thiazide-sensitive sodium chloride cotransporter (NCC) encoded by SLC12A3, is expressed selectively in the distal convoluted tubule along the apical epithelial membrane.	[4]
		Thick Ascending Limb of the Loop of Henle	SLC12A1	Solute carrier channel family 12 member 1, a canonical marker for TAL/LOH epithelial cells.	[5]
		Collecting Duct	AQP2	Water-reabsorption aquaporin channel 2, a canonical marker for CD epithelial cells.	[6]
		Intercalated Cell	ATP6V1G3	An ATPase identified as a top differentially expressed IC cell gene compared with other kidney epithelial cells.	[7]
		Kidney Fibroblast Cells	CFH	Complement Factor H plays a critical role in modulating the severity of innate immune activation following acute injury.	[8]
			MS4A7	This membrane-bound complex protein is a known suppressor macrophage marker gene.	[9]
			CCL8	A chemokine thought to promote the recruitment and polarization of M2-macrophages, supporting the establishment of auto/paracrine-like sustainment of chronic macrophage infiltration in late stage IRI and the establishment of chronic inflammation coinciding with fibrosis.	[10]
		Suppressor Macrophages	TREM2	Believed to regulate macrophage polarization in chronic kidney disease.	[11]
3	Kidney	T Cells	CCR7	CCR7+ T cells play a role in mediating kidney injury during transplant allograft rejection.	[12]
			NKX3-1	An androgen-regulated homeodomain gene localizing to prostate epithelium, which has shown to be positively expressed in the majority of primary prostate cancers.	[13]
			PCA3	Prostate cancer antigen 3 is a segment of noncoding mRNA overexpressed in 95% or more prostate cancers.	[14]
	Prostate Single Cell Database	Prostate Cancer Single Cells (PRAD)	FOLH1	FOLH1 encodes prostate specific membrane antigen (PSMA), a transmembrane protein with known carboxypeptidase activity that is commonly expressed in prostatic tissue, and overexpressed in prostate cancers.	[15]
			MLANA	Codes for the melan-A protein are believed to play a functional role in intracellular melanosome biogenesis, exclusively expressed in melanocytes, melanoma and retinal pigment epithelium.	[16]
			TRYP1	A tyrosinase-related protein found to correlate with metastatic melanoma clinical outcomes.	[17]
	Skin Cancer Single Cell Database	Melanoma Single Cells (SKCM)	MTRNR2L2	A cancer-associated mitochondrial related gene which codes for the anti-apoptotic peptide humanin.	[18]
			SCGB2A2, SCGB1D2	Secretoglobulins forming a protein complex commonly overexpressed in breast cancers.	[19,20]
			PRLR	Codes for prolactin receptor, which is overexpressed in a significant fraction of breast cancers and comprises one of the three major hormone receptors used for BC subtyping (ER, PR, and HER2).	[21]
			ELAPOR1	Endosome-lysosome autophagy regulator 1 has been known to be overexpressed in several subtypes of cancer including breast, endometrial, and prostate cancers.	[22]
		Breast Adenocarcinoma (BRCA)	AZGP1	An androgen-response secreted glycoprotein which has been associated with several cancers including breast, prostate, and hepatocellular carcinomas.	[23]
			LUM, COL1A1, COL1A2, COl3A1, FBLN2	Well-established canonical fibroblast markers representing various extracellular-matrix (ECM) genes	[24]
			DPT	A secreted extracellular matrix adhesion protein recently identified as a possible pan-tissue fibroblast marker.	[25]
			C1R	Complement CR1 is a component of the classical innate immune response pathway mediating local immuno-inflammatory responses.	[26]
		Fibroblast	COL12A1	Implicated in pro-inflammatory stromal desmoplasia and tumor progression	[27]
			CDH5	Endothelial-cell specific transmembrane cadherin located along intercellular junctions.	[28]
		Endothelial Cell	APLN	Supports pro-angiogenic states among endothelial cells, inducing migration and proliferation.	[29]
			MSR1	Known to be overexpressed in breast cancer TAMs and has been associated with poor clinical outcomes.	[30]
		Suppressor Macrophage	CCL8	C-C motif chemokine ligand 8 has been shown to be secreted by TAMs to promote active tumorigenesis.	[31]
		IgG Plasma Cell	IGHG1,IGHG2,IGHG3, IGHG4	immunoglobulin heavy chains 1-4 are commonly overexpressed among IgG plasma cells.	[32]
	Breast Adenocarcinoma Spatial Section	T Cell	TRAC	The t-cell receptor alpha constant gene is a ubiquitous component of MHC complexes on all alpha-beta t cell subtypes.	[33]
			KLK4	An intracellular kallikrein localizing to the nucleus and is believed to exert a pro-proliferative effect on prostate cancer cells via cell cycle signaling interactions.	[34]
		Prostate Adenocarcinoma (PRAD)	OR51E2	Ectopic olfactory G-protein coupled receptor is highly overexpressed in prostate cancers and may play a role in later-stage progression associated with neuroendocrine-like transdifferentiation	[35,36]
		Prostate Luminal Epithelial Cell	KLK2, KLK3	KLK3 encodes Prostate Serum Antigen (PSA), a secreted, chymotryptic-like enzyme involved in sperm cell maturation, which is cleaved from its zymogenic to active form by related secreted peptidase encoded by KLK2. Both genes are ubiquitously expressed in prostate luminal epithelial cells, both normal and cancerous.	[37]
		Prostatic Basal Cells	TP63, KRT5	Canonical basal cell marker genes. TP63 regulates epithelial differentiation processes, while cytokeratin 5 forms intermediate filaments of the basal cell cytoskeleton.	[38,39]
		Schwann Cells	MPZ, CDH19, SOX10	Established schwann cell marker genes. Myelin-protein Z forms part of the myelin sheath that insulates nerve fibers. Cadherin 19 secures tight junctions between schwann cells, while SOX10 is a critical transcription factor essential to schwann cell identity.	[40]
		Myofibroblasts	CNN1, LMOD1	Contractility promoting gene calponin-1 is known to be significantly upregulated in fibroblast populations that are treated with TGF-beta to induce myofibroblast-like differentiation, however it also plays a role in driving smooth muscle predictions	[41]
	Prostate Adenocarcinoma Spatial Section	Smooth Muscle Cells	LMOD1, CNN1	Leiomodin 1 is shown in recent studies to be expressed in only mature smooth muscle cells, although it does play a role, albeit smaller in myofibroblast predictions as well.	[42]
		Colorectal Adenocarcinoma	LGALS4, CEACAM6	Known COAD diagnostic marker genes.	[43]
		Fibroblast	COL1A1, COL1A2, LUM	Well-established canonical fibroblast markers representing various extracellular-matrix (ECM) genes.	[24]
			IGHG1-4	immunoglobulin heavy chains 1-4 are commonly overexpressed among plasma cells.	[44]
		Plasmablasts	MZB1	Supports a positive feedback loop with BLIMP1 to induce terminal differentiation of plasma cell phenotype.	[44,45]
		Macrophages	CCL8, CXCL10, CXCL9	Involved in the attraction of t cells into the tumor microenvironment via interaction with t-cell bound CXCR3	[46]
4	Colorectal Adenocarcinoma Spatial Section	T Cell	CXCL10	Secreted by t cells infiltrating tumor micro movements as part of positive feedback loops maintaining tumor immune responses	[47]
		Type II Pneumocytes (ATII)	SFTPA1, SFTPC, SFTPB	Encode surfactant proteins that function to coat the alveolar epithelium, supporting effective gas exchange. Canonical ATII cell markers.	[48]

		Type I Pneumocytes (ATII)	AGER	Encodes advanced glycosylation end-product specific receptor, overexpressed in mature, differentiated ATI cells forming the majority of alveolar surface area.	[49,50]
			COL1A1, LUM	Well-established canonical fibroblast markers	[24]
		Fibroblast	MXRA5, COL14A1,	Matrix remodeling gene and collagen associated with lung fibrosis.	[51,52]
		Smooth Muscle Cell	MYH11	Myosin heavy chain 11 is a core component of smooth muscle cell contractile apparatus.	[53]
	IPF, ALI Lung Bulk RNA Sample	Monocyte	LRRK2	Expressed in various myeloid cell populations and is associated with inflammatory disease processes.	[54]
			IAPP	Co-secreted with insulin and thought to be responsible for the accumulation of cytotoxic amyloid deposits characteristic of type 2 diabetes pathohistology, exacerbating cellular stress and eventually leading to beta cell death	[55]
		Beta Cell	G6PC2	Selectively overexpressed in pancreatic beta cells, serving to maintain high rates of intracellular glucose uptake	[56]
		Alpha Cell	GCG	Glucagon is selectively secreted by pancreatic alpha cells to counteract effects of insulin from beta cells.	[57]
		Delta Cell	SST	Somatostatin is secreted by pancreatic delta cells that is involved in the regulation of alpha nd beta cell activity.	[58]
	Type 2 Diabetes Pancreatic Bulk RNA Sample	PP Cell	PPY, SST	It has been shown in mouse and rat studies that upwards of 60% of PP cells co-express SST in addition to canonical pancreatic polypeptide (PPY).	[59,60]
			PLP1	Proteolipid protein 1 encodes a transmembrane protein that forms the primary component of myelin, insulating neurons and improving action potential transduction.	[61]
		Oligodendrocytes	MOBP	Myelin associated oligodendrocyte basic protein is overexpressed in oligodendrocytes and forms an integral component of the myelin sheath.	[61]
			GFAP, AQP4	traditional lineage-committed astrocyte markers	[62]
5	Multiple Sclerosis Bulk RNA Samples	Immature Astrocytes	FAM107A	Actin-binding protein that has previously been reported to be overexpressed in astrocyte progenitor populations.	[63]
		CD4+ Regulatory T Cell	FOXP3, CTLA4	Characteristic markers of CD4 regulatory t cells	[64]
		CD4+ Effector Memory T Cell	IL7R	Required for the maintenance of memory t cell phenotypes	[65]
			GZMH, GZMB	Granzymes functioning to enable cytotoxic behavior of t cells	[66]
		CD8+ Cytotoxic T Cell	SEPTIN7	Play a role in the related cytotoxic functions of immune cells.	[67].
		Natural Killer Cell	KLRF1	Well-known NK cell marker.	[68]
		Type I Pneumocyte	AGER	Encodes advanced glycosylation end-product specific receptor, overexpressed in mature, differentiated ATI cells forming the majority of alveolar surface area.	[49,50]
		Type II Pneumocyte	SFTPC	Encode surfactant protein that functions to coat the alveolar epithelium.	[48]
		Basal Cells	KRT5	cytokeratin 5 forms intermediate filaments of the basal cell cytoskeleton.	[69]
6	Non-Small Cell Lung Cancer Biopsy	Club Cells	SCGB1A1, SCGB3A2	Secretoglobulin proteins secreted by lung airway epithelial cells, specific for club cell phenotypes.	[70]

ID	Reference
1	Kusaba T, Lalli M, Kramann R, Kobayashi A, Humphreys BD. Differentiated kidney epithelial cells repair injured proximal tubule. Proc Natl Acad Sci U S A. 2014;111: 1527–1532.
2	Ransick A, Lindström NO, Liu J, Zhu Q, Guo J-J, Alvarado GF, et al. Single-Cell Profiling Reveals Sex, Lineage, and Regional Diversity in the Mouse Kidney. Dev Cell. 2019;51: 399–413.e7.
3	Singer D, Camargo SMR, Huggel K, Romeo E, Danilczyk U, Kuba K, et al. Orphan transporter SLC6A18 is renal neutral amino acid transporter B0AT3. J Biol Chem. 2009;284: 19953–19960.
4	Moes AD, van der Lubbe N, Zietse R, Loffing J, Hoorn EJ. The sodium chloride cotransporter SLC12A3: new roles in sodium, potassium, and blood pressure regulation. Pflugers Arch. 2014;466: 107–118.
5	Musso CG, Macías-Núñez JF. Dysfunction of the thick loop of Henle and senescence: from molecular biology to clinical geriatrics. Int Urol Nephrol. 2011;43: 249–252.
6	Kwon T-H, Frøkiær J, Nielsen S. Regulation of aquaporin-2 in the kidney: A molecular mechanism of body-water homeostasis. Kidney Res Clin Pract. 2013;32: 96–102.
7	Saxena V, Fitch J, Ketz J, White P, Wetzel A, Chanley MA, et al. Whole Transcriptome Analysis of Renal Intercalated Cells Predicts Lipopolysaccharide Mediated Inhibition of Retinoid X Receptor alpha Function. Sci Rep. 2019;9: 545.
8	Valoti E, Noris M, Perna A, Rurali E, Gherardi G, Breno M, et al. Impact of a Complement Factor H Gene Variant on Renal Dysfunction, Cardiovascular Events, and Response to ACE Inhibitor Therapy in Type 2 Diabetes. Front Genet. 2019;10: 681.
9	Arlauckas SP, Garren SB, Garris CS, Kohler RH, Oh J, Pittet MJ, et al. Arg1 expression defines immunosuppressive subsets of tumor-associated macrophages. Theranostics. 2018;8: 5842–5854.
10	Sierra-Filardi E, Nieto C, Domínguez-Soto A, Barroso R, Sánchez-Mateos P, Puig-Kroger A, et al. CCL2 shapes macrophage polarization by GM-CSF and M-CSF: identification of CCL2/CCR2-dependent gene expression profile. J Immunol. 2014;192:
11	Cao Y, Qiancheng X, Cong F, Yuwei W. FP340 TREM-2 regulates macrophage polarization in chronic renal fibrosis. Nephrol Dial Transplant. 2019;34: gfz106–FP340.
12	Kim KW, Kim B-M, Doh KC, Cho M-L, Yang CW, Chung BH. Clinical significance of CCR7+CD8+ T cells in kidney transplant recipients with allograft rejection. Sci Rep. 2018;8: 8827.
13	Gurel B, Ali TZ, Montgomery EA, Begum S, Hicks J, Goggins M, et al. NKX3.1 as a marker of prostatic origin in metastatic tumors. Am J Surg Pathol. 2010;34: 1097–1105.
14	Marks LS, Bostwick DG. Prostate Cancer Specificity of PCA3 Gene Testing: Examples from Clinical Practice. Rev Urol. 2008;10: 175–181.
15	Chang SS. Overview of prostate-specific membrane antigen. Rev Urol. 2004;6 Suppl 10: S13–8.
16	Du J, Miller AJ, Widlund HR, Horstmann MA, Ramaswamy S, Fisher DE. MLANA/MART1 and SILV/PMEL17/GP100 are transcriptionally regulated by MITF in melanocytes and melanoma. Am J Pathol. 2003;163: 333–343.
17	Journe F, Id Boufker H, Van Kempen L, Galibert M-D, Wiedig M, Salès F, et al. TYRP1 mRNA expression in melanoma metastases correlates with clinical outcome. Br J Cancer. 2011;105: 1726–1732.
18	Bodzioch M, Lapicka-Bodzioch K, Zapala B, Kamysz W, Kiec-Wilk B, Dembinska-Kiec A. Evidence for potential functionality of nuclearly-encoded humanin isoforms. Genomics. 2009;94: 247–256.
19	Zafrakas M, Petschke B, Donner A, Fritzsche F, Kristiansen G, Knüchel R, et al. Expression analysis of mammaglobin A (SCGB2A2) and lipophilin B (SCGB1D2) in more than 300 human tumors and matching normal tissues reveals their co-expression
20	Talaat IM, Hachim MY, Hachim IY, Ibrahim RAE-R, Ahmed MAER, Tayel HY. Bone marrow mammaglobin-1 (SCGB2A2) immunohistochemistry expression as a breast cancer specific marker for early detection of bone marrow micrometastases. Sci Rep.
21	Sleightholm R, Neilsen BK, Elkhatib S, Flores L, Dukkipati S, Zhao R, et al. Percentage of Hormone Receptor Positivity in Breast Cancer Provides Prognostic Value: A Single-Institute Study. J Clin Med Res. 2021;13: 9–19.
22	Pontén F, Jirström K, Uhlen M. The Human Protein Atlas--a tool for pathology. J Pathol. 2008;216: 387–393.
23	Tian H, Ge C, Zhao F, Zhu M, Zhang L, Huo Q, et al. Downregulation of AZGP1 by Ikaros and histone deacetylase promotes tumor progression through the PTEN/Akt and CD44s pathways in hepatocellular carcinoma. Carcinogenesis. 2017;38: 207–
24	Muhl L, Genové G, Leptidis S, Liu J, He L, Mocci G, et al. Single-cell analysis uncovers fibroblast heterogeneity and criteria for fibroblast and mural cell identification and discrimination. Nat Commun. 2020;11: 3953.
25	Zeltz C, Navab R, Heljasvaara R, Kusche-Gullberg M, Lu N, Tsao M-S, et al. Integrin α11β1 in tumor fibrosis: more than just another cancer-associated fibroblast biomarker? J Cell Commun Signal. 2022. doi:10.1007/s12079-022-00673-3
26	Afshar-Kharghan V. The role of the complement system in cancer. J Clin Invest. 2017;127: 780–789.
27	Jiang X, Wu M, Xu X, Zhang L, Huang Y, Xu Z, et al. COL12A1, a novel potential prognostic factor and therapeutic target in gastric cancer. Mol Med Rep. 2019;20: 3103–3112.
28	Breviario F, Caveda L, Corada M, Martin-Padura I, Navarro P, Golay J, et al. Functional properties of human vascular endothelial cadherin (7B4/cadherin-5), an endothelium-specific cadherin. Arterioscler Thromb Vasc Biol. 1995;15: 1229–1239.
29	Helker CS, Eberlein J, Wilhelm K, Sugino T, Malchow J, Schuermann A, et al. Apelin signaling drives vascular endothelial cells toward a pro-angiogenic state. Elife. 2020;9. doi:10.7554/eLife.55589
30	He Y, Zhou S, Deng F, Zhao S, Chen W, Wang D, et al. Clinical and transcriptional signatures of human CD204 reveal an applicable marker for the protumor phenotype of tumor-associated macrophages in breast cancer. Aging . 2019;11: 10883–109
31	Zhang X, Chen L, Dang W-Q, Cao M-F, Xiao J-F, Lv S-Q, et al. CCL8 secreted by tumor-associated macrophages promotes invasion and stemness of glioblastoma cells via ERK1/2 signaling. Lab Invest. 2020;100: 619–629.
32	Chen J, Tan Y, Sun F, Hou L, Zhang C, Ge T, et al. Single-cell transcriptome and antigen-immunoglobin analysis reveals the diversity of B cells in non-small cell lung cancer. Genome Biol. 2020;21: 152.
33	TRAC T cell receptor alpha constant [Homo sapiens (human)] - Gene - NCBI. [cited 21 Feb 2022]. Available: https://www.ncbi.nlm.nih.gov/gene/28755
34	Klokk TI, Kilander A, Xi Z, Waehre H, Risberg B, Danielsen HE, et al. Kallikrein 4 is a proliferative factor that is overexpressed in prostate cancer. Cancer Res. 2007;67: 5221–5230.

35 Pronin A, Slepak V. Ectopically expressed olfactory receptors OR51E1 and OR51E2 suppress proliferation and promote cell death in a prostate cancer cell line. J Biol Chem. 2021;296: 100475.

36 Abaffy T, Bain JR, Muehlbauer MJ, Spasojevic I, Lodha S, Bruguera E, et al. A Testosterone Metabolite 19-Hydroxyandrostenedione Induces Neuroendocrine Trans-Differentiation of Prostate Cancer Cells via an Ectopic Olfactory Receptor. Front Onc[illegible]

37 Adhyam M, Gupta AK. A Review on the Clinical Utility of PSA in Cancer Prostate. Indian J Surg Oncol. 2012;3: 120–129.

38 Kurita T, Medina RT, Mills AA, Cunha GR. Role of p63 and basal cells in the prostate. Development. 2004;131: 4955–4964.

39 Pignon J-C, Grisanzio C, Geng Y, Song J, Shivdasani RA, Signoretti S. p63-expressing cells are the stem cells of developing prostate, bladder, and colorectal epithelia. Proc Natl Acad Sci U S A. 2013;110: 8105–8110.

40 Stratton JA, Kumar R, Sinha S, Shah P, Stykel M, Shapira Y, et al. Purification and Characterization of Schwann Cells from Adult Human Skin and Nerve. eNeuro. 2017;4. doi:10.1523/ENEURO.0307-16.2017

41 Scharenberg MA, Pippenger BE, Sack R, Zingg D, Ferralli J, Schenk S, et al. TGF-β-induced differentiation into myofibroblasts involves specific regulation of two MKL1 isoforms. J Cell Sci. 2014;127: 1079–1091.

42 Nanda V, Miano JM. Leiomodin 1, a New Serum Response Factor-dependent Target Gene Expressed Preferentially in Differentiated Smooth Muscle Cells*. J Biol Chem. 2012;287: 2459–2467.

43 Ferlizza E, Solmi R, Miglio R, Nardi E, Mattei G, Sgarzi M, et al. Colorectal cancer screening: Assessment of CEACAM6, LGALS4, TSPAN8 and COL1A2 as blood markers in faecal immunochemical test negative subjects. J Advert Res. 2020;24: 99–10[illegible]

44 Andreani V, Ramamoorthy S, Pandey A, Lupar E, Nutt SL, Lämmermann T, et al. Cochaperone Mzb1 is a key effector of Blimp1 in plasma cell differentiation and β1-integrin function. Proc Natl Acad Sci U S A. 2018;115: E9630–E9639.

45 Shaffer AL, Lin KI, Kuo TC, Yu X, Hurt EM, Rosenwald A, et al. Blimp-1 orchestrates plasma cell differentiation by extinguishing the mature B cell gene expression program. Immunity. 2002;17: 51–62.

46 Tokunaga R, Zhang W, Naseem M, Puccini A, Berger MD, Soni S, et al. CXCL9, CXCL10, CXCL11/CXCR3 axis for immune activation - A target for novel cancer therapy. Cancer Treat Rev. 2018;63: 40–47.

47 Peperzak V, Veraar EAM, Xiao Y, Babala N, Thiadens K, Brugmans M, et al. CD8+ T cells produce the chemokine CXCL10 in response to CD27/CD70 costimulation to promote generation of the CD8+ effector T cell pool. J Immunol. 2013;191: 3025–[illegible]

48 Lee DF, Salguero FJ, Grainger D, Francis RJ, MacLellan-Gibson K, Chambers MA. Isolation and characterisation of alveolar type II pneumocytes from adult bovine lung. Sci Rep. 2018;8: 11927.

49 Buckley ST, Ehrhardt C. The receptor for advanced glycation end products (RAGE) and the lung. J Biomed Biotechnol. 2010;2010: 917108.

50 Garcia-de-Alba C, Pessina P, Kim CF. A new "age"r for lung research arrives: Genetic targeting of alveolar type 1 epithelial cells. American journal of respiratory cell and molecular biology. American Thoracic Society; 2018. pp. 661–662.

51 Yu DH, Ruan X-L, Huang J-Y, Liu X-P, Ma H-L, Chen C, et al. Analysis of the Interaction Network of Hub miRNAs-Hub Genes, Being Involved in Idiopathic Pulmonary Fibers and Its Emerging Role in Non-small Cell Lung Cancer. Front Genet. 2020;11: [illegible]

52 Manon-Jensen T, Karsdal MA. Chapter 14 - Type XIV Collagen. In: Karsdal MA, editor. Biochemistry of Collagens, Laminins and Elastin. Academic Press; 2016. pp. 93–95.

53 Kwartler CS, Chen J, Thakur D, Li S, Baskin K, Wang S, et al. Overexpression of smooth muscle myosin heavy chain leads to activation of the unfolded protein response and autophagic turnover of thick filament-associated proteins in vascular smooth [illegible]

54 Cabezudo D, Baekelandt V, Lobbestael E. Multiple-Hit Hypothesis in Parkinson's Disease: LRRK2 and Inflammation. Front Neurosci. 2020;14: 376.

55 Kanatsuka A, Kou S, Makino H. IAPP/amylin and β-cell failure: implication of the risk factors of type 2 diabetes. Diabetol Int. 2018;9: 143–157.

56 Bosma KJ, Rahim M, Oeser JK, McGuinness OP, Young JD, O'Brien RM. G6PC2 confers protection against hypoglycemia upon ketogenic diet feeding and prolonged fasting. Mol Metab. 2020;41: 101043.

57 Briant L, Salehi A, Vergari E, Zhang Q, Rorsman P. Glucagon secretion from pancreatic α-cells. Ups J Med Sci. 2016;121: 113–119.

58 Hauge-Evans AC, King AJ, Carmignac D, Richardson CC, Robinson ICAF, Low MJ, et al. Somatostatin secreted by islet delta-cells fulfills multiple roles as a paracrine regulator of islet function. Diabetes. 2009;58: 403–411.

59 Ludvigsen E, Olsson R, Stridsberg M, Janson ET, Sandler S. Expression and distribution of somatostatin receptor subtypes in the pancreatic islets of mice and rats. J Histochem Cytochem. 2004;52: 391–400.

60 Perez-Frances M, van Gurp L, Abate MV, Cigliola V, Furuyama K, Bru-Tari E, et al. Pancreatic Ppy-expressing γ-cells display mixed phenotypic traits and the adaptive plasticity to engage insulin production. Nat Commun. 2021;12: 4458.

61 Aston C, Jiang L, Sokolov BP. Transcriptional profiling reveals evidence for signaling and oligodendroglial abnormalities in the temporal cortex from patients with major depressive disorder. Mol Psychiatry. 2005;10: 309–322.

62 Wallensten J, Nager A, Åsberg M, Borg K, Beser A, Wilczek A, et al. Leakage of astrocyte-derived extracellular vesicles in stress-induced exhaustion disorder: a cross-sectional study. Sci Rep. 2021;11: 2009.

63 Sloan SA, Darmanis S, Huber N, Khan TA, Birey F, Caneda C, et al. Human Astrocyte Maturation Captured in 3D Cerebral Cortical Spheroids Derived from Pluripotent Stem Cells. Neuron. 2017;95: 779–790.e6.

64 Barnes MJ, Griseri T, Johnson AMF, Young W, Powrie F, Izcue A. CTLA-4 promotes Foxp3 induction and regulatory T cell accumulation in the intestinal lamina propria. Mucosal Immunol. 2013;6: 324–334.

65 Belarif L, Mary C, Jacquemont L, Mai HL, Danger R, Hervouet J, et al. IL-7 receptor blockade blunts antigen-specific memory T cell responses and chronic inflammation in primates. Nat Commun. 2018;9: 4483.

66 Patil VS, Madrigal A, Schmiedel BJ, Clarke J, O'Rourke P, de Silva AD, et al. Precursors of human CD4+ cytotoxic T lymphocytes identified by single-cell transcriptome analysis. Sci Immunol. 2018;3. doi:10.1126/sciimmunol.aan8664

67 Phatarpekar PV, Overlee BL, Leehan A, Wilton KM, Ham H, Billadeau DD. The septin cytoskeleton regulates natural killer cell lytic granule release. J Cell Biol. 2020;219. doi:10.1083/jcb.202002145

68 Yang C, Siebert JR, Burns R, Gerbec ZJ, Bonacci B, Rymaszewski A, et al. Heterogeneity of human bone marrow and blood natural killer cells defined by single-cell transcriptome. Nat Commun. 2019;10: 3931.

69 Swatek AM, Lynch TJ, Crooke AK, Anderson PJ, Tyler SR, Brooks L, et al. Depletion of Airway Submucosal Glands and TP63+KRT5+ Basal Cells in Obliterative Bronchiolitis. Am J Respir Crit Care Med. 2018;197: 1045–1057.

70 Naizhen X, Kido T, Yokoyama S, Linnoila RI, Kimura S. Spatiotemporal Expression of Three Secretoglobin Proteins, SCGB1A1, SCGB3A1, and SCGB3A2, in Mouse Airway Epithelia. J Histochem Cytochem. 2019;67: 453–463.

Supplementary Table 3. Top 50 Cell Type Marker Genes

CellType	Top 50 Genes (Sorted Left To Right Descending)																																																	
3t3	PROKR1	EN1	OSR1	PRKG2	DLX1	IGLON5	PRRX2	LOX	PRRX1	GXYLT2	ASPN	GAS1	MRGPRD	CRABP1	GNG8	FGF7	TSPAN11	POSTN	SLC1A3	GAS2	AHNAK2	PTN	CD248	GLIS1	SLC46A2	WFDC12	HOXA2	GHR	SNED1	KLHDC8A	BGN	ADAMTSL5	FBLN2	EBF1	PTX3	AEBP1	ADAMTS5	THBS2	HOXA7	LHX8	AZIN2	FGF10	CRLF1	IL1RL1	SHOX2	P3H3	PTGFR	PTGIS	ASB4	CAPN6
42mgba	LCE2A	SIGLEC15	GDF5	TEX29	CCL26	SPOCD1	MAGEA1	STC2	MMP3	MAGEA3	TGFBR3L	THY1	LOXL1	MRGPRF	HOXD11	GDNF	FST	CPA4	HMSD	OLFML3	DOK5	KCNMA1	LRRC15	ARHGAP22	TMEM171	APOBEC3B	BATF3	NGF	NKX2-5	OASL	WNT7B	MPP4	TMEM45A	STRA6	CD163L1	TRIM61	FAM180A	NRN1	KISS1	KCNG1	NPW	NEXN	PDCD1LG2	HES7	ZP1	JPH2	ALPK2	CBR3	PDLIM4	SPHK1
451lu	UCN2	TRIM51	TM4SF19	MMP20	BIRC7	MLANA	SLC45A2	HCG22	PMEL	TYR	PRAME	FRG2	EIF3CL	PRDM7	NBPF8	NUDT4B	CENPVL2	OPRD1	APOBEC3B	KRTAP4-9	CAPN3	DCT	TAS2R20	GPR143	OR9G1	MMP8	FGF23	LHX8	GOLGA6C	C1GALT1C1L	BAAT	POTEE	CSAG3	KRTAP4-12	TNFSF13B	MAGED4	MC1R	LCE2A	CA8	TBC1D3E	DDTL	FAM156B	COL19A1	PIGY	ATP6V0D2	NPIPB3	CENPVL1	MAGED4B	GSTT2B	SERF1B
8305c	LCN1	MMP1	SIGLEC15	KRT33B	PAEP	APCDD1L	PAGE5	CSF3	SPANXN3	CLDN14	SPOCD1	SPANXD	GABBR2	SIRPB1	KRTAP2-3	TFPI2	PRSS3	KRT34	KCNG1	BNC1	CXCL5	OR2B6	ELFN2	FGF5	HS3ST3A1	VSTM1	KIAA1549L	FOXD1	GREM1	LAYN	PAX8	TOX2	ADAMTS6	ARHGAP22	STEAP1B	CSAG1	HAS2	IFNE	VEPH1	TRIML2	G0S2	CLEC11A	TMEM158	ADTRP	KCNMA1	FUT10	CACNG8	SPOCK1	INHBA	LARP6
a204	CRH	CLDN20	ITGA10	FGFBP2	NPTX1	HHLA2	HPD	COL11A1	HGF	SPAAR	OXTR	BAALC	PRND	RTL9	FIBIN	BARX1	NRN1	OTP	MYH15	TRDN	C1QL4	ALDH8A1	SP9	ABCC2	MAMSTR	CYP27C1	FAM43B	SCG2	FOXL2	CA11	KCNQ2	DCDC1	PRRX1	FBXO27	MYBL1	DCAF8L2	TRPM3	MIA	CDH8	MMP16	BFSP1	PAPPA	CYP24A1	CUBN	FGFBP1	SIX1	PALM3	TBX2	MGP	DNER
a375	CT45A1	MAGEA2B	CTAG1A	CSAG3	MRGPRX4	MAGEA2	CT45A9	CTAG1B	SSX2B	CT45A10	SPATA31C2	SPANXA2	SAGE1	SPANXA1	MAGEC2	IL13RA2	MAGEA11	XAGE1B	CT45A3	MAGEA4	PAGE5	PAGE2	SSX2	TP53TG3C	NXF2B	FOXR2	OR51B4	UCN2	POTEM	SPANXB1	MAGEA12	VCX2	SOHLH2	OR51B6	EIF3CL	TP53TG3B	OR51B2	OR51H5	NAA11	CT45A5	IL24	GAGE12F	TRIM51	NUDT4B	OR51I1	FAM156B	MAGEA6	ANKRD30A	MIA	MAGEA3
a549	XAGE1B	H2AB3	GGTLC2	H4C12	XAGE1A	TM4SF20	ARSL	RAMACL	INSL4	CYP24A1	ANTKMT	SNORC	MUC5AC	H2AC14	RAB6D	PLAAT3	SERF1B	EOLA2	H2AC18	AKR1C2	EEF1AKMT4	MTLN	AKR1B10	H4C3	F8A3	RBIS	H2AC20	VCY	AKR1B15	KRT81	ALDH3A1	H1-3	SLC66A3	GAGE12D	TP53TG3	RTN4RL2	EIF3CL	NTAQ1	FAM174C	AKR1C3	ATP5MJ	ZC3H11B	LGSN	AKR1C1	H2AZ1	NR0B1	H1-2	BOLA2B	H2AZ2	AOPEP
abc1	IGFL2	TCHHL1	VGLL1	XAGE3	GGTLC1	CST5	VCX2	ALPP	PLEKHG4B	NPPB	UPK2	VCX	KRT80	IL36RN	CLDN6	PASD1	PXDNL	UPK1B	VCX3B	WNT6	SPRR3	VCX3A	TMPRSS13	WNT10A	ERVW-1	CYP1B1	CPA4	HRK	KRT3	DEFB126	PTGES	LIPH	SUN3	CLIC3	OR51I1	ANKRD1	ZNF530	IVL	CST6	KRTAP2-3	GPR87	MACC1	IGF2	BATF	KRT9	GJA5	LRTM2	S100A14	OPN3	PPP1R1C
acinar cell	CELA2B	AMY1A	AMY1B	CTRB2	CELA3A	AMY2A	CTRC	REG1B	CELA3B	CTRB1	PNLIPRP1	PNLIP	PRSS1	SYCN	CLPS	PRSS2	RBPJL	CPB1	GP2	CPA1	OR11G2	PLA2G1B	CELA2A	CPA2	REG1A	REG3A	CUZD1	CEL	CPA5	PNLIPRP2	PRB1	REG3G	CLDN22	PTF1A	PDIA2	CTRL	SERPINI2	LPO	CYP4A22	AMY2B	AMY1C	HNRNPCL4	AQP8	SERPINA3	MT1HL1	AQP12B	SERPINA6	SERPINA4	IL22RA1	ERP27
activated cd4-positive, alpha-beta t cell	OR2A25	OR11G2	ZNF831	IL22	CD40LG	KLRB1	OR3A3	OR4D9	TRAT1	ICOS	H2AB3	GPR183	OR2AP1	RGL4	IL2	OR4D1	SEPTIN1	OR6C4	CCR6	EOLA1	GPR174	P2RY10	H2AC7	CD69	IL7R	H2AC17	CD2	PTGER4	SIRPG	CD3D	CD28	CCR4	IL23A	CD6	IL17A	CD3E	H2BC10	OR5H14	GCSAM	OR7A17	TNFRSF4	H4C2	CXCR4	THEMIS	RGS1	CD96	IL23R	ITK	GIMAP7	PLAAT4
activated cd8-positive, alpha-beta t cell	ZNF80	SEPTIN1	H2BC8	H4C1	H4C6	GZMK	H2BC15	H2BC7	H4C13	H4C2	H2BC10	KLRC4	SHFL	H2BC6	H2AB3	H4C5	KIR3DL3	H2AC15	XCL2	CD8B	H2BC14	DTHD1	KLRG1	CD160	H2AC16	CD8A	H2BC9	H3-3A	H2AC7	ANTKMT	FASLG	FCRL6	H2AC8	CRTAM	TIGIT	H1-3	H2BC3	SH2D1A	RESF1	H2BC21	GZMM	SLC66A3	ATP5MGL	FAM174C	RTP5	GZMA	PLAAT4	H3C1	PTGDR	PYHIN1
adipocyte of breast	ADIPOQ	CDSN	CIDEC	HCAR1	NYX	PLIN1	TMEM72	PLIN4	UBTFL1	RFPL4A	SPAAR	ITIH4	SLC10A2	AQP7	TEX13B	ANKRD61	FLG2	SLC36A2	ADIG	MMP12	GRIP2	RTP3	DGAT2L6	PROX2	TRARG1	LGALS12	LRRC14B	ADRA1D	GPR152	CHRDL1	ULBP1	NPFFR1	GRK1	CSN2	KRT36	HSD3B2	DHRS9	PCDH12	ADHFE1	FABP4	RETN	DOCK10	CCIN	MROH2A	SAMD7	CEACAM20	GPD1	A3GALT2	AIRE	AOC3
adult endothelial progenitor cell	CRYGN	TMEM212	CCDC153	GOT1L1	MT3	RIIAD1	CFAP126	CFAP161	CCDC33	CFAP77	CDHR4	EFCAB10	NME9	LRRC74B	MEIG1	SNTN	MRO	FAM216B	CFAP206	VWA5B1	PRR29	ANKRD66	DYNLRB2	DNAH12	STOML3	CATIP	FRMPD2	SLC25A18	TEKT4	SLC35G3	FAM166B	SLC14A2	CFAP100	PPP1R32	CROCC2	RSPH10B	MLC1	CABCOCO1	DRC7	GHRH	ADAMTS20	MIA	DTHD1	MORN5	GPR37L1	PRR18	RSPH4A	CFAP54	FOXJ1	AK7
adventitial cell	SCYGR10	TAF11L11	PPIAL4F	OR4A8	OR2K2	ZSCAN5C	KRTAP4-2	USP41	OR13J1	TEX54	OR4D6	HSFX1	SUPT20HL1	SUPT20HL2	OR2C1	AADACL2	OR5K2	SMIM41	OR2AE1	HSFX2	GASK1A	FAM180B	REELD1	FGF16	OR5B21	OR10Z1	CCDC195	OR4K1	IFNW1	SPDYE18	AICDA	PYDC1	PPIAL4C	PNMA6E	PLA2G5	CDC14C	RTL1	CYP4Z1	ANGPTL5	SPDYE2B	GPRC5D	VIT	LBHD2	SCARA5	SPDYE6	MYOC	XPNPEP2	VSIG10L2	TULP2	PODN
ael	IFIT1B	HBD	GYPB	CA1	AHSP	HBM	HBB	RHCE	HBA1	HBA2	GYPE	GYPA	RHAG	TSPO2	ALAS2	SLC22A16	HBG1	HEMGN	SPTA1	EPB42	THEM5	TRIM10	SLC4A1	KLF1	HBQ1	HBZ	HBG2	RFPL2	AZU1	KEL	RIPOR3	TRIM58	YPEL4	OR2W3	RHD	GATA1	SLFN14	MYL4	STMP1	TUBB1	CA2	TAC3	OSBP2	GFI1B	EPOR	SNCA	TFR2	ATP5MGL	MFSD2B	SMIM5
alpha-beta t cell	KRTAP9-1	IZUMO1R	CBLIF	NTN3	CCL1	IFNG	CXCR6	IL12B	ZNF683	XCL1	CD3G	CCR8	XCL2	MAGEA2	IL2	CD28	CD3D	ITK	ICOS	SPRR2B	H4C7	CD40LG	TXK	CD8B	CD3E	CD2	CD5	SIRPG	IL4	KLRC1	THEMIS	AIRE	FOXP3	TRAT1	SUPT20HL1	SH2D1A	KRT2	IL13	IL2RA	ODAPH	CD8A	PVRIG	CD247	CASP14	GPR50	CTLA4	H3C7	IL2RB	IL7R	KLRK1
alternatively activated macrophage	VSIG4	TBC1D3B	FPR3	MS4A4A	FCGR1A	MARCO	TREM2	CD163	MSR1	MS4A6A	FCGR2A	MS4A7	FCGR2C	CCL18	MCEMP1	OLR1	HAMP	OSCAR	C3AR1	FCGR3A	MS4A14	CD209	SLAMF8	C1QA	ZNF705A	C1QC	TREM1	C1QB	FOLR2	CCL13	SIGLEC9	SIGLEC8	CD86	GH1	TLR8	SIGLEC7	LILRB5	SLC11A1	SIGLEC1	CLEC7A	HLA-DQA2	SDS	SLCO2B1	C5AR1	HLA-DQB2	CD14	RNASE2	OSM	DNASE2B	MNDA
alveolar macrophage	INSYN2B	OR6N1	CCDC200	ANKUB1	CCL18	OVCH1	SHOC1	TPRG1	MARCO	GLDN	SIRPB2	OR1L6	RXFP2	MSR1	SLC11A1	OR6N2	CD163	STAC	FPR3	INKA2	FLVCR2	MRC1	KCNE1	TEX52	OLR1	MS4A4E	STPG4	TFEC	SLCO2B1	ZNF438	HS3ST2	SNTB1	EDDM13	PIK3R5	ARMH3	MCF2L2	OSCAR	TM4SF19	LRMDA	ALOX5	SEPTIN9	ITGAX	AQP9	NIBAN1	SIGLEC1	MS4A14	CIITA	WDR49	PPARG	DCSTAMP
amacrine cell	PRR35	TAAR9	NAT8B	C1QL2	GSG1L2	ZNF648	SMIM28	RFPL1	RD3L	KLHL33	CHRNB3	GLRA3	PRDM13	CHRNA2	BARHL2	SLC32A1	LACTBL1	PABPC1L2B	LRRC3C	POU4F2	ZNF593OS	SNCB	TMSB4Y	CARTPT	MAB21L1	TFAP2D	CACNG3	CPLX3	NXPH2	FKBP1C	BHLHE23	SIX3	PAX6	RCVRN	SYNPR	LNP1	TRIM64	TFAP2B	HMX1	MINDY4B	LRTM2	IGLON5	NT5C1A	ZNF385C	IQSEC3	GLRA2	RGS8	RORB	MGARP	SIX6
ameloblast	PANX3	TNN	ODAM	ADGRF4	IBSP	AMBN	SP7	OCSTAMP	LRRC15	KLKB1	DYNAP	PTX4	ENPP6	DLX3	MRGPRE	ESYT3	BMP15	PLA2G2D	ANGPT4	WIF1	CARTPT	RSPO4	KRT27	OMD	ENPP3	FOXF2	PTGFR	IFNK	ALX1	SLAMF9	IL1RL2	LGR6	ALX3	TLR8	SLC26A1	DIO3	ADAMTS20	LHX8	BTNL2	PRND	TMEM26	PRSS35	SP6	WFIKKN2	SLC7A13	ANXA8	NLRC4	VIT	RTL3	SLC5A8
amnion	HAND1	TDO2	FOXF1	PRDM6	CFC1	LRRN4	CBLN1	PLAC1	FAM162B	AARD	HOXB1	C1QTNF12	MOGAT2	STARD8	TRPC5OS	PITX2	POPDC2	TBX20	HAPLN1	APLNR	FGF3	CRB2	TMEM108	TMEM88	BCO1	CRYGN	MSX2	DLK1	GDF2	COLEC11	WNT2	DUSP9	RIPOR3	VNN1	GDF6	BHLHA9	KLF14	ASB4	FIGNL2	HAND2	ASB12	SLC38A4	HSD11B2	PRTG	SLC1A6	HOXB6	LIN28A	BMP4	MSX1	NUP62CL
aortic endothelial cell	RTP3	BTNL9	TAS1R2	ESM1	FAM167B	SOX18	CD300LG	GPIHBP1	GPR182	TCHHL1	TMEM26	KDR	THRSP	MMRN2	EMCN	TBXA2R	FLT4	PLVAP	CLEC1A	SRMS	EHD3	TIE1	USHBP1	LIPK	RAMP3	OIT3	RSAD2	GPR4	FAM110D	TEK	DLL4	PLSCR2	LRRC3B	TMEM204	ROBO4	NPR3	FLT1	ARHGEF15	ENPEP	PTPRB	ADGRL4	GIPC3	RASIP1	RIPPLY3	ESAM	CLEC14A	PTPRU	EDNRB	TMEM88	POM121L2
aspc1	IGFBP1	KLK11	CREB3L3	PRSS21	FGF19	KLK10	SLC15A1	IGFL2	PRSS3	COL17A1	CEACAM6	FAM83A	UPK3B	SPAAR	HKDC1	HTR1D	MROH6	FOXL1	RFLNA	C4BPB	HHLA2	MOGAT3	DHRS9	NGEF	VNN1	HOXC11	FERMT1	STEAP1	MMP7	BAIAP2L1	ASPHD1	TM4SF18	P2RY2	NOS3	CDA	SLC6A20	USH1C	SHH	LMTK3	GNG4	CALB2	ARTN	GPR35	SMURF1	LGALS4	F12	MALL	CCDC68	WNT7B	B3GALT5
astrocyte	NTSR2	SLC7A10	SLC39A12	SLC6A11	ETNPPL	SLCO1C1	DBX2	ITIH3	GPR37L1	BCAN	MMD2	GFAP	SLC1A2	AQP4	HEPACAM	SLC25A18	KCNJ10	MLC1	GABRG1	GJB6	ATP1A2	BTBD17	RFX4	SLC6A1	PSD2	GRIN2C	ADCYAP1R1	DIPK1C	CMTM5	RPEL1	HES5	GRM3	SLC1A3	CSPG5	PROP1	TTYH1	SLC38A3	ATP13A4	NCAN	CDH20	TRIL	NR2E1	PRLHR	LUZP2	ADGRB1	TMEM229A	PTPRZ1	GABRB1	AGT	TIMP4
auditory hair cell	KCNA10	LRRC3C	POU4F3	LCE6A	RNF225	LOXHD1	CDCP2	GRID2IP	ATOH1	OTOL1	BARHL1	CCER2	OTOF	CIB3	PTPRQ	SKOR2	USH2A	LHX3	CLRN2	OTOG	CABP2	LCT	GRXCR2	P2RX3	SKOR1	CHRNG	DYTN	GIPC3	RASD2	TECTA	OTOGL	NTNG2	GPR156	SH2D4B	STRC	CLEC18A	PROB1	CHRNA9	KCNH6	CFAP65	KNCN	BTNL2	SCN4A	MKRN2OS	HHLA1	PCDH20	KCNQ4	MYO1H	SLC8A2	SERPINE3
autonomic neuron	PHOX2A	RAB41	TLX2	SCRT2	UNCX	RIPPLY1	MAB21L2	SHOX	ONECUT1	BCL2L10	NOVA2	GAPDHS	POU4F2	PSMA8	STX1B	SNCB	MAB21L1	NKAIN1	DRGX	ZC4H2	NAT8L	FNDC10	ELAVL3	GBX1	FOXO3B	RIMKLA	RNF175	DBH	ZSWIM5	CHAC1	PBX4	EGR4	PRR18	COX6B2	TFAP2B	DRAXIN	GNG3	CYP26C1	FABP3	NAT16	PSD2	TMIE	GLRA1	ELAVL4	ST8SIA5	GPR85	CADM4	POU4F1	SCML2	TP53INP2
b cell	MS4A1	FCRL1	FCER2	BLK	PAX5	FCRL2	CLEC17A	FCRL4	TLR10	BANK1	HLA-DOB	NIBAN3	FCRL5	TNFRSF13C	CD19	CD22	CD79A	FCRLA	CXCR5	FCRL3	VPREB3	TCL1A	CD79B	LCE2B	BTLA	TNFRSF13B	INSYN2B	CLEC20A	FCMR	POU2AF1	RUBCNL	OR2T11	TEX52	FFAR1	RHEX	IGLL5	LCE2C	HLA-DQA1	CD180	SPIB	HLA-DQA2	P2RX5	HLA-DOA	WDFY4	ADAM28	PARP15	LCN10	CIITA	CNR2	CCR7
b-1a b cell	OOSP1	PRG2	CACNA1S	TNFRSF17	JCHAIN	MZB1	LAX1	DERL3	BHLHA15	SRMS	TNFRSF13B	CCR10	CD28	SLAMF7	EAF2	CD79A	POU2AF1	USH1G	CTLA4	GPR21	ZBP1	FCRLA	CRLF2	LY6D	CD79B	CRYBA4	ST8SIA6	SLAMF6	ANKRD55	BLNK	IL5RA	TXNDC5	NUGGC	ENDOU	IZUMO1	TXNDC11	SLAMF9	RNASE6	BTLA	EDEM1	PON3	EVI2A	CEACAM16	VPREB3	GPR18	NLRP10	HCRTR2	PRSS57	IRF4	CDSL
b-all	TCL1B	RTP5	OR2AK2	OR11L1	MPIG6B	ARMH1	RHEX	IGLL1	TTC24	DNTT	TCL1A	HMHB1	USP41	VPREB1	CLEC20A	MYO18B	PAX5	SPANXN1	VPREB3	PTCH2	OR10Z1	KRTAP4-12	RAG2	CD19	SERHL2	OR8B12	PPIAL4A	SCN4A	WASHC1	SLC22A16	CD79A	ERG	WDR49	HLA-DOA	RAG1	GAL3ST2	GCSAM	MZB1	HLA-DRB5	ZC3H11B	CHRFAM7A	PRSS57	CYBC1	CALHM6	TPO	NLRP4	TBL1Y	PLEKHG4B	HSH2D	PARP15
basal cell	MRGPRX3	ECRG4	MMP10	IL20	SERPINB3	SERPINB4	UBE2L5	GJB4	SERPINB13	IL17B	HCAR3	LGALS7B	CLCA2	OR2A1	LBHD2	WFDC5	ADH7	KRT17	TP53AIP1	KRT5	TMPRSS11D	RNASE7	KRT15	SERPINB5	BPIFA1	CXCL17	DLK2	KRT31	FMO3	HCAR2	KRT14	KLK11	RNF39	COL17A1	KRT71	ADH1C	AQP5	LGALS7	CTAGE8	CHI3L1	KRT13	CSF3	SERPINB10	RASSF10	CYP2A13	SCGB3A1	GSDMC	ANXA8L1	NTF4	SYT8
basal cell of epidermis	LCE1D	LCE1B	THEM5	KRT75	MT4	KRTAP13-1	LCE1C	KPRP	KRTAP3-3	CHIT1	KRT77	LIPM	CCL27	KRT24	VSIG8	LCE1F	KRT36	TMPRSS11A	WFDC5	KRT84	LGALS7	LORICRIN	KRTDAP	ANXA8	LTB4R2	DSC3	KCNK7	LCE1E	COL17A1	SLURP2	PSAPL1	LAMB4	KRT5	DSG1	FAM83C	OTOP3	KRT15	CLDN17	KRT32	PNLIPRP3	SCGB2A2	PKP1	WNT10A	CARD18	KRT31	ADH7	DSC1	GDPD2	KLK9	KRT1
basal cell of olfactory epithelium	MMP10	RTP2	CNGA2	CNGA4	UGT2A1	SCGB1C1	RTP1	ADH7	ALOX15	OBP2A	FMO3	UMODL1	SERPINB4	FAT2	REG3G	TAAR2	ELOA2	MRGPRG	AQP4	PAX7	OBP2B	SERPINB10	SRD5A2	FMO2	KIRREL2	S100A5	ALDH3A1	ATP13A5	SULT1E1	GADL1	NOS2	DMRT3	KRT5	KCNH4	NR2E1	CCDC3	SFTPD	DSC3	CAPN13	KRT15	SERPINB13	NTF4	RD3	TP63	FETUB	SLC26A7	CNGB1	SIX2	AQP3	KRT74
basal cell of prostate epithelium	KRTAP8-1	KRTAP3-1	IFNA4	SRY	KRTAP9-1	OPN4	IFNA7	IFNA5	USH1G	KRT2	KRTAP1-1	KRTAP4-12	CHRNA7	TINAG	RNASE7	TRHR	HCAR3	CXCL8	KRT23	EPX	IL9	NKX2-2	PRAC1	FUT4	KRT37	SLC14A1	GUCY2D	KRT15	IL12B	TACR3	KRTAP12-1	FAM110C	TGM4	EDN2	TACSTD2	WNT8B	CNGA2	KRTAP13-2	KLK3	ARX	MMP10	MMP7	HOXB13	PRAC2	KRT5	KRT17	DEFB1	PADI3	RAX	DUOXA2
basal cell of urothelium	KRTAP3-2	KRTAP4-16	KRTAP17-1	KRT39	LORICRIN	HOXD12	SPRR1A	KRT75	SHH	HOXD13	CRYBB3	OVOL1	EPGN	KRT15	KRT33A	SOWAHB	LCE1B	IVL	GRHL3	CPA6	SBSPON	KRTAP5-4	ADGRB1	HOXA13	UPK3BL1	NPPC	FOXQ1	WNT6	KRT20	CERS3	SP8	LANCL3	TAS1R2	LGALS7	KRT36	FOXA1	LCE3C	KRT14	KRT5	ARC	RIPK4	SALL3	LYPD2	EVX1	FAM110C	OTOP1	RASSF10	WNT4	FOXN1	KRT4
basal epithelial cell of tracheobronchial tree	SCGB3A2	REG3G	BPIFA1	ADH7	FMO3	LYPD2	SFTPD	LIPF	SEC14L3	KRTAP3-2	KRT75	CHAD	LMAN1L	FGF16	SCGB1A1	KRT4	AQP5	SOX21	GSTA3	CLCA4	KRT15	KCNJ13	GABRP	SLC16A11	SCGB3A1	CYTL1	PGLYRP1	ANXA8	TMPRSS11A	VSIG8	SFTPA1	GDPD2	LTF	NKX2-1	AQP4	HTR6	A3GALT2	MSLN	CYP4B1	PADI1	FAM110C	KRTAP17-1	WNT4	C2CD4B	GJB4	PON1	PSAPL1	SPINT3	LRRC26	DAPL1
basophil	TPSD1	TPSAB1	TPSB2	MS4A2	CPA3	SIGLEC8	RHEX	RGS13	GCSAML	SIGLEC6	IL4	IL1RL1	DNASE2B	FCER1A	OR2A4	RAB44	HRH4	ERVFRD-1	MAS1L	OR5C1	OR2G2	OR1L6	SLC18A2	NTRK1	RNASE12	TMEM233	HPGDS	ATP1A4	OSM	CDK15	IL18RAP	GRM6	ALOX15	HDC	PRG2	INKA1	OR1B1	LTC4S	CD200R1	C3AR1	PIK3R6	TPSG1	ADCYAP1	ADGRE2	CSF2RB	GATA2	ALOX5	OR2T8	ADORA3	MAOB
basophil mast progenitor cell	CMA1	TPSB2	RAB44	IL4	MC5R	ADORA3	TMPRSS7	FUT7	RAB19	CPA3	SLC45A3	FCER1A	CYP11A1	GZMB	RAPSN	SCIN	IL9R	SLC34A2	ADGRG3	SLC6A12	TPH1	MRGPRE	FCGR3B	RGS18	BFSP2	SLC22A3	SLC6A13	ITGA2B	RAB37	SOAT2	KIT	ITGB7	LACTBL1	GATA2	ST8SIA6	P2RX7	NRROS	GPR34	NDST2	GATA1	ANGPT1	TEX13B	SLC30A2	CSF2RB	OIT3	P2RX1	RAPGEF3	SUCNR1	SPNS3	GNAZ
basophil progenitor cell	MS4A2	RNASE12	RAB44	FGF3	CPA3	FCER1A	FUT7	IL4	IL18RAP	ADGRG3	ALOX15	MPO	ITGA2B	SIGLEC5	GRM6	HK3	HGF	SLC22A3	TMEM150B	TAS1R3	SPNS3	CD200R1	SOAT2	PLCB2	CHST13	PADI2	CNR2	CD244	FCGR3B	ARAP3	CSF2RB	CARD9	CLEC12A	CCR1	HTR1B	SEC16B	CYP11A1	MC5R	CD300LB	MFSD13A	E2F8	PTGER1	ABCA2	BTNL9	GPR34	TFR2	RGS18	NFAM1	UBASH3A	PNPLA7
bcc	IL20	KRT85	DSG1	KRT75	FOXI3	S100A7	LGALS7B	GJB6	KRTAP20-2	CLEC2A	KRT37	KRT6B	KRTAP19-3	ABCC12	KRT16	KRTAP21-2	CASP14	ZNF750	GJB2	DSC3	ADGRF4	RAET1L	LCE1C	TP63	S100A7A	IGFL1	IRX4	CHI3L1	KRT17	CALML5	KRT33A	EDAR	FAM83B	KRT14	GRHL3	ZNF679	FRG2	CARD18	GSDMC	KRTAP17-1	PGLYRP4	PTCH2	KRT5	DSG3	POTEF	WFDC5	CYP2W1	ANOS1	HCAR3	DSC1
bcpap	COLEC10	MMP1	CXCL6	KRTAP2-3	SPANXD	LRRC38	CATSPER1	NAA11	MAGEB2	IL13RA2	CPA4	SPANXA2	OR51B4	FAM133A	MAGEA6	STATH	SERPINB7	VSTM1	LHX1	SIGLEC15	PYDC2	ADRA1D	ESM1	MAGEA3	FGF5	CCBE1	CYP4F11	TRIML2	APCDD1L	AJAP1	PRSS3	INHBA	MAGEA11	CARD18	KRT81	FOXL1	SLC17A9	ADAMTS12	RNF212	GDNF	ARSI	TGM2	DKK3	CDA	MYEOV	SPOCD1	TMPRSS11F	NPFFR2	S100A2	ATAD3B
bergmann glial cell	NTSR2	SLC7A10	CTXN3	GPR37L1	SLC39A12	CACNG5	ETNPPL	ITIH3	TIMP4	LCAT	SLC25A18	DBX2	BTBD17	GDF10	CMTM5	HEPACAM	GJB6	CDH22	KCNJ10	MLC1	LRTM2	AQP4	PAQR6	FAM107A	MMD2	SYT10	LUZP2	SLC1A3	GPLD1	SLC6A1	DAO	PLA2G7	TRIL	DMP1	FAM181B	MRO	LGI4	GRIA1	OMG	CPSF4L	GRIA4	GJC3	ATP1A2	SLC1A3	CASKIN1	HSD11B1	ADCYAP1R1	KCNN2	SLC38A3	
bftc909	SPANXN5	NOX5	SUN3	SIGLEC15	CD70	BAALC	TM4SF19	MSC	TRIML2	CACNA2D4	ARSI	LHX1	CHI3L2	FHAD1	IGFN1	LCTL	INHBA	TRIM16L	FOXP4	HS3ST3A1	HRCT1	SAA1	FBXO32	C1QL1	PDCD1LG2	NUTM2E	ATAD3B	FAT2	FOXS1	FABP6	ZP1	MYEOV	TAF8	PLAU	ERFE	LCE2A	DGKI	CTHRC1	PAX8	RASIP1	TFEB	P2RY6	CIBAR1	BYSL	APCDD1L	WNT2B	NR0B1	STEAP1B	PRICKLE4	FGF1
bicr16	LCE1F	LCE1C	LCE3D	SPINK6	SPRR1B	IGFL1	CALML3	IL1F10	LCE1E	SPRR2D	KRT31	KRT6A	OTOP3	TP53AIP1	CLCA2	KRT6B	RAET1G	SERPINB13	ZBED2	PTHLH	KRT5	IL20RB	HEPHL1	KLK10	KRT14	ARTN	LCE3E	SPRR2A	COL17A1	GPR87	KHDC1L	CFAP251	ARSI	GJB6	CSTA	TP63	RAET1L	WFDC5	FST	FAM83A	HAS3	BDKRB1	DLK2	FOXL2	FLG	SOX15	ONECUT3	KRT75	H2BW1	KRT16
bicr31	CALML5	KHDC1L	KLK5	NKX2-8	GPR87	KRT6A	CALML3	CST6	HOXD11	SOX15	ZBED2	NTF4	KRT13	DLK2	KRTAP4-1	RNASE7	KRT31	PADI3	S100A2	P2RY2	WDR72	NGB	KLK10	IL1F10	FAM83A	LY6K	CFAP251	CSTA	LCE3E	KRT37	IRX4	KRT74	FST	KRT5	FGFBP1	GJB3	VGLL1	SUN3	GJB6	GJB5	RHOD	HAS3	PTHLH	HMSD	GAL	WNT7A	SPRR1B	MAGEA11	TP53AIP1	NT5DC4
bipolar neuron	SMIM28	TMEM215	CABP2	AANAT	VSX1	GABRR3	VSX2	CABP5	ZNF593OS	CCL1	EIF4E1B	SLC1A7	CPLX4	LRTM1	RDH8	GSG1L2	RD3L	NEUROD4	OR2A25	LHX3	SPRR2B	TBC1D3K	CA10	TAFA4	OR10K1	GRM6	GABRR1	OR6C75	KCNG4	FCRL4	USH1G	TBC1D3E	CFC1B	RCVRN	PCP2	ZNF385B	GNB3	PRDM8	TMEM196	BHLHE23	TAS2R38	GPR179	OR52N1	ZNF804B	SLC17A7	TRPM1	LACTBL1	CACNA1F	AMER3	PDE6H
bj	PGLYRP3	HNRNPCL4	KRTAP13-1	OXTR	PSG5	PSG4	SSTR1	GREM1	LDLRAD2	LBX1	ATP5MC1	PPIAL4F	UQCRHL	PLPP4	MMP3	PPIAL4A	LSMEM2	RTL8C	TNFRSF6B	ADRA1D	KRTAP1-5	SERPINB7	REN	GJD3	C1QTNF5	SLC35G5	OR51B5	KRTAP2-3	PIGY	ATP5F1C	COL6A3	TWIST2	LOXL2	CD248	KRT34	COL1A2	SSX1	EIF2S3B	HTD2	FANCB	ZNF215	UCN2	ALX4	VEPH1	FOXD1	STMP1	NACA2	NDUFAF8	ULBP3	APCDD1L
bladder cell	KRTAP1-4	ASZ1	WNT2	CPZ	WFDC13	PI16	AVPR1A	PODN	HAS1	ITGA11	SCARA5	LBP	CHODL	DKK2	PAMR1	TNFSF9	WFDC1	NOTUM	BMP3	C1QTNF2	INMT	THBS2	FNDC1	LRRC55	TCF21	NKAIN4	ENTPD2	HHIP	C1QTNF7	GATA5	GJC2	PRDM6	BMP5	HSD11B1	PRELP	KRTDAP	TGM5	GREM2	TBX4	NPY1R	PRSS35	MUSTN1	ADH1A	CTSK	CLEC3B	HOXA11	PTGIS	FBLN5	KAZALD1	C1S
bladder urothelial cell	UPK1A	IVL	KRTAP5-10	SPRR1A	KRTAP5-8	UPK3A	KRTAP5-9	SNX31	OR5M11	KRTAP4-16	TMEM275	UPK2	KRTAP3-2	GRHL3	OR13A1	SPRR2G	SHH	DNMT3L	PSAPL1	FOXQ1	KRTAP4-9	LCE1E	LIPK	IFNE	FMO5	VSIG2	UPK1B	SLC14A1	CERS3	TMPRSS13	DNASE1L2	KRT4	KRT23	SOWAHB	CTSE	OR5P2	TREX2	ALDH3B2	SYT8	KRTAP4-6	KRT15	NIPAL4	FAM110C	LMO1	VSIG10L2	ZSCAN4	ALDH3A1	NIPAL1	KRT37	SLC25A48
blastocyst	DPRX	KHDC3L	MT1B	DPPA5	PRAMEF2	NLRP7	PRAMEF10	PGA4	TRIM60	PRR20C	KDM4E	FTHL17	PRAMEF19	PRR23B	KRTAP12-4	DNMT3L	DPPA3	CTAG1A	THEG	OOEP	OR9G4	SPDYC	OR4K1	ZNF280A	KRTAP2-4	ZNF878	H2AC19	TRIM49B	H2BW1	NLRP2	NANOG	APOBEC3H	VENTX	PRDM14	PRG3	MAGEB1	KRTAP1-4	FOXH1	PRR23C	USP17L8	FAM156B	LIM2	ZNF560	TCL1B	POU5F1	ZYG11A	SLCO1B7	OR1F1	KRTAP13-4	SPIC
blastoderm cell	H1-8	SPDYC	FOXI2	BTG4	ZP4	TUBA4B	NANOS2	ETV3L	H2AC1	KRTAP4-7	NOTO	MKRN3	TAS2R9	CCNA1	SPRR5	MAP1LC3C	NIPAL4	FOXD4L1	NKX1-1	ZP2	SOX3	TAS2R7	TAS2R8	PABPN1L	FOXH1	FIGLA	GDF1	CCNP	NPBWR2	KPNA7	STH	KLF17	QRFPR	OR6T1	HIGD1C	BANF2	FTMT	SNX31	SEC14L5	FAM240B	UPK3A	TENT5B	IRS4	TAS2R13	FAM83C	NPM2	SLC38A8	RLN3	S100Z	PLXNB3
blca	CRCT1	UPK1A	UPK2	CST4	SIGLEC15	AMTN	TAC3	KRTAP3-1	HTN3	UPK1B	SLC14A1	PSCA	CCL15	CST1	CHIA	KRT20	VGLL1	TNFRSF11B	PLA2G2F	RNF223	SPINK1	SIGLEC6	UPK3A	CALHM3	UPK3B	SNX31	KRTAP4-9	GDPD3	HTN1	CA9	DHRS2	SPTSSB	KRTAP4-7	CAPS	KRT17	PSG4	SLITRK6	GUCA2A	ANXA8L1	ULBP2	S100P	RAET1L	MAGEA3	SULT1E1	COL7A1	SNCG	CST6	HSPA6	PLAU	ADIRF
bone marrow cell	TIFAB	MMP8	LILRA6	CCR2	LILRB3	FUT7	PRTN3	MTARC2	GPR141	ELANE	CD177	PPFIA4	PLD4	NAPSA	CLEC9A	SLAMF8	CLEC12A	NFAM1	CD300LD	CTSG	RNASE6	FLT3	CLEC10A	TREX1	UPB1	TREML2	CD300A	TNFAIP8L2	CFP	CEBPE	IRF5	ITGB7	TICAM2	CD300LB	F13A1	TREML4	LY86	NRROS	MPEG1	LRRC25	APOC2	MPO	RASGRP4	KMO	LILRB4	ARL11	PGLYRP1	HCK	MS4A3	NFE2
brain pericyte	GPR20	NRIP2	HIGD1B	DMP1	RASL12	MYOCD	KLHL38	SLC38A11	FOXS1	PLN	MUSTN1	MYH11	GJA4	TMEM200B	LMOD1	SUSD5	CES5A	CRHR2	KCNMB1	CASQ2	NPY1R	HEYL	KCNE4	NGB	PCP4L1	FHL5	ASB2	RGS4	CCDC3	CSPG4	CYSLTR2	KCNJ12	ASB10	ATP13A5	ITGA7	SLC2A4	WTIP	KBTBD13	FBXL22	XIRP1	LRRC10B	EPHX3	KLHL30	GPR21	HSPB2	RGS7BP	SGCA	AOC3	C1QTNF7	KCNK3
brca	SCGB2A2	H1-6	PRR27	LACRT	SMR3B	CLEC3A	CYP4Z1	ANKRD30A	H2AC7	KRTAP5-10	OR52E8	DCD	UGT2B28	CALML5	SCGB1D2	MINDY4B	ANKRD30B	OR52E6	H3C4	SPANXA2	POTEJ	PYDC1	FDCSP	OR8K5	DYNAP	GLYATL2	NAT8B	NBPF6	POTEC	OR4K15	CTAGE15	TBC1D3E	SPANXA1	ITIH6	CLPSL1	OR1J1	OBP2B	CYP4F8	IFIT1B	POTED	H4C8	ESR1	OR13G1	CSN3	TTC6	CT83	NPIPA7	C4A	SLFN14	AQP10
bronchial epithelial cell	NPIPB7	LGALS9B	GSTA5	LGALS9C	NPIPB12	NPIPB9	PRORY	TAAR5	GOLGA8F	CYP2B6	NPIPA9	TUBB8B	NPIPA3	MT1HL1	NPIPB6	RAB6D	PGAP4	NPIPA8	NIBAN2	SCNN1G	NPIPB3	HSFY1	NOMO2	ERN2	PLEKHS1	NOS2	RAB6C	NIBAN1	H2AC18	HIGD2B	PLEKHG7	ST6GALNAC1	NOMO3	VN1R2	HOATZ	INAVA	VTCN1	CTAGE1	CRACDL	MYH7B	CYP2J2	VARS1	H2BC21	OR9Q1	ATP12A	BPIFA2	OTX1	UQCRHL	RUSF1	NPIPA7
bronchial smooth muscle cell	FAM90A26	RPE65	RERGL	KRTAP6-1	SLC16A8	BPI	IGSF22	MYOCD	SGCA	PLN	MYH11	LMOD1	DCD	RGR	FHL5	ZNF70	HRC	CNN1	ADRA2C	PLG	SLC6A20	RASL12	HAPLN2	ADAMTS8	SYNPO2	CASQ2	SULT2A1	KIAA2012	AOC3	COLEC11	UBD	RLBP1	ADRA1D	MAT1A	OXCT2	SLC39A12	SBSPON	ABCA6	LRAT	SLC22A8	FOXS1	MRGPRF	HEY2	CEACAM19	APOC3	CCN5	ITIH3	ANGPTL1	MAP3K7CL	NTRK3
brown fat cell	UCP1	PLIN1	OTOP1	IFNA16	CPT1B	PLIN4	ADIG	ADRB3	ASPG	UBTFL1	GYS2	CIDEC	THRSP	CIDEA	ADIPOQ	PCK1	ZAN	ANGPTL8	GPD1	FRMPD3	MLXIPL	KLHL33	SLC10A2	CCDC187	LGALS12	KRT26	LIPE	SLC36A2	AQP7	TRARG1	KLHDC7A	ADRA1A	UCP3	ZFP92	PLIN5	CPN2	TEX13B	NRG4	TULP1	KCNK4	FMO1	OPLAH	PFKFB1	MIGA2	EXD1	ELOVL3	MRAP	IL17F	PERM1	FABP4
brush border cell of the proximal tubule	GLYAT	MIOX	SLC34A1	GSTA2	HAO2	DAO	TTC36	SLC22A8	TMEM174	ALDH8A1	GUCA2B	SLC17A1	ASPDH	ALDOB	SLC6A18	SPP2	NAT8	PAH	SLC22A12	PCK1	UGT3A2	TMEM252	SLC22A6	SLC6A19	SLC13A1	SLC22A1	SLC17A3	SLC7A9	TMIGD1	MEP1A	GLOD5	HPD	CELA1	SLC51B	HGD	PLSCR2	AADAC	UPB1	AADAT	SPINK1	HAAO	SLC5A2	SLC13A3	FXYD2	SLC3A1	MEP1B	ASPA	GSTA3	ASS1	MOGAT1
brush cell	OR51I2	CYP4Z1	PRSS48	FAM240C	TNFRSF6B	SLC9C1	SLC22A13	CATSPERB	SPACA5	WFDC10B	OR6N1	ALKAL1	SCGB1A1	ATP13A4	TTC6	NIBAN2	GPRC5D	SLC22A7	RAB40A	SHOC1	CCDC200	SCGB3A1	SLC22A31	CAPN8	NKPD1	ERFL	CYP2F1	TP53TG5	DUOXA1	SCNN1G	SLC6A18	BPIFB1	ROS1	CYP2C19	SLC26A8	TMEM272	LDHAL6A	EQTN	FYB2	ANKUB1	DNAH11	ESR2	CAPN13	SLC26A5	ANOS1	PRSS51	MUC5AC	MEF2B	DUOX1	XKR9
cell of epithelium proper of large intestine	LY6G6F	IL25	GNAT3	FFAR3	TRPM5	SH2D7	CEACAM18	TRIM40	SIGLEC5	HMX3	LY6G6D	RGS13	CDHR2	CLDN14	KRTAP3-1	AVIL	CDHR5	EPPK1	LCE1D	GUCY2C	DIO1	MYO1A	IL1F10	FBP2	B4GALNT2	BCL2L14	BMX	HMX2	CD300LF	LRRC66	IL17RB	ESPN	PIK3R5	CHAT	CDX1	LTC4S	EPS8L3	NOX3	MATK	RGS1	ADH1A	ITPRID1	VIL1	B3GNT6	HCK	KRTAP4-9	POU2F3	PRSS53	AGT	ALOX5
bt474	CALML5	ALDH3B2	MUCL1	PNMT	CST4	SPDEF	DHRS2	MMEL1	ARHGAP40	KRT83	IRX4	ALOX15B	ITIH6	TFF3	EPN3	ANKRD30A	RNF224	HOXC11	PLAAT2	SPTSSB	CEACAM6	KRT37	FAM3B	TFF1	ANKRD30B	TFAP2B	ERP27	PHEX	ABCA12	ERBB2	SRCIN1	ZG16B	ZNF467	GRB7	MROH6	ASCL2	PRR15L	C2CD4A	TRPV6	CLPSL1	UGT2B11	CAPN8	S100A14	SPINK8	ABCC13	CREB3L4	HOXC10	MARVELD3	SMIM22	KMO
bt483	MUCL1	S100A7	ALDH3B2	C2CD4A	FGB	CLEC3A	FGG	SPINK8	CLCA2	SPDEF	TEX19	CFAP92	TDO2	KREMEN2	LMTK3	S100A8	TRPV6	NUTM2E	CLPSL2	ABCA12	SLC25A18	SERHL2	TFAP2B	FIBCD1	GNG4	CYP1B1	OAZ3	ABCC11	S100A9	CCR10	CARD14	AKR7A3	S100A7A	PGLYRP4	S100A14	TGFBR3L	TMEM121	RELL2	SMIM22	KISS1R	BRINP3	EFNA3	FGL1	HOXC10	CAPN8	SYNE4	FAM90A1	HRH3	GALNT6	CLPSL1
c32	KRT27	UCN2	PYROXD2	LCE2A	LOXL4	TRPM8	ITIH6	FCGR2A	VGF	S100A3	PNLIPRP3	DCT	PRAC2	CPA2	SPRR2D	FCRLA	MYEOV	S100B	ROPN1B	ANKRD30B	PAX3	ROPN1	BFSP1	F12	NES	NAT16	ETNK2	TP53I11	GDNF	CPED1	PRAME	GJB1	SYNM	BAIAP2L2	LYPD1	RNF175	CYGB	ETV4	SERPINE2	MIA	MOXD1	PRSS33	SLC39A12	GDF15	S100A1	MMP17	IRX6	DUSP23	SEMA6B	MELTF
cajal-retzius cell	NHLH2	TBR1	INSM2	OR4P4	OR6V1	ISLR2	GDF5	SLC17A6	SYNDIG1L	BARHL2	KRTAP4-2	NDNF	CABP7	SCRT1	GRM2	CACNA2D2	NYAP1	BHLHE22	KCNIP2	LHX5	RELN	DMRTA2	TP73	HCN4	NEUROG2	PNOC	CDH4	CCDC177	NHLH1	FOXO6	PSD2	NEUROD6	GPR149	EMX2	EBF3	TMEM163	LHX2	SLC7A10	KCNJ5	CNTN2	CALB2	CAMK2N2	MPPED1	TTC9B	GAP43	LY6H	KCNH7	CLSTN2	LRRC4B	PPP2R2C
caki2	CST2	NMUR2	IGFN1	CYP4F3	SEC14L6	GOLGA6L2	CST4	TINAG	CST1	AKR1C1	CYP4F11	AKR1C2	CD70	NR0B1	CD163L1	MMP7	MEIOB	FABP6	CLDN16	XKR7	CCM2L	ZNF280A	AKR1B10	PANX2	PADI3	CA12	FIBCD1	HOXA4	AKR1C3	ASCL5	SYCE1L	ABCC2	METTL27	HNF1B	SLC12A8	VEPH1	EVA1A	PAX8	TRIM55	TRNP1	POU3F3	MNX1	TRIM16L	GALNT14	MSC	SPATA12	CA8	PPM1N	PYDC2	ZSCAN31
call	KRTAP12-4	TCL1B	KRTAP12-3	LCN6	HMHB1	TCL1A	VPREB1	DNTT	OR5P2	OR13A1	KRTAP4-12	PTH2	VPREB3	OVCH2	OR5P3	CMTM2	OR2B6	CD19	UGT3A2	HBB	BIRC7	LILRA2	TMEM236	IGLL1	NPY	HBD	CD79A	RAG1	TMPRSS15	SH2D4B	MZB1	CD79B	KCNN1	CD22	STAG3	PAX5	SYNE3	SSX7	SOWAHD	BANK1	HLA-DOA	GRAPL	SLC22A16	CCL17	HBA2	ALOX5	ERG	LAMB4	HBA1	GCSAM
calu6	FOXR1	CARMIL2	PRSS21	TMEM145	CLEC2L	VGF	OR4C6	CACNA2D4	HOXD11	SHISA3	RAB39A	MARCHF4	MAGEB2	IL13RA2	STX1A	XKR7	CACNG8	CSAG1	CCDC152	MAGEA12	FABP4	LYPD6B	HOXD13	EEF1A2	UNC13A	FOXL1	KCNQ2	DLX1	TM4SF18	WDR72	CDKN2A	GAL	DLX2	CD163L1	GNG4	ZNF787	CPLX1	KIAA1549L	CACNG6	CATSPER1	PRSS3	DRD2	PPP1R14A	RPP25	HOXA9	PHF19	TEAD4	CDK15	AMIGO2	PIF1
cama1	ANKRD30A	CDSN	ANKRD30B	HOXC13	CLEC3A	SPDEF	PYDC2	SYT12	RNF223	FABP7	MUCL1	KREMEN2	ZNF467	F12	LMTK3	SPTSSB	HOXC11	SOWAHA	ARHGEF35	TFF1	S100A14	IRX4	CA12	ALDH3B2	ADRA2C	KCNK15	IRX5	IRX3	RHOV	CARD14	ANXA9	EPPK1	ZG16B	PRLR	ARHGAP8	EFHD1	GATA3	YBX2	RARRES1	FIBCD1	MICA	FOXA1	BSPRY	FAM111B	ADIRF	TFAP2A	TENT5B	GALNT6	FGFR4	INHBB
caov3	SPINK6	SPINK13	GALR2	KLK5	KCNJ6	MT1A	MT1M	KRT81	PNPLA5	NKX2-8	TLX3	DIRAS3	RBP7	DEFB126	PRSS21	ODAD3	MT1G	NPB	DOK7	CRYBB2	SULT1A2	GPRIN2	PRSS22	TCEAL2	DMBX1	DKK1	CST6	PTGES	PRAME	EPHB6	FBXO27	NMU	SLC10A4	NPAS1	IL11	HMSD	ERVMER34-1	TRIM15	MESP1	C1QL1	ART5	TMEM249	MT1E	ALPP	KCNG1	BHMT2	RAC3	PROSER2	KLRG2	SLC15A3
capillary endothelial cell	SLC5A4	ZNF366	FCN3	SHANK3	DIPK2B	UPK3BL2	BTNL9	ERICH4	INSYN2A	SPAAR	CA4	PRX	HECW2	SH3RF3	BTNL8	GALNT18	IL1RL1	ADGRL2	PRSS48	HTR3C	CLEC1A	TNFRSF6B	CCDC194	SLC22A24	TAC4	SHOC1	VIPR1	CCDC85A	KHDRBS2	SLCO2A1	PRICKLE2	F2RL3	PKD1L1	ADGRF5	CDH5	ARSF	SPDYE1	ROBO4	FGD5	RAPGEF5	AFF3	LDB2	SLC22A25	CLDN5	SLC22A10	ANO2	PLA2G4C	GOLGA6L7	PLEKHG1	EMCN
cardiac mesenchymal cell	MYL7	WNT2	SFRP5	ASB4	TNNI1	GATA5	CFC1	MYL4	TBX5	TNNT2	POPDC2	ASB12	SPIN2A	MAB21L2	NKX2-5	PRRT4	LRRN4	HAND2	HAND1	GATA4	CSRP3	SMYD1	MYBPC3	TBX18	PRRX2	LEFTY2	SMTNL2	TBX20	PWWP3B	FGF8	UNC45B	HOXB1	PTGES3L	SLC38A4	ALX1	DUSP9	TNNC1	GATA6	MYL3	HAPLN1	BVES	PRDM6	GDF2	TECRL	BHLHA9	ZAR1	FOXF1	NTN3	ZBTB12	IGDCC3
cardiac mesenchyme	TCF21	EEF1AKMT4	TBX20	PDLIM3	NKAIN4	MFAP2	HAPLN1	HAND2	MSX1	TWIST1	GNG8	TNNI1	RBM46	CTHRC1	MFAP4	MSTN	PRRX2	FBXL7	BAMBI	PTN	PLAC1	KCNMB4	REM1	MOSMO	NRK	SCX	MYL7	TAGLN	SEM1	UPP2	CSRP2	RBP1	RAMP2	IGF1	GRB10	RTRAF	SELENOH	REX1BD	ERG28	PAM16	SYCE2	F2R	CTXN1	WDCP	GUCY1B1	SPINT4	DNLZ	DMAC1	GATA6	ALYREF
cardiac muscle cell	MYBPC3	MYOZ2	MYH6	TECRL	LRRC10	CSRP3	MYH7	PLN	TNNT2	MYL3	MYBPHL	HSPB7	MYL7	LMOD2	MYL2	TCAP	TNNI3K	TRDN	NPPA	CKMT2	FBXO40	HRC	SMYD1	LDB3	XIRP1	CKM	SBK2	SYNPO2L	POPDC2	FITM1	TNNC1	STYXL2	DHRS7C	MYL4	TRIM63	COX6A2	TBX20	SMPX	TNNI3	MYO18B	TNNI1	ITGB1BP2	MYLK3	NPPB	CASQ2	NRAP	KLHL31	TBX5	KCNG2	PGAM2
cardiac muscle myoblast	CER1	PRAMEF6	TBC1D28	TMEM269	PSKH2	OR2Z1	MYL3	AMY2A	LEFTY2	MAGEB17	MYH7	OR11H2	MYBPC3	WDR93	VRTN	PRR20C	TBX20	DEFB107B	KLHL4	APLNR	GYPE	EXOC1L	NPPA	ALPK2	TNNT2	HAPLN1	TNNI1	ZNF730	ITGB1BP2	CLEC20A	EGFLAM	ZIC3	CSRP3	HAS2	ABRA	MYLK3	LRRC10	IL1RAPL2	SMYD1	GCNT7	SLC15A5	FBN2	PLN	TNNC1	MYH6	COL13A1	SRRM5	MESP2	SHISAL2B	HSPB7
cardiac myoblast	TECRL	PLSCR5	HAND1	WFIKKN1	RGS13	SLN	SLC39A2	SYT10	MYL7	PITX2	FRZB	SPRR2F	HTR1E	LCN1	TNNI1	IGDCC3	MAB21L2	PITX3	ALOX15	HAPLN1	GCK	AKR1B15	TMEM235	RSPO2	TYRP1	SBSPON	MMP23B	LCN15	CTSV	DOK4	CA14	TMEM200B	HOXB5	RSPO4	BMP4	HOXB6	FOXH1	HAND2	SHISA3	LIX1	MFAP4	TNNT2	PRDM6	TMEM92	FOXF1	ODAM	TMEM98	GNRH2	AQP10	BAMBI
cardiac neuron	KCNA1	GFRA3	TMPRSS5	LGI4	KCNA6	SCN7A	GPR37L1	FOXD3	RASGEF1C	DHH	EGFL8	ASPA	INSC	CMTM5	KCNA2	NPPA	C4B	GFRA2	ART3	PRIMA1	TMEM88B	PLAC9	PLP1	PAQR6	MATN4	RXRG	FNDC1	ADAM11	SOSTDC1	SOX10	NKD2	AATK	LY6G6F	SHPK	APOD	MRGPRF	MPZ	NRN1	SLITRK6	SEMA3B	TMEM200C	LY6G6D	PLXNB3	FXYD1	CADM4	TAFA5	WNT6	PCDH20	ADAM23	SFRP5
cardioblast	VAX1	MIXL1	VRTN	ZSCAN10	HOXD13	EVX1	LEFTY2	FOXH1	EVX2	TDGF1	FGF3	SOX3	FOXF1	CYP26A1	MESP2	SLC34A2	CER1	NKX2-4	MOGAT2	FOXE3	APLNR	EOMES	SCN4B	FGF4	FGF8	FOXE1	CFC1	POU5F1	MESP1	GSC	NODAL	SALL4	PRSS41	GATA4	TEDC2	KCNK12	SP5	LHX1	KRT82	ASB4	DNMT3B	WNT3	OTX2	TBX6	TRIM71	SALL3	TCP11X2	TEX13B	HOXB1	MSGN1
cardiocyte	MYL2	NPPA	CSRP3	MYH6	MYH7	PLN	MYL3	TNNT2	HSPB7	MYBPC3	TNNI3	COX6A2	TNNI1	SMPX	SLN	TNNC1	MYBPHL	MYL7	CKM	MYOZ2	MYL4	APOBEC2	KCNE1	TRDN	POPDC2	FBXL22	SH3BGR	TRIM63	MYL1	ASB11	TCAP	CASQ2	ADPRHL1	NKX2-5	MYLK3	ANKRD1	HSPB2	FABP3	LDB3	MLIP	OBSCN	DES	TBX20	MYOM2	CITED1	ASB2	SOST	TMOD1	CMA1	CRYAB
caudal ganglionic eminence derived gabaergic cortical interneuron	OLIG3	STH	POU3F2	POU3F4	ZIM2	DCX	MLN	TMSB15A	DRAXIN	NEUROG2	GALP	XKR7	IGLON5	PABPC1L2A	GOLGA6L10	FNDC9	PHF21B	GNG8	GPC2	SIX3	BARHL2	RFPL2	CELF5	STMN4	TCEAL7	CCDC177	POU6F2	SIAH3	SHISAL1	DLX5	PAK5	TMEM151B	GDAP1L1	TMEM121B	DPYSL5	MAP6	TLCD3B	FAM131B	ZNF660	ATCAY	NCAN	PAX6	LRRN3	PDZD4	SEMA5B	MYT1	CCDC152	NEFM	SCRT2	LEMD1
cck81	IGFL2	PHGR1	CCNI2	INSL4	CDX2	TACR2	CARD18	CCNP	TNFSF15	PNLDC1	PIWIL1	SFTA2	RBBP8NL	UTS2	DEFB126	ONECUT3	ASCL2	DPEP1	PADI3	PLAAT2	CDX1	ABHD12B	YBX2	MAGEA4	S100P	C8G	VIL1	MT1G	LRATD1	LGALS4	MROH6	GPX2	AKR7A3	CASP5	TMEM52	TRIM15	MISP	PCSK9	IL20RA	CPNE7	USH1C	MUC13	GAST	KLHL35	TFF1	CCL15	FUT3	SLCO1B3	FUT6	HNF1A
cd1c-positive myeloid dendritic cell	USP17L30	FCGR2C	OR2A1	CLEC4A	CD1C	OR4M2	OR56B1	VHLL	GOLGA8T	CRIP3	GOLGA8O	LRRC37A	DAOA	MS4A4E	CD1E	GOLGA8K	MNDA	CMTM1	HLA-DOA	SPDYE8	LILRA1	HLA-DMB	CD33	TRIM34	CLIC2	OR2L8	IGSF6	CTCFL	PPIAL4F	PRR20A	ADGRE3	RNASE6	SSX7	CARD16	DUX4	LGALS2	GPR33	CD300C	RASA4B	NPIPA8	IFNL1	SIGLEC14	TBC1D3H	CLEC12B	CD1D	KRTAP5-4	CLEC10A	OR11A1	SULT1A3	FOXD4L5
cd4-positive helper t cell	IL3	IL9	IL31	IL17F	IL5	IL22	OR10AC1	IL13	IL17A	IL2	RTP5	CD40LG	OR4D1	CSF2	GPR15	OR56B1	LTA	TMEM272	TAS2R41	TNFRSF4	IL21	ICOS	TNFSF8	PDCD1	TRAT1	IL2RA	TIGIT	CCR4	OR52I2	CTLA4	FOXP3	CXCR5	KLRB1	SIRPG	ZNF80	KRT72	PPP1R2C	CCR6	OR2A25	CCR8	CHI3L2	ARMH2	CD3D	OR4E1	BTLA	CD5	CD6	TNFRSF25	CD2	SLAMF1
cd4-positive, alpha-beta cytotoxic t cell	GZMH	FGFBP2	ZNF683	FCRL6	LAIR2	KIR2DL1	KLRG1	GZMA	GZMM	CD40LG	CD3D	PATL2	SAMD3	CD2	PYHIN1	CST7	PTGDR	CD6	CD3G	KIF19	PROK2	PRF1	HCST	KLRB1	S1PR5	KIR3DL1	PLAAT4	CD3E	APOBEC3H	TBX21	KLRF1	MYO1F	THEMIS	SLFN12L	SDR42E2	KIR2DL3	GOLGA8Q	CTSW	GIMAP7	SIT1	IFNL1	HOPX	TRAF3IP3	TRAT1	GZMB	APOBEC3G	FLT3LG	SHISAL2A	CD226	CCL4
cd4-positive, alpha-beta memory t cell	OR4F5	H4C5	IZUMO1R	F8A2	PPP1R2B	PRAMEF14	H4C11	H3C4	CD40LG	H2BC8	H2BC4	AMY1B	NBPF4	RAMACL	TRAT1	KLRB1	CD6	ZC3H11B	CD5	ETV3L	IFNL4	CD3D	CCR4	CD3E	CD28	CD3G	OR4D1	IL7R	RGL4	KIR2DL1	LTB	KRTAP12-3	ICOS	GIMAP7	CD2	OR5J2	TNFSF8	ITK	NPIPA7	PYHIN1	OR52I2	SPDYE17	HSD3B2	P2RY8	SIRPG	NCR3	AMY1A	GZMK	CD96	OR3A1
cd4-positive, alpha-beta t cell	ERFL	FOXP3	IZUMO1R	ZNF831	THEMIS	GPR25	ICOS	SLFN12L	CD40LG	SIRPG	CTLA4	TRAT1	CD6	CD5	CD28	ITK	H2AC14	CD3G	CD3E	CD3D	NIBAN1	CCR7	CCR4	H2AC17	TNFRSF4	CD247	FLT3LG	INSYN2B	SIT1	H3C12	KRTAP2-4	IL7R	H2AC13	GPR174	GIMAP7	P2RY8	SKAP1	FDCSP	BCL11B	GRAP2	TNFRSF25	SLAMF1	CD2	TNFSF8	SH2D1A	LAT	IL2RA	SCML4	ZAP70	CD4
cd4-positive, cd25-positive, alpha-beta regulatory t cell	IZUMO1R	FOXP3	TNFRSF4	CTLA4	IL2RA	PMCH	CCR8	TIGIT	ICOS	ZNF80	CCR4	TNFRSF9	IL3	IL10	SIRPG	TNFRSF18	PDCD1	CD28	LTA	TRIM43B	CXCR6	CPA5	CD2	LAIR2	CD3D	CD5	LRAT	IL21	CD3G	CD6	SIT1	SLAMF1	CCR6	CD4	CD40LG	CD247	CD27	ZNF831	SH2D1A	CD3E	ZC3H12D	IL2RB	GPR55	TNFRSF25	P2RY10	IL22	LAT	LTB	BATF	IL17A
cd4-positive, cv25-positive, alpha-beta regulatory t cell	FOXP3	CTLA4	PMCH	TNFRSF4	IL2RA	SIRPG	TIGIT	ICOS	CXCR6	LAIR2	SIT1	LTA	TNFRSF18	CXCR3	APOBEC3H	KLHDC7B	GNG8	ETV7	TNFRSF9	RGL4	CD2	BATF	SLAMF1	CD27	CD3D	ZBTB32	CARD16	IL2RB	GBP5	LTB	NCR3	IL12RB1	CD3E	TNFRSF8	CD7	KRT86	P2RY10	IL10	SH2D2A	UTS2	CCR8	APOBEC3G	IL32	CEACAM21	BFSP2	LCK	PDCD1	DOK2	CD5	CCR5
cd4-positive, cytotoxic t cell	KRTAP5-1	GZMH	FGFBP2	GNLY	KIR3DL2	KIR2DL1	FASLG	FCRL6	P2RY8	RTP5	KLRG1	GZMM	WNT1	KIR2DL1	KIR2DL3	NKG7	PRF1	GZMB	OASL	S1PR5	CST7	SH2D1B	IFNL1	HNRNPCL1	GZMA	CD3E	TBX21	HTR6	APOBEC3G	FLT3LG	CCL5	KLRB1	CD6	IFNG	SAMD3	SLA2	LAIR2	SH2D2A	CD247	TNFSF14	APOBEC3H	CD40LG	IL7R	CD5	IL21R	ZNF683	SLFN12L	ZAP70	CTSW	UQCRHL
cd8-alpha-beta-positive, alpha-beta intraepithelial t cell	KLRC2	NCR2	TMIGD2	CD160	XCL2	FASLG	SIRPG	RGL4	KIR2DL2	XCL1	GZMA	GNLY	CD7	NCR3	CCL5	KLRC1	ZNF683	KLRB1	TIGIT	CXCR6	KLRF1	GZMB	CD3D	HOPX	FABP1	PLAAT4	PRF1	NKG7	MS4A12	CTSW	KLRD1	SH2D1B	ABI3	IL12RB	CD3E	PTPRCAP	KRT86	CD8B	DEFA5	GZMM	PHGR1	CD8A	P2RY11	IFNG	PTGER2	CD247	HCST	CD96	IL32	GIMAP7
cd8-positive, alpha-beta cytotoxic t cell	H2AB3	OR5K2	PPIAL4H	H2BC10	KLRC4	SEPTIN1	NIBAN1	H4C13	H2BC6	RESF1	KIR2DL1	GZMH	H4C6	FGFBP2	ZNF80	H2AC15	TMEM275	GNLY	H2BC15	SHFL	KLRF1	H3C1	KIR3DL2	PYHIN1	GZMB	PRF1	DTHD1	ZNF831	H3-3A	RTP5	KIR2DL3	H4C2	KIR3DL3	H1-3	CD8B2	CD8B	CD8A	CRYBG1	KLRG1	H2AC16	KLRD1	CST7	FASLG	OR52E5	XCL2	SEPTIN7	GZMK	H2AC4	LAIR2	S1PR5
cd8-positive, alpha-beta memory t cell	KRTAP19-4	F8A2	ZNF80	SMIM38	LCE2D	OR6S1	OR7C2	FCRL6	CD8A	CRTAM	GLP2R	CTLA4	RTP5	IZUMO1R	GZMK	CCL5	KIR2DS4	GZMH	IFNL1	KLRC4	OR2A25	CD8B	KRTAP12-3	SLFN12L	CD160	FCRL3	LRRC52	NLRC4	ZNF683	THEMIS	RIMBP3B	TBX21	XCL1	CD3G	SH2D1A	DEFA3	IFNG	GZMA	FGFBP2	OR13D1	NCR1	SAMD3	GOLGA8O	HEATR9	CD3D	OR2M7	KLRD1	DTHD1	CTSW	GPR55
cd8-positive, alpha-beta t cell	CD8B	GZMK	ERFL	SLFN12L	THEMIS	CD8A	KLRC4	OR4E1	ZNF683	ZNF831	CRTAM	C1QTNF8	GZMH	IFNG	GZMA	SAMD3	GZMM	KLRC3	SIRPG	CD3G	APOBEC3H	CD3E	CD3D	GPR174	DTHD1	SH2D1A	FASLG	PRF1	CD8B2	ZNF80	NKG7	ITK	CXCR6	OR4D1	CD6	PRF1	CD247	NIBAN1	KLRC1	GRAP2	PDCD1	CCL4L2	INSYN2B	KLRD1	GZMB	TRAT1	AOAH	OR5B2	TBX21	SKAP1	IKZF3
cd14-low, cd16-positive monocyte	LIPN	LILRA1	LILRB2	CD300E	LILRA2	CASP5	LILRB1	FCGR3A	LILRA5	FCN1	GPBAR1	SIGLEC10	APOBEC3A	LILRA6	ADGRE3	ADGRE2	OR4E1	LRRC25	MS4A7	HCAR3	MS4A14	CCL4L2	CD300C	CEACAM3	TNFRSF8	OSCAR	PILRA	BCL2A1	TLR8	OR52K2	SIGLEC9	IFNB1	PRAM1	CALHM6	SIRPB1	FGF23	C5AR1	CYP4F22	MS4A4A	CLEC7A	SCIMP	AQP9	OR52K1	NLRP3	TNFSF13	HK3	FGR	MEFV	LILRB3	LST1
cd14-positive monocyte	S100A12	FCN1	CD300E	LILRA2	LILRA1	OSCAR	LILRB2	FOLR3	LIPN	RNASE2	SIRPB1	OR52K2	FCAR	LILRA5	LILRA6	APOBEC3A	FPR1	SIGLEC9	SIGLEC14	MCEMP1	TLR8	SIGLEC7	FCGR1A	SIRPB2	MS4A6A	MNDA	TREM1	LILRB1	IL27	CCL4L2	GPBAR1	CD1D	PRAM1	RETN	CLEC4A	CEACAM4	NLRP12	NLRP3	AQP9	S100A8	HCAR3	MEFV	CLEC7A	OR4E1	VNN2	FCGR2A	CD300C	LILRB3	SLC11A1	CLEC4E
cd14-positive, cd16-low monocyte	RFPL4A1	PRRG3	LILRB5	HSPA6	CLEC10A	MARCO	CD300E	CD1C	HLA-DQA2	LILRB2	MS4A6A	HLA-DRB5	FPR3	HLA-DQA1	CHRFAM7A	SIGLEC7	FCER1A	LILRB1	HLA-DQB1	MS4A4E	MS4A14	FCGR2A	CD300LB	SIRPB2	TNFRSF10C	LIPN	FCGR2B	SIGLEC9	SDS	F13A1	NAIP	HLA-DQB2	LYZ	HLA-DOA	HCAR3	HLA-DPA1	HLA-DRB1	ADGRE2	LILRA2	STAB1	HLA-DRA	SLAMF8	CEACAM4	CD1E	CD163	HLA-DPB1	CD300C	MS4A7	HLA-DMA	
cd14-positive, cd16-positive monocyte	LILRA1	OR4E1	LILRB2	FCN1	CD300E	FGF23	GPBAR1	LILRA5	FCGR3A	LILRA6	CALHM6	LILRB1	OR10G4	OR5A2	LIPN	OR52K2	APOBEC3A	ADGRE2	SIGLEC10	OR52K1	OSCAR	SIRPB1	MS4A7	ADGRE3	OR8B8	LRRC25	LILRA6	CASP5	SIGLEC9	CD300C	TLR8	OR2V2	OR4K13	OR1M1	SIGLEC7	ADA2	TNFRSF8	OR5AU1	PILRA	OR2V1	PRAM1	SECTM1	OR10H5	FPR1	OR5H14	TNFSF13B	SCIMP	MS4A4A	CLEC7A	FCGR2A
cd16-negative, cd56-bright natural killer cell, human	KIR2DL4	XCL2	KLRF1	KLRC3	SH2D1B	IL2RB	GZMK	KRTAP29-1	PTGDR	IL2RB	KLRD1	IFNL1	CTSW	OR10A3	CRTAM	FASLG	KLRB1	NCR3	PRF1	CD7	OR10H1	IL12RB2	LGALS9B	IL18RAP	NKG7	OR2Z1	OR10H3	NCR1	OR2M5	ITGAX	KIR2DL3	SIGLEC7	TNFSF14	KLRC4	SAMD3	SPTSSB	MATK	KLRC2	TXK	HSH2D	CHRFAM7A	KIR2DL1	CXCR3	GZMB	MAS1L	OR1L3	TMIGD2	APOBEC3G		
cd16-positive, cd56-dim natural killer cell, human	KIR2DL1	KIR3DL1	KIR3DL3	KIR2DL3	KLRF1	OR52L1	PRAMEF15	SH2D1B	OR5M3	KIR3DL2	OR10V1	OR52E5	OR1K1	FGFBP2	FCRL6	OR2M2	CXCR1	KIR2DL4	FCGR3A	OR4F4	OR2AE1	PTGDR	OR2L8	OR1L6	OR8U1	PRF1	OR5K2	NMUR1	GNLY	S1PR5	SIGLEC7	FCRL3	GZMH	GZMB	CD160	NCR3	OR5B2	KLRC4	OR52N5	KLRD1	LIM2	OR8H2	OR1L4	OR1L8	GOLGA8O	HEATR9	KLRB1	PRAMEF18	OR11H12	GZMM
cd141-positive myeloid dendritic cell	RFPL4A1	CENPVL1	CLEC9A	FCGR2C	CD1C	GPR31	IDO1	HLA-DQB2	CLEC4C	CD207	HLA-DOB	IDO2	OR52K2	HLA-DQB1	HLA-DMB	CD1D	HLA-DQA2	OR1J1	TRIM34	HLA-DQA1	OR3A1	CLEC10A	RFPL4A	MS4A6A	CPVL	CLEC4A	TLR10	GOLGA8K	TBC1D3F	GCSAM	DNASE1L3	F8A3	OR56B1	FCER1A	GPR33	CRIP3	LGALS2	SIGLEC16	POTEM	IGFLR1	CLEC7A	RIMBP3B	SIGLEC7	BTLA	SLAMF8	CLNK	HLA-DMA	OR11A1	SIGLEC10	FLT3	CHRFAM7A
cell	AVP	OTP	RTL1	MAGEL2	GPR139	SGCZ	NKX2-4	VWC2L	GRIN3A	AGRP	LRRTM4	SLC6A7	USP29	CALCR	PDYN	GABRG3	GABRG1	GHRH	FAM135B	KLHL1	OXT	ASIC2	SORCS3	NCAN	SLC5A7	PLPPR5	TRPC7	GALNTL6	DSCAM	GRID2	HS3ST4	IL1RAPL1	PCDH15	NXPH1	NWD2	HTR4	MDGA2	BRINP3	HCRTR2	CSMD1	GRM5	DCC	NRG3	IL1RAPL2	SLC6A11	CSMD3	LHFPL3	KRTAP17-1	LRRC4C	VWA5B1
cell of skeletal muscle	STYXL2	MYH4	MYH8	MYMK	TMOD4	MYH1	LRRC30	ATP2A1	TNNT3	TNNC2	MYBPC2	MYOZ1	CKM	METTL21C	MYH3	PRR33	MYL1	MYOT	ATP1B4	TNNI2	MYF6	DHRS7C	KLHL41	CACNG1	ASB5	LBX1	MYBPC1	XIRP2	TCAP	AMPD1	MYF5	TRDN	MYLK2	NEB	HJV	PVALB	KLHL31	PRR32	ABRA	FITM1	MYOD1	CASQ1	MYBPH	MYLK4	VGLL2	CRYAA	PPP1R27	MYOM3	LDB3	MYOG
central memory cd4-positive, alpha-beta t cell	TSPY1	FLT3LG	CCR7	TRAT1	CD5	CD40LG	GIMAP7	CD6	CD28	CD3E	GCSAM	LTA	P2RY8	SIRPG	IL7R	ITK	VHLL	OR10H1	TCAF2	TESPA1	LTB	OR7A17	GIMAP2	FCMR	RGL4	CD7	ICOS	TNFRSF25	CD3D	OR10K1	OR1A1	CEACAM21	CD247	OR2A25	CCR4	P2RY10	UQCRHL	ZAP70	SCML4	TAGAP	LIME1	CD28	OR2AP1	ANXA2R	CD3G	OR4F17	GPR25	OR5M1	OR2L2	CAMK4	CD2
central memory cd8-positive, alpha-beta t cell	CD8B	CD8A	KLRC4	FLT3LG	OR10J4	CCR7	ZNF683	SIRPG	CD3E	CPA5	CD6	KLRC3	GZMK	OR7A17	OR10H1	GZMM	PVRIG	CD7	RGL4	CD5	CRTAM	GIMAP7	ZNF80	THEMIS	TRAT1	P2RY8	GOLGA8F	CD3D	IL7R	LTA	GCSAM	ITK	KLRK1	CD2	SCML4	SLFN12L	CD96	OR1A1	CXCR3	GPR15	CD3G	ZAP70	OR2L2	GIMAP2	TNFSF8	P2RY10	CCL5	OR4F17	OR11G2	CEACAM21
central nervous system macrophage	PNMA6F	P2RY12	CRYBB1	GPR34	TREM2	SLC22A10	PNMA8C	TGM6	P2RY13	CRYBA4	CX3CR1	FGF20	TMEM119	ADORA3	C1QC	SLC2A5	CCR5	ADGRE1	GPR84	KCNK13	LTC4S	C1QB	C1QA	SMIM10L2B	CSF1R	KBTBD12	IL1A	LY86	CD180	HPGDS	MYMK	PLD4	LAG3	DSG4	VSIR	SLCO2B1	C3AR1	P2RY6	OLFML3	TLR7	LPCAT2	CD33	PPFIA4	TIFAB	ABI3	ZNF311	CLEC5A	LAIR1	FGD2	TNFAIP8L2
central nervous system neuron	NBPF6	TMEM272	KRTAP9-2	HTR2C	HTR4	ZNF735	HS3ST4	NMBR	GPR149	RAB41	PRAMEF6	HTR1E	CRACDL	PRB2	FOXD4L6	SLC6A5	GRM8	SCRT2	CACNA1E	ADGRA1	GBX1	PDZD9	LRRC7	RYR3	CDH9	CDH18	ANO3	TMEM132D	SLC35F3	FAM135B	GPR158	SNTG1	GRM5	AGBL4	RTL4	SLC22A10	MDFIC2	TBR1	GRM7	MPPED1	GRM3	DGKB	LRRTM3	KCNQ5	GRM1	HTR1F	BRINP3	BRINP1	STXBP5L	CACNA1B
cerebellar neuron	OR2L13	HTR1E	GPR149	GPR139	LPA	ZNF804B	GRIK4	SEPTIN3	GRM3	CNTNAP5	RFPL2	DMBX1	SSTR1	RAD21L1	MAGEC3	SLC24A4	ZPLD1	MINAR1	OVCH1	GRM7	ANKRD30BL	KLHL1	TMEM200C	TRPC7	CSMD2	DCHS2	GRIK3	CDH8	TMEM271	GRIN3A	NXF5	SLC9C2	FSTL5	LRRTM4	KCNIP1	SV2C	SLC44A5	RIT2	CHRM2	SAMD5	LRRTM3	CHRM3	THSD7B	ADARB2	RNF175	PNMA6F	CSMD3	INSYN2B	LUZP2	SHISAL1
cerebral cortex endothelial cell	BARHL2	TLX3	NPAS4	SHISA8	TMEM252	SLC19A3	NEUROD2	ADAMTS18	CCM2L	CDHR4	CBLN1	KCNK10	NTF3	NHLH2	LRRC75B	OLFM3	BHLHE22	ZIC4	NOS1	DIRAS2	LGI1	FAT2	BCL2L15	SOX17	GRM1	TRIM46	SCUBE1	KANK4	KCNH1	TEKT2	CCDC73	CNTNAP1	ZIC1	PLPPR4	PTCHD4	SLITRK5	SHE	KIF26A	NEUROD1	CLDN5	KCNK2	CASP12	IGSF21	SYNPR	PNPLA3	USHBP1	SP5	UNC5D	GJA4	PDZD4
cerebral cortex gabaergic interneuron	NMS	ANKRD63	LBHD2	PNMA8B	CTXN2	PHF24	CBARP	PLPPR4	SLC32A1	FAM163B	RPRML	GPR26	STUM	KCNJ9	IL25	NRSN1	KCNJ4	ADGRB1	NTSR2	TMEM130	ANKRD34B	TTC9B	C1QL3	MOBP	GAD2	SLC6A11	ADGRA1	CACNG2	RPH3A	PRKCG	DDN	TMEM179	MPPED1	SYN2	ZFP92	HPCAL4	SCRT1	CALY	NSG2	RESP18	PNMA3	GRIA2	GRIN1	ZCCHC12	CACNA1I	FRRS1L	SCN2A	CELF6	SHISA7	CACNG3
cerebral cortex neuron	GPR52	SERPINA9	KCNV1	GPR88	GPR6	CHRM1	FAM163B	OPRK1	HRH3	CACNG3	GABRD	C1QL2	DCLK3	PDYN	SYNDIG1L	KRT9	KCNS2	KCNA4	LINGO3	MCHR1	SHISA7	CHRM4	RPRML	DDN	GPR83	GABRA4	HPCAL4	KCTD4	HPCA	CACNA1E	ICAM5	KCNT1	SSTR4	KCNH4	CAMKV	RGS8	PHYHIP	GSG1L	CDH9	PTPN5	NEUROD6	CPNE6	KCNIP2	RASD2	HTR6	ANO3	GPR12	SYNPR	RASL10A	PDE1B
chla10	CENPVL3	CYP4F22	STH	PRAC2	GDF6	HOXC12	GRP	CALCB	PRAC1	SLCO5A1	RBM11	MBD3L3	TPTE2	FEZF1	NPW	VAX1	MBD3L2B	TMEM229A	NKX6-1	POU3F1	DLX4	ADRB1	PHOSPHO1	ZNF723	TMEM250	SLC18A3	APCDD1	BARHL2	SAMD11	ADRB3	ARTN	PCDH8	PRRT4	NKX2-4	TTLL6	PGLYRP2	TFDP3	PTGER3	MEIOB	PKP1	TEDC2	GYG2	HAND2	ZNF730	HOXC11	RNF175	MAFA	PLPPR3	ZIC2	INKA1
chol	CYP2C8	UGT2B15	CEACAM7	CPB2	APCS	SUCNR1	PLGLB2	C8G	C4BPA	IGFBP1	FGA	CA9	ORM2	HABP2	ITIH2	UGT2A3	CRP	ORM1	INHBE	ALB	AMBP	FGL1	CYP2C9	AKR1C4	TM4SF4	CHRDL2	KRTAP1-1	AADAC	ASGR2	VNN2	FGG	CYP2C18	AGT	SAP25	SERPINA6	UGT2B17	SCTR	CLRN3	DEFB1	SLC17A4	CXCL6	PKHD1	KCNK9	VTN	CCL20	ANXA13	MBOAT4	CREB3L3	PAEP	UGT2B4
cholangiocyte	UGT2B28	OR2AP1	UGT2B15	SERPINA4	TM4SF4	CHST4	ALB	FGF19	OR10K1	OR2B11	BAAT	APCS	AMBP	TBC1D3K	CYP3A7	SPINK9	CYP2C8	OR52N1	SLC17A4	TBC1D3E	SERPINA5	CENPVL2	OR6C75	UGT2A3	OR7A17	FCN2	HABP2	ALKAL1	CXCL6	CPB2	ANXA13	TFF2	DUOX2	DUOXA2	NR0B2	PAH	SERPINA6	UGT2B10	LCE6A	FGA	CYP4A11	ORM1	ABCB11	OR51M1	OR10H1	GVQW3	CA9	ORM2	AHSG	G6PC1
cholinergic neuron	CHAT	ALK	CPO	CORIN	DRGX	OTP	HRG	GRP	CRP	RAB26	EYS	MIP	RBFOX1	CLC	NYAP2	HEPH	SYN1	LRP4	DAO	UNC79	ART1	DCC	CADPS	SYT7	DYRK3	SYT4	SIAH3	LHX4	NAT1	PROC	SPX	NWD2	PDE1C	PDP1	CLVS1	IQCJ	NRK	GLRA1	HASPIN	CTNNA2	OXT	WDR62	UTS2B	SLC18A3	MID1	DDC	OCM	SCHIP1	SYT1	MED26
chondroblast	HOXD12	AGTR2	RTL3	MATN1	OTOR	ANGPTL5	UBE2NL	TBX5	KLF14	EPYC	COL11A2	COL9A1	CAPN6	TBX15	SHOX2	MEOX2	MATN4	NTN3	COMP	GAS2	TNMD	APCDD1L	BARX1	FIBIN	KERA	TBX18	COL2A1	TMEM26	MATN3	KRTAP21-2	GDF5	CYTL1	LPAR4	DKK2	SUSD5	ANGPTL1	ASB4	NRK	P4HA3	DLK1	CHI3L2	COL11A1	EGFL6	LHFPL1	BDKRB1	HOXD13	SCX	MAB21L2	FAM180A	CXCL5
chondrocyte	CHAD	UCMA	CLEC3A	EPYC	PANX3	COMP	MATN3	MATN1	COL11A2	COL9A1	CYTL1	SUSD5	RTL3	OTOR	COL2A1	FZD9	CNMD	HAPLN1	FAM180A	COL11A1	PRR33	WIF1	GDF5	NCMAP	COL9A2	FMOD	CILP2	IBSP	COL9A3	PRG4	HOXD12	FIBIN	SCRG1	OMD	CHADL	MATN4	MYOC	MIA	PLA2G5	KCTD4	ITGA10	COL10A1	CRISPLD1	LOXL4	CAPN6	COL8A2	MMP13	SP7	R3HDML	GDF10
choroid plexus epithelial cell	PRR32	WFIKKN2	HTR2C	KCNE2	IL25	KCNJ13	SLCO1C1	OR4P4	LIM2	SLC39A12	CFAP126	SLC13A4	SLC16A8	ATP2B3	VAT1L	PMCH	SLC7A10	CLIC6	SLC2A12	OPALIN	SLC22A8	SOSTDC1	OTX2	GMNC	COL8A2	MOBP	TMEM72	PLPPR4	LMX1A	ZIC4	SLC4A5	KLK4	TTR	DYNLT4	RD3	DRC7	DCAF12L1	CLDN2	AIFM3	ABCA4	PIFO	TRPM3	SLC6A15	CALML4	FOLR1	DYDC2	GDF5	ZIC1	SLC4A10	VWA5B1
chromaffin cell	DBH	MLN	PHOX2B	PVALEF	ASIC5	ARSF	CYP21A2	INSM2	PHOX2A	ADGRG4	SLC6A2	SLC22A10	CYP17A1	ONECUT3	FAM163A	RFX6	SLC18A1	APOL5	RIMBP2	CHRNA3	HAND2	PNMA6F	EYS	MARCHF11	SULT2A1	PAX4	FEV	SEPTIN3	ZNF804A	SLC38A11	TMEM132C	PRAMEF6	CHRNA7	LRRC53	PRRT4	STAR	BTNL8	HMX1	TMEM132D	MARCHF8	MGAM2	CDIN1	KCNB2	KCNQ5	GIPR	TPH1	KCNK17	MC2R	BNIP5	SSTR5
ciliated cell	WFDC6	FAM216B	LDLRAD1	GAS2L2	SNTN	ZNF474	ANKRD66	APOBEC4	TSPAN19	SCGB1D4	CFAP73	TUBA4B	DNAI2	STOML3	DYNLT4	MAP3K19	DYDC2	SRD5A2	AKAP14	TMEM190	STMND1	CATSPERD	RSPH4A	CIBAR2	CCDC78	FRMPD2	CDHR4	DNAH3	WDR38	PLEKHG7	PRR29	CD200R1L	DEFB124	PIFO	CFAP97D2	OR1L8	FAM81B	CCDC17	ECT2L	MORN5	CFAP157	FAM166B	DTHD1	EPPIN	DRC1	LRRC71	CFAP57	CFAP52	DNAH12	CCDC33
ciliated cell of the bronchus	HOATZ	DNAAF6	MARCHF10	FAM166C	CFAP91	NPIPA9	RSPH10B2	ANKRD66	CCDC190	GSTA5	CRACDL	OR6C76	NPIPB9	NPIPB7	ECRG4	NPIPA8	NPIPB12	NPIPA3	DNAI1	LGALS9B	EPPIN	TUBB8B	CIBAR2	MUCL1	H2AC18	NPIPA7	AMY1B	TMEM190	HSFY1	PLEKHG7	TOGARAM2	CACNG6	TSPAN19	PRR29	CFAP299	WDR38	DEFB124	FAM86B2	CCDC170	STOML3	FAM216B	LRRC74B	HHLA2	NWD1	LGALS9C	OR9Q1	NPIPB6	LRRC46	EFHB	ZBBX
ciliated columnar cell of tracheobronchial tree	SEC14L3	GAS2L2	DYNLT4	LDLRAD1	CCDC153	TNN	TEKT4	TMEM212	NHLRC4	SNTN	PRR18	FCAMR	TM6SF2	FAM47E	KLHL38	TCTE1	RSPH4A	CCDC154	CYP2S1	UBXN10	MFSD6L	CLDN18	LRRC10B	CCDC78	CIBAR2	FAM166B	ENO4	RIIAD1	KCNRG	CCDC33	TEKT1	AK7	PPP1R36	CCDC103	DYNLRB2	TTC29	SCGB1A1	AKAP14	NME9	CDKL4	LRRC43	DNAJB13	AK8	KCNJ13	NEK5	MAPK15	RNF186	TEKT2	SPAG6	RD3
ciliated epithelial cell	OR2T33	MARCHF10	SPATA31A7	ZNF474	OR2T11	OR8H1	OR5AU1	CFAP157	CFAP299	IFNL2	SNTN	FAM216B	MROH9	CFAP73	OR2AG1	CROCC2	DNAH3	CCDC190	OR10H5	CFAP97D2	FAM166C	OR4F15	DYNLT4	TMEM78	DNAH12	HOATZ	KIAA2012	OR2V2	OR2V1	LDLRAD1	OR1D2	WFDC6	ADGB	DTHD1	CDHR4	LMNTD1	ECT2L	MAP3K19	OR7D4	DNAH9	FRMPD2	CFAP99	OR10G4	DNAI2	DNAH10	CFAP91	OR5A2	TSPAN19	OR7G2	PLAAT2
class switched memory b cell	DEFA1B	DEFA1	PRR20E	OR10C1	F8A2	DEFA3	OR10P1	OR6N2	OR2T3	OR52E2	OR9A2	OR6C68	LCN10	KRTAP5-3	OR11H4	OR6C74	FCER2	MS4A1	OR2T2	PRAMEF5	OR7A10	BLK	OR10J5	CLEC17A	PRAMEF4	OR8H3	JCHAIN	OR6C3	CLEC20A	HLA-DOB	FCRL5	CALHM6	OR4D6	OR4K14	OR5AC2	KRTAP2-2	RIMBP3B	PRY	NBPF6	NUTM2F	FCRL1	POU2AF1	TLR10	BANK1	NUGGC	KRTAP1-3	SPATA31A6	OR4K15	TAS2R39	FCRL2
classical monocyte	OR52K2	S100A12	FCN1	CD300E	SCYGR2	TLR8	PEAK3	OR52K1	FCAR	FOLR3	LILRB3	SIRPB2	LILRA6	LIPN	CD1D	SIRPB1	LILRA1	FPR1	CLEC7A	MS4A6A	MNDA	LRRC25	OR52I2	LILRA5	RNASE2	MEFV	APOC2	OSCAR	CLEC4E	TREM1	GPR141	S100A8	LILRB2	SLC11A1	LILRA2	FGR	CCR2	PRAM1	CD300LB	CEACAM4	OSM	ADGRE2	SIGLEC9	LILRB4	TAS2R41	NLRP3	CSF3R	APOBEC3A	CLEC4A	VNN2

**cll**	FCER2	CLNK	FCRL5	TCL1A	TNFRSF13C	GDF7	CLEC17A	LILRA4	PAX5	ABCA6	WNT3	FCRLA	HLA-DOB	FCRL2	SCIMP	BLK	FCMR	OR2A25	RUBCNL	KLK4	SOWAHD	ARMH1	PXDNL	MS4A4E	HLA-DQA1	HLA-DQB1	MS4A1	CD79A	RESF1	ADTRP	CLEC20A	HLA-DMB	BTLA	HLA-DOA	KLK2	CCR7	ADAM28	GJB7	PNOC	CD19	CLEC4C	CNR2	ZNF107	CD79B	HLA-DRA	ZBTB32	P2RY10	HLA-DPA1	HLA-DMA	IKZF3
**club cell**	ROS1	ERFL	SFTPB	ATP13A4	MS4A15	SLC22A31	SFTPA2	SCGB3A2	DEFB131B	SFTPA1	SLC9C1	WFDC10B	AGBL1	SCGB1A1	SCGB3A1	BPIFB1	TTC6	ARHGEF38	SMIM35	CATSPERB	GPRC5D	SERPINB3	CAPN8	CYP2F1	INSYN2A	SLC34A2	PEBP4	CYP2B6	ANKUB1	C4BPA	SLC22A3	CXCL17	CAPN13	SHOC1	MSMB	GPR39	MUC4	CCDC194	BPIFA2	SLC6A14	SLURP2	SCNN1B	NUTM2G	LMO3	BPIFA1	CYP2A6	CLIC6	DUOXA1	OVCH2	DUOX1
**coad**	SEMG1	MUC17	CEACAM5	MUC3A	TRIM31	REG4	PRAP1	PRAC1	PHGR1	NOX1	TRIM15	FOXD4L5	KRT20	MOGAT3	CDH17	CLRN3	BTNL3	FUT3	TMEM238L	TFF3	PPP1R14D	MGAM2	LGALS4	AKR7A3	FABP1	AQP12B	SPINK4	CYP2D7	SMIM31	FAM3D	EPS8L3	UNC93A	FRMD1	NXPE1	CYP4F2	CEACAM6	MUC2	MS4A12	NPSR1	CEACAM7	MUC12	FUT6	SULT1C3	REG3A	RBBP8NL	CTAGE15	REG1A	BTNL8	ERN2	ARL14
**coad-adenoma**	TAAR8	DEFA5	MUC2	OR51G2	OR13C2	DEFA6	IGLL5	CLCA1	NXPE1	OR11H1	OR5B12	KRTAP5-5	OR6B2	MS4A12	OR8K3	OR12D3	OR1J2	CEACAM5	IFNA21	SPINK4	FRMD1	CEACAM7	OR4K1	FRG2	OR10J5	OR4C45	MAB21L3	OR5F1	RNASE8	OR10G7	B3GNT6	BTNL3	CYP2D7	OR1E1	ULBP3	OLFM4	RETNLB	OR2V1	UGT2B17	LYPD8	OR5M1	ZG16	OR6K2	OR4C11	DUOX2	FCGBP	OR4C13	HTR3D	NOX1	MUC3A
**colo680n**	CALHM3	KHDC1L	KRTAP4-1	ALPP	SYT8	ARL14	KRT37	IRX4	PADI3	CST6	KLK6	ERP27	PRAC2	SLC1A6	LY6K	TESMIN	PRSS21	KRT13	PRSS22	TMEM52B	ALPG	NT5DC4	NKX2-8	GAL	ZNF114	P2RY2	APOBEC3B	MALL	LY6D	PPP1R1C	RHOD	SMIM10	ZNF488	TMEM40	CATSPER1	KIAA1755	MYEOV	RNF223	NKX2-5	PSCA	FGFBP1	FOXE1	LHX5	RNF224	LCE5A	CAPN12	KLK5	ANKRD65	DMKN	RGS20
**colon epithelial cell**	URAD	CYP2W1	LCN15	ARSL	ISX	SIGLEC11	H2BC9	CTXND1	PGAP4	IHH	CHST13	SLC39A5	APOA1	SRY	MEP1A	FABP1	CDH17	H2AC8	SLC17A4	H4C11	PLA2G12B	H1-1	AGR3	H1-0	PRAC1	SLC13A2	MTARC1	LGALS14	H3C10	H3C7	H3-3A	GASK1B	MACIR	UPK1A	FOXD2	H2BC21	PABIR1	H2BC8	IFTAP	H2AC6	CLRN3	CDX1	CDX2	SLC19A3	LGALS2	INAVA	PNMA6F	MT1H	PRXL2B	H4C3
**common dendritic progenitor**	LILRA4	CLEC4C	TLR9	TBC1D3F	RHEX	PEAK3	GAPT	TTC24	LITAFD	LILRB4	IRF4	RNASE3	NIBAN3	PTCRA	H2AC16	CXCR3	H4C1	TIFAB	LCNL1	ASIP	SPIB	SIGLEC14	ADGRG5	SIRPB1	TLR7	SCT	TMIGD2	SEPTIN1	RNASE6	MPEG1	TASL	RNASE2	H3C11	LILRB1	FLT3	KCNK17	PPP1R2B	NOTCH4	MZB1	TCL1A	AZU1	SPNS3	DRD4	CMKLR1	SMPD3	ARRDC5	DEFA3	GJA9	IRF8	SHD
**common lymphoid progenitor**	IFNA13	DNTT	MPO	IGLL1	MC5R	FUT7	MYL10	FLT3	VPREB1	IL12A	BPIFC	NIBAN3	TREML2	MPL	LILRA4	NFE2	F2RL3	RGS18	PRTN3	SLC22A3	PRSS57	OTOS	CD34	LMO2	ARL11	SH2D5	LYL1	GAPT	GPR171	SPNS3	CTSG	CLEC4C	MYB	TICAM2	TAS1R1	SHISA8	DAPP1	TSPAN32	ADGRG3	ANGPT1	PGLYRP2	NKX2-3	HMHB1	BTK	ERG	CD27	P2RY14	UMODL1	SPINK2	RIPK3
**common myeloid progenitor**	OR2AK2	SPDYE8	LGALS9C	LRRC70	OR2T8	GOLGA8S	OR2L8	GOLGA8T	GLYATL1B	MPL	MPO	OR2T33	OR2A4	RNASE3	IL17REL	MLNR	DYTN	RNASE2	CRHBP	SPDYE6	TAS2R43	OR52M1	TBC1D3H	OR2L5	TBC1D3G	PNMA6E	OR2W3	CEACAM4	TAS2R7	TAS2R3	HTR1F	NUDT4B	NPIPB3	KRTAP12-2	TENT5D	MS4A3	DUXA	PRSS57	FCER1A	TPRX1	SPRR5	AZU1	SLC22A16	GOLGA6C	SPIB	GPR148	ELANE	TRIM74	CCDC27	CTSG
**cone retinal bipolar cell**	GSG1L2	BHLHE23	OTOR	CPLX3	GABRR2	GABRR1	LRIT1	RD3L	LRIT2	NRN1L	PLK5	LACTBL1	CABP5	NEUROD4	GRM6	RHO	GPR179	GNGT1	NYX	CPLX4	CABP2	LRTM1	TMEM215	KCNG4	IRX6	PCP2	GLRA1	GNG13	LHX4	GNAT1	PRPH2	RBP3	DRD1	RD3	VSX2	MGARP	CHRNA6	KCNA5	GNAT2	SNCB	SAG	PDE6G	GABRR3	GNB3	PDC	VSX1	CCDC177	KCNJ9	SLC17A7	OPN1MW
**connective tissue cell**	CCN4	CALHM5	AADACL2	GASK1A	PRL	OR52N2	APOD	OR52A1	OR4K17	EBF2	CCL11	COL6A5	INMT	ATP6V1FNB	ANGPTL5	OR6A2	TBX4	LVRN	AGTR1	LAMC3	XPNPEP2	FOXF2	HPSE2	PABPC5	ADH4	NKX6-1	EBF3	SCUBE1	GASK1B	FAM180B	OR52N4	ZNF676	RXFP2	EPHA3	AFF2	VIT	DCN	ALKAL2	MYOC	ALX3	PRIMA1	ITIH2	TBX5	REELD1	TCF21	OGN	PXDNL	FSHB	GJC2	COL13A1
**contractile cell**	CCL21	LGI4	MT1A	MT1M	C1GALT1C1L	SLC35G5	EGFL6	CIB4	CCL8	SGCA	ANGPTL5	CFHR1	ADAMTS4	CCL2	SLN	FAM162B	HIGD1B	C1R	GRIN2C	A2M	CCL19	MED14OS	LCN6	PLAC9	MPZ	COX4I2	NNMT	TRPV3	MRGPRF	MYOT	COL28A1	ADRA2C	PRIMA1	KIAA1755	HRH2	RGS16	FHL5	RERGL	TAGLN	GFRA3	COLEC11	CA11	CFH	FGF7	TMEM132E	COX7A1	CH25H	TCF21	ADIRF	CCL14
**conventional dendritic cell**	GPR31	CD1C	LIPN	HLA-DQB2	IFNL1	CD300C	FCER1A	HLA-DQA2	CD1D	CLEC4A	CLEC10A	SIGLEC9	LILRA1	IFNL4	HLA-DQA1	LILRA2	LILRB2	CCL22	CD1E	HLA-DOA	FCN1	HLA-DRB5	LILRA6	CLEC17A	MS4A4E	LGALS2	HLA-DQB1	IDO2	NLRP3	CHRFAM7A	OSCAR	BCL2A1	SIGLEC7	LYZ	IL1B	CD86	CD300LB	SPI1	CLEC9A	MS4A6A	HLA-DPA1	CEACAM4	TNFSF13	CFP	ZNF366	GPAT3	OR10K2	HLA-DMB	RNASE2	HLA-DPB1
**corneal epithelial cell**	MUC22	CRYAA	TRIM42	CRYGA	KRT12	APOBEC3A	CRYBA1	KRT4	CRYBA4	DSG1	KRT13	CRACDL	MUC16	TMEM244	CRYGD	RAX2	CRYBB2	CEACAM6	CRYGC	CRYGS	GGT6	SPINK9	MUC4	CRYBB3	ZNF727	GBA3	LYPD2	TACR3	MACIR	ADGRF1	VTCN1	CEACAM7	ZNF454	GRID2IP	SERPINB2	PLA2G4F	PP2D1	SLC34A2	SCEL	CA3	HS3ST6	GABRP	ZNF674	MYH14	TEX22	ZNF385C	XKRX	CALML5	DNAH17	BNIPL
**cortical cell of adrenal gland**	CYP21A2	MC2R	CYP11B1	CCN3	CYP17A1	SULT2A1	NR5A1	CYP11B2	STAR	LPA	GML	GPRC6A	SLC2A14	GNRHR	MAP3K15	PNMA6F	SMIM10L2B	GDPD4	SIGLEC11	ZNF541	C2CD6	CYP11A1	EZHIP	FAM166B	GASK1B	CLEC4M	ADAMTS19	AMDHD1	NRK	CNTN5	CFAP20DC	ZNF98	DPP10	ARHGAP36	CHRNA7	FREM2	CACNB2	SPINK13	INHA	CLRN1	FBXL7	ST8SIA5	ZIM2	MRAP	SLC16A9	MCF2	CALN1	TMEM26	LYVE1	MCF2L2
**cortical interneuron**	HTR3A	GJE1	SP8	TEX13B	HTR3B	CNR1	TRIM67	PROKR2	HSD3B2	OCSTAMP	NCAN	TEX50	ST8SIA2	SBK3	SHANK1	SP9	MYH8	IFNK	LHX6	DLX1	PSMB11	PRELID3A	CACNG4	RXFP3	CARMIL3	POU1F1	SLC6A1	GPR26	POU3F3	LZTS1	FOXG1	MN1	DLGAP2	RGS21	AMER3	NEXMIF	SOX11	NLGN3	BRINP2	NSG2	SCN3A	IL17F	CHL1	CAMK1G	NRXN3	TUBB3	CUX2	MYT1L	GSDME	REM2
**cortical thymic epithelial cell**	DCD	PSMB11	PAX1	FOXN1	PNLIPRP3	FRMD1	TBATA	SLC46A2	GOLGA6L22	TBX22	GHRH	KRTAP19-3	SCGB2A2	SCGB1D2	IL20	LCE3D	TP53AIP1	OVCH2	CCL25	PRSS16	KREMEN2	CCL19	TEPP	RAB42	PRICKLE4	SCX	DSG1	ZNF575	CLEC2A	ZNF852	PODNL1	CALML3	HOXB13	PRR29	ZNF415	KIF25	ZNF799	IGFL4	ANKRD35	LRCOL1	CXCL11	KDF1	TLR3	CYP3A4	TP63	S100A7A	COL17A1	ZNF750	KRT17	HCG22
**corticotroph**	TBX19	POMC	SBK2	PRL	ARHGAP36	NPAS4	CRHR1	CHIA	SERTM1	TAC4	IGSF1	TMEM190	LHB	DGKK	CLDN9	AVPR1B	LHX3	LAIR2	TDRD9	CGA	MRLN	GCK	LHFPL5	DIRAS3	SMIM18	TAS2R20	RESP18	GNB3	SIX6	RSPH10B	NNAT	TSHB	MROH2A	EPX	OR13C5	SCG2	SYTL4	GHRHR	PRLH	WFDC5	ASCL1	C2CD4B	TCEAL2	CHGB	CBARP	ZNF541	HSPB3	SEMG2	SMIM2	FSHB
**cumulus cell**	CNMD	LHCGR	INHA	HSD3B1	MRAP	BHMT	CYP11A1	SFRP4	AMH	APOA4	AMHR2	MGARP	EPHX2	TNNI3	STAR	KCTD14	HAO2	HSD17B1	PRLR	NRN1	IHH	FADS6	GSTM2	SLC38A5	EDN2	PTGFR	THBS4	HMGCS2	TENM4	MMP11	KRT79	NAP1L5	NPPC	SLC18A2	HS3ST1	HSD11B2	GRB14	DDIT4L	CRISPLD2	DERL3	RASL11A	INHBA	MYBPC3	APOA1	SLC7A4	CPE	FST	SEMA3G	MMD2	SLC38A3
**cytotoxic t cell**	KLRC4	ZNF683	FASLG	SLFN12L	KLRC1	CXCR6	IFNG	IL26	KLRC2	GZMH	IFNL1	GBP5	THEMIS	CD8A	RGL4	GZMA	CD8B	APOBEC3H	CD2	CD3G	GZMB	APOBEC3D	KLRC3	GNLY	XCL2	PRF1	NMUR1	CCL5	FCRL6	SIRPG	CTAGE6	TNFSF14	SPATA31D1	CD96	CD3D	GIMAP7	KLRD1	GZMK	PYHIN1	CRTAM	PLAAT4	GZMM	PDCD1	GIMAP5	KLRB1	CCR5	CD3E	LAG3	TIGIT	APOBEC3G
**dang**	OR4C6	IL37	PAEP	SIGLEC15	CST4	TM4SF18	SOX7	TNFSF18	CYP2B6	ZNF488	PTHLH	CD163L1	CST1	PIK3C2G	IL1A	MMP1	ZBED2	SPANXC	APLN	ABCA10	PRSS3	VSIG1	CCN6	ADRA1D	HAS3	CSAG1	CALB2	MAGEA12	CFHR3	CSTA	RIPPLY2	ERP27	ZNF853	BARX2	WNT7A	PRSS21	TMEM92	LETM2	SIRPB1	CLPSL1	PGF	CCZ1B	CARD11	MAGEB2	GAL	TNS4	IGFL1	SLCO1A2	KRT6A	GRIN2D
**daoy**	PAGE5	TLX3	MAGEA11	TMEM233	COX7B2	PYDC2	PAGE1	SPANXD	HOXA4	HOXC12	FMR1NB	FOXL2	FAM133A	NLRP11	MAGEA6	HOXD13	SPANXC	LCE2A	SPOCD1	CSAG1	NAA11	SLC25A21	SIGLEC15	DMRT2	HOXD11	POTEC	CPA4	KCNG1	KHDC1	BEGAIN	MAGEC2	NKX2-8	RFLNA	BAIAP2L2	MAGEB2	DMRTA2	CD70	FAM72B	SPANXA2	PDLIM4	STEAP1B	HOXA7	PASD1	NKX2-5	FOXE3	CCL2	MAGEA12	CACNG8	CNPY1	DIRAS3
**decidual cell**	SERTM2	GH1	HBE1	HBZ	GDF7	AADACL4	TAS2R1	PAGE4	GPBAR1	PLA2G2A	DEFB124	FOXL2NB	C1R	GJD3	XAGE3	OR8B8	MASP1	WNT2	OSR2	STUM	CGA	HTR2B	ADAMTS8	HAND2	CPZ	C1S	APOD	HSPB6	MRGPRF	TMEM37	ADRA2C	CFD	HBG2	CD248	LMOD1	TAS2R20	ULK4	PAEP	PROK1	CGB3	HOXA6	APCDD1	ANGPTL1	CPXM1	HBA2	COLEC11	BDKRB2	SLC22A11	PTGDS	HBG1
**deiter's cell**	CEACAM16	TECTB	OTOG	P2RX2	OTOGL	GDF6	MANSC4	ODF3L2	CRHR1	TMEM221	OTOS	CAMP	TMEM229A	GJB6	TMPRSS6	TMEM82	CLDN9	KLHL14	HHATL	TMPRSS7	PPP1R17	DGAT2L6	KCNJ10	MS4A3	SLC25A41	CLDN14	TMPRSS3	ANXA9	GAL3ST4	ELANE	CD177	FBXO2	KNCN	RBP7	PTPN5	EPYC	OTOL1	TMEM79	BTNL2	WNT7A	CABP7	UMODL1	OCM	CNTF	KCNJ16	COL11A2	CEMIP	INPP5J	ILDR1	LPAR3
**dendritic cell**	GPR31	LILRA4	CD1C	MS4A4E	CLEC4C	CD1E	HLA-DQB2	CLEC10A	CLEC9A	OR4E1	CIITA	IDO2	HLA-DQA2	FCER1A	MS4A6A	CLEC4A	INSYN2B	CCL22	LILRA1	FPR3	ZNF366	CD300C	SIGLEC9	CCL17	HLA-DOA	HLA-DQA1	CEACAM4	CD1A	XCR1	WDFY4	CD1D	FLT3	CD86	SIGLEC7	ITGAX	MEIKIN	LILRB2	IL12B	CD80	UPK3BL2	JAML	HLA-DQB1	CCDC200	MDFIC2	OSCAR	SLAMF8	TLR10	LILRB1	HLA-DMB	LILRA2
**dental pulp cell**	RSPO4	AMBN	ALX3	SP7	PANX3	WFIKKN2	DIO3	ANGPT4	AMELX	ALX1	LHX8	DLX3	MANSC4	SLC2A7	DLX4	ABCG5	ENPP6	OLFML1	DSPP	KLKB1	SCUBE1	RSPO2	WIF1	FAM167A	FOXF2	KRT82	MKX	MATN4	COL8A2	S1PR3	KRTAP4-16	MSX2	NELL1	ADAMTS20	OMD	ALX4	MMP13	MC4R	PROKR2	INSC	SIGLEC1	PRSS35	AVPR1A	TNN	TLR8	NTN3	FCRL6	DLX5	SSTR4	VIT
**dermal condensate**	ADAMTS20	GLIS1	NTN3	GLI1	FGF10	CACNA1G	TWIST2	ALX4	EGFL6	LIPI	CYP26B1	FREM1	ISM1	COL23A1	TMEM132C	HS3ST6	TBX15	PROKR2	FOXO6	LRRC15	UCN2	EDN3	TBX18	ST8SIA2	FOXD1	SOX18	COL26A1	COL7A1	CPNE5	AVPR1A	WIF1	NGFR	ADAMTS15	KIF26A	KCP	TEX15	SEMA3D	SMOC1	NDNF	PLA2G4B	HEYL	DAAM2	KCNS2	GLI2	TRIL	GRK4	TWIST1	VEGFD	TSPAN11	WFIKKN2
**detroit562**	IRX4	KRT81	IGFL1	GKN2	KISS1	ATP2C2	ZBED2	CST6	DOK7	UPK3B	LCE3E	TMEM139	OR5P2	ANKRD18B	NKX2-8	RNASE7	IRX2	KRTAP4-1	IL24	GJB5	DKK1	ARTN	GPRC5A	SERPINB7	FAM25A	GJB3	PTGES	FAM83A	KCNJ12	DMRTA2	DMBT1	BDNF	VGLL1	COL4A6	S100A2	IL18	IL11	FAM111B	RHOD	ARHGEF35	MMP28	PSG4	GPR87	ADIRF	HOXA11	SOX15	SNCG	AMIGO2	TNS4	FST
**dn1 thymic pro-t cell**	CCR8	CXCR6	RLN3	KRT83	QRFP	IL13	CD160	ICOS	IL4	KLRK1	LY6G5B	IL9R	HRH4	KLRC2	TBX21	CXCR3	XCL1	GPR68	CD3G	LRRC52	KLK8	GPR15	KLRC1	IL2RB	PGLYRP2	LAX1	ITGB7	IL18R1	CTSW	GRIFIN	IL1RL1	TESPA1	TXK	CCR2	SH2D2A	FAM124B	KLRD1	TNFRSF18	CCR6	CCL1	CCR9	CCDC116	CD200R1	CLNK	IFNG	RAB19	GPR18	SKAP1	STAB2	CCR10
**dn2 thymocyte**	MYL10	IL2RA	BPIFC	CPA3	DNTT	QRFP	ADGRG3	TAS1R1	DRC7	TMEM121	TREML2	ATP8B4	MYB	TEDC1	PGLYRP2	CD244	CD160	H2BC1	SPNS3	GPR174	FUT7	NRROS	GPR171	NRGN	NFE2	TNFRSF13B	DPP4	IL12RB2	ADGRG1	MPO	RAG2	SLC46A2	SCIN	TCF7	PLAC8	LAT	SYCE2	LCK	RGS18	IRAG2	IKZF2	RAB44	IL17RB	RHOH	INSL5	CBFA2T3	RIPK3	ZBED5	DKKL1	ITGA4
**dn3 thymocyte**	MYL10	GPR25	PTCRA	DNTT	RAG1	IL2RA	GRIFIN	SLC5A9	ENDOU	CCR9	AQP11	ARL5C	RAG2	CD3G	CHRNA9	RAB44	INSL5	FAM163B	ARPP21	PRSS57	LAT	TMEM121	THEMIS	SPO11	GFI1	RAPGEF3	LCK	LY6D	CD3D	BCL11B	SAPCD1	SH2D1A	GBP6	CD3E	RHOH	SLA2	TTLL9	H2BC1	DTX1	ABI3	SKAP1	MYB	TBXA2R	PGPEP1L	DYNLT5	BST1	IL21R	THY1	LACTBL1	CD247
**dn4 thymocyte**	IZUMO1R	CD8B	CCR8	CCR9	ENDOU	CD3G	DNTT	CD28	SH2D1A	LAT	H2BC1	CD5	LCK	CD8A	CD3D	IL21R	SKAP1	CCR7	GPR18	TREML2	CD6	CD247	GRIFIN	UBASH3A	PGLYRP2	RAG1	TCF7	PTPRCAP	ARL5C	ZAP70	MTARC2	GRAP	CD27	CD2	LIME1	ARHGAP45	SLAMF6	PTCRA	SIT1	THY1	KLK8	CD3E	GIMAP6	THEMIS	SATB1	SH2D2A	CXCR6	SLA2	GIMAP1	MYL10
**double negative t cell (dnt)**	KIR2DL3	GZMH	FCRL6	FGFBP2	GNLY	KLRF1	GZMM	S1PR5	KLRG1	P2RY8	ZNF683	PRF1	SLFN12L	KIR3DL2	KRTAP12-3	NCR3	IFNG	TBX21	CST7	CTSW	NKG7	GZMB	CD3E	KIR2DL1	PTGDR	KIR3DL1	FCRL3	GZMA	CCL5	SYTL3	RNF125	KRTAP16-1	PATL2	KLRD1	CCL4	FCGR3A	XCL2	SAMD3	CD3G	LAIR2	ZAP70	CD6	CD5	LIME1	KCNA3	GBP5	CD247	SLA2	GPR141	CD69
**double negative thymocyte**	GZMK	DTHD1	SIRPG	ZBTB32	FCMR	CD5	TIGIT	SH2D1A	ZNF80	PVRIG	P2RY8	GPR15	CHI3L2	CCR7	CD6	FASLG	CTLA4	IL10	TASL	RGL4	CD28	CXCR5	LGALS9C	CD27	PRDM8	ANXA2R	CD3E	LIME1	SAMD3	ZC3H12D	CPA5	GZMM	LAT	LGALS9B	PGAM4	CD247	CD3D	GIMAP7	ZAP70	S1PR4	TCAF2	SIT1	PARP15	CD8B	CRTAM	CD3G	AQP10	TESPA1	GZMA	IL23A
**double-positive, alpha-beta thymocyte**	CD1B	OR10T2	CD1A	CD1E	RAG2	RAG1	KRTAP12-3	PTCRA	CD1C	CD8B	OR10R2	CD8A	SH2D1A	CHRNA3	TMIGD2	CHI3L2	THEMIS	TRAT1	OR13A1	SH3TC1	CD3E	KCNA3	NPPC	GRAP2	TNFSF8	RTP5	CD1D	CD3G	SMPD3	EVPLL	TSHR	CD3D	ARPP21	CD5	LEF1	TCF7	SIT1	PTGDR2	FLT3LG	DNTT	SLAMF1	CYP2U1	GCSAM	MZB1	P2RY8	CD6	CAMK4	CD247	KIR3DL2	MAL
**dropshilia**	SSX2	GMCL2	TAAR5	CYP27B1	GSTT4	CPO	GRK1	PAEP	LGSN	ART1	ALLC	AMPD1	RGR	CRP	CLC	SPX	PKD2L1	GRP	RGN	HEPH	GIP	PRY	CABP1	ALG10	RS1	ENDOU	DAO	HRG	SOX14	SIGLEC1	PTGDR	REG1A	GK2	APOBEC3D	NAT1	TREH	HASPIN	OCM	APOBEC3H	CHAT	CSN3	ART4	HDC	STUM	RIT2	MMP1	PGR	OPN4	MED26	SAGE1
**drosophila**	MIP	TRPA1	GNMT	CLC	RBP2	CPO	GSTT4	GIP	FA2H	HEPH	BVES	CABP1	SOX14	CRP	IPP	RET	HRG	TRIM9	FAS	SYT7	ART1	NAT1	FST	PDP1	SPX	SLC5A11	DAO	MED26	RS1	AOX1	TREH	HDC	HASPIN	SYP	CAPS	ALG10	PGAP3	OCM	AGO1	RGR	SYT1	MED18	SPEN	DYRK3	SPN	GEM	GALE	MYD88	MOB2	PIGS
**drosphilia**	PSD	BEST2	CLC	NXF2	SOX14	CABP1	CPO	RHO	RBP2	PIP	BVES	SLC5A11	TREH	NAT1	OXT	HRG	NXF3	TTK	GIP	RGN	SPX	CRP	FKBP14	MED26	SOD3	GSTO2	RAB19	SYP	DYRK3	SPN	SKP2	RS1	GRP	GALE	HEPH	CSN3	SHD	ORC1	HDAC4	ALG10	GSTT4	MAEL	OMD	PAK3	PDK1	AGO1	MED18	GEM	E2F1	HASPIN
**drosphilia neuroblast tumor**	SPX	ART1	HRG	CRP	TREH	RS1	HDC	GRP	NXF2	ALG10	CORIN	HASPIN	ART4	REXO5	CLC	SYP	OCM	PEX7	GK2	CSN3	RGR	NAT1	MYB	SRL	NXF3	INSC	MED26	CORT	JMJD7	CABP1	HEPH	GIP	NEB	RAB19	INCENP	TUB	SIRT1	TAF4	DYRK3	E2F1	RBFOX1	AGO1	WDFY2	DPH5	EDC3	ELP1	WDR62	MED18	OMD	E2F2
**duct epithelial cell**	CCDC198	FAM166C	SCTR	CRNN	FDCSP	KCNJ18	ARSL	CALML5	KRT6B	CFTR	PLAAT3	SFRP5	MMP7	TCN1	PDX1	GLYATL2	SCGB3A1	MNX1	SAA2	CALHM3	SLURP1	OR6C74	UGT2A3	PKHD1	CXCL17	SPINK7	H1-0	GABRP	RARRES1	CCL28	NIBAN2	CRCT1	H2BC5	DEFB1	DEPP1	SERPINA4	FAM240C	ADAMTS16	MUC21	ECRG4	PGAP6	H2AC6	SAA1	DAOA	MAB21L4	FAM3D	VTCN1	BLACAT1	RHOV	BPIFB1
**ductal cell of salivary gland**	GRIN2C	OLIG1	RIOX1	CBARP	HES5	IGIP	SARDH	LONRF3	KCNN2	IFITM10	CPXM2	EPHA1	LYPD2	MIA	EYA4	LNX1	PYGO1	NYNRIN	LY6H	IL17RE	FGF9	RPH3AL	RNF208	FAT3	AARS2	FMO1	IZUMO4	PACRG	CEND1	AMOT	TMEM238	ZBTB40	MSX1	ENHO	RUSC2	DEPDC5	KDR	SSH3	HMGCS2	TTC38	DLG3	LYRM9	NCKIPSD	TNFSF12	MCF2L	RDM1	ZBTB7B	SH2D3C	EPB41L4A	SETD1A
**duodenum glandular cell**	OR51A7	SMIM33	A4GNT	FAM240A	KRTAP5-11	CD8B2	LIPF	SRARP	DCHS2	MUC6	KCNE2	OR52I1	CTXND1	ARMS2	SERPINA4	PDIA2	CLDN2	IFNE	IZUMO1	LDLRAD2	PDILT	OR7C1	RNF148	SERPINA5	ELF5	CCKBR	CST9L	ARHGAP40	GLYATL2	CHRM1	ANKRD60	CCNI2	HABP2	TFF2	TCN1	PGC	CCDC198	CFAP99	LINGO4	DKK4	TACR2	GLP1R	NPAP1	CMTM1	GSC	GMNC	BLACAT1	PRSS33	C1GALT1C1L	AQP5
**early embryonic cell (metazoa)**	PRAMEF25	UBTFL1	PRAMEF19	CTAG1A	PRAMEF10	PRAMEF20	PGA4	ZSCAN5B	SLC15A5	TRIM43	OOSP3	TEX13B	PRAMEF17	DPRX	OR4K15	MBD3L5	OOSP2	KRTAP4-6	KHDC1L	TAAR9	OOSP1	KLF17	KHDC3L	PRR20C	PRAMEF6	RFPL4B	ALPG	RFPL4A	TRIM60	PXT1	DPPA2	BTG4	WEE2	NLRP7	DPPA5	FTHL17	TRIM43B	DNMT3L	PRR32	NOBOX	DPPA3	KCNK18	RLN3	SLC25A41	H1-8	THEGL	ZNF280A	HCRT	NLRP14	TERB2
**early lymphoid progenitor**	CYP2C9	OR1F1	PRSS2	IL12A	H3C1	MIDEAS	H2AC7	BPIFC	MPO	FLT3	H4C9	SMCO1	TMEM272	DNTT	MYL10	OTOS	EIF2S3B	LRRC70	SPINK2	ALPK2	RGS18	TASL	CD34	H3-5	MS4A18	CTSG	CLNK	SH2D5	HNRNPCL3	OR51B4	FUT7	HNRNPCL1	ELANE	KNL1	TNFRSF13B	ARL11	S100Z	KCNK17	CALHM6	LMO2	ANGPT1	H2BC21	ADGRG3	ERG	STAP1	BCL11A	CRHBP	SHISA8	IFTAP	PRXL2C
**early t lineage precursor**	KRTAP4-12	QRFP	ADGRG3	SLC35D3	GRIFIN	DRC7	CPA3	MYL10	MPO	DNTT	BPIFC	BHLHE41	PGLYRP2	NCR2	TREML2	SPNS3	NRROS	KCNK17	TEDC1	KLK8	PRSS2	FCRL4	MYB	RAG2	PRSS57	DNMT3B	LAX1	CD244	CCR9	TMEM121	SLC22A3	CD160	ATP8B4	TESPA1	IL12RB2	RGS18	ADGRG1	CD34	BIN2	NFE2	ARL11	SASH3	GPR171	TNFRSF13B	ADGRG5	IL2RA	TBXA2R	PTPN7	PARVG	FUT7
**ectodermal cell**	KLF17	TMEM114	OLIG3	FOXI2	MAGEB16	UTF1	DUOXA2	OLFM4	HOXB1	ELF5	MUC5B	PSORS1C2	ZP4	OR6T1	FTMT	FOXB1	PRSS50	TEX13B	CLDN6	SOX3	NKX1-2	NUP62CL	HES3	FGF8	HAL	ERAS	NR0B1	EPOP	CYP26A1	KPNA7	LHX5	ETV3L	TAS2R3	TAS2R42	TFAP2C	NANOS2	PRDM12	DNMT3L	GTSF1L	KRT76	TUBA4B	FMR1NB	WNT6	MKRN3	TAS2R7	TKTL2	TAFA3	TAS2R9	FAM181A	NPBWR2
**efe184**	ASIP	CLDN16	DEFB4A	FOLR3	MMP1	VSTM1	SIGLEC15	FGF18	HAS3	KRTAP2-3	HNF1B	MAGEA10	ANKRD34B	PAX8	COLEC10	IGFN1	DCDC2	CCR10	TM4SF18	JSRP1	HKDC1	CATSPER1	HMSD	CSAG1	ELFN2	ALPK2	ERVMER34-1	CD70	BEST3	SPANXN5	PDZK1IP1	MAGEA4	BARX1	ZBED2	GPR143	BATF3	DBNDD1	MAGEA12	MNX1	KLRG2	TLL2	PADI1	QPRT	KRT81	TRIB3	ASPHD1	KRTAP4-8	KCNMA1	CLDN1	KCNK15
**effector cd4-positive, alpha-beta t cell**	CFHR4	FCRL6	PVRIG	STPG4	ZC2HC1B	LY6G5B	CXCR1	GZMH	CD40LG	PZP	TLDC2	FGFBP2	KRTAP5-1	THEMIS	PATL2	KLRG1	SLFN12L	TMEM273	ZACN	PRSS53	TM4SF19	ZNF683	FASLG	FUT5	OR2A2	PRF1	S1PR5	HCRTR1	MYL10	CD5	CX3CR1	SLAMF1	FSCN3	ZNF831	RIPOR2	GPR174	OR10S1	OR4D1	PTGDR	CD6	GNLY	LTB4R2	SLAMF6	OR5K2	CEACAM21	CD4	ITK	CYBC1	TBX21	PYHIN1
**effector cd8-positive, alpha-beta t cell**	KIR2DS4	KLRC4	GZMH	FCRL6	ZNF683	FGFBP2	PVRIG	CD8A	GNLY	CD8B	PRF1	KIR2DL3	FASLG	GZMA	KLRF1	KLRG1	KLRC3	GZMB	KIR3DL2	GZMK	IFNG	KLRK1	ZNF80	KIR3DL1	KIR2DL1	KLRD1	GZMM	PATL2	CTSW	CST7	NKG7	SAMD3	XCL2	S1PR5	PTGDR	FCGR3A	CCL5	THEMIS	FCRL3	TBX21	CXCR1	LAIR2	PYHIN1	LITAFD	APOBEC3H	SLFN12L	KRTAP16-1	OR2T5	H3C1	TIGIT
**effector memory cd4-positive, alpha-beta t cell**	GZMK	FLT3LG	TRAT1	P2RY8	CD40LG	CD6	CD5	CD3E	IL7R	KLRB1	OR10K1	TNFRSF25	GZMM	CXCR3	TBC1D3E	OR7A17	OR51M1	KLRG1	GZMA	H4C7	CCR6	GIMAP7	OR4F17	IFNL1	GBP5	LTA	OR10H1	RTP5	OASL	KRTAP12-3	WNT1	CD3D	TCAF2	KCNA3	CD2	SLFN12L	CCR7	SNAI3	OR1A1	SLAMF1	ZAP70	RGL4	LTB	CD28	ZNF831	CD3G	HLA-G	CCL5	CD247	TNFSF8
**effector memory cd8-positive, alpha-beta t cell**	KIR3DL3	KIR2DL3	KIR3DL2	KLRC3	GZMH	FCRL6	CD8A	RTP5	ZNF683	CD8B	KLRC4	GZMK	FGFBP2	KIR3DL1	GZMM	KIR2DL1	UPK3BL2	KLRG1	GNLY	P2RY8	PRF1	XCL2	CST7	FCRL3	GZMA	NKG7	CD3E	S1PR5	CRTAM	TBX21	CTSW	OASL	CCL5	GZMB	IFNG	KLRF1	FASLG	KLRD1	SAMD3	TRAT1	CD6	FCGR3A	SLFN12L	CD3D	APOBEC3G	CD7	ZAP70	CD5	KDM4F	NCR3
**efm192a**	CLEC3A	S100A7	KRT37	SPTSSB	KRT81	KRT4	HOXC13	RNF223	ERBB2	RNF224	ANKRD30A	S100P	PAGE2	INHBB	HOXC11	BCAS1	ZNF467	PGAP3	FAM25A	LRATD2	SPDEF	ZNF704	SLC49A3	NT5DC4	GRB7	SEMG1	IVL	PRAC2	HOXC10	ADIRF	FOXA1	ABCC3	S100A14	VTCN1	EPN3	CHAMP1	PHEX	DUSP13	PCGF2	CLDN9	FAM91A1	EPPK1	RHOV	NUTM2E	TFAP2C	ZG16B	KRT80	STARD3	ZHX1	KCNMB1
**ekvx**	CYP24A1	RAET1G	LGSN	DIRAS3	MAGEA4	BARX1	MAGEA1	SPINK6	LCE3E	MAGEA10	KRT81	RSPO3	TFPI2	TMSB4Y	RHOV	IGFL2	PRSS21	MAGEA3	TRIML2	GABRQ	PITX3	FIBCD1	CXCL5	FAM83A	C1QL4	PRAME	HORMAD1	RIMBP3C	S100A3	TCHH	SYT17	TRIM7	CD70	HBQ1	PODNL1	RNF212	MATN3	MESP1	OPN3	HOXA4	HSF2BP	TESMIN	GPRIN2	AP1M2	CGB2	BEST3	CRB1	FSTL3	ZNF280A	P4HA3
**el4**	INSL5	OVOL3	PDCD1	IL17F	KRTAP10-10	SAPCD1	SLC6A13	PMCH	FAM205C	CCR8	MPP4	VWA7	TMPRSS11F	ASCL4	IZUMO1R	IL9R	PKD1L2	TMPRSS11E	LEXM	DNAJB13	INAFM2	IGSF23	CCR9	FAM151A	GCSAM	DCLK3	TSPAN32	SNX22	CFAP157	SNX31	SLC24A5	CD3G	SLC36A3	CCDC116	MYLK4	PYROXD2	CPA3	ARL11	TMEM265	MILR1	MC5R	ULBP1	TCF7	FGD1	ESM1	TNP2	CDCP2	SMCO3	HOXD8	SLA2
**embryonic cell (metazoa)**	PRAMEF9	TSPY1	H2AC4	IL9	KRTAP12-1	H2BC1	NMBR	OC90	EPX	SSTR5	PRAMEF25	MAGEB3	NKX2-6	WEE2	NPY2R	H4C4	NPY5R	TRIM43	FOXI3	MAGEA8	SULT6B1	MAGEB1	NOBOX	H2AC13	VHLL	H3C13	FOXB2	MBD3L2	EPO	FOXD4L1	ALPG	ASB11	SLC18A3	KRTAP6-2	VRTN	OTOP1	UBTFL1	SEPTIN12	EIF4E1B	BMP10	F8A2	H2AC15	RRH	H2BC14	MC2R	KLF17	PKDREJ	H4C1	RPTN	PRAMEF17
**embryonic fibroblast**	LCE1F	AGTR2	KRTAP6-3	HTR1B	S100A7A	CCL7	FAM180A	EDA2R	HAND2	RTL3	TMEM202	KRT33B	ALKAL1	VEGFD	LIPK	TNMD	LOX	KLHDC8A	TWIST2	FBLN2	GPR50	EXOC3L4	SPRR2B	KRTAP22-2	ADAMTSL5	BDNF	CRCT1	COX6B2	LBHD2	EN1	CCN5	MSLN	COL8A1	DDIT4L	PRR32	GDF6	NGF	H2BC1	IL1F10	ASPN	OGN	PRRX1	HS6ST2	CDKN2B	KRT33A	PRR9	P3H4	TCP11X2	CARD19	LOXL1
**embryonic hemocyte**	RS1	ART1	GIP	CLC	TREH	CPO	CABP1	CRP	SPX	OCM	RGR	SOX14	HRG	NAT1	OXT	ALG10	GRP	SRL	CSN3	NXF2	GSTT4	CHAT	WDR62	MED26	STAC	MIP	HEPH	REXO5	COL4A1	PDP1	FKBP14	GK2	DRGX	ART3	KEL	ALG11	HDAC4	ELP1	CORIN	ARX	RGN	OTP	MYB	RNASEK	SIX4	HASPIN	RAB19	TTK	AGO1	DPH5
**embryonic stem cell**	MYMX	TRIML1	TAAR6	FGF4	ZSCAN10	PRAMEF17	TDGF1	ZFP42	TRIML2	NODAL	NANOG	DPPA2	LEFTY2	MORC1	GDF3	PRAMEF12	DNMT3L	CRYBA1	ESRRB	POU5F1	UTF1	FOXH1	TEX19	FBXO15	SLC28A1	MAGEB16	ERAS	DPPA4	PLA2G1B	TRH	SLC7A3	USP17L5	ZFP57	PRDM14	PRAMEF25	GLRA1	TRIM6	GTSF1L	RBMXL2	LRRIQ4	VRTN	GOT1L1	NR0B1	TRIM71	MYH13	FAM25C	SALL4	ADRB3	OOEP	PYDC1
**endocardial cell**	GPR182	ENPP6	CYTL1	AHSG	BMX	SPIN2A	SOST	NPR3	COMP	MYL7	MMRN2	COLEC11	VWF	TBX20	GATA5	EMCN	PTX4	WNT2	PLVAP	NOS3	TNNT2	ECSCR	TBX5	HAPLN1	TNNI1	ART4	CSRP3	TEK	MYBPC3	UNC45B	HAND2	POPDC2	LRRC10	GATA4	NPPA	ASB4	MYL4	ASB12	MYH6	SMTNL2	CFC1	CGNL1	ADCY4	KLHL4	KCNG2	CD93	HMCN1	CDH5	SFRP5	PRRT4
**endocardial cushion cell**	TBX20	MSTN	PRSS35	COL9A1	MEOX1	WNT16	ADAMTS19	HAPLN1	BTN1A1	POSTN	PDLIM3	SCX	ASB4	FBN2	FBLN2	NRK	MFAP2	GATA6	LUM	IGF1	TMEM26	SFRP2	GATA4	RASL11B	HAND2	ELN	VSIG8	TWIST1	VCAN	COL6A6	KRT82	MYH7	TGFB2	DPT	BMPER	MFAP4	GPR45	PTN	SCUBE1	LOXL2	COL5A2	TCF21	COL9A3	MYH10	WNT11	GAL3ST4	TNNI1	PLIN1	GPC3	FSTL1
**endocrine cell**	NEUROG3	PYY	A2ML1	GCG	GAST	FFAR2	IAPP	FEV	CLPS	FFAR1	SLC38A5	CRYBA2	CARTPT	AMBP	NKX6-3	MRLN	TTR	KRTAP17-1	SCT	RBP4	G6PC2	POU3F4	GHRL	ABCC8	PCSK2	PNLIPRP1	CCK	CHGB	SERPINA6	PAX6	GIP	BTBD17	ISL1	NEUROD1	PPY	PLA2G2D	IGFBPL1	CHGA	CTRB1	PCSK1	CPA2	DLK1	PLAC1	ZCCHC18	MIA	SYT13	RESP18	AFP	GYPA	HAP1
**endodermal cell**	MAGEB17	TRPC5OS	CER1	PLA2G12B	ARID3C	AQP8	SMLR1	TEX19	PGA5	APOA4	GPR32	MAGEB16	LECT2	ERICH4	SLC13A4	TRIM43	SPP2	PNMA5	CUBN	FMR1NB	SOAT2	DPPA2	DGKK	MRGPRX1	CREB3L3	UTF1	APOB	LIN28A	AFP	PRAMEF17	APOM	CYP26A1	TBXT	ZSCAN10	AMN	SALL4	CLDN6	FGF4	VRTN	TRIM71	SSX4	TDGF1	ERVV-2	TMEM82	S100G	DNMT3B	SLC2A2	FGF3	ZFP42	DNMT3L
**endothelial**	EPHX3	SOX18	UNC45B	GPIHBP1	MYZAP	ADAMTSL3	PLVAP	AARD	ECSCR	PIGY	LYVE1	GRAP	SRMS	PRND	CDKN2B	PTPRB	TEK	CD93	RGS9	KDR	KLK8	DCDC2B	TMEM100	VWF	SHROOM2	ROBO4	APLNR	CYP4B1	USHBP1	BMP15	ENPP6	CCM2L	ESAM	FLT4	CLDN5	NOSTRIN	ENHO	ESM1	TIE1	CD38	TBX2	ARHGEF15	ADAMTSL2	CD300LG	KANK3	NXPE2	CHST2	ADCY4	CLEC14A	EMCN
**endothelial cell**	SLCO1C1	SELE	FCN3	FCN2	CLDN5	TMEM252	SRARP	PPIAL4D	C1QTNF9	CDH5	VWF	BCL6B	SOX17	FLT1	EMCN	CLEC14A	SLC22A8	CYYR1	ADGRL4	SOX18	PTPRB	ROBO4	FAM110D	ARHGEF15	TIE1	APLNR	ADGRF5	TEK	MMRN2	CCL14	CCM2L	ECSCR	SHANK3	LY6L	USHBP1	KDR	KRTAP4-11	ERG	GPR4	ADCY4	PCDH12	SEMA3G	HSPA12B	SHE	CD93	RAMP3	MYCT1	DLL4	CLEC1A	KANK3
**endothelial cell of artery**	SLC5A4	ANO2	DKK2	CPAMD8	OR2A42	OR2A14	GJA5	CC2D2B	VWF	FCN3	ERICH4	LCN10	DEPP1	SEMA3G	SPAAR	INSYN2A	ST8SIA6	SNTG2	SHANK3	GNA14	SLC22A24	BMX	LDB2	TMTC1	TLL1	AWAT1	TDRD10	DLL4	TACR1	NOTCH4	CALCRL	PTPRB	BTNL9	PALM2AKAP2	RASGRF2	OR2A12	ARL15	FGD5	GSG1L2	RXFP1	PREX2	ZNF366	FBXL7	ST6GALNAC3	PTPRM	OVCH1	RAPGEF5	ERG	CPHXL	ZNF423
**endothelial cell of coronary artery**	C1QTNF9	AQP7	TCF15	GPIHBP1	CD300LG	KCNA5	MEOX2	USHBP1	BCL6B	TIMP4	SOX18	PRND	UNC45B	RTP3	FABP4	ARHGEF15	HSPA12B	CLEC1A	CDH5	SLC28A2	LIMS2	SOX17	NOS2	GPR4	ADCY4	BTNL9	CD36	MYCT1	MMRN2	NPPA	TIE1	CLDN5	RASIP1	DLL4	TMEM88	ROBO4	ECSCR	APLNR	FAM124B	KDR	KANK3	ADGRF5	FAM110D	NOS3	CD93	ADGRL4	JAM2	CAVIN2	EMCN	FMO1
**endothelial cell of hepatic sinusoid**	CLEC4G	OIT3	STAB2	GPR182	DNASE1L3	FLT4	GPIHBP1	FAM167B	CD300LG	CLEC1B	PCDH12	MRC1	IL1A	TPBGL	TMEM132E	STAB1	FCGR2B	WNT2	CYP26B1	C5AR2	ROBO4	PTPRB	USHBP1	KDR	LYVE1	EHD3	CLEC14A	ARHGEF15	BMP2	HPX	MMRN2	NPR1	WNT9B	CDH5	SOX18	CYP4B1	AQP1	COL13A1	FAM124A	APOA5	TEK	GJA4	CLDN5	PLXNC1	CLEC9A	ALB	COLEC10	C1QTNF1	BTNL9	SCARF1
**endothelial cell of lymphatic vessel**	CCL21	MMRN1	LYVE1	FLT4	STAB2	KRTAP4-11	SPINK7	DIPK2B	KRTAP10-9	CLDN5	FDCSP	GRAPL	KRTAP10-2	CRNN	GJC2	KLHL4	TSPEAR	TBX1	LCE3A	GPR182	SPRR2D	LRCOL1	PROX1	ECSCR	OR51G2	PGM5	PKHD1L1	MYZAP	TIE1	NTS	CCL14	RELN	LRRC70	SPRR2F	ROBO4	KANK3	BCL6B	EFCC1	ADCY4	PCDH12	CLEC4M	TFF3	DUOXA2	CCM2L	STAB1	CDH5	TM4SF18	BMX	SHE	SCGB1D2
**endothelial cell of sinusoid**	CLEC4G	OIT3	STAB2	GPR182	TPBGL	GPIHBP1	CLEC1B	FAM167B	DNASE1L3	FLT4	PCDH12	CD300LG	EXOC3L2	LYVE1	FCGR2B	MRC1	KDR	PTPRB	STAB1	EHD3	ARHGEF15	C5AR2	WNT2	BMP2	CLEC14A	AQP1	BTNL9	NPR1	CDH5	COL13A1	MMRN2	CYP26B1	TMEM132E	FAM124A	ROBO4	USHBP1	ADGRF5	CLDN5	CLEC1A	TEK	ADAM23	ADGRL4	SMIM9	CYP4B1	FABP4	CYYR1	PDE2A	PLXNC1	MYCT1	RNASE10
**endothelial cell of vascular tree**	CCDC194	TMEM252	SMIM33	C1QTNF9	CSF3	WNT9B	SELP	SELE	OR13G1	GMCL2	KRTAP10-2	OR4A16	OR2G2	GNG14	OR13J1	OR1C1	KRTAP6-3	CXCL9	TAS2R41	LCN10	GDPD3	FAM110D	OR6F1	OR52N1	DPEP2NB	OR56B1	ZNF878	OR5H2	SMIM9	FABP4	TAS2R9	OR52N4	AQP7	RBP7	OR52I2	SPTBN5	SCYGR4	KRTAP4-3	GPIHBP1	ERICH4	OR4K5	TPO	FOXD4L4	SPDYE2B	SLC10A6	LHX6	CD300LG	OR5W2	CCL16	LCN6
**enteric neuron**	SMIM28	PHOX2B	IQCJ	FAM163A	PHOX2A	CHRNA3	SLC5A7	SULT6B1	NXF5	HAND2	TMC3	TLX2	SLC18A3	CHRNB4	HMX2	VIP	ASCL1	DBH	CNGB1	TMEM271	PIRT	PRPH	SEPTIN3	RAB41	RET	CNTNAP5	CHRM2	ZNF804A	ZNF536	NOS1	OPRD1	PLPP4	EML5	SEPTIN5	TAC3	HOXB5	KCNQ5	CHRNA7	H1-0	MAB21L1	ELAVL4	ALK	OR4N2	GAPDHS	PNMA8C	XKR7	MAGEC3	KIAA0408	ISLR2	HOXD3
**enterocyte**	ENPP7	SCYGR2	MS4A18	ALPI	BTNL3	MS4A12	CYP3A4	TMEM275	G6PC1	LYPD8	MUC17	OSTN	TM4SF20	SLC10A2	RBP2	FABP2	OR9Q2	MS4A10	KRT20	MEP1B	GUCA2A	MUC3A	SLC51B	APOA4	GUCA2B	ERICH4	NPC1L1	UGT2B17	LCT	PRAP1	CEACAM7	CDHR2	BTNL8	TMEM253	CEACAM20	FABP1	ISX	BNIP5	MYO1A	CDHR5	KRTAP13-2	MGAM	CDH17	CHP2	URAD	MT1HL1	PPP1R14D	MEP1A	SLC6A19	REG3G
**enterocyte of epithelium of large intestine**	OR51D1	OR7E24	CEACAM20	HIGD2B	MS4A12	OR1I1	GUCA2A	AQP8	TMIGD1	KRT20	FABP2	TM4SF20	SLC10A2	CDHR2	SPRR2B	B4GALNT2	CEACAM7	ISX	SLC26A3	TRIM40	MUC17	SLC17A4	PPP1R14D	NLRP6	ALPI	ENTPD8	CDHR5	MEP1A	MEP1B	TRIM31	MYO1A	MYO7B	DIO1	SLC13A2	SULT1B1	TUBAL3	METTL7B	FAM83E	NR1I2	PHGR1	PRAP1	CLDN23	ADGRG7	TRIM15	BTNL3	DMBT1	EPS8L3	CLRN3	PTPRH	KRTAP13-2
**enteroendocrine cell**	MLN	PAX4	BNIP5	INSL5	IFNA14	GIMD1	FEV	SSTR5	ADGRG4	RXFP4	UCN3	PYY	GUCY2C	GPR119	AVPR1B	FFAR1	CEACAM18	LYPD8	SCT	KRT85	REG4	TPH1	SMIM32	TM4SF20	GCG	NEUROG3	CRYBA2	KRT20	RFX6	VWA5B2	MUC17	INSM1	SCGN	OR51E1	URAD	SLC18A1	SLC6A19	MS4A10	ENPP7	CDHR5	CDH17	NKX2-2	CEACAM20	KLK12	TM4SF5	PLA2G2F	BTNL3	HEPACAM2	MS4A8	KRT82
**enucleate erythrocyte**	HBM	GYPB	HBG1	AHSP	HBZ	GYPE	HBQ1	HBG2	IFIT1B	ALAS2	GHRHR	GYPA	HBA1	RHCE	TSPO2	SLC4A1	HBB	EPB42	HBA2	LHB	HEMGN	TRIM49	OR51V1	TSHB	SPTA1	KLF1	PHOSPHO1	FSHB	SLFN14	PRLHR	RHAG	TRIM10	CGA	HBE1	OTOS	FCN2	IGSF1	KRTAP4-9	ARHGAP36	KEL	RHD	GATA1	KRT1	PRL	TUBB1	TRIM58	MYL4	TMCC2	POU1F1	HTR3B
**eosinophil**	EPX	RIMBP3B	PRAMEF5	OR1L3	OR10Q1	KRTAP2-2	CHIT1	HSFX2	CCL18	OR4K14	DAZ2	IDO1	F8A2	OR5W2	CASP5	SIGLEC11	SUCNR1	OR52B6	GOLGA8O	GCSAML	KRTAP5-4	HRH4	GOLGA8J	TRIM64C	OR4F5	FFAR3	FCGR1A	CA6	MRGPRX1	CCR3	MMP25	CXCL11	TP53TG3C	TRIM48	CD101	AWAT2	TREM1	APOC2	OR8H2	OR1J1	NPIPA7	OXER1	IFIT1B	RBMY1E	CYSLTR2	SIRPB2	IL2RA	TBC1D3F	GOLGA8F	TRIM64B
**ependymal cell**	CRYGN	GOT1L1	FAM216B	DYNLT4	TMEM212	LRRC74B	CCDC153	TTLL8	VWA5B1	CFAP126	MRO	RIIAD1	AK7	STOML3	CROCC2	FAM166B	CATIP	SLC39A12	MEIG1	CFAP161	CCDC33	SLC25A18	ADAMTS20	CFAP100	MLC1	DRC7	TEKT4	CFAP77	DNAI3	RSPH4A	CDKL4	DYNLRB2	FERD3L	CFAP52	FOXJ1	RSPH10B	FRMPD2	KCNJ10	SLC14A2	ENKUR	SNTN	ANKRD66	CACNA1S	GHRH	LRRC43	CFAP206	PRR18	CFAP46	PPP1R32	CFAP65
**epiblast cell**	MRGPRX1	GPR32	ERVV-2	LIM2	GNRH2	AADACL3	NODAL	TBXT	MT1HL1	HHLA1	ADAM18	ZSCAN10	CGB5	LGALS16	CCR8	PRR23B	PRDM14	LEFTY2	IL36RN	PKD1L1	KDM4E	KLK13	TNFRSF8	CLEC6A	TDGF1	ERVFRD-1	ZNF729	UTF1	UNC93A	NKX1-2	MS4A15	VENTX	GDF3	DPPA2	HYAL4	CGB8	PSORS1C2	ATP12A	FGF8	TCP11X2	SLC35G3	TLL2	ABHD12B	RTP3	CGB7	ERVV-1	ERAS	NANOG	PLA2G4F	VRTN
**epicardial adipocyte**	CHST4	PKHD1L1	PRG4	UPK3B	LRRN4	ECRG4	MSLN	ITLN1	RSPO1	NXPH2	PTPRQ	CST9	XPNPEP2	BNC1	ADAM33	TM4SF5	HSD3B2	MYRF	TGM1	S100A7A	SBSPON	TBX18	NKAIN4	KCNK4	FYB2	WT1	MEDAG	TNFRSF8	SLC26A3	UPK1B	CALHM4	SLC23A3	CLDN15	COL6A6	FMOD	MYL7	ALDH1A2	CTNNA3	SULF1	PTGIS	LRP2	PLA2G2A	SLURP1	HAS1	CBLN2	AQP1	CALCB	WNT2B	CDON	ADGRD1
**epicardial cell**	LRRN4	UPK3B	MYL7	KCNE5	CFHR2	TBX18	APELA	RSPO1	TM4SF5	CST9	C4A	UPK1B	ANGPTL7	TCF21	WT1	BNC1	MSLN	NKAIN4	FADS6	TNNI1	SMIM1	NRK	CHST4	MYRF	CAPN6	CRYGB	ILDR2	TMEM108	ADAM33	HAND2	PKHD1L1	SEMA3D	TBX20	CRB2	SLURP1	ALDH1A2	PTGIS	FBLN2	TMEM151A	NPR3	PROKR2	CPZ	SOWAHD	FMOD	GDF6	GPC3	GATA6	MYBPC2	SULF1	PLPPR5
**epidermal cell**	KRT77	LIPM	MT4	CHIT1	KRT1	LY6G6C	KRTDAP	THEM5	FAM25C	PSAPL1	PLA2G2F	CASP14	CAPNS2	PGLYRP4	CLCA2	KRT78	BBOX1	PLA2G4F	ANXA8	KRT76	HS3ST6	ATP1B4	KPRP	KRT12	LGALS7	HYAL4	ALDH3B2	SERPINB12	KCNK7	GJB4	DSC3	ADGRF4	TMOD4	BNIPL	KLK9	WNT3	TRIM29	LCE1B	UCMA	IL36RN	SBSN	PKP1	SERPINB2	TMPRSS13	AADACL2	FAM83C	RHBG	LTB4R2	NIPAL4	GPHA2
**epidermal ciliary cell**	TAS2R42	ODF3	TEX47	RSPH6A	CLDN34	FOXN4	TEX49	ZNF534	SPATA48	SPATA45	MISP3	OMP	CCDC103	CATIP	CCER1	KRT76	KIAA2012	ZNF750	CCDC63	STMND1	HAL	SPAG8	FTMT	CCDC105	CFAP52	ANKRD60	ZP4	FOXJ1	TEKT3	TEX36	KPNA7	ANKRD7	KRTAP16-1	DNAI2	CCNO	ODAD4	MUC5B	SPATA4	DAW1	ODAD3	SPAG6	CCDC42	PSCA	ODAD2	PLSCR5	TFAP2C	CFAP161	TAS2R3	NOXRED1	MKRN3
**epidermoblast**	NKX1-2	HES3	ERAS	HOXA1	WNT8A	HOXB1	EPOP	GBX2	FOXB1	CDX1	OLIG3	SLC7A3	VRTN	APELA	ZIC3	DNMT3B	MOGAT2	TAL2	PLPP4	CDX2	CARNMT1	DQX1	LPAR4	SALL4	LIN28A	DKK4	FGF8	RTL8B	PRSS41	SELENOH	PTF1A	JPT2	SOX3	RIOX1	TBXT	HPF1	CNTFR	SP5	GON7	WDCP	RBPMS2	PRTG	HIKESHI	NECTIN2	MAIP1	TRH	ZIC5	RASL11A	SHISA8	AUNIP
**epididymal clear cell**	EDDM3B	ATP6V1G3	FOXI1	WFDC13	SPINT4	SPINT3	GDF1	SLC26A3	NXF3	LCN9	IL13RA2	ATP6V0D2	RNASE9	SPINK14	EDN3	MCF2	VSIG1	CLCNKB	PLET1	CES5A	STAP1	CPSF4L	DMRT2	CUZD1	MUC20	CLCA2	RTL4	KLKB1	PLIN4	SPAG11A	PRR27	RGS9	PLA2R1	RNASE12	DEFB1	WFDC9	OPRM1	IFNK	TFCP2L1	SVOPL	KRTAP3-2	CLDN8	ATP6V1B1	INSRR	ATP6V1C2	AVPR1A	BSND	KRTAP12-1	EXOC3L4	TDRP
**epididymis glandular cell**	DEFB129	DEFB118	DEFB110	DEFB127	SPAG11B	PATE1	DEFB132	RNASE13	WFDC10A	EDDM3A	DEFB135	SPAG11A	PATE3	ELSPBP1	WFDC9	CST11	CLPSL1	TEDDM1	CLPSL2	RNASE11	CST9L	LRCOL1	DEFB134	DEFB108B	BRS3	DEFB126	DEFB119	WFDC11	SCGB1D4	EDDM3B	SPINT4	LCN8	CRISP1	DEFB123	WFDC8	LIPI	DEFB136	ADAM7	PATE2	KRTAP5-2	LCN12	NPFF	GPX5	SCGB1D2	SPINK13	PATE4	SCGB2A1	MUC15	WFDC6	MAGEB4
**epididymis narrow epithelial cell**	SPINT4	IAPP	ATP6V1G3	FOXI1	LCN9	IL13RA2	ASCL4	NXF3	GDF1	MCF2	SLC26A3	CLCA2	VSIG1	CST11	CLCNKB	PLEKHD1	STAP1	ATP6V0D2	INSRR	CPSF4L	EDN3	SVOPL	CYP1A1	RNASE11	DMRT2	SPAG11A	IFNK	IL17C	KLKB1	WFDC9	CES5A	PLET1	TGM3	TMEM202	IFNB1	EDDM3B	SPINT3	OVCH2	TMEM151B	BSPH1	RNASE10	PLA2R1	LCN8	CUZD1	RSC1A1	BSND	LTBP2	MUC20	WFDC13	BMP8A
**epididymis principle epithelial cell**	LCN9	RNASE9	SPINK14	SPINT3	CES5A	WFDC13	EDDM3B	CUZD1	CRYGC	CRYGB	WFDC9	ADAM7	CST11	SPAG11A	RNASE12	LCN10	RNASE10	TEDDM1	SPINT4	CLPSL2	OVCH2	PATE2	GDF1	WFDC11	WFDC8	CWH43	EPPIN	LCN6	BSPH1	FXYD4	SRD5A2	ABCB5	RNASE13	AQP2	GLB1L3	PRR27	RNASE11	SPINK13	LCN8	LRCOL1	CLDN14	LYPD8	CRYBA4	LDHD	LCN12	KRT35	C4BPB	ANXA13	CST8	ADIG
**epithelial cell**	FDCSP	ERFL	PRAMEF26	HEATR9	KRT76	SPINK7	CLDN17	DSG1	DEFB131B	SERPINB12	LGALS7B	KRT3	CCDC195	CMTM1	SPRR2D	S100A7	NIBAN1	TP53AIP1	TAC4	SLFN12L	CRNN	LCE3A	LCE3D	SPRR3	H2BC3	SPRR1B	SPRR2F	SLC2A7	GIMD1	INSYN2B	THEMIS	TEX52	INKA2	ZNF831	OR2AG2	SMIM41	OR1L8	SPRR2A	MRGPRX4	CCDC200	FAM240C	ANKUB1	TGM7	SCYGR4	SLC47A2	SCGB1D2	SCGB1D4	SFTPA2	FAM83C	DCT
**epithelial cell of amnion**	POU5F1B	DIO3	POU5F1	CCKBR	HAND1	SEMG1	ZFP42	ZSCAN10	TDGF1	MSX2	TRIML2	NTS	DPPA4	CLDN6	VRTN	GABRP	FOXH1	CTSV	SLC7A3	TAC3	WNT6	PYDC1	HAPLN1	USP44	WFIKKN1	CYP26A1	UPK2	OR8S1	LRRN1	PLA2G2A	KCNK12	CA14	SHISAL2B	CBS	IGDCC3	DOK4	LIN28A	CLDN10	GYG2	TENT5B	FGF19	HTR1E	TRIM55	CRIP3	UGT3A2	SHD	NPPB	HOXB6	NPFFR2	DNMT3B
**epithelial cell of distal tubule**	OR4C3	HSFY1	TRIM49C	SPDYE11	POTEA	GPX6	SLC12A3	BSND	OR5H1	OR11H4	TMEM207	OR9Q1	MYMX	UMOD	KCNJ1	MYOCOS	ZNF705B	OR7G2	CCDC195	LRRC66	OR2F1	DEFB125	CLCNKA	EGF	TRPV5	GCGR	CALCR	TBL1Y	TRPM6	RBMY1E	CLDN19	CDH16	CLCNKB	HSFY2	SLC12A1	TMEM52B	ATP4A	SLC9C1	AVPR2	OR7C1	OR56A1	LRRC31	MCCD1	CPT1B	TRIM51	TMEM213	PIK3C2G	GBA3	TGM6	CLDN8
**epithelial cell of large intestine**	NLRP6	AQP12B	CLCA4	TM4SF20	B4GALNT2	ISX	FABP2	DMBT1	GUCA2A	TUBAL3	FAM83E	SYCN	PPP1R14D	GUCY2C	RNF186	OTC	FBP2	SLC17A4	MUC3A	PHGR1	MS4A12	NR1I2	LGALS4	ENTPD8	MYO1A	EPS8L3	GPA33	MGAM2	CDX1	HTR3E	CDH17	BTNL3	TRIM31	CDHR2	NOXA1	PIGR	KCNE3	METTL7B	CLDN2	REG4	HAO2	CDHR5	HMGCS2	NOXO1	MGAM	MYO7B	CEACAM7	CLRN3	ALPI	SLC39A5
**epithelial cell of lung**	UBE2L5	DEFB4B	CHIA	SFTPA1	SFTPC	SFTPB	DEFB4A	SFTPD	LAMP3	CLDN18	ROS1	SFTA2	SLC34A2	AGER	SEC14L3	NKX2-1	SCGB3A2	KRTAP19-3	SLC26A9	TM6SF2	SCGB1A1	SCGB3A1	LGI3	C4BPA	OR51B5	CXCL17	SLC22A31	CTAGE8	SFTPA2	CTAGE4	SMIM43	OR5B12	RBPJL	GKN2	TTC6	PGC	SNTN	MUC21	CHI3L1	SEC14L4	TMEM78	PLA2G1B	FAM216B	USP17L7	PVALEF	BPIFB1	SDR16C5	TBC1D3L	PIK3C2G	CCDC198
**epithelial cell of pancreas**	PTF1A	RBPJL	CCKAR	PNLIPRP1	CTRB1	SERPINA6	CPA1	CLPS	CPA2	BHLHA15	HABP2	CEL	SERPINI2	CXCR2	AFM	AQP12A	AMY2B	CELA2A	SLC7A14	CPN1	AMBP	NKX6-1	NPHS1	CELA3B	NEUROG3	KRTAP4-16	CELA1	CHRNA4	NKX2-2	CPB1	FREM2	TOR4A	FFAR2	CAPN6	MAT1A	SERPINA10	CLDN6	CYP2E1	IL22RA1	LRRC2	RAB33B	KIRREL2	TINAG	PRSS2	SLC38A4	LAMP3	SLC28A3	LGI2	PDIA2	ADAMTS16
**epithelial cell of parathyroid gland**	PTH	GCM2	MYBPC2	PAX1	IL1F10	GPR32	LIM2	CASR	VMO1	CLDN9	RD3L	PVALB	PLSCR5	LGI2	SCX	SYCP2L	DMRT2	PTGDR2	WDR86	FOXL2	CORIN	CYB5R2	ERVFRD-1	OC90	KLHDC7B	HCAR1	COL2A1	DMRT3	NRN1	LCN12	RASAL1	IL11RA	RGL3	FAM171A2	MED14OS	EMX1	RAB6C	FOXO6	TMC3	RAPGEF5	LRRC10B	ZNF316	ZNF132	SMIM10L2B	ESPN	SPAM1	CCNA1	ZNF835	KCNJ12	PNMT
**epithelial cell of prostate**	KRT2	KRTAP8-1	PNLIPRP3	OPN4	SRY	DUOXA2	KRTAP9-1	AWAT2	OPRPN	KRTAP13-2	KLK3	CYP4F8	USH1G	TGM4	IFNA5	IFNA7	ALOX15B	IFNA4	KRTAP3-1	IL9	KRT79	ELOVL3	KLK2	SMIM31	CHRNA7	TINAG	CXCL8	TMEM238L	KRTAP1-1	GSDMA	HOXB13	ADGRF1	OLFM4	TRHR	KRT36	CXCL17	CA6	MMP7	NKX2-2	KRT23	AZGP1	IRX6	PRAC1	RNASE7	UPK1A	FUT4	CXCL6	KRTAP4-12	GUCY2D	RARRES1
**epithelial cell of proximal tubule**	DMRTC1	DMRTC1B	LY6L	SLC7A13	SLC13A1	SLC17A3	SLC17A1	GLYAT	SLC22A12	AGXT2	SLC34A1	TMEM174	MCCD1	NAT8	SLC6A18	UGT3A1	UGT3A2	SLC22A6	MIOX	SLC5A10	ALDH8A1	SLC34A3	SLC5A12	GLYATL1	ERICH4	TMIGD1	ADRA2B	SLC6A19	ASPDH	SLC22A8	PAH	SLC47A1	SLC22A2	SLC18A1	MEP1A	SLC10A2	FAM151A	DAO	TINAG	PDZK1	F8A3	LRP2	ABCC2	CLTRN	GUCA2B	HAO2	SLC5A2	SLC28A1	RDH16	PRODH2
**epithelial cell of stomach**	CYP11B2	KRTAP9-9	KRTAP6-3	SPRR2B	SPRR2G	H3C11	KRT82	GALP	GHSR	TAAR6	FOXR2	IFNA16	TCP11X2	KRTAP29-1	KRTAP4-16	SBK3	GNRHR	ANKRD34C	CLDN18	BNIP5	PTF1A	IFNA2	G6PC1	FAM47C	KRTAP2-4	ALPI	NR1I3	CYP4F2	KRTAP4-2	FOXI2	CYP2C19	TBX22	UGT2B17	KRTAP27-1	KRT26	KRTAP19-4	HNF1A	EPHA8	TRIM50	MAGEB3	KRTAP20-2	NIPAL4	BRS3	TRIM42	KRTAP4-6	KRTAP4-7	KRTAP1-3	KRT222	CYP3A4	IYD
**epithelial cell of thymus**	TBATA	FGF21	DEFB103A	FAM163A	KRT24	LCE3B	TGM5	USP26	FOXN1	SERPINB12	AIRE	KRTAP10-10	TENT5D	GCG	HDGFL1	LACRT	GLP2R	RPTN	GSDMA	PRG3	SLC46A2	FEZF2	CSN2	H2AC7	IGFALS	GHRH	UNC93A	CHRM4	CELA2A	GPX5	IL36A	PAX1	SYNDIG1L	CRIP3	MYH8	MROH2A	CALCB	LRRC53	PRSS16	PROKR2	FRMD1	FRMPD3	GAL3ST2	LCE3C	FBXO47	FNDC7	KIF19	LCE3E	PRAMEF18	KLHL40
**epithelial cell of urethra**	NLRP10	PATE4	TGM4	DNASE1L2	LCE6A	HOXD12	LRRC31	GPR83	MSMB	SBSPON	PSCA	KRT4	HOXB13	HOXD13	SLC25A48	LRRC26	SPINK1	SMIM6	TIMP4	ABO	ADH1A	GSTA3	SPINK5	CATSPERD	ASIC5	KLHDC7A	SP8	BARX2	PGLYRP1	PPP1R1B	PAQR6	SLCO4C1	KCNE3	MMP7	PLEKHS1	HOXA13	AQP5	OSR1	KRTAP17-1	NRTN	MS4A5	CEACAM1	PRR15L	FOXA1	APOF	RASSF9	SLC22A1	TSPAN1	ETFBKMT	TCF15
**epithelial cell of uterus**	KRTAP22-2	LCE3C	SPRR2G	IL36A	GRIN3B	MYO1H	FAM166A	PLEKHS1	SPRR2B	SLC25A48	GDF1	STKLD1	VWA2	TMEM240	TOMM20L	MYH7B	TULP2	DCDC2B	SRPK3	KRTAP13-1	H3-5	SPRR2F	FGF20	PRICKLE3	PTX4	CAPN12	ANGEL1	KCNH6	PSPN	SPINT3	LGI4	NUTM1	LBP	TEX22	LCE3A	UPK3BL1	CERS3	S100A7A	TMCO6	PLA2G2E	OVGP1	IFNK	SMCO1	SLC5A11	CNTD1	ABHD1	STRA6	CCDC38	KANK3	ZSWIM2
**epithelial fate stem cell**	TBX22	LENEP	CCL27	LHFPL1	WFDC5	FRMD7	MADCAM1	LGALS7B	DSG1	NKX2-3	SPATS1	KCNH1	ADGRF4	SVOPL	LHX9	LCE1C	PITPNM3	NME9	LCE2C	KLKB1	IL20	KRT31	PRSS57	SERPINB2	LCE1A	CLEC2A	FAT4	KRT1	PITX2	ALPL	CCDC163	FNDC5	LCE2B	ILDR1	CNKSR2	SHANK1	NECTIN4	STON1	MCTP2	TRIM68	LRATD1	NDST2	ZFP30	CNTNAP1	MAMLD1	HCAR3	COL7A1	FAT2	HS3ST3B1	SLC24A4
**epn**	SPDYE8	TBC1D3F	HEPN1	OR13D1	GOLGA8T	OR4C45	OR13C2	USP17L7	CENPVL2	SPACA5B	OR2J2	USP17L4	OR4C13	OR1Q1	OR5AK2	FOXD4L4	USP17L3	USP17L1	OR52B2	OR2M5	OR11H12	HRNR	SLC47A2	HSFX1	ZNF474	OR2F2	OR13C9	KCNJ18	USP17L2	USP17L15	NUDT4B	OR2J3	OR12D3	TBC1D3H	OR1L4	TAS2R40	OR8K3	TAS2R39	CENPVL1	TBC1D3C	DEFB130A	OR13C5	OR13J1	OR1K1	CFC1B	OR5D16	DYNLT5	NUTM2D	CLEC19A	OR5C1
**erythroblast**	OR2W3	GYPE	RNF224	GYPB	SPTA1	RIPOR3	RHCE	HBM	TRIM10	KEL	SLC4A1	GYPA	RHAG	MARCHF8	HBG1	AHSP	EPB42	TSPO2	ALAS2	RHD	HEMGN	HBZ	KLF1	HBG2	SLFN14	SLC25A21	OR4N2	HBA1	SPTB	SLC22A16	THEGL	NXPE2	H1-5	HBA2	ERMAP	TFR2	CYP21A2	FAM180B	SEC14L4	GATA1	H2AC7	RGS6	ICAM4	FLACC1	TRIM72	ART4	H3C4	ANK1	FAM178B	H1-3
**erythroblastic island macrophage**	SLFN14	CD5L	TRIM10	CLEC4F	MARCO	TSPO2	NXPE2	GYPA	PAQR9	RHD	MAGEB16	RHAG	ALAS2	EPB42	HEMGN	ERMAP	SPTB	YPEL4	KLF1	ABCB4	TAL1	TMCC2	CTSE	SPTA1	PROKR1	TSPAN33	ICAM4	ALB	GATA1	E2F2	SLC43A1	ZFPM1	ERFE	FAM241A	ADGRE1	SOWAHA	NFE2	RANBP10	SPIC	ABCB10	ART4	PKLR	SLC38A5	CPOX	PLG	MAT1A	ANK1	SLC6A9	MMP27	CCL24
**erythrocyte**	ANKRD40CL	IFIT1B	HBD	ALAS2	HBM	HBB	AHSP	GYPB	TRIM10	LCE2C	LCE2B	SLC4A1	GYPA	DNAH10OS	LCE1A	LCE1D	HBA1	HBA2	TSPO2	CA1	KLF1	LCE2D	HBQ1	HEMGN	RHD	GYPE	SPTA1	TRIM58	EPB42	SRARP	PKLR	HBZ	NXPE2	RHAG	KEL	SLFN14	ZNF843	RHCE	GATA1	SLCO1C1	HBE1	HBG1	ERMAP	TACR3	TAAR5	REC114	PHOSPHO1	LCE5A	KPRP	SNCA
**erythroid lineage cell**	NXPE2	TSPO2	GYPA	TRIM10	RHD	KLF1	SLC4A1	HEMGN	KEL	SPTA1	PKLR	HBE1	RHAG	ALAS2	H2AC14	HBZ	ERMAP	AHSP	GYPB	GATA1	ICAM4	HBG1	H2BC7	SPTB	CTSE	HBM	HBG2	GYPE	MFSD2B	ABCB4	ASB17	SLC25A21	EPB42	CA1	GFI1B	MRGPRX2	EPOR	TAL1	HBA1	ADD2	TFR2	REC114	ANKLE1	HBA2	SLFN14	SLC22A9	MYLK3	SLC14A1	SLC43A1	HBD
**erythroid progenitor cell**	OR10Z1	IFNL4	OR2L8	KLF1	PKLR	GATA1	OR2AJ1	OR2L5	TSPO2	GNG14	AHSP	GYPE	MFSD2B	GFI1B	HBD	GYPB	RHAG	HBM	OR2T8	DCANP1	SPTA1	FAM178B	KEL	OR14A16	GYPA	OR1C1	CA1	OR4K13	PRSS50	TFR2	OR1N2	TRIM10	NXPE2	RHD	SUPT20HL1	HEMGN	KRTAP5-5	FOXD4L5	GDF3	SLC4A1	ZNF723	RHCE	PNMT	FAM124B	OR10A4	ICAM4	UBASH3A	MPL	NFE2	SLC14A1
**ews**	LIPI	IFNA17	IFNA10	MBD3L3	OR4E2	KRTAP4-11	TPTE2	FRG2B	KRTAP1-1	MBD3L4	NPY5R	SSX7	KRTAP4-9	CLEC2A	KRT40	CLEC12B	PAX7	KRTAP4-12	UGT3A2	KRTAP4-5	POU4F2	FRG2C	HCG22	RBM11	NPY1R	ADRA1D	KRTAP1-5	IFNW1	MBD3L5	OR4A16	HOXD13	OPN1SW	MAGED4	RGPD6	GABRR1	F2RL2	CCNA1	PRND	SLC17A8	LOXHD1	SLN	TAL2	EIF3CL	IFNA14	HMCN1	TDO2	BLID	SATL1	GRM2	SIM2
**excitatory neuron**	PNMA6F	NEUROD6	GABRA6	OR14I1	VXN	PROP1	IQCJ	TBR1	KLHL1	SEPTIN3	GRM4	SLC17A7	MPPED1	KIAA0319	SCN2A	CBLN3	PHYHIP	GLRA2	PNMA8C	NEUROD2	KIAA0408	LRRC7	SLC44A5	TAFA1	RAD21L1	NWD2	PLPPR5	FEZF2	GPR139	ARPP21	PAK5	HS3ST4	SATB2	MAGEC3	STUM	CSMD2	PABPC1L2B	GALR3	CRACD	MYT1L	NKAIN2	NKAIN3	LRRTM4	PCDH11Y	FBN3	GABRD	UNC5D	CAMKV	SEPTIN5	DSCAM
**exhausted t cell**	KLRC4	KIR2DL4	PDCD1	CXCR6	IFNG	FASLG	KIR2DS4	SIRPG	PVRIG	CD8A	IFNA17	GZMK	CXCL13	ZNF80	CRTAM	TIGIT	KLRK1	KLRC1	TNFRSF9	ZNF683	CTLA4	KLRC3	GZMH	GZMB	APOBEC3H	CD8B	PRF1	KIR3DL3	ICOS	SH2D1A	OR10G2	FCRL3	GZMA	LAG3	KLRC2	PPIAL4E	XCL2	THEMIS	CD2	CD3D	CD3G	NKG7	CCL5	HAVCR2	IL2RB	CCR8	OR10AG1	KLRD1	CXCR3	GPR174
**external epithelial cell of tympanic membrane**	CLDN22	FOXE1	UPK3BL1	CHST5	SLC16A11	SERPINB11	TREX2	KLHDC8A	PAX9	GABRP	DMRT3	HAMP	OSR1	KCNE2	PLET1	ABI3BP	UPK1B	STYK1	IL36RN	RARRES1	EPYC	LYPD2	FGFBP1	DMRTA2	CTXN3	BST1	CLDN23	LGR6	SMIM6	DMRT2	MUC16	CLIC5	SCNN1G	TRPV4	KRT4	SEMA3F	LOXL4	NTF4	SMIM5	KRT77	ATP6V1C2	UPK3B	TMEM238	TCF24	CDO1	ADGRG2	WNT9A	AQP5	SLC6A4	NPNT
**extraembryonic ectodermal cell**	ESX1	UTF1	PRAMEF12	MAGEB16	TMEM114	HAND1	EPOP	DNMT3L	ERAS	GTSF1L	FMR1NB	TEX13B	PSORS1C2	IRX4	GJB5	DUSP9	NUP62CL	ELF5	BMP8B	ASCL2	GJB3	WNT6	PRSS50	CITED1	TRIM6	ZFP42	EFNA3	CLDN6	NR0B1	DNMT3B	FABP3	CDX2	LRRN4	C1QTNF12	RIOX1	SLC38A4	DPPA4	PLAC1	BATF3	DUOXA2	POU3F1	TFAP2C	SFMBT2	SALL4	FAAP24	GLS2	KLHL13	PRSS41	NECTIN2	SELENOH
**extraembryonic endodermal cell**	PLA2G12B	GIMD1	SMLR1	AQP8	APOA4	S100G	CREB3L3	SPP2	LECT2	APOM	MBL2	RNF224	SLC13A4	AFP	APOB	IGSF23	SLC2A2	APOA5	ESX1	AMN	FMR1NB	CUBN	ARID3C	SLC39A5	SLC7A9	ERICH4	APOA2	APOA1	PDE6C	GLTPD2	MOGAT2	RBP4	PDZK1	EPOP	IHH	LGALS2	TTR	CITED1	APOC3	BCO1	TMEM82	CLIC6	SLC34A3	PNMA5	CLDN2	MAGEB16	CLDN6	NPL	PALM3	FGB
**extraembryonic mesodermal cell**	PLAC1	CDX4	HAND1	EVX1	TBX4	NKX1-2	FOXF1	KLF14	ETV2	SLC38A4	HOXD1	MOGAT2	CDX2	ASB12	CFC1	PRDM6	NUP62CL	MSX2	DGKK	TWIST2	APELA	GATA5	HOXD9	HOXA11	EPOP	SMTNL2	HOXB6	TBX20	ALX3	C1QTNF12	MSX1	AMOT	COX4I2	HOXC8	ASB4	SNAI1	RBPMS2	HOXB8	HAND2	HOXC9	FGF3	DLK1	CRABP1	TMEM88	LIN28A	PWWP3B	FIGNL2	SPOUT1	WNT2	PRRX2
**extravillous trophoblast**	ISM2	PRG2	LVRN	GPR78	XAGE2	AADACL3	HTRA4	POTEH	CSH1	NOTUM	GCM1	NLRP9	TAC3	AOC1	MDFIC2	PAPPA2	HLA-G	EBI3	XAGE3	CLDN19	LAIR2	MAGEA8	CRACDL	CSH2	SLC6A2	NOG	ASCL2	PSG2	TRIM64	ZNF593OS	ADAM12	GPR32	FLT1	GJA5	PAPPA	FSTL3	TRIM64B	VGLL1	SYT8	VGLL3	H2BC12	DIO2	CGB3	PAGE4	MMP11	WFDC10B	MFAP5	ATP1A4	CORO6	HAPLN3
**fast muscle cell**	CALML6	MYH2	MYH1	PCARE	MYF6	PPDPFL	MYBPC2	MYLK2	HJV	SMTNL1	AMPD1	MYOZ1	PRKAG3	PPP1R3A	CACNG1	NRAP	DHRS7C	DUSP29	KLHL40	LMOD2	UCP3	MYOT	ATP2A1	DUSP13	CASQ1	PERM1	KBTBD13	ASB10	MYBPC1	ABRA	MYADML2	HSPB3	PPP1R27	TRIM63	CAV3	MYPN	LBX1	XIRP2	CACNA1S	CCDC194	ATP1B4	SMCO1	PGPEP1L	VGLL2	FBXO40	MSTN	CA3	TRIM54	STRIT1	IP6K3
**fat body cell**	SOX14	SLC5A11	NAT1	CLC	INSC	GK2	RS1	BEST2	GSTT4	NXF2	SIX4	CPO	WDR62	ART1	RGN	CABP1	SPX	CRP	RGR	DMTN	PSD	SRL	TREH	HRG	HDC	RHO	PAK3	OXT	RAB19	RNASEK	HEPH	RINT1	SP7	SOD3	GRP	CSN3	SYP	GIP	MOS	CLN3	MED26	OCM	SPN	PDP1	ARX	ALG10	MED18	MYC	AGO1	LIMK1
**fat cell**	ADIPOQ	CIDEC	AGTR1	SLC36A2	FAM180B	CILP	FABP4	FAM162B	HAS1	SPAAR	APOC4	CIDEA	PLA2G2A	ABCA8	RETN	MRAP	MYOC	MXRA5	TNXB	NPFFR1	ADH1B	GSC	CFD	IL20	EGFL6	BDKRB1	PODN	WNT11	TRIM72	COQ8A	MEDAG	LRRN4CL	MMP23B	AVPR1A	PCK1	SLC1A7	THBS4	ASPG	HSPA6	SSC5D	HMCN2	DPT	ALG1L2	GJC2	ZNF843	VIT	WFDC12	SFRP4	CHRD	OPRD1
**fat stem cell**	ITLN1	PLA2G2A	IL20	PRG4	ADH1B	ITLN2	MSLN	UPK3B	CPB1	ANXA8L1	MT1M	ADIRF	HAS1	KLK11	FAM153B	ALOX15	OGN	TNFSF14	MT1A	CCL2	CALB2	TGM1	C1R	MGP	SLPI	INMT	TFPI2	CARNS1	CLDN15	AOX1	RARRES2	IL24	BNC1	MT2A	MT1E	AADACL2	TIMP1	NNMT	MEDAG	IGFL2	KRTAP13-4	KLK7	SERTM1	MST1	BCHE	HSD17B6	GALNT9	C1S	CCDC80	SERPING1
**female germ cell**	TP53TG3B	CT47A7	SPANXA2	ANHX	TP53TG3	OR10G9	OR2T27	OR10G8	OR4D5	DUX4	SPANXA1	CT47A9	DEFB124	OR3A1	CT47A10	OR2T2	OR1E1	ZNF728	CT47A12	ZNF705D	IFNL4	MPC1L	SPRR2F	CT47A1	MRGPRX3	CT47A5	TUBA3E	NLRP9	DPPA5	KHDC3L	USP17L15	FAM9A	NPIPA2	ZNF705B	TCL1B	H2BC1	OR10G7	CT47A3	NPFF	CT47A2	CGB8	LUZP4	TGM7	OR4F3	TP53TG3C	TUBB8	CT47A4	OR1J1	OR1N1	GPHA2
**fenestrated cell**	CD300LG	RTP3	ESM1	PLVAP	EMCN	PLSCR2	RSAD2	USHBP1	FAM167B	GPIHBP1	FAM110D	SOX18	SRMS	RAMP3	MMRN2	DLL4	RIPPLY3	ARHGEF15	MEOX1	TEK	ADGRL4	TIE1	SOX17	ROBO4	CLEC1A	FABP4	RASIP1	KDR	FLT4	CLEC14A	GPR4	TMEM204	PTPRB	TBXA2R	FLT1	GIMAP5	BCL6B	EXOC3L1	LIMS2	TCF15	CLDN15	CDH5	THRSP	UNC45B	PTPRU	BTNL9	ESAM	KANK3	THBD	ECSCR
**fetal cardiomyocyte**	WNT2	LCE3B	ARG1	MYBPC3	SPIN2A	MYL7	LRRC10	TECRL	TNNT2	TBX5	MYL2	GATA5	TNNI1	CSRP3	NKX2-5	MYOCD	MYL4	MYH7	POPDC2	FOXF1	ASB12	MYL3	HOXB1	NPPA	MYBPHL	LEFTY2	SMYD1	TRIM55	SFRP5	CASQ1	MYH6	TBX20	PRRX2	TNNC1	IFNA16	HAPLN1	LHFPL1	MYLK3	CFC1	PWWP3B	ALX1	ZBTB12	NPPB	PTGES3L	DUSP9	PRTG	FGF8	CLDN25	MYL1	CCDC141
**fetal hepatobiliary progenitor cell**	G6PC1	SLC22A25	CYP3A7	CYP2A7	SLCO1B7	SLCO1B1	SLC22A9	CYP4A11	CFHR5	CFHR4	ITIH1	UGT2B10	CYP2A6	AGXT	SLC17A2	SERPINA7	SERPINA4	CYP4A22	CCDC198	APCS	PLG	FCN2	CPB2	FGA	SERPINA11	HPR	SLCO1B3	UGT2B4	ANGPTL8	CCL16	CYP2C9	CFHR1	APOC3	SLC22A7	AKR1C4	APOA5	C8A	APOB	ITIH2	CFHR3	AHSG	LPA	CA5A	ALB	BAAT	ARSL	APOH	F11	ANGPTL3	HPX
**fibroadipose progenitor cell**	MYOC	CCL11	COL6A6	CILP	COL5A3	ANGPTL1	HMCN2	CLEC3B	FNDC1	HAS1	SSC5D	MEDAG	ASPN	DPT	LRRN4CL	RTL3	TNXB	CCL7	MEOX2	MFAP5	ARSI	FAP	LGI2	BMP5	ENTPD2	ADAMTS5	PODN	SMOC2	OGN	LRRC15	ABCA9	ADAMTS2	COL14A1	ECM2	DPEP1	SCARA5	IL17D	PRG4	SRPX2	EN1	COL15A1	IGSF10	PROKR1	ADGRD1	NAALAD2	FBLN7	THBS4	SHOX2	SVEP1	FBLN2
**fibroblast**	MMP27	DPT	MXRA5	FDCSP	USP17L1	LUM	CCL21	CCL11	APCDD1	MFRP	ADH1B	COL14A1	OR4C45	SCARA5	TWIST2	TNXB	THBS2	NPS	PODN	COL3A1	ASPN	ITGBL1	ABCA6	HTRA3	CCL19	C1R	CD248	ISLR	MEDAG	FAP	CCL13	CILP	COL6A3	TNFAIP6	COL1A2	APOD	FMOD	CTSK	SFRP2	TSPAN11	ADAMTS2	FBLN2	OLFML1	CFH	ABCA8	CLEC3B	OGN	COL15A1	COL6A5	CRISPLD2

**fibroblast of breast**	PRR33	IL1RL2	ADGRD1	MMP27	FNDC1	LVRN	RNASE10	TNXB	TMEM72	HCN4	IFNA16	GRIP2	SSC5D	SCARA5	CD248	KRT36	MEDAG	DPT	COL5A3	RTL1	CCL11	UBTFL1	PLA1A	PI16	BMP8A	HTRA3	DOCK10	TEX13B	COL14A1	ASB18	MMP3	LRRN4CL	VEGFD	GALNT16	C4B	CHRDL1	KLHL33	RTL3	ITGA11	ADAMTS15	PROKR1	WFDC12	LAMA2	ADAMTS2	DPEP1	TCF24	MRGPRF	IGSF10	ADAMTS5	PAMR1
**fibroblast of cardiac tissue**	TBX20	MFRP	DPT	C1QTNF5	CLEC3B	HTRA3	DPEP1	PI16	MFAP5	ENTPD2	CCN5	ANGPTL7	ADAMTSL2	TCF21	PLA2G2E	C1QTNF7	HSD3B2	HTRA4	TNXB	MEDAG	OGN	COL15A1	MEOX1	MYOC	SMOC2	PCSK6	CILP	GDF6	SCARA5	ADAMTS2	ASPN	CYGB	CCL19	LUM	PTGIS	PRELP	ISLR	CFH	PLA1A	ADAMTS5	PLAC9	ECM2	VIT	HEYL	SCN7A	C1QTNF2	CMKLR1	SRPX2	COL6A6	IL17D
**fibroblast of choroid plexus**	MXRA5	ALX4	TMEM200C	TTR	PMCH	SULT1E1	IL11RA	SMIM10L2B	TSPAN11	BPI	KIAA1755	FBN3	COL8A2	PABPC5	ADGRA2	ALX3	GDF7	LRRC17	TBX5	TWIST1	ZNF469	EGFL6	COL11A1	COL14A1	TMSB15A	NFATC4	DACT3	FAT4	COL3A1	EMILIN3	RAB40AL	KANK4	COL1A1	ZNF660	PRSS35	IGF2	CA9	TCEAL7	ZFHX4	PIEZO2	B4GALNT4	LHX9	PLPPR3	CILP2	RNF175	MAGEL2	PLAC9	PI15	GLT8D2	IGLON5
**fibroblast of connective tissue of prostate**	TGM4	CSF3	PATE4	COL6A5	APOD	IL1RL1	PTGS2	CXCL8	MRGPRG	FOXF1	TNFAIP6	EVX2	DPT	TWIST2	WNT2	PAGE4	PROK1	PTGDS	HOXD13	HNRNPCL3	MSMB	LUM	CRISPLD2	FBLN1	MGP	KLK3	MEDAG	CLEC3B	AKR1C1	ABCA8	HSD11B1	INMT	ADGRD1	BDKRB2	FZD10	SCARA5	TCF21	SPINK1	TSLP	MYOC	TNXB	IL13RA2	VIT	MFAP4	HOXA13	SERPING1	GEM	MT1A	CA3	SERPINF1
**fibroblast of dermis**	OR14J1	OR5AS1	KRTAP1-5	OR6C4	OR2T12	OR5AN1	FAM237B	ULBP3	CALHM5	OR2M3	H2AC17	LITAFD	H4C14	OR4K13	OR5A2	OR2A5	OR2AT4	H2AC14	OR7D4	OR2T7	FAM180A	OR8B8	MACIR	OR4K17	THSD8	IFNB1	PALM2AKAP2	OR7G2	KRTAP4-9	OR8H1	OR6Y1	OR4F17	CCN1	OR2M4	OR1M1	GARS1	OR52E4	HARS1	EOLA2	OR10G4	MMP3	HTD2	OR51L1	OR1D2	OR6B1	OR2G6	FGF5	FGF23	POTEJ	OR6K3
**fibroblast of lung**	SMIM41	GASK1A	CCN4	ABCA10	ABCA6	CCDC102B	ADH1B	COL6A6	PALM2AKAP2	MAMDC2	ABCA8	OR10J4	CEMIP	ABCA9	CACNA1C	LAMA2	SVEP1	ADAMTS8	PRDM6	TBX5	HMCN1	SNED1	ITGA8	SCN7A	ERFL	PIEZO2	FMO2	TEXS2	CPHXL	TBX4	CCBE1	DUXB	PLEKHH2	ZFPM2	FGF7	DEFB131B	INSYN2A	OR2F1	AWAT1	AOPEP	SHOC1	BNC2	OR2A14	A2M	SLIT3	COL21A1	CACNB2	DEPP1	PRKG1	SLIT2
**fibroblast of the aortic adventitia**	IL12B	CCL22	CLEC3B	CCL17	CCL11	MRGPRG	PI16	ZMYND15	INMT	DPEP1	DPT	TMEM100	VEGFD	PLA2G2E	SCARA5	MFAP5	MMP3	C4B	HTRA3	TNXB	PCOLCE2	PI15	RNASE10	ADGRD1	PLAC9	MMP25	TSPAN11	OSR1	ENTPD2	FNDC1	CCL8	PODN	ADAMTS2	GDF10	SFTPC	NUDT17	CCL19	CACNA1S	CYGB	SCN7A	CD248	ISM1	OGN	CCR7	MUSTN1	CMKLR1	C1QTNF7	MEOX2	CXCL13	THBD
**fibroblast of tunica adventitia of artery**	ADH1B	FAM237B	CCN5	OR2C1	SFRP4	HAS1	CHRDL1	C1R	MYOC	SERPINA3	MMP19	PODN	SCARA5	SPDYE2B	ABCA8	MFAP4	F10	ADAMTS8	NLRP2B	DEPP1	C1S	PI16	FIBIN	PRELP	MT1A	IL6	MEDAG	CH25H	MT1M	ABCA6	A2M	SFRP2	LUM	ANGPTL5	C1QTNF3	PDGFRL	PLAC9	OLFML1	AADACL2	FGF7	SCN7A	INMT	DCN	RARRES1	CRISPLD2	PLA2G5	S1PR2	CES1	FMO2	SPDYE18
**fibrochondrocyte**	APCDD1L	ANGPTL5	CHI3L2	COMP	BDKRB1	CXCL5	CEMIP	TBC1D3L	MEDAG	PTGFR	PRG4	BDKRB2	IL26	HTR2A	GALNT15	CSF3	MXRA5	GDF6	SHISAL1	NDP	MMP10	IL36RN	LIF	STC1	PF4V1	CRTAC1	IL36B	MMP13	NDUFA4L2	FMOD	CBLN3	TREM1	HHIPL2	FAM180A	SUPT20HL2	CCL20	C1R	TNFRSF11B	IL24	LRRC15	FGF7	TNC	SSC5D	C1QTNF1	SUPT20HL1	AKR1C2	TBX15	INHBA	NOS2	KCNE4
**fibrochondrocyte progenitor cell**	CCL13	OR1L8	HCG22	CALHM5	CSF2	KCNE4	LIF	TNFSF15	CXCL5	MEDAG	TREM1	PRG4	FGF7	ANGPTL5	APLN	COMP	CCL8	CCL20	NDUFA4L2	BMP8B	STC1	CCN5	BDKRB1	PTGIR	C1R	C2CD4A	CHI3L2	AOC3	CSF3	C1QTNF1	MSC	IL24	TNC	APCDD1L	COL5A3	CBLN3	TEX54	NR4A3	ADAMTS2	NOD2	BDKRB2	SHISAL1	HTR2A	GPR176	NOTCH3	MAP3K20	SGCA	IFI44L	PTX3	MCAM
**follicular b cell**	FCER2	FCRL2	HLA-DOB	MS4A1	NIBAN3	TCL1A	TNFRSF13C	TLR10	CXCR5	OR2T10	VPREB3	CD22	BLK	CD79A	FCRL1	BANK1	OR2T11	PAX5	OR56B1	P2RX5	CD19	FCRLA	HLA-DQA1	HLA-DQA2	CD79B	CLEC17A	FCRL3	FAM177B	HLA-DOA	FCRL5	HLA-DQB1	FCMR	HLA-DMB	SHISAL2A	GAPT	FCRL4	MARCHF1	ADAM28	BTLA	CHI3L2	LY9	CCR7	CALHM6	PNOC	OR2G6	SPIB	LTB	P2RY10	TASL	HLA-DMA
**follicular cell of ovary**	NXF2	RS1	CLC	SOX14	BEST2	CABP1	ART1	PSD	RGN	NAT1	SIX4	CRP	OXT	RGR	SRL	SPX	GK2	HRG	CPO	OCM	HDC	ALG10	WDR62	TREH	SYP	GSTT4	PDP1	GRP	RNASEK	HEPH	NXF3	MED26	CSN3	TTK	INSC	MED18	SOD3	BVES	DPH5	GSTO2	ARX	MYC	AGO1	FKBP14	COL4A1	SLC5A11	CIC	GIP	DYRK3	RAB19
**forebrain**	FOXD4	ERAS	HESX1	LHX5	FEZF1	RAX	FOXB1	WNT8B	ERMN	IRS4	OTX2	HES3	TAL2	DNMT3B	SIX3	FOXR2	EPOP	SHISA2	POU3F1	VSTM2B	LMO1	CRYGB	LPAR4	LIN28A	OTX1	JPT2	ZBTB12	FIGNL2	RIOX1	FEZF2	NPTX2	MOGAT2	ZIC3	TLCD3B	SALL4	CCDC177	SLC7A3	ZSCAN10	SELENOH	HPF1	LHX2	RCOR2	WNT1	SMTNL2	PRSS41	PRELID3A	PRTG	SALL3	AUNIP	GON7
**foregut**	APELA	TRH	NKX2-6	DGKK	PYY	CER1	NKX2-3	CFC1	CLDN6	ERAS	GSC2	NKX2-5	FOXD4	APOA5	SP5	FGF16	PAX9	NECTIN2	LIN28A	ZBTB12	DUSP9	FOXA2	KLK14	FIGNL2	AARD	MOGAT2	IRX1	EPOP	SMIM10L2A	WDR86	PRTG	SOX17	TAL2	SALL4	CLDN9	FOXR2	SELENOH	ISL1	TEDC1	PALM3	FGF8	JPT2	EEF1AKMT2	BEX1	SHH	GON7	TEAD2	HESX1	HPF1	SPEG
**foveolar cell of stomach**	GKN1	GKN2	GAST	MUCL3	MUC5AC	CLDN18	ANXA10	MUC17	REG4	TFF2	TMPRSS15	PGC	ARL14	AKR7A3	TFF1	CYP2C18	MSMB	PSAPL1	SULT1C3	TMEM238L	INAVA	REG1A	OTOP3	PHGR1	MLN	NKX6-3	BTNL8	IGLL5	BTNL3	VSIG2	FABP2	TM4SF20	SMIM31	MUC6	CTSE	TRIM31	CAPN8	RBP2	KRT20	LIPF	SULT1C2	VSIG1	SMIM24	SMIM32	PRAP1	DEFB131A	OTC	TM4SF5	CYP2D6	SPINK1
**fraction a pre-pro b cell**	IGLL1	VPREB1	VPREB3	LCN6	IFNA2	IFNA8	NIBAN3	UMODL1	OR2L8	PRSS57	PAX5	RGL4	RAG2	RAG1	KRTAP4-9	INKA1	HMHB1	HBG2	CD79B	KRTAP4-12	DNTT	CD79A	CCDC81	TSGA10IP	ENAM	NCR2	LTA	CALHM6	BLK	AHSP	ARMH1	MZB1	CETP	JCHAIN	H2AC12	KLRF2	MS4A1	HBM	CD22	HBA1	HBA2	ATP5MGL	ADA	LAT2	AIF1	FOXD4L3	LST1	LTB	PPP2R3B	SPINK2
**ftc133**	TMEM233	PSG1	SSX1	CD70	SHISAL1	IGFN1	BAALC	MAGEA10	DGKI	CSAG1	CPA4	GNAT3	CXCL6	LAMP5	MAGEA11	SLC38A5	MAGEC2	INHBA	ZP1	FGF5	ARL9	CCL26	WFDC3	ANKRD1	MAGEA1	BIRC7	FAM133A	PPP1R3G	TRIM55	MAGEA12	APCDD1L	PAGE5	MXRA5	HS3ST3A1	MAGEA3	G0S2	HRCT1	STC2	FAM180A	VCX3B	PAGE2	CXCL1	S100A5	S100A3	TAS2R10	EVA1A	CDKN2A	SLC35G5	AHNAK2	NKX2-8
**gabaergic neuron**	PROP1	MEPE	IFNA2	IFNA7	TAS1R2	SKOR1	IFNL3	KRTAP4-3	KRTAP12-1	CORIN	LBX1	INSYN1	ALK	GALR3	DUX4	GJA10	ASCL3	HAO1	IL9	SLC32A1	NEUROG2	NHLH2	SSTR4	SCRT1	CPO	GSX1	LHX5	GAD1	CASTOR2	EYS	OR4P4	PTF1A	KRTAP8-1	CRYGA	GPR26	MIP	DYTN	KRT39	SLC6A7	HRG	AANAT	IFNA5	GRP	OTP	IGFBPL1	SYN1	DAO	GPR33	KRTAP11-1	PRAMEF17
**gamma-delta t cell**	KIR3DL2	KIR3DL3	KIR3DL1	KIR2DL3	KIR2DL4	RTP5	KLRG1	FGFBP2	OR4E1	KLRC1	OR5AN1	ZNF683	GZMH	KLRB1	GNLY	GZMM	NCR3	OR5B2	FASLG	FGF23	PRF1	S1PR5	KLRD1	FCRL6	GZMK	XCL2	TBX21	CST7	GZMA	NKG7	FCRL3	GZMB	KLRC4	KIR2DL1	CD3E	P2RY8	CD247	CTSW	OR5AU1	OR4D1	CCL5	IFNG	FCGR3A	OR4K13	SAMD3	OR10H1	CD7	WNT1	CD160	SLFN12L
**ganglion interneuron**	CHRNB3	CHRM5	OR2L13	TFAP2D	GPR149	POU6F2	CHRNA6	CRYAA	TRPC7	SEPTIN3	DCDC1	KLHL1	VWC2L	EPHA6	SLC17A6	CNTNAP5	POU4F2	ZNF804B	CPNE4	POU4F1	CRYGC	PVALEF	CA10	FSTL4	TMEM271	CACNG5	GLYATL2	OPN4	DACH2	CALHM4	SEPTIN5	MSTN	IL1RAPL2	PNMA6F	DSCAM	MDGA2	LRFN5	MARCHF11	GALNTL6	P2RX3	CHRNA3	LINGO2	KCNIP4	ZNF804A	LHFPL3	GPR156	NTNG1	HS3ST5	CACNA1B	NRG3
**gbm**	HEPN1	PMP2	GFAP	VXN	SUPT20HL1	OR4N2	CFHR4	BCAN	GOLGA6C	USP17L7	USP17L1	CENPVL1	USP17L4	OLIG1	OLIG2	ZNF177	ZDHHC22	TNR	KLRC3	OR5I1	SCRG1	GPR17	USP17L3	NEU4	TAS2R39	FOXD4L4	ASIC4	TRIM48	FOXD4L3	USP17L10	OR2C1	DEFB109B	KLRC4	TP53TG3B	GSX1	USP17L8	USP17L2	MSTN	SPACA5B	KLRC2	PTPRZ1	CRB1	RFX4	DAZ2	PRLHR	GOLGA8T	ASCL1	MT3	BAALC	SCD5
**germ cell**	KDM4F	MAGEB17	STRA8	TRIM49D1	TSPY8	NXF2B	TEX13C	NANOS2	TEX12	CT47A6	CLEC4M	TBX22	FOXE3	SSX2	DAZL	TUBA3D	RHOXF1	OR8S1	TEX101	H2BW1	SLC25A31	RFPL3	PNMA5	FATE1	PASD1	DPEP3	DCAF4L2	TSPYL6	LPA	KRTAP5-7	TKTL1	FKBP6	LUZP4	CCDC172	TFDP3	SMC1B	GLYATL1B	SOHLH1	IFNL4	DCAF8L1	KRT26	MAGEB1	OR8G5	FTHL17	ZNF723	TSPY2	GAGE12H	OR7E24	CT47B1	ESX1
**germinal center b cell**	H2BC17	H3C7	H3C12	OR5B21	H2BC14	H4C13	H2BC13	AICDA	TASL	H2BC10	H2AC13	SERPINA9	H1-5	MS4A1	H4C1	H2AC7	RGS13	FAM72C	H4C2	H2BC9	TCL1A	OR13A1	SLC5A2	TPSAB1	H1-6	CXCR5	H1-3	H2AC4	FCRLA	BFSP2	CD79A	CD19	GLYATL1B	ADCYAP1	GJE1	SLC22A12	GPR18	GCSAM	H3C4	NLRP4	CD79B	TMEM174	PRAMEF25	S100G	H4C6	CFAP20DC	CD22	H2AZ1	TMEM265	RUBCNL
**glandular epithelial cell**	SEMG2	BPIFB3	BPIFB2	MUC7	ODAM	PIP	HTN3	TCN1	FDCSP	HTN1	PRB3	DMBT1	PRB2	PRB1	PRB4	SEMG1	ELOA2	MSMB	AZGP1	LTF	SMR3A	LPO	LACRT	CXCL17	PIGR	SCGB1D1	SLC5A8	CLDN22	BPIFB4	FAM3D	SCGB3A1	SMR3B	LHCGR	CRISP3	AQP5	CCL28	LRRC26	IL19	SLC13A2	ELF5	GJC3	SCGB2A2	LCN15	SLC6A14	ELAPOR1	PLEKHS1	RARRES1	TBPL2	GLYATL2	PABPC3
**glial cell**	NPVF	SMIM43	CDH19	LGI4	DIPK1C	DHH	ATP1B4	PRIMA1	MPZ	GBX1	GFRA3	KRTAP10-11	COL20A1	MYOT	CRYAA	SFRP5	CMTM5	SCN7A	PLP1	EGFL8	LBHD2	GPR37L1	TMPRSS5	RASGEF1C	GSX1	KRTAP10-10	GSX2	GFAP	LRRTM1	SEMA3B	FABP7	MATN4	KCNA1	TBC1D3I	COL28A1	ASPA	CATSPERZ	FOXD3	ART3	TMEM235	PAQR6	MRGPRX4	SOX10	TSPAN11	RLBP1	KCNA6	SP7	ABCA8	TREH	H2BC3
**glomerular capillary endothelial cell**	IL36B	SLC22A24	PRSS51	PTH2R	SLC5A12	SPANXA1	BTNL8	CYP4A11	EMCN	SELE	KLRF2	PSKH2	SHANK3	CHRM3	SOST	RXFP1	AGXT2	SLC17A1	SSX5	NRG3	CUBN	DRD3	LDB2	CRHBP	ITGA8	SLC13A1	MMP26	SNED1	SLC5A4	PTPRB	ZNF366	SLC2A9	GPC5	SLC28A1	RNF212B	TEK	HECW2	GNA14	OR9Q1	FCAMR	UGT2B7	SLC13A3	ADGRF5	BTNL9	PTPRM	SLC26A3	FLT1	ZNF804B	SLC22A12	PRODH2
**glomerular endothelial cell**	SOST	EXOC3L2	CD300LG	NPHS2	PI16	EMCN	TCIM	MMRN2	BSX	SOX17	DKK2	EHD3	FAM167B	SEMA3G	SRMS	CDH5	KDR	CYP4B1	RAMP3	USHBP1	TIE1	EXOC3L4	BCL6B	RASIP1	ARHGEF15	GATA5	PLAT	PTPRB	PLPP1	PHOSPHO1	ADGRL4	ESAM	CLEC14A	DHH	TMEM252	KANK3	UNC45B	TEK	SAPCD1	PECAM1	LIMS2	RIPPLY3	GNGT2	NOSTRIN	DLL4	FABP4	ENG	ADGRF5	BMP6	ENDOU
**glomerular visceral epithelial cell**	NPHS2	NPHS1	SOST	AGRP	GBX1	SEMA3G	TDRD5	PTPRO	WT1	FRMD1	COL4A3	CLIC5	DDN	R3HDML	OR4E2	RFPL1	PTPRQ	LMX1B	ARHGAP28	CST5	ENPEP	TMEM150C	AADACL4	C1QTNF7	COL4A4	MAGI2	DACH1	TCF21	NTNG2	THSD7A	ASB15	BSPH1	MERTK	PODXL	ST6GALNAC3	ZBTB7C	PARD3B	SHISA3	SYNPO	CRB2	SEMA3E	SNCAIP	OR4C3	WIPF3	AGXT2	LCTL	SNX31	PLCE1	PLA2R1	P3H2
**glutamatergic neuron**	OTP	ALK	BARHL1	CORIN	CPO	CHAT	LMX1A	NEUROD6	HRG	GRP	RAB26	CRP	HCRT	OR4P4	SYN1	CLC	NYAP1	MPPED1	RBFOX1	SYT4	LPA	ATOH1	TBR1	MIP	TRIM67	HEPH	DAO	SCRT1	PROC	EYS	DCX	SYT7	NWD2	FEZF1	NHLH2	DDC	STMN4	OCM	UNC79	DYRK3	ART1	LHX5	NEUROD2	STUM	ISLR2	NAT1	CELF3	CRMP1	PMCH	TTC9B
**goblet cell**	RETNLB	TBX10	P2RY4	CLCA1	TPSG1	ZG16	CYP2F1	LIPF	BNIP5	OR51F2	ITLN1	BPIFB2	OR9Q2	MUC2	URAD	SPINK4	ATOH1	BPIFB1	FCGBP	REG4	FAM3D	OR5B12	MSMB	B3GNT6	REP15	ST6GALNAC1	ENTPD8	CYP2A13	FER1L6	MUC5AC	CAPN9	OR10AC1	MUC6	SERPINB4	ERN2	SLC6A14	KLK15	IL17C	SCGB1A1	TFF3	FABP2	GIMD1	LYPD8	MUC5B	CXCL17	VMO1	SMIM31	BTNL3	SERPINB3	KLK12
**gonadtroph**	FSHB	BPIFB6	GNRHR	LHB	BPIFB3	PRLHR	TCF24	ECEL1	CGA	BPIFB4	FCN2	CST8	NR5A1	CHRNA2	NPPC	DGKK	FOXL2NB	IRS4	PRL	SCG2	FEV	INHBB	CST11	KRTAP12-1	CLDN9	CDK5R2	C1QL2	LRRN2	LHX3	INHA	GNG8	DPP10	MARCHF11	KHDRBS2	TAC4	EYA1	GUCY2F	TSHB	CHGA	NR0B1	ASB11	PTPRN	TGFBR3L	CIB3	RESP18	ST18	PCSK1	SBK2	NFASC	ERFE
**granule cell**	ZNF804B	KCNK9	CFAP299	XKR7	UNC13C	SEPTIN3	STUM	FREM3	OR2L13	CDH7	THSD7B	FSTL4	CBLN1	RYR3	GRM8	GRIK2	HS3ST5	CNTN5	NKAIN3	RELN	GLRA2	CNTNAP5	KCND2	BARHL1	NDST3	RASGEF1C	HS3ST4	INSYN2A	KCNH7	MYRIP	IQCJ	DCDC1	ZNF804A	AKAIN1	ZNF385B	KLHL29	CDH18	SEZ6L	IL1RAPL1	ZNF536	SLC26A7	CDH20	KCNJ6	DSCAML1	DOK6	AFF2	DSCAM	NRG3	GPC5	TMEM271
**granulocyte**	DEFA1	MAS1L	DEFA1B	OR4X2	DEFA3	CEACAM8	DEFA4	OR6K3	MMP8	KDM4E	RHEX	BPI	OR6K6	GCSAML	CD177	CEBPE	TPSAB1	SSX7	CD200R1L	OR2AG2	CAMP	MMP9	FPR2	TPSD1	TPSB2	OR9A4	CCR3	MLNR	PADI4	MS4A3	OR56B4	MS4A2	CD300LD	GPR151	RNASE3	LTF	CLEC4D	DCAF8L2	NUTM2B	ASPRV1	USP17L18	CHI3L1	CXCR2	PGLYRP1	IL36G	SIGLEC8	HK3	LILRB4	LILRA6	PTGDR2
**granulocyte monocyte progenitor cell**	H1-6	RNASE3	DEFA4	MS4A3	AZU1	RNASE2	CTSG	KLK14	CEACAM8	OR2L3	PRTN3	ELANE	OR10G2	DEFA3	NPAP1L	ELOA2	BPI	MPO	PRSS57	GOLGA8S	OR2AG1	OR10A5	CEACAM4	H4C6	TASL	RAB44	SERPINB10	H2BC17	MMP8	DCANP1	PEAK3	H3C11	CLEC12A	ATP8B4	HK3	SUPT20HL1	STYXL2	OR2L8	H2AC16	ADGRG3	FCGR3B	SPDYE8	SLC22A16	CEBPE	CLEC5A	CSF3R	SPDYE2B	GOLGA8T	NUTM2F	CYP2C9
**granulocytopoietic cell**	CD177	CEBPE	CAMP	MS4A3	LTF	CLEC5A	IZUMO1R	PGLYRP1	CHI3L1	ELANE	HK3	FFAR2	PRAM1	LRG1	ORM1	GPR84	S100A8	CHIT1	CLEC12A	TRIM10	PADI4	TSPO2	FPR2	RAB44	TREML2	ITGAM	EBI3	IGSF6	MOGAT2	CD300LF	PRTN3	NFAM1	DOK3	LCN2	HSD11B1	FOXD4	LILRB3	NCF4	GPR141	NXPE2	ALOX5	CYBB	PRSS57	MMP25	LBP	NFE2	PILRA	S100A9	PRG3	NCF1
**granulosa cell**	GSTA5	EXOC1L	NR5A1	MEPE	AMHR2	SPRR2F	AMH	SOST	SIGLEC11	CRYGB	TNFRSF6B	CST8	GADL1	GLB1L3	SYT10	NDP	WT1	FAM221B	HOXD4	PLA2G1B	METTL24	TRPC7	CRHBP	FOXL2	INHA	EMILIN3	RIMS4	DCAF12L2	EMX2	KCTD14	SHBG	KIAA1210	PLAAT3	CHRM4	LZTS1	RAD51AP2	NPAP1	GUCY1A1	CLEC4M	GAGE10	INSYN1	PNLIPRP2	HOXD9	LHX9	GSTA1	GREB1	PRSS35	FSHR	SLC24A5	KISS1
**group 1 innate lymphoid cell**	KIR2DL4	XCL2	SH2D1B	XCL1	KIR3DL1	KLRC1	KLRF1	KLRC3	OR4D1	KLRF2	KLRC4	TNFSF11	GNLY	CCR8	SIGLEC7	PTGDR	KIR2DL1	IFNL1	KLRB1	IL18RAP	IL2RB	NCR1	PRF1	NCR2	SH2D2A	TMPRSS11E	NCR3	CTSW	KIR3DL2	KLRD1	CD160	MMP25	CXCR6	TNFRSF4	FASLG	CD7	KIR2DL3	KLRD1	TNFRSF18	IL17A	GZMK	GZMB	GPR174	ADGRG5	GPR68	CD96	TXK	CST7	ITK	NKG7
**group 2 innate lymphoid cell**	IL13	IL5	CCL1	IL17A	OR2AE1	IL9R	PTGDR2	IL22	KLRB1	CALCA	OR4D1	CSF2	CCR4	GPR55	CCR8	IL4	KLRC3	XCL2	CXCR6	ICOS	PRSS38	NCR3	CCR9	XCL1	IL2RA	P2RY10	CTLA4	CYSLTR2	PTGDR	KIR2DL4	OR3A3	TNFSF11	ITK	KLRG1	CD200R1	IL7R	SCART1	FXYD7	TNFRSF18	TNFRSF4	GNLY	GPR132	IL18RAP	SH2D1B	CCR6	CTSW	IL17F	ITGB3	IL23R	KLRC1
**group 3 innate lymphoid cell**	KLRF2	NCR2	IL23R	OR3A1	OR4F6	IL17A	TMIGD2	OR10A5	CCDC194	KRT86	IL17F	XCL2	TNFSF11	CSF2	IL26	OR5F1	XCL1	KLRB1	OR4L1	PRAMEF1	SERPINA11	OR2T8	IL4I1	SIGLEC7	SPATA31A1	OR2T5	CCR6	LTA	ICOS	PTGDR	OR2V2	NCR3	LINGO4	TAS2R41	OR1M1	NMUR1	IFNL1	SOST	KLRC1	TNFRSF25	TNFSF14	IL9R	NUTM2B	CHAD	IL2RA	IL2	CCR4	SLFN12L	RORC	TAAR6
**growth plate cartilage chondrocyte**	MATN1	S100A7A	IL17B	TCHHL1	KLK9	PRKG2	SCRG1	SUSD5	GRAMD2A	CAPN6	DMP1	IFITM5	SLC6A12	CCN6	PRSS50	COL11A2	COL9A3	FMOD	SLC13A5	RTL3	OMD	MUSTN1	GDF10	FBLN7	SMPD3	SLURP1	RXFP3	NRK	PTGS2	PCOLCE2	P3H2	GJB4	SLC7A3	FIBIN	GFPT2	PRELP	TRPV4	S100A3	SMOC2	COLGALT2	CLDN20	CNNM4	KRT16	ADM2	KLF14	KRT73	PDGFRL	SP7	ANXA8	HSPB6
**gut endothelial cell**	PTCHD3	DIPK2B	OR6J1	CCL23	ZSCAN4	CCL14	DNAJC5G	C2CD4C	WNT9B	DOC2B	B3GALT9	NKX2-3	NPIPB8	CLEC4M	LCN6	EDDM13	ZNF792	HTR3C	LRRC70	ZNF727	GALR3	AVPR2	CCL21	MMRN1	LHX6	IL1RL1	OR6C75	TNFSF18	SELP	FAM110D	PKHD1L1	TMEM273	LRCOL1	TPO	GRAPL	CCM2L	GOLGA8M	GJC2	FZD10	SHE	SMCO3	CALHM5	SOX18	ROBO4	OXGR1	MYCT1	SHANK3	GIMAP8	PGM5	GASK1B
**h9**	NANOGP8	HHLA1	OR2M3	OR2C3	ZNF729	OR5A2	OR14J1	OR6Y1	OR6C70	SSX4	OR2M4	OR5AS1	OR2T12	OR5AN1	ZNF723	OR2AT4	ZNF705D	SEMG1	DPPA5	H3C11	CENPVL2	OR1D2	OR5A1	OR52K1	OR10G4	ALPG	SSX4B	KHDC1L	KHDC3L	OR6C4	H2AC17	ZNF878	DNMT3L	OR56A1	THSD8	OR52E4	FGF4	OR6C2	EZHIP	NLRP7	OR1C1	OR4F17	ABHD12B	OR56A4	OR14A16	ZNF728	OR5B2	OR4K17	HTD2	FAM151A
**hair cell progenitor cell**	OTOL1	OTOG	TECTA	ATOH1	ARID3C	PXT1	TMPRSS11F	KCNA4	LOXHD1	KCNA10	CDH19	POU4F3	SBK3	CCER2	CDCP2	LRRC3C	OTOF	KCNA6	GRID2IP	PTPRQ	USH2A	RNF225	DAGLA	GJC3	GUCY2F	EFCAB8	GSC2	KCNA1	H2BC1	GPR21	PRAG1	OTOGL	BARHL1	PCDH20	OMP	NGFR	MFRP	NKD2	NTN5	CIB3	SPACA6	RASD2	SOX10	ITGA7	LGR6	FOXD3	LCE6A	LGI4	SPRED3	CEP295NL
**hair follicle cell**	KRT77	KRT79	PGLYRP4	FAM25C	LY6G6C	CASP14	OR2L8	KLK7	AVP	KPRP	SPRR1A	PSAPL1	PLA2G2F	LGALS7	IL36RN	KRT1	KRT71	PRSS57	TRIM74	SBSN	MT4	IGLL1	THEM5	GJB4	CHIT1	SPINK2	HTR1F	CRHBP	AZU1	CAPNS2	CLCA2	KRT78	DSC3	KRT35	LY6D	CFAP57	ADGRF4	CDSN	GOLGA8Q	FAM83C	BPIFC	RNASE3	BARX2	IVL	CYTL1	PKP1	GCSAML	WFDC5	KRT25	SCEL
**hair follicular keratinocyte**	LIPM	KRT24	PSAPL1	KRTDAP	MT4	TGM5	CHIT1	PGLYRP3	CLCA2	PKP1	CLCA4	CAPNS2	WNT3	VWA2	PLA2G2F	FAT2	CLCA1	PGLYRP4	CDCC27	FAM25C	POF1B	KRT77	SERPINB10	ADGRF4	FOXN1	KRT1	TNS4	CASP14	EPPK1	SBSN	RAPGEFL1	PLCH2	SCEL	TNK1	PLA2G4F	IL22RA1	LARGE2	CDC42BPG	FCGBP	TFAP2B	HS3ST6	KRT32	IL20RB	PI15	FAM83C	GLI1	KRT79	MEGF6	KRT25	POU2F3
**hcc**	CFHR5	CFHR4	CFHR1	CFHR2	PLGLB2	APCS	UGT2B4	CFHR3	C4BPA	AGXT	CYP2C8	ORM2	AKR1C4	CYP2C9	CYP4A22	UGT2B11	FGA	SERPINA7	ITIH1	C8A	SAA4	HPR	UGT2B10	ORM1	CPB2	SLCO1B1	HPX	CCL16	C8B	C4A	ANGPTL3	CYP7A1	ITIH3	SERPINC1	KNG1	UGT2B15	APOH	SERPINA6	APOC2	APOB	CTAGE4	CTAGE8	FGG	CPN2	ANGPTL8	FGL1	BAAT	SERPINA10	FGB	APOC3
**hcc38**	MMP20	KLK5	HOXC12	EN1	NPW	GPRIN2	ZSCAN4	RNF212	OXCT2	PRAC2	MMP7	DSG1	GABRQ	COL9A3	MESP1	DSG3	ARL9	KCNK15	AARD	FOXL2	KLRG2	GAL	KRT81	ALX3	HMSD	TLX3	HOXC13	ERVMER34-1	HORMAD1	ENPP6	TGFBR3L	ANPEP	HOXC11	SIX2	LY6K	DEFB126	KLK6	TESMIN	F12	HPDL	EFEMP1	KLK10	ZNF695	NAGS	SLC6A11	LYPD8	FZD10	CFAP251	ZNF579	TRIM43
**hcc366**	FAM9C	HMSD	FAM133A	MMP1	TDRD12	VSTM1	CARD18	MAGEA4	DCAF4L2	MAGEA10	KRT34	CSAG1	FGF5	IFNE	IL13RA2	GPRIN2	GOLGA8H	SERPINB2	MAGEA12	SIGLEC15	KIAA1549L	SOHLH2	CCDC74B	SPIN4	FGF2	TLCD5	BLID	GTSF1	KHDC1L	CATSPER1	G0S2	TP53TG3D	MAGEA6	IL11	TMEM92	ZNF488	CCN6	MARCHF4	POPDC3	ARHGAP22	MAGEA3	ZNF667	VCX3A	CCL20	LHX1	LAMC2	HERC5	DKK1	OTOA	IL18
**hcc827**	DEFB4B	DEFB4A	H4C12	XAGE1B	NTAQ1	XAGE1A	H2BC9	H1-3	POTEI	CCN1	SFTA2	H2AB3	H3C7	PPIAL4H	PLAAT3	H2AC20	CT83	ANTKMT	FAM174C	PPIAL4E	PABIR1	KARS1	H2AC14	VARS1	SETSIP	NIBAN2	MTLN	H2BC11	H2AZ2	EOLA2	H2BC8	VXN	SNORC	PSME3IP1	COA8	ATP5MJ	SPRING1	GARS1	DEFB103B	CEP20	H1-0	ANP32D	ARSL	MTARC1	H2AC4	PRXL2C	EOLA1	RUSF1	H2AZ1	H1-5
**hcc1171**	TLX1	FAM133A	PLCZ1	PNLDC1	IFNL3	SPANXD	FAM83A	LCE5A	WFDC10B	RNF212	PLAU	CYP24A1	ESM1	FERMT1	NT5E	CD70	KLHL35	G0S2	FCRLB	PRSS3	SPOCK1	NGEF	ARHGAP22	SLC49A3	TRIM55	MELTF	NKX3-2	CBLN1	HRCT1	ADORA1	CATSPER1	NPB	TMEM156	ADAMTS6	PTHLH	COL13A1	IL37	ITGA2	SEMA7A	MDGA1	MT1M	PODXL	LAMB3	PLEK2	ANTXR2	S100A3	LCE1E	WFDC3	VEGFC	TNNT1
**hcc1359**	PASD1	TDRD12	PRDM13	FAM9C	HMSD	MAGEA4	RFLNA	MEIOB	CCDC74B	AK5	ZNF781	HBQ1	CCDC74A	CSAG1	MAGEA12	MAGEA1	CFAP251	MAGEA3	HORMAD1	FOXD1	VSTM1	FTCD	HES7	ANKRD62	PLAAT1	RNF212	BEST3	XKR9	MAGEA10	MARCHF4	HOXC8	CPNE7	YBX2	SOHLH2	GTSF1	FAM133A	RGS20	NEURL1	FBXO17	VGF	NKX3-2	PRAME	HOXB4	MAGEA6	DLL3	G0S2	C1QL4	CALB2	FAM72B	FAM50B
**hcc1419**	HOXC12	BCAS1	SPTSSB	DHRS2	VIPR1	EPO	BICDL2	CCL22	TINCR	CEACAM6	SPDEF	ALDH3B2	PRR15L	HOXC13	LRATD2	ERBB2	SYCP2	GPR157	PRSS27	SLC2A10	PSCA	CITED4	GRB7	CAPS	S100P	IRX4	TP53INP1	LMTK3	KREMEN2	GPR160	KAZALD1	CHMP4C	EPN3	FOXA1	MUCL1	NDUFAF6	TMC5	EVPLL	SPNS2	MMEL1	KCNG1	IL20RA	PRLR	TFF3	ATAD3C	FAM83F	P2RY6	S100A14	ADIRF	ADM2
**hcc1428**	KRT37	ODAD3	HSD17B1	PAGE2	PRLR	MMEL1	CASP14	CLPSL1	S100A7	PADI3	SYT8	SPTSSB	IFNL3	PLAAT2	ABCC11	SPDEF	MAP6D1	HOXC11	VTCN1	SYCP2	SLC49A3	HOXC13	MYRFL	HOXC10	HMX1	CA12	CLPSL2	VCX3A	PAGE5	DEFB126	TMEM270	TP53I11	CFHR3	IGFL1	ANKRD30B	LYPD6B	SERPINA6	LMTK3	KCNK12	TRPV6	ELF5	DEGS2	KCNK15	PGR	RNF223	STC2	EPN3	MUC1	PRSS27	ABCC12
**hcc2814**	FOXE1	SOHLH1	NTS	GBP6	RNF212	TP63	PKP1	DLX4	PTTG2	TP73	KRT6A	LRRC69	CALML3	WNT3A	SLC16A8	TMSB15A	ZFR2	PNLDC1	EVPLL	PCSK9	PRDM13	GREM1	COL17A1	SMC1B	DLX5	DSC3	SERPINB13	KRT74	CENPP	CXCL14	RAB40AL	PKDCC	LY6K	SLC52A1	KRT15	GDNF	CFAP251	RASSF9	KCNJ3	ARHGEF33	KCNMB3	FBLL1	DUOXA1	PABPC3	SYCP2	NEURL1	KRT32	NCR3LG1	NEUROD2	NMUR2
**hcec-1ct**	KRTAP1-5	KRT34	OR10J3	ADGRG7	CCDC9B	GREM1	KRTAP2-3	KRTAP4-9	GDF6	PSG4	CALHM5	KRTAP4-12	RTL8C	KRTAP2-2	TMEM250	TRIML2	TNFSF4	PTX3	BDKRB1	STC2	ZP1	SMIM29	EEF1AKMT4	CCBE1	GDF5	ZNF469	LOX	SERPINE1	MYPN	GSTT2B	BRD3OS	CSF3	IFNE	NPAS1	AOX1	MICA	AJM1	TRPA1	SPOCD1	OR3A3	STMP1	RTL10	ABLIM3	IFNB1	SELENON	TWIST2	ITGBL1	FBN2	NDUFAF8	TSLP
**hct116**	FUT5	GJA9	OR51B2	PRICKLE4	CSNK2A3	MAGEB2	WNT16	CSAG3	SEC31B	FBXO10	OR51B4	KIF4B	GTSF1	TMEM249	TREX2	IFNL2	TRIML2	CCDC142	SLCO1B3	NMU	HOXA9	ABCC2	SUN3	MAP1LC3B2	OR51I1	PNPLA5	PABPC3	EREG	ANP32D	ONECUT3	CD163L1	MYH15	PALM2AKAP2	KRTAP2-3	ADIRF	BMP4	HNRNPCL1	KITLG	NME2	SLC25A52	KRTAP19-1	PTH2	DNAAF3	ANKRD18A	CTAGE1	KRTAP3-1	KIFBP	DKK1	ANKRD23	FGF19
**hec6**	IGFN1	PYDC2	OR51B4	OR51I1	ZNF280A	UTS2	TLL2	KISS1R	FABP6	ERFE	HBE1	TLX3	PAX8	KCNK9	TRNP1	JSRP1	CST6	KRTAP2-3	BDNF	SIGLEC15	CYP4F11	UGT2B7	NT5DC4	HAS3	DCDC2	GPAT2	TRPM2	DNAAF3	CLDN1	TFPI2	TGFA	DMRTA2	NYX	ATAD3C	SIRPB1	ALX3	LCN2	RILP	TESC	ESPNL	ARHGEF35	IRX3	BEST3	MMP23B	GOLT1A	HKDC1	TINAG	ELOVL3	SLCO4A1	ODAD3
**hec59**	PYDC2	NT5DC4	KLK6	LZTS1	PLEKHG4B	DLX5	WNT7A	FABP6	KHDC1L	ZNF724	PAX8	HOXD1	C1QTNF9B	DLX6	KISS1R	RBP7	FGF18	TLX3	ANKRD34B	KITLG	ALKAL2	TNFRSF11B	PRDM6	DCDC2	EMX2	HOXA11	ZNF180	HOXA6	HOXD11	LMO3	NLRP2	IGFBP1	NEURL1	GAL	HOXA10	MORN3	UTS2	HNF1B	FLG	MT1F	ZNF93	CALCB	H2BW1	UGT2B7	FAM86B2	PRRX2	KLRG2	MAP6D1	SYNE4	HMX1
**hec108**	VSTM1	KISS1	PADI3	DHRS2	NT5DC4	TRIM55	ANKRD2	SIRPB1	ZP3	EMX1	ANKRD1	DEFB134	HRK	ONECUT3	FIBCD1	LBX2	SOWAHD	SAA1	ZBED2	SHISA9	CBLN1	F12	PTH2	TMEM253	TLL2	RAB17	HKDC1	EIF5AL1	PAX8	TACR2	BEST3	ERFE	PLLP	NXPH4	YBX2	MSMP	TFPI2	HAS3	NYX	EN2	ENHO	DNAAF3	FBXO2	TRPM2	LCE1B	MROH8	MACROD2	CA11	FXYD2	CCR10
**hek293**	ALKAL1	KRTAP26-1	SEC31B	KRTAP19-1	CRYGD	CALCB	HOXD13	OSTN	POU4F1	PLAC1	TMSB15B	HEY1	CNPY1	EPHA8	CA2	SCML1	PMAIP1	PRSS33	EN2	ANKRD18B	CBS	NCR3LG1	C1QL4	PIGY	GLA	UBE2T	KIAA0408	CA8	ARV1	DEPDC1	TMSB15A	PSAT1	HOXA11	RPF2	MAP7D3	LIN28B	TCF24	CCNB1IP1	ZNF215	HMGN5	ITGB3BP	MAD2L1	SPDL1	NELFE	TMEM35B	MND1	ASNS	CLGN	PHGDH	HNRNPCL1
**hek293t**	OR11H2	H2BC6	H3C12	THSD8	DEFB107B	TBC1D28	TRIM49B	OR8J1	CTXND2	MMP26	OR2V2	HNRNPCL2	SLC26A3	ANP32D	GPR174	H1-4	H1-3	ARL14EPL	H2BC17	OR2A12	H4C3	OOEP	RADX	OR1L6	ADGRE3	MTNR1B	CALHM4	CCDC194	OR8A1	TREX2	DCAF8L2	TPTE2	ADARB2	STEEP1	GNGT1	CFAP20DC	TBC1D21	MAP3K21	EIF2S3B	EFCAB3	POM121L2	ARMH4	POTEA	PRH1	MTARC1	MROH5	H4C6	CFAP92	H2AC13	POTEI
**hela**	PRAMEF26	MRGPRX4	FAM209A	CHRNA9	EFCAB3	AMY2A	KCNK15	PPP1R42	TEX36	GLP2R	KRTAP9-9	SRRM5	NTS	C4BPB	HTR1E	CT83	TBC1D28	CHRNB4	CCN5	ANP32D	FANCD2OS	CNGB3	PRSS21	CPS1	EFCAB10	CREG2	ABCC2	TRAPPC3L	ALX1	PSCA	SHC4	TMPRSS15	ETNPPL	NBPF6	ADGRE2	KLRC2	MEIOC	MYPN	SPINK13	PRAP1	SLC12A3	EYA4	EVPLL	SMIM10	OOEP	IL10RB	NDST4	ASIP	RADIL	PP2D1
**helper t cell**	IL3	IL21	CSF2	IL22	CD40LG	IL13	IL31	FASLG	IFNG	IL26	TNFSF8	CCR4	TNF	IL10	ZNF831	IL4	TNFRSF4	IL17A	GPR42	CCL4L2	IL9R	ICOS	PDCD1	BTLA	GCNA	NFKBID	TRAT1	OR3A3	BCL2A1	CCL20	ZNF165	TNFRSF18	THEMIS	NR4A3	CGAS	TMEM273	CXCR6	JAML	VSIR	CD2	CCR8	FAM241A	EPOP	EVI2A	IRF4	CCR6	CD96	IL21R	BATF	CD5
**hematopoietic cell**	P2RY12	CRYBB1	GPR34	TREM2	CCL24	P2RY13	CD1A	CX3CR1	ADGRE1	FCGR3B	TMEM119	C1QC	C1QA	CRYBA4	C1QB	ADORA3	LTC4S	CSF1R	OR6C70	PLD4	GPR84	KCNK13	TASL	HPGDS	MOBP	HLA-DQB2	SLC2A5	LY86	CCR5	CD207	SLCO2B1	FOLR2	VSIR	STYXL2	TCF24	PF4	ANGPTL7	AIF1	SIGLEC8	CCR6	STAB1	P2RY6	OTULINL	OR6V1	DCANP1	CD33	TNFAIP8L2	FGD2	C3AR1	CLEC5A
**hematopoietic multipotent progenitor cell**	IL12A	NUDT4B	FLT3	PACC1	ADGRG3	DNTT	BNIP5	PRTN3	MPL	TIFAB	DRC7	DCDC2C	OR2L8	SEPTIN1	FRMD7	TREML2	H2BC4	WDFY4	CGAS	RSC1A1	SEPTIN2	MYL10	MC5R	OR2L3	MPO	SERINC4	RGS18	TNIP3	RAG2	PIP4P1	ADGRL4	INAFM2	PRSS57	HBD	SLC18A2	GJE1	SLC35D3	FUT7	CLEC12A	H1-1	CD34	RESF1	NRROS	NAPSA	IRGM	GPR25	MYO1H	GPR171	BPIFC	EOLA1
**hematopoietic oligopotent progenitor cell**	MPIG6B	HBD	INKA1	GP9	GP1BA	GCSAML	HBG2	AHSP	MYL10	GATA1	HBM	PRSS57	CLEC1B	RGS18	ITGA2B	TREML1	CRYGD	FCER1A	HBA1	OTOS	HBG1	MPO	HBA2	SELP	GYPB	NFE2	PPBP	ICAM4	CMTM5	GFI1B	PF4	KLF1	BPIFC	AFP	ALB	TUBB1	FCN3	GYPE	HEMGN	HBZ	F2RL3	MMRN1	GP6	CPA3	ANGPT1	ADGRG3	AHSG	APOA2	FAM178B	FCN2
**hematopoietic precursor cell**	CENPVL1	PRTN3	CTSG	MS4A3	MPL	CRYGD	TREML2	PRSS57	MC5R	NFE2	FLT3	AVP	TSPAN32	IL12A	MPO	F2RL3	GP5	LYL1	LHCGR	ADAMTS13	FUT7	ANGPT1	PGLYRP2	IZUMO1R	PADI4	OTOS	RGS18	ELANE	RAB44	FRMD7	BPIFC	NKX3-3	MUC13	LMO2	CD34	OR5H8	GPR171	SPNS3	PRG3	IL4	KCNK12	SPINK2	ADGRG3	PTPRCAP	CD177	SH2D5	MYB	RAB38	DAPP1	SPNS2
**hematopoietic stem cell**	DYTN	PRSS57	LRRC70	OR2B2	CRYGD	TPSD1	CYTL1	CRHBP	GCSAML	SPINK2	KCNK17	CHRFAM7A	MPL	IFNL4	SMIM24	TPSAB1	OR10A2	AVP	HTR1F	OR2T8	NFE2	ATP8B4	FLT3	H2AC7	DEFA1	OR2L8	H2BC7	TESPA1	GAPT	PRTN3	MC5R	MRGPRX2	TAS2R60	FCER1A	MPO	H4C2	CPA3	IL12A	DNAH10OS	ADGRE2	RAB37	PRAM1	OR2AK2	TNFSF4	MS4A3	UQCRHL	KLRF2	OR10G2	TSPAN32	CLEC9A
**hepatic stellate cell**	GDF2	COLEC10	COLEC11	LRAT	BMP10	ADRA2B	ANGPTL6	VIPR1	HAND2	RELN	NGF	CLEC4F	DIO3	GDF10	NR1H4	CLEC4G	MUSTN1	HGF	WFDC1	PTH1R	TCF21	COL14A1	TNFRSF11B	PLVAP	ABCC9	STEAP4	TEX29	RSPO3	TBX20	GPBAR1	ALB	GPAT2	BMP5	BCO1	PRELP	ECM1	CD5L	GCGR	HHIP	NTN1	KCNJ8	HPX	HS3ST3B1	AARD	AHSG	LRRC32	CFHR2	ITGB3	ADRA1B	GJC2
**hepatic stem cell**	GNRH2	SMLR1	FGF19	ITLN2	AKR1D1	SERPINA4	UGT2B15	AFP	CHST4	SLC17A4	PLA2G12B	FGA	FGB	APOB	TM4SF4	TAFA4	CPB2	ASGR2	CYP4A22	DEFB1	ALB	DPYS	GSTA2	CXCL5	VTN	APOC3	CCKBR	CYP2A7	WFIKKN1	SERPINA6	LCN15	HABP2	SERPINA1	CXCL6	APCS	AMBP	AHSG	AGT	APOM	CA4	RBP4	ANXA8L1	IL1RL1	AVP	DHRS9	FTCD	TMEM92	FGG	A1CF	CYP4A11
**hepatocyte**	APOA5	SLC17A2	APOF	CYP8B1	MBL2	PLG	CFHR2	CPN2	ITIH1	APOC4	OTC	AGXT	SERPINA11	C8A	LECT2	TAT	CYP1A2	HPX	HAO1	MAT1A	BHMT	UROC1	SDR9C7	SERPINC1	AHSG	HAMP	ANGPTL3	ITIH4	SLC25A47	SERPINA7	GYS2	SERPINA12	C8B	APOC3	CPB2	PZP	TDO2	ITIH3	GNMT	GLTPD2	ABCB11	CREB3L3	HSD17B13	APOH	CYP4A22	SAA4	ADH4	BAAT	ELOVL3	MASP2
**hgsoc**	RHEX	SCGB1D1	CT45A3	CT45A5	LIMS3	CST5	H4C12	PAGE2	H2BC12	NPIPA9	TP53TG3C	KLK7	ADGRG7	H3C4	CTAGE8	H3C6	H2AC18	SCGB1D2	RGPD6	MUC16	H2AC8	ZNF716	RGPD1	H2BC5	H2AC17	KRTAP5-8	ANKRD20A1	ZNF679	H2BC4	H1-2	H3C1	NR2E3	LCN1	CSAG2	CALHM3	SCGB2A1	GALNT8	SINHCAF	OR8A1	H2AC19	RGPD8	FAM166C	H2BC13	GAGE12H	MSLN	WT1	BNIP5	KLK6	UGT2B7	OR56A1
**hindbrain**	HES3	WNT1	FOXB1	EN1	ERAS	EPOP	TAL2	FAM181B	ZIC3	OLIG3	SOX21	VSTM2B	FGF8	LHX5	NKX6-1	DNMT3B	FIGNL2	LPAR4	SOX1	MOGAT2	FAM181A	IRX5	SMTNL2	SP5	JPT2	LBHD2	WNT8A	LIN28A	STUM	ZBTB12	RIOX1	CRABP1	NPTX2	FGF3	GBX2	BTBD17	CCDC177	SELENOH	DQX1	POU3F1	RCOR2	MYCN	HPF1	PRTG	XKR5	SPOUT1	GON7	MYRFL	SALL4	NKX6-2
**hindgut**	APELA	CDX4	TRH	EVX1	FGF8	KRTAP17-1	LECT2	SP5	FGF17	IHH	SOX17	TLX2	KLK14	ERAS	UPK3A	MOGAT2	CDX2	CLDN6	MNX1	CYP26A1	SMIM10L2A	CITED1	HOXB8	IZUMO1R	WFIKKN1	FOXA3	DGKK	GPR83	LIN28A	NECTIN2	DUSP9	EPOP	KRT83	PSORS1C2	CFC1	VSTM2B	GLTPD2	APOM	FOXA2	NKX1-2	CDX1	WNT5B	PRTG	GREB1	HES7	FIGNL2	MSX1	TMPRSS2	HOXC8	SELENOH
**hippocampal granule cell**	C1QL2	C1QL3	FAM163B	DDN	KCTD4	NEUROD2	MOBP	GABRA5	RASL10A	ICAM5	KRT9	HPCA	LCT	CPNE6	CAMK2A	RPRML	GRM2	LRRTM1	SLC8A2	DGAT2L6	CABP7	SLC30A3	FRRS1L	RNF112	CHRM1	CPLX2	NPTX1	PCDH20	ANO3	NEFL	GRIA1	PDYN	CHST1	NPY2R	WIPF3	SOWAHA	GRIA2	LGI1	SERTM1	NPTXR	CAMKV	OLFM1	PNMA8B	DRD5	PCDH8	RGS7BP	PHYHIP	NRGN	GPR22	RPH3A
**hippocampal neuron**	PROP1	KRTAP12-1	IFNA7	SSTR4	IL9	ELOA2	GJA10	IFNA2	GALR3	KRTAP11-1	NEUROD6	VXN	WNT1	FAM237A	CPNE6	KRT73	SLC17A7	MPPED1	GABRA5	OR14I1	RPRML	PRKCG	FEZF2	TRIM48	HPCAL4	DRD5	ICAM5	MC4R	CTXND1	CAMKV	IL9R	GALNT9	NR0B1	CHRM1	SPINK8	OR11L1	HTR1A	IFNA5	HPCA	GRIA1	CAMK2A	FAM153B	SHISA7	LRRTM1	DDN	MAS1	TBX6	ADGRA1	ANKRD63	SPRN
**hippocampal pyramidal neuron**	NEUROD6	ANKRD63	DDN	GABRA5	MC4R	SPINK8	CPNE6	RPRML	SERTM1	C1QL3	HPCA	SSTR4	CAMKV	ICAM5	HTR1A	MPPED1	GPR22	GPR26	GABRA1	KCNJ4	SHISA7	SPRN	CCK	CAMK2A	FRRS1L	GRM5	GRIA1	CHRM1	LRRTM1	DRD5	GRIN2B	NEUROD2	GRIA2	HPCAL4	RNF112	GALNT9	CRYM	SV2B	FEZF2	SNURF	KCNV1	CELF5	BRINP1	GP1BB	ITPKA	SLITRK3	CREG2	RASGRF1	HAPLN4	CNKSR2
**hmc18**	FAM9C	COLEC10	PRSS33	NTS	CARD18	VSTM1	OBP2A	LIF	EREG	TRPM8	KREMEN2	PRDM13	NAT8L	OR2B6	HES7	DAW1	SLC45A1	OSM	SLC30A3	SAPCD2	NKX2-5	SEC14L4	TNFRSF10C	TRIM7	CEND1	MYEOV	CERS1	ANKS6	DIRAS1	LAMC3	SLC4A1AP	TUBD1	RGS17	PKN3	MAPK12	PRKCG	TRPM2	FCHO1	B4GALNT4	NR2F1	TWIST1	HSD17B1	HCN2	NANOS3	ASPHD1	CBS	SPRY1	TRABD2A	FSD1	PANX2
**hmel**	IGFL3	LCE3D	LCE1C	SPRR1B	KRT6B	MRGPRX3	KRT16	CARD18	WFDC12	KRT6C	LCE1E	SPRR1A	KRT6A	RNASE7	SERPINB5	IVL	GJB5	CALML3	KRT14	LCE1F	SDR9C7	KRT5	CRCT1	SPRR2D	PI3	CSTA	DSG1	SPINK6	COL17A1	MMP28	SAA2	SPRR4	HSD17B1	FAM25A	GJB2	QRFPR	EPGN	SAA1	SPINK7	NOX5	FGFBP1	S100A14	SERPINB13	GPR87	SPRR3	TNS4	SOX15	CA2	FOLR3	WFDC5
**hnsc**	MAGEA9	CT45A1	KLK9	OR6C70	CTAGE4	PNLIPRP3	ANXA8L1	SCGB1D1	CSAG3	TNFRSF6B	CSAG2	RNASE7	OR56B4	TP53TG3C	IL1A	CT45A10	KRT6C	CT45A3	OR52L1	CTAGE15	OR2A7	TP63	MAGEA9B	KRT6B	OR6C2	RAET1L	SAGE1	TAS2R39	TP53TG3B	OR1J1	TRIM49D2	ALPP	GSDMC	DSG3	TM4SF19	TBC1D3L	SERPINB13	SLCO1B3	OR1J4	CYP4F11	MMP13	CLDN24	TP53AIP1	CTAGE8	LGALS7B	AMTN	OR11H4	MAGED4	BNC1	COL17A1
**hofbauer cell**	GH1	LILRB5	TMIGD3	VSIG4	FOLR2	LYVE1	PAGE4	HBE1	FCGR1A	HBZ	MS4A4A	F13A1	SIGLEC1	CRYBB1	CD163	OTOA	SLC28A1	XAGE3	CGB3	SIGLEC9	FCGR2A	CCL13	RETN	CD5L	PCDH12	GPR34	TREM2	SIGLEC14	FPR1	SIGLEC12	CEMP1	MRC1	HPGDS	CD14	TIMD4	CGA	ADAP2	GRIN2C	CD180	BIRC7	MARCO	RNASE1	ERVFRD-1	SLCO2B1	C3AR1	SIGLEC11	LEP	NPL	SUCNR1	MS4A6A
**hop62**	CD70	AMBP	CHRDL2	SPANXC	LCE5A	CSAG1	TRIM16L	DIO3	MYEOV	STC2	CCL26	GREM1	FIBCD1	NNMT	MAGEA12	WDR72	BHMT2	CD163L1	ADAMTS12	SIX2	AKR1B10	MAGEA6	AKR1C1	SMIM10	HES7	AKR1C3	RASIP1	PRAC2	KISS1	IGFN1	MSC	CXCL1	SOWAHD	SPON2	CDKN2A	TMEM255B	LCE1E	MAGEA3	NR0B1	KRT81	RAB32	ADAMTS6	HRCT1	SPANXA2	FGF5	MMP7	NPB	SBSPON	POPDC3	CYP27C1
**hos**	FOXR1	NPPB	FOXE3	KISS1	MEIOB	ZNF732	NKX2-8	VSTM1	SLC10A4	HOXC12	BARX1	GAL	LY6K	KRTAP2-3	HMX2	LAYN	BHMT2	HS3ST3A1	C1QL1	FAM72D	ANKRD18B	NKX2-4	HPDL	TLCD5	PRDM13	FGF1	ARL9	CARMIL2	ADAMTS12	TMEM88	CCDC144A	IL11	CLDN11	TAGLN	GBX2	MARCHF4	NPY	HOXA11	BNC1	RBP7	GREM1	TXNRD2	MT1A	PRAME	HMX1	FAM72C	SPOCD1	ARHGAP11B	QRFPR	TRIM58
**hs729**	LCE3D	ASB5	KRTAP1-5	VAX1	HOXC12	LCE2A	GDF5	CHRNA1	GDF6	KRT33B	MYOD1	FAM180A	KRT34	MAP3K7CL	LRRC17	HAS2	STC2	PGF	KRTAP1-1	H2AC1	TMEM233	LRRC38	LRRC15	APCDD1L	CSAG1	TRIM55	LOX	MMP3	KRTAP2-3	FGF5	KRTAP4-8	ADAMTS6	PRAC2	IL11	JSRP1	AK5	VEGFC	ZNF699	MAGEA11	PITX3	BDNF	BAALC	SIGLEC15	DKK1	FGF2	NEXN	CXCL3	CPED1	INHBA	MAGEA12
**hs852t**	IL24	FLG	GDNF	LCE2A	SHC4	KLHL35	ZNF365	MYEOV	KIF6	MIA	FDCSP	DKK1	TPBGL	NES	CCDC81	COL22A1	UCN2	METTL7B	PHEX	SPOCD1	LYPD1	CATSPER1	EN1	WNT11	CTCFL	STEAP1B	MMP10	IFITM10	NXF3	RAB9B	SRPX	NDP	ITGA2	EN2	TMSB15B	HOXC13	TNFRSF10C	IL11	NAT8L	MELTF	FST	HOXA4	POPDC3	FANCB	NOTCH2NLA	AK5	LTO1	THAP12	S100A3	PRCD
**hs939t**	UCN2	ITIH6	KRTAP1-1	SLAMF9	MELTF	BAIAP2L2	S100B	GDNF	MIA	DCT	MLANA	KCNK15	LCE2A	CST1	S100A3	LOXL3	MAGEA3	SPRR2D	FAM78B	KLF14	MMP17	CYGB	KRTAP19-5	RTL9	LZTS1	BMP8B	KRT27	MC1R	EXTL1	PMP2	LYPD1	NES	MFSD12	COL19A1	TMEM171	FDCSP	STEAP1B	POPDC3	MYEOV	LIF	CSAG1	MMP1	NGFR	SERPINE2	CPA4	PAGE2B	SRPX	A1BG	TRPM8	APOBEC3B
**hs944t**	PAGE5	SSX1	KRT27	KRTAP19-1	FAM133A	SPRR2D	MAGEC2	CSAG1	S100B	MAGEA12	UCN2	LCE2A	CAPN3	CT55	MAGEB2	PMEL	PRAME	MAGEA6	MAGEA3	PASD1	ROPN1B	MIA	DCT	TRPM8	MAGEA1	COL9A3	PRSS33	BIRC7	COX7B2	MELTF	VCX3A	LYPD1	MFSD12	XAGE3	PARP16	MAGEA11	PMP2	HCG22	MEIOB	SRPX	MOK	PRCD	STEAP1B	VCX2	FAM86B1	MPZ	MRGPRX3	FAM78B	SAA1	MLANA
**ht55**	IGFL4	NT5DC4	PHGR1	KLK6	ASCL2	TPSG1	RBBP8NL	CYP2W1	CDX1	MOGAT3	EPS8L3	KRT20	DIO3	TRIM31	POU5F1B	GSDMA	DPEP1	DUSP29	VIL1	LCE1E	TRIM15	CYP2B6	RNF43	KRTAP4-1	IL20RA	BMP4	KLK10	HOXB8	FRMD1	PRR9	KRT74	USH1C	NOTUM	PPP1R14D	PDE4C	KRT23	LGALS4	KLK1	HOXB9	CCNI2	CD163L1	MROH6	PRSS3	CDX2	GPR35	LARGE2	FGF19	SCN5A	GPX2	MYT1
**ht1080**	DHRS2	OR51I1	CT55	PASD1	OR51B4	SSX1	PAGE5	MAGEA11	CATSPER1	KRT81	LCE1E	LCE2A	HBE1	HTR1D	USP26	MAGEB2	KCNMA1	MAGEA12	CSAG1	MAGEA3	POTEG	CACNA2D4	CD70	HS3ST3A1	PGF	KCNG1	STC1	AMIGO2	PLAU	HHIP	IGFL3	SPOCD1	SUGCT	UCN2	LYPD1	IL11	FCRLB	MTMR11	FAM72C	PAEP	ZP1	NPFFR2	ARHGAP22	POPDC3	FOXD1	TRIML2	MAGEA4	SPHK1	LETM2	SEMA7A
**ht1197**	CLEC2A	CST5	PRR9	SPRR3	IGFL2	DEFB126	KRT3	SPRR2D	LY6G6C	GJA5	LYPD5	VGLL1	RAET1L	OLR1	NAA11	HBE1	OR51B4	CST6	CLDN6	IL20RB	KHDC1L	ARHGAP40	ERP27	SLC1A6	CSAG1	LHX5	UPK1B	KLK6	GSDMC	HES2	OXTR	BFSP1	HSD3B1	TMEM52B	SOX15	ZNF114	ZNF488	TPTE	CDA	POTEG	IVL	PTGES	IL36RN	MAGEA12	CALHM3	GPR87	MAGEA6	SUN3	C4BPB	IL37
**ht1376**	PRR9	CRCT1	RNASE7	PAGE2	SPRR3	PAGE2B	SYT8	ZBED2	PAGE5	VGLL1	PSCA	S100A7	MMP28	SSX1	FAM133A	FAM9C	UPK1B	MAGEA12	IL20RB	TNNT3	LAMB4	KLK5	NEURL1	ANKRD65	LHX4	CSAG1	HRK	SLC35F3	SPRR2D	PCDH1	KRT14	ADAMTSL4	MAGEA3	GTSF1	GJB4	ARSI	ZNF488	SIRPB2	CALB2	CCK	SNCG	LGSN	MROH6	SPRR1B	TNNI2	H2BW1	FAM83A	PAGE1	GJB5	GJB3
**htmc**	KRTAP2-2	KRTAP2-1	KRTAP1-5	DIRAS3	NOX5	PLPP4	MYOC	CXCL6	CCL26	PITX2	GDF5	KRTAP1-1	KCNE4	CA12	VMO1	IRAG1	BDNF	RGS4	GMNC	MSMP	COL8A2	CYP1B1	CEMIP	WFDC1	NDUFAF8	STC1	MAP3K20	FIBIN	PAPPA	PPP1R3C	RAET1G	NGF	PMCH	PTGS2	CCN2	CMKLR1	TAGLN	BDKRB1	ITGBL1	CNTN3	THBS2	PF4V1	SYNPO2	LTBP2	ALX4	GSTT2B	FGF7	PRSS12	HAPLN1	CPA4
**huh1**	RBMY1A1	RBMY1D	RBMY1J	RBMY1E	UGT2B4	SSX4B	AKR1C4	RBMY1F	MAGEA2B	GAGE12H	MAGEA2	CTAG2	SSX4	PRAMEF19	CCL15	CSAG3	SULT1A4	AHSG	SMLR1	NR0B1	HHIPL2	FGL1	CFHR5	SSX2B	ORM2	CYP2B6	GAGE12C	APOH	AKR1B15	ASGR2	SLC22A9	KRTAP19-1	UGT2B10	APOA2	GSTA1	ORM1	GSTA2	ABCC2	AMBP	BAAT	ANGPTL3	CES1	HPD	SAGE1	GLP2R	FCRL4	CTAGE4	GLTPD2	PRAMEF18	SAA4
**huh6**	CST4	CST1	LCN15	NLRP7	BIRC7	UNC93A	IHH	NPPB	MT1H	ITLN2	CST5	NT5DC4	COL2A1	TMEM52B	SLC38A8	IGFL2	PAGE4	NEU4	SLC51B	FOXH1	DEFB126	AMHR2	KRT37	DRD2	MT1G	SHH	APOA1	NLRP2	FOLR3	LIN28A	NODAL	TAC3	SPAAR	C4BPB	ASGR2	THPO	IL17B	PLP1	SP5	DMRT1	RBP2	CDX2	SMIM10	SLC26A9	GIP	PKDCC	CLDN19	ODAM	HPD	CHIT1
**huh7**	TUBB8B	RFPL4B	KRT37	FGF19	SLC22A9	ANGPTL3	FGA	SERPIND1	ASGR2	AMBP	FGG	FGB	APOA2	ORM2	APOB	AHSG	AFP	ITIH2	KRTAP20-2	NPPB	KLB	HPX	IFNL4	LGSN	MATN3	SMLR1	ORM1	OR8U1	LCE2C	LCE2A	FAM151A	A1CF	APOA1	LIPC	HAVCR1	CPB2	PPIAL4A	OR8G1	SERPINA5	HGD	VTN	MT1H	SERPINA6	FGL1	TM4SF5	IL9	KRTAP19-5	NTS	PAH	GPRC6A	UNC93A
**huvec**	BMX	APLN	IL1RL1	MMRN1	SPAAR	TM4SF18	MSMP	FAM124B	ECSCR	ROBO4	ADGRL4	TMEM255B	EFEMP1	ICAM2	TIE1	PTX3	TNFSF4	MMP1	SULT1B1	ERG	SHE	BCL6B	ADTRP	KRTAP26-1	CLDN11	PGF	HHIP	CALCRL	ESAM	CCL14	LAMA4	SOX17	PROCR	CLDN5	CAVIN2	HSPG2	RNASE1	PALMD	LYL1	PLPP4	SERPINE1	SPOCD1	EDN1	SOX18	GIMAP8	GPR4	CD93	TFPI	FGF16	TNFAIP8L3
**hypothalamic progenitor cell**	FAM181A	GSX1	HELT	SOX3	ASCL1	OTP	HCRT	FEZF2	NKX2-1	CRYGD	SOX1	NR2E1	FGFBP3	TMSB15A	GOLGA6L2	ANKRD20A1	VAX1	NKX2-4	MAGEL2	PPP1R17	FEZF1	GPC2	BTBD17	LHX5	SOX2	RAX	SHH	OLIG2	HES5	TTYH1	RFX4	GHRH	ZSCAN1	SIX3	STMN4	PMCH	CXCR4	LIX1	NKX2-2	PTPRZ1	ZNF660	NKAIN4	NOVA1	NEUROG2	FABP7	TCEAL7	NUTM2E	SYT6	ZNF219	POU3F4
**ialm**	KRTAP4-8	COLEC10	KISS1	OR4C6	KRTAP2-3	MEIOB	ZNF534	FAM90A1	NKX2-1	PAEP	KRT33B	ZNF479	WFDC3	VSTM1	ZNF781	KCNF1	OR51I1	CFAP251	RNF212	PLCZ1	LAYN	STC2	SERPINB7	FGF1	KRTAP4-1	LY6K	TEAD4	ZNF699	NKX2-5	ESM1	MARCHF4	ZNF582	ZNF677	PRDM13	KRT34	KCNMA1	MYEOV	FAM72C	FOXM1	EVA1A	ASB9	KRTAP1-5	HUS1B	LCE5A	DEFB136	CACNA2D4	ZNF880	PHF19	FAM72D	CIB3
**iga plasma cell**	TNFRSF17	IGLL5	FCRL5	MZB1	DERL3	TASL	JCHAIN	CCR10	FCRL2	CD79A	TNFRSF13B	MEI1	H2BC8	POU2AF1	AMPD1	H2BC15	SLAMF7	HLA-DOB	ENAM	PNOC	NUGGC	TAS1R3	CD27	NPIPB13	JSRP1	PRDM1	H2BC10	IRF4	H2BC7	GNG7	KIF19	SPAG4	OR11G2	GPRC5D	H2AC8	EAF2	BLK	ZBP1	SEPTIN1	RETNLB	DEFA5	CD38	DEFA6	ZG16	GPR15	RASGRP3	HSH2D	PKHD1L1	CPNE5	FKBP11
**iga plasmablast**	F8A2	RIMBP3B	FOXD4L3	OR1C1	OR1G1	OR51G2	NUGGC	OR10C1	PRY	OR1J2	HSFX2	TBC1D3H	NBPF4	KRTAP5-4	JCHAIN	TNFRSF17	OR1E1	DEFA1B	FCRL5	FGF23	OR4K2	TRIM64C	SPATA31A6	NPIPA7	TBC1D3F	GOLGA8F	OR5C1	SLAMF7	OR12D3	PRAMEF4	MZB1	CTAGE15	PRAMEF1	RBMY1D	OR2A42	IRF4	LEUTX	BMP8B	RAB6C	GPRC5D	POU2AF1	OR5F1	OR2D3	TBC1D3G	RGPD4	TENT5C	OR4M2	NUTM2E	ZNF705B	OR52K2
**igg memory b cell**	OR2T11	OR2T10	TNFRSF13C	FCRL2	SEPTIN1	TASL	BLK	MS4A1	HLA-DOB	BANK1	NIBAN3	SHFL	TRAPPC14	SPRING1	PAX5	FCER2	TLR10	CD79A	CXCR5	P2RX5	H3-3A	HLA-DQA2	H2AC15	FCRL1	OR56B1	MARCHF8	TNFRSF13B	CD22	OR2T2	OR2G6	FCRL3	TMEM273	FCRL5	RESF1	ANTKMT	SHISA8	H2BC6	GDF7	PNOC	RUBCNL	H2AC20	FAM174C	CD1C	SPIB	FCRLA	RIPOR2	HLA-DQA1	PABIR1	PIP4P1	ZBTB32
**igg plasma cell**	FCRL5	H2BC8	TNFRSF17	TASL	H2BC7	FCRL4	MZB1	IGLL5	DERL3	H2AC8	JCHAIN	FCRL2	CCR10	H2BC10	OR11G2	CD79A	H2BC14	POU2AF1	H2BC11	H2BC15	PNOC	JSRP1	TAS1R3	H3C7	H4C13	H1-2	MEI1	H2AC11	H4C5	HLA-DOB	SPAG4	H3C10	H2AC4	H4C8	CD27	TNFRSF13B	H4C2	H4C1	H2BC4	H1-3	H2AC15	OR4C6	SEPTIN1	H2BC6	H2BC9	SLAMF7	H4C6	H2BC3	NUGGC	GPR15
**igg plasmablast**	F8A2	CDY1	OR10P1	CDY2A	OR7C2	OR1E2	OR10AD1	PRAMEF5	TBC1D3H	OR6C3	NUGGC	FOXD4L6	OR4K15	OR9A2	FCRL5	JCHAIN	DEFA1B	OR2V1	OR4D6	KRTAP1-3	SLAMF7	OR9A4	OR13C9	MZB1	SPATA31A6	OR6M1	FOXD4L3	HTR3D	OR4F5	TNFRSF17	KLHL14	TBC1D3F	GOLGA8F	TBC1D3G	POU2AF1	RGPD4	IRF4	OR2J3	PRAMEF4	TENT5C	OR4C12	LIMS3	TRIM49B	CCR2	GGTLC3	NOMO3	NOMO2	MAN1A1	ZNF705B	KIF4B
**igm plasma cell**	H2BC8	H2BC7	H4C13	H4C6	H2AC4	TNFRSF17	H2BC10	FCRL5	CLEC20A	H3C10	H2BC15	H2AC11	H2AC8	H2BC14	MZB1	H4C2	H4C5	FCER2	H4C1	H2AB3	H2BC9	H2BC11	DERL3	H2BC3	H3C12	H1-3	H3C11	TASL	H2AC13	H2BC6	POU2AF1	H1-2	DEFA3	H3C1	KLHL14	CLEC17A	CALHM6	H1-5	H2BC21	CD79A	CCR10	H3C7	PNOC	FCRL2	SAMD7	H2BC5	H4C8	MS4A1	SEPTIN1	H2AC16
**igr1**	PAEP	MLANA	KRTAP5-2	TYRP1	CAPN3	DCT	KRTAP19-1	PMEL	ABCB5	LCE2A	CLDN14	GPR143	SPRR2D	BIRC7	CST2	PLAAT1	DEFB136	LYPD1	SLAMF9	UCN2	GJB1	CSAG1	MYOZ2	TRPM1	EN2	TUSC1	S100B	PHACTR1	MELTF	FRZB	TRIM63	ROPN1B	POTEG	TRPM8	MAGEA12	HSD17B1	QPCT	CERS1	GYG2	ZNF775	CACNA2D4	MAGEA3	FAM86B1	MAGEC2	ST3GAL4	DAAM2	PSG11	SNCA	RAB32	POPDC3
**igr37**	KRTAP19-1	LCE2A	MLANA	BIRC7	DCT	PMEL	CAPN3	TYRP1	PAEP	ABCB5	TRPM1	UBAP1L	DEFB126	ROPN1B	UCN2	OCA2	S100B	LZTS1	MFSD12	SLC30A10	TRIM63	DEFB136	CA14	KRTAP5-2	GPNMB	IRX6	DNASE2B	FAM167B	QPCT	BACE2	MITF	RAB38	MGAT5B	SSX1	TNFRSF14	CERS1	CLDN14	MAGEA6	ST3GAL4	BAIAP2L2	PRAME	HMX1	KLF14	RAB32	PHACTR1	GPR143	LYPD1	SNCA	SLAMF9	MAD1L1
**igrov1**	IGFN1	CLDN16	ASCL5	BARX1	ZNF280A	FLG	CCM2L	TRIM55	FOXE1	KRT27	PADI1	TLX3	UGT2B7	C1QL1	DCDC2	KRTAP3-1	HKDC1	PAX8	HNF1B	EDN2	SBSPON	GLOD5	DPPA2	CXCL5	TM4SF18	FAM25A	FGF18	KCNK9	TNFRSF11B	WNT7A	NYX	HOXA6	FXYD2	COMP	ERVMER34-1	CITED4	HOGA1	PRAC2	PRODH	ABHD12B	PPP1R3G	FIBCD1	LY6K	CCR10	TLL2	B3GALT5	FAM86B2	HAS3	PITX3
**immature astrocyte**	HEPN1	PMP2	GFAP	FAM181A	GSX2	ARSF	AQP4	HEPACAM	GALR1	NDP	BCAN	RFX4	SCRG1	DBX2	BBOX1	BAALC	GSTT2B	MYBPC1	MLC1	NR2E1	SLC25A18	POU3F2	CHST6	OR5B12	GPR37L1	PAQR6	POU3F4	FAM107A	KCNF1	PIRT	ELOVL2	ADCYAP1R1	APC2	MT1M	FAM181B	CNGA3	SLC1A2	KLHDC8A	CPNE4	FAM131B	APOL4	TRIM9	S100B	RGMA	OLFM2	RPE65	VN1R1
**immature b cell**	FFAR1	PMCH	CLEC20A	MS4A1	FCRL1	TNFRSF13C	TRIM10	FCRLA	KRT83	CD79A	MMP8	CD19	PAX5	BTLA	CD79B	CXCR5	VPREB3	BLK	TSPO2	LY6D	CD22	BANK1	SPIB	CEBPE	GYPA	GPR18	CLEC17A	BFSP2	FCER2	TREML2	HVCN1	TLR8	SLAMF6	CD177	POU2AF1	ALAS2	CD72	XKRX	GRAP	SLC4A1	TLR9	MEP1B	CD180	FAM129C	FPR2	TNFRSF13B	CCR7	RHD	NEURL3	PGLYRP1
**immature natural killer cell**	DIPK1C	KRTAP20-4	OR5H1	OR52N2	HMHB1	REELD1	TMEM272	SCART1	NCR1	OR4D1	PTH2	OR3A2	NCBP2L	KCNK17	SH2D1B	IL36A	KLRF2	RFPL1	UMODL1	TNFSF4	CYP26C1	LCNL1	MORC1	PEAK3	ANKRD40CL	RFPL3	GCSAML	KLRK1	XCL1	RTP5	QRFP	NUTM2D	ATP6V1FNB	CMA1	KLRC3	TNFSF11	OR4D9	TBC1D3D	IL31	MMP25	GOLGA8R	KLRC1	OR2A25	B3GALT9	TRPM8	XCL2	ABCB5	FREM3	OR2B11	TAS2R31
**immature nk t cell**	CD160	RLN3	KLRC1	QRFP	GPR15	LACTBL1	TBX21	KLRK1	IL2RB	CXCR3	XCL1	CRTAM	CXCR6	NCR1	TXK	KCNH4	UBASH3A	GPR18	LAX1	GPR174	PGLYRP2	IL12RB2	IL18R1	NEURL3	TNFRSF18	GIMAP6	CTSW	KLRD1	HSD11B1	PRKCQ	GIMAP5	ZAP70	SH2D2A	GIMAP4	ITGAD	LCK	PGLYRP1	ADORA2A	GPR171	PYHIN1	GRAP	GIMAP8	KLRC2	SLA2	CD3G	NKG7	LY9	TRAF1	CD28	TREML2
**immature t cell**	CCR9	RAG2	RAG1	DNTT	ENDOU	CD8A	CCR8	WNT8B	THEMIS	COLQ	CD28	ARL5C	CD4	CHRNA9	IL21R	SH2D1A	UBASH3A	TDRD5	LCK	AQP11	TCF7	RORC	CD5	TRAT1	CD3G	TBXA2R	CD247	LAT	SLC16A5	SATB1	CD6	SIT1	CD27	SLA2	SLAMF6	BCL11B	TREML2	ARPP21	SKAP1	ZAP70	XKRX	KCNA3	SASH3	CD3D	GRAP	UNC5CL	LINGO4	IL16	CCR4	SLC6A19
**inflammatory cell**	KRTAP13-4	LCE1A	ANGPTL5	LCE2C	FAM180B	CCL19	LCE2B	CXCL14	CCL21	CXCL3	ADH1B	TNFAIP6	CFD	PLAC9	DCD	DPT	KRT1	LCE1C	APOD	CXCL2	HAS2	ABCA8	C1S	C1R	CCL2	FGF7	AGTR1	MXRA5	CTSK	GEM	DCN	LCE1C	CXCL12	MMP27	F10	IL11RA	LUM	PLA2G2A	MEDAG	SOD3	CCL13	FAM180A	KPRP	SFRP2	KRT14	RGS16	MT1M	PTGDS	CFH	IL6
**inflammatory macrophage**	SIGLEC8	FCGR2C	TMIGD3	FCGR1A	FCGR3A	FCGR2A	MS4A4A	VSIG4	OR52K2	LILRA1	SIGLEC14	MS4A6A	SIGLEC9	FPR3	SIGLEC10	SIGLEC7	MS4A7	LILRB4	CD209	FCN1	TNFSF18	LILRA2	FPR1	OLR1	TREM2	C3AR1	HLA-DQB2	FOLR2	CCL13	CD163	CCL4L2	NLRP3	LILRA5	GPR34	CLEC4A	LILRB5	OSCAR	CALHM6	BCL2A1	OR2T1	LIMS4	OR2B11	SDS	APOC2	GOLGA8T	CH25H	LILRB1	HLA-DOA	C5AR1	CLEC7A
**inhibitory interneuron**	SLC6A5	NPXH1	SLC35F4	DISP3	XKR4	SEPTIN3	MAGEC3	CNTNAP5	PRR35	EPHA8	OLFM3	SKOR1	ADARB2	ANO4	CACNG3	NXPH1	TLL1	GLRA2	CPLX4	CDH22	GRM3	MEGF11	MGAT4C	HCRTR2	PAX2	INSYN2A	SGCZ	GRIK3	UNC5C	ANOS1	DSCAML1	OR2L13	ZFPM2	LRRC38	ZNF521	AFF2	NKAIN2	DCC	KCNH7	GRID2	NETO1	GAD2	NRXN3	KIRREL3	ZNF385B	SIAH3	CSMD1	PRSS51	SGCD	GALNTL6

**intestinal crypt stem cell of small intestine**	CTLA4	LGALS2	CPS1	FGFR2	LGALS4	PIGR	PHKG1	CDK8	PRAP1	MAGEB18	KCNE3	REG3G	SPINK4	WDR89	SLC12A2	IL31RA	GPX2	YJEFN3	AKAIN1	NLRP6	ESRRG	NUP210L	VDR	MUC13	CAMK1D	ALDH1B1	FBXO47	SFI1	EEF1A1	DDAH1	ITGA11	SUMF1	BPIFC	OAT	CFTR	COX6B1	ZG16	EPO	PRR15L	TM4SF20	CFAP46	MYO1H	SEMA3C	PRDX1	PABPC1	DQX1	LRP1B	UQCR10	HES1	TFF3
**intestinal enteroendocrine cell**	GNG14	RXFP4	TMEM271	OR13C3	NLRP2B	ASIC5	AVPR1B	LCN15	UCN3	OR52N5	MLN	FEV	INSL5	GLYATL3	OR51A7	RFX6	PAX4	CRYBA2	LRRC53	SLC18A1	ADGRG4	FFAR1	OR2AG2	CYP2W1	OR51I1	NUTM2D	SCGB1C2	PRAC1	MBOAT4	TPH1	OR52N4	CCDC188	OR10A2	NKX2-2	SMIM32	NKX6-3	VWA5B2	KRT40	AMER3	TEX55	KRTAP5-10	REG4	PRR23C	LMX1A	RPRML	GUCY2C	MS4A8	TAS2R43	TGM3	INSM1
**intestinal epithelial cell**	ENPP7	BNIP5	MS4A18	BTNL3	CYP2C19	MUC17	GIMD1	PVALEF	MGAM2	KRTAP13-2	BTNL8	ANKRD40CL	OR4K14	SLC35G4	UGT2B17	MOGAT3	OR5M9	OR5H2	FABP2	ALPI	RBP2	ISX	OR5F1	ADGRG7	HNF4G	PRAP1	NXPE1	CYP2W1	GUCY2C	SNORC	ARSL	TM4SF20	APOA4	URAD	OTC	DEFA6	UGT2A3	CPS1	REG1A	MALRD1	FABP1	CHP2	SLC39A5	LCT	OR4L1	CDH17	MAMDC4	DMBT1	SLC28A1	CAPN14
**intestinal tuft cell**	HTR3E	HTR3C	OR6B1	TAS2R60	SH2D6	SH2D7	GNAT3	TRPM5	OR13A1	HMX3	TAS1R3	RGS13	BNIP5	IL37	ALOX12B	BMX	LPA	PLA2G4D	ALKAL1	POU2F3	ITPRID1	KLK13	KRT2	AVIL	TEX45	OR2A7	IL17RB	NCMAP	KRT40	CLRN3	MGAM2	SIGLEC16	ANXA13	IL25	GNG13	IRAG2	AZGP1	HMX2	OVOL3	G6PC1	CLEC17A	NPIPB8	KCNQ4	HPGDS	FFAR3	KLHDC7A	GRAPL	DIPK1C	FCRL2	AOC1
**intestine goblet cell**	MUC2	TBX10	SLC35G4	CLCA1	ITLN1	OR4D11	NXPE1	KRTAP10-6	OR1J2	B3GNT6	IFNA21	RETNLB	TPSG1	REP15	PRAC1	OR4K2	FCGBP	OR5F1	OR5B3	OR1S2	SPINK4	OR4C12	REG4	OR51G2	OR10Q1	OR52I2	KRTAP5-3	KLK15	CAPN9	OR10T2	OR13C2	ST6GALNAC1	ZG16	FRG2	OR2A2	OR5AK2	OR4C13	ENTPD8	BTNL3	FFAR4	TRIM64B	PRAMEF2	OR1N2	OR2V1	OR7G3	TAAR5	SMIM31	LCN15	ANO7	CEACAM5
**ionocyte**	ASCL3	BSND	FOXI1	TMPRSS11E	BPIFA2	FOXI2	TMEM61	KRTAP5-9	CLCNKA	DMRT2	PKD2L1	CLCNKB	MFSD6L	SRARP	KRTAP5-8	ASCL4	CLNK	SCNN1B	CEL	FAM131C	CARD18	CFTR	ATP6V1G3	FBXW12	PNMA6A	HEPACAM2	MUC20	ALKAL1	SCNN1G	ZNF750	STAP1	ATP6V0D2	ATP6V1B1	GPHB5	FAM153B	DGKI	ADGRF1	KIAA1210	NXPH2	GAS2L2	MUC16	CYP26C1	FAM3B	ATP6V0A4	CNGA2	FAM24B	SLC35F3	PMCH	RTP2	FYB2
**j1**	TRIM43B	PSMB11	TMC2	TRIML1	XCR1	FGF4	SLC10A1	IHO1	LRRC14B	PLA2G4B	NPHS1	CHRNB4	PRAMEF12	PRAMEF17	ZXDA	KCNN1	SLC17A8	LRRC2	EFCC1	FOXR1	RBMXL2	H2AC1	LRRC3	PLA2G2D	ZFP42	GJE1	H2AC21	CNTN2	APOF	SOAT2	SLC6A2	TRIML2	RIPPLY3	NODAL	LRRC8E	CCDC78	ESRRB	IL5RA	GDF3	MC1R	MORC1	PADI4	CTCFL	KREMEN2	MCIDAS	PRKAG3	TDGF1	PTCRA	PROX2	LGR5
**jhh7**	UGT2B11	CYP2B6	UNC93A	FGG	ASGR2	FGB	AFP	THPO	FGF19	AHSG	CCL20	SPINK1	RBP4	INHBE	MATN3	TRIM15	AMBP	MAGEA12	ORM2	ALB	HNF1A	CYP1A1	TNFRSF8	TDO2	CSAG1	MAGEA3	BAAT	VIL1	NR1H4	DUSP9	CARD18	NTS	FGL1	AGMAT	SERPINA6	UGT2B7	FGFR4	CHST13	DNAJC22	CENPV	CRYBB2	SLC51B	BTBD16	MASP1	CFHR3	TRIM31	GOLT1A	KLB	MOGAT3	KISS1R
**jurkat**	OR12D1	PRAMEF33	USP17L19	USP17L21	USP17L25	USP17L26	OR52E1	USP17L29	USP17L28	USP17L27	OR4K3	USP17L24	BPY2B	KRTAP23-1	USP17L5	USP17L13	OR13C7	BPY2	LCE4A	PRR20B	PRR20A	PRR20D	OR2T1	USP17L10	KRTAP9-8	OR10J4	KRTAP10-12	PRAMEF7	OR7G1	DEFB107A	OR2J1	OR5D18	OR4C5	USP17L17	CDY1	KRTAP10-9	KRTAP10-1	OR51S1	CDY1B	DEFB130A	OR1A2	KRTAP10-5	OR7G3	KRTAP10-3	MBD3L4	OR6S1	IFNA8	PRR20E	OR13F1	IFNA10
**k562**	OR52J3	OR51Q1	OR51B2	HNRNPCL4	RHOXF2B	OR51B4	OR51I1	OR51B6	CTAG2	PPIAL4E	OR52D1	OR51B5	XKR3	RHOXF2	HBZ	SSX1	MAGEB17	OR10A4	HBQ1	GYPB	PYDC2	SAGE1	MAGEC1	VHLL	CT45A10	SSX4	SSX2B	MAGEB1	GP1BB	PAGE1	PRAME	MS4A3	HBG1	GYPE	SSX3	EEF1AKMT4	MEIOB	RHAG	GTSF1	AQP10	PNMT	OR51I2	TERT	KCNH2	CTCFL	GATA1	HBE1	HBG2	NMU	GAGE1
**kals1**	SPANXC	SPANXD	KRTAP1-1	IL11	IGFL3	NPPB	CD70	KRT81	POU3F2	KRT33B	SPOCD1	TMEM158	DCAF4L2	KRT3	MAGEC2	CGB2	CCL26	RTL3	FOXD1	KRTAP2-3	NLGN4X	APCDD1L	COL8A1	FOXG1	INHBA	ALPK2	HS3ST3A1	OR5P2	CDKN2A	LHX8	VCX2	FLG	CNN1	CGB1	MAGEA12	PDCD1LG2	TENM2	SUN3	KRT34	CSAG1	ULBP2	ADAMTS6	SPOCK1	FGF5	EDIL3	ONECUT2	DIO2	BDNF	NDP	ZNF583
**keratinocyte**	CLDN17	LCE1D	KPRP	LCE1B	KRT35	DSG1	SDR9C7	DSC1	LIPM	LCE1F	FLG2	THEM5	MT4	WFDC5	KRT77	KRTDAP	KRTAP3-3	KRT2	KRTAP3-2	LCE3C	CASP14	HRNR	LCE1C	PSAPL1	KRTAP13-1	LIPK	KRT28	SERPINB12	AADACL2	CCL27	SLURP2	LCE1E	IL36RN	CLEC2A	KRT84	KRT1	KLK9	KRT6C	FAM83C	KRT24	LGALS7B	DYNAP	SPRR2G	RXFP3	TBC1D3E	KRT36	VSIG8	KRT16	CHIT1	SPRR2A
**keratinocyte stem cell**	OCM2	FAM25A	CCL27	TGM5	KRT71	PPIAL4D	THEM5	KRT24	KRT79	LIPM	LGALS7	SERPINA11	KRTAP3-3	TGM7	PSAPL1	KRT1	WFDC5	KRTDAP	SBSN	IL36RN	KCNK7	ANXA8	IFNK	RNF39	IL31RA	CALML3	CASP14	DSG1	KRT15	KRT77	GJB4	TGM3	KRT25	CCL1	KRT5	BNIPL	PGLYRP3	COL17A1	KRT17	SERPINB12	CAPNS2	DSC3	SERPINB2	SERPINB5	NLRP10	DSG3	EPGN	TRIM29	WNT16	LHX2
**kidney capillary endothelial cell**	CD300LG	DKK2	SOST	RTP3	CLEC1A	PLSCR2	ARHGEF15	PI16	EMCN	FAM167B	TMEM26	SRMS	MMRN2	GPIHBP1	EHD3	GATA5	CYP4B1	CYP26B1	CDH5	USHBP1	FAM110D	ENDOU	DLL4	CLEC14A	GIMAP5	PHOSPHO1	UNC45B	RASIP1	SOX17	IP6K3	MYCT1	TEK	TIE1	KDR	LRRC3B	LY6G6D	RIPPLY3	TBXA2R	PTPRB	SOX18	BCL6B	MIOX	SLC34A1	PLAT	SMAD6	HLX	SHE	RAMP3	ECSCR	GJA4
**kidney collecting duct epithelial cell**	KRTAP19-4	AVPR2	AQP2	KRTAP6-3	CDH16	KCNJ1	SLC34A1	BSND	UMOD	FXYD4	MIOX	UGT3A2	CLDN8	TRPV5	S100G	SLC34A3	CLCNKB	SPINK8	SLC5A2	ATP4A	SPP2	CLCNKA	SCNN1G	GPX6	APELA	WNT9B	SLC22A8	SLC22A12	TMEM174	LY6L	TMEM229A	MYMX	TMEM213	TLDC2	CLTRN	PAH	GLYAT	RNF183	SLC6A19	RNF186	ALDH8A1	SLC17A1	SLC5A12	FXYD2	RHBG	DCLK3	CNTF	SLC6A18	OXGR1	KCNE1
**kidney collecting duct intercalated cell**	AQP6	ATP6V1G3	SPINK8	TMEM213	OXGR1	RHBG	CLCNKB	FOXI1	HMX2	ATP6V0D2	BSND	SLC26A4	TMEM52B	ATP6V1B1	CLDN8	SLC12A3	INSRR	SLC4A9	GLOD5	GPX6	UMOD	CDH16	KCNJ1	ATP6V0A4	GUCA2A	ATP6V1C2	ALDH1L1	EGF	RHCG	GCGR	ATP4A	DEFB1	TMEM61	SLC12A1	KLK1	CLCNKA	CPSF4L	GLYAT	SLC4A1	TMEM72	PLET1	TTC36	PAH	SLC34A1	SPRR2G	S100G	HEPACAM2	TFCP2L1	ASB15	AVPR1A
**kidney collecting duct principal cell**	AQP2	FXYD4	AVPR2	SCNN1G	TRIM49C	KCNJ1	SPINK8	CDH16	DEFB125	WNT9B	FRMPD4	SIM1	SCNN1B	ATP4A	HOXD3	CLDN8	PAX2	OR7A10	APELA	BSPH1	PIK3C2G	MALRD1	KCNJ16	TMEM213	CLCNKB	HSD11B2	KCNH5	EMX1	FYB2	S100G	PDE8B	TRPV5	CDKL1	KCNE1	KCNC2	SLC12A3	PTGER1	SCGB1C2	RANBP3L	PKHD1	STYK1	KCNQ1	MGAT4C	RHBG	BMPR1B	ATP6V0A4	BSND	PRR35	IQCM	GREM2
**kidney connecting tubule epithelial cell**	TRPV5	SCNN1G	PLA2G3	OPRM1	AQP2	KCNJ1	TMEM52B	SIM1	KCNJ16	BSPH1	AFM	TRIM43	FRMPD4	SLC2A9	MALRD1	ATP6V0A4	ADAMTS16	SCNN1B	TMEM178A	TRPM6	HOXD3	IQCM	EMX1	KCNQ1	SLC12A3	KRT37	CABCOCO1	TRABD2B	NR3C2	SHROOM4	MROH5	MAGEB18	HOXC5	PHACTR1	GPR39	LRMDA	CALB1	KLHL3	SLC8A1	ARL15	PLCL1	CCDC83	KCNC2	SVOPL	RHCG	WDR72	ARHGEF38	COL4A3	PDE8B	SLC16A5
**kidney cortex artery cell**	SOX17	DHH	CLEC1A	PLSCR2	GJA5	GJA4	EGFL8	SRMS	FAM110D	MMRN2	FAM181B	USHBP1	SLC34A1	SHE	LRRC32	BCL6B	ADGRL4	ARHGEF15	CDH5	ECSCR	BMX	SOST	IP6K3	RSAD2	CLEC14A	NOS3	DKK2	MIOX	TIE1	MYCT1	RTP3	C1QTNF9	PTPRR	PTPRB	EMCN	ADCY4	RASIP1	TCF15	CLDN5	DLL4	ADGRF5	PI16	CD93	TEK	CLIC5	FGD5	ST8SIA6	CLDN15	APOLD1	SOX18
**kidney cortex collecting duct principal cell**	IL36B	PTH2R	PIK3C2G	AQP2	OR2T10	ZPLD1	OR4F15	SCNN1G	KCNIP4	OR2A14	SEMG2	AGBL4	SLC5A12	DCDC2	KCTD8	ZNF98	UGT2B28	OVCH2	NPAP1	FRAS1	NUTM2F	SLC17A1	CCSER1	SCN2A	CCDC178	RALYL	TRPM3	PDE10A	NAALADL2	TRHDE	WDR72	KCNJ16	PAPPA	IL1RAPL2	STPG2	KSR2	BMPR1B	SLC28A1	ZNF208	NR3C2	KCNJ15	ERBB4	ADAMTS16	PDE7B	TRPV5	TBL1Y	NLGN1	FAAH2	MYO3B	ABTB2
**kidney distal convoluted tubule epithelial cell**	SLC12A3	UROC1	TRPM6	KCNJ1	EMX1	ABCA13	LRRC52	TMEM52B	EGF	AFM	FCAMR	UMOD	ATP4A	SIM1	PLA2G3	CLCNKB	CWH43	BSPH1	KLHL3	TMEM213	BSND	ESRRB	KCNJ16	S100G	TMEM72	GPX6	RGS6	PKHD1	TRABD2B	PTGFR	ATP6V0A4	TARM1	IQCM	COL4A3	MALRD1	WNK4	RTL4	CLCNKA	CDH16	CABCOCO1	DEFB1	MROH5	STK32B	TBPL2	COL4A4	OPRM1	GCGR	PAQR5	MYO3B	PVALB
**kidney epithelial cell**	SULT1C3	LY6L	PNPLA5	PPIAL4D	GSTA5	PRR27	AMY1A	TRPV5	SNORC	OPRPN	HIGD1C	SLC22A25	OTC	SLC12A3	CABP2	OR2T2	APOC4	AGXT2	HPD	OR2T10	HAMP	ASPDH	SERPINA11	GLYAT	AKR1D1	SLC17A2	WFDC10A	ALDH8A1	CSN1S1	APOC2	SLC25A47	GBA3	LALBA	TAT	PLA2G3	LECT2	SAA4	FETUB	APOA5	BHMT	ANGPTL8	SMLR1	TMEM207	DPYS	BAAT	UROC1	GLTPD2	APOC3	UGT2B15	SDR9C7
**kidney granular cell**	NCR1	KLRC2	QRFP	CMA1	KLRK1	TXK	CCR5	GZMA	HEATR9	IL18RAP	XCL1	NEURL3	PRF1	IL2RB	IFNG	CCR2	KLRC1	S100G	KLRD1	TBX21	EOMES	CCL8	CD244	CCL5	SLC34A1	ADAMTS14	UMOD	GZMB	KRT83	NKG7	CYSLTR2	GPR174	CCL4	IL12RB2	IL18R1	CD160	RINL	STAT4	SYTL3	SH2D2A	STYK1	PTPN22	IRF8	CTSW	SPP2	SLC7A13	HSD11B1	CXCL9	MIOX	GIMAP8
**kidney interstitial cell**	IL36B	AADACL4	OR4F15	PTH2R	DUXB	HPSE2	SLC17A1	ARSH	UGT2B11	SLC13A1	NLRP13	AQP2	RERG	UGT2B7	TBL1Y	SLC2A9	COL4A4	SLC5A12	HAVCR1	LAMA2	GOLGA8T	SLC14A2	CCDC102B	PRKG1	SLC47A2	CTAGE6	SPANXA1	PRSS51	KCNIP4	STPG2	SVEP1	NLGN1	SLC17A3	GBA3	SLC16A12	A2M	PRR16	MUC19	PLCXD3	KRT39	ADH1B	COL4A3	SLC22A2	NAALADL2	ABCA6	NLGN4Y	PRKD1	SLC12A1	SLC22A24	SYNPO2
**kidney interstitial fibroblast**	GGTLC3	SULT1E1	AMY2A	AMY1A	COL21A1	AGTR2	SHISA3	RPEL1	GOLGA8R	NEUROG2	SPIN2A	ANKRD62	ATOH7	AMY1B	HIGD1B	PTPN20	SIGLEC11	PRSS35	FAM43B	PCDH18	TCEAL7	TCF21	ZNF391	TRPC6	HTR2B	ZNF534	KCNE4	HOXA6	PLAC9	FOXF2	OGN	GPR20	FAP	ZNF229	HSPA6	REN	FKBP1C	FOXS1	FZD10	ALX1	ZNF624	ADAMTS18	LRRC17	SLC2A14	MXRA5	CNTNAP3	ZNF649	KCNJ8	KCP	TECTA
**kidney loop of henle ascending limb epithelial cell**	BPY2C	UMOD	TMEM207	DEFB125	PKHD1	EGF	GPX6	TRIM49C	SLC12A1	KCNJ1	CLCNKA	LRRC66	DEFB107B	SPDYE11	CASR	POTEA	CLDN19	SLC9A3	TARM1	RGPD2	ADGRE3	ESRRB	ABCA13	OR4C3	CLCNKB	RANBP3L	GCGR	OR2F1	NPIPA2	HNRNPCL2	TRIM51	TMEM72	SLC22A14	CDH16	BSND	TBL1Y	OR7C1	PTGER3	TBC1D28	KCNJ16	SIM1	MYO3B	GJA10	COL4A3	LRRC31	SIM2	CTAGE15	NR1H4	ATP6V0A4	STK32B
**kidney loop of henle cortical thick ascending limb epithelial cell**	TARM1	SLC9A3	PLA2G3	TMEM207	ESRRB	LRRC31	BSPH1	MYO3B	RTL4	STK32B	EGF	KCNJ1	SIM1	KCNJ16	CASR	AFM	IQCM	SLC12A1	SLC16A5	IL7	CCDC83	SLC5A1	ABCA13	CACNB4	GCGR	CABCOCO1	MAGEB18	MROH5	PDGFD	PAQR5	RGS6	SHROOM4	COL4A3	ENOX1	PPARGC1A	AGRP	OPRM1	ATP6V0A4	ERBB4	TMEM72	HOXC5	CFTR	SLIT2	TEX50	LRRC66	IL34	ZC3H12B	DDO	PPEF1	ARHGAP6
**kidney loop of henle descending limb epithelial cell**	BPY2C	PKHD1	GBA3	TRIM49C	MYOCOS	DEFB125	ADGRE3	SPDYE11	OR3A2	DEFB107B	UGT2B11	UGT2B7	RGPD2	CTAGE15	SLC5A12	TBL1Y	NPIPA2	POTEA	SLC22A14	GOLGA8M	PAX2	SLC13A1	SLC17A3	MROH5	IGSF5	RIPPLY1	ZPLD1	NLRP13	TRIM51	ASIC5	TBC1D28	TBPL2	GALNT14	UGT2A3	RANBP3L	ITGB6	COL4A3	NXF5	RGS21	SLC22A2	OR9Q1	HOXC5	NR1H4	DACH2	HTR4	GLYAT	ZAN	SLC7A13	SLC9A3	CTAGE8
**kidney loop of henle epithelial cell**	MCCD1	SLC12A1	TRIM49C	UMOD	DEFB125	TMEM207	DEFB107B	OR11H4	OR7A10	KCNJ1	KRTAP2-2	CLDN16	CLDN19	OR2T35	CLCNKA	EGF	OR9Q1	BSND	CLCNKB	OR2T2	OR7C1	POTEA	SPDYE11	TMEM213	TMEM52B	OR14A16	GPX6	CCDC195	CDH16	HSFY1	TMEM72	CLEC18B	CASR	GCGR	POU3F3	NPIPB8	OR4E2	OR2A14	TRIM49B	SLC16A12	SLC22A24	SOSTDC1	PTGER3	MYOCOS	DAZ2	TRIM51	KNG1	OR1L6	ZNF705B	OR2L3
**kidney loop of henle medullary thick ascending limb epithelial cell**	SLC9A3	TMEM207	TARM1	MYO3B	SGCZ	ESRRB	BSPH1	SIM1	LRRC31	RANBP3L	KCNJ1	PLA2G3	PPEF1	CACNB4	IQCM	CCDC83	STK32B	LINGO4	PAQR5	AFM	MAGEB18	ELFN1	ABCA13	PPARGC1A	SHROOM4	IL7	IL34	KCNJ16	MTNR1B	PDGFD	PAX2	MROH5	COL4A3	CABCOCO1	AGRP	DDO	SIM2	RGS6	MALRD1	NMBR	CFTR	GPC5	TMEM72	ZC3H12B	SEPTIN14	MEF2B	CCDC148	ERBB4	HOXC5	FYB2
**kidney loop of henle thick ascending limb epit...**	SLC12A1	F11	PTH2R	UGT2B11	IL36B	CASR	PRAMEF26	CLCNKA	STPG2	CCDC178	KNG1	GPC5	FRG2C	PXDNL	SLC22A2	KCNJ1	CALCR	CLCNKB	EGF	RANBP3L	SLC16A12	TMEM207	ZNF98	CA10	KCNIP4	MCCD1	PRSS51	PTGER3	NELL1	ESRRB	PLCL1	ERBB4	TMEM72	CCSER1	STK32B	TBL1Y	SULT1C2	SIM2	NXF5	CCDC148	MYO3B	SCTR	CACNA2D3	GP2	CFAP221	SPANXA1	KCNJ16	CDH16	NAALADL2	PDE1A
**kidney loop of henle thick ascending limb epithelial cell**	FRG2B	FRG2C	UMOD	TMEM207	MCCD1	GPX6	SLC12A1	KCNJ1	CLCNKA	CLDN19	BSND	EGF	CLDN16	FXYD2	CLCNKB	CDH16	KNG1	NCCRP1	DEFB1	LRRC66	GCGR	TMEM72	PPP1R1A	SOSTDC1	TMEM213	FOLR3	ATP4A	TMEM52B	PTGER3	CLDN10	KLK1	PDILT	WNK4	RANBP3L	GLOD5	MFSD4A	KCNJ16	SGK2	SLC5A1	ATP6V1B1	PKHD1	BAIAP2L2	EGFL6	CLDN8	POU3F3	PAQR5	DUSP15	CDKL1	EFHD1	MAL
**kidney loop of henle thin ascending limb epithelial cell**	IL36B	UGT2B11	GGTLC3	ITGB6	UGT2B7	OR2A14	SLC12A1	PRAMEF26	SIM2	CDH16	SEMG2	SCGB2A2	PTH2R	NEK10	CACNA2D3	CA10	PRKD1	KCNIP4	RANBP3L	CFAP221	PAX8	CLCNKA	SEC14L6	DNAH5	AQP2	CASR	KNG1	OR2T10	SIM1	VCY	STPG2	ARHGEF38	SULT1C2	LCE3C	PDE1A	NLGN4Y	SLC6A12	RASAL1	FAAH2	ANKRD18A	ADGRF1	ZNF486	ERBB4	BICC1	MEPE	SPATA6L	CLDN10	SGK2	FAM83B	SCTR
**kidney proximal convoluted tubule epithelial cell**	SLC34A1	SPINK8	TMEM174	SLC22A12	SPP2	SLC34A3	MIOX	SLC5A2	KCNE1	OXGR1	TMEM213	FXYD4	ATP6V1G3	SLC17A1	SLC17A3	CLCNKB	UGT3A2	MMP12	SLC5A12	NEU2	SLC22A8	F13B	SLC13A1	DAO	S100G	HMX2	CDH16	FAM151A	CLTRN	GUCA2B	AQP2	SLC22A6	INSRR	AVPR2	SLC22A1	PAH	RDH16	GLYAT	BSND	ATP6V0D2	FOXI1	RHBG	AQP6	TTC36	SLC4A9	SLC6A18	SLC26A4	APELA	ALDH8A1	SLC6A19
**kidney tubule cell**	SLC22A13	KRTAP19-4	SLC7A13	SLC22A7	TMEM174	SLC6A18	SLC34A1	SLC17A3	SLC22A12	UGT3A2	UGT3A1	MEP1B	SPP2	SLC22A2	SLC17A1	ERICH4	TMIGD1	SLC34A3	ASPDH	AGXT2	MEP1A	SLC5A10	SLC22A1	TTC36	NAT8	SLC22A6	MIOX	ALDH8A1	CLTRN	PDZK1	MOGAT1	F13B	GUCA2B	CCKAR	GLYAT	SLC18A1	SLC23A1	SLC26A1	TINAG	GGT1	CLDN2	SLC5A2	HSD3B2	NLRP6	HOGA1	SLC13A1	LRRC19	SLC47A1	ALDOB	TCHHL1
**kns42**	EN1	GABRQ	WNT10B	LAYN	DIRAS3	SFRP4	PCDH20	CNPY1	DOK7	IGSF9B	NR2E1	BAALC	SYNDIG1	FAM86B2	EN2	DLX6	DOK5	ALPK2	PIPOX	GSX2	ADRA1D	GFAP	CDH6	NDP	LIF	TPRG1	CCL2	DLX5	FNDC4	GAP43	DBNDD1	CNIH3	KCNK12	TMEM198	CCL26	POU3F2	SOX2	BEGAIN	CRB2	TLR4	HRCT1	SH2B2	RFLNB	KCNJ6	CNR1	LRTM1	C1QL4	NPB	NGEF	DSC1
**kpl1**	SPTSSB	TFF1	SYT10	KCNK15	RNF223	KRT83	SPDEF	CALML5	BCAS3	HOXC13	SYT12	KRT80	EEF1A2	CIB3	LXN	ZG16B	CLEC3A	ADCY5	RNF224	FOXA1	TFF3	LMTK3	FIBCD1	GATA3	RBBP8NL	ZNF467	BMP7	TUBD1	CYP1B1	PARD6B	CCDC170	CCDC57	TRIM37	BCAS4	RAD51C	HOXC11	TBC1D30	USP6	SERHL2	LYPD6B	METRN	FBP1	MNX1	TBX2	MMEL1	VGF	F12	KRT86	MLPH	NMB
**kpnsi9s**	CLEC12B	TNFSF18	SPANXD	CACNG8	BDKRB1	IL11	APLN	PODNL1	MAGEC2	GREM1	SPAAR	STEAP1B	CCM2L	ZP1	ROBO3	CCDC68	CACNG6	ITGA10	PROCR	MAGEA10	KCNG1	LAYN	LYL1	NOS3	HMX1	SPOCD1	FABP6	GDF6	OR51B4	HTN1	OSCAR	SOX7	NOG	PPM1N	GDNF	CXCL6	INHBA	ANKRD18B	ASPHD1	HEY1	FGF5	HTR1D	MSC	SPATC1L	ZNF467	RNF212	TLX3	CSAG1	PLCD4	ZNF114
**kupffer cell**	CLEC4F	CD5L	CCL24	CD300LD	VSIG4	TIMD4	APOA5	CD207	FOLR2	TREML4	MARCO	CLEC1B	CETP	APOF	ANGPTL8	LILRB5	MMP27	CD163	PLG	ADGRE1	SPIC	C1QC	LILRA5	C1QA	C1QB	IL18BP	HPX	CXCL13	CFP	PZP	ITIH4	ALB	SIGLEC1	CYP8B1	MSR1	MRC1	CPN2	TMEM26	MPEG1	NR1H3	PAQR9	STAB2	CFHR2	CSF1R	LRRC25	FCN2	SDC3	ITIH1	CCR3	PROK1
**kyse180**	H1-1	RADX	HROB	ALPP	H3C11	H4C9	BLACAT1	FAM166C	ANXA8L1	CFAP300	H2AC7	CPA4	ABITRAM	MAB21L4	KLK5	RTL10	GARS1	B3GALT9	HOXC12	H3C1	STEEP1	EOLA2	MTARC1	OR1J1	IQANK1	SUSD2	KCNS1	CDIN1	SEPTIN3	EPRS1	MEAK7	PGAP6	WARS1	HARS1	OBI1	SEPTIN5	KLK6	TCHHL1	PACC1	H2AC14	VARS1	GPR148	YAE1	ESS2	KARS1	OLR1	MIDEAS	CCDC9B	H3C4	EOLA1
**kyse520**	LCE1F	LCE1E	SUN3	LCE3E	CALML3	IL13RA2	FAM83A	SPINK6	ARTN	CALB1	SLCO1B3	LCE1C	FOLR3	MAGEB2	KRT14	LAMA3	FST	NUTM2E	COL17A1	KRT6A	MAGEA10	FERMT1	LAMC2	MYH15	CYP4F11	APCDD1L	CFAP251	MAGEA1	ZBED2	SERPINB2	LY6K	LAMB3	EGFR	S100A2	HMSD	MROH6	HAS2	GJB5	AJAP1	KLHL35	ERVMER34-1	ESM1	PTHLH	SERPINB7	RHOD	MAGEA6	TGFA	MIB2	GJB3	KRT5
**l2/3-6 intratelencephalic projecting glutamatergic cortical neuron**	OR14I1	SMIM43	SLC22A10	ZNF735	PNMA6F	HTR1E	TBC1D3I	NXF5	KRTAP10-11	MCHR2	NWD2	SLC22A24	RFPL2	KRTAP9-2	CRACDL	OR5H15	PRB2	FAM237A	PNMA8C	TMEM132D	CNTNAP5	GRIN2A	VXN	TAFA1	ZNF804B	KCNV1	OR2L13	TMEM235	CBLN2	FAM153B	SLC17A7	HS6ST3	PABPC1L2B	CACNG3	KCNJ4	CDH12	HS3ST4	KCNH1	TMEM271	CDH9	LRRC7	ANKRD30BL	FRMPD4	CA10	LRFN2	GRM7	CDH18	MLIP	NEUROD6	FAM153A
**l1210**	NKX6-3	RNASE12	IFNA13	VPREB1	TAC4	BATF2	IGLL1	GPR152	CDCP2	LTA	DCDC2C	CD19	ZBTB32	LHFPL1	KRTAP5-1	SAPCD1	CXCR2	SLC6A19	GALR3	TLR9	PKD1L3	VPREB3	PAX5	ESPNL	TMEM119	SMPDL3B	APOBEC2	ADGRG3	DCLK3	OMD	MPL	ZFP69	CSDC2	ULBP1	CCDC116	TRPA1	LPAR5	CTSE	TNFRSF13C	NANOG	PTGIR	SCN4A	EMID1	MZB1	ALPL	POU2AF1	CHRNA1	PXT1	TTLL11	TYR
**laml**	RNASE3	DEFA4	CEACAM8	DEFA3	EPX	BPI	PRG3	RNASE2	MS4A3	PRSS57	AZU1	CXCR1	CEACAM4	CEBPE	CTSG	ELANE	RGL4	MMP8	RAB44	SERPINB10	SMIM24	RETN	PADI4	HBD	MCEMP1	VSTM1	PRAM1	PROK2	CSF3R	CPA3	CCL1	CRISP3	GAPT	IGLL1	SLC22A16	FOLR3	NFE2	S100A12	GFI1	FLT3	CCL23	SIGLEC12	PRTN3	CRYGD	AHSP	MNDA	TPSB2	CLEC12A	FCN1	TNFSF13B
**lamp5 gabaergic cortical interneuron**	LAMP5	CPLX3	NMBR	HCRTR2	GPR151	NPY	CORT	TRPC5	MPPED1	NXPH1	GABRA1	PNOC	ADRA1A	GPR12	ZMAT4	GABRD	RTL4	CLRN2	CPNE6	GAD2	GABRB2	NYAP2	SLC6A1	VSTM2A	PDE11A	TRIM17	FRMPD4	GAD1	GPR6	GABRG3	RXFP3	GRIK1	GABRA5	NRSN1	ADARB2	GRIN2A	DISP3	NT5C1A	RESP18	CYP46A1	SLC35F4	ST8SIA5	ATP6V1G2	RTL9	KCNS2	CRTAC1	GABRA4	RXFP1	SGCZ	KCNIP1
**langerhans cell**	CD207	KRTAP3-3	KRTAP13-1	CCL22	MT4	LCE3B	DCSTAMP	KRTAP1-3	KRT36	CCL17	KRT35	KRT84	LCE3C	P2RX2	KRTAP3-1	DEFB103A	IL1R2	ZMYND15	KRT85	GPR141	DPEP2	NLRP3	SERPINB12	IL36A	LCE3A	KRTDAP	KRT4	IL12B	OCSTAMP	MMP12	TNIP3	KRT33A	CD80	THEM5	CD86	LORICRIN	KRT76	FAM25C	TAMALIN	KRTAP3-2	LGALS7	SNX20	GPR132	CD72	KRT24	SLC15A3	SLC36A2	KRT16	HAVCR2	CCR1
**large intestine goblet cell**	TPSG1	GAL3ST2	HGFAC	CHST4	SPINK4	SYCN	ZG16	ATOH1	GUCA2A	A4GNT	REG4	REP15	B4GALNT2	GPR20	RNF186	B3GNT6	RETNLB	ERN2	PLA2G10	KLK1	ENTPD8	PLA2G2F	HPD	KRT85	HOXB13	DIO1	GCNT3	FCGBP	FAM83E	IHH	CAPN9	RNF39	BEST2	SERPINA9	HEPACAM2	P2RY4	FBP2	ATP2C2	MYO1A	EPS8L3	MUC13	FABP2	LRRC66	CD177	B3GALT5	FOXA3	SPDEF	ADGRG7	CLRN3	ISX
**late pro-b cell**	PRG3	OOSP1	RAG2	PRG2	FCRLA	VPREB3	POU2AF1	CD19	P2RX3	CACNA1S	BFSP2	BLK	MEF2B	ARL5C	VPREB1	TNFRSF13C	IGLL1	CD79A	SLAMF6	NIBAN3	CD79B	SLAMF7	BHLHA15	DOK3	SPIB	ULBP1	BLNK	CD72	PYHIN1	MYL4	TNFRSF17	APOBEC1	JCHAIN	MTARC2	GPR12	CECR2	KLHL6	TIFA	ELANE	IRF4	B3GNT8	NEIL3	CHST1	LY6D	CHST3	SPNS3	TREML2	IRAG2	IL12A	RAB43
**lateral mesodermal cell**	WNT8A	MSGN1	HAND1	PITX3	OR52M1	CCN6	SPRR5	GPR101	KRTAP12-2	FAM47A	BSX	OR6T1	TAS2R43	TUBA3C	OR13H1	TAS2R3	NPBWR2	KRTAP4-8	KRTAP4-7	FTMT	TAS2R7	IL17REL	PNMA6E	TAFA3	TAS2R8	TAS2R13	DSPP	TAS2R9	GOLGA6L22	RHCG	NMRK2	CCDC105	CYP26C1	PROZ	BHLHA9	ZP4	OPTC	NKX1-1	NTSR1	FAM240B	APLNR	MMP3	LRIT3	GATA5	LRRC72	RLN3	SLITRK2	MYOG	OR13C8	CCNB3
**lcml**	LRRC70	GOLGA8T	CCDC27	PPIAL4G	OR2T8	GOLGA8S	GOLGA8G	HNRNPCL2	OR2G3	NANOGNB	H3-5	MPL	TAS2R60	OR2L8	OR2AK2	UBE2NL	OR2T33	OR2L3	OR2L5	OR13D1	CRHBP	GOLGA6L6	DYTN	PRR23C	OR1S2	SPINK2	USP17L3	OR13C2	SMIM17	CDC14C	CRYGD	GOLGA6C	MAGEB10	FCAR	PGAM4	USP17L4	VN1R2	OR13C5	DUXA	OR52N4	AKR1C4	USP17L1	IFNA21	SPDYE8	ERVV-1	OR10A5	OR2M5	CYTL1	GCSAML	USP17L10
**leptomeningeal cell**	OR4P4	SLC22A6	SLC6A13	ZIC4	FAM180A	TBX18	RANBP3L	ATP13A5	ZIC1	SLC22A8	GJB6	SLC47A2	LAMC3	RTL4	CEMIP	SLC13A4	EDN3	SLC47A1	SLC30A10	BMP6	ITIH2	IL31RA	ATP1A2	ZIC5	APOD	CPED1	PRDM6	COL4A6	UNC13C	AMY1C	COL13A1	ALDH1A2	COL26A1	TRPM3	HOXC4	GPR182	AGMO	KCNK2	LAMA1	SLC7A11	ITIH5	ABCA9	GJB2	NTRK3	FOXD1	WFIKKN2	ALX3	CLEC3B	COL4A5	SLC38A3
**leukocyte**	TMEM275	PRORY	SPEM3	TEX55	SLC35G6	MAS1L	SPATA46	TNP2	KIR2DS4	MPC1L	CSTL1	KIR3DL3	CFAP97D1	DPEP2NB	CLEC18C	FAM24A	CCDC185	TEX50	MTNR1A	CD300LD	IQCA1L	MS4A13	ADGRE1	CCDC197	FNDC11	GLT6D1	H3C11	CCDC179	CCL24	SCP2D1	ARMH2	TSSK1B	IQCF1	H4C7	H2AC14	LLCFC1	IFITM5	ETDB	TEX48	SPEM2	PROP1	ODF1	CCIN	FCGR3B	TMCO2	PF4	CLEC4D	ABHD16B	TAAR5	CCDC196
**leydig cell**	HSD17B3	TEX55	PROK1	SPEM3	INSL3	ZC2HC1B	SLC35G6	PRM1	TNP1	SPATA46	TNP2	NUTM2B	ARMS2	SPPL2C	KRT9	CYP17A1	CCDC179	TEX50	CSTL1	GOLGA6L2	PRM2	MPC1L	ZNF479	CCDC70	TSSK1B	TRIM42	HMGB4	KIF2B	FAM24A	CCDC185	IQCF1	ARMH2	SPACA3	CBY2	CLEC18C	SCP2D1	MS4A13	GALNTL5	CFAP97D1	DNAJB8	TMCO2	RNF151	TPPP2	SPEM1	CYLC2	CMTM2	TSSK2	TEX29	ABCA8	CCDC54
**lihc**	PRAMEF4	ANGPTL8	CYP2A7	UGT2B10	CFHR5	SLCO1B1	CCL16	ITIH1	CYP4A11	AGXT	CYP2D7	AHSG	UGT2B4	PLG	CYP2A6	HPR	CYP2D6	INHBE	CPN2	SLC22A9	CYP8B1	CYP2C8	APOC3	SERPINA11	SLC22A7	BAAT	CYP3A7	MAT1A	CYP7A1	APOB	KNG1	APOH	FGA	ADH4	SERPIND1	CYP4A22	SULT2A1	ORM2	CFHR2	HAO1	ORM1	AKR1C4	SLC17A4	VCX2	SERPINC1	APCS	ITIH3	HPX	SERPINA7	CPB2
**lipfibroblast**	SIGLEC5	PLET1	CD300LD	CIDEC	SLC9A4	MRC1	OLR1	ITGAX	MPEG1	CD300LF	LTC4S	AMZ1	FFAR4	MMP12	CYBB	SLC15A3	OSM	FPR2	FCGR3B	BST1	LILRA5	SLAMF9	TLR2	TIFAB	ABCG1	ARL11	CSF2RB	SLC39A2	HTR2C	CD200R1	LPL	TREM2	TFEC	DPEP2	RUFY4	SLC6A4	PALD1	SBK2	PIK3R6	TLR7	KCNN3	ABCD2	CD274	SPI1	GPR55	MERTK	C5AR1	LY75	UCP3	TLR8
**lmsu**	SIGLEC15	MAGEA4	STEAP1B	OR2T8	MAGEA6	FOXL2	TRIML2	HS3ST3A1	HOXD13	LETM2	SERPINB7	MAGEA10	APCDD1L	MAGEA3	NKX6-1	PAGE5	NPFFR2	LY6K	CSAG1	EGFLAM	SMIM10	MAGEA12	MYO7B	TRIM58	HOXD11	SUN3	KCNMA1	BDKRB1	RFX8	FOSL1	FHAD1	CXCL5	CREB3L1	GDNF	AMIGO2	VEPH1	ARSI	DYNC2H1	ANKRD30A	CGB7	FGF5	EVA1A	ADAMTS6	FOLR3	TWIST1	YAP1	PRR16	ARHGAP22	BIRC2	CGB2
**long term hematopoietic stem cell**	MPL	DCDC2C	GJE1	TNIP3	ITGB3	SELP	MMRN1	GPR84	GBP6	TREML2	TRPC6	AKR1E2	F2RL3	NKX2-3	SLC18A2	PGLYRP2	TFR2	CXCL11	PEAR1	ART4	IRGM	ZFP92	PADI4	GRK4	SCARF1	ADGRL4	MUC13	NEURL3	TRAF1	GPR25	CX3CL1	VWF	CYSLTR2	LHCGR	GBP7	SOAT2	MYCT1	GJC3	TAS1R1	NWD1	ANKRD63	ADGRG1	KIT	PDCD1LG2	CXCL10	LIPI	RSAD2	PLCB2	CXCR1	TYR
**luad**	VCY	VCY1B	CSAG3	GGTLC1	SSX5	MS4A15	SFTPA2	XAGE1A	SFTA2	SFTPB	SLC22A31	GAGE1	IL37	SCGB3A2	SFTPA1	AMY1A	H2BC13	AMY1B	PPP4R3C	CT83	OR51B6	ANKRD62	XAGE1B	PAEP	GNAT3	MUC21	H2AC18	MAGEA9	DEFB4A	SPINK9	LGSN	SLC34A2	C4BPA	H2BC5	KRTAP3-1	CHIA	GAGE12C	EOLA2	SSX1	PAGE2	H2AC7	CEACAM6	KRTAP4-1	OR52N1	CSF2	H4C12	NKX2-1	NPIPA7	SDR16C5	GAGE12F
**luminal cell of lactiferous duct**	MRGPRX3	SERPINB4	MGAM2	OR5P2	IL20	SERPINA3	SERPINB7	S100A7	FDCSP	CYP24A1	CHI3L1	SAA4	DEFB4A	H4C15	TAS2R42	PI3	CHI3L2	SERPINB3	LTF	AZGP1	TMEM265	KRT6B	MMP7	CFB	RARRES1	SAA2	SCGB2A2	OLFM4	BBOX1	NPIPA3	GOLGA6L1	FRMPD1	RGPD6	OR2A7	VNN1	PLEKHS1	S100A8	HTN1	RGPD5	CXCL8	WFDC6	KYNU	MUCL1	ANKRD30A	TNFAIP6	SERF1B	AKR1B15	CYP1A1	SAA1	CTAGE8
**luminal cell of prostate epithelium**	KLK3	KLK2	KRTAP9-1	OPRPN	OPN4	KRTAP8-1	SCGB2A2	KRTAP13-2	SRY	IFNA7	TGM4	KLK4	SCGB1D2	IFNA4	IFNA5	USH1G	PATE4	SNORC	IL9	CD8B2	IL12B	CHRNA7	KRT2	SMIM31	IGFL4	TINAG	PABPC1L2B	GSDMA	ANKRD30A	RFPL2	PLA2G2A	OR51E2	TACR3	PRAC1	GLB1L3	HOXB13	NKX2-8	MT1HL1	FUT4	NKX3-1	ALOX15B	LMAN1L	CST9	GUCY2D	KRT36	PRAC2	FOLH1	GP2	PLA2G4D	PNLIPRP1
**luminal epithelial cell**	BTN1A1	LALBA	IFNL3	WFDC12	GJE1	TMPRSS13	SMCO1	IGFALS	SLC28A3	PLET1	H2BC1	MUC15	R3HDML	HHIPL2	IFNK	LIPM	LYZL4	THRSP	TPH1	DCDC2C	TREX2	CEL	CHRDL2	TYR	ADIG	CYSRT1	LIPK	UBD	DKKL1	TMEM265	ELF5	FAM25C	BCL2L15	PRR32	RNF225	WFDC3	RSPO1	SOAT2	PLB1	GJB4	INAFM2	CXCR2	TTPA	TRIM43B	GJB6	LDLRAD2	FGG	CD164L2	DUOXA1	CAPSL
**luminal epithelial cell of mammary gland**	AMELX	SMIM38	AMBN	MMP20	AMTN	R3HDML	BTN1A1	SCGB2A2	KLK4	ADGRF4	CSN3	OR2A1	ODAM	MUCL1	CSN1S1	ANKRD30A	WFDC12	PRLR	TMPRSS13	HHIPL2	SLC5A5	H2BC4	LALBA	PLB1	FAM25C	SLC28A3	H4C15	CSF3	SLC39A2	CLCA2	CTAGE4	SLC5A7	TPH1	IFNL3	ATP6V1B1	ESYT3	CSN2	CER1	ELF5	PLET1	TEX11	DKKL1	PGR	FCGBP	IGFALS	ARHGAP40	MUC15	H2AC7	SP6	H2BC14
**lung bud tip**	SPDYE11	OR5H6	POTEI	SHH	POTEF	RGPD6	TCP11X2	TAS2R7	CYP4F8	OPRPN	CA5A	MAGED4B	OR52H1	HTN1	GOLGA6L1	KRTAP9-6	TBC1D3L	H2BW2	POTEE	FREM2	ZNF286A	ULBP1	IGDCC4	B3GALT1	SULT1B1	RGPD5	ZNF177	GOLGA6B	FSIP2	SCUBE3	ADAMTS19	PRDM7	NKX2-1	TAS2R10	RFX6	TCF23	PRRG3	ERVW-1	CA14	NPIPB13	GOLGA6L9	PABPC5	NPIPB12	LAMA1	NR1I3	OR7D2	ZNF284	ZNF556	KLLN	SPDYE16
**lung ciliated cell**	CFAP97D2	CFAP299	CROCC2	CFAP47	CLEC19A	RGS21	MROH9	SLC9C2	DNAI2	LMNTD1	WDR49	DNAH3	AADACL2	RP1	CFAP73	CFAP157	ADGB	DNAH12	CCDC187	DTHD1	CFAP46	DCDC1	KIAA2012	ECT2L	CFAP99	DNAI1	ANKFN1	TTC29	DNAH7	LDLRAD1	PRRT1B	SLC9C1	GOLGA8J	ODAD2	ZBBX	DNAH9	DNAH6	NEK10	SMIM35	CFAP54	DNAH10	CFAP58	TMEM232	CFAP61	SNTN	CFAP77	VWA3B	ERICH3	FRMPD2	VWA3A
**lung endothelial cell**	ANO2	CC2D2B	SLC5A4	VWF	SNTG2	TLL1	SELE	PKHD1L1	GSG1L2	OR2A14	BMERB1	PALM2AKAP2	RXFP1	OR2A42	CNTNAP3C	ERICH4	SLCO2A1	GNA14	OR1L6	MYRIP	UPK3BL2	SPAAR	SH3RF3	SHANK3	SLC22A10	FCN3	DIPK2B	TMTC1	LCN10	APOL5	INSYN2A	CALCRL	ERFL	OR51I2	FBXL7	SHOC1	ZNF423	PRSS48	ST8SIA6	PTPRB	LDB2	MMRN1	ERG	LRMDA	SULT1E1	SELP	RAMP3	TNFRSF6B	PDZRN4	TEK
**lung goblet cell**	DEFB131B	CAPN13	CATSPERB	CYP2C19	DEFB4B	CPHXL	OR3A2	BPIFB1	MYOCOS	AADACL2	TTC6	FER1L6	CAPN8	ROS1	FYB2	SFTPB	INSYN2A	OR6C2	ERFL	UBE2L5	TACR1	SMIM35	CYP4Z1	TEX52	ATP13A4	PLEKHS1	SFTPA2	SCNN1G	ARHGEF38	VSIG10L2	GPRC5D	CDH26	EDDM13	SHOC1	PRSS48	B4GALNT3	WDR88	SLC9C1	OR51I2	CLDN20	NFILZ	ANKUB1	BMPR1B	TMC5	CCDC200	CHST9	SCGB1A1	SLCO1B7	NIBAN1	FAAH2
**lung macrophage**	CCL18	CCL7	SFTPC	MMP12	CD300LD	SLAMF9	CCL8	PF4	TREM2	C1QC	CXCL13	C1QA	MS4A7	FCGR3B	C3AR1	C5AR1	C1QB	ZMYND15	ADGRE1	CCL24	LILRA5	SLAMF8	FOLR2	RAB7B	NLRP3	MRC1	LRRC25	CCL13	CD163	CD14	SLX1B	MS4A4A	OCSTAMP	MCEMP1	MARCO	SCGB1A1	CLEC4D	CLEC7A	PLA2G2D	P2RY6	KCNK13	SLC11A1	CLEC12A	OLR1	FNDC7	DCSTAMP	KCNK4	CCR5	CLEC5A	CD300LB
**lung microvascular endothelial cell**	CCL14	LCN6	DIPK2B	TPO	ADM5	AVPR2	TACR1	OR6C4	OR1L3	OR2L3	DOC2B	CCDC178	SELE	LHX6	NAT8B	CCL16	ZNF366	ZNF676	CNTNAP3C	FAM124B	RBP5	CCL23	ZNF727	OR5H14	CALHM5	SELP	LRCOL1	MYRIP	MEOX1	TLL1	BMX	OR52H1	CPXM2	SYT15	TAS2R8	CAPN11	MYMX	C1QTNF9	ZNF385D	GALNT15	SMIM33	MMRN1	CETP	CCM2L	CYTL1	TMEM273	SMIM9	MEOX2	KIF19	EDDM13
**lung neuroendocrine cell**	CA6	SLC26A9	ZACN	KLK12	CALCA	TPH1	GRP	ASCL1	OR51E1	NMUR2	TMEM61	SLC35D3	SCGB3A2	GP2	GPR142	SFTPA1	SFTPB	SUCNR1	ENTPD8	SCGB1A1	GCG	KCNK16	MISP3	MS4A8	SFTPC	R3HDML	SCGN	TCERG1L	GALR2	KRTAP17-1	MYH7B	NKX2-1	GSTT2B	C2CD4B	PNMA6A	HS3ST6	CHIA	NR0B2	SHISA8	TFF3	CCN5	TRPM8	GPR21	HEPACAM2	SFTPD	PGC	DEPP1	SCGB1C1	CIB3	PRSS37
**lung secretory cell**	ADGRG7	ARSL	OPRK1	FAM240C	MIDEAS	SLC22A31	H4C9	UGT2B28	TMPRSS11E	RTL10	SPRING1	ITIH2	SCGB3A2	LAMP3	OR5M3	RTL9	SFTA2	H2BC9	BAAT	H2AC11	GASK1B	UGT2B10	SLC6A3	CHIA	ROS1	SFTPC	ATP13A4	PRSS1	NKX2-1	H4C3	H2AC4	EPRS1	PNLIPRP3	SPTSSB	C4BPA	ABITRAM	H3C10	MC4R	TCIM	HIF3A	PGAP6	EEF1AKMT3	KLLN	GGTLC1	EDA2R	PLAAT3	ERP27	SLC6A14	CAPN6	H3C6
**lusc**	DEFB103B	DEFB103A	LCE3E	SSX4B	TBC1D3K	TBC1D3E	RAB6D	FAM240C	SSX4	H3C3	SPINK9	TBC1D3G	LCE3D	THSD8	TBC1D3H	FAM90A26	SLURP2	CTAGE8	KRT6B	NTS	EEF1AKMT4	CARD18	SPRR2A	H2AC18	KRT6A	ALDH3A1	MS4A4E	TPRG1	CES1	SPRR2E	RAET1L	RAMACL	DSG3	TBC1D3	LGALS7B	CALML3	SPRR1B	KRT17	CTAGE4	TBC1D3C	TBC1D3F	OR6C75	CSTA	CYP4F11	OR6N1	AKR1C1	AKR1C2	CTAGE6	HSPA6	TP53AIP1
**luteal cell**	SOX14	SLC5A11	BEST2	GSTT4	RS1	TREH	CLC	NAT1	GK2	NXF2	ART1	HDC	INSC	SIX4	RGR	HRG	RGN	CRP	WDR62	CABP1	RAB19	SPX	SRL	SP7	CPO	OCM	SOD3	OXT	ALG10	SYP	PDP1	PSD	MED26	GRP	CSN3	DMTN	RNASEK	TTK	DYRK3	PAK3	CADPS	NXF3	AGO1	HEPH	MYC	SMG5	CIC	DPH5	SPEN	COL4A1
**lymph node stellate cell**	BPIFA2	SMR3A	CCL19	SERPINA10	NTRK1	CXCL13	LHX8	CCL11	C4B	CCL7	MEOX2	CLEC3B	MUSTN1	SHISA3	TNFSF11	GPR88	KCNJ8	MMP3	FAM180A	IL33	ZMYND15	INMT	VTN	INSRR	CPXM1	SLAMF8	DES	PABPN1L	CXCL12	WFDC12	IL7	CSF2RB	BST1	HMGCS2	STEAP4	ENPP2	RSPO1	MEI4	VCAM1	MSC	SLCO2B1	KCNE4	DPT	FNDC7	SPIC	FOXS1	SRPX	CHRDL1	SLC10A6	DCN
**lymphocyte**	KRTAP4-11	SCGB1D4	CCL21	FAM180B	SCGB1D2	PRG3	AMELX	MMP26	MS4A3	XCL1	PPP1R2C	ELANE	FAM162B	CEBPE	CTSG	QRFP	CCL14	AMBN	PRTN3	ALG1L2	CILP	FCRL1	GOLGA6B	MMP8	HRH4	PAEP	SFRP4	AVPR1A	TIFAB	MMRN1	CD177	IFNG	THBS4	MPO	GPR132	ZC3H12D	XCL2	EPX	CXCR5	TRIM72	RAB44	LRRC70	MYOC	HAS1	PGLYRP1	CD28	GPR174	KLRK1	CCR2	RSPO4
**lymphoid lineage restricted progenitor cell**	BFSP2	RAG2	VPREB1	IGLL1	IL12A	RAG1	DNTT	VPREB3	SAPCD1	RNASE12	MYL10	PAX5	ARL5C	MROH2A	NIBAN3	ADGRG3	SLAMF6	SLC35D3	FLT3	MZB1	CD19	WDFY4	DRC7	IRAG2	CD79A	SLC34A2	CD79B	CCR8	MSH5	SH2D5	TNFRSF13B	TSPAN32	TEDC1	BLNK	KLHL6	EBF1	MYB	CTSE	POU2AF1	TESPA1	CD93	IL18RAP	TIFA	FCRL6	TREML2	LAX1	GSTT2	JAKMIP1	DNAH8	ARPP21
**m398**	OR10AG1	OR13F1	OR6K6	OR1A2	OR10G2	OR10A7	OR4A47	OR5T2	TAS2R38	TAS2R41	OR51T1	OR2T27	OR1S2	OR2F2	OR11L1	OR8U1	MYBPH	OR6N2	OR9G1	OR11H6	MYH3	OR5K4	OR10P1	VN1R4	TAAR8	OR51S1	OR5J2	OR6C65	OR9Q2	OR51F2	OR4B1	OR9K2	OR1B1	KRTAP29-1	OR4C11	OR6C74	OR2T4	OR4S2	OR4C15	OR4K5	OR8K5	OR8J3	OR4C5	OR2T3	KRTAP10-5	OR7G1	OR5D16	OR6C1	OR6B2	OR10G7
**m cell of gut**	CCL23	CCL15	LYPD8	PRAC1	MS4A12	NXPE4	TMEM238L	LEFTY1	MUC12	FAM3D	PPP1R14D	NXPE1	UGT2B17	CEACAM7	CCL20	SPINK5	PI3	ADGRG7	NOX1	SPIB	CYP4F12	KCNE2	UGT2A3	PLA2G10	HOXB13	DRGX	TINAG	TRIM31	NOXO1	CLRN3	ST6GALNAC1	RXFP4	ARL14	ISX	TPSG1	SDR16C5	CTSE	ANKRD22	OLFM4	CDX1	CDH17	MUC13	TRIM15	IHH	PRAC2	SLC5A1	NEU4	CASP5	DUOXA2	MIA
**macroglial cell**	MBD3L2	GMNC	ANKRD33	MOG	TMEM88B	OPALIN	SLCO1C1	RAX2	NXNL1	MOBP	TMEM265	MAG	GRK7	SMCO3	SPTBN5	PGLYRP3	KCNV2	CNGB3	PDE6C	ARR3	GPR139	KCNE2	DGAT2L6	MRGPRX2	AIPL1	WFIKKN2	RLBP1	SLC7A10	RCVRN	ANKK1	GJC2	SLC6A5	PLGLB1	CHRNA3	GPR151	ABCC12	NTSR2	SPO11	GNAT1	ERMN	NPY2R	TULP1	PDE6H	GDF5	HEPACAM	MMP20	KRTAP27-1	IMPG1	NIM1K	PROB1
**macrophage**	PPIAL4D	CCL24	MS4A4E	ADGRE1	FCGR1A	FOLR2	CLEC10A	CD163	C1QC	TREM2	CCL7	C3AR1	C1QB	MRC1	C1QA	CD209	CCL8	MS4A7	LILRB5	MSR1	MS4A4A	FPR3	MCEMP1	VSIG4	C5AR1	F13A1	SLAMF9	CSF1R	PF4	CCL18	MS4A6A	SLC11A1	CD86	TNFSF13	MRGPRX3	CCL3	MRGPRX2	PLA2G2D	SPI1	NLRP3	TLR2	DNASE2B	SIGLEC1	SLCO2B1	SLAMF8	CD300LD	LRRC25	PLD4	GPR34	RAB7B
**macrophage dendritic cell progenitor**	HAVCR1	CCR9	CD300C	TIFAB	CTSG	MPO	UPB1	CLEC9A	COX6A2	CLEC12A	FRMD7	PRTN3	KMO	FLT3	TMEM221	PPFIA4	LY86	ATP2A1	CLEC10A	SLAMF9	RNASE6	MYH13	PLD4	SPNS3	FCRLA	GPR25	IL12A	MPEG1	LAG3	ADGRG3	RGS18	TMEM229B	ELANE	RAB44	CCR2	IRF8	LEFTY1	LY6D	NAPSA	TNFAIP8L2	GPR45	SPIB	PACSIN1	XKRX	PADI1	ARL5C	LAIR1	DNTT	F13A1	KLK1
**macula densa epithelial cell**	TSHR	NDST4	PLA2G3	PPEF1	NOS1	KCNC2	HOXC5	COL4A3	UROC1	STK32B	BSPH1	SPATA48	AFM	RANBP3L	PLCXD3	PGLYRP3	SLC12A1	WDR72	BMP3	KCNJ1	SLC5A1	ENOX1	MAGEB18	SLC9A2	SLC16A5	THSD4	PAPPA2	SPINK14	IQCM	LIPK	HOXD3	PRKD1	MYO3B	MEIKIN	TRABD2B	PDE10A	TENM2	CASR	AMY1C	SIM1	SH2D4B	ROBO2	CACNA1D	CABLES1	CCDC83	NT5C1A	KCNJ16	SLIT2	LINGO4	CWH43
**male germ cell**	FABP9	CETN1	LDHC	COX8C	SPZ1	CRISP2	PRM3	COX7B2	SPATA19	MEIG1	ROPN1	TCP11	IQCF3	UBL4B	EPYC	HMGB4	ROPN1L	HAPLN2	SPATA3	FAM229A	DNAJB8	SPINK2	AKAP4	TEX101	GHRL	RSPH1	SMCP	SLC25A31	CABS1	GTSF1	SH2D6	OAZ3	GAPDHS	SPA17	NKX6-2	SPATA33	SYCP3	WFDC3	GPX6	SPATA18	FAM229B	SMC1B	INSL3	IL4I1	PENK	FA2H	GSG1	TNP2	TNP1	CCDC180
**male germ line stem cell**	FAM25G	TSPY2	KRTAP19-8	RHOXF1	TSPY10	TRIM49	TFDP3	TSPY3	OR8S1	KRTAP20-4	VCX3A	GAGE2A	LUZP4	ANHX	MAGEB6	DCAF4L1	DCAF4L2	PASD1	PAGE2B	SOHLH1	VCX3B	ZSCAN5C	DDX53	KRTAP20-2	TSPYL6	GAGE1	POTED	ESX1	RHOXF2	NLRP4	RBMXL3	SAGE1	PAGE2	MAGEB1	LCE3A	MAGEC1	VCX	MAGEC2	ZSCAN5B	ZNF479	CTAG2	NUTM1	TRIM49C	TOPAZ1	DAZ1	SSX3	VCX2	FAM9A	CT47B1	XAGE5
**male germ line stem cell (sensu vertebrata)**	PPP4R3C	OR10H2	OR4N5	OR52E8	OR6C68	OR4K5	OR9I1	OR5F1	OR1G1	SUPT20HL2	OR5AP2	OR10R2	OR10J3	OR2M7	OR52N5	POTEH	OR2D3	MAGEB4	FRG2B	RFPL3	OR9G4	AMELY	TGIF2LY	LEUTX	OR6C76	EBLN1	TSPYL6	SOHLH1	OR6C2	MTNR1B	DCAF4L1	MAGEB6	OR4F6	ETDA	TRIM49	OR5I1	LCE2B	CALHM4	OR52N2	MAGEB1	SAGE1	TRIM77	RHOXF1	OR10AD1	OR14C36	DCAF4L2	LUZP4	TSPY4	OR4N4	TRIM64C
**malignant cell**	PPIAL4E	ESPNL	SCGB1D1	MROH9	SCGB1D2	CYP4A11	LDLRAD1	HMHB1	CFAP157	NR5A1	PLGLB2	STOML3	CCER2	CTAGE15	KIF19	CDHR4	DNAH10	TMEM190	EFCAB12	CCDC17	VWA3A	IL5RA	WDR49	ZPLD1	CLEC17A	NPIPA5	CYP21A2	LZTS3	CD164L2	LRRC46	FCRL2	GRXCR1	STAR	ZNF713	FABP6	FCER2	CTAGE4	WFDC6	FAM216B	MYO15A	CFAP100	UGT2B15	TSPAN19	SCGB3A1	PLGLB1	ZDHHC11	IGFBP1	DNAAF1	DLEC1	RABL2A
**mammary gland glandular cell**	CSN1S1	CSN2	LALBA	CSN3	BTN1A1	WFDC3	CIDEA	CEL	THRSP	FOLR1	ORM1	FABP3	SFTPD	PLET1	LPL	CCK	LCN2	LTF	NUPR1	MFGE8	PIGR	ALDOC	CD36	CLDN8	UPK2	ARG2	BHLHA15	CLU	GNMT	GGT1	ANKRD22	SLC7A4	MUC1	SELENOP	SLC9A4	G0S2	DERL3	SLC34A2	ATP4B	WFDC12	SLC22A1	FTH1	LRRC26	FXYD3	SOX10	COL9A1	EGF	GADD45G	RUFY4	COQ8A
**mammary stem cell**	KRT71	WFDC12	TEX36	SERPINB10	OCSTAMP	SERPINB11	THEM5	IL17B	R3HDML	SHISA8	SMR3A	FGF21	ADAMTS18	KRTAP17-1	CSN1S1	CSN2	OLAH	BTN1A1	PSORS1C2	COL9A1	CHRDL2	NDP	GJB4	RSPO1	CRYBA4	PLB1	SLC28A3	ASPRV1	KRT79	ST8SIA6	EGR4	CCL1	CHADL	ELF5	ADIG	PRKG2	CSN3	TNFSF11	EPGN	IL24	CEL	SBSPON	KCTD4	LSMEM1	SCGB1C1	SLC6A15	IQGAP3	MUC15	NKD2	BCL2L15
**mammotroph**	POU1F1	PRL	FNDC9	ARHGAP36	IGSF1	SIX6	LHB	LHX3	GPR6	TSHB	GHRHR	FSHB	OR6V1	CGA	NPAS4	CHRNB3	DIRAS3	TCEAL2	CHGB	TCEAL6	RIIAD1	ASIC4	KLF14	DCAF12L2	TCEAL7	CALY	PRLHR	CTXN3	GCK	GPR179	DIO2	POMC	TAS2R20	TMEM249	FRG2	RESP18	NNAT	SCG2	CLDN9	ASB4	NECAB2	OTOS	TCEAL5	DLK1	SMCO2	DRD2	TMEM59L	TLL2	CHGA	ECEL1
**marginal zone b cell**	TBXT	FOXD4L1	WNT8A	MSGN1	VENTX	TAS2R9	KRTAP12-2	MYF5	ZIC3	SPRR5	TUBA4B	NANOS2	NKX1-1	OR13C8	NPBWR2	TAS2R7	KRTAP4-7	H1-8	ETV3L	MKRN3	TAS2R3	OR6T1	OR13H1	TAS2R8	ZP4	TAFA3	OR6F1	SLC38A8	FTMT	MAP1LC3C	ZP2	MLNR	FGF20	BANF2	PNMA6E	GPR148	TAS2R16	CCN6	OR52D1	OR1L1	SEBOX	FOXI2	RHCG	FAM240B	H2AC1	UPK3A	GDF3	OR4Q3	TRPA1	KPNA7
**mast cell**	TPSAB1	TPSB2	TPSD1	MS4A2	RGS13	MAS1L	CPA3	OR1C1	GCSAML	SIGLEC8	OR1L8	CDK15	IL1RL1	RHEX	CMA1	ADCYAP1	KRTAP9-6	OR52E4	HPGDS	SIGLEC6	TMEM233	INSYN2B	SLC18A2	PZP	HNRNPCL3	SMIM38	OR1L6	OR4K13	SLC24A3	CPHXL	CLDN20	OR8A1	IL18R1	SLC22A24	ENTHD1	GRAP2	FDCSP	KIT	ZNF723	PIK3R6	RGS21	ADGRE2	CTSG	OR8D1	TMEM273	ALOX5	IL9R	IL13	NTRK1	GPR42
**mature b cell**	MS4A1	FCRL1	PAX5	CD19	CXCR5	TNFRSF13C	CD79B	FCRLA	BLK	CD79A	CD22	BANK1	POU2AF1	CD180	LY6D	FFAR1	FCMR	VPREB3	BFSP2	BTLA	TNFRSF13B	NIBAN3	ARL5C	CD72	MZB1	SPIB	EBF1	GPR18	GPR174	CCR7	SLAMF6	STAP1	GRAP	IL9R	IL12A	BLNK	CCR6	NAPSA	HAVCR1	SAPCD1	GIMAP6	SP140	TLR9	P2RY10	CD86	DOK3	IKZF3	HVCN1	PPP1R16B	CLEC20A
**mature natural killer cell**	NCR1	ADAMTS14	KLRK1	QRFP	EOMES	TBX21	KLRC3	IL2RB	CCR2	CD244	TIGIT	KLRC1	XCL1	CD160	CRTAM	CCR5	KLRC2	CMA1	PRF1	TXK	LAX1	CXCR3	IL18R1	IL12RB2	SH2D2A	IL18RAP	ADORA2A	KLRD1	GZMA	GPR171	STYK1	CTSW	LACTBL1	SAMD3	F2RL2	HSD11B1	S1PR5	TNFRSF9	GIMAP5	SH2D1A	NKG7	GIMAP8	SEMA4A	PGLYRP1	GIMAP6	GIMAP4	ITGAL	PKD1L2	PTPN22	LIM2
**mature nk t cell**	KLRC4	OR2Z1	ZNF683	GZMK	KLRC1	KLRC2	OR4D1	KIR3DL3	IL26	GZMH	FCRL6	XCL1	XCL2	KLRD1	ZNF831	GZMA	KLRF1	CD160	FGFBP2	PRF1	GZMB	KLRK1	KLRG1	NCR3	GNLY	NKG7	GZMM	CCL5	TBX21	IFNG	KIR3DL1	CTSW	KLRB1	KLRC3	GOLGA6L1	TXK	SAMD3	IL2RB	PTGDR	NCR1	KIR2DL1	CD8A	CRTAM	CST7	KIR3DL2	KIR2DL4	CXCR6	KIR2DL3	FASLG	GPR174
**mature t cell**	SFTPC	KLRB1	IFNG	SCGB1A1	CD69	IL2RA	CXCR4	ICOS	TNFRSF4	FCN3	SCGB3A1	GPR183	RGS1	XCL2	PDCD1	IL7R	CD40LG	CCR6	TIGIT	PMCH	XCL1	AREG	CD2	FOXP3	BATF	CCL18	CCR7	KIR2DL4	CD3D	SDR42E2	DUSP2	DEFA3	GPR171	CCL4	ZNF683	RGCC	TRAT1	ZNF331	SAMSN1	LAIR2	DEFA4	SOCS1	CD7	CTLA4	CD3E	RGL4	SFTPA2	CLEC2B	TNFSF8	TNFRSF18
**mb**	BARHL1	TP53TG3B	TLX3	ZNF679	TBATA	FOXD4L4	INSM2	NEUROG1	SSX4	TP53TG3	ATOH1	NPS	IGFBPL1	OR11H12	SLC6A3	GOLGA8T	ZIC4	NUDT4B	NHLH2	TAS2R40	MAB21L1	OR13D1	OR11H2	GSG1L	TRIM49D2	BARHL2	ZIC1	ZDHHC22	TP53TG3C	TAS2R39	MYOD1	ZIC5	POTEB	FOXD4L5	DRAXIN	GOLGA6L7	OR2B2	VN1R4	SSTR2	CACNG2	ODAPH	RFPL1	OR11H1	OTX2	CSAG2	OR4A5	OR5L2	INSM1	ANKRD40CL	NEUROD1
**mc38**	LUZP4	FNDC7	CXCL10	UBD	PNMA5	SLC9B1	TCP11X2	ADM2	ZBP1	CFAP58	FMR1NB	CXCL9	REC114	IP6K3	LBX1	BRINP3	EXTL1	NXF3	CNTN3	PRAMEF12	SPRY3	COL5A3	MMP9	CCL7	GDNF	RSAD2	EN1	CILP	PLA2G2E	SLAMF8	VCAM1	CCL2	EXOC3L4	CASP12	TWIST2	NOS2	RBP2	DMRTA2	DPPA2	TSGA10IP	FOXS1	DCLK1	SERPINE1	MEDAG	GJB4	INSL6	C1QTNF6	WFDC12	RNF19B	PAX3
**mcc**	CCER2	TEX13C	NHLH1	MYO15A	ATOH1	TAC3	TFAP2D	GRXCR1	PYDC1	SCGB1D1	VIP	TUBB8	SCGB2A1	TLX3	LHX3	CNPY1	BEST3	ADCYAP1	SCGB1D2	KCNH6	KRT20	KRT9	KIF19	NEUROG2	INSM1	TMEM74	AMBN	CRYBA2	SLC35D3	CT45A10	FOXE3	POU4F1	SAP25	KRT85	NPY	CHRNA3	NEUROD2	NEFM	DLX4	CCNA1	HEPACAM2	MAGEA9B	FRMD1	LGSN	GNG8	PCP4	DRGX	TFAP2B	FEV	CALCB
**mcf7**	NBPF4	ZNF593OS	H2AC7	H1-5	H2BC13	H2BC12	H1-4	H2AC20	NBPF6	H4C8	H3C12	IL20	H3C4	H2AC13	H2BC5	BRME1	ZNF888	H4C11	DEFB131B	H2BC4	MTARC1	H1-3	H2AC18	LBHD1	H1-2	CT83	NTAQ1	COA8	H2BC11	ANTKMT	OR10T2	RAMACL	EOLA2	SLC66A3	MTLN	H2AC11	NPIPB13	CYSRT1	NPIPA7	FAM174C	CEP20	PSME3IP1	H2BC15	MICOS10	H2AC8	KARS1	ATP5MJ	MTRES1	KCNJ18	NPIPA9
**mcf10a**	LCE1B	LCE1E	SPINK6	LCE1F	LCE1C	LCE3D	RNASE7	KRT81	RAET1L	FOLR3	PNLIPRP3	RAET1G	KRT6A	CRYBB2	ALOX15B	PLD5	PSCA	SPESP1	CSF3	KRTAP10-2	SYT8	ULBP2	KRTAP10-9	IL20RB	ROS1	SERPINB5	HSF2BP	KRT34	CADM3	SUGCT	KRTAP9-4	GPR87	OR2B6	KRT14	IRX4	TP53I3	TNS4	TNFRSF10D	KRT5	GJB5	LY6K	COL17A1	IL18	PAGE3	IGFL2	DKK1	ERVMER34-1	S100A2	LAMC2	HOXC13
**mdamb231**	RFPL4AL1	MAGEC1	CSNK2A3	C4BPB	KIF4B	SETSIP	ANKRD1	SLCO1B3	GLUD2	COL13A1	COX7B2	LCE5A	TM4SF18	RFPL4A	FGF5	FBXO43	SEC31B	SHISA3	STK31	MAP7D3	TMEM156	SUSD5	KIFBP	FOXA2	MCOLN2	TRIML2	CIP2A	ARHGAP40	ZNF215	TGIF2LX	VWDE	DNMBP	ADGRF5	FAM111B	MPP4	PPP1R2B	FAM9B	PPP1R1C	GPAT2	JSRP1	DKK1	EREG	SPDL1	NEXN	ADGRF1	KIF4A	KIF14	TRAM2	SHCBP1	TAF1L
**mdamb436**	MAGEA4	IL13RA2	IGFN1	MAGEA10	TLX3	KRT34	PI3	BHMT2	ZNF280A	LCE5A	HES7	HORMAD1	KRT33B	ESM1	HMX1	PRDM13	NKX2-5	DDX43	GAL	KCNG1	FAM72D	CLEC2L	DIRAS3	NOTCH2NLA	GDF5	OXTR	HRK	MAGEA11	SIRPB1	TGFBR3L	FAM72B	KHDC1L	LY6K	HOXC12	TRIM7	FGF2	IL24	FAM72C	SMKR1	RTP3	CDKN2A	SLC39A4	ZNF696	IL1B	PRSS21	SYDE1	PRAME	ESPNL	MAGEB2	ATAD3B
**mdapca2b**	KLK2	KLK4	IL1F10	DNASE2B	FOLH1	PRAC2	MMEL1	HOXB13	GLYATL1	NKX3-1	MESP1	ZG16B	SYCE3	FOXD4	ADM2	PRSS27	TMEM210	SLC45A3	CHRNA2	STEAP2	NETO1	TMPRSS2	TRPV6	OAZ3	SPDEF	HPN	CSAG1	NUTM2E	PLA2G4F	TMEM125	SPON2	SLC35G5	RLN1	LPAR3	SYNE4	KRTAP5-9	CTAGE9	UGT2B11	ZNF613	NCAM2	TRPM4	POTEJ	ALOX15	ST6GALNAC1	CCDC57	TMEM52	TTC6	SMKR1	ARHGEF26	TNFRSF10C
**medium spiny neuron**	GPR6	SERPINA9	GPR88	SYNDIG1L	GPR139	GPR52	GABRD	RASD2	MCHR1	PDYN	CHRM1	HRH3	PTPN5	TAC1	LINGO3	GPR83	SCN4B	DCLK3	RPRML	PDE1B	HPCA	DRD2	KCNS2	GABRA4	KCNA4	ST8SIA3	KCNV1	NEU2	CACNG3	KCNJ4	KCNH4	CAMKV	SCN2B	NECAB2	RGS8	SYT6	HIPK4	RGS9	ICAM5	ITPKA	LRRC10B	ASB11	CALN1	CAMK2A	ARPP21	KRT9	SSTR4	RXRG	PHYHIP	LRRTM1
**medullary thymic epithelial cell**	KRTAP19-3	KRT38	FGF21	IFNL2	ADGRD2	OR5P2	CAPN14	FAM240A	DEFB103A	AIRE	IFNL3	LCE3B	RPTN	KLK12	OR51I2	SPRR2A	MUC21	PAX1	GPR32	UBD	KRT24	OR2A4	SERPINB12	FOXN1	CCL19	SPRR3	SLURP1	KRT33B	TBATA	TMPRSS11A	CSN2	CEACAM18	PRG3	BLID	FEZF2	UNC93A	CALCB	RAET1L	A2ML1	CYSRT1	TMPRSS11D	LPO	GOLGA6L7	SLC46A2	OTOP3	ATP12A	KLK13	NUTM2G	KRT31	FAM25A
**megakaryocyte**	MPIG6B	TUBB1	TREML1	SCGB1C2	GP6	LY6G6F	GP1BA	GP9	H3C4	PF4V1	OR2W3	PPBP	H1-6	AQP10	SCYGR4	CLEC1B	PF4	TRAPPC3L	PTCRA	RIPOR3	ITGA2B	ITGB3	SELP	OR2B2	BEND2	CMTM5	OR4E1	CD226	GP5	SLFN14	MMRN1	RGS18	TNFSF4	COL24A1	F13A1	INKA1	OR2V1	PKHD1L1	FGF23	NAT8B	TRIM58	LGALS12	GRAP2	OR1M1	ALOX12	CYP4A22	H2BC15	F2RL3	SLC35G5	DYTN
**megakaryocyte progenitor cell**	DCANP1	MPL	GP5	PRG2	GP9	PF4	TREML1	GATA1	NFE2	GFI1B	MFSD2B	GP1BA	NCR2	MPO	NUTM2B	RGS18	ITGA2B	SIGLEC16	ZNF221	SIGLEC14	NOX3	HBD	GCSAML	KRTAP5-10	CIB3	ICAM4	IGSF10	SLC22A3	MED12L	RNASE3	GOLGA8K	TAL1	GAB3	MMRN1	GP1BB	KCNK17	ADGRG3	SIGLEC5	HEMGN	ANGPT1	GPR171	LILRB5	PRTN3	KLF1	ZFPM1	PLEK	NAT8B	GATA2	CYP2C8	DAPP1
**megakaryocyte-erythroid progenitor cell**	OR2G3	TREML1	GCSAML	GATA1	USP17L1	GP1BA	GP5	F2RL2	CEBPE	TUBB1	GP9	GFI1B	HBD	MPL	RHAG	SPDYE8	MS4A3	KLF1	OR2AK2	SELP	FCER1A	OR2AT4	OR2L3	HRH4	OR52H1	NFE2	AQP10	TBC1D3G	MFSD2B	CPA3	TBC1D3H	PKLR	GOLGA8S	CA1	F2RL3	PF4V1	PRSS57	NAT8B	FAM124B	ELANE	MMRN1	TAL1	AHSP	RNASE12	RGS18	TREML2	BTK	OR51I1	TFR2	KEL
**meis2 cortical gabaergic interneuron**	FRMD7	GRM2	CPNE6	SHISA8	TRIM17	HTR1F	KCNF1	SYT6	PROKR2	GABRA5	HS3ST2	CPA6	ANKRD63	SYNPR	ZAR1	TCERG1L	FOXR2	HPCAL4	SCGN	ICAM5	KCTD4	RPRML	CCKBR	KCNB2	ADCY8	MYO16	CACNG5	DLGAP2	KCNC2	ANO4	ADAMTS19	NETO1	RASGRF1	IL1RAPL1	SLC35F4	CHRM4	DGKB	GAD1	FAM131B	KCND2	GRM4	SYT10	SLC4A10	LRP1B	SEZ6	FAM163B	MAGEE2	SHANK1	ZMAT4	ATP6V1G2
**melanocyte**	DCT	TYRP1	CLEC12B	MLANA	TYR	OCSTAMP	PMEL	SLC45A2	OCA2	PLA2G2E	PAX3	GPR143	TRPM1	CDH19	VHLL	TRIM63	TSPAN10	LCE2A	DRD3	CAPN3	DCD	SLC22A2	ROPN1B	PLP1	OR1B1	GPNMB	OPN5	MCOLN3	EDNRB	SLC24A5	CA14	BIRC7	KCNJ13	MITF	TRIM48	SOX10	ALX1	TFAP2E	BCAN	ABCB5	LCE3A	CDK15	GAPDHS	LGI3	CYSLTR2	MCHR2	OR10P1	PRIMA1	MPZ	QPCT
**melanocyte stem cell**	KRTAP11-1	KRT33A	TYR	ALPI	KRT35	SLC45A2	OCA2	TYRP1	TRPM1	TSPAN10	ALPG	PMEL	GUCY2F	KRT28	PAX3	SLC24A4	KRT31	MC1R	KRT25	DSG4	KRT73	SLC6A17	TCHHL1	KRT33B	MYO7A	SLC26A7	KRTAP13-1	MEGF10	MCOLN3	GPR143	KRT72	KRT26	GPR37	TMEM268	KRTAP7-1	KRT83	SLC24A5	KRTAP19-4	BMP15	KRT75	KRT27	EDNRB	SOX10	GPNMB	NATD1	PIANP	CHSY3	ATP6V0D2	KRTAP24-1	KIT
**melanocyte stimulating hormone secreting cell**	POMC	PRL	CRISP1	PAX7	RESP18	ALOXE3	GPR6	SIX6	LHX3	PCSK2	MC3R	NECAB2	ESM1	TBX19	PPP1R17	MT3	ZFP92	SIX3	ASIC4	CRYBA2	LHB	CBARP	PABPC1L	BHLHA15	SBK3	IL22	CALY	CALB1	CHST8	SYNDIG1L	CHGA	SPHKAP	MEGF11	GLRA2	PDYN	CGA	FSHB	CELF3	SCG2	PTPRN	KASH5	SLITRK6	PNMA8B	KRT27	VSTM2L	NAP1L5	RSPH10B	TMEM59L	VAX1	NRTN
**memory b cell**	MS4A1	BLK	TLR10	CLEC17A	HLA-DOB	LCN10	FCER2	FCRL1	BANK1	FCRL2	HLA-DQA2	CXCR5	TNFRSF13C	HLA-DQB2	PAX5	CD79A	CD19	SCIMP	HLA-DQA1	CD22	FCRL4	FCRLA	TNFRSF13B	FCRL3	VPREB3	HLA-DOA	GPR25	CPNE5	POU2AF1	HLA-DRB5	FCMR	SPIB	PNOC	HLA-DQB1	ZBTB32	ZNF860	PARP15	CD180	CD79B	GCSAM	FCRL5	NCR3	CD80	GAPT	UPK3BL2	SIGLEC10	HLA-DMB	HLA-DPA1	UQCRHL	ADAM28
**memory t cell**	GPR15	CD8B	IL22	FLT3LG	CCR6	SLFN12L	CD8A	P2RY8	TNFSF8	CCR4	KCNA3	CD6	KRTAP12-3	CD3E	IL7R	LIME1	SCML4	GPR55	CD5	PTGER2	ITK	TRAT1	THEMIS	CD96	RNF125	CD69	GZMM	SHISAL2A	CD101	AIRE	TNFAIP3	RFPL2	WNT1	OR5B21	CCL5	CD3G	ZAP70	CEACAM21	LCNL1	ZNF683	CAMK4	CASS4	NPBWR2	CCR9	PTGER4	TMC8	GBP5	KLHDC7B	LTB	TMIGD2
**meningeal fibroblast**	SLC22A6	SLC6A13	ZIC4	TBX18	ALX3	COL26A1	ZIC1	ZIC5	RANBP3L	LAMC3	ZIC2	VTN	FOXD1	EDN3	SMCO3	TMEM132C	SLC30A10	RXFP3	ALX4	ATP1A2	ADAM30	OSR1	CLDN11	ARHGAP20	COL13A1	WFIKKN2	COL23A1	RBM46	CPED1	ISLR	TRIL	FMO1	ADRA2A	KCNK2	MRGPRF	GJB6	ITGA11	HIC1	SP5	OPTC	STRA6	ABCA9	NKD1	COLEC12	IL2	SLC38A3	BMP5	FBN2	LAMA1	TEX15
**mero48a**	KRT34	SPANXB1	KRTAP1-1	ZP1	KRT33B	IGFL3	KRTAP1-5	SPANXA2	TEX29	MAGEA10	MT1HL1	KRTAP2-3	KHDC1L	SUN3	CGB2	CA9	CDSN	FAM133A	TMPRSS15	CSAG1	PTTG2	FOLR3	SPINK6	CDK15	CPA4	CCN6	CCIN	LRRC69	RECK	CDA	SPANXN3	CALHM3	SPANXA1	KRTAP19-5	SPINK13	GREM1	CCL26	SPANXC	MAGEA12	AFP	ANXA8L1	GLIPR2	OR5P2	MAP3K7CL	XAGE2	NGF	KCNJ3	IL11RA	CCBE1	KRT9
**mesangial cell**	PLA2G2E	DKK2	HTR1B	VCY	ITGA8	SULT1E1	CXCL13	NPHS2	RERGL	OR2T10	VEGFD	PCDH12	IRAG1	ADAMTS5	GUCY1A1	SLC5A12	CCN4	AGTR2	KRT82	DAAM2	LBHD2	GPHB5	AGTR1	GJA5	HOXD10	NRIP2	PLCL1	PRAMEF6	SPINK8	MUSTN1	COL21A1	EMCN	CD248	S1PR3	GUCY1A2	GDF10	PIEZO2	ERICH4	PRKG1	RASL12	KCNA5	PDE3A	ALX1	SLC17A3	PDGFRB	TRARG1	RIPOR3	SLC17A1	NGF	P2RX1
**mesc**	KRTAP9-3	KRTAP7-1	KRTAP6-2	IFNA1	H3C8	INHBC	TRHR	EFCAB9	AMTN	LCN8	TMCO5A	FGF17	GJA3	COL10A1	NGB	BMP15	TBX4	CYLC1	GHRH	DRD1	MOS	ANKS4B	MAGEB3	BGLAP	KRT76	TEX45	SPINDOC	KERA	HTR3A	ZFP42	LCN12	HOXA2	HAO2	HTR5A	PGLYRP2	LMX1B	TMC1	SLC7A9	MPZ	SPTA1	EPB42	SLC15A1	CILP2	MUSK	FOXD3	ANKRD2	APOA4	SALL3	ENAM	ALOX12
**mesenchymal cell**	KRTAP1-1	EPYC	HOXD12	OR5AK2	TBX20	SULT1E1	OR4F4	SLC35G4	TRIM48	SHISA3	SULT6B1	MFAP4	MAB21L2	COLEC10	TCF21	ADH1B	LUM	COL21A1	BARX1	PCDH18	ANGPTL5	OR13C2	OTOR	GSC	KRTAP1-5	AGTR2	LRRC17	MMP23B	TRIM64C	HAND2	ITGA11	COL9A1	PRSS35	COL3A1	OGN	RTL3	DKK2	HAPLN1	POSTN	GPC3	SEPTIN4	TMSB15A	RSPO2	PDGFRA	SFRP2	DCN	MSTN	FBN2	ASPN	ALX3
**mesenchymal stem cell**	MMP27	PPIAL4D	C1QTNF5	A3GALT2	CALHM5	LY6L	HAS1	LRRN4CL	DPT	CCL7	SCARA5	CLEC3B	DUX4	CPZ	PI16	AOC3	ANGPT4	CLIC2	MYOC	MFAP5	FNDC1	HTRA3	GSTA1	C1R	IL33	MEDAG	ITGBL1	FMO3	PLA2G2A	CCL27	ENPP3	OGN	TNFAIP6	C1QTNF2	CXCL9	C1S	IL17D	CADM3	C1QTNF1	OLFML1	IL6	DCN	FAM25A	PLAC9	TNFSF18	VEGFD	PRELP	ECM2	SRPX	C1QTNF7
**mesenchymal stem cell of adipose**	MRGPRG	CCL11	CLEC3B	COL6A5	DPT	GDF10	DPEP1	SCARA5	LRRN4CL	INMT	MMP3	MMP27	HAS1	ENTPD2	PLA1A	TNXB	HTRA3	VEGFD	SULT1E1	PENK	CILP	FNDC1	PODN	PI16	CD248	MFAP5	OGN	MYOC	ADGRD1	SVEP1	OSR1	C4B	SLC10A6	RNASE10	CCL7	GABRA3	TNFAIP6	C1S	ADAMTS2	PCOLCE2	XPNPEP2	RSPO1	CCL8	ISLR	COL5A3	CXCL13	LBP	KRT36	SFRP4	GPHB5
**mesenchymal stem cell of the bone marrow**	NCR1	KLRK1	TBX21	EOMES	TNFSF18	CCR2	IL2RB	ITGAD	KLRC2	CCR5	QRFP	CD160	MS4A15	SAMD3	TXK	IL12RB2	LAX1	KLRD1	ADAMTS14	KLRC1	CRTAM	SH2D2A	PGLYRP2	IL18RAP	RASL12	GZMA	MTARC2	SH2D1A	XCL1	GPR171	CXCR3	KLHL30	CD244	CTSW	TNFRSF9	IL18R1	ATP8B4	LY9	MBOAT4	SEMA4A	TIGIT	KLRC3	RAB19	PRF1	KBTBD13	ITGB7	GPR174	CD72	GIMAP5	NKG7
**mesodermal cell**	TBXT	MSGN1	DEFA4	EVX1	CDX4	HOXB1	FGF8	LEFTY2	CFC1	CER1	MIXL1	CYP26A1	RNASE3	AZU1	WNT3A	VRTN	MOGAT2	TDGF1	TLX1	FOXF1	FGF3	SALL4	FGF4	NKX1-2	ECRG4	TRH	ZIC3	FOXH1	HOXD1	WNT8A	HAND1	FOXB1	NKX3-2	ZSCAN10	MYF5	FGF17	DEFA3	SP5	TRIM71	PRTG	FOXD4L1	CCN6	CRABP1	FOXC2	RNASE2	FIGNL2	DQX1	LIN28A	DEFA1	TAS2R16
**mesodermal progenitor cell**	NKX1-2	CDX4	EVX1	HES3	FGF17	WNT3A	FGF8	HOXB1	FOXB1	CDX1	HES7	CDX2	HOXB8	SP5	HOXA1	WNT8A	EPOP	TMEM114	TLX2	SELENOV	HOXB9	MOGAT2	HOXC4	FGF3	GBX2	WFIKKN1	HOXC8	FZD10	FGF4	CYP26A1	FGFBP3	OLIG3	LIN28A	EPHA5	HOXD1	NOTUM	TBX6	PRTG	GREB1	JPT2	RSPO3	ERAS	CCNJL	DKK4	IGDCC3	DQX1	TRH	WNT5B	SALL4	EPHA1
**mesothelial cell**	DAOA	LRRN4	UPK3B	TMEM235	RSPO1	BNC1	OR51I1	ITLN1	PRG4	ECRG4	HPR	MROH2A	MSLN	HAS1	CHST4	IL20	PKHD1L1	WT1	SLURP1	ANXA8L1	PLA2G2A	AADACL2	PTPRQ	ITLN2	PTGIS	FAM180A	OR5H14	MYRF	FAM153B	UPK1B	RAET1G	TMEM151A	FAM153A	NPY4R	C4B	CRB2	CCN4	TGM1	CFC1B	TMEM275	ADGRD1	NXPH2	SPRR2F	OSR1	PODN	CFB	MEDAG	ZNF705A	COL6A5	SERTM1
**metanephric mesenchyme stem cell**	H4C4	H2BC14	H3C3	H2AC8	HOXD12	SMIM41	H1-5	H3C6	GPR45	MC2R	NPS	H2AC15	SHISA3	H4C1	CEP20	H4C6	STEEP1	PACC1	H2AC13	INKA1	MTRES1	IL36G	PABPC4L	H2BC4	SINHCAF	INSYN2A	HOXD10	H4C2	H4C9	HROB	TEX50	SEPTIN3	ARG1	H2AZ2	DCDC2C	PRXL2B	CDIN1	RNF227	CRYM	PSME3IP1	DCAF12L2	ZFP57	H2AC11	H3C1	CFAP410	ASB4	H2BC13	CZIB	KLF14	RBIS

metanephric nephron tubule epithelial cell	MMP26	CCDC198	HOXD10	KCNG3	PAX2	PVALEF	HOXD3	NFILZ	H1-1	FBN3	TRABD2B	DCHS2	CFAP20DC	CLEC18B	EYA1	ITGA8	TBL1Y	BMPER	RBMY1J	ZNF730	CFAP91	SEC14L6	FAM240C	WT1	UNCX	NKAIN3	OR51B5	KLHL14	CDIN1	CYP27C1	CNTN5	PCDH15	KIF26B	AADACL4	HOXA6	GRIN2A	CRACD	ZNF423	FAM237A	SELENOV	ADGRG2	ZNF732	TMEM132D	CALHM4	H1-3	MARCHF10	SNTG2	HOXB3	NPHS2	C1QTNF7
mfe280	WFDC11	FLG	SELENOV	DEFB136	CLDN16	PLGLB1	PNMT	CLPSL1	BARX1	ONECUT2	LYPD1	RIPPLY1	EMX2	KCNK15	CCN6	SLC30A3	KRT23	PAX8	FOXL2	CDH6	SBSPON	JSRP1	WT1	PRDM13	SYNE4	SEMA5B	EYA4	FGD5	FOXG1	FOXC1	LCNL1	NEXMIF	NDP	PRSS21	NMU	CCNA1	GDF7	SLC34A2	TCF24	CCDC74B	RBP7	SLPI	TMEM92	TMSB15A	FGF18	MAG	ANKRD34B	HRK	GRB7	SAMD11
microcirculation associated smooth muscle cell	MFRP	C1QTNF5	GUCY1B1	GUCY1A1	TRARG1	CCL11	REM1	LAMC3	EPHX3	KCNJ8	KLHL38	CRHR2	COX4I2	TRPC3	SYN3	MUSTN1	NKX2-3	RASL12	ASPN	RGS5	NDST3	EPO	EDNRA	PDGFRB	CSPG4	AVPR1A	IRAG1	BATF2	ART3	GPR20	DMP1	ADAMDEC1	FHL5	FOXS1	SERINC4	GJA4	SLC38A11	NMUR1	HEYL	PRKCG	TACR3	VTN	GALNT16	WDR38	GGT5	PRCD	IL34	MYH11	ADRA2A	HIGD1B
microglial cell	STYXL2	P2RY12	SMIM35	CRYBB1	SIGLEC8	P2RY13	GPR34	TREM2	CX3CR1	HCG22	KCNK13	SLCO2B1	ADORA3	TNFSF18	CRYBA4	GPR84	SLC2A5	IL1A	CTAGE1	TASL	CSF1R	C1QC	TLR7	C3AR1	H4C13	TMEM119	VSIG4	ADGRE1	TMIGD3	C1QB	CCR5	HPGDS	HAMP	MERTK	GAL3ST4	WNT1	FCGR3B	NPIPB2	LY86	PLD4	ADAP2	FCGR1A	C1QA	TRIM49D1	ITGAM	LTC4S	CLEC5A	TLR10	SYNDIG1	CD86
microvascular endothelial cell	CA4	RBP5	NKX2-3	PLVAP	TM4SF18	RAMP3	VWF	FAM167B	F2RL3	EXOC3L2	CLEC14A	PASK	CLIC2	FCN3	ECSCR	SLC14A1	AVPR2	LCN6	PCDH12	CETP	APLNR	FAM110D	CYYR1	TMEM88	A2M	ADGRL4	ROBO4	KIF19	IDO1	CD36	TMEM204	TNFRSF4	NOTCH4	DLL4	ZNF366	CLDN5	ANKRD65	NOSTRIN	RNASE1	ESAM	CAVIN2	SIGLEC9	HLX	VWA1	RAMP2	EMCN	GIMAP7	MMRN2	BCL6B	SLCO2A1
middle ear epithelium	BPIFA1	PAX1	SERPINB11	LYPD2	SLC16A11	UPK1B	AQP5	LGR6	REG3G	MSLN	FOXE1	KLHDC8A	PADI1	CLDN8	ABI3BP	TREX2	GSTA3	PAX9	GABRP	CHST5	SMIM6	MUC16	LY6D	KCNJ16	FMO3	RARRES1	ASGR1	LRRC26	KRT23	ASIC5	TBX1	LTF	SFTPD	SLC15A2	ADGRG2	UPK2	PLET1	LBP	WFDC2	EDN2	ALDH3B2	CLDN22	KRT4	ILDR1	SLC10A5	PGLYRP1	TST	DMRT3	GSTM2	OSR1
migratory cranial neural crest cell	UCMA	TKTL2	RPE65	ATP1B4	OR52M1	IL17REL	NMRK2	GPR101	OPTC	FTMT	TFAP2E	TUBA3C	TAS2R3	NTSR1	CCNB3	CYP26C1	SOX8	CCDC105	STH	DSPP	TWIST1	NPBWR1	TAFA3	OR6T1	HTD2	TFAP2B	TTC16	EXOC1L	PNMA6E	SPRR5	EEF1AKMT3	GFRAL	SOX10	KRTAP4-8	PROZ	TAS2R7	GLYATL1B	RHCG	MYOG	DLX2	LRIT3	MYCN	TUBAL3	RLN3	TENT5D	KRTAP12-2	KCNH3	ASPG	CDCP2	TAS2R16
mkn45	ONECUT3	SLC6A20	PDE4C	SEMG1	BTNL8	LGALS4	FFAR4	KLK11	PRSS3	CEACAM6	CAPN8	ANXA10	AKR7A3	SGK2	TFF2	KLK10	CEACAM5	EMX1	C4BPB	TMEM139	TSPAN8	TRIM31	BTBD16	GALNT5	FOXL2	ARL14	TMPRSS4	PRR15	GPX2	HOXA13	LAMA3	IGFL2	PPP1R1B	H2BW1	HOXC11	CYP2B6	SLC16A5	GPR35	MNX1	TRIM7	VIL1	ALPP	B3GNT3	LIPH	AGMAT	F12	SFTA2	TMEM238L	MALL	MYEOV
mm	TSPY4	DAZ1	TSPY3	GPRC5D	TSPY1	TSPY10	C4A	TSPY8	DAZ4	FCRL2	TNFRSF17	OR10A4	IGLL5	SSX2B	OR10A5	CSAG3	TBC1D3F	PLAAT2	NPIPA7	TBC1D3	DAZ3	MAGEA9	FCRL5	CSAG2	HLA-DOB	TSPY2	LAMP5	SPATA31C2	MAGEB17	DEFB4A	OR2D2	TGIF2LX	UTS2R	SSX2	RGPD6	H4C15	TAS2R38	TBC1D3G	MEI1	OR52E8	OR1J1	SLAMF7	MC4R	IRF4	RGPD5	FCRL4	AMPD1	H2AC19	OR10V1	OR52N4
monocyte	OR5D14	FCN1	S100A12	CD300E	LILRA1	SIRPB2	LILRB2	INSYN2B	OR52K2	OSCAR	LIPN	TLR8	OR10S1	SIGLEC9	SIRPB1	KRTAP27-1	CD163	MS4A6A	SLC11A1	SIGLEC14	SIGLEC7	LILRB1	ADGRE2	LILRA6	FCGR2C	FCGR1A	LILRA5	FPR1	LILRA2	RNASE2	FCGR2A	CCDC200	ANKUB1	IFIT1B	FCAR	MS4A4E	MS4A4A	PRAM1	SIRPD	TLR2	CLEC7A	MNDA	AQP9	MEFV	DAZ4	TREM1	AOAH	CLEC4A	CLEC6A	FCGR3A
mononuclear cell	CD300LD	GPR141	MCEMP1	TREML4	TTR	KRT83	SLCO1C1	MMP8	CAMP	KIF5A	MT3	CX3CR1	TREM2	GNGT2	NTSR2	P2RY12	SRARP	CD300E	PGLYRP1	SPI1	S100A8	CLEC4E	DBX2	GPR34	FPR2	LST1	FCER1G	OSM	CYBB	SLC1A2	TYROBP	CRYBB1	CLEC4D	CSF1R	NFAM1	CD177	SLC15A3	SLC22A8	CCR2	LRRC25	IL1B	ADGRE1	MPEG1	PLD4	GDA	C1QC	CCL3	ALOX5AP	TMEM119	GPR84
mononuclear cytotrophoblast cell	SLC22A11	PAGE4	XAGE2	XAGE3	SIGLEC6	MAGEA8	INSL4	LGALS16	VGLL1	ISM2	ERVFRD-1	KRTAP2-2	SLC52A1	SLC13A4	CYP2J2	GJA5	OR2F1	AMELY	OLR1	CGA	CYP19A1	GRAMD2A	ERVV-1	FAM3B	LGALS13	GCM1	ATP6V1B1	ATP1A4	SLC6A4	CKMT1B	TMEM247	KRT23	SLC27A6	SPESP1	IL36RN	SLC19A3	KRTAP2-1	GSTA3	DUSP9	NAA11	MUC15	CSF3R	MLLT1	HBZ	METTL21C	HBE1	SLC13A3	MYORG	TINCR	CLIC3
mononuclear phagocyte	CD209	CALHM6	TASL	H4C13	HLA-DQB2	CLEC10A	DEFA5	SDS	MS4A6A	CD1E	CD1D	ADA2	H3-3A	CD1C	LILRB5	CEACAM4	FPR3	GPR31	CCL4L2	HLA-DQA1	VSIG4	CD207	H2BC6	TMEM273	LGALS2	H4C1	DNASE1L3	HLA-DQB1	CLEC4A	KRTAP9-6	MS4A7	SIGLEC10	SLC66A3	RNASE6	CD163	CLEC9A	H2AC16	H3C1	FCER1A	HLA-DOA	SIGLEC12	JAML	NLRP3	LYZ	HLA-DPA1	ALOX15B	CLEC7A	C1QA	HLA-DMB	ATP5MJ
msto211h	DIO2	FGF5	FGF2	HMSD	PSG4	CLDN11	NPAS1	CARMIL2	MOK	IGFN1	TRIM58	LAYN	PTX3	BDNF	HSD17B1	HOXC8	MAGEB2	CACNG8	ESM1	GAL	SLC7A11	POLR3G	ADAMTS6	DKK1	SAMD11	PRR16	RASL10B	TRABD2A	CCDC74B	FLNC	TXNRD2	CGB7	ZNF804A	FOXL2	TMEM200B	SHC3	VEPH1	SPHK1	HOXC9	NUDT6	AMIGO2	HS3ST3A1	RFLNB	AFP	CCDC74A	ZNF787	ANKRD18B	SLFN12	GFRA1	PSG11
mucosal invariant t cell	NCR3	GZMK	KLRB1	IL23R	KLRG1	CCR6	RTP5	LTK	PRF1	OR10G2	CD8A	FLT3LG	WNT1	GZMM	S1PR5	GZMA	P2RY8	TMIGD2	SLC4A10	IL7R	IFNL1	CD3E	TNFSF14	CXCR6	SLFN12L	GBP5	NKG7	COLQ	CST7	CD40LG	TNFRSF25	CD6	SLAMF1	LTA	GPR65	FASLG	OR10K1	IL18RAP	CTSW	OR10A3	OR2AP1	GNLY	TRAT1	SIRPG	KRTAP16-1	CCL5	PVRIG	OR7A17	SH2D2A	ZAP70
mucosal invariant t cell (mait)	NCR3	GZMK	CCR6	KLRB1	KRTAP16-1	KRTAP12-3	IL23R	WNT1	KLRG1	CD8A	TMIGD2	P2RY8	GZMM	PRF1	FLT3LG	COLQ	SLFN12L	CD3E	S1PR5	CTSW	LTA	GBP5	SLC4A10	GZMA	IL7R	GNLY	CD6	CST7	CD69	NKG7	GPR65	RNF125	SPOCK2	KCNA3	TNF	SHISAL2A	TNFAIP3	LTK	CCL5	SIRPG	DUSP2	IL12RB1	CD96	IL18RAP	PTGDR	CD7	CD8B	CXCR4	TENT5C	TC2N
mucus secreting cell	CTXND2	CFAP299	CLEC19A	ERFL	WFDC10B	CFAP47	OR6A2	BPIFB2	LMNTD1	WDR49	CFAP97D2	ZC2HC1B	RP1	CROCC2	ECT2L	DNAH3	SCGB2A2	DCDC1	CYP2C19	LEXM	OR1L6	MROH9	DNAI2	LRRC53	CFAP46	ROS1	DNAH7	SLC9C1	CFAP157	DGAT2L6	ODAD2	EDDM13	CFAP58	DNAH11	DNAH6	TTC29	CCDC187	KIAA2012	DNAH12	PBOV1	CATSPERE	ZBBX	ATP13A4	MAP3K19	BPIFB1	CATSPERB	TPO	KRTAP10-4	SERPINI2	RGS21
mueller cell	MINDY4B	NPVF	RLBP1	FOXI3	CLRN1	ANTXRL	CA14	RRH	IRX6	ZNF593OS	CRYAA	TAAR9	NAT8B	WIF1	CYP4A22	OPTC	PRLH	NR2E1	OPN1LW	TRPC5	ZNF648	FCRL4	RCVRN	SIX6	RAX	VSX2	KLHL33	GPR37	PDE6H	NEUROG2	AQP4	FRZB	RGR	PDC	ARR3	MAB21L1	TMEM269	SIX3	HHATL	RERGL	PDE6G	ANKRD33	PRSS35	MINAR1	MGARP	OR52N1	ABHD12B	RHO	PXDNL	OR2A25
muller cell	NPVF	RLBP1	CPHXL	CLRN1	DUXB	PRSS56	RGR	RRH	GASK1A	FAM237B	RGS21	MINDY4B	TRDN	ABHD12B	OR5P3	ZNF593OS	WIF1	SMIM38	TRH	DEFB135	RHO	ABCB11	KRTAP9-4	CRB1	MYO3A	GNAT1	HKDC1	STAC2	CLEC20A	CPA6	G6PC2	KRT40	PDE6G	PDC	GPR37	CA14	DIPK1C	SMIM36	RCVRN	NR2E1	RAX	LRIT3	SAG	CTXND2	PDE6H	PRSS35	GABRG3	PRSS48	VSX2	ZNF705G
multi-ciliated epithelial cell	CDC20B	C1QTNF8	TSPAN19	CFAP73	FAM216B	TMEM190	MCIDAS	STMND1	DNAI2	CIBAR2	SNTN	ANKRD66	ZMYND10	GAS2L2	STOML3	CCDC78	AGR3	WDR38	PIFO	ATP4B	LRRC46	DNAAF1	WFDC6	FAM166B	MORN5	DYNLT4	WDR49	CATSPERD	FOXJ1	DEUP1	ZBBX	MS4A8	NHLRC4	GJB7	SPAG8	DNAH12	CDHR4	FABP6	TEKT2	DNAI1	LDLRAD1	DYDC2	AKAP14	TEX26	CAPSL	CCNO	GRIN3B	CAPS	SPAG6	LRRC71
mural cell	HIGD1B	MFRP	ATP13A5	GPER1	C1QTNF5	GPR20	CASQ2	NRIP2	SLC38A11	NODAL	MUSTN1	RASL12	PTX4	KCNJ8	MYH11	RGS5	VTN	ASPN	PLN	GUCY1A1	COX4I2	DMP1	GJA4	ATP1A2	TRPC3	GUCY1B1	NDUFA4L2	LMOD1	FOXS1	IRAG1	SLCO1C1	KCNA5	PCP4L1	RGS4	SUSD5	P2RY14	KBTBD13	COLEC11	HEYL	MYOCD	PDGFRB	CSPG4	ART3	SLC2A4	MOBP	ABCC9	SLC22A8	REM1	ECM2	CRHR2
muscle cell	FOXD4L4	OR4A5	SULT6B1	FOXD4L5	MYOG	MYH1	MYF6	SOST	TNNC2	MLC1	FAM180B	TNNT3	CLDN17	MYBPH	MYMK	MYH2	TMOD4	CKM	MYL1	CST5	MYOD1	TRIM72	MYBPC1	CHRND	NEB	SLC45A2	ATP2A1	VGLL2	OCSTAMP	IP6K3	LCE3A	HOXD12	UBE2NL	SLN	CHRNA1	KLHL41	PRR33	CASQ2	MYBPC2	MYOZ1	SGCA	PRKAG3	DES	CORO6	CHRNG	CA3	HSPB3	AGTR2	TCAP	MYOT
muscle fibroblast	PTX4	MYOC	HMCN2	RTL3	HAS1	THBS4	TNMD	ANGPTL1	FNDC1	CCL11	SLC10A6	COL5A3	ASPN	KERA	KLF14	C1QTNF2	LRRN4CL	TNFAIP6	CPZ	PRG4	ADGRD1	COL14A1	FBLN7	FAP	ABCA9	GPHB5	ISLR	PODN	PDGFRL	CCL7	OGN	NPY4R	ITGA11	OLFML2B	SMOC2	SEC16B	MMP3	IL6	C1QTNF3	HTRA3	ARSI	ECM2	GFPT2	POSTN	BMPER	THBS2	MEDAG	ITGBL1	IGSF10	THBS3
muscle precursor cell	FAM180B	DBX1	LBX1	MYF5	PAX7	MYMK	CDH15	TRIM72	ALG1L2	CHRNA1	FAM162B	CILP	C1QTNF3	HMCN2	ASB5	R3HDML	RTL3	VGLL2	PITX3	AVPR1A	JSRP1	MYOD1	STAC3	CACNG1	HAS1	AVPR2	GALR1	ABCA8	FGFR4	THBS4	SFRP4	RAPSN	MYOG	ADAMTS4	TNNT3	ADM5	MXRA5	NTN5	FITM1	FNDC1	ADPRHL1	AGTR1	DPT	DES	CHRD	LRRN4CL	GJC2	VIT	FAM153B	OLFML2B
myelinating schwann cell	COL20A1	PLXNB3	SFRP5	RASGEF1C	CMTM5	GJC3	SLITRK1	ART3	BCHE	ASPA	KANK4	DHH	AJAP1	P2RY12	PRIMA1	COL5A3	RXRG	MATN2	MAMDC2	FIBIN	EGFL8	KCNA2	MATN4	REM1	LGI4	VSTM2A	SOX10	POSTN	ADAMTS20	DEPDC7	CNTF	SLITRK3	CLEC14A	APOD	PTH2R	TNFAIP6	GFRA3	NKD2	MLIP	SLC10A6	DBX1	PLEKHB1	ADAM11	GAS7	NKAIN4	MPZ	ATP1B2	IL1RAP	FABP7	MEGF10
myeloblast	KRTAP4-4	DCANP1	DEFA4	KRTAP4-11	NCR2	SIGLEC14	RETN	RNASE3	RNASE2	SIGLEC12	PRG2	CD1C	NBPF8	TLR10	PRAM1	AZU1	CD1D	LILRB5	TBX10	TAS2R20	FOLR3	BPI	SIGLEC5	KCNK17	GCSAML	CEMP1	ASGR2	LILRA2	SIGLEC16	FCAR	SLC22A16	VSIG4	ADGRG5	OR8J1	HRH2	AIF1	TIMD4	LYVE1	SERTM2	GOLGA8K	FOXO3B	PGA4	IFNB1	MBD3L4	GPR34	PRG3	MPO	FOLR2	ADAP2	CD163
myeloid cell	LCE2D	SMIM35	CD209	TASL	STYXL2	H3C7	MS4A4E	DUXB	CD300LD	LILRB5	CCL17	CXCR1	MCEMP1	MMP8	DEFB4A	CLEC10A	FPR3	CLEC4D	CD163	RFPL4AL1	MS4A7	VSIG4	CCL24	CD200R1L	F13A1	CBLL2	FOLR2	SIGLEC9	C1QC	SLAMF8	C3AR1	CD86	MSR1	ADGRE1	VHLL	TARM1	CLEC9A	CCL8	CCL22	HLA-DQB2	TSPY1	MS4A6A	TREM2	NLRP4	MS4A4A	CCL13	IFNL2	SIGLEC16	C1QB	PLD4
myeloid dendritic cell	CD1A	FDCSP	CD1E	CLEC10A	FCER1A	LILRB5	HLA-DQB2	CD209	HLA-DQA1	CCL4L2	CCL18	P2RY13	OSCAR	HLA-DMB	MS4A7	SLAMF8	CD1B	MS4A4A	LYZ	RFPL4AL1	CLEC7A	HLA-DPB1	HLA-DQA2	RNASE6	LILRB2	CD207	HLA-DRA	FGL2	GPR31	GPR183	CCL13	HLA-DRB1	OR2B11	ADGRE2	HLA-DPA1	CLEC5A	FCN1	JAML	VSIG4	IGSF6	CCL17	MNDA	SIGLEC7	FCGR2B	AIF1	SPDYE2B	FPR3	CLEC4F	FPR1	TLR10
myeloid leukocyte	S100A12	FCN1	GOLGA8Q	MNDA	RETN	LILRA5	CLEC7A	ERICH5	FOLR2	MS4A4A	RNASE2	HSPA6	AIF1	CD300E	MS4A6A	CD1D	GPR34	CLEC4F	TLR8	TREML4	ZNF681	GPBAR1	RNASE6	LILRA6	LILRB3	IGSF6	CLEC4A	SIGLEC14	CD300LD	LRRC25	SIGLEC7	OSCAR	VNN2	P2RY13	SIRPB1	LGALS2	CD33	PRAM1	CYBB	BCO2	CEACAM4	MS4A7	LILRA2	CLEC10A	LILRA1	FCGR1A	CLEC12A	APOC2	LST1	LILRB2
myeloid lineage restricted progenitor cell	PPIAL4D	MRGPRX2	DEFA5	CLEC4M	FCRLA	LY6L	CAMP	H4C13	CHRNE	CD19	FCER2	ELL3	CABCOCO1	PDE6H	LAX1	RNASE9	H2AC14	MMP8	TRIM34	H4C2	SAPCD1	CCR6	CLEC4F	CALHM6	POU2AF1	FCMR	GPR174	IGLL1	SPIB	CD79B	TNFRSF13C	BLK	FCRL1	CRYBA4	SPATA31C2	ARL5C	H2AC16	H2AC4	OR2AJ1	MCEMP1	S100A8	H2BC7	H2BC17	RAB5IF	ATP5F1B	OR2T8	TRAPPC2B	OR2AG1	CYBC1	BLNK
myoblast	MYMK	MYOG	PRR32	MYH8	KRT33A	MYOD1	MYBPH	MYH3	ITGB1BP2	KRT33B	CHRNA1	VGLL2	CACNG1	FGF6	STYXL2	KLHL41	TNNT3	MYF5	MYLK4	MYMX	PITX3	ASB5	KCNE5	TNNT2	CDH15	SYNPO2L	TNNI1	MSTN	KLHL40	LCE3A	MYL1	CHRNG	ZNF593OS	MYOZ2	TNNC1	MYBPC1	HSPB3	DHRS7C	CAV3	RAPSN	MYH1	PGAM2	H2BC13	TRIM55	KRTAP3-2	LMOD3	SMYD1	CASQ2	IL17B	GJD4
myoepithelial cell	CSF3	IL17B	PLET1	GJB4	C2CD4B	CRYBB3	KRT17	KRT14	LGALS7	CXCL14	NPY	CSN3	IRX1	CNN1	HTR1D	CHADL	LMO1	WNT10A	WNT11	MIA	CSN1S1	SLCO2A1	SLPI	COL9A2	KRT16	LCN2	STAC2	LYPD3	THEM5	SFN	IRX5	VCAM1	PTX3	MATN4	GJB3	CDA	IL24	IFITM10	OSGIN1	TLR2	FST	KRTAP17-1	IRX4	SERPINB5	MYH11	KRT5	LIPG	WIF1	CSF1	SMIM3
myofibroblast	FBXL22	PATE4	CNN1	IL17B	MYH11	DES	OPTC	PGM5	LMOD1	TGM4	TNNC2	HOXD13	RSPO3	EVX2	SH3BGR	JPH2	HOXD12	MYLK	CPXM2	SMOC2	TAGLN	WFDC1	HSPB6	WIF1	TMEM200C	HHIP	SYNM	NPTX2	GDPD3	SYNPO2	PCP4	SRD5A2	ANGPT4	IRAG1	ISM1	HMGCS2	KCNK3	RBFOX3	NMUR2	RASL11A	MYL9	DEFB1	FST	PDLIM3	DMPK	POPDC2	TPM2	CKM	CASQ2	CHRDL1
myofibroblast cell	TRIM49C	RERGL	REN	TMEM275	OR51E1	FAM162B	HIGD1B	ASPN	MYH11	SGCA	ECRG4	KCNMB1	ALKAL1	OR52A1	A2M	RASL12	FHL5	GREM2	KCNJ8	LMOD1	C1R	SCYGR4	CST5	HHIP	MYOCD	WFDC1	ADH1B	PLN	SPDYE11	RGS5	MRGPRF	MRGPRX3	TAGLN	KCNE4	ADGRE3	HSFX4	ADAMTS8	AOC3	COL10A1	ADAMDEC1	NPIPA2	FOXF1	GJA4	MXRA5	CST2	SYNPO2	COLEC11	KCNK17	CALHM5	FOXS1
myxliposarc	HROB	OR1E1	EIF2S3B	WARS1	NPPB	GCG	PPP1R2B	BRME1	HARS1	ERICH5	PCSK9	KARS1	PACC1	KIFBP	PGAP4	CEP20	CFAP20DC	GARS1	FKBP1C	RAB6D	NTAQ1	RMC1	CCN1	MEAK7	MARCHF5	LBHD1	CYREN	RFPL4AL1	PSME3IP1	STEEP1	IFTAP	PLAAT3	TP53TG3D	ABITRAM	BMERB1	H2BC12	H1-0	ZUP1	RTL8C	CGAS	KIF4B	H4C3	CDIN1	EPRS1	VPS35L	SEPTIN2	MAGEA4	DDIAS	COA8	TP53TG3
naive b cell	FCER2	CLEC17A	FCRL1	HLA-DOB	LCN10	TCL1A	MS4A1	PAX5	CXCR5	FCRL3	CD19	HLA-DQA2	CD22	FCRL2	BLK	CD79A	HLA-DQB2	BANK1	FCRLA	TLR10	TNFRSF13C	HLA-DOA	FCRL5	IGLL5	BTLA	HLA-DQA1	VPREB3	FCMR	COL19A1	HLA-DRB5	OR2AP1	UPK3BL2	CCR7	CD79B	HLA-DQB1	GAPT	HVCN1	PNOC	PARP15	CD72	KRTAP4-11	HLA-DMB	CD180	FAM177B	NCF1	OR10H1	SPIB	HLA-DPA1	HLA-DPB1	NIBAN3
naive cd4+ t cell	OR2AK2	SHISAL2A	FLT3LG	TNFSF8	KCNA3	TRAT1	CCR7	CD6	KRT73	FAM153A	TMIGD2	CD5	CD3E	ADTRP	ITK	P2RY8	TXK	SLFN12L	KRTAP12-3	LTA	IL7R	CD40LG	CD28	NPIPB4	LEF1	ICOS	PTGER4	CAMK4	CD69	TMC8	TAGAP	FCMR	TRABD2A	LTB	GIMAP5	SIRPG	MAL	LIME1	ANXA2R	SCML4	CEACAM21	CD7	GIMAP7	ANKRD55	TESPA1	CD3G	PIK3IP1	FAM153B	IPCEF1	TNFAIP3
naive cd8+ t cell	CD8B	CD8A	KIR2DL1	FLT3LG	OR2AK2	GZMM	P2RY8	KRTAP12-3	CD3E	FCRL6	GZMH	TNFSF8	SLFN12L	SHISAL2A	THEMIS	FGFBP2	KRTAP16-1	KCNA3	CTSW	KIR2DL3	CCR7	CD6	CD5	RNF125	ITK	GNLY	LIME1	CPA5	ZNF683	TTC24	CD3G	TXK	CD7	SIRPG	ZAP70	KIR3DL2	NCR3	SCML4	NELL2	CD69	IL7R	PTPRCAP	KLRG1	LTA	ANXA2R	CD3D	CD96	PLAAT4	PATL2	PTGER4
naive t cell	MS4A3	ELANE	H4C7	CEBPE	CTSG	PRTN3	SPDYE2B	TAS2R60	SPDYE2	OR4D1	SEPTIN1	MPO	PRSS57	KRTAP4-6	EIF2S3B	CCR7	CA6	TRAT1	SIRPG	PPP1R2B	RIPOR2	H2AC7	SPDYE18	GMCL2	KRT72	CD5	CLEC12A	RAB44	CLEC5A	FAM153A	CD3G	GCNT4	GFI1	CHST13	PGLYRP1	ITK	CD40LG	NBPF26	HK3	ARMH1	FCMR	IL7R	CD28	CD6	UBASH3A	OOSP1	SCML4	ICOS	TNFSF8	SIGLEC5
naive thymus-derived cd4-positive, alpha-beta t cell	DEFA3	CCR7	TRAT1	FLT3LG	SIRPG	OR10G2	ELOA2	CD40LG	GIMAP7	FAM153A	KRT73	CD5	KRT72	RGL4	CD6	CD3E	TMIGD2	TNFSF8	IL7R	ITK	GIMAP2	FCMR	CA6	OR10J5	TRABD2A	CD3D	DEFA4	LEF1	TESPA1	CD3G	ADTRP	LTB	PVRIG	CHI3L2	TXK	GCSAM	OR7A17	SCML4	CD7	LTA	IL36A	ANXA2R	TRAF3IP3	SELL	UBASH3A	OR10H1	OR4D1	THEMIS	ZAP70	TMC8
naive thymus-derived cd8-positive, alpha-beta t cell	CD8B	CPA5	CD8A	CCR7	CA6	FLT3LG	KRTAP12-3	KLRC4	CD3E	CRTAM	SIRPG	THEMIS	GIMAP7	RGL4	OR10H1	GZMM	CD6	OR2AP1	OR7A17	CD7	CD5	FCMR	ITK	CD3D	LTA	TMIGD2	OR5D18	GCSAM	OR1A1	IL7R	PASK	CD3G	GIMAP2	P2RY8	OR10K1	ZAP70	OR2L2	TESPA1	LEF1	TRABD2A	SCML4	TXK	GRAP2	OR4F17	TNFSF8	TTC24	ANXA2R	CEACAM21	UBASH3A	TCAF2
nasal mucosa goblet cell	SCGB1C1	BPIFB4	MRGPRG	UGT2A1	FETUB	REG3G	GLYATL3	PRSS33	CYP1A2	BPIFB3	ATP13A5	SRD5A2	BPIFA1	PON1	SERPINB11	OBP2A	FER1L6	OBP2B	SLC5A9	GPX6	SEC14L3	MUC5B	SFTPD	ADH7	AQP4	NR2E1	GABRP	TEX33	KCNJ5	SULT1C2	BPIFB6	VMO1	KLHDC7A	LYPD2	RTP2	SLC10A5	SLC22A2	CNGA2	CNGA4	DEFB103A	LDHD	KCNJ15	DMRT3	GSTA3	TFF2	TACR1	GP2	FGF20	RTP1	CYP1A1
nasopharyngeal epithelial cell	CYP2F1	BPIFA2	CYP2A6	CYP2A13	CYP2A7	MUC21	WFDC5	FOXE3	SERPINB4	PADI1	FUT3	RAET1G	LGALS7B	PGLYRP4	ADH7	MSMB	SEC31B	ADGRF1	FUT2	TCN1	TMPRSS11D	ATP12A	LCE1C	SERPINB13	LYPD2	DHRS9	MUC20	VTCN1	MUC16	ADH1C	ALOX15B	FUT6	AMTN	HSD17B3	GJB5	SLC13A2	OTX1	CLCA4	IL20RB	SLC4A11	SCGB2A1	GCNT3	ALOX15	CERS3	TMPRSS4	CAPN13	FGFR3	ERN2	EDN2	MMP10
natural killer cell	KLRF1	KIR2DL1	KIR2DL3	SH2D1B	KIR3DL1	KIR3DL3	KIR2DL4	KIR3DL2	FGFBP2	KIR2DS4	KLRC3	GNLY	XCL2	FCRL6	KLRC1	PRF1	GZMH	KLRC4	CXCR1	FASLG	FCGR3A	GZMB	PTGDR	KLRD1	S1PR5	TBX21	NCR3	OR4E1	SLFN12L	NMUR1	KLRB1	LAIR2	NCR1	CD160	GZMA	KLRF2	CD247	SIGLEC7	LIM2	TXK	GZMM	SAMD3	KLRC2	NKG7	IFNG	IL2RB	SMIM38	CTSW	FCRL3	XCL1
natural killer cell cell	KIR2DS4	GNLY	GZMB	THSD8	HSFX3	XCL2	OR11G2	FSCN3	KIR2DL4	KIR2DL1	KIR3DL1	KLRC3	GZMA	KLRD1	NKG7	CCL4	GZMH	KIR3DL2	UBXN11	CCL5	ATG10	KLRC1	BEST1	DDTL	SPATA21	KLRC2	FBXL18	CCL3	ATP5MGL	SNX22	KLRK1	CCL4L2	PRF1	AREG	IFNG	MCMDC2	SPDYE1	TRABD2A	SULT1A4	CD8B2	WDR38	GRIN3B	NR1I3	NPIPB8	TNFRSF6B	GPR174	DLEC1	APOBEC3H	NPIPB9	SPDYE16
ncih358	OR56A3	SSX5	PYDC2	MAGEA2B	H2BC13	SPANXA2	OR51B4	OR51I1	SPANXA1	OR12D3	H2BC12	MAGEA8	OR56A1	H4C11	XAGE1B	C1QTNF9B	H1-5	OR8B12	PAGE5	OR51B5	H2BC4	OR52D1	CT83	DHRS2	H1-1	H2BC11	OR51B6	OR8G5	NTAQ1	H2AC18	CSF2	NPIPA7	MIDEAS	CGB5	SULT1A4	MAEL	H2AC13	FOXR2	FMR1NB	XAGE1A	EIF2S3B	ABITRAM	RNF212	COX7B2	POTEH	H2BC5	TAS2R7	CEP20	BRME1	POTEF
ncih446	PAGE1	MAGEA10	PYDC2	MAGEA1	FAM133A	C1QL1	STAC2	LY6H	GFAP	MAGEA12	ELN	ATOH8	CSAG1	ALX3	FABP6	MAP3K7CL	NPR3	HGF	C1QL4	MAGEA6	CPA4	CYP26A1	HRCT1	TLX3	FOXS1	MAGEA3	DMRTA1	COCH	CDKN2A	MAGEA11	CA9	NKX2-8	HMX2	HES7	FOXG1	CDH7	LHX9	MGP	OR51B4	LRRN2	PRSS12	LGALS9B	PRDM13	MAGEC2	TMSB15B	FSD1	LGR5	BCHE	CCDC102B	TP73
ncih460	GLP2R	NR0B1	MAGEA1	ALDH3A1	HES7	TRIM16L	FHAD1	CD70	CPS1	TRIML2	MAGEB2	MT1A	LCN1	GAL	BEGAIN	EGFLAM	WNT10B	GREM2	MPZ	AKR1B10	RSPO3	TRIM7	INSL4	CYP1B1	AKR1C2	ABCC2	DAW1	CYP4F11	AKR1C1	EXO1	SLC7A11	G6PD	AKR1C3	SLC27A5	ZNF469	GKN1	TRIM16	OPN3	TGFBR3L	GDA	NQO2	ANKRD2	EPGN	P2RX5	KCNG1	CXCL1	ATAD3B	EIF1AY	ANKS6	AGR2
ncih522	RFPL4B	CDH7	FAM9C	SOSTDC1	ZNF860	ZNF280A	ADM5	COL11A1	NPW	FGF2	DLX2	SFRP4	GBX2	FOXL2	RNF212	USP44	CCDC74B	ZNF492	FOXE3	SOX2	MAP6D1	GPR27	KLF14	GJA3	HOXC8	ZFP69B	TMEM30B	ALDH1A1	BARX1	ARL9	SEMA3D	SELENOV	HSD17B1	HOXC6	SIX1	CDKN2A	PDGFC	OTOGL	ID4	ZXDA	GLI1	ANKRD18B	CHD5	ZNF257	SAMD11	TLX1	NR2F1	RIPPLY3	HOXC9	HOXC10
ncih650	MT1HL1	ARL14EPL	CACNA2D4	BEST3	PAGE5	MAGEB2	IL31RA	VSTM1	SOHLH2	KRT79	HMSD	MAGEA4	PRCD	ZNF716	CATSPER1	MAGEA10	SPANXC	CCR10	TRIM7	APOBEC3B	STEAP1B	OCA2	MAGEA6	NAGS	CFAP251	PTPRN2	PLEK2	NKX2-8	CACNG8	PRSS21	MARCHF4	CPNE7	SUN3	KLHL35	MAGEA3	MYEOV	TLCD5	KIAA1755	NPW	SH2D2A	LY6K	VEPH1	BATF3	LAT2	APCDD1L	CCDC71L	RIN1	IL13RA2	PRAME	GFPT2
ncih838	GAGE12G	GAGE12D	CT47A8	CT47A4	GAGE12B	GAGE2E	CT47A3	GAGE12C	CT47A2	CT47A11	GAGE12E	CT47A1	CT47A5	CTAG1B	GAGE12J	CSAG2	CT45A3	CT47A9	CT47A10	GAGE10	CT47A12	CT45A5	GAGE13	GAGE12F	GAGE12H	CT47A6	CT45A9	XAGE5	CT47A7	CGB1	MAGEA2	GAGE2A	GAGE1	CTAG2	DEFB103B	TP53TG3	MAGEA9B	H2AC4	H2BC17	H4C12	CT45A10	CGB8	XAGE1B	OR56A3	H2AB3	H2AC18	H2BC13	OR52N5	DEFB4B	RAMACL
ncih1048	HMX1	OR2T8	POU2F3	KISS1R	SYCP1	C1QL4	TPTE2	HELT	TRIM58	NPW	HMX2	TMEM270	CDH24	PAX7	PLA2G4D	NXPH4	STC1	FOXI1	TFAP2B	BATF3	KRTAP3-1	DLX5	TMSB15A	HOXC12	SUCNR1	HOXC11	TMEM238L	UTS2	BHLHE22	CCM2L	C1QL1	NMU	PRAC2	KIF1A	ETV7	CRYBB2	NKX2-5	LCE1B	NUTM2E	NPB	SYT10	GDF6	PALM3	GNAT3	TMSB15B	FAM111B	RBM11	ECEL1	HOXD13	CBS
ncih1435	SFTA2	KLK6	KLK5	IGFL4	TMEM249	TMPRSS11E	MAGEB2	NT5DC4	TRIM15	MAGEA12	MAGEA3	MAGEA4	ABCC2	SULT2B1	ZBED2	NOTUM	CYP4F3	CSAG1	IL20RA	SLC52A3	ASCL2	SLCO1B3	EREG	TNFSF15	OR4C6	CYP4F11	POTEI	GNG4	CTAGE9	OR51B4	ONECUT3	MAGEA6	ARHGEF35	KRTAP3-1	LMTK3	CDA	RNF223	KCTD21	KRT23	RNF43	HOXB8	NR0B1	RGS20	CARD14	LHX5	EPS8L3	HSD17B1	GJB3	ALG8	KLRC2
ncih1568	PSG8	PSG4	MAGEA4	SLC1A6	KRT80	VGLL3	SLC44A3	CGA	FAM25A	FAM83A	IVL	NPTX1	UPK1B	MAGEA6	CST6	S100A3	NKX2-8	LCE3E	MARCHF4	EFNA1	MT1F	ADAMTSL4	RHOV	GJB3	CSAG1	PRDM13	WNT9A	MAGEA3	RNF212	GRAMD2A	GPR27	OLR1	SPNS2	CITED4	ZNF185	MPZL2	H2BW1	S100A9	IGFBP3	ERP27	STC1	MAGEA12	TMEM40	GPRC5A	EFNA3	RNF224	GIPC3	NKAIN4	ITGA2	SFRP1
ncih1792	CARD14	PYDC2	CCL26	ONECUT2	SHC3	PI3	HOXC9	SPANXA2	CCDC68	GAL	IL11	PRSS21	WNT7B	RNF212	NPW	GPR143	RAET1E	ERVMER34-1	RAET1G	CST2	ZBED2	COCH	PLEKHG4B	TNFRSF10C	PDZK1IP1	IFNE	CGB2	MUC12	MAGEA10	CDKN2A	CYP24A1	GJB7	HOXC8	SMC1B	ASCL5	SMKR1	ZNF469	TNFRSF8	RIBC2	SLC27A5	MESP1	FSTL3	NMB	C2CD4D	PLEK2	PCSK9	ULBP2	POPDC3	ARL9	ARTN
ncih1944	LCE3E	LCE3D	HPD	VCX3B	VCX	CYP24A1	FAM83A	RSPO3	MT1A	TNIP3	CXCL5	FAM133A	AKR1C2	CRB1	IGFBP1	UGT2B4	CSAG1	CPS1	GLP2R	ABCC2	CARD14	MAGEA12	AKR1C1	TRIM16L	FGF2	SLC51B	KRTAP19-5	SLCO1B1	SLCO1B3	AKR1B10	CA12	CFHR1	PAQR5	RAET1E	TDO2	CA6	KRT80	AKR1C3	EVPLL	GYS2	CFHR3	KCNK15	PI3	CES1	SPP1	MYEOV	GCKR	IGFN1	WNT7B	WDR72
ncih1975	CT45A9	XAGE1A	XAGE1B	H4C12	CT45A3	CT45A5	POTEI	H2AC14	H1-1	H2BC9	SETSIP	HNRNPCL4	PPIAL4H	ANP32D	H2AB3	DHRS2	PPIAL4E	EIF2S3B	CGB1	H2BC5	H3C7	H2BC11	ZNF676	VARS1	RUSF1	PPIAL4A	SMIM43	GARS1	NIBAN2	RAB6D	RAMACL	H2AZ1	CT45A1	H2AZ2	CEP20	FAM174C	PABIR1	H2AC16	H2AC20	CCN1	H4C1	KARS1	UBE2L5	RBIS	EPRS1	PSME3IP1	H2BC4	CFAP20DC	GMCL2	H4C3
ncih2087	XAGE1A	MYBPH	UBE2L5	CST2	KRT75	SLC35G4	DCD	PPP1R2B	TM4SF19	CST4	CYP27B1	XAGE1B	CYSRT1	PPIAL4C	CHI3L1	ANP32D	NKX2-1	SUMO4	CST1	TRIM55	MINAR2	CEACAM6	FAM187A	ADGRD2	CSF2	PAEP	NACA2	POTEE	SLC22A31	FAM83A	PLAU	OR9Q1	MT1A	PPIAL4E	SEMA4B	SPRR2D	HCAR1	SFTPA2	BOLA2B	MFSD2B	CALB2	MT1HL1	AVIL	ZNF185	GALNT6	ABCG1	KLHDC7A	FOSL1	EEF1A2	NECTIN1
ncih2110	KRTAP4-1	KHDC1L	TFF1	C2CD4D	CD163L1	MUC5AC	HORMAD1	AKR1C2	RBP7	TGFBR3L	LY6K	RSPO3	RHOV	EVPLL	CFAP251	FAM83A	ERVMER34-1	SOX15	NMB	CEACAM6	GAL	SH2D3A	AKR1B15	CCNP	MUC4	ELOVL3	TMEM61	S100A14	CPS1	TRIM16L	FIBCD1	ZNF530	CAGE1	AGR3	EPHX3	KRTCAP3	GSDMC	ARTN	KRT6A	AKR1B10	HHIPL2	MROH6	RNF223	EPGN	KCNF1	PPP1R1C	NKD2	CMBL	KRT86	MYEOV
ncih2126	IGFL1	AKR1B15	LCE3E	NCCRP1	TFF1	PSCA	KLK5	SLC1A6	EPGN	INSL4	S100A9	LGSN	LCE3D	SULT2B1	RHOV	S100A7	FAM83A	S100P	PPBP	CREG2	S100A8	PTGES	TMEM139	PRSS22	MAGEA1	RNF223	CSAG1	CRCT1	IRX4	MAGEA6	LYPD3	KRT78	MAGEA12	GSDMC	MAGEA3	SPANXC	IVL	LCN2	CES1	LY6K	CRISP3	FGG	GCNT3	OPN3	TNS4	P2RY6	SCEL	WNT7B	NPW	SPANXD
ncih2170	CLEC2L	SPANXD	KRT4	PNMT	AKR1C2	PRDM13	RAPGEFL1	PAX9	FAM72C	CDC6	HES7	SLC27A5	ERBB2	SPANXC	TRIM16L	MAGEA10	AKR1C3	CD163L1	NR0B1	PGAP3	PRSS21	POTEG	TFF1	ALDH3A1	G6PD	AKR1C1	CEACAM6	AGR2	GAL	SMKR1	EPN3	RAC3	MIB2	NMB	SPANXN5	TCAP	NKX2-8	GPX2	KCNS3	CAPN8	ST6GALNAC1	S100P	SAPCD2	SPTSSB	SPINK5	KRTAP4-1	RNF224	LY6K	KRTCAP3	ZNF695
ncih2228	SMIM43	XAGE1A	XAGE1B	POTEI	PPIAL4H	DEFB4B	SPRR2F	SETSIP	CXCL6	KRTAP2-4	KRTAP4-1	CEP20	H2AB3	PABIR1	HNRNPCL4	CCN3	NIBAN2	H1-1	PPIAL4E	H2BC5	H2AC14	VARS1	ALDH3A1	RAB6D	PPIAL4C	H4C3	RUSF1	F8A3	ANTKMT	ANP32D	H2AC20	CCN1	MTRES1	HNRNPCL1	PSME3IP1	H2AZ1	H2AZ2	PABPC3	H4C12	CXCL5	GARS1	H2BC11	HHIPL2	MTLN	KARS1	IFTAP	PPIAL4A	EPRS1	PLAAT3	RBIS
ncih2347	IL37	SFTA2	KRT33B	OR4C6	NT5DC4	KRTAP2-3	CST6	IGFL2	CFAP251	SUN3	HMSD	PDE4C	FAM83A	TM4SF18	RAB42	PSORS1C1	PLAU	VARS2	MELTF	MROH6	TMSB4Y	FGF5	KLHL35	G0S2	ADAM18	MAL2	KRTAP1-1	JSRP1	GALNT5	LMTK3	LY6K	APOBEC3B	NKX2-1	SPOCD1	ITPKA	TMEM171	NAGS	CLDN6	VGLL1	FAM25A	GOLT1A	F12	EREG	CDA	CRB3	MICB	TRIM61	GPR87	KRTAP3-1	RHOD
ncih2444	ALPP	KRT3	IGFL1	CEACAM19	LAMA3	NT5DC4	KRT81	NOX5	ADAMTS12	SAA2	CD163L1	ZBED2	INHBA	SAA1	SPANXC	ZNF114	CALB2	ALDH1A3	SPANXD	IGFN1	CDA	LAMC2	CALB1	LCN1	TGFA	SAA4	FOXD1	UPK2	KRT34	CLEC2L	ARL14	KHDC1L	KLK6	TESMIN	BLID	OR51B4	LAMB3	FAM83A	WNT7A	ZNF699	DMKN	GJB3	ERBB2	KRT86	ARHGAP40	KLK10	PMEPA1	IL11	ANKRD1	PAEP
ncih2452	MYLK2	NPPB	APCDD1L	ARSI	BDKRB1	CPA4	SLC12A8	MELTF	TMEM200B	BAIAP2L2	LOX	ZNF469	C1QL1	PSG5	FBXO2	S100A3	PSG1	ALPK2	SSC5D	OR2T8	CD70	TNFSF15	MSC	MRGPRF	ADAMTS12	PSG4	NNMT	FOXS1	CCL2	LAYN	TRIM16L	RFLNA	GALNT9	NGF	SERPINE1	LRRC61	LRRC46	KCNG1	FSTL3	ADAM12	AKR1B1	ADAMTS6	ZNF467	TRIM58	KRT33B	SHISAL1	RARRES2	RRAD	MARCHF3	CCDC80
nephron progenitor	HOXD12	UNCX	FGF20	SHISA3	HOXD11	HOXD10	SIX2	REM1	PAX2	HOXA11	WT1	GDNF	HOXC10	TRIL	CLEC18A	NTSR1	HOXA10	CAPN6	CRYM	OSR1	HS3ST6	EYA1	HOXA9	FOXD2	ALDH1A2	COL26A1	TCF21	S100G	MEOX2	MEOX1	HOXC9	HOXC6	GPC3	ITGA8	LHX1	HOXD9	RXFP3	H2AC12	CCDC8	REN	HOXA6	SALL1	ZBTB12	HOXB9	CLRN1	CPED1	AGTR2	SNCAIP	HOXD4	HOXB8
nephron progenitor cell	CLEC18B	RIPPLY1	NOX3	MMP23B	PAX2	CLEC18C	SHISA3	SLC15A1	ECEL1	RASSF10	WT1	LYPD1	FOXC2	TMEM100	CDH7	ATOH7	NKAIN3	HOXD1	TMSB15A	SIX2	CLEC4M	DAPL1	CLEC18A	HOXA11	HOXA6	TCEAL7	HOXA10	COL9A1	HTR2B	HOXD11	ZNF385C	LRRTM1	HOXA9	UNCX	CST5	LHX1	KLK6	CALCA	COL13A1	PNMA6A	SOST	ZNF503	ZSCAN1	MEOX1	NLRP1	NR2F1	ITGA8	PAX8	AQP12B	TMSB4Y
net	MBD3L2B	MBD3L5	MBD3L3	SMIM32	KRT40	MBD3L2	TPH1	ADGRG4	KRTAP5-5	PRODH2	KRTAP3-3	OR51E1	TAC1	SLC18A1	CYP3A7	CYP3A4	KRT39	MS4A8	GLRA3	KCNH6	PCSK1	MNX1	CBLN4	RXFP4	CYP4F3	PRLHR	CRYBA2	SYT13	TGM3	GHSR	FTCD	CHGA	ASGR1	FEV	DRD5	SLC38A11	PHGR1	GUCY2C	GIPR	TM4SF5	MYOM3	CDHR3	CHGB	EPS8L3	CDHR5	ERICH5	RIMBP2	TCEAL2	LMX1A	SCGN
neural cell	KRTAP10-4	TSPEAR	DIPK2B	PKHD1L1	GNAT3	CCL21	GRAPL	CATSPERB	DUXB	MMRN1	AWAT1	NMS	SNTG2	CYP8B1	SHOC1	FLT4	ZFPM2	STK32B	PALM2AKAP2	INSYN2A	TLL1	PGM5	TRMT9B	ZNF521	SLC5A4	DLGAP2	PIEZO2	FBXL7	PRSS48	RELN	STAB2	UPK3BL2	KLHL4	SEMA3A	ITGA9	IL7	TBX1	SHANK3	ST6GALNAC3	PDE7B	INKA2	LRCOL1	CNTNAP3B	BMERB1	SEMA3D	CCDC102B	LDB2	LRMDA	SLC9C1	OR1L8
neural crest cell	FOXD3	WNT1	PHOX2B	UBE2NL	SULT6B1	PNMA6E	SOX10	CLDN24	HOXD12	CYP26C1	UCMA	CDH19	CRABP1	EPOP	GBX1	PSMA8	BCL2L10	MOGAT2	AGTR2	DLX2	H1-1	SOX8	TFAP2B	ZNF536	TECRL	KRTAP12-2	H1-0	TFAP2E	NKX1-1	MYCN	COL9A1	PLP1	BSX	MACIR	ASCL1	INSC	HAND2	RAB41	RCOR2	FIGNL2	NPBWR2	SLITRK6	PGAP4	MAB21L1	TKTL2	TWIST1	OR52D1	RPE65	MSX2	OC90
neural progenitor cell	H2AC17	H3C12	H4C7	TSPY1	H2AC14	PRAMEF25	NEUROG2	SLC35G4	TEX13D	PRY	H2AC7	H1-6	MAGED4B	H2AC4	HNRNPCL3	H3C11	CLEC6A	CTXND2	MAGED4	DEFB107B	FAM181A	H4C5	OLIG3	H1-5	PRAMEF5	NEUROG1	H4C1	DEFB107A	NEUROD6	H2AC16	H2AC13	DBX1	RFX4	NR2E1	FABP7	POU3F2	SULT6B1	H2AC15	UPK3BL2	ASCL1	PRAMEF26	DRAXIN	HES5	NHLH2	GFAP	FEZF2	H4C13	GSX1	FEZF1	TBX22
neural tube	HES3	HOXA1	FOXB1	GRIFIN	WNT8A	NKX1-2	EPOP	HOXB1	ERAS	OLIG3	SLC7A3	ARID3C	SFRP5	LPAR4	CRABP1	GABRG1	ZIC3	SOX1	CDX1	JPT2	MOGAT2	ZBTB12	PRTG	LIN28A	FGF3	SPSB4	SHISA8	SALL4	FOXD4	CRABP2	DACT2	DNMT3B	GBX2	HOXA2	SELENOH	FGFBP3	NKX6-1	FIGNL2	MYCN	RIOX1	IGDCC3	HPF1	GON7	CNTFR	SPOUT1	APELA	PCSK9	FAM181A	DQX1	RCOR2
neurecto-epithelial cell	VWA7	PRSS1	SERPINA4	CYP4F12	ZNF774	CTRC	CELA3A	CEL	CPA1	REG1A	SCTR	CTRB1	DUOX2	RASAL1	INS	CPA2	LRRC69	PTK6	C4BPB	EPS8L3	REG1B	SLC2A9	CAPN8	GPC2	REG3A	ZNF614	SPINK4	HGD	REG3G	BBS10	ZNF700	CDH17	PYROXD2	UGT2A3	SPINK1	PDX1	CUZD1	MYO15B	VSIG2	OLFM4	IL17RE	FGB	SAA4	CFTR	UGT2B15	TNFRSF25	SERPINA5	OR12D2	HABP2	BTN3A1
neurectodermal cell	ERAS	FOXD4L1	SOX3	ZIC3	LHX5	FOXB1	HES3	OLIG3	CYP26C1	HESX1	GBX1	TAL2	FOXD4	RFX4	WNT1	DBX1	FTMT	ZP4	GLRA1	TAS2R3	IRS4	ADGRV1	PAX3	TKTL2	FEZF1	OTX2	SEMA5B	ETV3L	RAX	DCC	WNT8B	KPNA7	CCNB3	SLC7A3	EPOP	NPAS3	MSI1	FAT3	NANOS2	NKX1-1	DSPP	TUBA3C	TAFA3	PAX7	IGDCC3	NOTO	SPRR5	SOX1	DNMT3B	LIN28B
neuroblast (sensu nematoda and protostomia)	PROKR2	PATE3	GPR21	SP9	SLITRK3	POU3F4	CACNG5	ZFP57	ISLR2	PRR36	SP8	DCX	SEZ6	IGFBPL1	HSD3B2	SLIT1	DLX6	DLX5	DLX2	GAREM2	AMER3	VAX1	TRIM67	MAGEE2	BRINP2	NOL4	KRT73	STMN4	PIPOX	GAD2	SMCO1	NSG2	CHODL	TUBB3	DPYSL5	RGS8	CAMK1G	DMP1	SCN3B	TMEM35A	GAD1	SLC6A1	OTOP3	TEDC2	CRMP1	GPR19	SYNPR	FAM241B	WDR31	ST8SIA2
neuroblast (sensu vertebrata)	SP9	HELT	HES5	POU3F4	GSX2	DLX1	DLX2	IGFBPL1	SOX1	SP8	NTSR2	ISLR2	DCX	POU3F2	ARX	FOXG1	SCRT1	TTC9B	GSX1	POU3F3	GAD2	DLX5	ELAVL3	PROKR2	NOL4	PLPPR3	STMN4	ZFP57	SOGA3	SCRT2	NCAN	SOX11	VAX1	BRINP2	SAG	REM2	DLX6	MT3	CDK5R1	NRXN3	SLC24A5	SBK1	TUBB3	SOX21	GPR21	SLC1A2	TIAM2	NSG2	MPPED2	RTN1
neuroendocrine cell	FAM237B	SCGB1D1	KRTAP24-1	GHSR	OR51E1	GRP	SLC35D3	MMP26	POTEJ	KRTAP5-5	ASCL1	CNMD	SLC6A5	CA6	CALCA	TPH1	PKD2L1	MS4A8	SLC6A3	SLC26A9	GLYATL3	PHOX2B	ASCL5	OR52D1	KLK12	GHRL	PPP1R17	SCGN	OR5B21	DBH	RFX6	KCNH6	TMEM61	GP2	PLA2G4D	SLC38A11	ADCYAP1	DDC	RPRM	NR0B2	SST	TFF3	SCGB3A2	NKX1-1	OTP	DIRAS3	CRACDL	GFRA3	DEPP1	COL22A1
neuroepithelial stem cell	KRTAP21-1	TAS2R46	DEFB109B	CDK3	DEFB108B	TAS2R50	FOXD4L6	VN1R5	TBC1D3	USP17L2	VHLL	OR4F21	OR2H2	OR2D2	OR4F3	LCE1A	RNASE8	H4C15	TBC1D3C	OR10A4	NUTM2A	TAS2R19	VN1R2	LIMS4	DEFB131A	PRR23A	HSFX2	MAGED4B	H3C13	CLDN24	SIGLEC16	OR9A2	CDC14C	TAS2R5	H2BC18	NUTM2G	SPDYE6	ARGFX	TAS2R40	OR2D3	OR10A5	KRTAP19-8	CLCA1	TRIM48	OR7D2	OR1F1	PBOV1	H2AC21	SPDYE2	DUXA
neuromesodermal progenitor cell	HOXB1	WNT8A	SP8	NKX1-2	TBXT	VRTN	HOXA1	WNT3A	GBX2	MIXL1	TBX6	CDX1	HES7	DGKK	FGF3	EVX1	LIN28A	MOGAT2	FOXH1	PRTG	POU5F1	FOXB1	OTOP3	SALL3	TCP11X2	ARG1	OGDHL	APLN	FGF17	FGF8	CDX2	TRH	AMT	SLC7A3	GSDME	PFAS	TAL2	DKK4	PSORS1C2	IGF2BP1	KBTBD8	ARL5C	SP5	AWAT2	M1AP	IGDCC3	ALDH1A2	SMIM10L2A	DLL1	NPBWR1
neuron	GABRA6	NEUROD6	PHF24	SCN2A	SEPTIN3	ANKRD63	IQCJ	MAGED4	GPR26	SLC17A6	NSG2	OR1Q1	PIRT	TTC9B	PHYHIP	LBHD2	JPH4	CAMKV	PTPN5	SYNDIG1L	SCRT1	DCX	PLPPR4	ADGRB1	CELF5	CTXN2	GRIN1	GLRA2	KCNJ4	VXN	CARMIL3	CACNA1I	FAM131B	KCNJ9	GRM4	GRIA2	TAFA1	PHOX2B	MRGPRD	OR14I1	CBARP	FXYD7	KIF5A	STMN4	GRIN2B	ADGRB2	ARPP21	FAM163B	SLC6A7	PAK5
neuron associated cell	BARX1	MYMX	FOXD3	GPR17	MPZ	EGFL8	GUCY1A1	MYOD1	GUCY1B1	PITX3	EEF1AKMT4	SLITRK2	CDH19	KLHL30	COL20A1	KCNE5	INSC	CHST5	HEYL	MYMK	TCIM	GFRA3	SLITRK6	C1QTNF12	MOSMO	KCNJ8	CDH6	DMP1	PHOX2A	PRSS12	FABP7	AJAP1	FOXS1	ATP10B	IGSF9B	MYOG	CDH10	PRIMA1	SOX10	NDST3	COX4I2	PAX7	MYF5	MOXD1	TECRL	PABPC4L	COL26A1	FAM178B	LHX8	HSPB2
neuronal stem cell	TAAR6	OR4F21	DEFB109B	OR4F4	GSX2	HELT	OR4F3	FRG2	FOXD4L6	TAS2R40	OR4F5	DEFB131A	TAS2R50	KRTAP5-6	TAS2R43	POU3F4	CDK3	VHLL	OR52W1	MAGED4B	SP9	TAS2R19	NTSR2	TAS2R39	PRR23A	LCE1A	MAGEB10	FAM186A	TBC1D3	HSFX1	VN1R5	MYMX	HES5	NUTM2A	GSX1	OR51B2	VN1R2	SIGLEC16	MAS1	OR52B6	NR2E1	LIMS4	TAS2R5	PBOV1	ASCL1	OR2D2	IFNA21	IGFBPL1	ARGFX	SOX1
neutrophil	SPATA31A7	MMP8	MCEMP1	CXCR1	CLEC4D	CD300LD	CXCR2	FPR2	ASPRV1	TREM1	CAMP	CLEC4E	IL36G	MMP9	CEBPE	OR8H1	OSM	TARM1	CD177	S100A8	OR7G2	OR6M1	CD300LB	CD300LF	PROK2	S100A12	CSF3R	CCR1	FPR1	OR4E1	OR2V2	OR5A2	PGLYRP1	LILRA6	IL1B	HCAR2	C5AR1	OR2J2	OR52K2	PILRA	IL1R2	NLRP3	CLEC5A	MEFV	SCYGR4	CEACAM8	FCGR3B	RASGRP4	OR51L1	OR10H5
neutrophil progenitor cell	ELANE	CTSG	MS4A3	MPO	PRTN3	TIFAB	GPR141	SERPINB10	HK3	F13A1	RAB44	TNFRSF13B	CD300LB	FUT7	ANKLE1	CLEC12A	FCGR3B	NFE2	ADGRG3	PRG3	CLEC5A	MMP8	ATP8B4	CEP85	MCEMP1	TFEC	NFAM1	CCR2	CARD9	IL12A	ARHGEF39	ITPRIPL1	NRROS	IL31RA	PRSS57	PYHIN1	MILR1	CCDC163	NAPSA	HSD3B2	ADSS1	IGSF6	LY86	CFP	MEFV	DOK3	HAAO	RSC1A1	TNFSF13	PIK3R6
non-classical monocyte	LILRA1	LIPN	CASP5	CD300E	LILRB2	FCN1	FCGR3A	LILRA5	GPBAR1	SIGLEC10	ADGRE3	ADGRE2	LRRC25	TNFRSF8	TLR8	LILRA2	FCGR2C	TREML4	PEAK3	SIRPB1	LILRB1	PILRA	CYP4F22	CD300C	SIRPB2	GPR42	MS4A7	LCE2D	SIGLEC9	APOBEC3A	CD300LD	SECTM1	PRAM1	SIGLEC16	MS4A14	FGR	FPR1	SCIMP	FPR2	CEACAM4	SLC11A1	CEACAM3	OSCAR	SIGLEC14	OR5H14	MS4A4A	OR52K2	S100Z	C5AR1	ITGAX
notochordal cell	FERD3L	NOTO	EMILIN3	SRARP	SLC38A8	IQCJ	CHRD	RFX4	FOXB1	HOXB1	OR52D1	CFC1	AVPR1B	MLNR	SP5	FOXD4L1	IL17REL	NODAL	WNT8B	CFAP126	FOXD4	TMEM150B	CFAP77	LMX1A	NKX1-1	ADGRV1	TAS2R9	SLIT1	TAS2R8	WNT3A	CFAP206	RSPH6A	WNT1	PLA2G2E	SHH	HES3	CFAP52	COL2A1	KRTAP24-1	DCHS2	OR52M1	ERICH2	SPRR5	TEX49	KRTAP16-1	CCDC103	DNAJB13	TAS2R13	LRRC18	TBXT
npc	SLURP2	CSAG3	OR10AD1	CFAP97D2	OR1B1	C1QTNF8	LRRC15	UBD	CSAG2	CFAP157	WFDC6	MAGEB17	MMP13	MXRA5	CCDC187	DNAI2	DUOXA2	GAS2L2	MUC16	CALHM5	CFAP73	LDLRAD1	SOST	AJM1	DEFB4A	CIBAR2	LCE1C	NKX3-2	TUBA4B	CLEC3A	SPRR2A	PRR29	WDR38	KLHDC7B	DNAH3	CFAP300	STOML3	TMEM190	CCDC78	NUTM2E	ZNF469	MMP11	CCDC33	OR6A2	KRT39	IQANK1	PLEKHG7	CROCC2	TSPAN19	HCAR3
nucleate erythrocyte	GMCL2	PRAMEF14	H3C4	OR2T8	ZC3H11B	KLF1	PKLR	MPIG6B	TSPO2	GATA1	RAMACL	FAM178B	SETSIP	SPDYE17	NBPF4	ABCB4	EIF2S3B	HBD	GFI1B	RAB6D	FAM124B	H2BC13	PRSS50	EPGN	AHSP	EPX	PNMT	MFSD2B	H1-5	UBE2L5	ICAM4	RHAG	H2AC13	SULT1A4	H2BC5	RHD	H2BC4	ERMAP	GLUD2	HBG2	PPP1R2B	TASL	KEL	H2BC12	NFE2	MPO	GBGT1	GYPB	HBM	PRSS57
nucleated thrombocyte	GP5	CLEC1B	GP9	PPBP	TREML1	ALOX12	GP6	PF4	GP1BB	APOC4	F2RL2	ITGA2B	SLFN14	TDO2	GP1BA	APOA2	P2RY12	ALB	NRGN	C8A	ITGB3	AHSG	CFHR2	PLG	CLEC4G	APOH	HAMP	PZP	APOF	FABP1	FGG	CYP1A2	MAT1A	LY6G6F	TTPA	CELA2A	TMEM40	OTC	APOA1	BCO1	STX11	FGB	RGS18	IGFBP1	SLC25A47	MFSD2B	TREML4	SLC6A4	HPSE	HPX
oe21	KRT6C	CALML3	OR51I1	KRT31	KRT6A	MMP13	KRT16	KRT14	ZBED2	ANXA8L1	CFAP251	IL20RB	SOX15	TPRG1	LCE3E	SPRR1B	KRT17	PTTG2	TP63	IGFL1	OR51B4	KRT5	CCL26	SPRR2D	TP53AIP1	CRYBB2	PTHLH	MAGEA11	GPR87	S100A2	APCDD1L	COL17A1	GJB5	WNT3A	PKP1	RAET1L	LY6K	HBE1	MAGEA4	FGFBP1	MAGEA1	MROH2A	LRRC69	CSTA	KRT9	FAM83A	BDKRB1	GREM1	PLEK2	GJB6
olfactory bulb tufted cell	FEZF1	NEUROG1	DMRT3	WEE2	NHLH2	FOXO6	RTP2	INSM1	GMNC	CNGA2	LHX2	CNPY1	EBF2	PCDH8	SP8	OBP2A	RTP1	CDHR1	DMRTA2	CNGA4	NEUROD1	NHLH1	XKR7	UNCX	SHISA7	RBM24	GNG8	OTX2	TMPRSS6	SOGA3	THSD7B	OBP2B	OLIG2	STIL	PRSS41	ATP8B4	MDM1	GPC2	CCP110	EBF3	ST18	ADGRB2	SCGB1C1	MYBL1	ELAVL4	DPYSL5	TTC24	SBK1	SOWAHB	ELAVL3
olfactory epithelial cell	OMP	UGT2A1	CNGA2	SCGB1C1	CYP1A2	RTP1	RTP2	BPIFB6	SEC14L3	STOML3	CNGA4	PRSS56	ATP13A5	UMODL1	CTXN3	TEX33	BPIFB4	BPIFB3	FETUB	PON1	GLYATL3	GDPD4	ERMN	S100A5	GPX6	ELL3	IL25	GNG13	FAM217A	GADL1	NRN1L	CARTPT	SULT1C2	PRSS33	FLRT1	KCNH4	SP8	TAAR9	TAC4	KIRREL2	RD3	GLDN	KCNJ5	OBP2A	FSTL5	CDH26	EBF2	FEZF1	SLC26A7	MUC2
olfactory receptor cell	FOXB2	GFY	GSX2	RTP2	CNGA2	GRPR	RTP1	CNGA4	GABRA6	DBX1	DBH	FOXD4	EDAR	CHAT	UMODL1	CCKAR	ADRB3	ADRA1B	S100A5	CTXN3	HTR1D	KCNH4	CCKBR	CPO	FAM217A	STOML3	GADL1	KIRREL2	ESX1	AKAP3	EPHA8	RD3	HAO1	ALPG	ADRA1D	GRM1	GRIN2C	HTR1B	ALK	NRN1L	ADIPOQ	ELL3	TMPRSS6	RAB26	CHRND	AGTR2	CNGB1	CARTPT	CARNS1	CYP1A1
oligodendrocyte	OPALIN	MOG	HAPLN2	TMEM88B	MAG	ERMN	MOBP	TMEM235	NKX6-2	OLIG1	GJC2	OLIG2	LGI3	SMCO3	GPR17	PRR18	PLP1	GPR62	PLXNB3	CMTM5	VXN	GPR37	CNTN2	CNDP1	CLDN11	FA2H	GJC3	ENPP6	KCNJ10	HEPN1	TAS2R40	TPPP	HEPACAM	STMN4	TAS2R39	TMEM151A	CARNS1	TMEFF2	AATK	ASPA	PLLP	TNR	GRM3	HCN2	BCAS1	PEX5L	MAP6D1	TMEM229A	KNDC1	SOX8
oligodendrocyte precursor cell	GPR17	OLIG2	OLIG1	NEU4	TNR	BCAN	LHFPL3	KRTAP11-1	GSX1	NXPH1	CSPG5	GPR37L1	C1QL2	SCRG1	MEGF11	RLBP1	PCDH15	MATN4	CACNG4	BRINP3	PTPRZ1	PLPP4	SOX8	LUZP2	DSCAM	PLPPR5	CA10	NLGN3	OR11L1	KRTAP12-1	SHISA7	GRIN3A	PMP2	DBX2	PLXNB3	MMD2	KCTD4	GJC3	KCNIP3	PLPPR1	SUSD5	SLC35F1	LRRTM3	SLITRK3	CDH10	CSMD3	C1QL3	SSTR1	HEPACAM	FABP7
oncodg1	DEFB4A	CLDN16	FLG	PI3	FGF18	VSIG8	NMU	PAX8	SLC34A2	CCNA1	PTX3	COX7B2	CDH6	ZBED2	CA6	CXCL1	MEOX1	TRHDE	CLDN1	KLK15	BHLHE41	IQCD	EMX2	WNT10A	VSTM1	BTC	ZNF503	DUSP13	ITGB8	KLHL35	RAPGEF3	ANKRD34B	H2BW2	NKX2-8	HKDC1	DCDC2	CXCL3	TRPM2	CDKN2A	CCM2L	UPK1B	TSKU	SLPI	CNGB1	CA2	FOXL2	THAP12	KRT23	EPHX4	PRAME
oocyte	NOBOX	OOSP3	OOSP2	PADI6	NLRP5	NLRP14	PRAMEF20	RFPL4A	ASTL	FIGLA	MBD3L2	ZAR1L	PABPN1L	ZP2	ZAR1	ZP1	MOS	BTG4	OOSP1	NOTO	WEE2	STRA8	GDF9	BMP15	UMODL1	MBL2	BCL2L10	SERPINB12	SLC25A31	DMRTC2	DPPA3	TRIM61	MAGEB4	EIF4E1B	KRT84	IZUMO1R	DAZL	TAAR9	CPSF4L	PRAMEF12	GCKR	LHX8	SYCP1	TERB1	TBPL2	PKD2L2	TRAPPC3L	TAF7L	TEX101	TXNDC2
oogonia	RBMXL2	DPPA3	UTF1	FKBP6	USP26	DND1	DAZL	MAGEB16	DPPA2	DPPA4	POU5F1	ZGLP1	ASZ1	SOHLH2	MAGEB4	REC114	LRRIQ4	SLC25A31	GDF3	MORC1	DMRT1	STK31	GOT1L1	CRYGS	FOXR1	NANOG	M1AP	DDX4	TLX3	RBM46	TCL1A	ISL2	AURKC	TDRD12	ZFP42	GTSF1L	ARL14EPL	HORMAD2	LRRC34	ADAD2	PLA2G10	PRDM14	CPSF4L	PSORS1C2	RNF17	IL2	TEX14	FBXO15	NANOS3	SYCE1
oogonial cell	KRTAP10-8	H3C3	LITAFD	OR4K17	OR4P4	OR8B8	OR1M1	OR56A4	H4C14	OR1I1	OR6K3	KRTAP9-6	OR4F15	OR2J2	OR7D4	OR6C6	H3C1	OR10A3	OR2V2	OR2T33	RHOXF2B	ECRG4	FOXL2NB	RERGL	GOLGA8O	OR7G2	OR10G3	KRTAP12-3	H4C5	OR2B11	GAGE12F	OR5A1	OR51E1	OR10H5	H2AC17	SEPTIN4	OR52A1	ERVV-2	NPIPA9	OR6Y1	OR4D9	OR4D1	HEPACAM	MIXL1	OR2A5	OR6J1	OR1D2	OR2G6	OR51M1	LIMS3
optic vesicle	DBX1	RAX	FEZF1	WNT8B	SIX6	LHX5	FAM181A	LHX2	FEZF2	IRS4	FIGNL2	SLC38A8	SIX3	OTX2	ZBTB12	CCDC177	LIN28A	WFIKKN1	LIX1	RPRM	OTX1	VAX2	POU3F4	NECAB2	SPSB4	MAB21L2	ERAS	COL9A1	DMRTA2	SOX1	CRB2	SHISA2	PAX6	SOX3	FOXB1	PRTG	GPR50	SALL2	IGF2BP1	IGDCC3	CRABP1	TEAD2	HMX1	MSI1	CRABP2	RCOR2	USP50	CNTFR	DLK1	WDR86
osteoblast	OSTN	IBSP	SERPINA12	MMP13	BGLAP	TNMD	SP7	TNN	PANX3	KRTAP13-4	IFITM5	DMP1	GDPD2	KERA	PODNL1	CHAD	ESM1	COMP	CCN5	UTS2R	SLC36A2	FAP	CLEC3A	ADIPOQ	ANGPT4	SFRP4	GPR88	COL11A1	OLFML2B	WNT16	PTX4	CHRDL1	SHOX2	GALR2	OMD	ALPL	SLC26A7	FMOD	KNG1	ASPN	FGF7	WIF1	ADAMTS2	LEPR	COL8A2	PDZRN4	EBF3	OGN	PTH1R	DLX5
osteocyte	AMBN	PANX3	TNN	IBSP	ADGRF4	DMP1	ODAM	SP7	RSPO4	AMELX	AMTN	DYNAP	BTNL2	ANGPT4	ALX3	LRRC15	DIO3	TLR8	MMP13	DLX3	ALX1	WIF1	SIGLEC1	NTN3	TCHHL1	WFIKKN2	MANSC4	SLAMF9	DLX4	ENPP6	PMCH	DSPP	OMD	LHX8	ABCA9	MSX2	SP6	PTGFR	TLR7	CD300LD	TIFAB	ALG10B	APOBEC1	FCGR3B	FERD3L	STAB1	C3AR1	CCL24	KLKB1	P2RY13
ov	GAGE12F	OR5AN1	OR2AT4	OR6Y1	OR5AS1	OR2M3	OR4F17	OR14J1	OR2T12	OR2M4	OR5A1	OR8A1	GAGE13	OR2G6	OR5A2	OR8D1	MAGEA9B	OR1D2	OR52E4	OR56A1	OR52K1	OR1A1	OR5H8	H4C14	TP53TG3C	OR10G4	OR8G1	OR2L2	OR5B2	OR2A5	SSX4	OR10H1	SPINK9	OR6C4	SPDYE17	OR8B3	OR2AG1	OR4K17	OR1C1	SCGB1D1	GAGE12J	OR56A4	MAGEA9	GAGE1	OR2V2	POTEC	OR5AU1	TP53TG3B	OR4K13	THSD8
ovarian follicle cell	GSTT4	BEST2	SOX14	TREH	NXF2	HDC	CLC	NAT1	RS1	ART1	SOD3	CRP	SIX4	RGN	HRG	OCM	OXT	WDR62	GK2	INSC	SP7	SRL	SPX	RGR	CABP1	RAB19	PDP1	CPO	RNASEK	ALG10	SYP	NXF3	PSD	MED26	DYRK3	BVES	PAK3	CAPS	SMG5	TTK	ARX	KEL	MYC	AGO1	CIC	RBFOX1	DMTN	SPEN	E2F1	GRP
ovarian microvascular endothelial cell	CCL14	SELE	CCL23	CRHBP	TM4SF18	CLIC2	TNFRSF10D	ANGPT2	HSPA6	IFI44L	CLEC14A	VWF	MYCT1	GIMAP8	KRTAP4-11	MMRN1	HSPA2	CLDN5	CCL21	EMCN	CALCRL	CCN2	ADGRL4	GIMAP7	CLEC2B	SPARCL1	CCDC178	A2M	ZNF385D	TMEM255B	NLRP2B	AQP1	HSPA1L	ERG	HOXD1	SOX17	SNAI1	IFIT1	CYYR1	FAM110D	DLL4	RAMP3	TNFSF10	LPAR6	ADCY4	LCN6	CNTNAP3B	EDN1	PCDH17	HSPA1B
ovarian surface epithelial cell	CLEC18C	AMH	ATP4B	AQP8	GRIP2	MS4A18	ADGRF4	NR5A1	SLC39A2	AMHR2	UPK3B	PDYN	EPX	HSD17B3	LHX9	WT1	FBXW12	RSPO1	SIGLEC1	TCP11X2	CHRNA2	ANGPTL7	PRXL2B	IHO1	LBHD2	SBK2	CTCFL	ZNF705A	BNC1	VSIG10L	ARMH4	CES5A	WNT6	MYO1H	DHH	INHA	TBATA	HOXD9	LORICRIN	CST8	GSTA1	SERPINA5	MMACHC	PLA2G1B	ILDR2	CXCR2	APOA1	NPAS2	NLRC3	BMT2
ovcar4	IGFL1	FGF18	WNT7A	EDN2	KLK10	MMP7	TNNC1	LAMA3	CCNA1	EDN1	VSTM1	KCNK15	GPRIN2	DEFB4A	FABP6	SFTA2	APOL1	SLC34A2	GXYLT2	IL1R2	ST6GALNAC1	RAB42	PORCN	EPPK1	TMEM139	ANKRD1	ARHGEF35	NMU	CCN6	NUTM2E	MUC16	CD40	CDC42EP5	CXCL5	TFPI2	NRCAM	KCNC3	SLC26A2	ZNF787	CYGB	HMX1	KLRG2	PI3	GFOD1	ITGB6	GJB2	AJAP1	SIM2	SAA2	RTKN2
oviduct glandular cell	BEST2	TREH	GSTT4	CLC	SOX14	NXF2	ART1	OCM	NAT1	RS1	HRG	WDR62	RGR	HDC	SRL	CPO	SIX4	RGN	INSC	CABP1	SYP	CRP	PDP1	RETN	SPX	SOD3	ALG10	CSN3	AMPH	HEPH	RNASEK	MMP1	GRP	OXT	MED26	PSD	TTK	CAPS	FKBP14	AGO1	DPH5	ELP1	MYC	ARX	KEL	COL4A1	STAC	SP7	RAB19	CORT
ovsaho	FLG	FGF18	C1QL4	MAGEA1	EMX2	CAPN9	WNT7A	HMX1	AQP5	KLHL35	TMEM100	DOK7	FBXO2	AJAP1	PI3	S100A5	FOLR3	NEURL1	TMEM139	FAM107A	KLK6	CRYBB2	ZNF492	NOX4	UGT2B7	RBP2	C1QL1	SMKR1	PAX8	NMU	ZNF599	BMP7	ATOH8	CDH6	CERS1	CLDN16	RNF144B	ZNF503	LCE5A	NME8	CEND1	PLCD3	AP1M2	COL9A2	MEIOB	LYPD6B	S100A1	RAPGEF3	POTEG	TMPRSS3
panc1	DLL3	FBXO27	FOXE3	PPP1R14A	ONECUT3	OR51B4	CARD18	FBXO17	BARX1	VSTM1	CACNG8	CACNG6	ZNF780A	PLEKHG2	ARSI	MT1A	CAPN12	TM4SF18	CCDC74A	TESMIN	HOXB5	HOXB6	MALL	FAM72B	HOXC9	NPB	ASPHD1	ATP8B3	BEGAIN	CD70	CHD5	HOXC10	ARMS2	HOXC13	RGS20	HOXC11	DLK2	HOXC8	EID2	ZNF546	AKT2	FAM72D	PLAU	CYB5R2	SAMD4B	TBX1	PAK4	TNNT1	TIMM50	FOXL2
panc0203	NOX5	MYH15	AK5	PADI1	NT5DC4	KLK6	EREG	CALB2	LCE5A	LIF	CST6	RNF223	LY6K	MROH6	BLID	RNF224	ARL14	MYEOV	PRSS22	G0S2	UPK3B	ZNF74	FGF2	RHOD	GJB3	B3GNT3	MESP1	CDA	HOXB3	MELTF	MOK	SPRR1B	SH2D3A	LCN2	LAMA3	TGM2	CALHM3	PLAU	MILR1	PRSS21	HSBP1L1	A2ML1	F12	IVL	MAL2	CCDC57	SIM2	ABCC3	PDE4C	FOXQ1
panc0327	KRTAP4-1	IL37	CXCL5	SPANXC	TMEM92	SAA1	ERVMER34-1	CD163L1	EREG	MACC1	ZBED2	FBXO27	F2RL1	SPANXD	IL20RA	KRT81	TFF2	MELTF	SLCO1B3	CST6	CATSPER1	PRSS3	PRDM13	IL1A	FBXO17	LCN2	EPHB6	SAA2	PTHLH	POPDC3	SPRR2D	NCCRP1	HS3ST1	FOXL1	MALL	GPR87	HOXB8	PRSS22	SIM2	KISS1	PRSS21	GAL	SPOCD1	MYEOV	TMEM171	PI3	ITGA2	ZNF114	GSDMC	CLDN9
pancreatic a cell	CFC1B	GPR119	OR51A4	KCNK16	SLC30A8	PRRT1B	GCG	GPR148	GPR142	G6PC2	BRS3	RFX6	FFAR1	CRYBA2	MBOAT4	C8B	SSTR3	SCGN	TM4SF4	ABCC8	AVPR1B	TMEM236	PRLHR	PYY	PCSK2	SERPINA10	CLTRN	PAPPA2	FEV	UCN3	PPY	CFC1	KCNK17	OR52N5	AKAIN1	LOXL4	VWA5B2	SERPINA7	INS	TTR	IAPP	MAP3K15	LCE2B	SCGB2A1	OR51A2	NEUROD1	KDM4E	NPC1L1	AMER3	CRH
pancreatic acinar cell	PNLIP	AQP12B	AQP12A	PNLIPRP2	CTRC	CELA3B	CELA2A	CTRB2	CELA3A	PNLIPRP1	AMY2A	CELA2B	CTRB1	CPB1	CTRL	PRSS1	REG1B	PRSS2	CPA1	GP2	SYCN	PDIA2	CEL	RBPJL	CUZD1	PLA2G1B	CPA2	CLPS	REG1A	BNIP5	SERPINA3	AMY2B	KDM4F	REG3A	PTF1A	DUOXA2	SERPINI2	ZG16	OR10G7	CELA1	TMEM275	DUOX2	OR10G4	REG3G	OR8D4	TMED6	SDR9C7	TFF2	FGF21	CCKAR
pancreatic d cell	REELD1	CRYGB	SST	KCNK16	CD8B2	DKK4	SHISAL2B	GHSR	KRT40	PPY	INSYN1	ANKRD34C	PYY	AVPR1B	IAPP	CBLN4	INS	GPR142	GHRH	FGF20	SSTR3	ABCC8	SLC30A8	NKX6-3	CABP7	RFX6	NEUROG3	MMP21	PDX1	FAM166C	LCE2C	PCSK1	LAMB4	DIO1	HGFAC	SCGN	GCG	SERPINA10	CCDC188	GAST	CRYGD	ZIM2	TMPRSS6	C8B	SLC35G5	ASPDH	PLA2G2F	PCSK2	GALNT8	CTXN2
pancreatic ductal cell	SMIM38	SERPINA2	CELA2A	DUOXA2	PNLIP	SERPINA3	CELA3B	CEACAM7	BNIP5	CTRB1	CTRL	PRSS2	SYCN	CTRB2	CELA3A	CLPS	DUOX2	REG1A	PNLIPRP1	CPB1	PRSS1	UGT2B15	PNLIPRP2	SCTR	TRPV6	APCS	CPA1	SLC28A3	CFTR	AQP12A	REG1B	CEL	CTRC	UGT2A3	MMP7	LCE6A	HABP2	INS	HNRNPCL3	SERPINA5	KRTAP1-1	AQP12B	TM4SF4	HNRNPCL4	AMBP	CRISP3	PKHD1	MUC6	ZG16	SLC6A20
pancreatic endocrine cell	NPIPB8	OR4K14	NPIPB9	G6PC2	SLC30A8	OR13C9	GPR142	RGPD4	UCN3	CTRB2	INS	RGPD3	KCNK16	IAPP	PPY	RGPD1	CPA5	NR2E1	PRSS53	OR52N4	CELA3A	SSTR1	CELA3B	RFX6	ABCC8	CPB1	ERVV-1	INSM1	NKX2-2	REG3A	AKAP3	SST	GCG	GOLGA8S	SCGN	HNRNPCL4	TMEM132E	OR52H1	PDX1	DEUP1	PRSS2	SSTR3	RGPD2	SLC35D3	REG1B	PCSK1	PRSS1	POU5F1B	GJD2	SHISAL2B
pancreatic epsilon cell	GPR42	PPIAL4C	INS	GHRL	UCN3	OR2A4	TBPL2	TBC1D3C	SEBOX	REG1B	SST	ASCL5	SUMO4	CFC1	SHISAL2B	PDX1	DEPP1	NPIPA5	SCGN	IAPP	MAB21L4	PCSK1	ANKRD34C	POU5F2	TM4SF4	SPTSSB	SEZ6L	MSANTD1	HIGD2B	CDK5R2	VSTM2L	GCG	TMED6	RADX	CNGA3	FOXA2	ZNF396	CST4	NPAS4	HTD2	PPY	TM4SF5	NR0B2	APOH	A1CF	MBOAT4	VWA5B2	RGPD3	SMOC1	KCNH6
pancreatic pp cell	OR2T34	OR6C1	PPY	OR51V1	OR2A42	CFC1B	OR10G9	GPR119	SERPINA7	FAM90A26	PYY	KCNK16	OR1D4	GPR148	GPR142	DMRTC1	C8B	TP53TG3C	SSTR3	GCG	AKAIN1	SERPINA10	ABCC8	SLC30A8	TMEM271	MAGED4B	SCGN	CLTRN	FFAR1	SMIM32	AVPR1B	SHISAL2B	RESP18	NKX6-3	TM4SF4	RADX	OR4K3	PLA2G2F	PCSK2	HGFAC	CCDC198	HTN1	ZNF705B	IAPP	SCGB2A1	SERTM1	CTXN2	SPDYE2B	SLC2A5	SFRP5
pancreatic stellate cell	CELA2A	PNLIP	LCE2C	CELA3B	CTRL	PRSS2	CTRB1	SYCN	RERGL	PNLIPRP1	PNLIPRP2	CPB1	DUXA	CLPS	KCNK17	FHL5	CCL11	ZG16	AQP12A	CPA1	CEL	CELA1	SPRR4	GPR20	GJD3	COL5A3	PDIA2	GDF10	INS	LAMC3	ERVV-1	SPDYE16	FOXS1	LRRC55	FCAMR	HTRA3	DPEP1	OR51E1	PENK	CPA2	CCN4	GP2	PLA2G1B	CILP	PLA1A	PPP1R2B	ZNF469	SVEP1	ENTPD2	HSD11B1
paneth cell	DEFA6	RETNLB	ITLN2	REG3A	GIMD1	CA7	P2RY4	PRSS2	MUC2	OR2D2	GUCA2A	LYPD8	OTOP2	MS4A12	GUCA2B	URAD	CLCA1	BEST4	PLA2G2A	REG1A	CEACAM7	ITLN1	TPSG1	CA4	KRTAP5-1	KRT20	MS4A18	PPP1R14D	REG1B	BNIP5	ATOH1	TM4SF5	KLK12	CHP2	REG4	APOA4	REP15	BTNL3	MUC3A	OLFM4	CDH17	ZG16	RNF186	HEPACAM2	MT1H	CDHR5	PLA2G10	FABP1	CCL15	B4GALNT2
paneth cell of epithelium of large intestine	OTOP2	CA7	BEST4	LCN1	CEACAM3	OR8D4	MAB21L4	POTEJ	ABCG8	UBE2NL	USP41	OTOP3	NPIPA2	ADRA2A	B3GALT9	ABCG5	EIF2S3B	GALR3	OBP2B	KRT40	DMBT1	RPEL1	MT1B	GBGT1	ALOX12B	KCNK7	SERPINA7	DNASE2B	ADAMTS14	ADAM30	MMP10	RAB40A	ZNF878	MMEL1	HS3ST6	GAL3ST2	LGALS9B	WDR97	SPIB	VWC2	NBPF8	ULBP3	SLC25A41	SHLD3	ZNF888	OTC	MSLN	SLC17A8	IGSF22	SLC35D3
paneth cell of epithelium of small intestine	KRTAP5-1	OR13C4	OR8B12	PRAMEF14	PRRT1B	NMUR2	OR51B6	TRIM77	NUTM2F	ZNF705D	KRT74	TMEM272	DIPK1C	CTAGE4	OTOP3	RFPL4AL1	OR2A5	NKX6-3	ARSH	SMIM32	TRIM40	BLACAT1	OR2AG2	B3GALT9	ANKRD40CL	CBLIF	MUCL3	KRT72	GLYATL1B	ZNF888	OR5H14	EVPLL	OPRPN	OR2A7	UGT2B15	NAT8B	GOLGA8J	GLP1R	SLC22A13	CYP2C19	OR3A3	TBC1D3D	ONECUT3	UGT2B17	SULT1C3	MAFA	CCDC198	IFNE	CENPVL3	NKX6-2
paraxial cell	OR6X1	CHRNG	GJD4	CACNG1	CHRND	ITGB1BP2	TMOD4	KLHL40	KLHL41	MSGN1	SYPL2	MYF5	MYL1	EXOC1L	MYBPH	TCF15	TNNT3	FOXC2	RIPPLY2	UNC45B	CER1	PYGM	KLHL31	GPR101	FAM240B	NAT16	FERD3L	VGLL2	ARG1	COL2A1	SMYD1	SPRR5	MYF6	ASB12	PRKAG3	MYOG	MEOX1	TAFA3	NEB	TNNC2	KRTAP24-1	TAS2R3	RBM24	FGF8	HOXB1	ENPP6	THBS4	LMOD3	TBXT	HOXC12
parietal epithelial cell	AGRP	TAFA1	WNT16	HOXC5	BSPH1	BMP6	PTCHD4	PDGFD	GRID1	TRPM6	HOGA1	CCDC83	ADRA1D	ZC3H12B	SH3TC2	IL34	CDH6	PRR33	ROR1	ADAMTS16	SLC22A14	FOLH1	IGSF5	CHRM3	DDO	WT1	SPNS2	PAX2	IQCM	SLC25A21	CFTR	FMN1	SORCS3	PAX8	MEF2B	C1QTNF7	MROH5	SHROOM4	TRABD2B	CHSY3	CDH18	SYTL5	NKAIN3	METTL24	SDK1	GLIS3	AFM	MEIKIN	PNPLA1	TFAP2D

**pc3**	MSMP	PRAC2	HOXB13	KRT81	SIM2	PRSS3	KRT34	CLDN14	LRRC38	SERPINB7	GBX2	PKP1	KRTAP2-3	NUTM2E	TMEM255B	BLID	FZD8	KCNMA1	INHBB	FGF5	DKK1	TFF2	FST	TFF1	ZNF672	TRIM58	DPF3	FAM131C	KCNG1	WNT7A	C1QL4	FBXO43	G0S2	ZNF185	ZNF248	TGFBR3L	SMKR1	GPR176	MESP1	TRNP1	SPDEF	GPR4	CDCP1	PI3	TRPM2	DSEL	FOSL1	MAP6D1	ONECUT3	F12
**pdac**	KRTAP4-16	DYNAP	MUC17	DUOXA2	HNRNPCL3	LIPK	GKN1	ALPI	MUC3A	DCD	DUOX2	PTX4	CENPVL2	OTOP3	H2AC17	GKN2	TBC1D3K	H4C9	TM4SF20	OR9Q1	ONECUT2	EIF2S3B	CTAGE8	CLDN18	ANXA10	DMBT1	SLC6A20	HNRNPCL4	TM4SF4	CTSE	RAMACL	MIDEAS	SLCO1B3	H3C6	RPEL1	IGSF23	CLDN2	TAS2R50	H2BC11	PSG4	H2BC6	RAB6C	ONECUT3	TBC1D3E	LYPD8	TNMD	H2BC5	EPRS1	GCNT4	TFF2
**pecapj15**	LGALS7B	PTTG2	ALPG	SHCBP1L	PRSS21	BCL2L10	ALPP	SLC16A8	TM4SF19	KRT13	CCL26	HBQ1	SLC47A2	SMC1B	RNF212	TMEM270	OR2B6	ARHGEF33	EVPLL	SYCP2	B4GALNT2	LRRC69	AKR1C2	SLC52A1	CFAP251	LY6K	IL1F10	SOX15	KRT4	NTF4	HPDL	NEURL1	GREM1	NKX1-2	ERVMER34-1	PNLDC1	RIBC2	KCNJ3	GPR87	LGALS7	GABRE	PRDM13	KRT3	TINCR	CSTA	NRG4	KRT17	TRIM16L	CBLC	FGF23
**pecapj49**	SPANXB1	OR4C6	SPANXA1	KLK5	KRT9	RAET1G	LRRC38	RNASE7	FGF19	IL1F10	SERPINB3	SUN3	SPANXA2	SERPINB4	LCE1C	GPR87	IGFL1	KRT6A	CSTA	ARSI	PSG4	SOX15	KHDC1L	FGFBP1	ANXA10	KRT14	IL36G	ANXA8L1	CALML3	PGLYRP4	DLK2	NTF4	ZBED2	KRT5	CALHM3	MAGEA11	SAA1	KRT17	FAM83A	SAA2	SERPINB13	CCL26	PLCXD2	MMP28	ZP3	S100A2	MAGEA3	TP63	KRT13	NKX2-8
**peptidergic neuron**	ALK	CHAT	CPO	MIP	PROC	CORIN	CRP	GRP	CLC	RAB26	HRG	EYS	ART1	OTP	LRP4	GK2	SPX	HEPH	RBFOX1	OCM	RS1	CABP1	SYT4	NAT1	VSX1	DDC	UNC79	IPP	PDP1	DYRK3	SYT7	DAO	MID1	MED26	GIP	SYN1	HASPIN	TREH	DRGX	WDR62	SLC5A11	ANK2	CSN3	PDE1C	SYT12	SYT1	GSC	CADPS	AGO1	RAB19
**pericyte cell**	HIGD1B	TRPC6	FAM162B	CCDC102B	ATP13A5	FHL5	TBX5	COX4I2	GUCY1A2	TRPC3	KCNJ8	TRPC4	LAMC3	PDE3A	RASL12	GPER1	KCNK17	LRCOL1	PDGFRB	DUXB	CACNA1C	IRAG1	RGS5	PRR16	GJA4	HEYL	NOTCH3	OR2A42	MYH11	AOC3	ADAMTSL3	PRKG1	NDUFA4L2	FOXS1	ADGRD1	GUCY1B1	ABCC9	SEPTIN4	PALM2AKAP2	TBX4	LMOD1	TBX2	RERGL	ANKRD62	EDNRA	GUCY1A1	INKA2	PDE8B	RNF152	SMIM41
**perineuronal satellite cell**	KLK9	KCNA1	TMPRSS5	RASGEF1C	GPR37L1	PLP1	CDH19	GFRA3	COL20A1	CMTM5	SCN7A	LGI4	SLCO1C1	MYOCD	SOSTDC1	RSPO4	MATN4	KCNA6	SFRP5	SLITRK1	DHH	SERPIND1	ASPA	GFRA2	KCNA2	IGSF9B	SLC22A8	FABP7	EGFL8	KRT28	ATP1A2	PANX3	ART3	GPR20	ADAM11	PHOX2B	OLIG2	GPER1	INSC	AMBN	PAQR6	KCNJ10	MMD2	TMEM88B	ALX3	SOX10	COL8A2	SLITRK6	GJC3	CHADL
**peritubular myoid cell**	PROK1	TSPY8	TEX13A	SPRR4	KRT3	LCNL1	PRM2	ANHX	DEFB123	NXF2B	KIAA1210	SSX2	ZCCHC13	PRM1	LELP1	CST9L	LYZL1	INSL3	ZDHHC19	KCNMB1	LAMC3	GOLGA8Q	TFDP3	TNP1	TCF23	TPD52L3	FATE1	MMP23B	TPPP2	PLA2G5	BOD1L2	UBQLNL	DPEP1	TSSK1B	DEFB119	MYOZ3	RHOXF1	MYH11	SLC51A	OR13C5	BPIFA3	RNF133	PTGDS	NT5C1B	SERPINA5	KLK15	EVPLL	UBL4B	MYOCD	GLI1
**perivascular macrophage**	CCL24	ADGRE1	PF4	C3AR1	TREM2	FOLR2	TNFSF18	STAB1	CCL8	MRC1	P2RY13	FCGR3B	P2RY12	F13A1	CX3CR1	GPR34	KRT26	CLEC10A	LYVE1	CRYBA4	CSF1R	MS4A7	TLR7	CD163	LTC4S	CRYBB1	TSLP	TLR9	FCGR2B	SLCO2B1	TIFAB	ABCA9	P2RY6	C1QC	CCL7	PLA2G2D	PLD4	TLR8	P2RX7	HPGDS	C1QB	C5AR1	CD300LD	CD86	APOBEC1	ASB10	C1QA	CD33	KCNK13	LILRA5
**peyer's patch follicular dendritic cell**	IL22RA2	PLA2G2D	ADAMDEC1	SERPINA11	MCEMP1	SLAMF8	TREML4	ASB2	IL4I1	ADGRG5	CFP	DNASE1L3	C1QC	NXPE4	PLA2G7	CLEC5A	CCR1	PILRA	HS3ST2	MEFV	C1QA	C1QB	ITGAX	MSR1	CD33	CCL17	FPR2	NR1H3	LRRC25	CCR2	AMZ1	IL18BP	GDF3	P2RY13	APOC4	TGM1	CSF1R	PTAFR	CD300LF	CCR9	LTC4S	IL1RN	SLC2A6	IL1A	TMEM26	SPI1	MPEG1	TSPAN10	CST7	CFB
**phagocyte**	TMEM235	H2BC3	KRTAP10-11	MRGPRX4	SLCO1A2	HS3ST4	TBC1D3I	OR13C5	OPALIN	OR5H15	CCDC200	IFNA13	PPIAL4H	MOBP	ST18	ZNF735	FAM237A	CDH20	SYNDIG1	CNDP1	ZNF365	RNASE10	DIPK1C	MAGEC3	SMIM35	TP53TG5	STYXL2	SIGLEC8	SEPTIN4	ANO4	ZNF536	KCNH8	GLDN	LRMDA	SLC24A2	HCG22	GRM3	CNTNAP4	MOG	PEX5L	SLC5A11	TMEM144	LRRC63	LINGO1	NUTM2F	KLHL32	OR7A5	MARCHF1	ZDHHC11B	CNTN2
**pharyngeal mesodermal cell**	FGF8	EPOP	CER1	MOGAT2	ARG1	CFC1	TCF15	ALX1	HOXB1	FOXC2	CYP26A1	PRRX2	MYL7	CRABP1	TBX1	PRTG	HAPLN1	RSPO1	IRX1	MYCN	ZBTB12	DUSP9	RIPPLY1	LEFTY2	IRX5	APLNR	FOXF1	LIN28A	IGFBPL1	XKR5	FIGNL2	MACROH2A2	SELENOH	JPT2	FERD3L	RIOX1	RCOR2	SFRP5	HPF1	MAIP1	GRIFIN	TEAD2	SPOUT1	GON7	HOXA1	AUNIP	C1QTNF12	WDR86	COL26A1	SP5
**photoreceptor cell**	TMEM244	AIPL1	GNAT1	IMPG1	RBP3	AANAT	TRIM49D1	TRIM49D2	PDC	OR56B4	IMPG2	OR2M7	RCVRN	NRL	RP1L1	EIF4E1B	RAX2	CPLX4	CLUL1	RTBDN	CNGA1	CABP2	GUCY2D	PRPH2	PCARE	CRX	SLC1A7	CACNA1F	ABCA4	GRM6	OPN1LW	CRYAA	SAMD7	RDH8	OR13C2	NR2E3	OR10T2	THEG	NUTM2F	TULP1	CDHR1	PPEF2	TRIM49B	USH2A	SCGB1D4	PSKH2	MPP4	OR4K1	GNB3	ARR3
**photoreceptor precursor cell**	GALP	ATOH7	TMEM262	CHRNA3	GDF9	NEUROD4	CHRNB4	PTH2	SERPINI2	GABRR3	CABP7	RCVRN	INSM2	PRSS33	IMPG2	GFY	ISL2	FAM166A	RAX	KCNV2	HCRTR1	GNAT2	AANAT	SNX32	CRX	PRR19	SIX6	B3GNT4	EYS	SLC6A5	KRTAP4-1	SRRM5	GNB3	JSRP1	MYL5	TMEM31	DBX1	MCMDC2	LRIT3	NPFFR2	PPP1R17	PRCD	KCP	SNX22	GPR62	SSTR2	TMEM244	CIB4	TCEAL7	PDE6C
**pigment cell**	SLC45A2	ASMT	MCHR2	PSMA8	TFAP2E	SULT6B1	GPR143	DRD3	SMIM29	OPN5	GAPDHS	RAB41	SLC22A31	FOXD3	TFEC	COX6B2	WBP2NL	SOX10	SLC22A2	ANXA13	TSPAN10	SDCBP2	CDK15	LIPF	DCT	CHAC1	BCL2L10	RHAG	EIF5A2	EIF4EBP3	GLOD5	FABP3	NCCRP1	PRLH	AKR7A3	NKAIN1	RNF175	CHP2	RIPPLY1	PRSS16	LCN15	CNPY1	CREG2	SLC37A2	SLC38A11	PLA2G15	SPOCK3	PAIP2B	BCO1	RIMKLA
**pituitary gland cell**	MC3R	PRL	CRHBP	POMC	RNF183	FAM221B	ECEL1	LHB	RESP18	ODAPH	HAPLN4	PTX4	ZCCHC12	UTS2	POM121L2	CGA	RXFP1	LRRC10B	NEUROD4	SLURP2	MT3	CAMK1G	MROH5	CCDC154	PDYN	TMEM63C	FGF21	MRGPRE	ASB4	TMEM266	ALDH1L2	GNRHR	EPHA10	GDAP1L1	ERFE	RBM20	STK32A	CALR3	SCG2	CHAC1	SRXN1	TMC3	MROH2A	CHGB	NRSN1	SLC6A17	ESPNL	KLHL30	CHST8	HMCN2
**pk45h**	CD163L1	KRT33B	FOXL1	MT1A	ZNF185	BLID	FOXD1	GDF5	HES7	FOXL2	NFE2L3	MT1M	KRTAP4-8	MELTF	ADAMTS6	SLFN12	TMEM78	PRSS3	MYEOV	PODXL	KRT34	IL11	FLNC	NT5E	ANKRD1	NCR3LG1	ZNF696	EEF1A2	EPHB6	KCNJ14	CEP55	FGFRL1	NETO2	CDCP1	IL37	CCDC85C	MYBL1	CCDC169	ZNF469	TMEM249	MUC12	TOR4A	AK5	DKK1	TRIM7	DIAPH3	CHST3	FAM131C	GAL	TMEM158
**pk59**	KRT3	KRT86	KRT13	CST4	NT5DC4	TFF2	CEACAM6	MUCL3	SPINK7	B3GNT3	KLK10	CST2	FAM25A	KRT6A	S100A5	IGFL1	PTK6	FAM83A	MROH6	WNT7A	FLG	IGFL2	RNF223	ZNF185	CALB1	MUC16	CST1	SCEL	CALHM3	PRSS3	NMU	FCHO1	NUTM2D	SPRR3	TNFSF15	GOLGA7B	OXTR	CD163L1	GAST	ZNF787	MALL	CCDC68	MUC4	CPA4	PTHLH	IL22RA1	FERMT1	PTGES	PTPRH	KRT80
**placental villous trophoblast**	SLC22A11	ERVW-1	ERVFRD-1	TMC1	EZHIP	ADAMTS19	CALHM4	CYP19A1	PAGE4	ISM2	ERVV-1	NAA11	RGPD1	SLC13A4	GRAMD2A	LRRC53	CSH1	LGR5	COL21A1	PGAP6	IL1RAPL2	OR8G1	CSH2	OR51B5	TUBB8B	SVEP1	NOX3	SIGLEC6	ZFAT	SLC19A3	FBN2	SLC13A3	NIBAN2	TP63	MTARC1	STS	VGLL1	PCDH11X	CRYBG1	SMIM43	SLC6A4	SLC52A1	RHOBTB1	RADX	GCM1	ERVV-2	ZNF730	NRK	LRP2	USP43
**placode cell**	BHLHA9	WNT6	EPOP	ERAS	CLDN6	CDX4	FGF8	MOGAT2	LRRN4	HAPLN1	HESX1	DNMT3B	MSX2	LIN28A	EVX1	FGF17	FOXD4	NECTIN2	TAL2	JPT2	HOXB1	PRTG	ZBTB12	DGKK	UPK1A	GRHL3	DLX5	FOXI2	SRMS	NKX1-2	CLDN9	SP6	SELENOH	RIOX1	MSX1	SMTNL2	SMIM10L2A	CRABP1	GJB3	HPF1	HOXA1	C1QTNF12	MAIP1	CDX1	SP5	SPOUT1	SALL4	MYCN	AUNIP	GON7
**plasma cell**	LCE2B	LCE2C	LCE2D	LCE1A	FCRL5	TNFRSF17	IGLL5	MZB1	KRTAP10-3	KPRP	NUGGC	IL26	JCHAIN	RHEX	DERL3	POU2AF1	MEI1	FLG2	TEX52	KRTAP10-2	KRTAP4-6	LCE1B	MAGEB17	LCE2A	ZNF215	LCE1D	HLA-DOB	AMPD1	ANKK1	JSRP1	GPR15	FCRL2	OR6M1	OR6A2	ENAM	PNOC	TENT5C	INSYN2B	IRF4	SLAMF7	KRT74	CCR10	TNFRSF13B	FDCSP	KRT38	CDRT4	TPTE2	KRTAP4-3	KCNN3	SPAG4
**plasmablast**	TNFRSF17	IGLL5	NPIPB8	GPRC5D	JCHAIN	NPIPB7	MZB1	GPR25	DERL3	POU2AF1	TNFRSF13B	H2BC10	KLHL14	FCRL5	GPR15	NUGGC	ZBP1	IRF4	CPNE5	CD79A	MEI1	CD38	UQCRHL	ZBTB32	HSH2D	LIME1	NPIPB6	SLAMF7	CCR10	PNOC	BMP8B	CD27	EAF2	HLA-G	LILRB1	LTK	CD19	GNG7	H2BC7	TMEM156	CEACAM21	ABCB9	PKHD1L1	NCF1	H4C13	KCNA3	BLK	ENAM	PLAAT2	OAS1
**plasmacytoid dendritic cell**	IFNA6	IFNA14	IFNL2	IFNA2	LILRA4	CLEC4C	H1-6	IFNA16	IFNL3	IFNA7	IFNA4	IFNA13	TLR9	IFNA5	PTCRA	ASIP	LCNL1	OR1D5	NIBAN3	KCNK17	RHEX	GAPT	LAMP5	PLD4	SPIB	GZMB	CCR9	CD300C	RNASE6	NME8	ADGRG5	OR56A4	TLR7	HAVCR1	SCT	OR3A3	MYMX	IRF4	MPEG1	LILRB4	DCANP1	HMHB1	IL3RA	OR5AN1	TTC24	TCL1A	IRF8	JCHAIN	MS4A6A	P2RY14
**platelet**	SCGB1C2	TUBB1	AQP10	TREML1	PF4V1	PPBP	GP9	SCYGR4	GP1BA	TRAPPC3L	SLFN14	GP6	PTCRA	LGALS12	BEND2	TNFSF4	CLEC1B	H3C10	CMTM5	MPIG6B	DYTN	TRIM40	PF4	SPDYC	TRIM58	LY6G6F	GRAP2	MAP3K7CL	H2BC11	RGS18	NAT8B	F13A1	SELP	ITGA2B	ALOX12	CD226	KIR2DL3	TMEM40	CABP5	PTGIR	OR2B6	GFI1B	ENKUR	FAXDC2	GATA1	NRGN	TMSB4Y	GP5	TUBA8	NFE2
**pluripotent stem cell**	KRTAP4-3	NANOGP8	HSFY1	DKK4	SEMG1	ZSCAN10	TDGF1	NODAL	VRTN	OR52E4	DPPA4	OR5B17	FGF4	OR10AG1	POU5F1	CENPVL2	NANOG	GCSAML	HHLA1	SPDYE11	OR1D2	CNMD	KRTAP10-2	FGF19	PRDM14	TREML1	ZFP42	MAGED4	SHISAL2B	KRTAP10-1	TRIML2	USP44	UTF1	GDF3	SLC7A3	FOXH1	ZNF730	MAGEB17	MAGED4B	OR52N2	DMRTC2	ERAS	OPN1MW2	PRAMEF7	GLB1L3	OR5H8	OR51Q1	CA14	UPK3BL2	MYH1
**pneumocyte**	KRTAP5-2	WFDC12	SFTPA2	FOXI3	SFTPC	SFTPB	GGTLC1	SFTPA1	GPR55	C4BPA	SCGB3A2	SLC22A31	SFTPD	SLC26A9	CHIA	TCIM	SFTA2	UNC5CL	MFSD6L	CYP2D6	CCL13	NKX2-1	PGC	ALOX15B	GPRIN2	CXCL9	ATP5MGL	VNN2	CFAP410	SIRPG	CXCL17	AGER	SLC34A2	NCMAP	NME8	ETV7	HABP2	SCGB3A1	PPP1R14D	UBE2U	CLDN14	RNASE1	CCDC180	GLYATL2	DEPP1	UBD	CEACAM16	LAMP3	RTL10	LAIR2
**podocyte**	NPHS2	SEMA3G	NPHS1	APELA	TBPL2	WNT8B	HS3ST6	SLC6A18	SNX31	SLC7A13	CDH16	R3HDML	CLTRN	COL4A4	RNF183	SHISA3	TCHHL1	GLYAT	DIO1	TMEM174	WT1	CLDN8	DDN	TTC36	SLC5A2	ENPEP	PKHD1	UMOD	ERICH4	GUCA2B	FXYD4	SPP2	SLC4A11	COL4A3	TDRD5	BST1	PTH1R	SLC22A12	SLC13A1	CLIC5	SLC34A3	CYS1	SLC7A9	RANBP3L	CLDN2	AQP1	RNF186	PLSCR2	NPNT	DAO
**polar body**	SOX14	RGN	NXF2	OCM	RGR	CPO	CRP	CLC	HDC	NAT1	WDR62	BEST2	RS1	SIX4	ART1	MOS	GK2	NXF3	HRG	ALG10	TREH	CABP1	SRL	GSTT4	SPX	SYP	TTK	OXT	ENDOU	PDP1	GRP	INSC	HEPH	SHF	MED26	RAB19	DYRK3	PSD	CSN3	AGO1	REEP1	CAPS	MMP1	GSTO2	TAF4	TUB	OMD	CIC	GIP	DPH5
**polydendrocyte**	OLIG2	GPR17	C1QL2	OLIG1	NEU4	PLPP4	MATN4	CSPG5	GSX1	FZD9	GPR37L1	DBX2	BCAN	AVP	NMS	IGSF9B	CHST5	LHFPL3	NXPH1	KCTD4	C1QL1	TNR	SCRG1	GJC3	CHADL	TAFA1	MMD2	KCNIP3	MRO	PLPPR5	BRINP3	TMEM100	SUSD5	LRRTM3	NTSR2	C1QL3	NLGN3	RLBP1	MT3	SOX8	SLC7A10	TIMP4	WSCD1	SLITRK1	FAM181B	PCDH11X	LUZP2	GRIN3A	NKX6-2	RPRM
**pp cell**	OR10W1	OR5C1	OR5M8	OR6C65	PPY	OR2T2	SSX7	OR2T35	PBOV1	MBOAT4	CFC1B	NLRP9	OR10J5	RFX6	GPR119	SNURF	SLC30A8	GHSR	KIAA0319	G6PC2	ELOA2	SERTM1	SCGB2A1	SCGN	GPR148	OR11H1	F11	OR52H1	ABCC8	OR11H2	AMER3	TM4SF4	ZNF177	SPATA31D1	NPFFR2	RTL9	SHISAL2B	OR6S1	OR8K5	TMED6	INS	SST	KCNK16	ANO5	KIAA0408	PCSK1	KRTAP10-3	KRTAP10-12	BMP8A	FOXD4L3
**prad**	OR51E2	KLK3	KLK4	KLK2	TRPM8	OR2B2	ALOX15B	PABPC1L2B	KRTAP4-9	FOLH1	SEMG2	IFIT1B	CYP4F2	KLK12	MSMB	HOXB13	GLYATL1	NKX3-1	POTEB3	HSD17B6	ZACN	CHRNA2	PRAC1	CRISP3	TBX10	DNASE2B	CST1	KLK15	POTEJ	SLC45A3	OR2W3	LMAN1L	OR51E1	HBD	RLN1	PRICKLE4	DDTL	SPDEF	TBC1D3	FUT5	KRTAP4-7	UGT2B28	PPBP	AZGP1	LRRC26	TMPRSS2	SFTPA2	ZNF8	SCGB1D2	POTEH
**pre-conventional dendritic cell**	LILRA4	CLEC4C	ASIP	PTCRA	TLR9	KCNK17	SCT	IRF4	LILRB4	GZMB	PLD4	IL3RA	LCNL1	TTC24	LAMP5	GAPT	RUFY4	NME8	LILRB1	SPIB	LRRC26	IGFLR1	JCHAIN	ADGRG5	DERL3	ZFAT	PHEX	RNASE6	GPR31	MS4A6A	CCDC183	DNASE1L3	MPEG1	HMHB1	TNFSF13	MZB1	TLR7	SMPD3	KCTD19	LILRB2	CLIC3	TMIGD2	LTK	TCL1A	GPR183	CD4	WNT10A	SIRPB1	HLA-DQA2	WDFY4
**pre-natural killer cell**	TSPO2	TRIM10	NXPE2	RHD	GYPA	NCR1	KLF1	SLC4A1	KEL	HEMGN	RHAG	CTSE	ALAS2	TBX21	KLRK1	ICAM4	ERMAP	GATA1	EOMES	ABCB4	PKLR	GPR150	IL2RB	CD160	PDIA2	TNFSF18	ITGAD	CCR2	KLRC2	TAL1	MFSD2B	GFI1B	ERFE	ASB17	CCR5	QRFP	TXK	CPOX	GPR15	EPOR	SH2D2A	SAMD3	E2F2	MS4A15	PGLYRP2	LAX1	CRTAM	ABCG4	TSPAN33	NEIL3
**preadipocyte**	TMDD1	KRTAP13-4	CCL11	CCN5	SULT1E1	COL6A6	COL6A5	CLEC3B	ADH1B	TNXB	AGTR1	SCARA5	MYOC	DPT	GDF10	PODN	CCN3	ABCA6	ABCA8	SVEP1	CILP	BMPER	COL5A3	HTRA3	COL15A1	MMP27	FAM180B	OGN	PLA2G2E	ADAMTS5	CCN1	ANGPTL5	AADAC	GSC	PI15	MEDAG	INMT	ABCA9	CCL7	VIT	SLC66A3	DEPP1	SRPX	DPEP1	PENK	APOD	ECM2	FGF10	GALNT15	TNFAIP6
**prechondroblast**	GDF5	COL8A2	AGTR2	CHST5	MKX	BARX1	GRPR	SCX	SFRP2	PAMR1	SSC5D	FGF21	LRRC17	MATN1	CAPN6	FBN2	FGF16	EGFL6	GFRA4	GAS2	TRABD2B	SCRG1	FIBIN	GAS1	PAX9	COL2A1	VWA2	EPYC	C1QTNF3	MFAP4	COL9A2	OSR2	SHISA2	HOXA11	FMOD	NDUFA4L2	COL12A1	PRSS35	DLK1	CRLF1	ANGPTL1	P4HA3	CBLN2	COL11A1	P3H3	ADAMTSL1	LRIG3	ADAM33	SULF1	HTR1B
**precursor b cell**	HMHB1	OR4F5	PRAMEF14	MMP8	GMCL2	PPP1R2B	RAG2	RAG1	IGLL1	CD177	PRG3	CAMP	GOLGA8K	VPREB3	H3C4	FCRLA	ZC3H11B	TASL	NIBAN3	HAVCR1	H2BC13	MMP9	IL12A	FPR2	CD19	VPREB1	GPR25	RGPD1	RAB6D	SPIB	ARL5C	LCN6	GOLGA8T	CALHM6	MYL10	GOLGA8J	DNTT	POTEJ	PAX5	IL36G	TCL1A	DOK3	CD300C	CCR9	RAMACL	OPALIN	COX6A2	BLK	FOXD4	CD79B
**pregranulosa cell**	CCDC182	CST8	NR5A1	LORICRIN	KCTD14	AMHR2	TMEM114	LHX9	WNT6	METTL24	LGALS7	WT1	RSPO1	C1QTNF7	DCAF12L1	GNG13	LBX2	CST9	KCNG4	GATA4	EMX2	TGM3	WFDC13	AQP12A	TMEM174	CPA2	GABRA4	TULP2	SOHLH1	DCAF12L2	CRIP3	NRTN	SHBG	DOC2B	CCDC61	WNT4	CCNJL	PNLIP	SP5	INHA	ETNK2	NR0B1	PNLIPRP2	RASSF10	KCNT1	LZTS1	HMGCS2	DDIT4L	CCDC42	PANK1
**preodontoblast**	KRTAP10-10	AMBN	RSPO4	PANX3	SP7	AMELX	ALX3	ANGPT4	KRTAP5-3	TAAR6	DLX3	ENPP6	DIO3	DLX4	LHX8	ALX1	WFIKKN2	OLFML1	DSPP	KRT82	GPHB5	BTNL2	SCUBE1	SP6	DMP1	WIF1	ALX4	KRTAP2-4	MMP13	MSX2	MKX	ANKRD63	MATN4	S1PR3	PRSS35	OMD	INSC	PROKR2	RSPO2	ADAMTS3	FAM167A	SLC2A7	SATB2	FMOD	VIT	ECEL1	SIGLEC1	ADAMTS20	TNN	TLR8
**presomitic mesodermal cell**	MSGN1	CDX4	MYF5	CCN6	HOXB1	TBXT	OR52M1	FOXB1	NKX1-1	TBX6	FOXC2	EVX1	HOXD4	FGF3	FGF8	KRTAP12-2	HES7	VENTX	FOXD4L1	GLYATL1B	GPR101	SPRR5	TKTL2	WNT8A	HOXC4	FTMT	TUBA3C	CLDN24	KRTAP4-7	TAS2R3	WNT3A	TLL2	HOXC10	MMP3	APLNR	HOXD10	NMRK2	KIF26A	TAFA3	OPTC	NTSR1	DSPP	IFITM5	HMCN2	RHCG	HOXA1	CDX2	SP5	CDX1	MOGAT2
**primary spermatocyte**	CT47B1	MEIOSIN	TEX13D	MAJIN	RBMXL3	SSX3	CT55	DCAF8L1	FAM9A	PRSS38	TEX13C	MAGEA9B	TUBA3D	VCX3A	MAGEB6	H2BW1	TERB2	VCX2	PRR23B	FAM9B	RAD51AP2	TSPY3	DMRTC2	VCX3B	PRSS50	TPTE	TUBA3E	SPATA22	PRM2	H2BW2	FTHL17	TEX19	RBMY1E	CT45A10	TEX101	OTUD6A	RNF212B	TMEM225B	VCX	DPEP3	ZPBP2	TOPAZ1	BOLL	MEIOC	TEX12	H2AC1	PRDM7	HORMAD2	PRM1	GAGE2A
**primitive cardiac myocyte**	NKX2-6	TLX1	FGF8	SPIN2A	NKX2-5	MYL7	LEFTY2	ASB4	IRX1	HAPLN1	IGFBPL1	LCE3B	PRRX2	ARG1	HOXB1	APLNR	PRTG	TBX1	GATA5	ZBTB12	PLA2G4B	CFC1	DUSP29	HAND2	TNNI1	IRX5	FIGNL2	LHFPL1	POPDC2	MYL4	MYLK3	ISL1	SMYD1	CLDN25	MYH7	PWWP3B	TMEFF1	RBM24	FGF10	RCOR2	LRRN4	CAPN6	DUSP9	TNNT2	MOGAT2	IGDCC3	LIN28A	MYCN	MPPED2	CRABP1
**primitive erythroid lineage cell**	KEL	SLC25A21	SLC4A1	SPTA1	SPTB	PKHD1L1	PKLR	RELN	RHAG	ERMAP	SLC30A10	ST6GALNAC3	LEXM	RHD	ANK1	GYPA	CACNA2D3	HEMGN	ALAS2	PIP5K1B	NXPE2	ABCB10	SLC22A4	ANO1	SLFN14	SLC16A10	ATP8A1	ADRA2B	FRRS1	CAMK1D	KNL1	CCDC141	TRIM10	TAL1	TSPAN33	SLC22A23	PROKR1	RAD51B	DPF3	ARHGAP23	DIAPH3	NMNAT3	EPB41	BMP2K	EPB42	SLC39A8	SNCA	CDK8	GAREM1	SLC6A9
**primordial germ cell**	SBK2	STRA8	RBMXL2	DPPA3	USP26	DAZL	UTF1	FKBP6	ZNF705A	TRIM77	FBXW12	TUBA3E	MAGEB4	SLC25A31	HCRT	GRIFIN	LEFTY2	NANOG	STK31	ASZ1	POU5F1	ARL14EPL	KLK12	MAGEB16	SLC28A1	ASB18	DND1	REC114	RBM46	SMC1B	SYCP1	SOHLH2	NANOS3	TKTL1	VSIG10L	GLYATL3	DPPA4	DMRTC2	PRXL2B	M1AP	DMRT1	FIGLA	DDX4	TEX14	DPPA2	ZGLP1	RNF17	MOV10L1	SYCE1	ADAD2
**pro-b cell**	GMCL2	PRAMEF14	IGLL1	H2BC13	VPREB1	SPDYE17	RAG2	SETSIP	RAMACL	RAG1	VPREB3	HMHB1	DNTT	USP41	OPALIN	LCN6	EIF2S3B	H3C4	NIBAN3	ZC3H11B	TASL	TRIM74	NPIPB8	MYL10	SPDYE2B	IL12A	OPRPN	CD79A	GCSAM	CD79B	PAX5	CD19	UBE2L5	TAS1R2	TCL1B	NPIPB9	PRG3	BLNK	PPP1R2B	SPDYE16	CLDN17	KRTAP4-12	BFSP2	POU2AF1	H4C11	INKA1	CALHM6	NBPF4	PPIAL4G	H4C8
**pro-t cell**	KIR3DL2	KIR2DL1	KIR3DL1	CXCR1	OR4D1	KLRF1	KIR2DL3	SH2D1B	GZMH	KLRC3	FGFBP2	FCRL6	CD8B2	LIM2	UTS2	LAIR2	PRF1	NCR3	XCL2	FCGR3A	S1PR5	KLRC4	PTGDR	GZMB	APOBEC3H	GZMA	SIGLEC7	GPR25	GZMM	IFNL1	GPR174	TBX21	NMUR1	MYOM2	LEXM	NBPF26	CD247	KLHDC7B	FCRL3	ZAP70	SAMD3	PAXX	THSD8	AJM1	KLRD1	CTSW	SLFN12L	CYBC1	CX3CR1	LIME1
**proerythroblast**	MMP8	CD177	IL36G	CEBPE	FPR2	ASPRV1	CLEC4D	MMP9	CD300LD	PGLYRP1	TREML4	CXCR2	LILRB3	PHOSPHO1	CD300LB	PADI4	LILRA6	PROK2	RASGRP4	CHI3L1	TARM1	LTF	CD300LF	CLEC5A	FCGR3B	GPR141	PILRA	LRG1	CLEC4E	LILRB4	TSPO2	PRAM1	OSM	CCR1	ELANE	C5AR1	NFAM1	HK3	BMX	CLEC12A	SLC36A3	ITGAM	FFAR2	FPR1	CXCR1	MS4A3	NCF4	NFE2	TNFAIP8L2	DOK3
**professional antigen presenting cell**	GPR31	PRAMEF17	KRTAP5-5	FCRL1	PRAMEF6	KRTAP22-2	KRT74	CCL22	CXCR5	KRTAP20-2	CD19	IL12B	DCSTAMP	TNFRSF13C	MS4A1	IL9R	GPR33	OCSTAMP	BFSP2	BTLA	KRTAP4-7	BLK	FCRLA	WDFY4	XCR1	TLR9	CD22	CCL17	MEFV	GPR18	CD79A	TASL	SLAMF9	NOX3	CIITA	FLT3	CD72	ARL5C	TNIP3	CD79B	LTA	ZC3H12D	CD80	KRTAP1-3	CACNA1S	CCR6	LY86	ZNF366	FCMR	NAPSA
**progenitor cell**	BSX	OR6T1	C8A	URAD	C8B	HAO1	OR1L1	SUPT20HL1	TAS2R8	NKX2-6	ARSL	TAS2R9	KRTAP24-1	ISX	ADH1C	PPIAL4A	C8G	PMP2	KRTAP13-4	SEPTIN14	BTNL3	FABP1	PNMA6F	MYOC	SETSIP	CST9	SLC39A5	GADL1	PLA2G2A	RHEX	MC2R	OR2B2	ARMH1	PRORY	KRTAP4-8	SIGLEC11	HEPN1	SLC5A8	GJB1	CHP2	FAM221B	NR5A1	DSPP	OLFM4	NOX1	MUC3A	CCL11	INKA1	FGF20	GATA5
**progenitor cell of endocrine pancreas**	PAX4	NEUROG3	RFX6	VWA5B2	FEV	NKX2-2	KRTAP4-16	KCNJ11	GPR119	NKX6-1	GCK	INSRR	GPR142	INSM1	PLA2G2D	MBOAT4	GJD2	SLC35D3	GRIN2C	PLA2G2F	ZFP92	KRTAP17-1	FFAR2	CER1	BAIAP3	FAM163A	MYT1	BTBD17	CRYBA2	CACNA1H	NEUROD1	ABCC8	NHLH1	MLXIPL	FFAR4	OTOP3	SLC38A5	DISP2	SYT13	FOXA2	SCGN	SLC7A14	PYY	SSTR5	C2CD4C	GALR1	ST18	PRODH2	TMEM121B	MAPK15
**promonocyte**	RNASE3	SUPT20HL1	RNASE2	OR4M2	SERPINB10	ELANE	PPIAL4A	RXFP2	SETSIP	MS4A10	CTSG	MS4A3	PRTN3	F13A1	MMP8	TIFAB	AZU1	LILRA6	GPR141	DCANP1	CCR2	MNDA	MPO	CLEC5A	CLEC12A	LILRB3	RETN	APOC2	LYZ	ADA2	GAPT	GOLGA8J	FCAR	HIGD2B	TFEC	CEBPE	F10	MEFV	S100A8	PABPC3	NBPF8	S100A12	FCGR3B	KCNJ3	POTEM	PLD4	HNRNPCL1	CSTA	MCEMP1	IGSF6
**prostate gland microvascular endothelial cell**	TMEM252	TGM4	PATE4	C1QTNF9	FAM110D	GDPD3	SELP	GPIHBP1	RBP7	RND1	CD300LG	PLVAP	ECSCR	APOLD1	CRYBB3	AQP1	CDH5	RASIP1	MMRN2	FLT1	DLL4	BCL6B	EMCN	CYYR1	MYCT1	SEMA3G	GPR4	PTPRB	SELE	GALNT15	USHBP1	ARHGEF15	ROBO4	APLNR	ESAM	KDR	ADGRL4	PECAM1	ADAMTS4	LRG1	CH25H	SLC10A6	CLEC14A	UNC45B	SOX18	CCM2L	FMO2	SOX7	CAVIN2	CSF3
**pulmonary interstitial fibroblast**	GPR33	HHIP	GREM2	MUSTN1	SGCG	CLEC9A	KRT82	TARM1	NGB	TBX4	SFTPC	P2RY14	XCR1	ASPN	OOSP1	PRG2	CCL17	PI15	C1QTNF7	WDFY4	CYP8B1	KCNJ8	P4HA3	FOXF2	PNMT	CYP2E1	CCDC42	PLAC9	TBX5	ISM1	DPT	GATA5	PPEF2	FGF18	ADAMTS8	MTARC2	CACNA1S	CDH4	SRMS	RASGRP4	RAB43	KAZALD1	FAM180A	BTLA	PTGIS	CCL19	PRDM6	SCGB1A1	OGN	BMP5
**purkinje cell**	SKOR2	NPSR1	CNTNAP5	KLHL1	IL1RAPL2	OR2L13	CNTNAP4	SIAH3	SHISA6	COL24A1	TMEM132D	GRIK1	TRPC3	INSYN2B	SEPTIN3	ANKRD30BL	FAM237A	LRRC3B	HS6ST3	GABRA6	CA8	KCTD8	SLC1A6	HS3ST2	PRMT8	CDH18	FBN3	GRM7	GALNT17	PDE11A	RAD21L1	EPHA8	TMEM271	PVALEF	SCN2A	CDH12	LINGO2	GRID2	IQCJ	CNTN5	ANO4	SEPTIN5	MAGEC3	CARMIL3	PLD5	LRFN5	ESRRB	GLRA3	ZNF385D	TBL1Y
**pvalb gabaergic cortical interneuron**	SSTR5	LCE5A	PVALB	LCN1	OPN1LW	NXPH2	TCTE1	PDE6H	CDRT15L2	CARTPT	ONECUT1	SLC32A1	MRGPRX2	CHRNA2	PCP2	KCNC2	CORT	ZIM3	MAGEB18	CABP5	SCGB1D4	ANKRD34B	TRPC5	TAF1L	RFPL1	KCNAB3	TAC1	ARR3	GABRD	SPACA5	RCVRN	HAPLN4	AANAT	EIF4E1B	GABRA1	KCNC1	CCDC38	NXPH1	GPR62	MYO3A	TMEM215	OPRD1	DLGAP2	KCNA6	SCN1A	FAM135B	SLCO1B1	CNTNAP4	ANKRD34C	LRRC38
**pyramidal neuron**	KCNJ4	KCNV1	GP1BB	TBR1	SNURF	NEUROD6	LAMP5	KCNJ9	C1QL3	GABRD	GABRA1	KRT12	RNF112	BRINP1	ANKRD63	DDN	SV2B	CAMK2A	SLC30A3	GABRB2	GPR26	SPRN	ARPP21	GABRA4	SLC8A2	RPH3A	ATP2B2	PCSK2	SHISA7	HPCA	HPCAL4	FAM131B	GRM2	SLC1A2	GABRG2	FRRS1L	GRIN2B	GPR61	CACNG2	CACNA1I	RBFOX3	SLC24A2	PLA2G4E	NRGN	KCNF1	KCNB1	CCKBR	SLITRK1	OPALIN	GRIA2
**radial glial cell**	CASP14	WNT8B	TSPY1	GNG14	SEPTIN14	RFX4	PLEKHD1	MOG	OR10Q1	FEZF2	LIPG	NR2E1	HEPN1	NEUROG2	GFAP	BCAN	IRS4	FABP7	DRP2	PMP2	TEX47	SLCO1C1	POU3F2	FOXG1	NCAN	PTPRZ1	SLC35F1	TMEM132B	FAM181A	HES5	DOK5	MEGF10	SOX3	ETFBKMT	GLRA1	SLC6A11	ZNF383	POU3F4	LRRC3B	SALL3	AVP	EMX1	SLC44A5	POU3F3	GRM3	KCNK10	MASP1	ABCA6	NPAS3	MYH6
**raw 264.7**	CLEC4D	GPR84	CLEC5A	CLEC4E	DCSTAMP	CD300LD	CD300LB	SLC6A12	LILRB4	TNIP3	OCSTAMP	FCRL1	RAET1E	CD14	TNFSF9	CCL3	ZAN	NLRP3	MSR1	ALPK2	TMEM26	FXYD2	PTGS2	TNF	TREM2	C5AR1	ZBTB32	IL36G	USP50	IL36A	SLC9A4	DOCK10	IL27	PLA2G4A	DNMT3L	IGSF6	CD36	STAP1	PRKN	CSF1R	MMACHC	MILR1	C3AR1	HTR1B	CD68	CXCL10	TLR7	LPL	DPEP2	ZC3H12C
**rcc10rgb**	SEC14L6	FLG	KRTAP2-3	C1QL1	CD70	GAL3ST1	NDUFA4L2	LCE5A	IGFN1	FIBCD1	RAB42	C1QL4	NXPH4	SYT13	KCNMA1	TRIM55	HNF1B	VSTM1	ELFN2	GPRIN2	IGFBP3	SERPINB7	ZP1	GALNT14	TRIB3	INHBA	SPOCK1	APOL1	TMSB15B	SLC28A1	PAX8	UGT2B7	TGFA	MYEOV	PYDC2	SPAG4	MMP24	EVA1A	LRRC61	IL6	RRAD	TMEM200B	EGLN3	ALPK2	EDN1	LRRC38	LIF	LYPD1	TMEM92	FGF5
**regular atrial cardiac myocyte**	MYBPHL	SBK2	NPPA	MYH6	MYBPC3	LRRC10	BMP10	DHRS7C	CSRP3	MYL7	TNNI3K	TNNT2	TECRL	HRC	SLN	TBX5	ITGB1BP2	MYLK3	POPDC2	YIPF7	MYOZ2	SMYD1	HSPB7	TCAP	CKMT2	TNNI3	NT5C1A	TBX20	XIRP1	LMOD2	MYL1	KCNG2	TXLNB	MYL4	LDB3	FSD2	TNNC1	NPPB	COX6A2	NKX2-5	HJV	OPN4	TRDN	SMPX	CASQ2	UNC45B	MYL3	KCNJ5	TRIM63	FBXL22
**regular ventricular cardiac myocyte**	YIPF7	LRRC10	MYBPC3	MYH6	TNNI3K	DHRS7C	CKMT2	MYL3	ASB11	NT5C1A	MYOZ2	CSRP3	PLN	HRC	MYL2	XIRP1	FSD2	ITGB1BP2	XIRP2	TNNT2	ADPRHL1	SMYD1	ASB10	POPDC2	MYLK3	FITM1	TECRL	TRDN	MYH7	KLHL31	TCAP	CASQ2	TNNI3	LMOD2	HSPB7	ASB14	TXLNB	LRRC14B	HJV	NRAP	MYO18B	COX6A2	MYADML2	SGCG	CKM	LDB3	KCNG2	PPP1R3A	MYOM1	SMPX
**regulatory t cell**	FOXP3	ERFL	CCR8	ZNF831	IL2RA	CTLA4	TIGIT	HSFY1	FCRL3	SIRPG	INSYN2B	CCR4	NIBAN1	SLFN12L	ICOS	OR10A7	INKA2	TNFRSF4	NPAP1L	IL36A	SLAMF1	CPA5	ITK	CD5	LAIR2	ZC3H12D	DAZ4	IL12RB2	GBP5	RTKN2	FAM240C	SKAP1	CD28	CD247	CEACAM21	CALHM4	IKZF2	CD3E	ZNF80	OPRM1	CCR6	IPCEF1	CD6	LTA	GPR174	GPRIN3	FLT3LG	CCR7	P2RY10	TCAF2
**renal alpha-intercalated cell**	SLC4A9	AQP6	ATP6V0D2	CPSF4L	CLNK	ASB15	MALRD1	LRRC31	RTL4	OXGR1	DMRT2	KRTAP5-8	ATP6V0A4	LINGO4	MCCD1	ATP6V1B1	TMEM117	SLC26A7	CHST8	ADRA1A	CLCNKB	SVOPL	SH2D4B	TSHR	WSCD2	BSND	OPRM1	ATP6V1G3	BSPH1	WDR72	CABCOCO1	TMEM213	KCNQ1	PLA2G3	BMPR1B	PLEKHD1	MYO3B	IQCM	CALCR	ATP6V1C2	RGS6	CLCNKA	RHBG	SPINK8	TMEM61	TFCP2L1	MROH5	ARHGEF38	SYTL5	SLC4A1
**renal beta-intercalated cell**	SLC4A9	KRTAP5-8	SLC26A4	RTL4	KRTAP5-9	GPRC6A	ATP6V0D2	SH2D4B	INSRR	MALRD1	TSHR	HMX2	ATP6V0A4	PLA2G3	SVOPL	OXGR1	CPSF4L	PLEKHD1	TMEM117	WDR72	ATP6V1G3	BSPH1	ATP6V1C2	ATP6V1B1	ASB15	KRTAP5-3	SPINK8	OPRM1	TMEM163	RP1	BMP8A	CLNK	SCTR	FOXI1	CLCNKB	TRPC5	WSCD2	MAGEB18	TMEM213	BSND	SLC16A5	KLKB1	ARHGEF38	LSAMP	AFM	TAFA1	NAALADL2	CABCOCO1	IQCM	GLT1D1
**renal intercalated cell**	DEFB125	OR11H4	MCCD1	OR2T35	TRIM49C	OR7A10	OR6P1	OR2F1	AQP6	OR4C3	ATP6V1G3	DAZ2	SLC26A7	TRIM51	NXPH2	KRTAP5-8	MYOCOS	ATP6V0D2	LIMS4	CCDC195	OR14A16	TMEM213	OR9Q1	FOXI1	CLNK	OR2T2	SLC22A24	OR7G2	OR2L3	PTGER3	RHBG	ATP6V1B1	ADGRE3	ZNF705B	GBA3	POTEA	OR7C1	ATP6V0A4	FAM24B	DMRT2	OR3A2	NXF5	DCAF8L2	SLC28A1	OR13A1	RGPD4	SLC4A1	VWA5B1	FYB2	CLDN8
**renal vesicle**	CLEC18B	CLEC18A	CLEC18C	REN	AGTR2	MANSC4	ALX1	HOXD10	HOXD11	HOXD12	TCF21	RIPPLY1	PAX2	RASSF10	HOXA11	LHX1	PENK	WT1	GIMD1	TMSB15A	HOXC10	DACH2	PTTG2	HNRNPCL1	UNCX	HOXA10	HOXD9	EMX2	PAX8	HOXA9	FOXC2	SHISA3	NTS	CALCA	EMID1	C1QTNF12	ANP32D	SCT	LYPD1	FOXL1	HOXC11	FBN3	GPC3	COL9A1	TRIL	NTRK1	CCDC182	COL26A1	SOST	TRPM5
**rerflcai**	KRTAP2-3	FGF5	KRT33B	ZNF185	VSTM1	KISS1	FLG	MOK	KRT34	OXTR	UTS2	ANKRD18B	GBX2	IGFBP1	MARCHF4	CFAP251	LAYN	CXCL5	SLC38A5	IGFBP3	ZNF578	CACNG8	SERPINE1	HMSD	FGF2	RBP7	FGF1	SCN5A	TNFSF4	KIF4A	BLID	ZNF724	SMIM10	G0S2	PLCXD1	PODXL	ZNF732	FBXO27	ATAD3B	NRIP3	FLNC	CCDC80	FAM72C	SMURF2	CGB2	CEP55	KHDC1	MORC4	SPIRE1	ZNF677
**respiratory basal cell**	DEFB131B	STATH	OR52E5	INSYN2A	ATP13A4	TBL1Y	TTC6	KRTAP4-5	TP63	TRPC6	ANOS1	SLC9C1	OVCH2	ANKUB1	ERFL	CHST9	SHOC1	SMIM35	CDH26	CAPN13	SFTPB	SEPTIN9	DUOX1	DUOXA1	EYA2	AOPEP	HRH4	SCGB2B2	NUTM2G	CDRT4	CARD18	WDR49	CYP2B6	CATSPERE	INKA2	GPR39	BMERB1	DAZ4	SCART1	SLC2A9	CTXND2	CLDN20	MTMR8	KIAA1217	NFILZ	AADACL2	NPFFR1	ESR2	CYP2A7	CCDC9B
**respiratory goblet cell**	CYP2A13	VSIG10L2	OR2AJ1	CYP2F1	OR9A4	OR5AC2	CLDN22	OR4K14	OR5H8	PF4V1	OR10S1	MCIDAS	SLURP2	OR56B1	ATP12A	LEUTX	BLACAT1	UGT2A1	KRT39	OR2L5	FUT6	BPIFB2	PPIAL4H	OR13G1	CLDN34	KLK12	SMIM31	GMNC	GLYATL2	CYP2B6	OR7C1	MSMB	KLK13	CYP2J2	STATH	NLRP2B	OR5P3	FAM237B	KRTAP20-4	CYP4Z1	CYP2A6	CYP2C18	MAB21L4	FER1L6	BPIFB1	PGLYRP4	KCNJ18	MUC5AC	SERPINB11	ALKAL1
**resting double-positive thymocyte**	RAG1	CCR9	CD8B	CHRNA9	RAG2	ARPP21	ENDOU	SPO11	CD8A	MIA2	TRAT1	TCF7	SH2D1A	PTCRA	CD3G	LCK	AQP11	KNL1	DNTT	ADAMTS13	WDCP	NOCT	LAT	COLQ	THEMIS	IL2RA	HMGB2	DNAH8	RORC	LEF1	PCLAF	INSL5	SATB1	MKI67	THY1	CD4	SLAMF6	GRIFIN	SLAMF1	HMGB1	ARL5C	INCENP	CCDC62	CD3D	CD28	TOP2A	SKAP1	MFSD4A	MYB	ULBP1
**reticular cell**	LBHD2	TLX1	MFRP	CXCL13	CCL19	GDPD2	C1QTNF5	SERPINA10	C4B	GDF6	MADCAM1	NKX2-3	CCL11	LHX8	HSD3B2	FCAMR	KAZALD1	TCF21	EXOC3L4	GUCY1A1	NTRK1	SLC36A2	MMP27	GABRQ	KCNK4	TNXB	STBD1	CPXM1	PLA2G5	UBD	COL14A1	ADAMTSL2	FOXS1	VCAM1	DES	CLEC3B	GPR88	IL33	SVEP1	MYLK3	SMOC2	KCNJ8	SLAMF8	CCL7	CRYBB3	REM1	BST1	DPT	MSC	HMCN1
**retina horizontal cell**	LCE5A	PRRT1B	FCN2	NMS	GJA10	ONECUT1	KIAA1210	ONECUT3	NAT16	SEPTIN4	FGF19	MINDY4B	SSTR5	TAAR9	NAT8B	LNP1	CACNG3	ZNF593OS	CPHXL	PVALB	CLEC20A	CHRNB3	DRD2	BARHL2	GJA9	CHRNA2	TMSB4Y	KLHL33	PKD2L1	TNR	ZNF648	SLC4A5	PLEKHD1	DRD5	DUXB	TCERG1L	RORB	HMX1	ONECUT2	TFAP2B	PRLH	SCRT2	SMIM28	BEGAIN	FKBP1C	DRD1	KCNJ6	PAK5	SNTG2	OXT
**retinal bipolar neuron**	SMIM36	GABRR3	CABP5	AANAT	CA10	TMEM215	EIF4E1B	ZNF593OS	RDH8	OR2L3	GRM6	CABP2	VSX2	GABRR1	GABRR2	LRTM1	SLC1A7	VSX1	CPLX4	CPHXL	LACTBL1	PCP2	RD3L	OR2T6	GSG1L2	CLEC20A	ZNF804B	TAFA4	CACNA1F	ZNF385B	ASIC4	KCNG4	OR2L5	TMEM196	TRPM1	DUXB	DOK6	SMIM38	OR2M2	GPR179	DRD4	OR5P3	CPLX3	KCNH5	NETO1	TSHB	FAM135B	IGFL4	NEUROD4	DNAH17
**retinal cone cell**	OPN1MW2	OPN1MW3	OPN1LW	GUCA1C	OPN1MW	GRK7	PDE6C	ANKRD33	ARR3	PRLH	RBP3	PSKH2	IMPG1	RP1L1	PDE6H	CPLX4	PDC	IMPG2	GNAT2	NXNL1	SAMD7	RAX2	RTBDN	KCNV2	AIPL1	CNGB3	RCVRN	TULP1	MPP4	FSCN2	RDH8	FABP12	PRPH2	SEPTIN4	GUCA1B	LRIT2	OPN1SW	RS1	GUCA1A	PNMA8C	GUCY2D	PCARE	OR4K13	TAAR9	LITAFD	RAB41	LRIT1	ANTXRL	GNB3	SCGB1D4
**retinal ganglion cell**	CHRNB3	POU4F2	CHRNA6	AKAIN1	ZNF593OS	RFPL1	SLC17A6	IRX6	TFAP2D	SCRT1	POU4F1	PHF24	NEFL	SCGB1D4	TRARG1	CENPVL2	PVALB	HTR1B	CARTPT	NEFM	SYT2	OR6C75	GPR26	DIRAS1	IQSEC3	TAAR9	SNCB	OR10K1	OR10H1	IGLON5	POU4F3	OR52N1	LGI3	CEND1	VAX2	ZNF648	TMEM74B	RPH3A	CLVS2	SCN1A	ZDHHC19	PCP2	TTC9B	NAT8B	GPR12	SPINK9	IFNL1	SULT4A1	CALB2	OR1A1
**retinal pigment epithelial cell**	RPE65	AQP12B	CNGB3	SLC16A8	CNBD1	PRSS33	NPFFR1	CST5	PXDNL	TYR	RGR	SLC6A20	MFRP	SLC39A12	TRPM1	RLBP1	RRH	BCO1	SLC38A11	TYRP1	PLD5	LRIT3	OR51E2	DCT	SLC38A8	GSX2	CNDP1	FYB2	TMEM235	CPHXL	TRPM3	PMEL	ABCA4	HTR3D	KCNH5	NCCRP1	FSBP	C1QTNF5	MYRIP	CA14	TSPAN10	OTX2	SLC4A5	BTNL3	PKD1L2	LRAT	TRIM49D2	HORMAD2	SLC22A8	MROH7
**retinal progenitor cell**	VSX2	NEUROG2	RORB	CRB1	RAX	PTH2	SIX6	FOXN4	SIX3	PRDM13	NXPH2	MAB21L1	PNMA8C	FEZF2	PAX6	RFPL1	NEUROD4	SERTM2	ADAMTS18	NR2E1	VAX2	POU4F2	TFAP2D	GFY	HMX1	DIPK1C	RCVRN	FOXI3	RRH	CHRNA3	MGARP	MAB21L2	CARTPT	LCN1	PDC	SAPCD1	ATOH7	RLBP1	NHLH2	MSTN	CDH7	CLVS1	INSM2	DAPL1	SORCS1	PRSS35	OTX2	HES5	COL9A1	ZNF536
**retinal rod cell**	RDH8	PDC	OR5P3	NR2E3	SAMD7	IMPG1	RHO	CLUL1	RBP3	ANKRD33	GNAT1	KCNV2	PDE6G	RCVRN	AIPL1	CNGA1	NRL	PPEF2	NXNL1	CPLX4	RTBDN	RP1L1	PRPH2	TULP1	HTR6	SAG	RAX2	PDE6A	IMPG2	SMIM36	DEFB119	RP1	PCARE	CABP4	TMEM244	FSCN2	CNGB1	GUCA1B	OR5P2	CABP5	RDH12	AANAT	EIF4E1B	GUCA1A	FABP12	CRX	DRD4	ABCA4	ELSPBP1	PDE6B
**rko**	FUT5	GJA9	SUN3	PRICKLE4	LCE1F	CSNK2A3	TM4SF19	FBXO10	LCE1C	LCE1B	CRYBB2	SEC31B	ZNF474	LCE1E	TREX2	MAP1LC3B2	KRTAP4-9	ZNF556	SLCO1B3	CCDC142	KIF4B	TNFRSF10D	ZNF23	CCNE2	HOXA9	MT1M	ANKRD23	CTAGE15	PTTG2	UBE2NL	CTAGE9	DGKI	PIP5KL1	HMSD	ULBP2	BCL2A1	RRM2B	UTS2	ESCO2	E2F7	PABPC3	CHMP4C	SRFBP1	NLRP13	ZNF28	PAGR1	SERPINE1	DNAAF3	MT1E	KIFBP
**rod bipolar cell**	SEBOX	BHLHE23	RD3L	GPR179	PCP2	GABRR1	CPLX3	CABP5	GRM6	GABRR2	LRIT1	LRIT2	NRN1L	VSX2	GNG13	TRPM1	LRTM1	LACTBL1	KCNE2	NEUROD4	PRPH2	PLK5	RHO	VSTM2B	TMEM215	GNB3	IL22	NYX	MGARP	GNAT1	CCDC177	RD3	ASIC3	B3GALT2	WSCD2	SAG	NTNG1	RSPH10B	GLRA1	SLC5A8	SLC1A7	ASIC4	OPN1MW	KCNJ9	PDE6G	SNCB	KCNA5	ADRB1	NDNF	TRNP1
**rohon-beard neuron**	DRGX	OR6X1	PRDM14	NEUROG1	SKOR2	IL17REL	SLC18A3	BSX	TAS2R8	PRPH	NHLH1	KCNK18	OR52M1	FERD3L	OR6A2	AVP	NAT16	CYP26C1	RGS9BP	MYT1	ZNF735	ADCYAP1	FAM47A	TAS2R13	GPA33	LGALS9C	STX1B	FAM240B	FOXD4L1	PPP1R17	P2RY4	CRYBA1	ADGRD2	DPYSL4	SLC2A7	TAS2R19	ETV3L	HIGD1C	AKAIN1	FTMT	NEUROG2	CLEC4M	ISL2	SPRR5	OR13C8	TAS2R10	KIAA0408	GAREM2	NPY2R	TAS2R7
**rt4**	CST1	BTBD16	PLA2G2F	TRIM31	KRTAP5-10	IVL	TRPV6	SPOCD1	GJB6	ZNF488	ANXA10	IGFL1	FABP4	UPK2	NIPAL4	SPTSSB	OR5P2	SNX31	EPHB6	VGLL1	CLCA4	CDH26	RHBG	SLC14A1	HOXA13	SPRR3	VSIG2	CST4	UPK1B	BCAS1	TMPRSS4	ZNF320	UPK3B	SNCG	SYT10	AKR1C2	CAPN8	NT5DC4	ATP1A4	PROM2	RASSF6	S100A2	S100P	KRTAP5-9	HAS3	SLC16A5	TMEM238L	HPGD	VIPR1	ADIRF
**s-shaped body**	CLEC18B	CLEC18C	RFPL1	GLYATL1	MCCD1	NPHS2	SOST	REN	AADACL4	UGT3A1	CLEC18A	IRX6	RIPPLY1	SCGB1C2	RASSF10	FRMD1	HNRNPCL3	OLFM3	MST1	KIF12	EMX2	DCDC2	AGXT2	SMIM24	CST5	LAMB4	NOX3	SLC3A1	CA4	CLDN19	PAX2	KRTAP4-11	PNMA6A	HNF1B	SULT1C4	PDZK1	C1QTNF3	NPHS1	LHX1	ADAMTS16	TMEM266	SLC13A1	HOXA9	ZSCAN1	CRB2	TCF21	RFPL2	SLC5A8	MMP23B	PAX8
**saccharomyces cerevisiae**	PRY2	TAAR1	PGA3	HTN3	GSC2	ABCC11	CCL1	CLCN1	GSX1	PTH2	ADH6	PRB1	MAS1	MCHR1	PROKR1	AQP9	PMP2	CFAP91	RHOXF2	DDI1	FPR3	ADH4	GPHA2	ADH1A	PSG1	APOBEC3A	CIB3	RAX2	AIRE	MSS51	ADH1C	TRIM21	ORM2	CWH43	RO60	CEBPE	CCR4	VCX	DNAI1	SIM1	AOC1	TAL1	SIT1	FPR1	PSD2	GPD1	SLC9B1	HTD2	OCA2	GPT
**scaber**	FAM83C	KRT31	KRT6A	NAA11	FAM83A	CCN6	CALML3	SPRR1B	OR51B4	CFAP251	MYEOV	GJB5	GJB3	CST6	TP53AIP1	GAL	PLCXD2	RASIP1	CARD18	MAGEA10	NMB	S100A2	RHOD	FST	SOX7	CSAG1	CDKN2A	COL17A1	MROH6	TESMIN	SLC38A5	LY6K	SFN	MAGEA12	HBE1	CYP4F11	TRIM7	FOSL1	FERMT1	KHDC1L	MAGEA3	DMKN	GPR87	PRSS21	NMU	LAMC2	PLEK2	MT1E	PTHLH	LAMB3
**scc**	CLDN17	MMP3	PRKG2	EPGN	CRYAA	MMP13	SERPINB10	LCE3C	BRINP3	MMP10	LRRC15	TCIM	CCL7	PRR9	CCN5	BDKRB1	LIPK	ANXA8	PADI3	SPRR2G	LSMEM1	CASP12	LOXL3	KRTDAP	PLA2G7	KRTAP4-16	THBS2	FBLN2	TNFSF11	GDNF	GREM1	PI15	TNN	HCAR2	PGLYRP3	CXCL1	GJB4	ASPN	ADAMTS2	PTGS2	POSTN	C1QTNF12	FNDC1	MSLN	FMO2	LOX	PKP1	BNC1	DUOXA1	ANK1
**scc25**	PTTG2	TP53AIP1	CCL26	MMP13	CALML3	ULBP2	ANXA8L1	IL20RB	KRT17	CXCL11	KRT6A	CASP14	HNRNPCL1	SAA1	KLK5	GREM1	KRT14	KRT13	KCNK9	KRT5	CSTA	LY6D	ANP32D	SERPINB13	LRRC69	IFNL3	KCNJ3	IL1F10	TP63	GPR87	IL1B	IL1A	S100A2	MT1HL1	FGFBP1	KRT16	SERPINB3	HES2	EIF5AL1	KRT31	MMP28	AIM2	CCDC152	SOX15	SERPINB4	HCAR3	FGF23	ANXA10	NEUROD2	RAET1G
**schmidtea mediterranea**	KRTAP6-1	CBLIF	GPR33	KRT32	BMP10	FSHR	DRD3	IFNB1	IFNA5	F13B	DRD5	IFNA4	CRYGA	KRT84	FOXL1	DRD2	ENAM	GJA8	KRTAP9-1	ADAM2	FGF6	CYP7A1	RPTN	CYP1A1	CAV3	SLC6A3	CRHR2	GALR1	HSD17B3	INHBE	GIP	ADAM7	IL10	KERA	IL4	NKX3-2	HMX3	GCGR	OPN1SW	HTR5A	KCNS2	HES2	TPSG1	LIPC	GDF10	GNRHR	CST9	HMX2	FOXN1	DLX3
**schwann cell**	MPZ	CDH19	PRIMA1	CLDN19	GOLGA8F	FOXD3	PPIAL4C	SMCO3	KLK9	SOX10	CYP21A2	KCNA1	PRSS56	OLFML2A	NCMAP	COL20A1	PRX	PLP1	DHH	LGI4	INS	CCN3	PHOX2B	CYP17A1	GFRA3	GASK1B	RASGEF1C	TIMD4	TMPRSS5	SFRP5	CTNNA3	SHC4	COL28A1	CYP11B2	SCN7A	CMTM5	SLC36A2	TSPAN11	MAG	KIAA1755	ART3	SORCS1	MLIP	FAM237A	ASPA	ABCA8	NUTM2F	GJC3	SPATA31C2	DRP2
**schwann cell precursor**	CDH19	MEGF10	DCC	CDH6	PAX3	PRDM12	IL1RAPL1	GRID2	GRIK2	SORCS1	GRIP1	SLC35F1	COL25A1	SNTB1	NDST3	TMEM132E	PDZRN3	FIGN	STARD13	B3GALT1	GPC6	PDE7B	LUZP2	CDH10	TENM3	TSPAN18	PARD3B	GFRA1	FBXL7	NXPE4	DACH2	SOX5	FOXP2	SCUBE1	ARHGAP28	PLXNA4	COL23A1	PKNOX2	IGDCC3	NPAS3	SPON1	DBX1	EDNRB	CTNND2	NKAIN2	TMEM132C	PRIMA1	KCNQ5	ADAM12	TMTC2
**sebaceous gland cell**	KRT71	KRT79	KRT35	KRT28	BPIFC	KRT25	PSAPL1	LGALS7	KRTAP11-1	KRTAP26-1	KRTAP8-1	SBSN	KRT75	BARX2	PINLYP	KLK13	LY6G6C	FAM25C	KRT27	TMPRSS7	PRR9	KLK7	KRT17	DSC3	KRT14	RHBG	SPRR1A	KRT73	KRT77	SOSTDC1	CAPNS2	RNASE12	PI15	HOXC13	LYPD3	KRTAP19-3	NLRP10	KRT5	KRTAP22-2	LHFPL1	PKP1	GJB2	SERPINB5	TRIM29	CALML3	KRT33A	AWAT1	ADGRF4	SLC39A2	GJB4
**secretory cell**	LACRT	OR4E2	DCD	OR4K13	SPATA31A7	CYP2F1	SCGB2A2	OR10R2	CYP2A13	GSTA5	SERPINB3	CA6	BPIFB1	MSMB	VMO1	CXCL17	OR7G2	SLC6A14	OR10H5	MUC21	MUC5AC	KCNJ18	LYPD2	OR2V2	SCGB1A1	CYP2B6	MUC16	RHOXF2B	SCGB1D2	PRB4	OR2J3	ATP12A	CRISP3	OR52K1	TMPRSS11B	OR7D4	OR5AU1	FAM3D	OR2J2	OR2T11	GABRP	OR6C6	SERPINB4	AQP5	BPIFA1	SPRR2A	PLAAT2	ADGRF1	OR51D1	MUC22
**sensory hair cell**	KRTAP19-5	KNCN	GRXCR2	KCNA10	CLRN2	PLSCR5	NPY4R	GRXCR1	POU4F3	GPR152	CHRM4	LCE6A	LRRC3C	CHRNA9	CHRNA10	MAGEA2	GPX5	FSCN2	DEFA5	SRMS	SEPTIN5	OCM	NEU2	DSC1	TECTB	OTOF	SPRR2B	STRC	SLC26A5	CLRN1	LHCGR	TAL2	GPR156	MEF2B	CNTF	SLC22A12	NMUR1	TMC1	TBX5	SDR42E2	SLC18A3	LRRC52	DYTN	CST9	TBX6	LHFPL5	LOXHD1	DEFA3	GRK1	LRTM2
**sensory neuron**	PIRT	SCN10A	CALCB	CALCA	SCRT2	CARTPT	TLX2	P2RX3	TRPV1	UTS2B	MRGPRD	NTRK1	ONECUT1	THSD7B	TRARG1	CHRNA6	PRPH	CHRNB3	POU4F1	DRGX	BCL2L10	PSMA8	GAPDHS	SLC17A6	CHRNB4	HTR3A	PRDM12	PPP1R1C	MYMK	NEUROG1	FXYD7	KCNMB2	CHRNA3	NEUROD1	BHMT	TRAPPC3L	PPEF1	GPR151	PHF24	EBF2	MSANTD1	VSTM5	TCAP	ISL2	TECTB	TMEM178B	HTR3B	HS3ST5	XKR7	RIMKLA
**sensory neuron of dorsal root ganglion**	MRGPRD	SCN10A	KCNK18	GPR151	PIRT	CSNK1A1L	KRTAP27-1	PRDM12	OR4D6	P2RX3	OR5M1	BHLHA9	OR5W2	MRGPRX1	ISL2	SCN11A	PPEF1	POU4F1	INSM2	CALCB	TRARG1	A3GALT2	CHRNA6	OR8I2	OR5B21	TRAPPC3L	VSTM5	SLC17A6	PRPH	CTXN3	POTEM	OR4C6	AVIL	KRTAP4-3	NTRK1	KRT27	OR2V1	F2RL2	TMEM233	BEAN1	KRTAP4-6	KRTAP9-9	TAFA4	LIX1	OR1J2	AMIGO3	RET	POU4F3	VWA7	JPH3
**serotonergic neuron**	TPH2	MINAR2	HCRTR1	HTR1A	SLC10A4	RNF227	SLC6A4	KCNK9	CASTOR2	ZDHHC22	PHF24	GABRQ	VWA5B1	SLC18A2	TMEM255A	NPFFR1	TMEM114	FEV	OPRL1	CBLN4	ARHGAP36	CELF6	SCN2B	MINAR1	TMEM179	SLC17A8	TMEM250	DDC	ZCCHC12	MRAP2	SLC5A5	LHFPL5	HRH3	FAM43B	IRS4	HTR1B	DGKK	BEGAIN	RASGRF1	MAGEE2	GLRA2	TMEM130	RIT2	TRPC7	WFDC12	PDYN	SYNGR3	JPH3	NECAB2	CBLN2
**serous cell of epithelium of bronchus**	HSFX3	MAGEB5	SMR3B	PRB4	MUC7	IL19	SMR3A	PRB3	LPO	STATH	SPDYE18	ITPRID1	TEX54	CRISP3	PRB2	PIP	CES3	SLC13A2	TCN1	OXGR1	PRH2	PRB1	AKR7L	ODAM	SLC5A1	TMEM210	C2CD4A	PRSS41	ROPN1	VSIG10L2	CCDC192	GLYATL2	ZG16B	LRRC26	POU5F1B	PRPS1L1	AZGP1	GJC3	FBN3	SOX10	TLX1	SLC13A5	HSD17B2	LTF	ZNF491	CALML5	OVCH1	NPY5R	FUT6	SLCO1A2
**serous secreting cell**	PRH2	PRB3	PRB1	PIP	PRB4	PRB2	BPIFB1	MUC7	SMR3A	SCGB3A2	SFTPA2	CRISP3	SCGB3A1	LPO	SERPINA3	DMBT1	SFTPA1	ZG16B	ODAM	TCN1	PRH1	LTF	CXCL17	PRR4	AZGP1	BPIFA1	LRRC26	FAM3D	CYP4X1	FDCSP	SCGB1A1	LYZ	AQP5	OR2A4	SFTPB	PIGR	SLCO1A2	SLPI	RNASE1	STATH	SLC13A2	ELF5	MSMB	ITIH6	SFTPD	BPIFB2	CCL28	FOLR1	ELAPOR1	PPP1R1B
**sertoli cell**	FATE1	DEFB123	CST9	TRPC5OS	CST9L	CLEC12B	KIAA1210	KRTAP21-2	TEX55	SPATA31D4	WFDC6	SLC35G6	DEFB119	SPATA31A1	SPDYE4	OR8B2	HSD17B3	DCAF8L1	GOLGA6D	OR8S1	OR8B3	CT47A6	EBLN1	BIRC8	SPATA46	IQCA1L	TGIF2LY	SPEM3	CCDC185	CSTL1	SPATA31D3	GAB4	SPATA31E1	OR10J1	OR6F1	AMH	PRND	NUTM2B	TNP2	GLT6D1	FAM24A	TEX28	NXF2B	KRTAP5-7	OR4M1	OR2H1	MPC1L	FAM47B	SSX2B	CFAP97D1
**set2**	SIGLEC12	TPSD1	OR2W3	CA1	FCER1A	KLF1	GATA1	GYPE	PGAM4	OR2A4	ITLN2	GCSAML	ITGA2B	TPSAB1	PRSS57	HBA1	TBC1D3H	MS4A3	CYTL1	NAT8B	TPSB2	HDC	TBC1D3B	NFE2	LCN6	SLC10A4	IL9R	PIK3R6	AMHR2	RHCE	GTSF1	GYPB	DPEP3	ICAM4	CETP	NTRK1	GP1BA	SIGLEC6	CMTM5	VN1R1	PNMT	SFRP5	H3-5	FAM124B	ZNF560	MFNG	ITLN1	NCF4	PPIAL4G	TMEM35B
**sh10tc**	SSX1	VCX	VCX3B	PASD1	CDH15	PLCZ1	FAM9C	LCN1	MAGEA10	C1QL4	MAGEA12	ZNF280A	MAGEA4	MAGEA3	CSAG1	CACNA2D4	PAGE5	POTEG	PAEP	CRYBB2	MAGEC2	CALHM3	KIAA1549L	LY6K	VSTM1	C4BPB	KCNG1	MARCHF4	PADI1	VCX2	HOXD11	CCL26	MAGEA6	FIBCD1	TEAD4	SPOCD1	PAGE2	COX7B2	ZNF703	LRRC61	ADAMTS12	BDNF	GALNT9	CREB3L1	ZFP57	BLID	CACNG8	TNNT1	ULBP2	CIBAR1
**short term hematopoietic stem cell**	DCDC2C	MPL	GPR25	GJE1	TNIP3	MC5R	DCSTAMP	TREML2	MYL10	GBP6	PADI4	GPR84	F2RL3	ITGB3	PDCD1LG2	ADGRL4	NLRP10	SLC18A2	IRGM	AKR1E2	FLT3	TFR2	GRK4	TRAF1	PEAR1	CHAD	PGLYRP2	NEURL3	WDFY4	TRPC6	EBI3	CX3CL1	KIT	GBP7	NKX2-3	SOAT2	AMELX	GJC3	NWD1	TAS1R1	MUC13	MYCT1	ADGRG1	TYR	IL12A	P2RY4	RSAD2	CXCL10	ADGRG7	MMRN1
**skcm**	OR2B3	OR5B17	OR4C12	OR10S1	OR6N2	OR1K1	OR4K2	OR8H3	OR10V1	OR5H6	OR52A5	OR6P1	OR5H2	OR6C3	OR8H2	OR52B6	VN1R4	OR4B1	OR13C4	OR10K2	OR13C9	OR14C36	OR4D10	OR52E2	OR51Q1	OR51B6	PLA2G2E	OR6B3	OR1L1	OR1J4	OR6C76	OR1L4	OR2Z1	OR10P1	OR4F6	IFNA14	OR12D3	OR5M8	VCY1B	OR4S2	DAZ4	OR4K1	OR4C13	OR5M9	OR56B4	TP53TG3C	OR1D5	OR4F5	OR8D1	OR5H1
**skeletal muscle satellite cell**	MYF5	ASB5	CHRDL2	PAX7	MYOD1	CHODL	CDH15	MYMK	MUSK	ERFE	NTN5	MYMX	CHRNA1	VGLL2	CALCR	SLC10A6	LBX1	NPPC	NEU2	RAPSN	BDKRB1	CRLF1	STYXL2	DRP2	PITX3	MYOG	DES	TACR3	C1QTNF3	MEGF10	FGFR4	EDN3	DPT	SGCA	JSRP1	MSTN	HTRA3	CHRND	EN1	DBX1	SBK3	FITM1	RTL3	RHOJ	OLFML2A	OLFML2B	DYNAP	HEYL	FAM240C	TAC4
**skeletal muscle satellite stem cell**	OR10AC1	LCE3A	MYOD1	MYF5	CHRDL2	ASB5	MUSK	SCYGR4	FAM240A	OR9A2	OR13G1	OR5K1	FAM237B	PAX7	CHODL	CDH15	KRTAP10-9	SLC10A6	OR4M2	BLID	IFNA8	CALCR	CHRNA1	OR6F1	PITX3	OR4D2	TACR3	RAPSN	TBC1D3D	VGLL2	SCYGR10	ATP6V1FNB	FOXD4L5	OR52A1	BDKRB1	ULBP3	EEF1AKMT4	OR2T34	OPN5	DPT	DPEP2NB	TRIM72	DRP2	EDN3	MEGF10	ZNF878	NLRP2B	IFNA6	OR10AD1	SGCA
**skin fibroblast**	UPK3BL2	KRTAP1-5	POTEJ	OR2T7	PSG5	KRTAP4-9	H4C14	MAGED4	SMIM43	OR6K3	OR7D4	THSD8	LRRC15	KRTAP4-12	TWIST2	OR5A2	OR5AN1	H2AC14	TBX15	CASTOR2	MMP3	TMDD1	MAGED4B	WARS1	OR2AT4	OR14J1	OR8B8	OR7G2	OR4K17	OR4K13	LITAFD	OR5AS1	H2AC17	GOLGA6B	OR2M3	SPDYE11	OR6C4	SCYGR4	CCN4	OR6Y1	GREM2	CTAGE6	SPDYE17	OR10G4	OR52K1	GREM1	H3C3	PSG4	OR1D2	CCN3
**skmel5**	LCE2A	BIRC7	MLANA	PMEL	DCT	TYRP1	CAPN3	PRDM7	DEFB136	WFDC1	GPR143	MAG	DNASE2B	KRTAP19-1	ABCB5	S100B	ANKRD30B	MFSD12	KRT27	TRPM1	LYPD1	OR51B4	MELTF	VGF	ROPN1B	SLC16A6	UCN2	TRPM8	HTN1	EN2	MMP17	ZNF280A	QPCT	LZTS1	CLDN14	NES	MAGEA12	BAIAP2L2	CSAG1	TRIM63	MIA	PRAME	HRK	GOLGA7B	PAEP	ZNF697	RAB38	GJB1	CYGB	PAX3
**skmes1**	TMEM244	PRDM13	LHX1	TRIML2	PRSS3	RNF212	FGF5	IL13RA2	STEAP1B	TMEM92	MELTF	MAGEA3	APCDD1L	FOXL2	MAGEA1	IL11	NPW	FIBCD1	DMRT1	SPANXC	OXTR	MSC	EREG	HS3ST3A1	GABRQ	POPDC3	OR51B4	CXCL5	EPHB6	CSAG1	G0S2	PTHLH	CALB2	TMEM156	TNIP3	MAGEB2	IFNE	PLEK2	SPOCD1	RGS20	TRIM16L	MAGEA6	DGKI	CGB1	LYPD1	ULBP2	AHNAK2	CFAP251	PLAAT1	NPB
**sknas**	TLX2	VIP	PHOX2A	LCN1	PRRT4	PRCD	CHRNA3	MAGEA11	HGF	COLEC11	PRSS12	RGS5	LHX8	S100A3	IL13RA2	SYNPO2	TMEM229A	RET	CRYGC	PTGIR	VGF	ATOH8	SAMD11	NTRK1	DBH	NPY	HOXD8	PRSS3	PRPH	GAL	CRABP2	HTR2B	IL11RA	DOK4	TYRP1	GNG8	GCG	PRAME	SHD	RASL10B	ZNF804A	FAM89A	ASS1	ZNF467	GATA4	MAB21L1	ZNF853	HMCN2	CCDC74B	HOXD9
**slamf1-negative multipotent progenitor cell**	TREML2	MPL	MC5R	MYL10	PRTN3	NFE2	F2RL3	FUT7	FLT3	MPO	OTOS	TSPAN32	PGLYRP2	SH2D5	IL12A	GPR171	PADI4	EBI3	RAB38	KCNK12	SPNS3	BPIFC	LMO2	CD34	MUC13	NKPD1	CD27	DNTT	RIPK3	SLC22A3	TFR2	ANGPT1	NKX2-3	RGS18	DKKL1	DAPP1	HK3	LYL1	IL21R	ITPRIPL1	NAPSA	GFI1	KCNN4	TAS1R1	CD93	GIMAP1	UBASH3A	KLK8	SPNS2	PTPRCAP
**slamf1-positive multipotent progenitor cell**	MPO	MPL	TREML2	FUT7	PRTN3	FLT3	F2RL3	BPIFC	MC5R	HK3	OTOS	NFE2	CTSG	TICAM2	SLC22A3	SH2D5	PADI4	SPNS3	RIPK3	MUC13	TAS1R1	KCNK12	CD34	TSPAN32	PGLYRP2	TFR2	NKPD1	DNTT	GPR171	PRSS57	FFAR2	ANGPT1	MBNL3	UBASH3A	ULBP1	RAB38	DAPP1	IL12A	UHRF1	RGS18	SIGLEC5	LMO2	TESPA1	PARVG	NAPSA	DKKL1	CSF3R	EBI3	RAB44	GP5
**slow muscle cell**	STRIT1	IDI2	PPDPFL	SMTNL1	MYLK2	KLHL40	LMOD2	HJV	KLHL34	MYOT	ANKRD2	CACNG1	UCP3	MYOZ2	NRAP	ASB10	AMPD1	VGLL2	PPP1R27	MYF6	PGPEP1L	DUSP13	MYL2	HSPB3	PRKAG3	LRRC38	PERM1	CA3	MYH7	MYOZ1	DUSP29	TRIM54	YIPF7	ABRA	CHRND	SMCO1	MRLN	XIRP2	IP6K3	ASB12	MYPN	XIRP1	PPP1R3A	CACNA1S	MYBPC1	MYOG	DHRS7C	CASQ1	KLHL30	LRRC39
**small luteal cell**	CYP17A1	HSD3B1	INSL3	CYP11A1	LHCGR	HAO2	STAR	INHA	CST9	MGARP	SCT	MRAP	NPPC	SERPINA5	ALDH1A1	PRLR	CYP11B1	CAPN9	CPA1	HMGCS2	AMHR2	GSTM2	WNT6	CLPS	OSR2	FOLR1	SLC45A3	GSTA2	WFDC1	MMD2	PLA2G1B	NTS	KCTD14	FDX1	AEBP1	VSNL1	SFTPC	GSTM1	NPY	AGT	GRB14	CTRL	C4B	ETNK2	KCNK3	GADD45G	SMPX	SFRP4	SMOC2	AOC3
**smooth muscle**	KRTAP8-1	IL12B	IFNA7	IFNA5	OPN4	DEFB136	SRY	IFNA4	USH1G	KRTAP12-1	HIGD1B	TACR3	IFNB1	CHRNA7	TINAG	IL9	RGS5	KRTAP9-1	TRHR	RERGL	CASQ2	FUT4	GJA4	BTN1A1	MYH11	MLNR	ADAMTS4	GPR20	KCNA5	AOC3	COLEC11	GPX5	GUCY2D	COX4I2	MFRP	NRIP2	NKX2-2	HTR5A	PLN	C1QTNF9	LMOD1	KCNK17	GUCY1B1	AVPR1A	FHL5	NDUFA4L2	CST9	EPX	GLP2R	RASL12
**smooth muscle cell**	RERGL	IL9	MYH11	HIGD1B	GPR20	LMOD1	PLN	AOC3	OR51E1	RASL12	MYOCD	NRIP2	MUSTN1	CASQ2	OR10AC1	SGCA	FAM162B	GJA4	SOST	DAZ3	SUSD5	COX4I2	IRAG1	FOXS1	RGS5	C1QTNF8	KCNA5	KCNMB1	SYNPO2	CNN1	ITIH4	FHL5	ITGA7	MRGPRF	WNT8B	CCDC3	JPH2	ITGA8	PLA2G5	MDFIC2	ODF3L1	HEYL	AVPR1A	HRC	GRIP2	NOTCH3	WFDC1	NDUFA4L2	MCAM	XIRP1
**smooth muscle cell of prostate**	MT1A	RERGL	PATE4	KCNE4	PI15	FAM153B	MT1HL1	IL1RL1	RGS5	TGM4	AVPR1A	LMOD1	CRISPLD2	MYH11	SYNPO2	MT1M	PLN	MUSTN1	STUM	GJA4	TAGLN	MSMB	CNN1	HNRNPCL3	KLK3	NRIP2	NDUFA4L2	ANGPT4	ADRA2A	WFDC1	HOXA13	SYNM	KCNA5	STEAP4	RASL12	HIGD1B	GPR20	CXCL8	IRAG1	EDNRB	NPTX2	FBXL22	ADAMTS4	HSPA6	SSTR2	MYL9	KRTAP4-12	CCL19	SOD3	HAPLN2
**smooth muscle cell of trachea**	PTH	HIGD1B	MUSTN1	CHRDL2	NPS	IL6	NRIP2	CCL11	ADAMTS8	ADRA1D	BPIFA1	MYH11	AOC3	EPHX3	MYF5	SLC10A6	CYTL1	TNMD	PLAC9	ADAMTS4	ASPN	IRAG1	ERFE	SUSD5	GPHB5	ASB5	DES	COX4I2	CASQ2	XIRP1	KPRP	CHAD	GPR20	KCNE4	GJA4	TRARG1	CHODL	RASL11A	RRAD	ITGA7	NGF	MUSK	RHOJ	LMOD1	CCL19	ALKAL2	NPY1R	IRGM	NOTCH3	RBMY1A1
**sncg gabaergic cortical interneuron**	KRT73	HTR3A	CNR1	VIP	NPY2R	NPAS1	VSTM2A	ADARB2	TNFAIP8L3	GPR12	KCNIP1	SLC35F4	CCK	ADGRA1	PNOC	SYNPR	NPY5R	NETO1	NECAB1	SMIM18	CPNE6	COL19A1	CTXN2	ZCCHC12	ZDHHC22	ST8SIA5	RGS8	TRIM17	FRMD7	ELMOD1	NECAB2	ATP6V1G2	KCNS2	CAMK1G	RPH3A	GAD1	CACNG3	HTR2C	HTR6	CELF6	SORCS3	FAM163B	GAD2	SERPINA12	NPY	NAP1L2	GPR61	GALNTL6	SMCO3	CNTN6
**snu61**	IGFL2	PHGR1	TMEM238L	CCNI2	ASCL2	ABHD12B	CDX1	MT1G	KLK7	ST6GALNAC1	FFAR4	NMU	LGALS4	PAGE1	PYDC2	SLCO1B3	ONECUT3	AKR7A3	CDX2	PRSS3	RNF43	MUC5AC	KLK10	PAGE2B	SPINK4	PPP1R1B	FUT3	HOXA13	BTBD16	NOXO1	BMP4	GALNT5	TRIM15	PDE4C	IL20RA	VIL1	GPX2	TNS4	FTHL17	TRABD2A	MISP	CCNP	IL37	SLC9A2	AGR3	MNX1	GPR35	GAL	AGR2	TMEM52
**snu213**	KRTAP4-1	CALHM3	OR4C6	CD163L1	KLK6	RNF223	SHC4	KLK7	NT5DC4	CALB2	NOX5	KRT20	MALL	RNF224	SH2D3A	CST6	KLK10	ESPN	THAP10	STEAP1	FAM111B	ZBED2	NOXO1	IGFL4	RIBC2	OR51B4	GPR87	DKK1	MET	LY6K	MROH6	KRT34	B3GNT3	HBE1	MUC4	HOXA6	HOXB8	BIK	MMP7	ARL14	KRTAP3-1	BLID	GJB3	FOSL1	ANKRD22	PRSS3	ST14	POPDC3	ADIRF	PRSS22
**snu398**	TMPRSS15	HGF	GCG	NTS	DKK2	NEURL1	NCR3LG1	DLX6	CCDC160	TTPA	TFPI2	DLX2	CHODL	SP9	DLX5	ZIC1	WNT16	SP5	NEFH	HOXD11	HMX1	DLX1	SKIDA1	SLC30A8	TLX1	HOXD13	VAX1	ANKRD18B	HOXD8	HOXD9	HEY2	SYPL2	HOXB5	NPFFR2	RBM20	ADRA2C	PRAME	FAM171B	KIF1A	RIMS4	FAM9C	CRISPLD1	PEG10	ATP6V1E2	PCDH9	CFAP92	HOXA6	MAFA	EPHA7	ZNF280A
**snu423**	KRTAP4-8	SPANXC	KISS1	PSG1	KRTAP2-3	PSG9	TRIM55	SIGLEC15	IGFN1	OR2T8	RTL3	ANKRD1	CPA4	KRT34	GDNF	CGB2	SPANXD	SPANXA2	TRIM58	IGFBP1	KRT81	HCN4	FGF1	ZNF716	PSG5	COX7B2	PSG4	GREM1	CNN1	STC2	ARSI	IL11	SLC16A2	MYEOV	FAM133A	SERPINA9	SFRP1	ADAMTS6	FOXL1	KIF4A	PSG6	STC1	SERPINE1	KCNMA1	FGF5	MOK	NRIP3	BDNF	LIF	TMEM158
**snu449**	OXTR	FGF5	TM4SF18	RNF182	MAGEA1	CNTNAP3B	PADI3	TRIM55	SYT15	ZNF488	CNTNAP3	LHX1	CYP24A1	IAPP	SULT1C4	PXDNL	BMP5	LYPD1	NIPAL4	HAS3	DIO3	MARCHF4	CITED4	ZNF724	KCNMA1	EDN1	WNT7B	C1QL1	BATF3	AHNAK2	MSMP	ANKRD18B	COL13A1	STC1	FGB	NPTX1	RGS7	ZBED2	MORC4	HNF1B	SLC27A5	IRS1	STC2	TRNP1	FJX1	FZD2	PADI2	ANKRD30B	ZNF732	ASB9
**snu738**	SSX1	XAGE3	C4BPB	ZNF479	IGFL3	VSTM1	BARX1	MAGEA4	MAGEA10	ZNF578	MAGEA12	CSAG1	FOXD1	CLEC2L	MAGEA6	TEX29	PRDM13	MEIOB	TMEM200B	MARCHF4	MAGEA3	RFLNA	CDCA2	TXNRD2	NKX2-5	SLC1A6	HTR1D	TP53TG3D	CACNG8	IGFBP1	ZNF180	ZNF569	TRIML2	CD70	FGF2	TENM2	LY6K	PSORS1C1	TRIM7	TGM2	DCAF4L2	GBX2	SLCO1B3	ZNF568	CITED4	FGF1	BDNF	FAM83A	NPPB	CCL2
**snu1077**	WNT2B	CXCL6	BARX1	IL11	OR51B4	JSRP1	HS3ST3A1	FOXS1	LHX1	UGT2B7	NKX2-4	GJB7	IGFL3	HOXA10	CDH6	RASL10B	FOXL2	HOXA7	GALNT14	CDKN2A	HOXA4	RAC3	ASCL5	HAPLN1	QPRT	HOXA3	HNF1B	GAL	ARSI	GALR2	CD70	HOXA9	KCNG1	GREM1	CARD18	PEG10	MACROD2	FIBCD1	PTGER2	PAX8	ZNF576	ZNF677	ZFHX4	ZNF771	CCBE1	HOXA6	DMRTA1	PPP1R14A	ASPHD1	DMKN
**snu1196**	KLK6	KLK5	NT5DC4	KLK7	PRICKLE4	MDFI	DEFB136	MIA	ONECUT3	MT1A	KLK10	CAPN8	RNF43	FIBCD1	LMTK3	IGFL4	HOXC11	AQP5	MROH6	COL9A3	CALHM3	MT1M	HOXA13	USP49	BMP4	EREG	ASCL2	SLCO1B3	CALB2	S100A5	FOXP4	NOTUM	ANKRD22	ZBED2	ANKRD65	TLX1	ZNF488	RASSF6	SYT8	TNS4	NIPAL4	EMX1	TMEM52	SGK2	FGF19	ETFRF1	CAPS	IFNE	HSD17B1	BYSL

**somatic cell**	VCY1B	OR13C3	USP17L20	OR4D5	OR1N1	USP17L11	USP17L18	USP17L22	USP17L15	DUX4	CYP21A2	SPACA5B	OR10G9	OR8D4	KRTAP10-11	PRORY	USP17L7	POTEB	TSPY4	CYP11B2	SULT2A1	CYP11B1	IFNL2	C4A	MC2R	CLEC4M	SIGLEC16	KCNJ18	HSFX1	PRR23C	SIGLEC11	LIMS4	TRIM74	OR3A1	STAR	NPIPA8	SPRR2F	NPIPA2	IFNW1	DUXA	FATE1	GSTT2B	GOLGA6L22	PANO1	VN1R2	OR2J3	PROK1	SHOX	FRG2	RHOXF2B
**somatotroph**	GHRHR	POU1F1	GHSR	OTOS	GH2	PRL	SSTR5	IGSF1	ARHGAP36	LHB	CHRNB3	TSHB	OR7A5	FNDC9	MRLN	CGA	FSHB	MYO15A	DEFA6	DLK1	CTXN3	KRTAP9-4	RESP18	OVOL3	DIRAS3	RIIAD1	POMC	TCEAL6	TMIE	TCEAL2	LRRC52	TDRD9	RXRG	FFAR4	CLDN9	PLA2G2E	CELF6	ABCC12	GNB3	OR10Q1	PAPPA2	KLF14	GPR179	FETUB	SYTL4	NPAS4	NEUROD4	CHGB	SIX6	GJB7
**somitic mesoderm**	CER1	TCF15	MEOX1	ARG1	FOXC2	HOXB1	EPOP	GRIFIN	HOXA1	ALDH1A2	LCE3B	HOXA2	CRABP1	MOGAT2	RIPPLY1	FIGNL2	PAX3	ZBTB12	RIPPLY2	PRTG	DMRT2	ZIC3	FOXB1	PPP1R1A	PRRX2	XKR5	STRA6	MACROH2A2	DACT2	SPSB4	RIOX1	JPT2	SELENOH	ALX1	FST	WNT16	FOXD2	HOXD1	MYCN	TEAD2	HAPLN1	SMOC1	RCOR2	TBX18	MAIP1	HOXD4	APLNR	HPF1	LEFTY2	LIN28A
**sperm**	SPEM2	STPG3	LLCFC1	H2AB1	TEX46	SPEM1	FSCB	TEX48	TEX37	SPPL2C	BPIFA3	ATXN3L	PRR30	TEX53	UBQLN3	CBY2	IQCF5	CCDC70	RNF133	TBC1D21	SPEM3	TXNDC8	PFN3	CCDC197	HSFY2	CCDC188	DUSP21	CCDC179	IQCF1	ZNRF4	SPATA32	DNAJB8	CABS1	SPATA3	SLFNL1	H1-7	CYLC2	SCP2D1	ODF1	IQCF2	TSSK2	ZCCHC13	PAGE3	TSPAN16	FAM170B	CCDC54	SMIM23	FNDC8	MS4A13	SPATC1
**spermatid**	ETDA	ETDB	OR14A16	SPATA31D4	HSFX4	SPANXN4	FAM205A	SPATA31D3	LYZL2	PRSS58	SPATA31E1	IQCF6	OR4M1	MS4A6E	OR2H1	CBLL2	FAM47B	SUN5	SMIM23	CCT8L2	OR4N4	SPANXN3	CDRT15	SPATA31A1	GOLGA6A	OR8B2	KIF2B	CCDC185	OR10J1	LYZL1	PRSS54	SPANXN2	CDRT15L2	TAAR2	IQCF2	MBD3L1	PRSS37	SMIM21	SPDYE4	PAGE3	SPACA4	GOLGA6L2	IZUMO3	SPATA46	LYZL6	TGIF2LX	CPXCR1	PRR30	UBQLN3	CCDC54
**spermatocyte**	BIRC8	FAM170A	POM121L12	TMEM225	LDHAL6B	WDR87	PRKACG	MGAT4D	ODF4	SIGLECL1	PDHA2	PRPS1L1	PPP3R2	RNF113B	GOLGA8G	SPATA16	DPEP2NB	POM121L2	SSMEM1	GOLGA6L2	SLCO6A1	HDGFL1	GOLGA6L7	ZPBP2	ADAM30	SPDYE4	PTCHD3	GOLGA6B	KRT72	SHCBP1L	ETDA	SLC25A2	LYPD4	ZSWIM2	LYZL6	TMEM89	ETDB	MS4A13	CBLL2	FAM186B	TMPRSS12	FAM205A	ARMH2	GAB4	IQCA1L	FAM47C	CCDC42	GOLGA6A	SLC25A52	MROH2B
**spermatogonium**	MAGEB4	PPP4R3C	TSPY10	POM121L12	SPATA31D1	SSX3	OR4M2	FAM47B	PNMA6E	OR4N4	OR8B2	KRTAP21-2	TGIF2LY	DUSP21	USP26	RBMXL3	DDX53	SPANXN2	SUPT20HL2	TEX13D	SPRR4	OR8B3	SOHLH1	TSPY2	DCAF8L1	TSPY3	FTHL17	LUZP4	PRSS38	TGIF2LX	FAM170A	FAM9A	OR10J1	OR8G5	TEX13C	ATXN3L	MAGEB6	SAGE1	EBLN1	POM121L2	SPATA31A1	LDHAL6B	TBC1D3B	POTEH	SPATA31D4	TEX19	TEX101	SPDYE4	CT47B1	CT45A10
**spinal cord interneuron**	FAM47A	HES3	EXOC1L	ADGRD2	TAS2R3	GRIFIN	FERD3L	CYP26C1	SOX3	BSX	KRTAP16-1	FOXB1	OLIG3	IL17REL	TKTL2	ARID3C	TUBA3C	FTMT	TAFA3	NEUROG1	DSPP	HOXD4	ZIC3	TAS2R16	CCNB3	SOX1	MSI1	HOXA1	NPBWR1	GJD4	RLN3	OPTC	SPRR5	RPE65	TEX49	NOTO	NMRK2	GPR101	KIRREL2	PROZ	SPATA48	NKX6-2	TECTA	TTC16	LRRC30	RHCG	NKX1-2	CRYBA1	EPOP	CDCP2
**spiral ganglion neuron**	LRRC30	ASIC3	HAPLN4	LRRC55	AMIGO3	POU4F2	VWA7	KCNS1	SCN4B	FGF10	LGI3	NEFH	FBXO40	SYT3	SLC15A5	SYT2	KCNA1	STAC2	CPNE9	P2RX6	SLC17A6	CORO6	LHFPL3	KASH5	NOS1	MYADML2	CLRN1	PVALB	SHOX2	ATP2B3	NEFL	ABCG4	GLDN	LANCL3	MDGA1	CDK15	PLA2G4E	JPH3	KRT222	IQSEC3	NEFM	CPLX1	CALB2	CLEC18A	KCNC1	CLEC2L	POU4F1	VAMP1	SIAH3	DIRAS1
**splanchnic mesodermal cell**	OR13H1	TAS2R16	OR6T1	NKX2-6	OR52D1	OR52M1	GLYATL1B	GPR101	MLNR	TAS2R43	DSPP	GATA5	GPR148	TUBA3C	SPRR5	CCDC105	LRRN4	HAND1	FAM240B	FTMT	KRTAP4-8	PIWIL3	TKTL2	TAFA3	NKX1-1	NMRK2	TUBA8	TAS2R3	HTD2	TPRX1	PITX3	CCNB3	RHCG	KRTAP16-1	PROZ	APLNR	MYOG	LRIT3	CLDN34	RLN3	KIRREL2	NTSR1	OR6F1	EVX2	TTC16	OCM2	TDRD15	GPHB5	DDX4	PTCH2
**splenic red pulp macrophage**	ITGAD	TREML4	SPIC	CD5L	CCR3	IL27	ADAMDEC1	PRG3	C1QC	CFP	C1QA	C1QB	ADGRE1	VCAM1	CSF1R	CD300E	SLC11A1	LILRB3	LRRC25	PAQR9	CAMP	ASB2	FCGR3B	P2RY13	HMOX1	CLEC1B	MARCO	PLA2G2D	GNGT2	UBD	GFRA2	EPO	GALP	CCL24	TMEM26	CD300LF	HCAR2	HCK	SLC40A1	NR1H3	CD300LD	PLD4	FCER1G	CELA1	MRC1	IL12B	SLAMF8	PILRA	LY86	CD180
**splenic red pulp macrophagec**	CCR3	SPIC	ITGAD	CCL24	TREML4	CD5L	UBD	ADGRE1	ADAMDEC1	PLA2G2D	KCNJ10	BTNL2	FCGR3B	GFRA2	PAQR9	C1QC	VCAM1	TMEM26	C1QA	C1QB	TIMD4	CFP	GPR141	MRC1	LILRA5	PILRA	MPEG1	CELA1	CSF1R	SLC40A1	LST1	P2RY13	HMOX1	CD300A	DNASE1L3	HCAR2	MERTK	ASB2	GNGT2	FPR2	SLC45A3	SMIM27	FCRL1	AIF1	TRPM2	BANK1	KCNK13	CD86	HPGDS	CD163
**spongiotrophoblast cell**	CEACAM3	TAF7L	TNFRSF9	CEACAM5	SCT	PGA5	PAPPA2	FBLN7	FLT1	GKN1	FOXO4	NRK	CITED1	PLAC1	SLC6A12	CREG1	GKN2	NCMAP	RARRES2	PLET1	ADA	CLPS	BHLHA15	CMA1	S100G	DERL3	CLEC4D	CSF1R	SCNN1B	MMP12	NPPC	PF4	PROCR	CCL7	AQP8	PLA2G7	DIO3	APOA2	CDA	SPP2	DTX1	PLTP	APOA1	GUCA2B	SLCO2A1	ITGB3	HMOX1	ANKRD33B	SLC25A31	MMP8
**squamous epithelial cell**	TMPRSS11B	SPRR2A	KRT78	MUC21	IL36A	SPINK7	MUC22	SPRR3	CRNN	RNASE7	SPRR2D	RPTN	TMPRSS11D	SPRR2E	TMPRSS11E	OR7G2	CRCT1	HCG22	PRSS27	A2ML1	SPRR1B	KRT13	OR10K2	OR2V2	SPATA31A7	OR1D2	OR6C4	TMPRSS11A	LCE3E	SPRR2F	LYPD2	SLURP2	IL36RN	OR10A3	KRTAP9-6	OR10H5	FAM25A	OR2A12	NCCRP1	FGF23	OR7D4	S100A7	KPRP	DUOXA2	OR6M1	SCEL	DEFB4A	PADI1	OR8G1	ADGRF1
**sst gabaergic cortical interneuron**	CORT	MEPE	TAS1R2	ZIM3	HTR1A	INSYN1	ASCL3	ANKRD34C	KRTAP13-2	IFNL3	AKAIN1	HAO1	SLC32A1	CRHBP	MRGPRX2	SLC6A7	DYTN	RXFP3	MAGEB18	SLC5A7	SLC3A1	SST	GPR151	CRYGA	GLTPD2	COL19A1	GABRG1	GDF1	TACR1	SPRN	FBLL1	PLA2G4E	IFNA2	GPR26	CASTOR2	NPY	CHRM2	BRICD5	FAM166A	CHRNB2	MYO3A	CRTAC1	SSTR4	KRT85	GPR62	CCDC38	ATP2B3	B3GAT1	MPPED1	TMEM179
**stad**	GAST	GKN1	RBP5	PGC	CA4	GKN2	MUC5AC	MADCAM1	RAMP3	MUC6	PLVAP	IGLL5	TCIM	TFF2	TM4SF18	TMEM273	AVPR2	MUCL3	FCN3	FAM167B	VWF	TFF1	APLNR	CLDN18	CLEC14A	CLDN5	TPO	LIPF	SELE	CYYR1	RND1	ADCY4	ECSCR	A2M	RNASE1	DEPP1	COL15A1	CLIC2	NKX2-3	MMRN2	REG1A	ZNF541	JCHAIN	JAM2	RAMP2	SLCO2A1	TMEM88	ROBO4	ADGRL4	FAM107A
**stem cell**	URAD	PROP1	ARSL	CYP2W1	OLFM4	MT1H	UGT2B17	LHX3	ISX	SLC39A5	LEFTY1	NOX1	MUC3A	FAM3D	ADH1C	UGT2A3	NXPE1	IGSF1	RNF186	AGR3	TMEM238L	MLN	MT1G	PNMA6F	CLRN3	NXPE4	ASCL2	CDX1	ANXA13	RBP2	NOS2	CDX2	PRAC1	MTARC1	PPP1R14D	CHP2	RBBP8NL	CDH17	OTC	MUC12	SULT1E1	DEFA5	FABP1	PTGDR	PHGR1	AMN	CCL15	RETNLB	TSPAN8	SRY
**stem cell of epidermis**	FAM25C	CHIT1	HS3ST6	ANXA8	CAPNS2	GPHA2	CLCA2	KLK9	LGALS7	LCE1B	WNT10A	SERPINB2	PGLYRP4	WNT3	KRT14	KRT5	IL22RA2	KRT32	SERPINB7	WNT16	KRT15	SLC6A2	COL17A1	SLC25A48	KCNK7	EPGN	BNIPL	KRTDAP	LTB4R2	FCGBP	CASP14	IL20RB	PHOSPHO1	KLRG2	DSC3	PLA2G2F	PKP1	LORICRIN	CCDC27	LY6D	POU3F1	TRIM29	NLRP10	FZD10	SERPINB5	IL1F10	KRT1	CFAP57	BBOX1	FGFBP1
**stem cell of gastric gland**	PGA5	GKN1	GKN2	MUCL3	ATP4B	PGC	A4GNT	ANXA10	PDIA2	GLP1R	MUC6	SLC9A4	CLDN18	PLA2G1B	VSIG1	TFF2	SULT1C2	HABP2	PNLIPRP2	MOGAT1	CLPS	MUC5AC	FAM83C	PPP1R3A	ERN2	ATP4A	CPN1	PSCA	IYD	GCNT3	CCKAR	CTSE	TFF1	IFNA16	SULT1B1	LRRC31	CWH43	PLA2G10	GALNT4	RNF225	VSIG2	CFI	LYPD8	TNN	LGALS2	IHH	ADH7	NPY4R	TMED6	GATA4
**stratified epithelial cell**	CRNN	KRT6C	SPRR2E	SPINK7	SLURP1	MUC21	CYSRT1	DYNAP	A2ML1	KRT6B	MAB21L4	SERPINB13	SPRR2D	SPRR1B	RAET1L	CRCT1	IVL	FAM240C	TMPRSS11B	KLK13	CAPN14	TMPRSS11A	GBP6	TGM3	KLK12	CLCA4	TGM1	CLDN17	TMPRSS11D	SERPINB3	LGALS7B	SPINK5	CALML3	S100A7	HCG22	DSG3	KRT16	FAM83C	ZNF750	DSG1	OTOP3	TMPRSS11E	SERPINB4	KRT31	SDR9C7	NIBAN2	IL1RN	CYP2C18	SPRR2F	KLK11
**strial intermediate cell**	SMIM41	CCN4	GASK1A	CCDC102B	PALM2AKAP2	COL6A6	DEFB131B	CACNA1C	DUXB	ABCA6	ABCA10	INSYN2A	PIEZO2	CEMIP	HMCN1	HPSE2	AWAT1	LAMA2	AOPEP	SHOC1	PRDM6	CPHXL	TEX52	ERFL	TBX5	CYP7B1	RYR2	PRR16	CCBE1	SNED1	UPK3BL2	KIF26B	BNC2	ROR2	FGF7	EDDM13	PRKG1	ADAMTS12	ITGA8	FBXL7	PLEKHH2	ABCA9	CACNB2	PDZRN3	ST6GALNAC5	SDK1	SVEP1	GALNT17	ZFPM2	FAM227B
**stromal cell**	MRGPRG	OR8K5	CCL13	ADAMDEC1	ADH1B	CCL11	HPSE2	HAS1	ABCA8	MMP27	DPT	TCF21	SCARA5	CCN3	CCL8	OR2AJ1	MXRA5	OGN	ABCA6	CLEC3B	LUM	COL14A1	CCN4	LAMA2	MMP3	COL6A6	ITGA8	ECRG4	ADGRD1	TNFAIP6	AGTR2	CCL7	FNDC1	C1R	CYP21A2	LRRN4CL	FGF7	COL21A1	BMP5	TNXB	CYP17A1	DCN	MFAP5	MFAP4	C1S	CD248	VEGFD	SULT1E1	IL6	PAMR1
**stromal cell of bone marrow**	IBSP	SERPINA12	ADIPOQ	GDPD2	MMP13	ESM1	ANGPT4	SP7	BGLAP	PODNL1	CCL19	KNG1	C4B	CHRDL1	SLC36A2	SFRP4	EBF3	RSPO2	SLC26A7	DMP1	GPR50	CXCL12	BMP5	IFITM5	PDZRN4	DPEP1	AGT	SMCO3	LEPR	GGT5	TNC	VCAM1	TNFSF11	PI15	P2RX2	PHEX	FAP	WDR86	GPR88	VIT	CXCL14	CAMP	ADAMTS2	IL34	FGF7	WIF1	PAPPA	COL8A1	MEPE	ISLR
**stromal cell of ovary**	TNFRSF6B	ZNF705A	OR4F17	OR10H1	THSD8	OR52N1	CLEC18C	PRXL2B	ARSH	SPINK9	OR1A1	FBXW12	SERTM2	TBC1D3K	OR2L2	TBC1D3E	OR10K1	RAB6D	TMEM190	ANXA10	CCN4	SERF1A	ANXA8L1	H2BC10	CPLANE2	DIRAS3	VSIG10L	AMH	TCF23	IFITM5	H2AC14	EIF3CL	CTAGE6	OR2B11	H2AC13	CZIB	H2AC16	NPPB	CTAGE8	NPAS2	H3C3	INSYN1	EPGN	H2AC17	CXCL6	OR2AP1	CCN1	H3C12	OR10G3	CTAGE15
**sum149pt**	PNLIPRP3	SETSIP	KIF4B	KRT81	SPINK6	ALPP	MCF2	SERPINB7	EPGN	PF4	CRYBB2	KRT6B	CSNK2A3	RAB42	GREM1	CTAGE1	HOXC12	CXCL3	CXCL1	LAMB3	CXCL6	KRT6A	RFLNA	LAMA3	AARD	EREG	INHBA	S100P	KRT86	HPDL	CA9	KRTAP13-2	MYH15	CSTA	FPR1	ZDHHC11	WNT5A	KLHDC7B	CYP1B1	ANPEP	LPAR3	TAF1L	HAS2	KLHL35	PPP1R2B	BNC1	SMIM2	HES7	EN1	CSF2
**supporting cell of cochlea**	IL36G	CLEC4D	CEACAM18	FPR2	HCAR2	CLEC4E	CAMP	MMP9	LILRA6	CXCR2	IL1R2	SPATC1	CD300LB	CCR1	CD300LF	NPSR1	IL18RAP	CSF3R	C5AR1	IL1B	CLEC5A	CXCR1	CD177	CD101	CASS4	PTAFR	CD33	FPR1	TECTB	PLB1	RASGRP4	KRT83	MRGPRX2	PGLYRP1	FCGR3B	HDC	CLEC7A	OSM	DHRS9	PGLYRP3	BMX	SELENON	PILRA	CXCL2	ASPRV1	GDA	PLEK	NLRP12	ITGAM	NCF2
**suppressor macrophage**	CD209	CCL13	LILRB5	CCL18	FPR3	MS4A4A	FOLR2	MMP12	CCL4L2	MS4A7	ADGRE1	TREM2	CCL8	C1QC	C1QA	C1QB	MS4A6A	FCGR2A	C3AR1	VSIG4	SIGLEC12	SDS	GPR34	CD163	ADORA3	OTOA	SLAMF8	STAB1	F13A1	FCGR1A	FCGR3A	MSR1	LYVE1	CCL3	CD14	MRC1	RAB7B	LRRC25	SLAMF9	ADAMDEC1	CLEC10A	AIF1	CD300LD	FCGR2B	SLCO2B1	SIGLEC1	CXCL8	MS4A14	MPEG1	TMIGD3
**surface ectodermal cell**	BHLHA9	RAX	FOXN4	OC90	VSX1	TAS2R9	TKTL2	NKX1-1	PLEKHG7	TAS2R8	FAM47A	TUBA3C	FTMT	OR52M1	EXOC1L	SLC2A2	TAS2R42	CCNB3	TAS2R3	TAFA3	IL17REL	FAM240B	TAS2R7	DSPP	KIRREL2	GPR101	SOX3	SPRR5	GLYATL1B	TAS2R13	NMRK2	GJD4	NTSR1	CLDN24	ZP4	MSI1	PRDM12	CCER1	TAS2R43	TTC16	PROZ	MAB21L2	TMEM221	GFRAL	OPTC	CYP26C1	WNT6	SOX1	KRTAP12-2	OR1L1
**sw480**	FUT5	GJA9	KRTAP19-1	PRICKLE4	CSNK2A3	TREX2	FBXO10	OSTN	SEC31B	KRTAP21-2	FOXD4L3	CCDC142	KIF4B	HOXA9	MAP1LC3B2	ALKAL1	CRYGD	HOXA6	HOXD10	PAGR1	SCML1	KIFBP	ZNF547	ZNF23	KRTAP19-3	ZNF530	DNAH14	IRS4	OR51B2	TMSB15B	ZNF711	HNRNPCL1	ELOA2	CKAP2	TNNI3K	LIN28B	FOXC1	RPEL1	UBE2NL	HOXA5	KIAA1586	RAB39A	POLN	EN2	HOXD13	CMC4	XRCC2	CCNE2	CA2	POU4F1
**sw579**	APCDD1L	IL24	KCNG1	MAGEC2	SLC14A1	FHAD1	MMP1	CSF3	STEAP1B	FAM180A	RFX8	TMEM244	LRRC15	HAS2	ESM1	ADTRP	STATH	SHISAL1	LYPD1	CFAP251	CTHRC1	FAM20C	IL11	COL7A1	LY6K	CNIH3	HHIP	GREM1	FGF5	MSC	EN1	BDKRB1	ANPEP	ADRA1D	ARHGAP22	LTBP2	COL1A1	GDNF	COL6A3	CYP27C1	EVA1A	INHBE	ROBO3	STC2	APOBEC3B	PRRX1	MALL	THY1	NKX6-1	HIC1
**sw900**	CALCA	B3GNT6	PRSS1	VIP	SLC1A7	FAM9B	TFF1	PAEP	SYTL5	RAB39A	DRD2	NTS	AMBP	TFF3	CATSPER1	VIL1	IL17B	VGF	LCE1E	BTBD16	OR4C6	XKR7	KCTD16	CEACAM6	VSTM2A	ASCL1	GPER1	ST6GAL2	SIGLEC15	KCNF1	RNF224	CD163L1	GJB7	GNG4	CAPN8	GOLGA7B	SYT13	AKR7A3	AGTR1	RIMKLA	CRYBA2	PRSS3	PROC	EREG	WDR72	LCE2A	CDK5R2	LCN1	WFDC10B	PTHLH
**sw1783**	CCL26	BDKRB1	SPOCD1	KRTAP1-1	LAYN	IL11	S100A3	CD70	ZNF469	HMX1	ZP1	KIAA1755	CPA4	COL6A2	SHISAL1	LOXL1	CCL2	FGF5	ARSJ	HOXD11	COL1A1	GLI1	GDF5	EVA1A	IL6	IL13RA2	THY1	LOX	NPPB	COL6A1	EN1	FAM20C	MYEOV	SYDE1	NKX2-5	SERPINE1	BDNF	BAALC	IGFBP6	SIGLEC15	FOXS1	TMEM171	CREB3L1	SUGCT	KRTAP1-5	CXCL3	FCRLB	APOBEC3B	SMIM10	EFEMP2
**sw1990**	SLCO1B3	IL37	KRT81	MYEOV	IFNE	ZNF185	KISS1	RFLNA	CGB3	KIAA1549L	FERMT1	MELTF	CST6	DKK1	RFX8	CYP27C1	PLEK2	ANKRD1	BDNF	CALB1	TGFBR3L	CXCL5	TGM2	SOX7	ADAMTS12	MALL	LAMC2	FJX1	LCE3E	ITGBL1	PRSS3	TEAD4	TMEM171	SLCO1B7	AHNAK2	SLFN12	DCBLD2	AMIGO2	G0S2	CALHM3	FOXD1	CCNQ	ADAMTS6	TRIM7	KRT7	CGB7	EREG	LAMA3	BLID	GALNT5
**syncytiotrophoblast cell**	LGALS13	LGALS16	PSG7	CGB5	PSG11	PSG8	CGB8	LEP	CGB3	CGB7	LGALS14	CGB2	PSG3	PSG6	CGB1	GH2	INSL4	PSG2	XAGE2	CYP19A1	PSG5	CSH2	SIGLEC6	PSG1	SPANXN5	ARMS2	SLC22A11	XAGE3	KRTAP26-1	CSHL1	PSG9	CSH1	PSG4	MAGEA8	PAGE4	CGA	SLC52A1	GCM1	LAIR2	PAEP	CRH	OLAH	OR7C2	GH1	HTRA4	HSD3B1	TINCR	GLDN	VGLL1	CYP11A1
**synovial cell**	SPDYE5	ZNF630	ARR3	ZNF709	TMEM233	SYCP2L	CCNP	ZNF214	MMP25	ZNF79	ADCY8	SYTL5	SLC28A2	ZNF700	ZKSCAN2	ZNF319	PRORP	SOX8	ZNF558	ZNF667	CYP2E1	DNAH1	HSF4	PHEX	ZC3HAV1L	GARNL3	CMYA5	PDE4C	ZNF586	ZNF382	LRAT	ZNF774	MTBP	NEK11	STARD9	STRIP2	FOXG1	FAM200A	ZNF772	CUL9	PADI2	IL18BP	ZNF490	ROR1	ZSWIM5	TRIM52	TMEM35B	ESPL1	ZNF780B	ZXDA
**t47d**	H3C10	H4C8	PYDC1	H3C6	CLEC3A	PLAAT3	SPINK13	H2AC8	XAGE2	FAM174C	H1-2	OTOR	SERPINA6	IQANK1	GAGE1	SPRING1	IFTAP	ANTKMT	H2BC4	PIP	H2BC8	PGR	SRARP	MYRFL	H2BC5	H2AZ2	ODAPH	CLPSL1	SLC66A3	H2BC9	H2BC15	H1-0	COA8	MICOS13	PRXL2A	PRLR	LMTK3	H4C5	SPDEF	MICOS10	VARS1	MACIR	ELOVL3	H2BC21	CFAP20DC	PGAP6	GARS1	RBIS	NTAQ1	CEP20
**t cell**	OR11H2	ERFL	INSYN2B	KIR2DS4	MS4A18	SLFN12L	OR52A5	THEMIS	OR10G2	KIR2DL4	CCL1	ZNF831	IZUMO1R	KLRC4	NIBAN1	PDCD1	CXCR6	SIRPG	IFNG	ICOS	INKA2	IL26	FASLG	GPR174	CTLA4	CXCL13	APOBEC3H	CCR8	CD28	DEFB131B	CCDC200	CD8A	TIGIT	PVRIG	ITK	CD3G	CD6	OR4K2	MS4A4E	ZNF683	CD5	SH2D1A	OR6N1	CD2	TENT4A	GRAP2	CD3D	CD96	STPG4	KLRK1
**t follicular helper cell**	OR4C45	CXCL13	SIRPG	PDCD1	IL21	TIGIT	USP17L12	ICOS	CD40LG	USP17L20	OR11G2	PVRIG	USP17L11	TNFSF8	ZNF80	OR5D16	TNFRSF4	CTLA4	USP17L18	BTLA	CCR4	TRIM49D2	OR4A15	OR11H1	TRAT1	ZNF831	FOXP3	IGFL2	CD5	OR2A25	OR1L4	KRTAP29-1	CXCR5	CD6	CCR8	RTP5	ITK	OR6B2	KLRB1	OR2G3	CD2	USP17L22	USP17L17	CPA5	CD28	SH2D1A	PPIAL4E	OR5M8	SLAMF1	LY6G5B
**t-helper 1 cell**	OR4C45	OR5L2	OR4C13	OR12D2	OR2T5	USP17L12	OR52B4	OR4C46	OR2A2	OR5M10	OR4F4	OR10G8	OR11H1	OR52E2	OR11H12	OR2T35	OR4L1	OR4F3	OR2T29	OR51T1	OR52E6	OR4D2	OR10K2	OR5J2	USP17L15	OR1D5	OR52L1	OR52J3	OR11H6	OR4A16	CDY2B	OR2M5	OR2M2	OR9K2	OR13G1	OR4D11	OR6M1	OR2T6	OR2T27	OR1N2	CDY2A	OR8J1	TAS2R38	OR51A2	OR4S2	OR10T2	OR4A5	OR6K2	KRTAP6-3	OR8K1
**t-helper 2 cell**	IL5	SPATA31C2	IL13	TMEM78	IL4	IL9	PTGDR2	GPR25	FFAR3	IL9R	GMCL2	OR2A7	GPR42	ICOS	OR4D1	IL17RB	SEPTIN1	CCR4	NUTM2B	TMEM273	TRAT1	OR3A1	GOLGA8J	PRR33	SIRPG	CD40LG	GOLGA8G	H4C14	EOLA2	KLRB1	PPP1R2B	TNFRSF4	CXCR6	H4C9	TIGIT	SLC66A3	ITK	IL7R	ZNF831	CD3D	SLAMF1	TNFRSF25	CD3G	CD2	RAB6D	F8A2	CD28	TESPA1	KIR3DL2	CCR7
**t-helper 17 cell**	IL17A	IL22	IL26	IL23R	IL21	CD40LG	CSF2	OR11G2	CXCR6	IL3	NCR2	CCR6	IL17F	KLRB1	TRAT1	PDCD1	SIRPG	GPR25	GPR15	FASLG	ICOS	TNFSF8	THEMIS	TNFRSF4	IFNG	CD6	PVRIG	SLAMF1	CCR4	CD2	CD5	CD96	KRTAP12-3	ODAPH	KLRF2	TNFRSF25	TMEM273	RGL4	IL7R	TMIGD2	IL2	OR2A25	IL9R	IL12RB1	CD3E	ZNF831	CD3D	APOBEC3H	SLFN12L	ITK
**tanycyte**	GPR50	NKX2-4	SLC39A12	COL23A1	RAX	AVP	VWA5B1	RTL1	MLC1	HEPACAM	KRTAP17-1	SLC6A11	PMCH	COL25A1	GRPR	PTX4	CDKL4	CFAP100	IL1RAPL2	MMD2	TSHR	BCAN	MAGEL2	PTPRT	LRRC9	THRSP	NR2E1	FGF10	SORCS1	FEZF2	ENKUR	LIX1	CFAP74	SIX3	CBLN4	MAPK4	CCDC85A	NKX2-1	EFHB	SHC3	TTYH1	NIM1K	ILDR2	DCHS2	CRYM	CFAP126	SORCS3	RFX4	DYNLRB2	OXT
**tc1 cell**	H2AC16	OR6N2	H4C2	H3C1	H4C9	H3C11	H2BC10	H4C12	H3C12	H2BC14	H4C1	H2AC13	OR10P1	H3C10	H2BC7	H2BC9	H2AC20	SLC2A14	PPP1R2B	CTAGE6	H2BC21	KRTAP12-3	H2BC17	OR5K2	LACRT	RGPD2	H4C11	H2BC3	SLC35G4	H1-4	H2BC15	H4C5	OR1S2	IFNG	GPR15	NIBAN1	KIR2DS4	MPZL3	OR6X1	RESF1	H2BC12	CCR8	DEFB107A	H2BC11	TEX54	CD8B2	H2BC13	ZNF831	H2AC6	SPRR4
**tc17 cell**	OR1D4	IL17A	OR1D5	H4C9	IL26	H4C6	GPR15	NPAP1L	H4C12	H2BC10	NUTM2F	OR5K2	PRAMEF18	OR10AC1	CXCL13	IL17F	H3C6	NLRP2B	H2BC17	CXCR6	H2BC7	CTAGE6	OR5M10	H2BC12	CTLA4	OR10A5	OR1J1	H4C2	RESF1	JAML	NIBAN1	ODAPH	H2BC14	GNLY	H2BC9	PRAMEF1	H2BC15	CD96	H2AC16	ZNF831	RBMY1J	MARCHF7	H4C5	OR2V1	OR2AE1	OR4E2	ZBED2	H3C10	OR10AG1	CALHM4
**tccsup**	SPANXD	SPANXA1	SPANXA2	VCX2	COLEC10	SPANXC	PAGE5	TNFSF4	CPA4	DIO2	DIRAS3	ADAM18	TMEM52B	SPOCD1	TFF2	VCX	KRT81	LHX9	CACNG6	C1QL1	RFLNA	CSAG1	BDKRB1	SLC12A8	CACNG7	HMSD	ZSCAN31	TP53I11	HSPB3	TMEM196	HRCT1	GNAT3	ASPHD1	SYCP2	HTR7	DKK1	MAGEC2	GPRIN2	OXTR	FOXL2	NKX2-5	CDKN2A	BDNF	TMSB15B	SUCNR1	HTR1D	TMSB15A	STC2	IGFN1	CACNG8
**te1**	KRTAP3-1	KLK5	SPINK6	CPA4	KLK6	KRT81	KLF14	CALML5	HMX1	GPRIN2	POTEG	ALX3	TESMIN	FAM131C	SUSD2	GAL	CPNE9	CD163L1	PAGE2	SOX15	ALPP	SLC1A6	PRSS22	PI3	KIAA1755	PRSS21	LY6K	CST6	RTN4R	MYEOV	TMEM139	CAGE1	HOXC12	UPK2	TMEM40	FAM9C	VGLL1	ZBED2	HOXA11	CFAP251	LHX5	NT5DC4	ZNF853	RNF223	FHAD1	MIB2	CITED4	FOXE1	RHOD	FAM90A1
**te6**	FLG	CYP4F11	ARTN	MAGEA1	BRDT	KRT6A	MAGEB2	PTHLH	KLRG2	ZBED2	S100A2	FST	DMRT2	TPRG1	CYP4F3	AKR1C1	MAGEA4	CYP1A1	AKR1C2	TRIM16L	ZNF556	GPX2	GBP6	RNF212	CLEC2L	DMKN	SLC7A11	PLEK2	CHRNA5	CSTA	TP63	CCNA1	EPHX4	ZNF114	AKR1B10	FADD	LHX6	GPR87	DSC3	NRG1	PRSS21	NKX2-8	S100A14	SLC35E3	CIBAR1	POPDC3	SOX2	AKR1C3	MMP10	DLK2
**te14**	STATH	TMPRSS11D	TNFSF18	SPANXC	SPRR2A	SERPINB13	KRT13	KCNK9	MAGEA11	BARX1	KLK10	KLK11	ALDH3A1	AKR1C2	OTOP3	ERVMER34-1	KRT6A	CYP4F11	NKX2-8	LY6D	CALML3	SPRR1B	PRDM13	FABP6	FOXL2	GALR2	SPANXD	KLK13	AKR1C1	SPINK7	SPRR2E	AKR1C3	SERPINB3	IRX4	TMPRSS11E	TP63	NMU	ARTN	H2BW1	WNT10A	TLX1	CFHR3	GAL	FOXF2	LY6K	KRT5	HOXD11	GPX2	PTHLH	S100A2
**ten**	CALCR	SFTA2	KCNK15	FGF18	UGT2B7	WNT7A	PADI1	CST6	KLK6	FOLR3	DEFB136	HBE1	TGM7	TFPI2	ANKRD2	RAB42	MMP7	HOXA4	PDZK1IP1	STAC	QPRT	HOXA9	PPP1R14A	ERVMER34-1	SLC5A5	VGLL1	HOXA3	ERP27	CCNE1	JSRP1	EREG	SPP1	ZBED2	APOL1	KLK11	HKDC1	CDKN2A	PAX8	DENND2A	RNF224	PDE4C	FOXE1	F12	TLX3	DCDC2	MACROD2	PPP1R1C	TGM5	CXCL5	RHPN2
**tendon cell**	UTS2R	TNMD	PTX4	KERA	CILP2	ANGPTL7	FOXD4L4	CHAD	COMP	ANGPTL5	THBS4	CILP	FOXD4L5	SCX	OR5AU1	FMOD	CPHXL	MYOC	CHODL	PRG4	NCBP2L	COL11A2	SSC5D	FAM180B	OR6A2	MKX	CPXM2	NUTM2E	EVX2	GOLGA8J	PCARE	CCN3	ITGBL1	OR2AG2	COL8A2	ASPN	TMEM95	FIBIN	MSTN	GALR2	FNDC1	ANGPTL1	OR3A3	CCN4	C1GALT1C1L	MEOX2	OGN	FAM180A	COL11A1	MATN4
**theca cell**	GASK1B	GTF2A1L	C1QTNF5	INKA1	CCN4	CCN1	LDHAL6A	ATP1A4	GSTA5	PJVK	TCF21	KIAA1755	MMP23B	HOXD4	KLK15	EGR3	DIPK2A	PADI6	KIAA1210	RAD51AP2	TUBA3E	ZNF879	NR5A1	REELD1	TGM7	OGN	COLEC11	LRRC17	TERB1	TENT5A	HIC1	EDNRA	COL14A1	PLAAT3	CPLANE2	ASTL	CZIB	KISS1	RDH5	ADGRA2	ZSCAN23	CFAP410	PRSS35	DHRS2	FIGLA	FAM228A	TCF23	ZNF300	TNFRSF6B	COL6A6
**thp1**	AZU1	CXCL11	NKX2-4	MLNR	SDS	ALKAL1	FCGR1A	APOC2	CHI3L1	RNASE2	TKTL1	DYNLT5	ETV7	ELANE	MCEMP1	TREM2	BHLHE22	SLAMF8	SOWAHD	BARX1	SP9	GOLGA8N	CXCL9	SLC22A31	RAB7B	CD70	SIGLEC11	PRTN3	RUFY4	CTSG	OTX1	STAC3	SHOX2	GBP5	DMRTA2	IL4I1	TMEM150B	CEBPE	CCL13	PPP1R27	ANOS1	COL9A2	TFEC	CMPK2	IL6R	APOL4	ADORA3	SPI1	IGFLR1	OR52K2
**thymic macrophage**	CD300C	CCR9	HAVCR1	KMO	UPB1	TMEM221	COX6A2	XKRX	TLR9	PPFIA4	RNASE6	PLD4	CLEC9A	KPRP	SPIB	MPEG1	CLEC10A	TMEM229B	SLAMF9	ITGAD	TLR7	LY6D	FOXR1	TIFAB	ATP2A1	TREX1	IRF8	FCRLA	GAPT	KLK1	FLT3	RUNX2	LAIR1	DNTT	CARD11	PACSIN1	LAG3	CCR5	MYH13	LEFTY1	CLEC12A	P2RY14	SPNS3	CYTH4	LY86	BHLHA15	CYBB	CSF2RB	ADAM11	FGR
**thymocyte**	H2AC15	H2BC3	GRAPL	SMIM38	CD1B	TRAPPC14	CCR9	OR10G2	RAG2	RAG1	CD1A	CD1E	H1-3	CD8B	TTC24	H2BC15	ENDOU	GYPE	HBM	CD8A	SLC22A16	MACIR	SIRPG	DNTT	TRIM34	THEMIS	SH2D1A	OR10T2	SLC4A1	OR2D2	LRRC37A	CA1	H2AC20	SPTA1	ZNF575	XCL2	PABIR1	CD8B2	PPIAL4H	TRIM10	DIPK1C	PGAP6	AHSP	RHCE	CD6	NBPF26	ZNF683	WNT8B	MARCHF8	PTCRA
**thyrotroph**	TSHB	POU1F1	FOXL2NB	PRLHR	FNDC9	ARHGAP36	IGSF1	LHB	GHRHR	DIRAS3	CGA	LRRC52	DIO2	FOXL2	TCEAL2	GPR6	RXRG	TCEAL6	SMIM18	TAS2R20	IFNL4	LHX3	TSPEAR	KRTAP12-1	OTOS	MYO15A	OPRK1	GJB7	BEX5	RIIAD1	TCEAL7	FSHB	CRHR2	TMEM249	COL22A1	DCAF12L2	SHISAL1	CHGA	LY6H	CNGA3	MAGEL2	TCEAL5	HBG2	KRT74	PRL	ELAPOR1	ASPDH	SMIM17	PPEF1	NXPH2
**tip cell**	OR6N1	CCL18	OR7A17	OR6C75	OR51M1	OR2B11	H2AB3	OR10K1	OR52N1	ABCB11	OR10G3	GOLGA6L6	MCEMP1	SPINK9	OR11G2	MARCO	GOLGA6L1	OR10H1	OR2A25	OR52I1	FCN3	FOLR3	GOLGA6L22	GVQW3	KIR2DS4	OR2AP1	MS4A4E	DUOX2	VSIG4	TAS2R4	OR10A2	OR7C1	TSGA13	DNASE2B	PCDH11Y	H2AC18	TREM1	OR1A1	TASL	OR4D9	MS4A4A	OR1I1	CYP4F8	OSCAR	OR52A1	TBC1D3K	HTR3B	RETN	FCGR1A	TNFSF13
**tissue-resident macrophage**	FAM180B	FOLR2	MS4A6A	MS4A4A	FAM162B	FCGR2A	CILP	AVPR1A	NLRP3	HAS1	THBS4	LILRB2	FAM153B	MS4A7	SFRP4	ADM5	VSIG4	C5AR2	MYOC	MXRA5	RNASE6	ADAMTS4	ADGRE2	ABCA8	C3AR1	GPR132	MSR1	RAB7B	TNNT3	OPRD1	HLA-DQA1	SPI1	CD14	DPT	ADORA3	IL12B	CD163	COLGALT2	CLEC9A	GPR34	IL6	GALR1	SLC1A7	SLAMF9	LRRN4CL	AIF1	TLR2	GJC2	CCL14	C1QA
**tonsillar macrophage**	IL22RA2	SIGLEC12	ADAMDEC1	GPR42	MMP12	DCANP1	MMP9	SIGLEC14	ETV3L	SIGLEC7	SIGLEC10	CLEC6A	LILRB2	FPR3	LILRB1	CCL4L2	CD1E	IL4I1	CLEC4A	HLA-DQB2	FCN1	LILRA1	LGALS2	MS4A6A	SLAMF8	HLA-DRB5	HLA-DQA1	GPR84	SIGLEC9	LYZ	LRRC25	CEACAM3	LAMP3	ADGRE2	TLR8	ALOX15B	HAMP	LILRB4	S100A12	HLA-DQB1	EBI3	CEACAM4	FPR1	ITGAX	HLA-DMB	CCL3	IGSF6	RNASE2	HLA-DQA2	CD300C
**tov112d**	FAM9C	HOXC12	VGLL2	C1QL4	KISS1R	DSC1	HOXC13	MMP10	HBQ1	DSG1	VAX2	GCG	MEIOB	NPW	HOXC6	NTS	PAGE2	RTBDN	HES7	MAGEA4	SPDYC	HOXC10	CA11	FAM133A	IRX6	CLDN11	HOXC9	PRSS12	ADM5	PRAME	MAMSTR	HSD17B1	HOXB5	BARX1	KCNQ2	HGF	CSAG1	PASD1	PAGE5	TMEM100	HOXC8	CBS	SKIDA1	REEP2	SAMD11	CCDC106	TMEM253	PTH2	NAA11	PALM3
**tracheal epithelial cell**	MDFIC2	CNTNAP3C	CCDC195	CTXND2	DCAF8L2	POTED	DAOA	OR4N2	OR51V1	CLEC6A	OR6K2	HNRNPCL2	OR4C3	DAZ3	TMEM275	ANKRD30BL	OR5AR1	RGS21	PPIAL4H	MMP26	POTEA	DEFB131A	MCHR2	CFAP97D2	OR4C15	TGM6	KRTAP5-11	CAPN14	TRIM48	POTEB	OR4C11	IQCM	TRIM49B	SSX7	TRIM64C	OR5W2	ARSH	OR5V1	POTEB3	IFNA6	HTR3D	CNBD1	NFILZ	SERPINB3	CFAP47	OR52A1	NLRP13	ARSF	OR5AP2	OR5T3
**tracheobronchial smooth muscle cell**	OR2A14	MYOCD	GASK1A	HPSE2	CC2D2B	FAM240C	CACNA1C	NIBAN1	IRAG1	MYH11	PALM2AKAP2	SYNPO2	AOPEP	DEFB131B	PRDM6	LMOD1	AWAT1	CCN4	OR8A1	OR51I2	TBX4	CNTNAP3C	OR2A1	PDE3A	PRKG1	EDDM13	GSG1L2	RYR2	FHL5	PDZRN3	MAGEC3	RAB40A	ZFPM2	INSYN2A	CCDC102B	CACNB2	ITGA8	KCNT2	SOX5	SLC22A24	STPG4	COL21A1	SH3RF3	SLIT3	BMERB1	SORBS1	CLDN20	GALNT17	TBX5	ATP10A
**transit amplifying cell of small intestine**	SPACA5B	OR2J3	UGT2B17	PRORY	PPIAL4A	NXPE1	GOLGA6D	PRAC1	MINDY4B	PPIAL4E	PPIAL4F	TMEM238L	URAD	ADH1C	NOX1	UGT2A3	FAM240A	SMIM32	MYOCOS	CHP2	SNORC	FAM3D	GALR3	MS4A12	KRT3	KRTAP13-2	KRTAP5-10	MUC3A	PHGR1	PRAC2	BTNL3	UBE2NL	LEFTY1	NXPE4	ZNF648	PPP1R14D	ANKRD40CL	KRTAP5-2	MOGAT3	RXFP4	CCL15	DEFB131A	CASP5	ISX	GIMD1	TEX13D	GJD3	PLA2G2A	CCDC196	MUC12
**transitional stage b cell**	CLEC17A	FCRL2	MS4A1	FCRL3	FCER2	FCRL1	HLA-DOB	LCN10	BLK	TLR10	PAX5	CXCR5	FCRL5	BANK1	FCRLA	CD19	CD79A	HLA-DQA2	TNFRSF13C	CD22	HLA-DQA1	OR5AU1	HLA-DQB2	FCRL4	PARP15	HLA-DOA	CD1C	H2AB3	TNFRSF13B	HLA-DRB5	HLA-DQB1	CCR7	SPIB	FCMR	ZBTB32	GRAPL	UPK3BL2	PNOC	OR10H1	OR1A1	HLA-DPA1	HLA-DPB1	VPREB3	CD79B	BCL2A1	HLA-DRA	SCIMP	BTLA	NFKBID	LILRB1
**trophectodermal cell**	ERVV-2	MRGPRX1	GPR32	AADACL3	TAAR5	OR51B5	ERVFRD-1	TEX19	LGALS16	PRR23B	PIWIL3	ERVV-1	CLEC6A	PRR23A	CEMP1	CGB5	TRIM77	MAGEC1	MS4A15	PRR9	CCDC116	RFPL4B	HYAL4	TMEM114	GPR78	ZNF560	TFDP3	IL36RN	HAVCR1	CCKBR	UTF1	CGB8	LEP	TSPYL6	CGB7	NANOGNB	ZNF878	ZNF729	ZNF728	KDM4E	ZNF835	WNT16	TEKT5	GCM1	ADAM18	DNMT3L	ALPP	ELF5	NPFFR2	RBBP8NL
**trophoblast cell**	TRIM64B	NLRP9	ISM2	LAIR2	CSH1	HTRA4	DMRTC1B	GCM1	LVRN	TAC3	AADACL3	CGB3	HLA-G	XAGE3	GJA5	KRTAP2-1	PSG2	GPR32	MAGEA8	PRAMEF12	CLDN19	CSH2	MMP12	NOTUM	PSG3	SLC6A2	PSG5	OR2T11	KRTAP29-1	LEP	CGB8	CALHM3	AOC1	VGLL1	HSD3B1	EBI3	OR1E1	SYT8	PAPPA2	ANGPT4	CGB5	ETDB	NOS2	PLAC1	IFNK	CHRM5	CGA	DCST2	GPR78	GPR50
**trophoblast giant cell**	CSHL1	CSH2	PSG9	PSG6	PSG3	PSG2	GH2	PSG11	CSH1	PSG1	LGALS14	EZHIP	PSG5	CYP19A1	PSG4	PSG7	LGALS13	ERVW-1	ALPP	PSG8	CGB3	KISS1	MTNR1B	ERVV-1	PLAC1	DPEP2NB	PAPPA2	CALHM4	TRIM40	INSL4	ERVFRD-1	CGA	SMIM9	TRPV6	GPR78	PAPPA	NIBAN2	GCM1	GH1	ADAM12	STS	GNGT1	SVEP1	HSD17B1	HSD3B1	H2BC12	VGLL3	GLDN	EBI3	CGB1
**type b pancreatic cell**	G6PC2	INS	UCN3	SLC30A8	KCNK16	FFAR1	PRSS53	IAPP	SHISAL2B	CFC1B	MAFA	TMEM271	PDX1	FAM151A	ABCC8	GPR142	ADCYAP1	SSTR3	GPR119	GJD2	SCGN	NKX6-1	PCSK1	INSRR	RFX6	TMEM215	GLP1R	TRPM5	SLC2A2	OR13A1	AKAIN1	GPR148	CABP7	NPS	ENTPD3	OR5B3	CELA3A	PNMA8C	GCK	PLA2G2F	IGFALS	SST	PCSK2	ANKRD34C	C2CD4B	FFAR3	MLXIPL	NKX2-2	PPY	KCNJ11
**type i pneumocyte**	GGTLC1	MS4A15	AGER	GPRC5D	GGTLC3	ATP13A4	OVCH2	SLC9C1	CLDN18	ROS1	SFTPB	SLC22A31	SCEL	WFDC10B	OR5P3	PEBP4	ERFL	C4BPA	ANOS1	WNT3A	OR5P2	ITLN2	CLIC5	PRRT1B	NKX2-1	CNTN6	COL4A3	GPC5	GPR39	SHOC1	SCNN1G	SFTPA2	MAB21L4	KHDRBS2	UPK3BL2	STPG4	GRAMD2A	CYP4Z1	SFTPD	ZNF385B	DEFB131B	RTKN2	SLURP2	LMO3	DAPK2	SLC34A2	SFTPA1	DUOXA1	NDNF	COL4A4
**type ii cell of adrenal cortex**	CYP11B1	HSD3B1	APOC4	CTXN3	STAR	CYP11A1	KCNK3	MGARP	HAO2	AKR1D1	CALN1	MRAP	SMPX	LY6D	GPR55	CPB1	WNT4	GSTM2	RAPGEF4	VSNL1	TPBGL	PRLR	C4B	HOXB8	BCAN	CPE	C1QC	FDX1	C1QB	CELA1	CMA1	FADS6	AEBP1	GPIHBP1	GSTM1	FABP4	C1QA	MMP12	PLA2G2D	COQ8A	RGS4	ECM1	EMCN	VEGFD	PCP4L1	CLU	OSR2	CDKN1C	P2RY14	AGTR2
**type ii pneumocyte**	SFTPA1	SFTPA2	ROS1	SFTPC	SLC22A31	SFTPB	SFTPD	LAMP3	ALOX15B	AGBL1	PEBP4	SLC26A9	SFTA2	C4BPA	SLC34A2	CHIA	SERPINA2	ERFL	ZNF385B	ATP13A4	CLDN18	NKX2-1	OR2L5	DEFB4B	WFDC10B	SLC46A2	PGC	LRRK2	PLA2G4F	SCTR	GGTLC1	SLC9C1	AGER	RBPJL	SCGB3A2	GKN2	PLA2G1B	SDR16C5	DUOXA1	CAPN8	ARHGEF38	UBE2L5	LGI3	OR7G2	SLC6A14	SLCO4C1	TMEM163	DUOX1	LMO3	ESYT3
**type-6 epithelial cell of thymus**	OBP2A	OBP2B	ITPRID1	BHLHA15	ATP13A5	KLHDC7A	PAX7	RTP2	KLK14	LTF	SLC10A5	DMBX1	DMBT1	RTP1	SFTPD	CNGA2	DMRT3	ELF5	CNGA4	GSTA3	MANSC4	RASSF10	ENPP3	CABS1	CXCL17	GLYATL3	UMODL1	KRT23	SLC39A2	TFCP2L1	PAX3	SLC5A8	SOWAHB	TEX15	TAC1	SCGB1C1	CLIC6	ILDR1	SMR3A	FEZF1	SLC5A9	FADS6	LRRC26	SPINK14	OTX1	EHF	S100A5	PIP	ADH1A	BARX2
**unipolar neuron**	LMX1A	NWD2	TLL1	TRPC3	BARHL1	ZNF804A	CBLN1	ST6GAL2	SEPTIN3	STUM	ZNF804B	NDST4	STXBP5L	VWC2	XKR7	ZNF536	LRP1B	GSG1L	GALNTL6	TRPC5	DSCAML1	KIRREL3	TAFA2	DISP3	CNPY1	ZNF385B	CCDC175	UNC5D	LINGO2	GRM8	LRRTM4	NLGN1	CRACD	DRAXIN	PVALEF	EOMES	ZIC4	EN2	PTPRO	KHDRBS2	OR2L13	RPH3A	CHRM5	UNC5C	FSTL4	ZIC1	RGS7	SLC35F3	CNKSR2	CNTN5
**unknown**	OR11H2	LY6L	MBD3L3	PPIAL4D	PRAMEF26	OR10K2	OR2J3	H4C13	MYH1	KRTAP5-4	H2BC3	OR10A3	SCGB2A2	MS4A18	KRTAP12-1	OR2C3	EFCAB8	MRGPRX2	FCRL4	SCGB1C2	SDR9C7	H2AC4	TAAR5	OCM2	CHRNE	DEFA5	CYP2C9	EPO	OR8J1	DEFB107B	OR2T1	H2BC14	MMP8	OR7D4	TPSD1	KRTAP11-1	MCEMP1	SCN4A	P2RY12	GOT1L1	CCL27	PGLYRP3	NTSR2	SLC35G3	NBPF4	CCL1	H2AC13	HNRNPCL1	KRTAP21-2	OR4K2
**unswitched memory b cell**	CLEC20A	CLEC17A	LCN10	FCRL2	MS4A1	FCRL3	FCER2	BLK	CALHM6	LILRA4	HLA-DOB	CD1C	TLR10	FCRL1	FCRLA	TLR9	BANK1	CD19	CD79A	NBPF26	FCRL5	RUBCNL	PAX5	OR2T2	ZNF860	P2RX5	TNFRSF13B	FCMR	CD22	HLA-DQA1	PARP15	GRAPL	PNOC	SIGLEC6	SIGLEC10	RIPOR2	TNFRSF13C	CD79B	CXCR5	SPIB	SCIMP	HLA-DOA	HLA-DQB1	TRIM34	GAPT	FCRL4	POU2AF1	TMEM273	CD180	NCR3
**upciscc131**	KRT6C	PTTG2	ALPP	SERPINB13	AMTN	KRT13	TP63	GSTT2B	CALML3	RAET1L	NMU	KRT14	CALHM3	KRT9	SPRR1B	KRT17	KRT6A	GAST	KLK5	KLK10	ANXA8L1	KRT16	RNF212	CSTA	RPTN	ANP32D	KRT5	HCAR2	CA2	CFAP251	HES2	PLCXD2	LRRC69	GREM1	SERPINB5	GPR87	OLR1	MFAP5	ZNF114	SUN3	KCNJ3	LCE1B	FGFBP1	TMEM52B	TGM1	CBLC	ZBED2	PROM2	HOXD11	LYPD5
**ureteric bud**	PRR35	AQP2	ADH1C	ELF5	KRTAP2-2	SCGB1C2	UGT3A1	REN	EMX1	EMX2	WNT9B	AOC1	PTGER1	PAX2	SCNN1G	DOC2B	UPK1A	LHX1	CDH16	MCCD1	SBSPON	DCDC2	CLDN8	SCUBE3	SCIN	CLDN19	BLACAT1	PLA2G4F	KIF12	KCNJ15	DHRS2	ZNF44	NHLRC4	POU3F4	UPK2	TBC1D3D	OVCH2	CAPS	CLPSL1	HOXD8	FAM3B	MST1	HOXD11	PAX8	HOXB6	MMP7	CRTAC1	TMEM52B	LEAP2	MAL
**urothelial cell**	SNX31	UPK1A	TGM4	GSDMA	UPK3A	UPK2	IQCM	MS4A5	TTC6	STYK1	BSPH1	GPC5	SYTL5	BMP3	CPA2	CRYBG1	TEPP	NMBR	CCDC83	OR2T10	LIPK	HEMGN	NAALADL2	CAPN13	GLB1L3	GPR39	ALAS2	HTR4	PDE8B	MAGEB18	CATSPER4	ANKFN1	GRIP1	NPS	MROH5	COL4A6	LINGO4	APOF	MALRD1	SMIM9	SEPTIN14	CFTR	FYB2	KCNRG	PSCA	AFM	MEF2B	SLC25A48	PF4	PGLYRP3
**uvm**	HTN1	HTR2B	ABCB5	TYRP1	MLANA	BIRC7	CA14	TSPAN10	OR7A5	CRTAC1	TRPM1	CAPN3	TMEM200C	PAX3	TYR	PMEL	ALX1	STK32A	PNMA6A	CITED1	GAPDHS	COL11A2	ABCC11	LRRN4CL	GPR143	KRTAP9-2	ITIH6	AJM1	KCNJ13	HHATL	SSX5	TRIM63	TRIM51	CDH19	CYSLTR2	LY6L	SGCD	ZNF280A	OCA2	HSD17B14	KRTAP5-2	SPATA31A1	ZNF749	LY96	HSF4	PNMA3	MTLN	CTSK	IRX6	CFAP410
**uwb1289**	DHRS2	NKX2-8	FGF18	CLDN16	WNT7A	ERP27	SMCO2	PAX8	ITGB6	ZBED2	UGT2B7	TMEM139	RNF224	TMEM125	CDKN2A	KRTAP2-3	MROH6	EMX2	CDH6	AQP5	TRPM8	VGLL1	RHOD	SYNE4	CDH15	NXPH4	CBS	ZNF48	TMSB15B	TLX3	MAGEA11	RAC3	TM7SF2	LAMC2	MYEOV	JSRP1	LMO3	PIP5KL1	NPW	BICDL2	ERVMER34-1	CRB3	BSPRY	DLX6	TACSTD2	IGFBP3	ZNF853	MAGEA3	ARHGEF35	UPK2
**valve cell**	COLEC11	KCNJ8	HIGD1B	TRARG1	TRPC3	ADRA2B	CASQ2	EPHX3	NPY4R	REM1	ABCC9	RASL12	HEYL	FOXS1	GJA4	TBX20	PLA1A	ART3	STEAP4	COX4I2	VTN	ITGA7	RTN4RL2	RGS5	ASPN	CSPG4	IRAG1	ECM2	ADAP2	IL34	RGS4	P2RY14	S1PR3	NAALAD2	MRAP	ART4	PDGFRB	PLXDC1	ADRA1D	NDUFA4L2	HRCT1	GPR182	ANGPTL2	KBTBD13	AOC3	C1QTNF2	KCNMB1	RERG	ADORA2A	SLC2A4
**vasa recta ascending limb cell**	KRTAP5-3	CCL14	CCL23	TM4SF18	RAMP3	DNASE1L3	CETP	SELE	NUTM2E	FAM167B	SPAAR	SELP	RBP5	FCN2	FCN3	PLVAP	EXOC3L2	RNASE1	NOTCH4	ROBO4	PCDH12	NOSTRIN	ANP32D	EMCN	CLEC14A	TMEM88	MMRN2	ANKRD20A1	LRRC32	CRHBP	GALNT15	ADCY4	TEK	ADGRL4	ZNF366	CNTNAP3B	SYT15	GRAPL	A2M	PTPRB	CAVIN2	SLC51B	GDF3	F2RL3	SLC2A14	CYP4A22	TIE1	CEACAM1	SLCO2A1	HRCT1
**vasa recta descending limb cell**	OR2T10	FCN3	SEMA3G	SSUH2	NOTCH4	CRHBP	ZNF366	RBP7	LCN6	ADCY4	TMEM78	NUTM2E	SOX17	BTNL9	CLDN5	SLC14A1	A2M	FAM107A	CLEC14A	RNASE1	KCNN3	GLYATL2	HSPA12B	SLC5A4	ADGRL4	GPIHBP1	APOL3	MMRN2	AQP1	GJA5	TIE1	PALMD	RAMP2	F2RL3	ADGRF5	BCL6B	TM4SF18	GOLGA8R	PECAM1	PTPRB	ADM5	TDRD10	ITGA10	SOX18	HIF3A	LRRC32	CCM2L	FRG2C	SPAAR	EMCN
**vascular associated smooth muscle cell**	OR2A12	SMIM41	OR51E1	FDCSP	SLC5A4	SLC22A25	FCN3	PRSS48	CCDC102B	SLC22A24	BTNL8	GOLGA8O	TNFRSF6B	IRAG1	GGTLC3	LMOD1	PALM2AKAP2	GOLGA6B	RAB40A	TRPC6	AOC3	ANO2	EGFL6	NDUFA4L2	WFDC1	WFDC10B	TEX52	TBX2	AVPR1A	FHL5	FMO3	ERICH4	RERGL	SYNPO2	CCN4	RASL12	FAM162B	KCNE4	DIPK2B	MCAM	OR5AK2	CACNA1C	SLC38A11	FOXS1	GASK1A	COL14A1	FRZB	MROH9	VWF	SLIT3
**vascular leptomeningeal cell**	OR6C65	SLC22A6	OR6V1	SLC6A13	SERPIND1	FAM180A	SLC22A8	SLC13A4	ITIH2	EDN3	TBX18	GJB6	ZIC4	LAMC3	CFHR2	APOD	RXFP3	ATP1A2	VTN	ABCA9	RANBP3L	ISLR	ATP13A5	SMCO3	COL4A6	OR8K1	ZIC1	ITIH5	SLC38A3	WFIKKN2	SLC6A12	SLC5A5	EPHA8	LAMP5	LAMA1	KCNK2	SLC30A10	OR13D1	LAMA2	MC5R	CPED1	TMEM132C	CALHM1	FMOD	ALX3	KCNJ13	ALDH1A2	OGN	CPZ	CEMIP
**vein endothelial cell**	SLC5A4	DIPK2B	ANO2	OR2A14	VWF	SELE	SNTG2	FCN3	CC2D2B	NFILZ	NR5A2	TLL1	SHANK3	PALM2AKAP2	ZNF385D	LCN10	SHOC1	MYRIP	ESR2	CCDC200	ADAMTS9	TDRD10	SLC22A10	ERG	BMERB1	MCTP1	ERFL	TEX52	GNA14	LDB2	OR2A42	TACR1	ERICH4	ABCB1	SLCO2A1	CALCRL	NUTM2B	RASGRF2	SH3RF3	ZNF366	DEFB131B	FAM241A	DUXB	INSYN2A	ZNF423	ADGRL4	ADCY4	PREX2	ST6GALNAC3	PRSS48
**ventricular cardiac muscle cell**	LRRC10	MYL2	KCNG2	MYBPC3	MYL7	MYH6	MYH7	MYL3	CSRP3	TNNT2	SMYD1	MYL4	ABRA	POPDC2	NPPA	TNNI1	NKX2-5	MYL1	FBXL22	HSPB7	MYLK3	IRX4	CASQ1	TNNC1	SH3BGR	SYNPO2L	TBX20	APOBEC2	PPP1R17	DUSP29	XIRP1	TECRL	MYBPHL	ASB12	SMPX	TTN	HSPB2	HRC	PTGES3L	UNC45B	TRIM63	TNNI3	TRDN	FITM1	TBX5	ASB2	CCDC141	PGAM2	SPIN2A	NPPB
**vip gabaergic cortical interneuron**	VIP	ZIM3	CRH	CBLN2	ADRA1B	MRGPRX2	GRPR	VWC2L	NPY2R	RGS8	GPR62	SLC32A1	ADARB2	UTS2R	CNTN6	KCNK18	MAGEB18	GLRA2	SLC5A7	SORCS3	HTR3A	SPACA5	TCTE1	PCDH8	GALNTL6	PLA2G4E	SLC35F4	SYNPR	CNR1	NPAS1	KRT85	NPY5R	ASIC4	VSTM2A	SPATS1	GDF1	CHRNA4	HTR2C	HS6ST3	HRH3	ELFN1	FRMPD4	GRM8	LRFN2	HTR6	KCNIP1	GABRG3	CCDC38	CTCFL	SERPINA12
**visual system neuron**	FOXN4	VSX1	NPBWR2	RAX2	FGF6	TAS2R16	KRTAP12-2	TKTL2	OR13H1	BHLHE22	CCNB3	SDS	RAX	NEUROD4	KRTAP16-1	TMEM221	GPHB5	SOX1	TUBA3C	TEX49	EXOC1L	FTMT	ETV3L	MC3R	NEUROG1	TTC34	MAB21L1	DSPP	OR52M1	MYT1	ZP4	OR6X1	NEUROD1	GPR101	PLPPR3	NMRK2	GLYATL1B	ITLN2	IL17REL	TLDC2	SLC2A2	TAFA3	SIX6	MSI1	TAS2R9	AWAT1	TMEM150B	DLL1	AADACL4	MTNR1A
**vmrcrcz**	IGFN1	UGT2B7	SLC17A3	C1QL1	AK5	TM4SF18	CD70	SIGLEC15	FIBCD1	NXPH4	TINAG	HNF1B	BHMT2	ALPK2	GALNT9	DCDC2	SEC14L6	JCAD	GALNT14	CD163L1	SPINK13	TLCD4	HS3ST3A1	PDZK1IP1	TRNP1	ADORA1	GPRIN2	PYDC2	LRRC61	FXYD2	SLFN13	BATF3	EVA1A	PXDNL	FGF2	TGFA	PAX8	MORC4	MNX1	TMEM200B	C1QL4	PEG10	IGFBP3	ELFN2	ERFE	AMIGO2	PAX2	MET	OGFRL1	ZP1
**white fat cell**	OTOP1	UCP1	PLIN1	ADIG	ADIPOQ	CIDEC	ADRB3	PLIN4	SLC36A2	LGALS12	RETN	MRAP	CYP2E1	THRSP	TRARG1	CIDEA	AMY1C	PCK1	HCAR1	GYS2	ANGPTL8	ORM1	GPD1	AOC3	CDSN	FABP4	PON1	LIPE	SLC7A10	NRG4	PAQR9	B3GALT2	CFD	ABCD2	DGAT2	STEAP4	IGFALS	TMEM45B	SLC2A4	LPL	KCNK3	FMO1	AQP7	GHR	ANKEF1	CXCL9	ATP1A2	CDO1	PNPLA3	HCAR2
**xanthophore cell**	BCO1	TAT	GRIFIN	OPN5	PON1	SULT6B1	RHAG	SLC45A2	LIPF	AGMO	PRLH	TMEM130	GPR143	PRR33	WBP2NL	TNFAIP8L3	CDK15	FOXD3	NCCRP1	ALDOB	ANXA13	TPTE	CHP2	TSPAN10	TFAP2E	FOXO3B	SMIM29	RAB41	KRT4	EIF4EBP3	PHYHD1	COX6B2	BCL2L10	PRTFDC1	GAPDHS	COX4I2	PSMA8	SGCG	CREG2	SDCBP2	ARHGEF33	DCT	FABP3	SOX10	EXTL1	ANGPTL5	ATG9B	FABP2	TMEM19	TTPA
**yd38**	PADI3	IL24	SOX15	RNASE7	CFAP251	MAGEA3	PRCD	TMEM40	MAGEA4	ARTN	MROH6	LY6K	PTHLH	STEAP1B	RGS20	ZBED2	S100A2	KRT5	VGLL1	GPR87	CST6	FAM83A	SYT8	IL20RB	MIB2	GTSF1	MAGEA6	DLK2	CSTA	FOXL2	SAA1	FST	TEAD3	HES2	RHOD	GJB5	KRT6A	CDA	MT1E	SLC38A5	MMP24OS	COL17A1	PSORS1C1	PHF19	WDR72	GJB3	WNT7A	GNA15	KHDC1L	SFN
**yolk sac hematopoietic stem cell**	NCR2	DCANP1	DEFA4	OR2D3	SIGLEC14	OR5M3	OR2T8	KCNK17	SIGLEC12	LILRB5	TLR10	CEMP1	SIGLEC5	LYVE1	OR8D4	RNASE3	TIMD4	TMIGD3	PGA4	ADGRG5	GCSAML	PRG2	VSIG4	CRYBB1	SLC22A16	GPBAR1	BPI	OR2T33	RETN	CETP	HBD	AZU1	CYTL1	MPL	NBPF8	SELP	TAS2R20	PRAM1	FOXO3B	GP9	UGT2B28	SIGLEC16	RXFP1	SIGLEC11	AIF1	RNASE2	FCGR2C	CD33	GPR34	SMIM24

Supplementary Table 4. Human/Mouse Gene Expression Correlation By Cell Type

celltype	rsquared	pearsonr	pvalue
mesenchymal cell	0.766547139	0.875526778	0
fibroblast	0.741143568	0.860896956	0
smooth muscle cell	0.718319375	0.847537241	0
myeloid leukocyte	0.717259219	0.846911577	0
stromal cell	0.712580435	0.844144795	0
alpha-beta t cell	0.699252546	0.836213218	0
endothelial cell	0.697971623	0.83544696	0
neural progenitor cell	0.692733897	0.832306372	0
cd4-positive, alpha-beta t cell	0.691272678	0.831428095	0
embryonic stem cell	0.686942848	0.828820154	0
mature nk t cell	0.680472306	0.824907453	0
intestinal crypt stem cell	0.678931847	0.823973207	0
myeloid cell	0.678338536	0.823613099	0
cd8-positive, alpha-beta t cell	0.674044306	0.821002013	0
renal vesicle	0.668384091	0.817547608	0
keratinocyte	0.662700289	0.814064057	0
mesothelial cell	0.659277151	0.811958836	0
radial glial cell	0.659148773	0.811879777	0
macrophage	0.657978611	0.811158808	0
alternatively activated macrophage	0.657877392	0.811096413	0
endodermal cell	0.657799648	0.811048487	0
pluripotent stem cell	0.656262108	0.810100061	0
b cell	0.654036381	0.808725158	0
neuron	0.653101907	0.808147206	0
natural killer cell	0.651541603	0.80718127	0
dendritic cell	0.651468788	0.807136165	0
basal cell	0.650220674	0.80636262	0
melanocyte	0.649867648	0.80614369	0
exhausted t cell	0.646896228	0.804298594	0
nephron progenitor	0.645476647	0.803415613	0
chondrocyte	0.641348301	0.800842245	0
gabaergic neuron	0.63827854	0.798923363	0
oligodendrocyte precursor cell	0.636584782	0.797862633	0
endothelial cell of lymphatic vessel	0.635966953	0.797475362	0
innate lymphoid cell	0.634463883	0.796532412	0
hematopoietic stem cell	0.634387026	0.796484165	0
central memory cd8-positive, alpha-beta t cell	0.63311108	0.795682776	0
neutrophil	0.632429759	0.795254524	0
naive thymus-derived cd8-positive, alpha-beta t cell	0.625898645	0.791137564	0
progenitor cell	0.621727103	0.788496736	0
oligodendrocyte	0.616481143	0.785163132	0
cd14-positive monocyte	0.615737095	0.784689171	0
erythroid progenitor cell	0.614480925	0.783888337	0
t cell	0.614175134	0.783693265	0
epithelial cell of large intestine	0.613529517	0.78328125	0
naive thymus-derived cd4-positive, alpha-beta t cell	0.61334463	0.78316322	0
neural crest cell	0.612878412	0.782865514	0
epithelial cell	0.612392419	0.782555058	0
intestinal epithelial cell	0.610000884	0.781025533	0

sertoli cell	0.60632176	0.778666655	0
enteroendocrine cell	0.606138059	0.778548687	0
cardiac muscle cell	0.601644405	0.775657402	0
plasmacytoid dendritic cell	0.59968975	0.774396378	0
myofibroblast cell	0.595721363	0.77182988	0
spermatogonium	0.594721082	0.771181614	0
regulatory t cell	0.592573567	0.769788001	0
glomerular visceral epithelial cell	0.591104516	0.768833217	0
granulocyte monocyte progenitor cell	0.588711137	0.767275138	0
monocyte	0.588645837	0.767232583	0
glutamatergic neuron	0.58822128	0.766955853	0
retinal ganglion cell	0.586097782	0.765570233	0
plasma cell	0.585985977	0.765497209	0
brca	0.585753585	0.765345403	0
astrocyte	0.585575787	0.765229238	0
kidney interstitial fibroblast	0.582705159	0.763351268	0
gonadtroph	0.581730697	0.762712723	0
erythroid lineage cell	0.58171026	0.762699325	0
leukocyte	0.571141801	0.755739241	0
cd4-positive, cd25-positive, alpha-beta regulatory t cell	0.569969244	0.754963074	0
suppressor macrophage	0.568340729	0.753883764	0
effector cd8-positive, alpha-beta t cell	0.568274984	0.753840158	0
ciliated cell	0.566189357	0.752455552	0
mesodermal cell	0.564015391	0.751009581	0
enterocyte	0.563387244	0.750591263	0
somatotroph	0.563156095	0.750437269	0
intermediate monocyte	0.561829094	0.749552596	0
megakaryocyte-erythroid progenitor cell	0.561419898	0.749279586	0
type b pancreatic cell	0.561108862	0.749072001	0
gamma-delta t cell	0.55670387	0.746125907	0
lung macrophage	0.556634876	0.746079671	0
immature b cell	0.551320762	0.742509772	0
pancreatic a cell	0.551116804	0.742372416	0
cholangiocyte	0.547830288	0.740155583	0
preadipocyte	0.546147162	0.739017701	0
luminal epithelial cell of mammary gland	0.544108777	0.737637294	0
corticotroph	0.543383392	0.737145435	0
basal cell of epidermis	0.540842022	0.735419623	0
mammotroph	0.539844951	0.734741418	0
hematopoietic precursor cell	0.539239506	0.73432929	0
t-helper 1 cell	0.53793749	0.73344222	0
inflammatory macrophage	0.535899295	0.732051429	0
common lymphoid progenitor	0.535842913	0.732012919	0
classical monocyte	0.535160213	0.731546453	0
kidney loop of henle thick ascending limb epithelial cell	0.534655971	0.731201731	0
cd4-positive, alpha-beta memory t cell	0.53385235	0.730652003	0
common myeloid progenitor	0.530915887	0.728639751	0
retinal progenitor cell	0.530544342	0.728384749	0
hematopoietic cell	0.53035166	0.72825247	0
nucleate erythrocyte	0.530154493	0.728117087	0
schwann cell	0.52865766	0.727088481	0
primordial germ cell	0.528248097	0.726806781	0

microglial cell	0.527881288	0.726554394	0
pro-b cell	0.527847436	0.726531097	0
kupffer cell	0.526552265	0.725639211	0
lymphocyte	0.525827652	0.725139747	0
naive b cell	0.524790564	0.724424299	0
hepatocyte	0.521191559	0.72193598	0
mesangial cell	0.520535174	0.721481236	0
pericyte cell	0.519992409	0.721104992	0
glial cell	0.519176249	0.72053886	0
megakaryocyte progenitor cell	0.518880184	0.720333384	0
mature b cell	0.517745876	0.719545604	0
keratinocyte stem cell	0.515328785	0.717864044	0
hematopoietic oligopotent progenitor cell	0.515170137	0.717753535	0
mb	0.514013949	0.716947661	0
precursor b cell	0.512599493	0.715960539	0
kidney loop of henle epithelial cell	0.510524949	0.714510286	0
mural cell	0.508245587	0.71291345	0
myoblast	0.506965455	0.712015067	0
double negative thymocyte	0.50445955	0.710253159	0
non-classical monocyte	0.503923001	0.709875342	0
lung neuroendocrine cell	0.496235341	0.704439736	0
epithelial cell of lung	0.490464263	0.700331538	0
enterocyte of epithelium of large intestine	0.487572398	0.698263846	0
smooth muscle	0.483241174	0.695155504	0
leydig cell	0.482514453	0.694632603	0
kidney collecting duct principal cell	0.482342144	0.694508563	0
granulocyte	0.481766551	0.69409405	0
spermatocyte	0.481358523	0.69380006	0
memory b cell	0.48118548	0.693675342	0
skin fibroblast	0.479691749	0.692597827	0
mast cell	0.478261655	0.691564643	0
pancreatic ductal cell	0.476607382	0.69036757	0
group 1 innate lymphoid cell	0.475788569	0.689774289	0
hematopoietic multipotent progenitor cell	0.475279323	0.68940505	0
cd14-low, cd16-positive monocyte	0.47412168	0.688564942	0
pdac	0.460495807	0.678598413	0
basophil	0.460368801	0.678504827	0
erythrocyte	0.46033299	0.678478437	0
megakaryocyte	0.459302238	0.677718406	0
ovarian surface epithelial cell	0.45806238	0.676803059	0
granulosa cell	0.449802378	0.670673079	0
secretory cell	0.449277029	0.670281306	0
epithelial cell of proximal tubule	0.448651917	0.669814838	0
vascular associated smooth muscle cell	0.448520543	0.669716763	0
mesenchymal stem cell	0.446360841	0.668102418	0
renal alpha-intercalated cell	0.442170341	0.664958902	0
tissue-resident macrophage	0.44112903	0.664175451	0
goblet cell	0.438561418	0.662239699	0
group 2 innate lymphoid cell	0.437389986	0.66135466	0
cd14-positive, cd16-positive monocyte	0.434807629	0.659399446	0
skcm	0.430116289	0.655832516	0
enteric neuron	0.429456022	0.655328941	0

luminal cell of prostate epithelium	0.428259515	0.6544154	0
hepatic stellate cell	0.425671113	0.652434758	0
retinal rod cell	0.414708641	0.643978758	0
renal beta-intercalated cell	0.414679993	0.643956515	0
muscle precursor cell	0.413332752	0.642909598	0
laml	0.413063964	0.642700525	0
fibroblast of connective tissue of prostate	0.410287024	0.640536513	0
pancreatic acinar cell	0.407553096	0.638398853	0
interneuron	0.406224776	0.637357651	0
chondroblast	0.405933563	0.637129158	0
promonocyte	0.402515847	0.634441366	0
pre-conventional dendritic cell	0.39539089	0.628801153	0
retinal cone cell	0.382402539	0.618387046	0
epithelial cell of prostate	0.379131172	0.615736284	0
muscle cell	0.379038871	0.615661328	0
cd8-positive, alpha-beta memory t cell	0.37515491	0.612498906	0
type ii pneumocyte	0.373678729	0.61129267	0
neuronal stem cell	0.366599149	0.605474317	0
pancreatic pp cell	0.365513852	0.604577416	0
kidney loop of henle descending limb epithelial cell	0.363080101	0.602561284	0
spermatid	0.361739064	0.601447474	0
kidney loop of henle ascending limb epithelial cell	0.353281484	0.594374868	0
pancreatic endocrine cell	0.35321626	0.594319998	0
effector cd4-positive, alpha-beta t cell	0.348291969	0.590162664	0
pancreatic stellate cell	0.342476591	0.585214996	0
transitional stage b cell	0.340962433	0.583919886	0
naive t cell	0.340879612	0.583848963	0
hair follicle cell	0.338981825	0.582221457	0
sperm	0.33776958	0.581179473	0
renal intercalated cell	0.332786689	0.576876667	0
osteoblast	0.32753548	0.572307156	0
club cell	0.32560281	0.570616167	0
smooth muscle cell of prostate	0.325030513	0.570114474	0
platelet	0.323484412	0.568756901	0
l2/3-6 intratelencephalic projecting glutamatergic cortica	0.32099042	0.566560164	0
early lymphoid progenitor	0.312531976	0.559045594	0
paneth cell	0.305000934	0.552268897	0
erythroblast	0.303612	0.551009981	0
pancreatic d cell	0.301330329	0.548935632	0
kidney connecting tubule epithelial cell	0.296216324	0.54425759	0
type i pneumocyte	0.281604874	0.530664558	0
common dendritic progenitor	0.276512747	0.525844794	0
amacrine cell	0.275419772	0.524804508	0
germinal center b cell	0.269532354	0.519165055	0
thymocyte	0.262847899	0.512686941	0
fibroblast of lung	0.256865582	0.506819082	0
neurecto-epithelial cell	0.252372128	0.502366528	0
central nervous system neuron	0.249715959	0.499715878	0
inhibitory neuron	0.235222509	0.484997432	0
lung ciliated cell	0.232133459	0.481802303	0
macroglial cell	0.226045835	0.475442778	0
capillary endothelial cell	0.222016421	0.471186185	0

urothelial cell	0.220333287	0.469396727	0
vein endothelial cell	0.211534529	0.45992883	0
endothelial cell of vascular tree	0.205868791	0.453727662	0
cerebral cortex gabaergic interneuron	0.205611704	0.453444268	0
epithelial cell of stomach	0.201965466	0.449405681	0
endothelial cell of artery	0.19869768	0.445755179	0
mucus secreting cell	0.179732863	0.423949128	0
bladder urothelial cell	0.178767349	0.42280888	0
skeletal muscle satellite stem cell	0.169719741	0.411970558	0
fat cell	0.163537428	0.404397611	0
group 3 innate lymphoid cell	0.161303979	0.401626667	0
alveolar macrophage	0.160567296	0.400708493	0
mucosal invariant t cell	0.14480799	0.38053645	0
kidney distal convoluted tubule epithelial cell	0.127341559	0.356849491	0
tendon cell	0.119279403	0.345368504	0
excitatory neuron	0.101230444	0.31816732	0
tracheobronchial smooth muscle cell	0.100529688	0.31706417	0
neural cell	0.091485335	0.302465429	0
medullary thymic epithelial cell	0.085043086	0.291621478	0
respiratory basal cell	0.07803871	0.279354094	0
lung endothelial cell	0.077591469	0.278552453	0
brush cell	0.075365163	0.274527163	0
connective tissue cell	0.071271907	0.266967989	0
acinar cell	0.064041459	0.25306414	0
mueller cell	0.064017856	0.253017502	0
central nervous system macrophage	0.058101365	0.241042248	0
immature natural killer cell	0.042973699	0.207300986	1.09E-277
muller cell	0.035731414	0.189027548	2.15E-230
epiblast cell	0.000546809	0.023383941	7.09E-05
trophectodermal cell	0.000350932	0.018733177	0.001457763

Supplementary Table 5. Studies used for building training dataset.

accession	PMID	Date	Title
E-HCAD-18	30796046	2/24/19	Maturation Of Heart Valve Cell Populations During Postnatal Remodeling.
E-MTAB-10026	33879890	4/20/21	Deciphering the molecular immune response to COVID-19 using single cell multi-omics
E-MTAB-10197	29752062	5/13/18	Single-Cell Rna Sequencing Of Lymph Node Stromal Cells Reveals Niche-Associated Heterogeneity.
E-MTAB-2983	26444631	10/9/15	Adult Human And Mouse Ovaries Lack Ddx4-Expressing Functional Oogonial Stem Cells.
E-MTAB-3857	26950746	3/8/16	T Cell Fate And Clonality Inference From Single-Cell Transcriptomes.
E-MTAB-3929	27062923	4/12/16	Single-Cell RNA-Seq Reveals Lineage and X Chromosome Dynamics in Human Preimplantation Embryos
E-MTAB-4547	28479188	5/10/17	Vitamin A-Retinoic Acid Signaling Regulates Hematopoietic Stem Cell Dormancy.
E-MTAB-4850	26860370	2/11/16	Linking The T Cell Receptor To The Single Cell Transcriptome In Antigen-Specific Human T Cells.
E-MTAB-5485	31092921	5/17/19	Tracing The Origin Of Adult Intestinal Stem Cells.
E-MTAB-5553	28522527	5/20/17	Lineage-Dependent Spatial And Functional Organization Of The Mammalian Enteric Nervous System.
E-MTAB-5661	28652613	6/28/17	Flipping Between Polycomb Repressed And Active Transcriptional States Introduces Noise In Gene Expression.
E-MTAB-5727	29330484	1/14/18	Single-Cell Rna-Sequencing Resolves Self-Antigen Expression During Mtec Development.
E-MTAB-5802	29925944	6/22/18	A Stromal Cell Population That Inhibits Adipogenesis In Mammalian Fat Depots.
E-MTAB-6031	32178760	3/18/20	Coordinated Hedgehog Signaling Induces New Hair Follicles In Adult Skin.
E-MTAB-6051	30013148	7/18/18	T Cell Cytolytic Capacity Is Independent Of Initial Stimulation Strength.
E-MTAB-6058	29153988	11/21/17	Isolation And Comparative Transcriptome Analysis Of Human Fetal And Ipsc-Derived Cone Photoreceptor Cells.
E-MTAB-6077	30478328	11/28/18	Heart Enhancers With Deeply Conserved Regulatory Activity Are Established Early In Zebrafish Development.
E-MTAB-6108	29437159	2/14/18	Single Cell Rna Sequencing Of Stem Cell-Derived Retinal Ganglion Cells.
E-MTAB-6142	29045817	10/19/17	Transcriptomic Characterization Of The Human Cell Cycle In Individual Unsynchronized Cells.
E-MTAB-6149	29988129	7/11/18	Phenotype Molding Of Stromal Cells In The Lung Tumor Microenvironment.
E-MTAB-6153	29311656	1/10/18	Defining Murine Organogenesis At Single-Cell Resolution Reveals A Role For The Leukotriene Pathway In Regulating Blood Progenitor Formation.
E-MTAB-6173	29346760	1/19/18	Single-Cell Transcriptional Profiling Reveals Cellular Diversity And Intercommunication In The Mouse Heart.
E-MTAB-6308	31935371	1/15/20	An Integrated Gene Expression Landscape Profiling Approach To Identify Lung Tumor Endothelial Cell Heterogeneity And Angiogenic Candidates.
E-MTAB-6362	30602787	1/4/19	Genomic Encoding Of Transcriptional Burst Kinetics.
E-MTAB-6385	31657037	10/28/19	Biologically Indeterminate Yet Ordered Promiscuous Gene Expression In Single Medullary Thymic Epithelial Cells.
E-MTAB-6386	29659703	4/17/18	B-Cell Receptor Reconstruction From Single-Cell Rna-Seq With Vdjpuzzle.
E-MTAB-6487	30042420	7/26/18	Interferon Gene Therapy Reprograms The Leukemia Microenvironment Inducing Protective Immunity To Multiple Tumor Antigens.
E-MTAB-6524	30267684	9/30/18	Single-Cell Profiling Identifies Key Pathways Expressed By Ipscs Cultured In Different Commercial Media.
E-MTAB-6653	29988129	7/11/18	Phenotype Molding Of Stromal Cells In The Lung Tumor Microenvironment.
E-MTAB-6677	29925944	6/22/18	A Stromal Cell Population That Inhibits Adipogenesis In Mammalian Fat Depots.
E-MTAB-6678	30429548	11/16/18	Single-Cell Reconstruction Of The Early Maternal-Fetal Interface In Humans.
E-MTAB-6701	30429548	11/16/18	Single-Cell Reconstruction Of The Early Maternal-Fetal Interface In Humans.
E-MTAB-6818	29909970	6/19/18	Reversible De-Differentiation Of Mature White Adipocytes Into Preadipocyte-Like Precursors During Lactation.
E-MTAB-6819	30673604	1/24/19	Transcriptional Heterogeneity In Naive And Primed Human Pluripotent Stem Cells At Single-Cell Resolution.
E-MTAB-6879	31422913	8/20/19	Defining The Identity And Dynamics Of Adult Gastric Isthmus Stem Cells.
E-MTAB-6911	30581079	12/26/18	Identification Of Embryonic Neural Plate Border Stem Cells And Their Generation By Direct Reprogramming From Adult Human Blood Cells.
E-MTAB-6912	30581079	12/26/18	Identification Of Embryonic Neural Plate Border Stem Cells And Their Generation By Direct Reprogramming From Adult Human Blood Cells.
E-MTAB-6925	30581079	12/26/18	Identification Of Embryonic Neural Plate Border Stem Cells And Their Generation By Direct Reprogramming From Adult Human Blood Cells.
E-MTAB-6945	30443254	11/18/18	Single-Cell Analysis Identifies Thymic Maturation Delay In Growth-Restricted Neonatal Mice.
E-MTAB-6967	30787436	2/23/19	A Single-Cell Molecular Map Of Mouse Gastrulation And Early Organogenesis.
E-MTAB-6970	30787436	2/23/19	A Single-Cell Molecular Map Of Mouse Gastrulation And Early Organogenesis.
E-MTAB-6976	30173915	9/4/18	Self-Maintaining Gut Macrophages Are Essential For Intestinal Homeostasis.
E-MTAB-6987	31996681	1/31/20	Single-Cell Transcriptomics Identifies Cd44 As A Marker And Regulator Of Endothelial To Haematopoietic Transition.
E-MTAB-7008	30595546	1/1/19	Method To Synchronize Cell Cycle Of Human Pluripotent Stem Cells Without Affecting Their Fundamental Characteristics.
E-MTAB-7037	30446505	11/18/18	Single-Cell Transcriptional Analysis Reveals Ilc-Like Cells In Zebrafish.
E-MTAB-7051	30356220	10/26/18	Gene Expression Variability Across Cells And Species Shapes Innate Immunity.
E-MTAB-7052	30356220	10/26/18	Gene Expression Variability Across Cells And Species Shapes Innate Immunity.
E-MTAB-7094	30988302	4/17/19	Origin And Differentiation Trajectories Of Fibroblastic Reticular Cells In The Splenic White Pulp.
E-MTAB-7098	28479188	5/10/17	Vitamin A-Retinoic Acid Signaling Regulates Hematopoietic Stem Cell Dormancy.
E-MTAB-7117	30446505	11/18/18	Single-Cell Transcriptional Analysis Reveals Ilc-Like Cells In Zebrafish.
E-MTAB-7142	30643263	1/16/19	Reference-Based Analysis Of Lung Single-Cell Sequencing Reveals A Transitional Profibrotic Macrophage.
E-MTAB-7149	31141696	5/30/19	Single-Cell Transcriptional Profiling Of Aortic Endothelium Identifies A Hierarchy From Endovascular Progenitors To Differentiated Cells.
E-MTAB-7159	30446505	11/18/18	Single-Cell Transcriptional Analysis Reveals Ilc-Like Cells In Zebrafish.
E-MTAB-7249	32054838	2/15/20	A Living Biobank Of Ovarian Cancer Ex Vivo Models Reveals Profound Mitotic Heterogeneity.
E-MTAB-7303	27460926	7/28/16	Single-Cell Tcrseq: Paired Recovery Of Entire T-Cell Alpha And Beta Chain Transcripts In T-Cell Receptors From Single-Cell Rnaseq.
E-MTAB-7311	30737144	2/10/19	Single-Cell Transcriptomics Of Regulatory T Cells Reveals Trajectories Of Tissue Adaptation.
E-MTAB-7316	31436334	8/23/19	A Single-Cell Transcriptome Atlas Of The Adult Human Retina.
E-MTAB-7320	30846445	3/9/19	Single Cell Transcriptomics Reveals Spatial And Temporal Dynamics Of Gene Expression In The Developing Mouse Spinal Cord.
E-MTAB-7365	28263961	3/7/17	Power Analysis Of Single-Cell Rna-Sequencing Experiments.
E-MTAB-7376	30912746	3/26/19	Single-cell expression profiling reveals dynamic flux of cardiac stromal, vascular and immune cells in health and injury
E-MTAB-7381	30692988	1/30/19	Maturing Human Cd127+ Ccr7+ Pdl1+ Dendritic Cells Express Aire In The Absence Of Tissue Restricted Antigens.
E-MTAB-7407	31597962	10/11/19	Decoding Human Fetal Liver Haematopoiesis.
E-MTAB-7417	32433953	5/21/20	Single-Cell Rna Sequencing Reveals A Dynamic Stromal Niche That Supports Tumor Growth.
E-MTAB-7427	32433953	5/21/20	Single-Cell Rna Sequencing Reveals A Dynamic Stromal Niche That Supports Tumor Growth.
E-MTAB-7606	30778243	2/20/19	Human CD8+ T cell cross-reactivity across influenza A, B and C viruses
E-MTAB-7660	31092921	5/17/19	Tracing The Origin Of Adult Intestinal Stem Cells.
E-MTAB-7678	32614947	7/3/20	Single-Cell Analyses And Machine Learning Define Hematopoietic Progenitor And Hsc-Like Cells Derived From Human Pscs.
E-MTAB-7703	30988302	4/17/19	Origin And Differentiation Trajectories Of Fibroblastic Reticular Cells In The Splenic White Pulp.
E-MTAB-7895	32130914	3/5/20	Dynamic Interstitial Cell Response During Myocardial Infarction Predicts Resilience To Rupture In Genetically Diverse Mice.
E-MTAB-7901	31422912	8/20/19	Distinct Molecular Trajectories Converge To Induce Naive Pluripotency.
E-MTAB-7919	28652613	6/28/17	Flipping Between Polycomb Repressed And Active Transcriptional States Introduces Noise In Gene Expression.
E-MTAB-8007	32066951	2/19/20	Distinct Microbial And Immune Niches Of The Human Colon.
E-MTAB-8077	32059779	2/16/20	Single-Cell Transcriptome Atlas Of Murine Endothelial Cells.
E-MTAB-8142	33479125	1/23/21	Developmental Cell Programs Are Co-Opted In Inflammatory Skin Disease.
E-MTAB-8221	32109386	2/29/20	In¬†Vitro And In¬†Vivo Development Of The Human Airway At Single-Cell Resolution.
E-MTAB-8263	26950746	3/8/16	T Cell Fate And Clonality Inference From Single-Cell Transcriptomes.
E-MTAB-8474	32066951	2/19/20	Distinct Microbial And Immune Niches Of The Human Colon.
E-MTAB-8483	31699795	11/9/19	Single-Cell Rna Sequencing Reveals Stromal Evolution Into Lrrc15
E-MTAB-8495	33602855	2/20/21	Cholangiocyte Organoids Can Repair Bile Ducts After Transplantation In The Human Liver.
E-MTAB-8559	29311656	1/10/18	Defining Murine Organogenesis At Single-Cell Resolution Reveals A Role For The Leukotriene Pathway In Regulating Blood Progenitor Formation.
E-MTAB-8561	33116305	10/30/20	Snrna-Seq Reveals A Subpopulation Of Adipocytes That Regulates Thermogenesis.
E-MTAB-8581	32079746	2/23/20	A Cell Atlas Of Human Thymic Development Defines T Cell Repertoire Formation.
E-MTAB-8629	30356220	10/26/18	Gene Expression Variability Across Cells And Species Shapes Innate Immunity.
E-MTAB-8809	32317663	4/23/20	Exploiting Evolutionary Steering To Induce Collateral Drug Sensitivity In Cancer.
E-MTAB-8810	32795101	8/17/20	High-Resolution Transcriptomic Profiling Of The Heart During Chronic Stress Reveals Cellular Drivers Of Cardiac Fibrosis And Hypertrophy.
E-MTAB-8901	33290721	12/9/20	Single-Cell Sequencing Of Developing Human Gut Reveals Transcriptional Links To Childhood Crohn'S Disease.
E-MTAB-9024	33575589	2/13/21	Comparative Performance Of The Bgi And Illumina Sequencing Technology For Single-Cell Rna-Sequencing.
E-MTAB-9221	32810439	8/19/20	Elevated Calprotectin And Abnormal Myeloid Cell Subsets Discriminate Severe From Mild Covid-19.
E-MTAB-9268	27460926	7/28/16	Single-Cell Tcrseq: Paired Recovery Of Entire T-Cell Alpha And Beta Chain Transcripts In T-Cell Receptors From Single-Cell Rnaseq.
E-MTAB-9492	30846445	3/9/19	Single Cell Transcriptomics Reveals Spatial And Temporal Dynamics Of Gene Expression In The Developing Mouse Spinal Cord.
E-MTAB-9510	33586340	2/16/21	Myogenesis Modelled By Human Pluripotent Stem Cells: A Multi-Omic Study Of Duchenne Myopathy Early Onset.

E-MTAB-9969	30429548	11/16/18	Single-Cell Reconstruction Of The Early Maternal-Fetal Interface In Humans.
E105114	32246845	4/5/20	Sars-Cov-2 Receptor Ace2 And Tmprss2 Are Primarily Expressed In Bronchial Transient Secretory Cells.
EGAS00001002	31554641	9/27/19	The Phenotypes Of Proliferating Glioblastoma Cells Reside On A Single Axis Of Variation.
EGAS00001002	30355494	10/26/18	Transcriptional Programming Of Normal And Inflamed Human Epidermis At Single-Cell Resolution.
EGAS00001004	33208946	11/20/20	A Molecular Cell Atlas Of The Human Lung From Single-Cell Rna Sequencing.
EGAS00001004	32591762	6/28/20	Covid-19 Severity Correlates With Airway Epithelium-Immune Cell Interactions Identified By Single-Cell Analysis.
EGAS00001004	33361824	12/29/20	Hypertension Delays Viral Clearance And Exacerbates Airway Hyperinflammation In Patients With Covid-19.
ENCSR713GIS	32728245	7/31/20	The Changing Mouse Embryo Transcriptome At Whole Tissue And Single-Cell Resolution.
GSE100337	28666115	7/1/17	IfnŒ≥-Dependent Tissue-Immune Homeostasis Is Co-Opted In The Tumor Microenvironment.
GSE100426	30540934	12/13/18	Heterogeneous Responses Of Hematopoietic Stem Cells To Inflammatory Stimuli Are Altered With Age.
GSE100471	29371425	1/27/18	Defining The Earliest Step Of Cardiovascular Lineage Segregation By Single-Cell Rna-Seq.
GSE100597	28768204	8/3/17	Single-Cell Landscape Of Transcriptional Heterogeneity And Cell Fate Decisions During Mouse Early Gastrulation.
GSE100618	29167569	11/24/17	Single-Cell Analysis Reveals The Continuum Of Human Lympho-Myeloid Progenitor Cells.
GSE100861	29689192	4/25/18	Hippo Signaling Plays An Essential Role In Cell State Transitions During Cardiac Fibroblast Development.
GSE101334	28886383	9/9/17	Anatomically And Functionally Distinct Lung Mesenchymal Populations Marked By Lgr5 And Lgr6.
GSE101712	28899870	9/14/17	Mbd3/Nurd Controls Lymphoid Cell Fate And Inhibits Tumorigenesis By Repressing A B Cell Transcriptional Program.
GSE101901	30254269	9/27/18	Single Cell Molecular Alterations Reveal Target Cells And Pathways Of Concussive Brain Injury.
GSE101984	28957441	9/29/17	Diversity Amongst Trigeminal Neurons Revealed By High Throughput Single Cell Sequencing.
GSE102090	29706538	5/1/18	Hmgb2 Loss Upon Senescence Entry Disrupts Genomic Organization And Induces Ctcf Clustering Across Cell Types.
GSE102130	29674595	4/21/18	Developmental And Oncogenic Programs In H3K27M Gliomas Dissected By Single-Cell Rna-Seq.
GSE102455	29030486	10/17/17	Single-Cell Rna Sequencing Reveals Developmental Heterogeneity Among Early Lymphoid Progenitors.
GSE102475	29240790	12/15/17	Single-Cell Rna Sequencing Reveals Intrinsic And Extrinsic Regulatory Heterogeneity In Yeast Responding To Stress.
GSE102479	29487567	3/1/18	Regulatory Architecture Of The LŒ≤t2 Gonadotrope Cell Underlying The Response To Gonadotropin-Releasing Hormone.
GSE102596	29449449	2/17/18	Conserved And Divergent Features Of Mesenchymal Progenitor Cell Types Within The Cortical Nephrogenic Niche Of The Human And Mouse Kidney.
GSE102665	29728440	5/8/18	Peripheral PDGFRŒ± + gp38 + mesenchymal cells support the differentiation of fetal liver-derived ILC2
GSE102827	29230054	12/13/17	Single-Cell Analysis Of Experience-Dependent Transcriptomic States In The Mouse Visual Cortex.
GSE102934	29724792	5/5/18	Bigscale: An Analytical Framework For Big-Scale Single-Cell Data.
GSE102962	29483303	2/28/18	Single-Cell Rnaseq Reveals Seven Classes Of Colonic Sensory Neuron.
GSE103154	29802404	5/29/18	Tracing The Temporal-Spatial Transcriptome Landscapes Of The Human Fetal Digestive Tract Using Single-Cell Rna-Sequencing.
GSE103221	30772174	2/18/19	Resolving Cell Fate Decisions During Somatic Cell Reprogramming By Single-Cell Rna-Seq.
GSE103224	30041684	7/26/18	Single-Cell Transcriptome Analysis Of Lineage Diversity In High-Grade Glioma.
GSE103322	29198524	12/5/17	Single-Cell Transcriptomic Analysis Of Primary And Metastatic Tumor Ecosystems In Head And Neck Cancer.
GSE103334	29020624	10/12/17	Temporal Tracking Of Microglia Activation In Neurodegeneration At Single-Cell Resolution.
GSE103866	29315726	1/10/18	Single-Cell Analysis Reveals Cancer Stem Cell Heterogeneity In Hepatocellular Carcinoma.
GSE103867	29315726	1/10/18	Single-Cell Analysis Reveals Cancer Stem Cell Heterogeneity In Hepatocellular Carcinoma.
GSE103892	29466745	2/22/18	Massively Parallel Single Nucleus Transcriptional Profiling Defines Spinal Cord Neurons And Their Activity During Behavior.
GSE103918	29657097	4/17/18	Single-Cell Transcriptomic Profiling Of Pluripotent Stem Cell-Derived Scgb3A2+ Airway Epithelium.
GSE103919	29657097	4/17/18	Single-Cell Transcriptomic Profiling Of Pluripotent Stem Cell-Derived Scgb3A2+ Airway Epithelium.
GSE103983	29513653	3/8/18	Developmental Diversification Of Cortical Inhibitory Interneurons.
GSE104154	29590628	3/29/18	Single-Cell Deconvolution Of Fibroblast Heterogeneity In Mouse Pulmonary Fibrosis.
GSE104156	29513653	3/8/18	Developmental Diversification Of Cortical Inhibitory Interneurons.
GSE104157	29513653	3/8/18	Developmental Diversification Of Cortical Inhibitory Interneurons.
GSE104556	30204153	9/12/18	Single-Cell Rna Sequencing Of Adult Mouse Testes.
GSE104600	29309048	1/9/18	Early B Cell Changes Predict Autoimmunity Following Combination Immune Checkpoint Blockade.
GSE104995	30291229	10/7/18	Myelo-Lymphoid Lineage Restriction Occurs In The Human Haematopoietic Stem Cell Compartment Before Lymphoid-Primed Multipotent Progenitors.
GSE105054	28296635	3/16/17	Dynamics Of Embryonic Stem Cell Differentiation Inferred From Single-Cell Transcriptomics Show A Series Of Transitions Through Discrete Cell States.
GSE105451	30765193	2/16/19	Unravelling Intratumoral Heterogeneity Through High-Sensitivity Single-Cell Mutational Analysis And Parallel Rna Sequencing.
GSE106218	31558476	9/29/19	Alterations In The Transcriptional Programs Of Myeloma Cells And The Microenvironment During Extramedullary Progression Affect Proliferation And Immu
GSE106236	30250253	9/27/18	Discovery Of A Periosteal Stem Cell Mediating Intramembranous Bone Formation.
GSE106466	29700225	4/28/18	Single-Cell Reconstruction Of Developmental Trajectories During Zebrafish Embryogenesis.
GSE106481	30154223	8/30/18	Single-Cell Rna-Sequencing Reveals The Existence Of Naive And Primed Pluripotency In Pre-Implantation Rhesus Monkey Embryos.
GSE106514	30865898	3/14/19	Single-Cell Analysis Reveals Heterogeneity Of High Endothelial Venules And Different Regulation Of Genes Controlling Lymphocyte Entry To Lymph Nodes.
GSE106542	29352091	1/21/18	Precursors of human CD4+ cytotoxic T lymphocytes identified by single-cell transcriptome analysis
GSE106543	29352091	1/21/18	Precursors of human CD4+ cytotoxic T lymphocytes identified by single-cell transcriptome analysis
GSE106973	29588278	3/29/18	A Single-Cell Hematopoietic Landscape Resolves 8 Lineage Trajectories And Defects In Kit Mutant Mice.
GSE107185	30448000	11/19/18	Mapping Cellular Reprogramming Via Pooled Overexpression Screens With Paired Fitness And Single-Cell Rna-Sequencing Readout.
GSE107585	29622724	4/7/18	Single-Cell Transcriptomics Of The Mouse Kidney Reveals Potential Cellular Targets Of Kidney Disease.
GSE107618	31269016	7/4/19	Dissecting The Transcriptome Landscape Of The Human Fetal Neural Retina And Retinal Pigment Epithelium By Single-Cell Rna-Seq Analysis.
GSE107632	29320739	1/11/18	Variation In Activity State, Axonal Projection, And Position Define The Transcriptional Identity Of Individual Neocortical Projection Neurons.
GSE107653	29300724	1/5/18	A Novel Prospective Isolation Of Murine Fetal Liver Progenitors To Study In Utero Hematopoietic Defects.
GSE107727	29588278	3/29/18	A Single-Cell Hematopoietic Landscape Resolves 8 Lineage Trajectories And Defects In Kit Mutant Mice.
GSE107858	30463022	11/22/18	Single-Cell Transcriptome Analysis Reveals Estrogen Signaling Coordinately Augments One-Carbon, Polyamine, And Purine Synthesis In Breast Cancer.
GSE108020	29499164	3/3/18	Single-Cell Rna-Seq Of Mouse Dopaminergic Neurons Informs Candidate Gene Selection For Sporadic Parkinson Disease.
GSE108097	29775597	5/19/18	Mapping The Mouse Cell Atlas By Microwell-Seq.
GSE108108	29031728	10/17/17	Single-Cell Rna-Sequencing Reveals A Distinct Population Of Proglucagon-Expressing Cells Specific To The Mouse Upper Small Intestine.
GSE108155	30021172	7/19/18	Differences In Cell Cycle Status Underlie Transcriptional Heterogeneity In The Hsc Compartment.
GSE108383	30061114	8/1/18	Single-Cell Rna-Seq Analysis Identifies Markers Of Resistance To Targeted Braf Inhibitors In Melanoma Cell Populations.
GSE108699	29457792	2/20/18	On The Design Of Crispr-Based Single-Cell Molecular Screens.
GSE108788	29398364	2/6/18	Graded Arrays Of Spinal And Supraspinal V2A Interneuron Subtypes Underlie Forelimb And Hindlimb Motor Control.
GSE108849	29630593	4/10/18	A Multitask Clustering Approach For Single-Cell Rna-Seq Analysis In Recessive Dystrophic Epidermolysis Bullosa.
GSE108892	31296938	7/13/19	Author Correction: The Bone Marrow Microenvironment At Single-Cell Resolution.
GSE108970	30404016	11/8/18	The Mammalian Spermatogenesis Single-Cell Transcriptome, From Spermatogonial Stem Cells To Spermatids.
GSE108974	30404016	11/8/18	The Mammalian Spermatogenesis Single-Cell Transcriptome, From Spermatogonial Stem Cells To Spermatids.
GSE108977	30404016	11/8/18	The Mammalian Spermatogenesis Single-Cell Transcriptome, From Spermatogonial Stem Cells To Spermatids.
GSE108989	30479382	11/28/18	Lineage Tracking Reveals Dynamic Relationships Of T Cells In Colorectal Cancer.
GSE109037	30404016	11/8/18	The Mammalian Spermatogenesis Single-Cell Transcriptome, From Spermatogonial Stem Cells To Spermatids.
GSE109100	31374198	8/3/19	Interconversion Between Tumorigenic And Differentiated States In Acute Myeloid Leukemia.
GSE109100	31374198	8/3/19	Interconversion Between Tumorigenic And Differentiated States In Acute Myeloid Leukemia.
GSE109113	29550076	3/20/18	Long-Term Correction Of Diabetes In Mice By In¬†Vivo Reprogramming Of Pancreatic Ducts.
GSE109262	29472610	2/24/18	Scnmt-Seq Enables Joint Profiling Of Chromatin Accessibility Dna Methylation And Transcription In Single Cells.
GSE109308	31744909	11/21/19	Genomic And Transcriptomic Profiling Of Carcinogenesis In Patients With Familial Adenomatous Polyposis.
GSE109324	30792200	2/23/19	Bone Marrow Endothelial Cell-Derived Interleukin-4 Contributes To Thrombocytopenia In Acute Myeloid Leukemia.
GSE109535	30462277	11/22/18	Scftd-Seq: Freeze-Thaw Lysis Based, Portable Approach Toward Highly Distributed Single-Cell 3' Mrna Profiling.
GSE109564	29980650	7/8/18	Single-Cell Transcriptomics Of A Human Kidney Allograft Biopsy Specimen Defines A Diverse Inflammatory Response.
GSE109726	29434354	2/13/18	Single-Cell Gene Expression Reveals A Landscape Of Regulatory T Cell Phenotypes Shaped By The Tcr.
GSE109774	30283141	10/5/18	Single-Cell Transcriptomics Of 20 Mouse Organs Creates A Tabula Muris.
GSE109816	31915373	1/10/20	Single-Cell Reconstruction Of The Adult Human Heart During Heart Failure And Recovery Reveals The Cellular Landscape Underlying Cardiac Function.
GSE109822	29391249	2/3/18	Spatial And Single-Cell Transcriptional Profiling Identifies Functionally Distinct Human Dermal Fibroblast Subpopulations.
GSE109999	30096152	8/11/18	Scpipe: A Flexible R/Bioconductor Preprocessing Pipeline For Single-Cell Rna-Sequencing Data.
GSE110010	29643508	4/13/18	Aspm Knockout Ferret Reveals An Evolutionary Mechanism Governing Cerebral Cortical Size.
GSE110265	30128894	8/22/18	Dgcr8 Deletion In The Primitive Heart Uncovered Novel Microrna Regulating The Balance Of Cardiac-Vascular Gene Program.
GSE110357	29670281	4/20/18	Identification Of The Tumour Transition States Occurring During Emt.
GSE110371	29666189	4/19/18	Single-Cell Rna-Seq Reveals Cell Heterogeneity And Hierarchy Within Mouse Mammary Epithelia.
GSE110499	29898899	6/15/18	Linking Transcriptional And Genetic Tumor Heterogeneity Through Allele Analysis Of Single-Cell Rna-Seq Data.

GSE110513	29608179	4/3/18	Integrating Single-Cell Transcriptomic Data Across Different Conditions, Technologies, And Species.
GSE110547	29434354	2/13/18	Single-Cell Gene Expression Reveals A Landscape Of Regulatory T Cell Phenotypes Shaped By The Tcr.
GSE110558	29434354	2/13/18	Single-Cell Gene Expression Reveals A Landscape Of Regulatory T Cell Phenotypes Shaped By The Tcr.
GSE110568	29434354	2/13/18	Single-Cell Gene Expression Reveals A Landscape Of Regulatory T Cell Phenotypes Shaped By The Tcr.
GSE110679	29434354	2/13/18	Single-Cell Gene Expression Reveals A Landscape Of Regulatory T Cell Phenotypes Shaped By The Tcr.
GSE110686	29942092	6/27/18	Single-Cell Profiling Of Breast Cancer T Cells Reveals A Tissue-Resident Memory Subset Associated With Improved Prognosis.
GSE110688	29434354	2/13/18	Single-Cell Gene Expression Reveals A Landscape Of Regulatory T Cell Phenotypes Shaped By The Tcr.
GSE110692	29434354	2/13/18	Single-Cell Gene Expression Reveals A Landscape Of Regulatory T Cell Phenotypes Shaped By The Tcr.
GSE110746	30559380	12/19/18	Loss Of Adar1 In Tumours Overcomes Resistance To Immune Checkpoint Blockade.
GSE110894	31222014	6/22/19	Targeting Enhancer Switching Overcomes Non-Genetic Drug Resistance In Acute Myeloid Leukaemia.
GSE111014	31996669	1/31/20	Chromatin Mapping And Single-Cell Immune Profiling Define The Temporal Dynamics Of Ibrutinib Response In Cll.
GSE111027	30487586	11/30/18	Single-Cell Rna Sequencing Unveils An Il-10-Producing Helper Subset That Sustains Humoral Immunity During Persistent Infection.
GSE111070	29434354	2/13/18	Single-Cell Gene Expression Reveals A Landscape Of Regulatory T Cell Phenotypes Shaped By The Tcr.
GSE111108	30096152	8/11/18	Scpipe: A Flexible R/Bioconductor Preprocessing Pipeline For Single-Cell Rna-Sequencing Data.
GSE111113	30089273	8/9/18	Single-Cell Transcriptomes Distinguish Stem Cell State Changes And Lineage Specification Programs In Early Mammary Gland Development.
GSE111229	30514914	12/6/18	Spatially And Functionally Distinct Subclasses Of Breast Cancer-Associated Fibroblasts Revealed By Single Cell Rna Sequencing.
GSE111360	30550791	12/15/18	Organoid Modeling Of The Tumor Immune Microenvironment.
GSE111402	30357357	10/26/18	Single-Cell Stabilization Method Identifies Gonadotrope Transcriptional Dynamics And Pituitary Cell Type Heterogeneity.
GSE111461	30357357	10/26/18	Single-Cell Stabilization Method Identifies Gonadotrope Transcriptional Dynamics And Pituitary Cell Type Heterogeneity.
GSE111588	30265241	9/29/18	Identification Of Functionally Distinct Fibro-Inflammatory And Adipogenic Stromal Subpopulations In Visceral Adipose Tissue Of Adult Mice.
GSE111672	31932730	1/15/20	Integrating Microarray-Based Spatial Transcriptomics And Single-Cell Rna-Seq Reveals Tissue Architecture In Pancreatic Ductal Adenocarcinomas.
GSE111860	30108108	8/16/18	Induced Pluripotent Stem Cell-Based Mapping Of Œí-Globin Expression Throughout Human Erythropoietic Development.
GSE111892	31227543	6/23/19	Single-Cell Transcriptomic Analysis Of Tissue-Resident Memory T Cells In Human Lung Cancer.
GSE111896	31227543	6/23/19	Single-Cell Transcriptomic Analysis Of Tissue-Resident Memory T Cells In Human Lung Cancer.
GSE111901	29434354	2/13/18	Single-Cell Gene Expression Reveals A Landscape Of Regulatory T Cell Phenotypes Shaped By The Tcr.
GSE111976	32929266	9/16/20	Single-Cell Transcriptomic Atlas Of The Human Endometrium During The Menstrual Cycle.
GSE112008	30218073	9/16/18	Med23 Serves As A Gatekeeper Of The Myeloid Potential Of Hematopoietic Stem Cells.
GSE112013	30315278	10/14/18	The Adult Human Testis Transcriptional Cell Atlas.
GSE112177	29970990	7/5/18	A Guide To Single-Cell Transcriptomics In Adult Rodent Brain: The Medium Spiny Neuron Transcriptome Revisited.
GSE112271	31941899	1/17/20	Intratumoral Heterogeneity And Clonal Evolution In Liver Cancer.
GSE112417	29871976	6/7/18	Histone Variant H2A.Z Is Required For The Maintenance Of Smooth Muscle Cell Identity As Revealed By Single-Cell Transcriptomics.
GSE112507	30681766	1/27/19	Crx Expression In Pluripotent Stem Cell-Derived Photoreceptors Marks A Transplantable Subpopulation Of Early Cones.
GSE112692	30463007	11/22/18	The Molecular Signature Of Megakaryocyte-Erythroid Progenitors Reveals A Role For The Cell Cycle In Fate Specification.
GSE112865	30613266	1/8/19	Arg1 Expression Defines Immunosuppressive Subsets Of Tumor-Associated Macrophages.
GSE112903	29752062	5/13/18	Single-Cell Rna Sequencing Of Lymph Node Stromal Cells Reveals Niche-Associated Heterogeneity.
GSE113046	31126997	5/28/19	Identification Of Two Distinct Pathways Of Human Myelopoiesis.
GSE113049	30913038	3/27/19	Single Cell Rna Sequencing Identifies TgfŒ≤ As A Key Regenerative Cue Following Lps-Induced Lung Injury.
GSE113074	29700227	4/28/18	The Dynamics Of Gene Expression In Vertebrate Embryogenesis At Single-Cell Resolution.
GSE113099	29795293	5/26/18	Profiling Human Breast Epithelial Cells Using Single Cell Rna Sequencing Identifies Cell Diversity.
GSE113127	29795293	5/26/18	Profiling Human Breast Epithelial Cells Using Single Cell Rna Sequencing Identifies Cell Diversity.
GSE113196	29795293	5/26/18	Profiling Human Breast Epithelial Cells Using Single Cell Rna Sequencing Identifies Cell Diversity.
GSE113198	29795293	5/26/18	Profiling Human Breast Epithelial Cells Using Single Cell Rna Sequencing Identifies Cell Diversity.
GSE113415	32699019	7/24/20	Parallel Bimodal Single-Cell Sequencing Of Transcriptome And Chromatin Accessibility.
GSE113417	32699019	7/24/20	Parallel Bimodal Single-Cell Sequencing Of Transcriptome And Chromatin Accessibility.
GSE113576	30385464	11/6/18	Molecular, Spatial, And Functional Single-Cell Profiling Of The Hypothalamic Preoptic Region.
GSE113616	31155233	6/4/19	Stromal Microenvironment Shapes The Intratumoral Architecture Of Pancreatic Cancer.
GSE113807	29899446	6/15/18	Induction And Transcriptional Regulation Of The Co-Inhibitory Gene Module In T Cells.
GSE113854	30737373	2/10/19	Single-Cell Analysis Reveals Fibroblast Heterogeneity And Myeloid-Derived Adipocyte Progenitors In Murine Skin Wounds.
GSE113973	30420755	11/14/18	Disease-Specific Oligodendrocyte Lineage Cells Arise In Multiple Sclerosis.
GSE114156	29980650	7/8/18	Single-Cell Transcriptomics Of A Human Kidney Allograft Biopsy Specimen Defines A Diverse Inflammatory Response.
GSE114157	30865901	3/14/19	Insights Into The Biology Of Hearing And Deafness Revealed By Single-Cell Rna Sequencing.
GSE114161	30127440	8/22/18	Single-Cell Analysis Reveals That Stochasticity And Paracrine Signaling Control Interferon-Alpha Production By Plasmacytoid Dendritic Cells.
GSE114396	31263279	7/3/19	C-Kit-Positive Ilc2S Exhibit An Ilc3-Like Signature That May Contribute To Il-17-Mediated Pathologies.
GSE114446	32362655	5/5/20	Integrated Single-Cell And Bulk Gene Expression And Atac-Seq Reveals Heterogeneity And Early Changes In Pathways Associated With Resistance To Cetuxi
GSE114530	30789893	2/23/19	Single-Cell Transcriptomics Reveals Gene Expression Dynamics Of Human Fetal Kidney Development.
GSE114687	31477929	9/4/19	A Pooled Single-Cell Genetic Screen Identifies Regulatory Checkpoints In The Continuum Of The Epithelial-To-Mesenchymal Transition.
GSE114704	32499301	6/6/20	Discovery Of New Targets To Control Metastasis In Pancreatic Cancer By Single-Cell Transcriptomics Analysis Of Circulating Tumor Cells.
GSE114727	29961579	7/3/18	Single-Cell Map Of Diverse Immune Phenotypes In The Breast Tumor Microenvironment.
GSE114793	29899037	6/15/18	Dissection Of Progenitor Compartments Resolves Developmental Trajectories In B-Lymphopoiesis.
GSE114822	30560925	12/19/18	Integrative Single-Cell Analysis Of Transcriptome, Dna Methylome And Chromatin Accessibility In Mouse Oocytes.
GSE114986	31566561	10/1/19	Coopted Temporal Patterning Governs Cellular Hierarchy, Heterogeneity And Metabolism In
GSE114997	30078709	8/7/18	Sensory Neuron Diversity In The Inner Ear Is Shaped By Activity.
GSE115006	29967419	7/4/18	Human In Vivo-Generated Monocyte-Derived Dendritic Cells And Macrophages Cross-Present Antigens Through A Vacuolar Pathway.
GSE115007	29967419	7/4/18	Human In Vivo-Generated Monocyte-Derived Dendritic Cells And Macrophages Cross-Present Antigens Through A Vacuolar Pathway.
GSE115125	29434354	2/13/18	Single-Cell Gene Expression Reveals A Landscape Of Regulatory T Cell Phenotypes Shaped By The Tcr.
GSE115149	30679801	1/27/19	Inflammation-Induced Glycolytic Switch Controls Suppressivity Of Mesenchymal Stem Cells Via Stat1 Glycosylation.
GSE115189	30228881	1/5/19	Comparison Of Clustering Tools In R For Medium-Sized 10X Genomics Single-Cell Rna-Sequencing Data.
GSE115214	30827679	3/5/19	Lineage Tracing In Humans Enabled By Mitochondrial Mutations And Single-Cell Genomics.
GSE115280	30143032	8/26/18	Comparative Transcriptomic Analyses And Single-Cell Rna Sequencing Of The Freshwater Planarian Schmidtea Mediterranea Identify Major Cell Types And
GSE115469	30348985	10/24/18	Single Cell Rna Sequencing Of Human Liver Reveals Distinct Intrahepatic Macrophage Populations.
GSE115600	30827680	3/5/19	Quiescence Modulates Stem Cell Maintenance And Regenerative Capacity In The Aging Brain.
GSE115622	30827680	3/5/19	Quiescence Modulates Stem Cell Maintenance And Regenerative Capacity In The Aging Brain.
GSE115861	30728416	2/8/19	Combined Quantification Of Intracellular (Phospho-)Proteins And Transcriptomics From Fixed Single Cells.
GSE115934	30455179	11/21/18	Characterization Of Spatial And Temporal Development Of Type I And Type Ii Hair Cells In The Mouse Utricle Using New Cell-Type-Specific Markers.
GSE115943	30712874	2/5/19	Optimal-Transport Analysis Of Single-Cell Gene Expression Identifies Developmental Trajectories In Reprogramming.
GSE115978	30388455	11/6/18	A Cancer Cell Program Promotes T Cell Exclusion And Resistance To Checkpoint Blockade.
GSE115997	32350740	5/1/20	Integrative Analysis Of In Vivo Recording With Single-Cell Rna-Seq Data Reveals Molecular Properties Of Light-Sensitive Neurons In Mouse V1.
GSE116106	32386599	5/11/20	Single-Cell Analysis Of Human Retina Identifies Evolutionarily Conserved And Species-Specific Mechanisms Controlling Development.
GSE116113	30361550	10/27/18	Expansion And Differentiation Of Human Hepatocyte-Derived Liver Progenitor-Like Cells And Their Use For The Study Of Hepatotropic Pathogens.
GSE116222	30814735	3/1/19	Colonic Epithelial Cell Diversity In Health And Inflammatory Bowel Disease.
GSE116256	30827681	3/5/19	Single-Cell Rna-Seq Reveals Aml Hierarchies Relevant To Disease Progression And Immunity.
GSE116481	30420752	11/14/18	Venetoclax With Azacitidine Disrupts Energy Metabolism And Targets Leukemia Stem Cells In Patients With Acute Myeloid Leukemia.
GSE116530	32367048	5/6/20	Differentiation Of Transplanted Haematopoietic Stem Cells Tracked By Single-Cell Transcriptomic Analysis.
GSE116555	29986945	7/11/18	Large-Scale Single-Cell Rna-Seq Reveals Molecular Signatures Of Heterogeneous Populations Of Human Induced Pluripotent Stem Cell-Derived Endothelial C
GSE117156	30523328	12/14/18	Single Cell Dissection Of Plasma Cell Heterogeneity In Symptomatic And Asymptomatic Myeloma.
GSE117218	30154223	8/30/18	Single-Cell Rna-Sequencing Reveals The Existence Of Naive And Primed Pluripotency In Pre-Implantation Rhesus Monkey Embryos.
GSE117225	30827895	3/5/19	Rate Of Progression Through A Continuum Of Transit-Amplifying Progenitor Cell States Regulates Blood Cell Production.
GSE117228	30827895	3/5/19	Rate Of Progression Through A Continuum Of Transit-Amplifying Progenitor Cell States Regulates Blood Cell Production.
GSE117231	30827895	3/5/19	Rate Of Progression Through A Continuum Of Transit-Amplifying Progenitor Cell States Regulates Blood Cell Production.
GSE117295	32066983	2/19/20	Spatiotemporal Single-Cell Analysis Of Gene Expression In The Mouse Suprachiasmatic Nucleus.
GSE117403	30566875	12/20/18	A Cellular Anatomy Of The Normal Adult Human Prostate And Prostatic Urethra.
GSE117570	31033233	4/30/19	Dissecting Intratumoral Myeloid Cell Plasticity By Single Cell Rna-Seq.
GSE117617	30096152	8/11/18	Scpipe: A Flexible R/Bioconductor Preprocessing Pipeline For Single-Cell Rna-Sequencing Data.
GSE117618	30096152	8/11/18	Scpipe: A Flexible R/Bioconductor Preprocessing Pipeline For Single-Cell Rna-Sequencing Data.

GSE117790	31216470	6/20/19	Rational Reprogramming Of Cellular States By Combinatorial Perturbation.
GSE117792	31216470	6/20/19	Rational Reprogramming Of Cellular States By Combinatorial Perturbation.
GSE117793	31216470	6/20/19	Rational Reprogramming Of Cellular States By Combinatorial Perturbation.
GSE117794	31216470	6/20/19	Rational Reprogramming Of Cellular States By Combinatorial Perturbation.
GSE117837	31068579	5/10/19	Single Cell Transcriptomic Analysis Of Human Mesenchymal Stem Cells Reveals Limited Heterogeneity.
GSE117872	30467425	11/24/18	Longitudinal Single-Cell Rna Sequencing Of Patient-Derived Primary Cells Reveals Drug-Induced Infidelity In Stem Cell Hierarchy.
GSE117988	30250229	9/27/18	Acquired Cancer Resistance To Combination Immunotherapy From Transcriptional Loss Of Class I Hla.
GSE118055	30154153	8/30/18	Transcription Factor Prox1 Suppresses Notch Pathway Activation Via The Nucleosome Remodeling And Deacetylase Complex In Colorectal Cancer Stem-Like
GSE118068	31043743	5/3/19	Childhood Cerebellar Tumours Mirror Conserved Fetal Transcriptional Programs.
GSE118127	31320652	7/20/19	Single-Cell Reconstruction Of Follicular Remodeling In The Human Adult Ovary.
GSE118184	30449713	11/20/18	Comparative Analysis And Refinement Of Human Psc-Derived Kidney Organoid Differentiation With Single-Cell Transcriptomics.
GSE118234	32084387	2/23/20	High-Resolution Dissection Of Chemical Reprogramming From Mouse Embryonic Fibroblasts Into Fibrocartilaginous Cells.
GSE118257	30747918	2/13/19	Altered Human Oligodendrocyte Heterogeneity In Multiple Sclerosis.
GSE118292	30765463	2/16/19	The Transcription Factor Duxbl Mediates Elimination Of Pre-T Cells That Fail Œí-Selection.
GSE118403	32066983	2/19/20	Spatiotemporal Single-Cell Analysis Of Gene Expression In The Mouse Suprachiasmatic Nucleus.
GSE118487	32792525	8/15/20	Cellular And Molecular Properties Of Neural Progenitors In The Developing Mammalian Hypothalamus.
GSE118546	30712875	2/5/19	Molecular Classification And Comparative Taxonomics Of Foveal And Peripheral Cells In Primate Retina.
GSE118704	30096152	8/11/18	Scpipe: A Flexible R/Bioconductor Preprocessing Pipeline For Single-Cell Rna-Sequencing Data.
GSE118706	30096152	8/11/18	Scpipe: A Flexible R/Bioconductor Preprocessing Pipeline For Single-Cell Rna-Sequencing Data.
GSE118734	30245212	9/25/18	Reproducibility Of Molecular Phenotypes After Long-Term Differentiation To¬†Human Ipsc-Derived Neurons: A Multi-Site Omics Study.
GSE118828	30383866	11/2/18	Identification Of Grade And Origin Specific Cell Populations In Serous Epithelial Ovarian Cancer By Single Cell Rna-Seq.
GSE118852	30712875	2/5/19	Molecular Classification And Comparative Taxonomics Of Foveal And Peripheral Cells In Primate Retina.
GSE118953	31073041	5/11/19	Temporal Patterning Of Apical Progenitors And Their Daughter Neurons In The Developing Neocortex.
GSE119352	30445041	11/18/18	High-Dimensional Analysis Delineates Myeloid And Lymphoid Compartment Remodeling During Successful Immune-Checkpoint Cancer Therapy.
GSE119373	30397350	11/7/18	Human retinoic acid-regulated CD161 + regulatory T cells support wound repair in intestinal mucosa
GSE119455	30463022	11/22/18	Single-Cell Transcriptome Analysis Reveals Estrogen Signaling Coordinately Augments One-Carbon, Polyamine, And Purine Synthesis In Breast Cancer.
GSE119506	31072818	5/11/19	Human Lymphoid Organ Cdc2 And Macrophages Play Complementary Roles In T Follicular Helper Responses.
GSE119507	31072818	5/11/19	Human Lymphoid Organ Cdc2 And Macrophages Play Complementary Roles In T Follicular Helper Responses.
GSE119531	30510133	12/5/18	Advantages Of Single-Nucleus Over Single-Cell Rna Sequencing Of Adult Kidney: Rare Cell Types And Novel Cell States Revealed In Fibrosis.
GSE119766	30893600	3/21/19	Dissecting Cell Lineage Specification And Sex Fate Determination In Gonadal Somatic Cells Using Single-Cell Transcriptomics.
GSE119893	30548510	12/15/18	Deconstructing Retinal Organoids: Single Cell Rna-Seq Reveals The Cellular Components Of Human Pluripotent Stem Cell-Derived Retina.
GSE119926	31341285	7/26/19	Resolving Medulloblastoma Cellular Architecture By Single-Cell Genomics.
GSE119945	30787437	2/23/19	The Single-Cell Transcriptional Landscape Of Mammalian Organogenesis.
GSE120046	32923614	9/15/20	Single-Cell Transcriptome Analysis Reveals Cell Lineage Specification In Temporal-Spatial Patterns In Human Cortical Development.
GSE120064	32098504	2/27/20	Single-Cell Reconstruction Of Progression Trajectory Reveals Intervention Principles In Pathological Cardiac Hypertrophy.
GSE120372	30735127	2/9/19	Specification Of Diverse Cell Types During Early Neurogenesis Of The Mouse Cerebellum.
GSE120410	30335147	10/20/18	Single-Cell Rna Sequencing Reveals Novel Markers Of Male Pituitary Stem Cells And Hormone-Producing Cell Types.
GSE120506	30315278	10/14/18	The Adult Human Testis Transcriptional Cell Atlas.
GSE120508	31950241	1/18/20	Evaluating Genetic Causes Of Azoospermia: What Can We Learn From A Complex Cellular Structure And Single-Cell Transcriptomics Of The Human Testis?
GSE120575	30633907	1/12/19	Defining T Cell States Associated With Response To Checkpoint Immunotherapy In Melanoma.
GSE120629	30760929	2/15/19	Spatial And Temporal Heterogeneity Of Mouse And Human Microglia At Single-Cell Resolution.
GSE120716	30566875	12/20/18	A Cellular Anatomy Of The Normal Adult Human Prostate And Prostatic Urethra.
GSE120744	30760929	2/15/19	Spatial And Temporal Heterogeneity Of Mouse And Human Microglia At Single-Cell Resolution.
GSE120745	30760929	2/15/19	Spatial And Temporal Heterogeneity Of Mouse And Human Microglia At Single-Cell Resolution.
GSE120909	30389797	11/6/18	Combination cancer immunotherapy targeting PD-1 and GITR can rescue CD8+ T cell dysfunction and maintain memory phenotype
GSE120960	30655503	1/19/19	Setd5 Haploinsufficiency Alters Neuronal Network Connectivity And Leads To Autistic-Like Behaviors In Mice.
GSE120966	31474569	9/3/19	Single-Cell Transcriptomics In Medulloblastoma Reveals Tumor-Initiating Progenitors And Oncogenic Cascades During Tumorigenesis And Relapse.
GSE120974	31474569	9/3/19	Single-Cell Transcriptomics In Medulloblastoma Reveals Tumor-Initiating Progenitors And Oncogenic Cascades During Tumorigenesis And Relapse.
GSE121069	30540963	12/13/18	Axon-Seq Decodes The Motor Axon Transcriptome And Its Modulation In Response To Als.
GSE121265	32312741	4/22/20	Characterizing And Inferring Quantitative Cell Cycle Phase In Single-Cell Rna-Seq Data Analysis.
GSE121380	31730855	11/16/19	Mucosal Profiling Of Pediatric-Onset Colitis And Ibd Reveals Common Pathogenics And Therapeutic Pathways.
GSE121393	30575716	12/24/18	Molecular And Functional Heterogeneity Of Il-10-Producing Cd4
GSE121579	30503262	12/7/18	Transcription Factor Levels After Forward Programming Of Human Pluripotent Stem Cells With Gata1, Fli1, And Tal1 Determine Megakaryocyte Versus Eryt
GSE121650	31827285	12/13/19	Multi-Omics Profiling Of Mouse Gastrulation At Single-Cell Resolution.
GSE121654	30471926	11/26/18	Single-Cell Rna Sequencing Of Microglia Throughout The Mouse Lifespan And In The Injured Brain Reveals Complex Cell-State Changes.
GSE121862	31249312	6/30/19	A Single-Nucleus Rna-Sequencing Pipeline To Decipher The Molecular Anatomy And Pathophysiology Of Human Kidneys.
GSE121891	30517858	12/6/18	Single-Cell Rna-Seq Of Mouse Olfactory Bulb Reveals Cellular Heterogeneity And Activity-Dependent Molecular Census Of Adult-Born Neurons.
GSE121893	31915373	1/10/20	Single-Cell Reconstruction Of The Adult Human Heart During Heart Failure And Recovery Reveals The Cellular Landscape Underlying Cardiac Function.
GSE121904	30893341	3/21/19	Dynamic Transcriptome Profiles Within Spermatogonial And Spermatocyte Populations During Postnatal Testis Maturation Revealed By Single-Cell Sequenc
GSE121937	31804471	12/6/19	Heterogeneity And Dynamics Of Active Kras-Induced Dysplastic Lineages From Mouse Corpus Stomach.
GSE121940	31804471	12/6/19	Heterogeneity And Dynamics Of Active Kras-Induced Dysplastic Lineages From Mouse Corpus Stomach.
GSE121957	30418965	11/13/18	Single-Cell Rna-Sequencing Reveals Transcriptional Dynamics Of Estrogen-Induced Dysplasia In The Ovarian Surface Epithelium.
GSE122009	32459995	5/28/20	A Multiplexed Barcodelet Single-Cell Rna-Seq Approach Elucidates Combinatorial Signaling Pathways That Drive Esc Differentiation.
GSE122012	30872278	3/16/19	Defining Developmental Diversification Of Diencephalon Neurons Through Single Cell Gene Expression Profiling.
GSE122026	30595537	1/1/19	Dermal Condensate Niche Fate Specification Occurs Prior To Formation And Is Placode Progenitor Dependent.
GSE122198	30765193	2/16/19	Unravelling Intratumoral Heterogeneity Through High-Sensitivity Single-Cell Mutational Analysis And Parallel Rna Sequencing.
GSE122403	31142541	5/31/19	Single Cell Expression Analysis Reveals Anatomical And Cell Cycle-Dependent Transcriptional Shifts During Heart Development.
GSE122466	31399471	8/11/19	Single-Cell Transcriptional Logic Of Cell-Fate Specification And Axon Guidance In Early-Born Retinal Neurons.
GSE122675	30778252	2/20/19	Subsets of exhausted CD8+ T cells differentially mediate tumor control and respond to checkpoint blockade
GSE122712	30778252	2/20/19	Subsets of exhausted CD8+ T cells differentially mediate tumor control and respond to checkpoint blockade
GSE122730	30728335	2/8/19	Cxcr3-Expressing Leukocytes Are Necessary For Neurofibroma Formation In Mice.
GSE122743	31477698	9/4/19	Single-Cell Transcriptomics Reveals Multi-Step Adaptations To Endocrine Therapy.
GSE122817	32259486	4/8/20	Defining The Design Principles Of Skin Epidermis Postnatal Growth.
GSE122843	31222014	6/22/19	Targeting Enhancer Switching Overcomes Non-Genetic Drug Resistance In Acute Myeloid Leukaemia.
GSE122846	30664737	1/22/19	Memory CD4+ T cells are generated in the human fetal intestine
GSE122969	30635236	1/13/19	Checkpoint Blockade Immunotherapy Induces Dynamic Changes In Pd-1
GSE122970	32386599	5/11/20	Single-Cell Analysis Of Human Retina Identifies Evolutionarily Conserved And Species-Specific Mechanisms Controlling Development.
GSE123022	30606613	1/5/19	Developmental Heterogeneity Of Microglia And Brain Myeloid Cells Revealed By Deep Single-Cell Rna Sequencing.
GSE123024	30606613	1/5/19	Developmental Heterogeneity Of Microglia And Brain Myeloid Cells Revealed By Deep Single-Cell Rna Sequencing.
GSE123025	30606613	1/5/19	Developmental Heterogeneity Of Microglia And Brain Myeloid Cells Revealed By Deep Single-Cell Rna Sequencing.
GSE123046	30959515	4/9/19	The Emergent Landscape Of The Mouse Gut Endoderm At Single-Cell Resolution.
GSE123067	30643267	1/16/19	Thymic Regulatory T Cells Arise Via Two Distinct Developmental Programs.
GSE123078	30971824	4/12/19	The Bone Marrow Microenvironment At Single-Cell Resolution.
GSE123139	32359441	5/4/20	Dysfunctional Cd8¬†T Cells Form A Proliferative, Dynamically Regulated Compartment Within Human Melanoma.
GSE123476	28111004	1/24/17	Single Cell Sequencing Reveals Heterogeneity Within Ovarian Cancer Epithelium And Cancer Associated Stromal Cells.
GSE123515	31019301	4/26/19	Single-Cell Transcriptomes Of The Regenerating Intestine Reveal A Revival Stem Cell.
GSE123813	31359002	7/31/19	Clonal Replacement Of Tumor-Specific T Cells Following Pd-1 Blockade.
GSE123899	32699019	7/24/20	Parallel Bimodal Single-Cell Sequencing Of Transcriptome And Chromatin Accessibility.
GSE123904	32042191	2/12/20	Regenerative Lineages And Immune-Mediated Pruning In Lung Cancer Metastasis.
GSE123926	30770823	2/17/19	Barcoding Reveals Complex Clonal Behavior In Patient-Derived Xenografts Of Metastatic Triple Negative Breast Cancer.
GSE124172	31907413	1/8/20	Multipotent Rag1+ Progenitors Emerge Directly From Haemogenic Endothelium In Human Pluripotent Stem Cell-Derived Haematopoietic Organoids.
GSE124174	30886407	3/20/19	Cerebral Organoids At The Air-Liquid Interface Generate Diverse Nerve Tracts With Functional Output.
GSE124263	30726734	2/7/19	The Neonatal And Adult Human Testis Defined At The Single-Cell Level.
GSE124299	30735633	2/9/19	Establishing Cerebral Organoids As Models Of Human-Specific Brain Evolution.

GSE124312	31116992	5/23/19	An Atlas Of Vagal Sensory Neurons And Their Molecular Specialization.
GSE124334	30949159	4/6/19	Single-Cell Transcriptome Analysis of CD34+ Stem Cell-Derived Myeloid Cells Infected With Human Cytomegalovirus
GSE124454	27131741	5/2/16	Heterogeneity Of Mesp1+ Mesoderm Revealed By Single-Cell Rna-Seq.
GSE124742	32302527	4/18/20	Patch-Seq Links Single-Cell Transcriptomes To Human Islet Dysfunction In Diabetes.
GSE124872	30814501	3/1/19	An Atlas Of The Aging Lung Mapped By Single Cell Transcriptomics And Deep Tissue Proteomics.
GSE124887	31308046	7/17/19	Set Domain-Containing Protein 4 Epigenetically Controls Breast Cancer Stem Cell Quiescence.
GSE124888	31308046	7/17/19	Set Domain-Containing Protein 4 Epigenetically Controls Breast Cancer Stem Cell Quiescence.
GSE124904	31243281	6/28/19	Developmental Kinetics And Transcriptome Dynamics Of Stem Cell Specification In The Spermatogenic Lineage.
GSE125269	31573981	10/2/19	Low- And High-Thermogenic Brown Adipocyte Subpopulations Coexist In Murine Adipose Tissue.
GSE125449	31588021	10/8/19	Tumor Cell Biodiversity Drives Microenvironmental Reprogramming In Liver Cancer.
GSE125527	32826341	8/23/20	Heterogeneity And Clonal Relationships Of Adaptive Immune Cells In Ulcerative Colitis Revealed By Single-Cell Analyses.
GSE125881	31924795	1/12/20	Clonal Kinetics And Single-Cell Transcriptional Profiling Of Car-T Cells In Patients Undergoing Cd19 Car-T Immunotherapy.
GSE125970	31753849	11/23/19	Single-Cell Transcriptome Analysis Reveals Differential Nutrient Absorption Functions In Human Intestine.
GSE126022	30982595	4/16/19	Human Pluripotency Is Initiated And Preserved By A Unique Subset Of Founder Cells.
GSE126111	31270426	7/5/19	An Optimised Tissue Disaggregation And Data Processing Pipeline For Characterising Fibroblast Phenotypes Using Single-Cell Rna Sequencing.
GSE126128	31341279	7/26/19	Single-Cell Analysis Of Cardiogenesis Reveals Basis For Organ-Level Developmental Defects.
GSE126388	30799483	2/26/19	Superct: A Supervised-Learning Framework For Enhanced Characterization Of Single-Cell Transcriptomic Profiles.
GSE126480	31420539	8/20/19	Single Cell Transcriptome Analysis Of Developing Arcuate Nucleus Neurons Uncovers Their Key Developmental Regulators.
GSE126819	31399471	8/11/19	Single-Cell Transcriptional Logic Of Cell-Fate Specification And Axon Guidance In Early-Born Retinal Neurons.
GSE126906	30096152	8/11/18	Scpipe: A Flexible R/Bioconductor Preprocessing Pipeline For Single-Cell Rna-Sequencing Data.
GSE127005	31053654	5/6/19	Distinct Immunocyte-Promoting And Adipocyte-Generating Stromal Components Coordinate Adipose Tissue Immune And Metabolic Tenors.
GSE127235	30846559	3/9/19	Single-Cell Rna Profiling Of Glomerular Cells Shows Dynamic Changes In Experimental Diabetic Kidney Disease.
GSE127471	31061481	5/8/19	Determining Cell Type Abundance And Expression From Bulk Tissues With Digital Cytometry.
GSE127472	31061481	5/8/19	Determining Cell Type Abundance And Expression From Bulk Tissues With Digital Cytometry.
GSE127683	31033441	4/30/19	Transcriptomic And Epigenetic Regulation Of Hair Cell Regeneration In The Mouse Utricle And Its Potentiation By Atoh1.
GSE127774	32424074	5/20/20	Single-Cell-Resolution Transcriptome Map Of Human, Chimpanzee, Bonobo, And Macaque Brains.
GSE128003	33078414	10/21/20	Single-Cell Molecular Profiling Provides A High-Resolution Map Of Basophil And Mast Cell Development.
GSE128074	33078414	10/21/20	Single-Cell Molecular Profiling Provides A High-Resolution Map Of Basophil And Mast Cell Development.
GSE128147	31448339	8/27/19	Single-cell transcriptome analysis of CD8 + T-cell memory inflation
GSE128423	31130381	5/28/19	A Cellular Taxonomy Of The Bone Marrow Stroma In Homeostasis And Leukemia.
GSE128531	31010835	4/24/19	Single-Cell Lymphocyte Heterogeneity In Advanced Cutaneous T-Cell Lymphoma Skin Tumors.
GSE128553	32367190	5/6/20	Dissecting The Initiation Of Female Meiosis In The Mouse At Single-Cell Resolution.
GSE128639	31178118	6/11/19	Comprehensive Integration Of Single-Cell Data.
GSE128691	31466525	8/31/19	Light-Induced Injury In Mouse Embryos Revealed By Single-Cell Rna Sequencing.
GSE128759	32822583	8/22/20	Single-Cell Analysis Of Neonatal Hsc Ontogeny Reveals Gradual And Uncoordinated Transcriptional Reprogramming That Begins Before Birth.
GSE128761	32822583	8/22/20	Single-Cell Analysis Of Neonatal Hsc Ontogeny Reveals Gradual And Uncoordinated Transcriptional Reprogramming That Begins Before Birth.
GSE128854	31061494	5/8/19	A Single-Cell Atlas Of Mouse Brain Macrophages Reveals Unique Transcriptional Identities Shaped By Ontogeny And Tissue Environment.
GSE128879	32457298	5/28/20	Single Cell Transcriptomics Reveals Opioid Usage Evokes Widespread Suppression Of Antiviral Gene Program.
GSE128892		7/1/19	A hierarchy of migratory keratinocytes maintains the tympanic membrane
GSE128934	31693907	11/7/19	Doubletdecon: Deconvoluting Doublets From Single-Cell Rna-Sequencing Data.
GSE129087	31142839	5/31/19	Distinct Fibroblast Subsets Drive Inflammation And Damage In Arthritis.
GSE129096	32001747	2/1/20	Systematic Comparison Of High-Throughput Single-Cell And Single-Nucleus Transcriptomes During Cardiomyocyte Differentiation.
GSE129150	31209379	6/19/19	Sensory Lesioning Induces Microglial Synapse Elimination Via Adam10 And Fractalkine Signaling.
GSE129256	32444476	5/24/20	IRF5 guides monocytes toward an inflammatory CD11c+ macrophage phenotype and promotes intestinal inflammation
GSE129363	32066997	2/19/20	Single-Cell Analysis Of Human Adipose Tissue Identifies Depot And Disease Specific Cell Types.
GSE129857	33419925	1/10/21	Permissive Selection Followed By Affinity-Based Proliferation Of Gc Light Zone B Cells Dictates Cell Fate And Ensures Clonal Breadth.
GSE130001	32111252	3/1/20	A Reference Profile-Free Deconvolution Method To Infer Cancer Cell-Intrinsic Subtypes And Tumor-Type-Specific Stromal Profiles.
GSE130019	32049009	2/13/20	Transcriptional Programs Define Intratumoral Heterogeneity Of Ewing Sarcoma At Single-Cell Resolution.
GSE130020	32049009	2/13/20	Transcriptional Programs Define Intratumoral Heterogeneity Of Ewing Sarcoma At Single-Cell Resolution.
GSE130021	32049009	2/13/20	Transcriptional Programs Define Intratumoral Heterogeneity Of Ewing Sarcoma At Single-Cell Resolution.
GSE130023	32049009	2/13/20	Transcriptional Programs Define Intratumoral Heterogeneity Of Ewing Sarcoma At Single-Cell Resolution.
GSE130025	32049009	2/13/20	Transcriptional Programs Define Intratumoral Heterogeneity Of Ewing Sarcoma At Single-Cell Resolution.
GSE130070	33037057	10/11/20	Deconvolution Of Monocyte Responses In Inflammatory Bowel Disease Reveals An Il-1 Cytokine Network That Regulates Il-23 In Genetic And Acquired Il-10
GSE130073	31155493	6/4/19	Modeling Steatohepatitis In Humans With Pluripotent Stem Cell-Derived Organoids.
GSE130148	31209336	6/19/19	A Cellular Census Of Human Lungs Identifies Novel Cell States In Health And In Asthma.
GSE130151	31462300	8/30/19	High-Resolution Analysis Of Germ Cells From Men With Sex Chromosomal Aneuploidies Reveals Normal Transcriptome But Impaired Imprinting.
GSE130212	32716582	7/28/20	Single-Cell Rna Sequencing Reveals The Landscape Of Early Female Germ Cell Development.
GSE130318	31317052	7/19/19	Single-Cell Rna-Seq Analysis Identifies A Putative Epithelial Stem Cell Population In Human Primary Prostate Cells In Monolayer And Organoid Culture Condi
GSE130430	31477722	9/4/19	Heterogeneity Of Human Bone Marrow And Blood Natural Killer Cells Defined By Single-Cell Transcriptome.
GSE130473	31350390	7/28/19	Single Cell Analysis Of Human Foetal Liver Captures The Transcriptional Profile Of Hepatobiliary Hybrid Progenitors.
GSE130606	31118232	5/24/19	Single Cell Analysis Of The Developing Mouse Kidney Provides Deeper Insight Into Marker Gene Expression And Ligand-Receptor Crosstalk.
GSE130626	31902528	1/7/20	Trem2 Regulates Microglial Cholesterol Metabolism Upon Chronic Phagocytic Challenge.
GSE130636	31075224	5/11/19	Molecular Characterization Of Foveal Versus Peripheral Human Retina By Single-Cell Rna Sequencing.
GSE130756	32977829	9/27/20	A Single-Cell Survey Of Cellular Hierarchy In Acute Myeloid Leukemia.
GSE130888	31167927	6/7/19	Inhibition Of Hyperglycolysis In Mesothelial Cells Prevents Peritoneal Fibrosis.
GSE130919	31522976	9/17/19	Lgr5 And Col22A1 Mark Progenitor Cells In The Lineage Toward Juvenile Articular Chondrocytes.
GSE130973	32327715	4/25/20	Single-Cell Transcriptomes Of The Human Skin Reveal Age-Related Loss Of Fibroblast Priming.
GSE131038	31128961	5/28/19	Polychromic Reporter Mice Reveal Unappreciated Innate Lymphoid Cell Progenitor Heterogeneity And Elusive Ilc3 Progenitors In Bone Marrow.
GSE131181	31201182	6/16/19	A cellular atlas of Pitx2- dependent cardiac development
GSE131365	31149730	6/1/19	Single-Cell Transcriptomes Of Murine Bone Marrow Stromal Cells Reveal Niche-Associated Heterogeneity.
GSE131391	31844660	12/18/19	Characterizing Smoking-Induced Transcriptional Heterogeneity In The Human Bronchial Epithelium At Single-Cell Resolution.
GSE131498	32685006	7/21/20	Single-Cell Transcriptome Profiling Reveals Dermal And Epithelial Cell Fate Decisions During Embryonic Hair Follicle Development.
GSE131535	31606264	10/14/19	Tcf-1-Centered Transcriptional Network Drives An Effector Versus Exhausted Cd8¬†T Cell-Fate Decision.
GSE131629	32194870	3/21/20	Alginate Oligosaccharides Improve Germ Cell Development And Testicular Microenvironment To Rescue Busulfan Disrupted Spermatogenesis.
GSE131630	31900405	1/5/20	Single-Cell Rna Sequencing Analysis Reveals Alginate Oligosaccharides Preventing Chemotherapy-Induced Mucositis.
GSE131734	31590918	10/9/19	Single-Cell Rna-Seq Analysis Of Mesp1-Induced Skeletal Myogenic Development.
GSE131776	31359001	7/31/19	Atheroprotective Roles Of Smooth Muscle Cell Phenotypic Modulation And The Tcf21 Disease Gene As Revealed By Single-Cell Analysis.
GSE131777	31359001	7/31/19	Atheroprotective Roles Of Smooth Muscle Cell Phenotypic Modulation And The Tcf21 Disease Gene As Revealed By Single-Cell Analysis.
GSE131778	31359001	7/31/19	Atheroprotective Roles Of Smooth Muscle Cell Phenotypic Modulation And The Tcf21 Disease Gene As Revealed By Single-Cell Analysis.
GSE131907	32385277	5/10/20	Single-Cell Rna Sequencing Demonstrates The Molecular And Cellular Reprogramming Of Metastatic Lung Adenocarcinoma.
GSE131928	31327527	7/23/19	An Integrative Model Of Cellular States, Plasticity, And Genetics For Glioblastoma.
GSE132042	32669714	7/15/20	A single-cell transcriptomic atlas characterizes ageing tissues in the mouse
GSE132080	31932729	1/15/20	Titrating Gene Expression Using Libraries Of Systematically Attenuated Crispr Guide Rnas.
GSE132143	32972203	9/26/20	Single-Cell Rna Sequencing Analysis Reveals A Crucial Role For Cthrc1 (Collagen Triple Helix Repeat Containing 1) Cardiac Fibroblasts After Myocardial Infar
GSE132164	32923584	9/15/20	Single-Cell Transcriptomics Identifies Multiple Pathways Underlying Antitumor Function Of Tcr- And Cd8ŒëŒ≤-Engineered Human Cd4
GSE132229	31843893	12/18/19	Comprehensive Analysis Of A Mouse Model Of Spontaneous Uveoretinitis Using Single-Cell Rna Sequencing.
GSE132257	32451460	5/27/20	Lineage-Dependent Gene Expression Programs Influence The Immune Landscape Of Colorectal Cancer.
GSE132274	31851941	12/19/19	The Cellular Diversity And Transcription Factor Code Of Drosophila Enteroendocrine Cells.
GSE132300	31332193	7/25/19	Predicting Bacterial Infection Outcomes Using Single Cell Rna-Sequencing Analysis Of Human Immune Cells.
GSE132364	31160419	6/5/19	A Transcriptomic Roadmap To Œë- And Œí-Cell Differentiation In The Embryonic Pancreas.
GSE132465	32451460	5/27/20	Lineage-Dependent Gene Expression Programs Influence The Immune Landscape Of Colorectal Cancer.
GSE132504	31152001	6/4/19	Neuro-Mesodermal Progenitors (Nmps): A Comparative Study Between Pluripotent Stem Cells And Embryo-Derived Populations.
GSE132509	32415257	5/18/20	Single-Cell Analysis Of Childhood Leukemia Reveals A Link Between Developmental States And Ribosomal Protein Expression As A Source Of Intra-Individua
GSE132573	31685531	11/7/19	Developmental And Cellular Age Direct Conversion Of Cd4+ T Cells Into RorŒ≥+ Or Helios+ Colon Treg Cells.

GSE132608	32066983	2/19/20	Spatiotemporal Single-Cell Analysis Of Gene Expression In The Mouse Suprachiasmatic Nucleus.
GSE132642	31751331	11/22/19	Single-Cell Transcriptomics Of The Naked Mole-Rat Reveals Unexpected Features Of Mammalian Immunity.
GSE132672	31996853	1/31/20	Cell Stress In Cortical Organoids Impairs Molecular Subtype Specification.
GSE132730	32499648	6/6/20	Molecular Design Of Hypothalamus Development.
GSE132757	32732423	8/1/20	Allele-Specific Open Chromatin In Human Ipsc Neurons Elucidates Functional Disease Variants.
GSE132771	32317643	4/23/20	Collagen-Producing Lung Cell Atlas Identifies Multiple Subsets With Distinct Localization And Relevance To Fibrosis.
GSE132867	31623513	10/19/19	Transcriptomic And Single-Cell Analysis Of The Murine Parotid Gland.
GSE132880	31162546	6/5/19	Single-Cell Transcriptome Analyses Reveal Novel Targets Modulating Cardiac Neovascularization By Resident Endothelial Cells Following Myocardial Infarct
GSE133015	31399586	8/11/19	Wwp2 Regulates Pathological Cardiac Fibrosis By Modulating Smad2 Signaling.
GSE133204	31455604	8/29/19	Single Cell Transcriptomic Landscapes Of Pattern Formation, Proliferation And Growth In
GSE133345	32499656	6/6/20	Deciphering Human Macrophage Development At Single-Cell Resolution.
GSE133382	31784286	12/1/19	Single-Cell Profiles Of Retinal Ganglion Cells Differing In Resilience To Injury Reveal Neuroprotective Genes.
GSE133449	31871141	12/25/19	Single-Cell Rna-Seq Analysis Identifies Meniscus Progenitors And Reveals The Progression Of Meniscus Degeneration.
GSE133486	31644425	10/24/19	Single Cell Analysis Reveals Immune Cell-Adipocyte Crosstalk Regulating The Transcription Of Thermogenic Adipocytes.
GSE133531	31768071	11/27/19	Stalled Developmental Programs At The Root Of Pediatric Brain Tumors.
GSE133535	32051003	2/14/20	Robustness And Applicability Of Transcription Factor And Pathway Analysis Tools On Single-Cell Rna-Seq Data.
GSE133540	32051003	2/14/20	Robustness And Applicability Of Transcription Factor And Pathway Analysis Tools On Single-Cell Rna-Seq Data.
GSE133689	31827285	12/13/19	Multi-Omics Profiling Of Mouse Gastrulation At Single-Cell Resolution.
GSE133699	31471106	9/1/19	Oncolytic Viruses Engineered To Enforce Leptin Expression Reprogram Tumor-Infiltrating T Cell Metabolism And Promote Tumor Clearance.
GSE133725	31827285	12/13/19	Multi-Omics Profiling Of Mouse Gastrulation At Single-Cell Resolution.
GSE133912	31527803	9/19/19	A Repeated Molecular Architecture Across Thalamic Pathways.
GSE133948	33138772	11/4/20	Functional Module Detection Through Integration Of Single-Cell Rna Sequencing Data With Protein-Protein Interaction Networks.
GSE134174	32427931	5/20/20	Dissecting The Cellular Specificity Of Smoking Effects And Reconstructing Lineages In The Human Airway Epithelium.
GSE134355	32214235	3/28/20	Construction Of A Human Cell Landscape At Single-Cell Level.
GSE134520	31067475	5/9/19	Dissecting The Single-Cell Transcriptome Network Underlying Gastric Premalignant Lesions And Early Gastric Cancer.
GSE134571	31511693	9/13/19	Controlled Modelling Of Human Epiblast And Amnion Development Using Stem Cells.
GSE134722	31746739	11/21/19	Single cell transcriptome atlas of the Drosophila larval brain
GSE134809	31474370	9/3/19	Single-Cell Analysis Of Crohn'S Disease Lesions Identifies A Pathogenic Cellular Module Associated With Resistance To Anti-Tnf Therapy.
GSE134918	32579931	6/25/20	Single-Cell Profiling And Scope-Seq Reveal Lineage Dynamics Of Adult Ventricular-Subventricular Zone Neurogenesis And Notum As A Key Regulator.
GSE135060	32710103	7/28/20	Cell Surface Protein Mrnas Show Differential Transcription In Pyramidal And Fast-Spiking Cells As Revealed By Single-Cell Sequencing.
GSE135132	31647409	10/28/19	Single-Cell Transcriptomes And Whole-Brain Projections Of Serotonin Neurons In The Mouse Dorsal And Median Raphe Nuclei.
GSE135133	31995762	1/30/20	Integration Of Eqtl And A Single-Cell Atlas In The Human Eye Identifies Causal Genes For Age-Related Macular Degeneration.
GSE135185	33526922	2/3/21	Cns Fibroblasts Form A Fibrotic Scar In Response To Immune Cell Infiltration.
GSE135194	32556286	6/20/20	Sequencing Of Rna In Single Cells Reveals A Distinct Transcriptome Signature Of Hematopoiesis In Gata2 Deficiency.
GSE135437	31740814	11/20/19	Mapping Microglia States In The Human Brain Through The Integration Of High-Dimensional Techniques.
GSE135663	32656538	7/14/20	Profiling APOL1 Nephropathy Risk Variants in Genome-Edited Kidney Organoids with Single-Cell Transcriptomics
GSE135703	32116546	3/3/20	Characterizing Adult Cochlear Supporting Cell Transcriptional Diversity Using Single-Cell Rna-Seq: Validation In The Adult Mouse And Translational Implicati
GSE135710	32142650	3/7/20	Complement Signals Determine Opposite Effects Of B Cells In Chemotherapy-Induced Immunity.
GSE135769	31878887	12/28/19	Exploring The Changing Landscape Of Cell-To-Cell Variation After Ctcf Knockdown Via Single Cell Rna-Seq.
GSE135922	31712411	11/13/19	Single-Cell Transcriptomics Of The Human Retinal Pigment Epithelium And Choroid In Health And Macular Degeneration.
GSE136001	33608526	2/21/21	Single-Cell Rna Sequencing Reveals Functional Heterogeneity Of Glioma-Associated Brain Macrophages.
GSE136103	31597160	10/10/19	Resolving The Fibrotic Niche Of Human Liver Cirrhosis At Single-Cell Level.
GSE136206	31730857	11/16/19	B Cells And T Follicular Helper Cells Mediate Response To Checkpoint Inhibitors In High Mutation Burden Mouse Models Of Breast Cancer.
GSE136229	32066997	2/19/20	Single-Cell Analysis Of Human Adipose Tissue Identifies Depot And Disease Specific Cell Types.
GSE136353	31630146	10/21/19	Single-Cell Sequencing Reveals The Relationship Between Phenotypes And Genotypes Of Klinefelter Syndrome.
GSE136394	31484655	9/6/19	Single-Cell Transcriptome Analysis Reveals Gene Signatures Associated With T-Cell Persistence Following Adoptive Cell Therapy.
GSE136611	31651061	10/28/19	Single-Cell Study Of Neural Stem Cells Derived From Human Ipscs Reveals Distinct Progenitor Populations With Neurogenic And Gliogenic Potential.
GSE136805	31802004	12/6/19	C-Jun Overexpression In Car T Cells Induces Exhaustion Resistance.
GSE137082	31784108	12/1/19	Type I Interferon Signaling Disrupts The Hepatic Urea Cycle And Alters Systemic Metabolism To Suppress T Cell Function.
GSE137165	31629685	10/21/19	Single-Cell Analysis Reveals Regulatory Gene Expression Dynamics Leading To Lineage Commitment In Early T Cell Development.
GSE137398	31784286	12/1/19	Single-Cell Profiles Of Retinal Ganglion Cells Differing In Resilience To Injury Reveal Neuroprotective Genes.
GSE137525	31908317	1/8/20	Single Cell Sequencing Of Radial Glia Progeny Reveals The Diversity Of Newborn Neurons In The Adult Zebrafish Brain.
GSE137537	31653841	10/28/19	Single-Cell Transcriptomic Atlas Of The Human Retina Identifies Cell Types Associated With Age-Related Macular Degeneration.
GSE137540	32719519	7/29/20	Single-Cell Transcriptome Profiling Reveals Neutrophil Heterogeneity In Homeostasis And Infection.
GSE137799		9/26/19	Single-cell time-series mapping of cell fate trajectories reveals an expanded developmental potential for human PSC-derived distal lung progenitors
GSE137828	31784286	12/1/19	Single-Cell Profiles Of Retinal Ganglion Cells Differing In Resilience To Injury Reveal Neuroprotective Genes.
GSE137863	31784286	12/1/19	Single-Cell Profiles Of Retinal Ganglion Cells Differing In Resilience To Injury Reveal Neuroprotective Genes.
GSE137869	32109414	2/29/20	Caloric Restriction Reprograms The Single-Cell Transcriptional Landscape Of Rattus Norvegicus Aging.
GSE138266	31937773	1/16/20	Integrated Single Cell Analysis Of Blood And Cerebrospinal Fluid Leukocytes In Multiple Sclerosis.
GSE138302	33226180	11/24/20	A Robust Culture System To Generate Neural Progenitors With Gliogenic Competence From Clinically Relevant Induced Pluripotent Stem Cells For Treatmer
GSE138536	31974247	1/25/20	Single-Cell Transcriptional Diversity Is A Hallmark Of Developmental Potential.
GSE138585	31866069	12/24/19	Progressive Pulmonary Fibrosis Is Caused By Elevated Mechanical Tension On Alveolar Stem Cells.
GSE138794	31554641	9/27/19	The Phenotypes Of Proliferating Glioblastoma Cells Reside On A Single Axis Of Variation.
GSE138826	32248062	4/6/20	Temporal Dynamics And Heterogeneity Of Cell Populations During Skeletal Muscle Regeneration.
GSE138852	31768052	11/27/19	A Single-Cell Atlas Of Entorhinal Cortex From Individuals With Alzheimer'S Disease Reveals Cell-Type-Specific Gene Expression Regulation.
GSE139079	32049047	2/13/20	The Repertoire Of Serous Ovarian Cancer Non-Genetic Heterogeneity Revealed By Single-Cell Sequencing Of Normal Fallopian Tube Epithelial Cells.
GSE139103	32994417	10/1/20	Satellite Glial Cells Promote Regenerative Growth In Sensory Neurons.
GSE139107	32571916	6/24/20	Cell Profiling Of Mouse Acute Kidney Injury Reveals Conserved Cellular Responses To Injury.
GSE139248	31937348	1/16/20	Dendro: Genetic Heterogeneity Profiling And Subclone Detection By Single-Cell Rna Sequencing.
GSE139249	31801909	12/6/19	Discovery Of Specialized Nk Cell Populations Infiltrating Human Melanoma Metastases.
GSE139324	31924475	1/12/20	Immune Landscape Of Viral- And Carcinogen-Driven Head And Neck Cancer.
GSE139448	32004492	2/1/20	Adult Human Glioblastomas Harbor Radial Glia-Like Cells.
GSE139598	31894150	1/3/20	Prevention Of Tuberculosis In Macaques After Intravenous Bcg Immunization.
GSE139827	31883835	12/31/19	Arterial Sca1+ Vascular Stem Cells Generate De Novo Smooth Muscle for Artery Repair and Regeneration
GSE139829	31980621	1/26/20	Single-Cell Analysis Reveals New Evolutionary Complexity In Uveal Melanoma.
GSE140228	31675496	11/2/19	Landscape And Dynamics Of Single Immune Cells In Hepatocellular Carcinoma.
GSE140312	32054662	2/15/20	Comparative Single-Cell Rna Sequencing (Scrna-Seq) Reveals Liver Metastasis-Specific Targets In A Patient With Small Intestinal Neuroendocrine Cancer.
GSE140405	31924806	1/12/20	Generation Of Mesenchyme Free Intestinal Organoids From Human Induced Pluripotent Stem Cells.
GSE140807	32179751	3/18/20	Dissecting The Early Steps Of Mll Induced Leukaemogenic Transformation Using A Mouse Model Of Aml.
GSE141445	33420488	1/10/21	Single-Cell Analysis Reveals Transcriptomic Remodellings In Distinct Cell Types That Contribute To Human Prostate Cancer Progression.
GSE141460	32663469	7/15/20	Single-Cell Rna-Seq Reveals Cellular Hierarchies And Impaired Developmental Trajectories In Pediatric Ependymoma.
GSE141634	32661339	7/15/20	Persistence Of A Regeneration-Associated, Transitional Alveolar Epithelial Cell State In Pulmonary Fibrosis.
GSE141701	33340458	12/20/20	A Progressive Somatic Cell Niche Regulates Germline Cyst Differentiation In The Drosophila Ovary.
GSE141776	32268097	4/9/20	Differentiation Paths Of Peyer'S Patch Lysodcs Are Linked To Sampling Site Positioning, Migration, And T Cell Priming.
GSE141946	31883794	12/31/19	A Patient-Derived Glioblastoma Organoid Model And Biobank Recapitulates Inter- And Intra-Tumoral Heterogeneity.
GSE141982	32105316	2/28/20	Ensemble Learning For Classifying Single-Cell Data And Projection Across Reference Atlases.
GSE142116	32217638	3/29/20	Organoid Cultures Of Early-Onset Colorectal Cancers Reveal Distinct And Rare Genetic Profiles.
GSE142200	33128445	11/1/20	System-Level Analyses Of Keystone Genes Required For Mammalian Tooth Development.
GSE142213	32330454	4/25/20	C/EbpŒ± And Gata-2 Mutations Induce Bilineage Acute Erythroid Leukemia Through Transformation Of A Neomorphic Neutrophil-Erythroid Progenitor.
GSE142267	31951107	1/18/20	Novel Alzheimer Risk Genes Determine The Microglia Response To Amyloid-Œí But Not To Tau Pathology.
GSE142356	33205894	11/19/20	The Transcriptome Dynamics Of Single Cells During The Cell Cycle.
GSE142564	32431172	5/21/20	Single-Cell Rna Sequencing To Dissect The Immunological Network Of Autoimmune Myocarditis.
GSE142585	32504559	6/7/20	Single-Cell Rna Sequencing Of Human, Macaque, And Mouse Testes Uncovers Conserved And Divergent Features Of Mammalian Spermatogenesis.
GSE142653	33077725	10/21/20	Single-Cell Transcriptomics Identifies Divergent Developmental Lineage Trajectories During Human Pituitary Development.

GSE142999	32735845	8/1/20	Differential Irf8 Transcription Factor Requirement Defines Two Pathways Of Dendritic Cell Development In Humans.
GSE143002	32735845	8/1/20	Differential Irf8 Transcription Factor Requirement Defines Two Pathways Of Dendritic Cell Development In Humans.
GSE143038	32059767	2/16/20	Single-Cell Transcriptomes Reveal Diverse Regulatory Strategies For Olfactory Receptor Expression And Axon Targeting.
GSE143158	32735845	8/1/20	Differential Irf8 Transcription Factor Requirement Defines Two Pathways Of Dendritic Cell Development In Humans.
GSE143363	31974170	1/25/20	Monocytic Subclones Confer Resistance To Venetoclax-Based Therapy In Patients With Acute Myeloid Leukemia.
GSE143437	32160558	3/12/20	Single-Cell Analysis Of The Muscle Stem Cell Hierarchy Identifies Heterotypic Communication Signals Involved In Skeletal Muscle Regeneration.
GSE143545	32912294	9/12/20	Starr-Seq Identifies Active, Chromatin-Masked, And Dormant Enhancers In Pluripotent Mouse Embryonic Stem Cells.
GSE143637	32402290	5/14/20	Iterative Single-Cell Analyses Define The Transcriptome Of The First Functional Hematopoietic Stem Cells.
GSE143704	32624006	7/7/20	A Reference Single-Cell Transcriptomic Atlas Of Human Skeletal Muscle Tissue Reveals Bifurcated Muscle Stem Cell Populations.
GSE143758	32341542	4/29/20	Disease-Associated Astrocytes In Alzheimer'S Disease And Aging.
GSE143877	32426372	5/20/20	A Single-Cell Transcriptional Roadmap Of The Mouse And Human Lymph Node Lymphatic Vasculature.
GSE143949	33158872	11/8/20	Transcriptional Priming As A Conserved Mechanism Of Lineage Diversification In The Developing Mouse And Human Neocortex.
GSE144271	32521264	6/11/20	Prmt1-P53 Pathway Controls Epicardial Emt And Invasion.
GSE144430	32271902	4/10/20	Diversity Of Peripheral Blood Human Nk Cells Identified By Single-Cell Rna Sequencing.
GSE144444	32152318	3/11/20	Functional Hypoxia Drives Neuroplasticity And Neurogenesis Via Brain Erythropoietin.
GSE144730	32058160	2/15/20	Elevated Circulating Th2 But Not Group 2 Innate Lymphoid Cell Responses Characterize Canine Atopic Dermatitis.
GSE144735	32451460	5/27/20	Lineage-Dependent Gene Expression Programs Influence The Immune Landscape Of Colorectal Cancer.
GSE144980	32568072	6/23/20	A Single-Cell Transcriptomic And Anatomic Atlas Of Mouse Dorsal Raphe
GSE145013	32640237	7/9/20	Single-Cell And Population Transcriptomics Reveal Pan-Epithelial Remodeling In Type 2-High Asthma.
GSE145137	32460812	5/29/20	Single-Cell Rna Sequencing Reveals The Tumor Microenvironment And Facilitates Strategic Choices To Circumvent Treatment Failure In A Chemorefractory
GSE145241	32663195	7/15/20	Hemolysis Transforms Liver Macrophages Into Antiinflammatory Erythrophagocytes.
GSE145281	32405063	5/15/20	High Systemic And Tumor-Associated Il-8 Correlates With Reduced Clinical Benefit Of Pd-L1 Blockade.
GSE145307	32315970	4/22/20	Transcriptional And Clonal Characterization Of B Cell Plasmablast Diversity Following Primary And Secondary Natural Denv Infection.
GSE145443	32729827	7/31/20	An Atlas Of Cell Types In The Mouse Epididymis And Vas Deferens.
GSE145502	32632238	7/8/20	Zipseq: Barcoding For Real-Time Mapping Of Single Cell Transcriptomes.
GSE145531	33763704	3/26/21	Single-Cell Transcriptomics Dissects Hematopoietic Cell Destruction And T Cell Engagement In Aplastic Anemia.
GSE145539	33241896	11/27/20	Directed Induction Of Alveolar Type I Cells Derived From Pluripotent Stem Cells Via Wnt Signaling Inhibition.
GSE145638	32302558	4/18/20	Transcriptome Dynamics Of Hematopoietic Stem Cell Formation Revealed Using A Combinatorial Runx1 And Ly6A Reporter System.
GSE145688	33239393	11/27/20	Kidney Single-Cell Transcriptomes Predict Spatial Corticomedullary Gene Expression And Tissue Osmolality Gradients.
GSE145726	32367046	5/6/20	In Vitro Capture And Characterization Of Embryonic Rosette-Stage Pluripotency Between Naive And Primed States.
GSE145861	32497356	6/5/20	Urethral Luminal Epithelia Are Castration-Insensitive Cells Of The Proximal Prostate.
GSE145926	32398875	5/14/20	Single-Cell Landscape Of Bronchoalveolar Immune Cells In Patients With Covid-19.
GSE145928	32497356	6/5/20	Urethral Luminal Epithelia Are Castration-Insensitive Cells Of The Proximal Prostate.
GSE146026	32572264	6/24/20	A Single-Cell Landscape Of High-Grade Serous Ovarian Cancer.
GSE146040	32339165	4/28/20	A Single-Cell Atlas Of Adult Drosophila Ovary Identifies Transcriptional Programs And Somatic Cell Lineage Regulating Oogenesis.
GSE146046	32532832	6/14/20	Single-Cell Transcriptomic Analysis Of Allergen-Specific T Cells In Allergy And Asthma.
GSE146115	33531041	2/4/21	Clonal Evolution In Liver Cancer At Single-Cell And Single-Variant Resolution.
GSE146123	32968047	9/25/20	Dental Cell Type Atlas Reveals Stem And Differentiated Cell Types In Mouse And Human Teeth.
GSE146194	32231336	4/2/20	Combinatorial Single-Cell Crispr Screens By Direct Guide Rna Capture And Targeted Sequencing.
GSE146216	32151196	3/11/20	Deep Phenotyping By Mass Cytometry And Single-Cell Rna-Sequencing Reveals Lyn-Regulated Signaling Profiles Underlying Monocyte Subset Heterogeneity
GSE146221	32290418	4/16/20	Reconstruction Of Ewing Sarcoma Developmental Context From Mass-Scale Transcriptomics Reveals Characteristics Of Ewsr1-Fli1 Permissibility.
GSE146244	32351546	5/1/20	Single-Cell Transcriptomes Reveal A Complex Cellular Landscape In The Middle Ear And Differential Capacities For Acute Response To Infection.
GSE146264	33309739	12/15/20	Single-Cell Rna Sequencing Of Psoriatic Skin Identifies Pathogenic Tc17 Cell Subsets And Reveals Distinctions Between Cd8
GSE146456	33304758	12/12/20	Deficiency Of Sting Signaling In Embryonic Cerebral Cortex Leads To Neurogenic Abnormalities And Autistic-Like Behaviors.
GSE146637	32728211	7/31/20	Mechanisms Of Stretch-Mediated Skin Expansion At Single-Cell Resolution.
GSE146672	32327659	4/25/20	Deletion Of Topoisomerase 1 In Excitatory Neurons Causes Genomic Instability And Early Onset Neurodegeneration.
GSE146740	33432227	1/13/21	RorŒ± Is A Critical Checkpoint For T Cell And Ilc2 Commitment In The Embryonic Thymus.
GSE146741	33432227	1/13/21	RorŒ± Is A Critical Checkpoint For T Cell And Ilc2 Commitment In The Embryonic Thymus.
GSE146771	32302573	4/18/20	Single-Cell Analyses Inform Mechanisms Of Myeloid-Targeted Therapies In Colon Cancer.
GSE146799	32407674	5/15/20	High-Resolution Mrna And Secretome Atlas Of Human Enteroendocrine Cells.
GSE146811	32355025	5/2/20	Regenerative Potential Of Prostate Luminal Cells Revealed By Single-Cell Analysis.
GSE146912	32651223	7/12/20	Single-Cell Transcriptome Profiling Of The Kidney Glomerulus Identifies Key Cell Types And Reactions To Injury.
GSE147100	32597756	7/1/20	A Tgfbr1/Snai1-Dependent Developmental Module At The Core Of Vertebrate Axial Elongation.
GSE147143	32671793	7/17/20	Single-Cell Analysis Reveals Bronchoalveolar Epithelial Dysfunction In Covid-19 Patients.
GSE147202	32375897	5/8/20	Single-Cell Rna-Seq With Spike-In Cells Enables Accurate Quantification Of Cell-Specific Drug Effects In Pancreatic Islets.
GSE147298	33184221	11/14/20	Bmp Signaling: At The Gate Between Activated Melanocyte Stem Cells And Differentiation.
GSE147349	32375022	5/7/20	A Nutrient-Sensing Transition At Birth Triggers Glucose-Responsive Insulin Secretion.
GSE147390	34111027	6/11/21	Single-Cell Rna Sequencing Of Human Femoral Head
GSE147581	33340713	12/20/20	Single-Cell Transcriptomics Reveals Zone-Specific Alterations Of Liver Sinusoidal Endothelial Cells In Cirrhosis.
GSE147668	32484158	6/3/20	Diverse Homeostatic And Immunomodulatory Roles Of Immune Cells In The Developing Mouse Lung At Single Cell Resolution.
GSE147693	32887883	9/6/20	Pharmacologically Reversible Zonation-Dependent Endothelial Cell Transcriptomic Changes With Neurodegenerative Disease Associations In The Aged Brair
GSE147863	32333836	4/26/20	Inhibition Of Sars-Cov-2 Infections In Engineered Human Tissues Using Clinical-Grade Soluble Human Ace2.
GSE147883	32375047	5/7/20	Interstitial Cell Remodeling Promotes Aberrant Adipogenesis In Dystrophic Muscles.
GSE147989	32601337	7/1/20	Targeting Cd70 With Cusatuzumab Eliminates Acute Myeloid Leukemia Stem Cells In Patients Treated With Hypomethylating Agents.
GSE148063	33548738	2/7/21	Single-Cell Transcriptional Analysis Reveals Developmental Stage-Dependent Changes In Retinal Progenitors In The Murine Early Optic Vesicle.
GSE148077	32555229	6/20/20	Cell Atlas Of The Human Fovea And Peripheral Retina.
GSE148127	33264626	12/3/20	Multi-Modal Single-Cell Analysis Reveals Brain Immune Landscape Plasticity During Aging And Gut Microbiota Dysbiosis.
GSE148190	32539073	6/17/20	Layilin Augments Integrin Activation To Promote Antitumor Immunity.
GSE148367	32377875	5/8/20	An Organoid Model To Assay The Role Of Cftr In The Human Epididymis Epithelium.
GSE148506	33149301	11/6/20	Paracrine Signalling By Cardiac Calcitonin Controls Atrial Fibrogenesis And Arrhythmia.
GSE148665	32379315	5/8/20	Brem-Sc: A Bayesian Random Effects Mixture Model For Joint Clustering Single Cell Multi-Omics Data.
GSE148729	33585804	2/16/21	Transcriptomic Profiling Of Sars-Cov-2 Infected Human Cell Lines Identifies Hsp90 As Target For Covid-19 Therapy.
GSE148829	32413319	5/28/20	SARS-CoV-2 Receptor ACE2 Is an Interferon-Stimulated Gene in Human Airway Epithelial Cells and Is Detected in Specific Cell Subsets across Tissues
GSE148842	33975634		Deconvolution of cell type-specific drug responses in human tumor tissue with single-cell RNA-seq
GSE148882	32497523	6/5/20	m6A Modification Prevents Formation of Endogenous Double-Stranded RNAs and Deleterious Innate Immune Responses during Hematopoietic Developmer
GSE148946	32895383	9/9/20	Transcriptomic Analysis Links Diverse Hypothalamic Cell Types To Fibroblast Growth Factor 1-Induced Sustained Diabetes Remission.
GSE149100	32531351	6/13/20	Bulk And Single-Cell Gene Expression Analyses Reveal Aging Human Choriocapillaris Has Pro-Inflammatory Phenotype.
GSE149224	32846134	8/28/20	Single-Cell Transcriptome Analysis Of Colon Cancer Cell Response To 5-Fluorouracil-Induced Dna Damage.
GSE149301	32769974	8/10/20	Single-Cell Analysis Uncovers Fibroblast Heterogeneity And Criteria For Fibroblast And Mural Cell Identification And Discrimination.
GSE149512	33173058	11/12/20	Single-Cell Analysis Of Developing And Azoospermia Human Testicles Reveals Central Role Of Sertoli Cells.
GSE149629	33372178	12/30/20	Single-Cell Rna Sequencing Reveals Regulation Of Fetal Ovary Development In The Monkey (Macaca Fascicularis).
GSE149656	32769974	8/10/20	Single-Cell Analysis Uncovers Fibroblast Heterogeneity And Criteria For Fibroblast And Mural Cell Identification And Discrimination.
GSE149689	32651212	7/12/20	Immunophenotyping Of Covid-19 And Influenza Highlights The Role Of Type I Interferons In Development Of Severe Covid-19.
GSE149859	32769974	8/10/20	Single-Cell Analysis Uncovers Fibroblast Heterogeneity And Criteria For Fibroblast And Mural Cell Identification And Discrimination.
GSE150132	33097590	10/25/20	A distinct GM-CSF + T helper cell subset requires T-bet to adopt a TH1 phenotype and promote neuroinflammation
GSE150219	32634398	7/8/20	Single-Cell Transcriptomic Analyses Of The Developing Meninges Reveal Meningeal Fibroblast Diversity And Function.
GSE150289	32769974	8/10/20	Single-Cell Analysis Uncovers Fibroblast Heterogeneity And Criteria For Fibroblast And Mural Cell Identification And Discrimination.
GSE150338	33769948	3/27/21	Targeted Single-Cell Rna-Seq Identifies Minority Cell Types Of Kidney Distal Nephron.
GSE150580	33378681	12/31/20	Aging-Associated Alterations In Mammary Epithelia And Stroma Revealed By Single-Cell Rna Sequencing.
GSE150660	32675368	7/18/20	Cancer Cells Deploy Lipocalin-2 To Collect Limiting Iron In Leptomeningeal Metastasis.
GSE150672	33053333	10/15/20	Second-Strand Synthesis-Based Massively Parallel Scrna-Seq Reveals Cellular States And Molecular Features Of Human Inflammatory Skin Pathologies.
GSE150728	32514174	6/10/20	A Single-Cell Atlas Of The Peripheral Immune Response In Patients With Severe Covid-19.
GSE150825	33750785	3/23/21	Comprehensive Single-Cell Sequencing Reveals The Stromal Dynamics And Tumor-Specific Characteristics In The Microenvironment Of Nasopharyngeal Carc
GSE150861	32764665	8/9/20	Single-Cell Analysis Of Two Severe Covid-19 Patients Reveals A Monocyte-Associated And Tocilizumab-Responding Cytokine Storm.

GSE150903	32527923	6/13/20	Human Cns Barrier-Forming Organoids With Cerebrospinal Fluid Production.
GSE150930	33194728	11/17/20	Evolution Of Advanced Chronic Lymphoid Leukemia Unveiled By Single-Cell Transcriptomics: A Case Report.
GSE151087	32610077	7/2/20	Transcriptional And Functional Analysis Of Cd1C
GSE151089	32610077	7/2/20	Transcriptional And Functional Analysis Of Cd1C
GSE151152	32814028	8/20/20	Paneth Cell-Derived Lysozyme Defines The Composition Of Mucolytic Microbiota And The Inflammatory Tone Of The Intestine.
GSE151244	32690951	7/22/20	The Activation Trajectory Of Plasmacytoid Dendritic Cells In Vivo During A Viral Infection.
GSE151245	32690951	7/22/20	The Activation Trajectory Of Plasmacytoid Dendritic Cells In Vivo During A Viral Infection.
GSE151246	32690951	7/22/20	The Activation Trajectory Of Plasmacytoid Dendritic Cells In Vivo During A Viral Infection.
GSE151247	32690951	7/22/20	The Activation Trajectory Of Plasmacytoid Dendritic Cells In Vivo During A Viral Infection.
GSE151630	32579887	6/25/20	Combinatorial Single-Cell Analyses Of Granulocyte-Monocyte Progenitor Heterogeneity Reveals An Early Uni-Potent Neutrophil Progenitor.
GSE151658	33448928	1/16/21	The Orchestrated Cellular And Molecular Responses Of The Kidney To Endotoxin Define A Precise Sepsis Timeline.
GSE151682	32579887	6/25/20	Combinatorial Single-Cell Analyses Of Granulocyte-Monocyte Progenitor Heterogeneity Reveals An Early Uni-Potent Neutrophil Progenitor.
GSE151723	32458983	5/28/20	Single-Cell Analysis Of The Gene Expression Effects Of Developmental Lead (Pb) Exposure On The Mouse Hippocampus.
GSE151735	32699019	7/24/20	Parallel Bimodal Single-Cell Sequencing Of Transcriptome And Chromatin Accessibility.
GSE151876	32946788	9/19/20	Multi-Layered Spatial Transcriptomics Identify Secretory Factors Promoting Human Hematopoietic Stem Cell Development.
GSE151877	32946788	9/19/20	Multi-Layered Spatial Transcriptomics Identify Secretory Factors Promoting Human Hematopoietic Stem Cell Development.
GSE152745	33133158	11/3/20	A Systems Biology Approach To Identifying A Master Regulator That Can Transform The Fast Growing Cellular State To A Slowly Growing One In Early Color
GSE152906	33591267	2/17/21	An Atlas Of Neural Crest Lineages Along The Posterior Developing Zebrafish At Single-Cell Resolution.
GSE152962	33173202	11/12/20	Unveiling The Heterogeneity Of Nkt Cells In The Liver Through Single Cell Rna Sequencing.
GSE152981	32731885	8/1/20	Gmm-Demux: Sample Demultiplexing, Multiplet Detection, Experiment Planning, And Novel Cell-Type Verification In Single Cell Sequencing.
GSE153056	33649593	3/3/21	Characterizing The Molecular Regulation Of Inhibitory Immune Checkpoints With Multimodal Single-Cell Screens.
GSE153487	33020472	10/7/20	Glioma-Derived Il-33 Orchestrates An Inflammatory Brain Tumor Microenvironment That Accelerates Glioma Progression.
GSE153647	32961131	9/23/20	Single-Cell Analyses Identify Brain Mural Cells Expressing Cd19 As Potential Off-Tumor Targets For Car-T Immunotherapies.
GSE153697	33558546	2/10/21	Single-Cell Profiling Identifies Pre-Existing Cd19-Negative Subclones In A B-All Patient With Cd19-Negative Relapse After Car-T Therapy.
GSE153701	33318148	12/16/20	Androgen Action In Cell Fate And Communication During Prostate Development At Single-Cell Resolution.
GSE154126	32739450	8/3/20	Single-Cell Transcriptomics Of Human Islet Ontogeny Defines The Molecular Basis Of Œí-Cell Dedifferentiation In T2D.
GSE154386	33513191	1/30/21	Temporally Integrated Single Cell Rna Sequencing Analysis Of Pbmc From Experimental And Natural Primary Human Denv-1 Infections.
GSE154579	33116143	10/30/20	Murine Interfollicular Epidermal Differentiation Is Gradualistic With Grhl3 Controlling Progression From Stem To Transition Cell States.
GSE154679	33047672	10/14/20	Dynamics Of Nevus Development Implicate Cell Cooperation In The Growth Arrest Of Transformed Melanocytes.
GSE154692	32883094	9/5/20	Anti-Inflammatory Actions Of Soluble Ninjurin-1 Ameliorate Atherosclerosis.
GSE154763	33545035	2/6/21	A Pan-Cancer Single-Cell Transcriptional Atlas Of Tumor Infiltrating Myeloid Cells.
GSE154773	32853177	8/28/20	Contribution Of Plasma Cells And B Cells To Hidradenitis Suppurativa Pathogenesis.
GSE155081	33718838	3/16/21	Single-Cell Rna-Seq Analysis Reveals Compartment-Specific Heterogeneity And Plasticity Of Microglia.
GSE155960	33907320	4/27/21	Single-cell sequencing of human white adipose tissue identifies new cell states in health and obesity
GSE155988	32680935	7/19/20	Prdm8 Regulates Pmn Progenitor Specification For Motor Neuron And Oligodendrocyte Fates By Modulating The Shh Signaling Response.
GSE156057	32888418	9/6/20	Osteopontin Expression Identifies A Subset Of Recruited Macrophages Distinct From Kupffer Cells In The Fatty Liver.
GSE156234	32868786	9/2/20	Single-Cell Rna Sequencing Uncovers Heterogenous Transcriptional Signatures In Macrophages During Efferocytosis.
GSE156644	33301706	12/11/20	Heterogeneous Bone-Marrow Stromal Progenitors Drive Myelofibrosis Via A Druggable Alarmin Axis.
GSE156793	33184181	11/14/20	A Human Cell Atlas Of Fetal Gene Expression.
GSE157204	33404500	1/7/21	Subcellular Sequencing Of Single Neurons Reveals The Dendritic Transcriptome Of Gabaergic Interneurons.
GSE157220	33128048	11/1/20	Pan-Cancer Single-Cell Rna-Seq Identifies Recurring Programs Of Cellular Heterogeneity.
GSE157278	33603736	2/20/21	Single-Cell Rna Sequencing Reveals The Expansion Of Cytotoxic Cd4
GSE157292	32970632	9/25/20	Single Cell Transcriptomics Of Mouse Kidney Transplants Reveals A Myeloid Cell Pathway For Transplant Rejection.
GSE157421	33754041	3/24/21	Deciphering The Autophagy Regulatory Network Via Single-Cell Transcriptome Analysis Reveals A Requirement For Autophagy Homeostasis In Spermatoger
GSE157743	33203734	11/19/20	The Lipogenic Regulator Srebp2 Induces Transferrin In Circulating Melanoma Cells And Suppresses Ferroptosis.
GSE157829	32954948	9/22/20	An Atlas Of Immune Cell Exhaustion In Hiv-Infected Individuals Revealed By Single-Cell Transcriptomics.
GSE158002	33376219	12/31/20	Hypr-Seq: Single-Cell Quantification Of Chosen Rnas Via Hybridization And Sequencing Of Dna Probes.
GSE158055		10/29/20	Large-scale single-cell analysis reveals critical immune characteristics of COVID-19 patients
GSE158724	34712959	6/2/21	Serial single-cell genomics reveals convergent subclonal evolution of resistance as early-stage breast cancer patients progress on endocrine plus CDK4/6 the
GSE158866	33208554	11/20/20	Single-Cell Omics Analysis Reveals Functional Diversification Of Hepatocytes During Liver Regeneration.
GSE159107	33393903	1/5/21	A Cell Atlas Of The Chick Retina Based On Single-Cell Transcriptomics.
GSE159354	33294861	12/10/20	Osteopontin Links Myeloid Activation And Disease Progression In Systemic Sclerosis.
GSE159599	33197070	11/17/20	Characterization Of Sheep Spermatogenesis Through Single-Cell Rna Sequencing.
GSE159759	33665640	3/6/21	Single-Cell Analysis Shows That Adipose Tissue Of Persons With Both Hiv And Diabetes Is Enriched For Clonal, Cytotoxic, And Cmv-Specific Cd4+ T¬†Cells.
GSE159843	33703914	3/12/21	Mir503Hg Loss Promotes Endothelial-To-Mesenchymal Transition In Vascular Disease.
GSE159929	33287869	12/9/20	Single-Cell Transcriptome Profiling Of An Adult Human Cell Atlas Of 15 Major Organs.
GSE160251		11/12/20	Multi-Modal Profiling of Human Fetal Liver-Derived Hematopoietic Stem Cells Reveals the Molecular Signature of Engraftment Potential
GSE160384	33198269	11/18/20	Methamphetamine Increases The Proportion Of Siv-Infected Microglia/Macrophages, Alters Metabolic Pathways, And Elevates Cell Death Pathways: A Sing
GSE160400	33151910	11/6/20	Dynamic Single-Cell Rna Sequencing Identifies Immunotherapy Persister Cells Following Pd-1 Blockade.
GSE160536	33168063	11/11/20	Single-Cell Transcriptome Conservation In A Comparative Analysis Of Fresh And Cryopreserved Human Skin Tissue: Pilot In Localized Scleroderma.
GSE160753	33158814	11/8/20	Enhanced Efficacy Of Simultaneous Pd-1 And Pd-L1 Immune Checkpoint Blockade In High-Grade Serous Ovarian Cancer.
GSE160754	33158814	11/8/20	Enhanced Efficacy Of Simultaneous Pd-1 And Pd-L1 Immune Checkpoint Blockade In High-Grade Serous Ovarian Cancer.
GSE160766	33674303	3/7/21	Single-Cell Transcriptomic Analysis Of Mihc Images Via Antigen Mapping.
GSE161089	32766588	8/9/20	Direct Exposure To Sars-Cov-2 And Cigarette Smoke Increases Infection Severity And Alters The Stem Cell-Derived Airway Repair Response.
GSE161617	33453151	1/17/21	Single-Cell Analysis Of The Developing Human Testis Reveals Somatic Niche Cell Specification And Fetal Germline Stem Cell Establishment.
GSE161918	33713619	4/1/21	Time-resolved systems immunology reveals a late juncture linked to fatal COVID-19
GSE161934	33283287	12/8/20	An Organoid-Derived Bronchioalveolar Model For Sars-Cov-2 Infection Of Human Alveolar Type Ii-Like Cells.
GSE162025	33531485	2/4/21	Tumour Heterogeneity And Intercellular Networks Of Nasopharyngeal Carcinoma At Single Cell Resolution.
GSE162121	33555999	2/9/21	Single-Cell Transcriptomes Of Developing And Adult Olfactory Receptor Neurons In
GSE162621	33589525	2/17/21	Therapeutic depletion of CCR8+ tumor-infiltrating regulatory T cells elicits antitumor immunity and synergizes with anti-PD-1 therapy
GSE162692	33646886	3/2/21	Strategies To Identify Mesenchymal Stromal Cells In Minimally Manipulated Human Bone Marrow Aspirate Concentrate Lack Consensus.
GSE162726	31710321	11/12/19	Single-Cell Rna-Sequencing Of Migratory Breast Cancer Cells: Discovering Genes Associated With Cancer Metastasis.
GSE162806	33320841	12/16/20	A Road Map From Single-Cell Transcriptome To Patient Classification For The Immune Response To Trauma.
GSE162952	33275120	12/5/20	Similarities Between Bovine And Human Germline Development Revealed By Single-Cell Rna Sequencing.
GSE163029	33538002	2/5/21	Single-Cell Transcriptomes Of Mouse Bladder Urothelium Uncover Novel Cell Type Markers And Urothelial Differentiation Characteristics.
GSE163120	33782623	3/31/21	Single-Cell Profiling Of Myeloid Cells In Glioblastoma Across Species And Disease Stage Reveals Macrophage Competition And Specialization.
GSE163792	33472597	1/22/21	Systematic Comparison Of High-Throughput Single-Cell Rna-Seq Methods For Immune Cell Profiling.
GSE164241	34129837	6/14/21	Human oral mucosa cell atlas reveals a stromal-neutrophil axis regulating tissue immunity
GSE164378	34062119	6/2/21	Integrated Analysis Of Multimodal Single-Cell Data.
GSE164898	33763657	3/26/21	A Single-Cell Atlas Of The Healthy Breast Tissues Reveals Clinically Relevant Clusters Of Breast Epithelial Cells.
GSE165193	33758834	3/25/21	Single-Cell Immunophenotyping Of The Fetal Immune Response To Maternal Sars-Cov-2 Infection In Late Gestation.
GSE165552	33577798	2/13/21	Targeting Primary And Metastatic Uveal Melanoma With A G Protein Inhibitor.
GSE165555		2/16/21	Single-cell analysis of the ventricular-subventricular zone reveals signatures of dorsal and ventral adult neurogenic lineages
GSE165880		2/9/21	Single cell transcriptomic analysis reveals cellular diversity of murine esophageal epithelium and age-associated mitochondrial dysfunction
GSE165917	33712605	3/14/21	Pgc1/Ppar Drive Cardiomyocyte Maturation At Single Cell Level Via Yap1 And Sf3B2.
GSE166452	33587034	2/16/21	A Œí-Catenin-Driven Switch In Tcf/Lef Transcription Factor Binding To Dna Target Sites Promotes Commitment Of Mammalian Nephron Progenitor Cells.
GSE166766	33730024	3/18/21	Single-Cell Longitudinal Analysis Of Sars-Cov-2 Infection In Human Airway Epithelium Identifies Target Cells, Alterations In Gene Expression, And Cell State
GSE171524	33915568	4/30/21	A Molecular Single-Cell Lung Atlas Of Lethal Covid-19.
GSE52529	24658644	3/25/14	The Dynamics And Regulators Of Cell Fate Decisions Are Revealed By Pseudotemporal Ordering Of Single Cells.
GSE54006	24531970	2/18/14	Massively Parallel Single-Cell Rna-Seq For Marker-Free Decomposition Of Tissues Into Cell Types.
GSE54695	24747814	4/22/14	Validation Of Noise Models For Single-Cell Transcriptomics.
GSE57249	25096407	8/7/14	Cell Fate Inclination Within 2-Cell And 4-Cell Mouse Embryos Revealed By Single-Cell Rna Sequencing.
GSE57872	24925914	6/14/14	Single-Cell Rna-Seq Highlights Intratumoral Heterogeneity In Primary Glioblastoma.
GSE59129	25053437	7/24/14	Single Cell Dissection Of Early Kidney Development: Multilineage Priming.

GSE59130	25053437	7/24/14	Single Cell Dissection Of Early Kidney Development: Multilineage Priming.
GSE60297	25224068	9/17/14	Population And Single-Cell Genomics Reveal The Aire Dependency, Relief From Polycomb Silencing, And Distribution Of Self-Antigen Expression In Thymic E
GSE60361	25700174	2/24/15	Brain Structure. Cell Types In The Mouse Cortex And Hippocampus Revealed By Single-Cell Rna-Seq.
GSE60768	26040288	6/5/15	Single-Cell Polyadenylation Site Mapping Reveals 3' Isoform Choice Variability.
GSE60781	26054720	6/10/15	Identification Of Cdc1- And Cdc2-Committed Dc Progenitors Reveals Early Lineage Priming At The Common Dc Progenitor Stage In The Bone Marrow.
GSE61288	26000486	5/23/15	Single-Cell Transcriptome Analyses Reveal Signals To Activate Dormant Neural Stem Cells.
GSE63472	26000488	5/23/15	Highly Parallel Genome-Wide Expression Profiling Of Individual Cells Using Nanoliter Droplets.
GSE63576	26691752	12/23/15	Somatosensory Neuron Types Identified By High-Coverage Single-Cell Rna-Sequencing And Functional Heterogeneity.
GSE64016	26301841	8/25/15	Oscope Identifies Oscillatory Genes In Unsynchronized Single-Cell Rna-Seq Experiments.
GSE65529	26343579	9/8/15	Pathogen Cell-To-Cell Variability Drives Heterogeneity In Host Immune Responses.
GSE65924	27003939	3/24/16	Spatial Transcriptome For The Molecular Annotation Of Lineage Fates And Cell Identity In Mid-Gastrula Mouse Embryo.
GSE66688	25867923	4/14/15	Spatial Reconstruction Of Single-Cell Gene Expression Data.
GSE67310	27281220	6/10/16	Dissecting Direct Reprogramming From Fibroblast To Neuron Using Single-Cell Rna-Seq.
GSE67602	27641957	9/20/16	Single-Cell Transcriptomics Reveals That Differentiation And Spatial Signatures Shape Epidermal And Hair Follicle Heterogeneity.
GSE67833	26235341	8/4/15	Single-Cell Transcriptomics Reveals A Population Of Dormant Neural Stem Cells That Become Activated Upon Brain Injury.
GSE67980	26383955	9/19/15	Rna-Seq Of Single Prostate Ctcs Implicates Noncanonical Wnt Signaling In Antiandrogen Resistance.
GSE69405	26084335	6/19/15	Single-Cell Mrna Sequencing Identifies Subclonal Heterogeneity In Anti-Cancer Drug Responses Of Lung Adenocarcinoma Cells.
GSE70236	27580035	9/1/16	Single-Cell Analysis Of Mixed-Lineage States Leading To A Binary Cell Fate Choice.
GSE70239	27580035	9/1/16	Single-Cell Analysis Of Mixed-Lineage States Leading To A Binary Cell Fate Choice.
GSE70240	27580035	9/1/16	Single-Cell Analysis Of Mixed-Lineage States Leading To A Binary Cell Fate Choice.
GSE70241	27580035	9/1/16	Single-Cell Analysis Of Mixed-Lineage States Leading To A Binary Cell Fate Choice.
GSE70242	27580035	9/1/16	Single-Cell Analysis Of Mixed-Lineage States Leading To A Binary Cell Fate Choice.
GSE70243	27580035	9/1/16	Single-Cell Analysis Of Mixed-Lineage States Leading To A Binary Cell Fate Choice.
GSE70244	27580035	9/1/16	Single-Cell Analysis Of Mixed-Lineage States Leading To A Binary Cell Fate Choice.
GSE70630	27806376	11/5/16	Single-Cell Rna-Seq Supports A Developmental Hierarchy In Human Oligodendroglioma.
GSE70657	27009448	3/25/16	Single-Cell Rna Sequencing Reveals Molecular And Functional Platelet Bias Of Aged Haematopoietic Stem Cells.
GSE70798	26237550	8/4/15	Aire Controls Gene Expression In The Thymic Epithelium With Ordered Stochasticity.
GSE71315	27081004	4/16/16	Single-Cell Analysis Of Long Non-Coding Rnas In The Developing Human Neocortex.
GSE71453	27558660	8/26/16	Single-Cell Rna-Seq Reveals Distinct Injury Responses In Different Types Of Drg Sensory Neurons.
GSE71982	26469390	10/16/15	Single-Cell Rna-Seq Resolves Cellular Complexity In Sensory Organs From The Neonatal Inner Ear.
GSE72056	27124452	4/29/16	Dissecting The Multicellular Ecosystem Of Metastatic Melanoma By Single-Cell Rna-Seq.
GSE72857	26627738	12/3/15	Transcriptional Heterogeneity And Lineage Commitment In Myeloid Progenitors.
GSE74207	27263970	6/7/16	A Primate Lncrna Mediates Notch Signaling During Neuronal Development By Sequestering Mirna.
GSE74534	26752769	1/12/16	Parallel Single-Cell Sequencing Links Transcriptional And Epigenetic Heterogeneity.
GSE74923	26732280	1/7/16	A Microfluidic Platform Enabling Single-Cell Rna-Seq Of Multigenerational Lineages.
GSE75140	26644564	12/9/15	Human Cerebral Organoids Recapitulate Gene Expression Programs Of Fetal Neocortex Development.
GSE75330	27284195	6/11/16	Oligodendrocyte Heterogeneity In The Mouse Juvenile And Adult Central Nervous System.
GSE75367	27556950	8/25/16	Her2 Expression Identifies Dynamic Functional States Within Circulating Breast Cancer Cells.
GSE75386	27531958	8/18/16	Single-Cell Rnaseq Reveals Cell Adhesion Molecule Profiles In Electrophysiologically Defined Neurons.
GSE75413	26541607	11/7/15	Single-Cell Transcriptomics Reveals Receptor Transformations During Olfactory Neurogenesis.
GSE75688	28474673	5/6/17	Single-Cell Rna-Seq Enables Comprehensive Tumour And Immune Cell Profiling In Primary Breast Cancer.
GSE75790	28212749	2/19/17	Comparative Analysis Of Single-Cell Rna Sequencing Methods.
GSE75804	26804902	1/26/16	Serum-Based Culture Conditions Provoke Gene Expression Variability In Mouse Embryonic Stem Cells As Revealed By Single-Cell Analysis.
GSE76005	26780092	1/19/16	Characterizing Transcriptional Heterogeneity Through Pathway And Gene Set Overdispersion Analysis.
GSE76157	27160914	5/11/16	Systematic Reconstruction Of Molecular Cascades Regulating Gp Development Using Single-Cell Rna-Seq.
GSE76312	28504724	5/16/17	Single-Cell Transcriptomics Uncovers Distinct Molecular Signatures Of Stem Cells In Chronic Myeloid Leukemia.
GSE76483	27150361	5/7/16	Simultaneous Profiling Of Transcriptome And Dna Methylome From A Single Cell.
GSE77740	27811054	11/5/16	Progressive Alterations In Multipotent Hematopoietic Progenitors Underlie Lymphoid Cell Loss In Aging.
GSE77847	27016502	3/27/16	Dnmt3A Haploinsufficiency Transforms Flt3Itd Myeloproliferative Disease Into A Rapid, Spontaneous, And Fully Penetrant Acute Myeloid Leukemia.
GSE77980	26951663	3/10/16	Use Of The Fluidigm C1 Platform For Rna Sequencing Of Single Mouse Pancreatic Islet Cells.
GSE78779	27121950	4/29/16	Cel-Seq2: Sensitive Highly-Multiplexed Single-Cell Rna-Seq.
GSE78907	27580035	9/1/16	Single-Cell Analysis Of Mixed-Lineage States Leading To A Binary Cell Fate Choice.
GSE79280	29760424	5/16/18	Parental Haplotype-Specific Single-Cell Transcriptomics Reveal Incomplete Epigenetic Reprogramming In Human Female Germ Cells.
GSE79363	27841856	11/15/16	Single-Cell Rna-Seq Ties Macrophage Polarization To Growth Rate Of Intracellular Salmonella.
GSE79818	27338705	6/25/16	Microglia Development Follows A Stepwise Program To Regulate Brain Homeostasis.
GSE80032	27923766	12/8/16	Single-Cell Analysis Uncovers Clonal Acinar Cell Heterogeneity In The Adult Pancreas.
GSE81076	27345837	6/28/16	De Novo Prediction Of Stem Cell Identity Using Single-Cell Transcriptome Data.
GSE81547	28965763	10/3/17	Single-Cell Analysis Of Human Pancreas Reveals Transcriptional Signatures Of Aging And Somatic Mutation Patterns.
GSE81682	27365425	7/2/16	A Single-Cell Resolution Map Of Mouse Hematopoietic Stem And Progenitor Cell Differentiation.
GSE81812	31638164	10/23/19	Population And Single‚ÄëCell Transcriptome Analyses Reveal Diverse Transcriptional Changes Associated With Radioresistance In Esophageal Squamous Ce
GSE82187	27425622	7/19/16	Cellular Taxonomy Of The Mouse Striatum As Revealed By Single-Cell Rna-Seq.
GSE83139	27364731	7/2/16	Single-Cell Transcriptomics Of The Human Endocrine Pancreas.
GSE84133	27667365	10/28/16	A Single-Cell Transcriptomic Map Of The Human And Mouse Pancreas Reveals Inter- And Intra-Cell Population Structure.
GSE84465	29091775	11/2/17	Single-Cell Rna-Seq Analysis Of Infiltrating Neoplastic Cells At The Migrating Front Of Human Glioblastoma.
GSE84498	28166538	2/7/17	Single-Cell Spatial Reconstruction Reveals Global Division Of Labour In The Mammalian Liver.
GSE85152	27545347	8/23/16	The Spectrum And Regulatory Landscape Of Intestinal Innate Lymphoid Cells Are Shaped By The Microbiome.
GSE85234	29187729	12/1/17	Inference Of Differentiation Time For Single Cell Transcriptomes Using Cell Population Reference Data.
GSE85241	27693023	10/28/16	A Single-Cell Transcriptome Atlas Of The Human Pancreas.
GSE85527	28664195	7/1/17	Partial Exhaustion Of Cd8 T Cells And Clinical Response To Teplizumab In New-Onset Type 1 Diabetes.
GSE85534	28249587	3/3/17	Single-Cell Transcriptome Conservation In Cryopreserved Cells And Tissues.
GSE85875	28135720	1/31/17	Identity And Dynamics Of Mammary Stem Cells During Branching Morphogenesis.
GSE86153	28445462	4/27/17	Cell Diversity And Network Dynamics In Photosensitive Human Brain Organoids.
GSE86310	28431249	4/22/17	Macrophages Facilitate Electrical Conduction In The Heart.
GSE86469	27864352	11/20/16	Single-Cell Transcriptomes Identify Human Islet Cell Signatures And Reveal Cell-Type-Specific Expression Changes In Type 2 Diabetes.
GSE86618	27942595	12/13/16	Single-Cell Rna Sequencing Identifies Diverse Roles Of Epithelial Cells In Idiopathic Pulmonary Fibrosis.
GSE87375	28467935	5/4/17	Deciphering Pancreatic Islet Œí Cell And Œë Cell Maturation Pathways And Characteristic Features At The Single-Cell Level.
GSE87527	27863245	11/20/16	Epigenetic Memory Underlies Cell-Autonomous Heterogeneous Behavior Of Hematopoietic Stem Cells.
GSE87544	28355573	3/30/17	Single-Cell Rna-Seq Reveals Hypothalamic Cell Diversity.
GSE87849	28061811	1/8/17	The Nature And Nurture Of Cell Heterogeneity: Accounting For Macrophage Gene-Environment Interactions With Single-Cell Rna-Seq.
GSE89232	27864467	11/20/16	Human Dendritic Cells (Dcs) Are Derived From Distinct Circulating Precursors That Are Precommitted To Become Cd1C+ Or Cd141+ Dcs.
GSE89236	27940562	12/13/16	A Cost Effective 5ŒÑ Selective Single Cell Transcriptome Profiling Approach With Improved Umi Design.
GSE89237	27940562	12/13/16	A Cost Effective 5ŒÑ Selective Single Cell Transcriptome Profiling Approach With Improved Umi Design.
GSE89497	30042384	7/26/18	Single-Cell Rna-Seq Reveals The Diversity Of Trophoblast Subtypes And Patterns Of Differentiation In The Human Placenta.
GSE89567	28360267	4/1/17	Decoupling Genetics, Lineages, And Microenvironment In Idh-Mutant Gliomas By Single-Cell Rna-Seq.
GSE89910	29678445	4/22/18	Spatial Patterns Of Gene Expression Are Unveiled In The Chick Primitive Streak By Ordering Single-Cell Transcriptomes.
GSE90546	27984733	12/17/16	A Multiplexed Single-Cell Crispr Screening Platform Enables Systematic Dissection Of The Unfolded Protein Response.
GSE90856	28285904	3/14/17	Mex3A Marks A Slowly Dividing Subpopulation Of Lgr5+ Intestinal Stem Cells.
GSE90860	28134272	1/31/17	Transcriptomic And Anatomic Parcellation Of 5-Ht
GSE92522	28942923	9/26/17	Transcriptional Architecture Of Synaptic Communication Delineates Gabaergic Neuron Identity.
GSE92707	28462073	5/4/17	Heterogeneity Of Hypothalamic Pro-Opiomelanocortin-Expressing Neurons Revealed By Single-Cell Rna Sequencing.
GSE92842	29174332	11/28/17	Injury Induces Endogenous Reprogramming And Dedifferentiation Of Neuronal Progenitors To Multipotency.
GSE92872	28099430	1/19/17	Pooled Crispr Screening With Single-Cell Transcriptome Readout.
GSE93321	28445465	4/27/17	Assembly Of Functionally Integrated Human Forebrain Spheroids.
GSE93374	28166221	2/7/17	A Molecular Census Of Arcuate Hypothalamus And Median Eminence Cell Types.

GSE93524	28826820	8/23/17	Antagonistic Activities Of Sox2 And Brachyury Control The Fate Choice Of Neuro-Mesodermal Progenitors.
GSE93593	28279351	3/11/17	Single-Cell Profiling Of An In¬†Vitro Model Of Human Interneuron Development Reveals Temporal Dynamics Of Cell Type Production And Maturation.
GSE93811	28445465	4/27/17	Assembly Of Functionally Integrated Human Forebrain Spheroids.
GSE94333	28851704	8/31/17	Psychrophilic Proteases Dramatically Reduce Single-Cell Rna-Seq Artifacts: A Molecular Atlas Of Kidney Development.
GSE94383	28396000	4/12/17	Measuring Signaling And Rna-Seq In The Same Cell Links Gene Expression To Dynamic Patterns Of Nf-Œöb Activation.
GSE95194	30078729	8/7/18	Transcriptional Convergence Of Oligodendrocyte Lineage Progenitors During Development.
GSE95432	29158510	11/22/17	Construction Of Developmental Lineage Relationships In The Mouse Mammary Gland By Single-Cell Rna Profiling.
GSE95445	29158510	11/22/17	Construction Of Developmental Lineage Relationships In The Mouse Mammary Gland By Single-Cell Rna Profiling.
GSE95446	30270042	10/3/18	Structural Remodeling Of The Human Colonic Mesenchyme In Inflammatory Bowel Disease.
GSE95448	29158510	11/22/17	Construction Of Developmental Lineage Relationships In The Mouse Mammary Gland By Single-Cell Rna Profiling.
GSE95450	30270042	10/3/18	Structural Remodeling Of The Human Colonic Mesenchyme In Inflammatory Bowel Disease.
GSE95630	29802404	5/29/18	Tracing The Temporal-Spatial Transcriptome Landscapes Of The Human Fetal Digestive Tract Using Single-Cell Rna-Sequencing.
GSE95837	28445465	4/27/17	Assembly Of Functionally Integrated Human Forebrain Spheroids.
GSE96106	28463226	5/4/17	Prospective Isolation Of Nkx2-1-Expressing Human Lung Progenitors Derived From Pluripotent Stem Cells.
GSE96562	28566371	6/2/17	Single-Cell Rna Sequencing Reveals Expanded Clones Of Islet Antigen-Reactive Cd4
GSE96564	28566371	6/2/17	Single-Cell Rna Sequencing Reveals Expanded Clones Of Islet Antigen-Reactive Cd4
GSE96568	28566371	6/2/17	Single-Cell Rna Sequencing Reveals Expanded Clones Of Islet Antigen-Reactive Cd4
GSE96958	29555020	3/21/18	Single-Cell Transcriptomics Reveals A New Dynamical Function Of Transcription Factors During Embryonic Hematopoiesis.
GSE97168	28475900	5/6/17	Innate Immune Landscape In Early Lung Adenocarcinoma By Paired Single-Cell Analyses.
GSE97478	30134177	8/23/18	Diversity Of Interneurons In The Dorsal Striatum Revealed By Single-Cell Rna Sequencing And Patchseq.
GSE97519	29425512	2/10/18	Deciphering Cell Lineage Specification During Male Sex Determination With Single-Cell Rna Sequencing.
GSE97564	28438991	4/26/17	Differentiation Of V2A Interneurons From Human Pluripotent Stem Cells.
GSE97849	29857010	6/2/18	Deconstructive Somatic Cell Nuclear Transfer Reveals Novel Regulatory T-Cell Subsets.
GSE97941	28650342	6/27/17	Inflammatory Ly6Chi Monocytes And Their Conversion To M2 Macrophages Drive Atherosclerosis Regression.
GSE98048	28408401	4/15/17	Synergistic Immunostimulatory Effects And Therapeutic Benefit Of Combined Histone Deacetylase And Bromodomain Inhibition In Non-Small Cell Lung Can
GSE98415	28768198	8/3/17	Enhanced Neuronal Regeneration In The Cast/Ei Mouse Strain Is Linked To Expression Of Differentiation Markers After Injury.
GSE98451	28625536	6/20/17	Reconstructing Lineage Hierarchies Of Mouse Uterus Epithelial Development Using Single-Cell Analysis.
GSE98638	28622514	6/18/17	Landscape Of Infiltrating T Cells In Liver Cancer Revealed By Single-Cell Sequencing.
GSE98664	29434199	2/13/18	Single-Cell Full-Length Total Rna Sequencing Uncovers Dynamics Of Recursive Splicing And Enhancer Rnas.
GSE98816	29443965	2/15/18	A Molecular Atlas Of Cell Types And Zonation In The Brain Vasculature.
GSE98852	29408715	2/7/18	Single-Cell Profiling Of Peanut-Responsive T Cells In Patients With Peanut Allergy Reveals Heterogeneous Effector T
GSE99058	29443965	2/15/18	A Molecular Atlas Of Cell Types And Zonation In The Brain Vasculature.
GSE99095	29030335	10/17/17	Single-Cell Rna-Seq Reveals A Distinct Transcriptome Signature Of Aneuploid Hematopoietic Cells.
GSE99235	29443965	2/15/18	A Molecular Atlas Of Cell Types And Zonation In The Brain Vasculature.
GSE99254	29942094	6/27/18	Global Characterization Of T Cells In Non-Small-Cell Lung Cancer By Single-Cell Sequencing.
GSE99305	28922540	9/19/17	Ape1/Ref-1 Knockdown In Pancreatic Ductal Adenocarcinoma - Characterizing Gene Expression Changes And Identifying Novel Pathways Using Single-Cell R
GSE99701	29089413	11/2/17	Transcriptomes Of Major Renal Collecting Duct Cell Types In Mouse Identified By Single-Cell Rna-Seq.
GSE99735	28671690	7/4/17	Skewing Of The Population Balance Of Lymphoid And Myeloid Cells By Secreted And Intracellular Osteopontin.
GSE99915	30518857	12/7/18	Single-Cell Mapping Of Lineage And Identity In Direct Reprogramming.
GSE99989	29334988	1/18/18	Epidermal Wnt Signalling Regulates Transcriptome Heterogeneity And Proliferative Fate In Neighbouring Cells.
HCA-abe1a013	31604275	10/12/19	Spatiotemporal Immune Zonation Of The Human Kidney.
HRA000150	32788748	8/14/20	Single-Cell Landscape Of Immunological Responses In Patients With Covid-19.
NEMO-DAT-0R	33723434	3/17/21	Single-Cell Atlas Of Early Human Brain Development Highlights Heterogeneity Of Human Neuroepithelial Cells And Early Radial Glia.
NEMO-DAT-JB	34004146	5/19/21	A Taxonomy Of Transcriptomic Cell Types Across The Isocortex And Hippocampal Formation.
PHS000424-V8	28846088	8/29/17	Massively Parallel Single-Nucleus Rna-Seq With Dronc-Seq.
PRJEB31843	31892341	1/2/20	Scrna-Seq Assessment Of The Human Lung, Spleen, And Esophagus Tissue Stability After Cold Preservation.
PRJNA434002	31097668	5/18/19	Single-Cell Genomics Identifies Cell Type-Specific Molecular Changes In Autism.
PRJNA438862	30096314	8/11/18	Molecular Architecture Of The Mouse Nervous System.
PRJNA544731	31316211	7/19/19	Neuronal Vulnerability And Multilineage Diversity In Multiple Sclerosis.
PRJNA637987	34321664	7/28/21	Molecular architecture of the developing mouse brain
SCP253	30135581	8/24/18	Allergic Inflammatory Memory In Human Respiratory Epithelial Progenitor Cells.
SCP256	32251406	4/7/20	Integrated Single-Cell Analysis Of Multicellular Immune Dynamics During Hyperacute Hiv-1 Infection.
SCP259	31348891	7/28/19	Intra- And Inter-Cellular Rewiring Of The Human Colon During Ulcerative Colitis.
SCP806	32413319	5/28/20	SARS-CoV-2 Receptor ACE2 Is an Interferon-Stimulated Gene in Human Airway Epithelial Cells and Is Detected in Specific Cell Subsets across Tissues
TSP1-2	35549404	5/13/22	The Tabula Sapiens: A multiple-organ, single-cell transcriptomic atlas of humans
