# Supplementary material for: Interpretable and context-free deconvolution of multi-scale whole transcriptomic data with UniCell deconvolve
Source: Nat Commun. 2023 Mar 11;14:1350. doi: 10.1038/s41467-023-36961-8 (PMC10008582; doi:10.1038/s41467-023-36961-8)
Supplement: Supplementary file 2 — Reporting Summary [file 41467_2023_36961_MOESM2_ESM.pdf]

nature portfolio

Corresponding author(s): Robert P Sebra

Last updated by author(s): Feb 11, 2023

Reporting Summary

Nature Portfolio wishes to improve the reproducibility of the work that we publish. This form provides structure for consistency and transparency in reporting. For further information on Nature Portfolio policies, see our Editorial Policies and the Editorial Policy Checklist.

Statistics

For all statistical analyses, confirm that the following items are present in the figure legend, table legend, main text, or Methods section.

n/a	Confirmed	
☐	☒	The exact sample size (*n*) for each experimental group/condition, given as a discrete number and unit of measurement
☐	☒	A statement on whether measurements were taken from distinct samples or whether the same sample was measured repeatedly
☐	☒	The statistical test(s) used AND whether they are one- or two-sided<br>*Only common tests should be described solely by name; describe more complex techniques in the Methods section.*
☐	☒	A description of all covariates tested
☐	☒	A description of any assumptions or corrections, such as tests of normality and adjustment for multiple comparisons
☐	☒	A full description of the statistical parameters including central tendency (e.g. means) or other basic estimates (e.g. regression coefficient) AND variation (e.g. standard deviation) or associated estimates of uncertainty (e.g. confidence intervals)
☐	☒	For null hypothesis testing, the test statistic (e.g. *F*, *t*, *r*) with confidence intervals, effect sizes, degrees of freedom and *P* value noted<br>*Give P values as exact values whenever suitable.*
☒	☐	For Bayesian analysis, information on the choice of priors and Markov chain Monte Carlo settings
☐	☒	For hierarchical and complex designs, identification of the appropriate level for tests and full reporting of outcomes
☐	☒	Estimates of effect sizes (e.g. Cohen's *d*, Pearson's *r*), indicating how they were calculated

Our web collection on statistics for biologists contains articles on many of the points above.

Software and code

Policy information about availability of computer code

Data collection	Data generated for this manuscript (scRNA-Seq Fig 6) was processed through the CellRanger v6.1.0 pipeline.
Data analysis	The UniCell Deconvolve user API is publically available as a Python package and can be found at https://github.com/dchary/ucdeconvolve/. Documentation and tutorials are available at https://ucdeconvolve.readthedocs.io/en/latest/. All jupyter notebooks used to collect, analyze, and visualize results presented in this manuscript, with an associated list of all external software libraries and corresponding versions, are publicly available at https://github.com/dchary/ucdeconvolve_paper and upon request.<br><br>Specific package versions used for analysis are as follows (python):<br><br>anndata 0.8.0<br>combat 0.3.3<br>h5py 3.1.0<br>harmonypy 0.0.5<br>keras 2.6.0<br>jupyterlab 3.2.9<br>leidenalg 0.8.9<br>matplotlib 3.5.1<br>numba 0.55.2<br>numpy 1.21.6<br>pandas 1.3.5

pynndescent 0.5.6
python
requests 2.28.1
scaden 1.1.2
scanpy 1.8.2
scipy 1.7.3
scvi-tools 0.15.1
seaborn 0.11.2
statannotations 0.4.4
statsmodels 0.13.2
tensorboard 2.6.0
tensorflow 2.6.3
tensorflow-datasets 4.3.0
textwrap3 0.9.2
umap-learn 0.5.2

Specific package versions used for analysis are as follows (R):

R 4.2.1
rstudio 2022.07.1+554
singlecellexperiment 1.20.0
summarizedexperiment 1.28.0
omnideconv 0.0.0.9000
music 1.0.0
spotlight 1.2.0
seuratobject 4.1.3
seurat 4.3.0
giotto 3.1
stdeconvolve 1.3.1
spacexr 2.0.7

For manuscripts utilizing custom algorithms or software that are central to the research but not yet described in published literature, software must be made available to editors and reviewers. We strongly encourage code deposition in a community repository (e.g. GitHub). See the Nature Portfolio guidelines for submitting code & software for further information.

Data

Policy information about availability of data

All manuscripts must include a data availability statement. This statement should provide the following information, where applicable:

- Accession codes, unique identifiers, or web links for publicly available datasets
- A description of any restrictions on data availability
- For clinical datasets or third party data, please ensure that the statement adheres to our policy

Data generated for this manuscript (scRNA-Seq count matrices, used in Fig 6) is publically available at https://github.com/dchary/ucdeconvolve_paper. All accession codes for publicly available studies used as training data for the model presented in this study are listed in Supplementary Table 5. All previously published datasets used for benchmarking in this study are listed along with web links to source data in Supplementary Table 1, and are additionally made available at https://github.com/dchary/ucdeconvolve_paper.

Human research participants

Policy information about studies involving human research participants and Sex and Gender in Research.

Reporting on sex and gender	Reporting on sex and gender is not an applicable element of study design for this manuscript, as the work describes a novel computational method.
Population characteristics	Population characteristics are not an applicable element of study design for this manuscript.
Recruitment	Recruitment is not an applicable element of study design for this manuscript.
Ethics oversight	Ethics oversight is not an applicable element of study design for this manuscript.

Note that full information on the approval of the study protocol must also be provided in the manuscript.

Field-specific reporting

Please select the one below that is the best fit for your research. If you are not sure, read the appropriate sections before making your selection.

- [x] Life sciences
- [ ] Behavioural & social sciences
- [ ] Ecological, evolutionary & environmental sciences

For a reference copy of the document with all sections, see nature.com/documents/nr-reporting-summary-flat.pdf

Life sciences study design

All studies must disclose on these points even when the disclosure is negative.

Sample size	Experimentally, we profiled human biopsy samples acquired from the Mount Sinai Biorepository, in order to demonstrate the prospective utility of our computational tool. Two biopsy samples representing adjacent normal and suspected malignant components derived from the same patient showed sufficient levels of cellular viability and minimal debris upon dissociation, and were selected for downstreaming processing, sequencing and demonstrative analysis. For computational analysis of previously published datasets, no statistical methods were used to determine sample size, and all available samples were used as described and provided in the literature for each study.
Data exclusions	No data were excluded from the analysis.
Replication	Validation of our computational model was done through multiple in-silico approaches. Model performance during training was validated using a train-test split strategy, whereby data from the training set was intentionally held-out to compare deconvolution performance at test time. Uniformly preprocessed data sets derived from the same tissue source (i.e. PBMCs) using varying single cell technology platforms were used to validate our platform's robustness to technical variation (Supplementary Figure 2G). Computational platform performance and stability with respect to changes in input hyperparameters (i.e. gene dropout noise, cell type complexity, number of cells in mixture) was assessed and demonstrated in Supplementary Figure 2D. All attempts at validation were successful, and input conditions for optimal performance of the model are described in the manuscript text (see results, hyperparameter sensitivity analysis).
Randomization	Randomization is not an applicable element of our study because the objective was not an intervention trial.
Blinding	Blinding is not relevant or necessary to our study because the objective was not a comparison of two or more experimental interventions.

Reporting for specific materials, systems and methods

We require information from authors about some types of materials, experimental systems and methods used in many studies. Here, indicate whether each material, system or method listed is relevant to your study. If you are not sure if a list item applies to your research, read the appropriate section before selecting a response.

Materials & experimental systems

n/a	Involved in the study	
☒	☐	Antibodies
☒	☐	Eukaryotic cell lines
☒	☐	Palaeontology and archaeology
☒	☐	Animals and other organisms
☒	☐	Clinical data
☒	☐	Dual use research of concern

Methods

n/a	Involved in the study	
☒	☐	ChIP-seq
☒	☐	Flow cytometry
☒	☐	MRI-based neuroimaging
