# Supplementary material for: Interpretable and context-free deconvolution of multi-scale whole transcriptomic data with UniCell deconvolve
Source: Nat Commun. 2023 Mar 11;14:1350. doi: 10.1038/s41467-023-36961-8 (PMC10008582; doi:10.1038/s41467-023-36961-8)
Supplement: Supplementary file 4 — Description of Additional Supplementary Files [file 41467_2023_36961_MOESM4_ESM.pdf]

Title: Supplementary Software

Description: This legend contains basic information about each script included in the supplementary software file. The software file is broken up by major figures. Scripts used to generate panels in primary manuscript figures are provided in the *figure<x>_primary.ipynb* files, while scripts used to generate panels for supplementary figures are provided by *figure<x>_supplementary.ipynb*, where supplementary information was organized with respect to supporting information for a given primary figure.

Item	File	Description of File Contents
Figure 1 Primary	figure1/figure1_primar.ipynb	Analysis code used to generate plots shown in figure 1.
Figure 2 Primary Lung	figure2/figure2_primary_lung.ipynb	Analysis code used to generate benchmarks for lung dataset.
Figure 2 Primary PBMC	figure2/figure2_primary_pbmc.ipynb	Analysis code used to generate benchmarks for PBMC dataset.
Figure 2 Primary Retina	figure2/figure2_primary_retina.ipynb	Analysis code used to generate benchmarks for retina dataset.
Figure 2 Primary Hippocampus	figure2/figure2_primary_hippocampus.ipynb	Analysis code used to generate benchmarks for hippocampus dataset.
Figure 2 Primary Comparators	figure2/figure2_primary_comparatorss.ipynb	Analysis code used to run benchmarks against comparator methods.
Figure 2 Supplementary	figure2/figure2_supplementary.ipynb	Code used to generate supplementary figures supporting benchmarking shown in Figure 2.
Figure 3 Primary	figure3/figure3_primary.ipynb	Analysis code used to generate plots shown in figure 3.
Figure 4 Primary	figure4/figure4_primary.ipynb	Analysis code used to generate plots shown in figure 4.
Figure 4 Supplementary	figure4/figure4_supplement.ipynb	Analysis code used to generate information for deconvolution of tumor microenvironment and cancer biology supporting information presented in primary figure 4.
Figure 5 Primary	figure5/figure5_primary.ipynb	Analysis code used to generate plots shown in figure 5.
Figure 6 Primary	figure5/figure5_primary.ipynb	Analysis code used to generate plots shown in figure 6.
Pseudo-Bulk Generator	scripts/pseudobulkgen.py	Python script used to generate pseudobulk mixtures for model training data.
Train Deconvolve	scripts/train_deconvolve.py	Script used to train deconvolution model.
LICENSE	LICENSE	GNU General Public License Version 3
Readme	README.md	Markdown readme file describing supplementary software.
Software Version List	software_version_list.txt	List of python packages used in the analysis environment and associated versions.
